# Regioselective *trans*‐Hydrostannation of Boron‐Capped Alkynes

**DOI:** 10.1002/chem.202101901

**Published:** 2021-08-04

**Authors:** Romain Melot, Tomas J. Saiegh, Alois Fürstner

**Affiliations:** ^1^ Max-Planck-Institut für Kohlenforschung 45470 Mülheim an der RuhrMülheim/Ruhr Germany

**Keywords:** alkynylboranes, boron heterocycles, *gem*-dimetallic alkenes, hydrostannation, ruthenium, *trans*-hydrometalation

## Abstract

Alkynyl‐B(aam) (aam=anthranilamidato) derivatives are readily available bench‐stable compounds that undergo remarkably selective reactions with Bu_3_SnH in the presence of [Cp*RuCl]_4_ as the catalyst. The addition follows a stereochemically unorthodox *trans*‐selective course; in terms of regioselectivity, the Bu_3_Sn‐ unit is delivered with high fidelity to the C‐atom of the triple bond adjacent to the boracyclic head group (“*alpha,trans*‐addition”). This outcome is deemed to reflect a hydrogen bonding interaction between the protic −NH groups of the benzo‐1,3,2‐diazaborininone ring system and the polarized [Ru−Cl] bond in the loaded catalyst, which locks the substrate in place in a favorable orientation relative to the incoming reagent. The resulting isomerically (almost) pure *gem*‐dimetalated building blocks are amenable to numerous downstream functionalizations; most remarkable is the ability to subject the −B(aam) moiety to Suzuki‐Miyaura cross coupling without need for prior hydrolysis while keeping the adjacent Bu_3_Sn‐ group intact. Alternatively, the tin residue can be engaged in selective tin/halogen exchange without touching the boron substituent; the fact that the two ‐NH entities of −B(aam) do not protonate organozinc reagents and hence do not interfere with Negishi reactions of the alkenyl halides thus formed is another virtue of this so far underutilized boracycle. Overall, the ruthenium catalyzed *trans*‐hydrostannation of alkynyl‐B(aam) derivatives opens a practical gateway to isomerically pure trisubstituted alkenes of many different substitution patterns by sequential functionalization of the 1‐alkenyl‐1,1‐heterobimetallic adducts primarily formed.

## Graphical abstract

**Peripheral**: A hydrogen‐bonding array in the periphery of the loaded ruthenium catalyst is the likely cause for the excellent regioselectivity observed in the hydrostannation of bench‐stable alkynyl‐B(aam) derivatives (aam=anthranilamido). The reaction follows a stereochemically unorthodox *trans*‐addition mode and delivers highly versatile *gem*‐dimetalated alkene building blocks for synthesis.

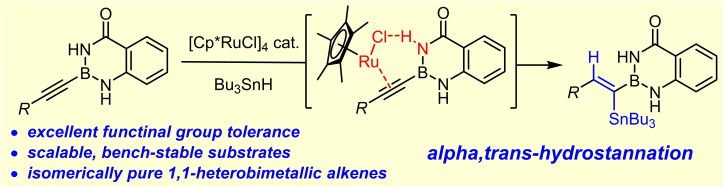

## Introduction

[Cp*Ru]‐based complexes are capable of catalyzing a variety of unusual addition reactions to alkynes that deviate from the conventional suprafacial *syn*‐selective course.^[1]^ The discovery of *trans*‐hydrosilylation marked the lead finding;[^2^, ^3^, ^4^, ^5^, ^6^] for its stereo‐complementary outcome, this transformation was rapidly embraced by the scientific community.[^7^, ^8^] Importantly, the underlying reactivity mode is not limited to *trans*‐hydrosilylation but was shown to be far more general: it is also manifested in *trans*‐hydrogenation and the intimately linked *gem*‐hydrogenation,[^9^, ^10^, ^11^, ^12^] in *trans*‐hydroboration,[^13^, ^14^] *trans*‐hydrogermylation,[^15^, ^16^] *trans*‐hydrostannation,[^16^, ^17^, ^18^] as well as *trans*‐hydroalkynylation and *trans*‐chloroalkylation of internal alkynes;^[19]^ all of these reactions have little, if any, precedent in the earlier literature. Combined experimental, spectroscopic and computational studies suggest that metallacyclopropene derivatives are the key reactive intermediates accountable for the unorthodox *trans*‐addition mode.[^1^, ^10a^, ^10b^, ^18^, ^20^, ^21^, ^22^, ^23^]

**Scheme 1:**
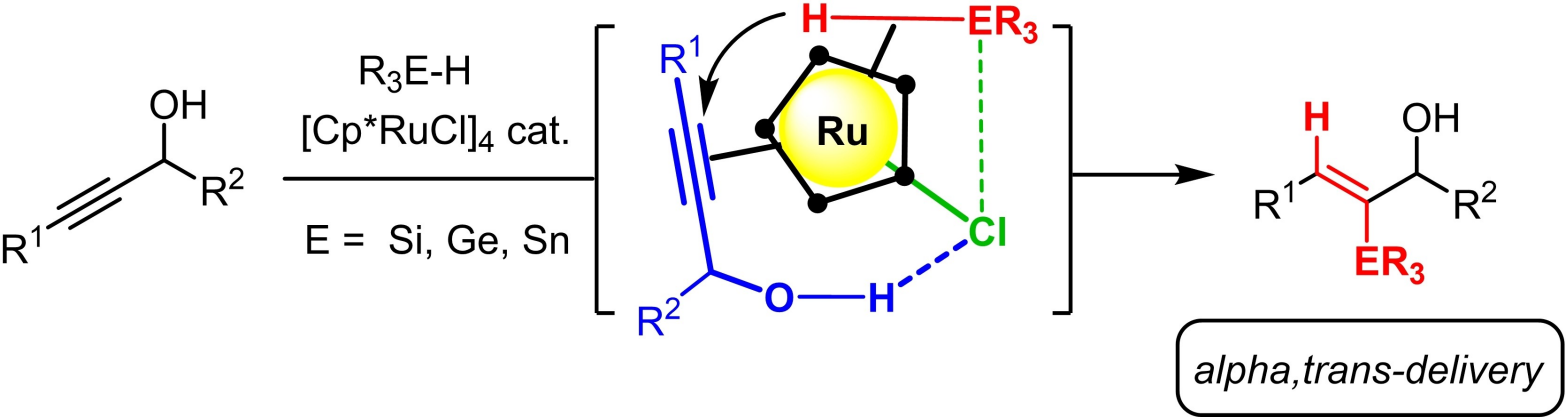
Regio‐ and stereoselective *trans*‐hydrometalation of propargyl alcohols; Newman‐type projection of the presumed loaded ruthenium catalyst with an interligand hydrogen bonding network; for the sake of clarity, • denotes the −CMe edges of the Cp* ring; Cp*=pentamethylcyclopentadienyl.

To render these reactions truly enabling, however, the regioselectivity of addition also needs to be controlled when working with unsymmetrical substrates.^[24]^ For an important subset of alkyne derivatives, this goal is easily reached: thus, propargyl alcohols succumb to highly selective delivery of the R_3_E‐ (E=Si, Ge, Sn) group to the proximal C‐atom, provided that [Cp*RuCl]_4_ is used as the catalyst.[^16^, ^17^, ^18^, ^25^] Hydrogen bonding between the −OH group and the polarized [Ru−Cl] unit locks the substrate in place; at the same time, the chloride ligand steers the incoming R_3_E−H reagent by an attractive interaction with the metalloid center (Scheme 1).[^16^, ^18^, ^19^] These synergetic interactions entail a highly ordered transition state that explains the usually excellent levels of *alpha,trans*‐delivery. The fidelity of this addition mode was showcased by applications to total‐ and/or diverted total syntheses of increasingly complex targets of biological significance.^[26]^

A priori, substrates other than (homo)propargyl alcohols carrying a protic substituent in proximity to the triple bond should follow the same path. So far, however, this option has been realized only for a limited number of propargyl amide and ‐sulfonamide derivatives,^[16]^ as well as for a few protic heterocycles (indole, imidazole, 2‐pyridone) carrying an appropriately placed alkyne substituent.^[18]^ Outlined below is an extension of the concept to an entirely different class of starting materials, namely alkynylboranes, which lead to very versatile 1‐alkenyl‐1,1‐heterobimetallic building blocks for synthesis[^27^, ^28^, ^29^] when subjected to ruthenium catalyzed *alpha,trans*‐hydrostannation.

## Results and Discussion

### Screening and reaction optimization

Heteroatom‐substituted alkynes tend to be less reactive in ruthenium catalyzed *trans*‐hydrogenation or *trans*‐hydrometalation reactions than “ordinary” internal alkynes;^[30}^ good results, however, were obtained in *trans*‐hydrostannations of silylated acetylene derivatives as well as 1‐chloroalkynes.^[17]^ Therefore it was mandatory to first check if alkynylboranes are amenable to such reactions at all. Indeed, the trifluoroborate derivatives **1 a**,**b** failed to react with Bu_3_SnH under standard conditions (Table 1); the analogous pinacolboronate **1 c** was transformed into a mixture of regio‐ and stereomers, with the *beta,trans*‐adduct as the major component.^[31]^ As the individual isomers proved difficult to separate, it was clear that better control over the course of the addition was mandatory to render this transformation useful.

**Table 1:** Evaluation of alkynyl boranes (borates) with different head groups.

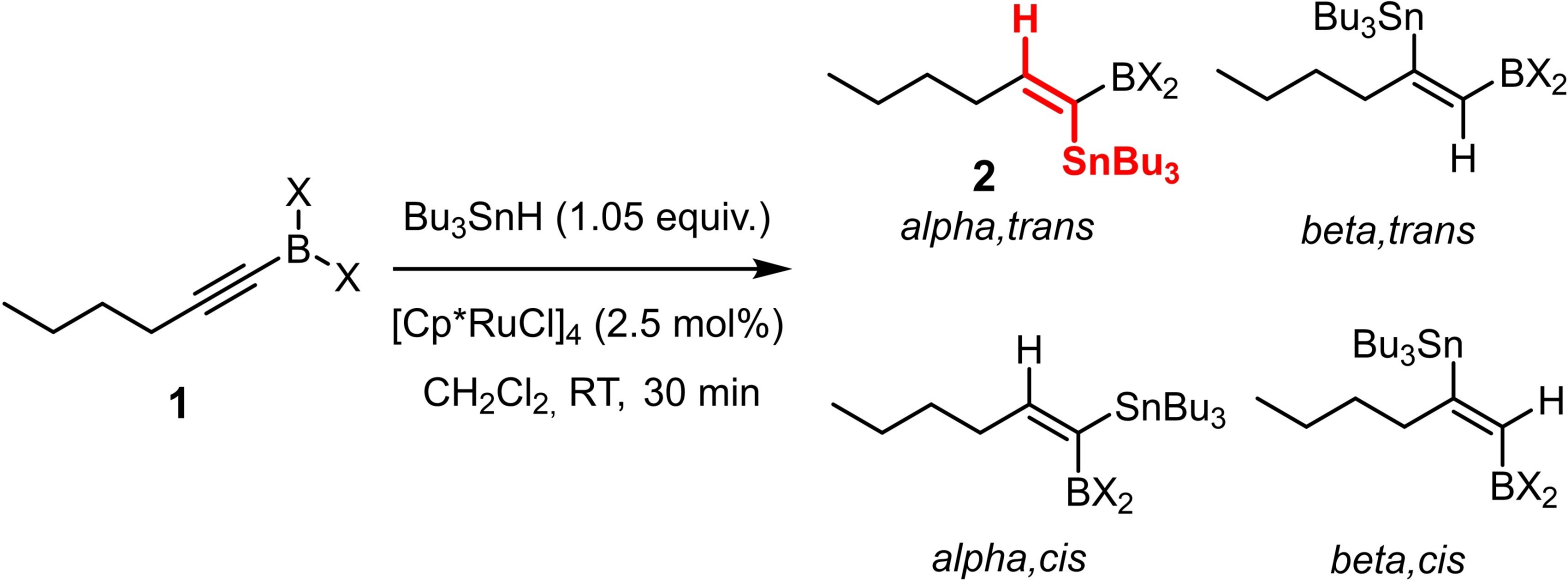				
Entry	Compound	‐BX_2_	Conversion (%)^[a]^	α,*trans*:β,*trans*:α,*cis*:β,*cis* ^[a]^
1	**1 a**	−BF_3_K	0	–
2	**1 b**	−BF_3_(NBu_4_)	0	–
3	**1 c**	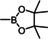	100	26 : 49:0 : 25
4	**1 d**	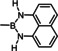	100	85 : 15
5	**1 e**	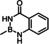	100 (88)^[b]^	96 : 4:(Σ<1)
6	**1 e**		80^[c]^	96 : 4:nd^[d]^
7	**1 f**	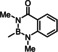	50	nd^[e]^
8	**1 g**	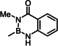	92	87 : 13:(Σ<1)
9	**1 h**	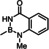	86	73 : 22:0 : 5
10	**1 i**	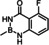	100	98 : 2:(Σ<1)
11	**1 j**	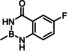	100	98 : 2:(Σ<1)

[a] Conversion and isomer ratios were determined by ^1^H NMR of the crude product after 30 min reaction time; [b] isolated yield in brackets; [c] with [Cp*Ru(MeCN)_3_]PF_6_ as the catalyst; [d] the crude material contains additional unknown byproducts in the order of ≈10 %; [e] the exact ratio could not be determined because of the formation of unknown side‐products

**Scheme 2:**
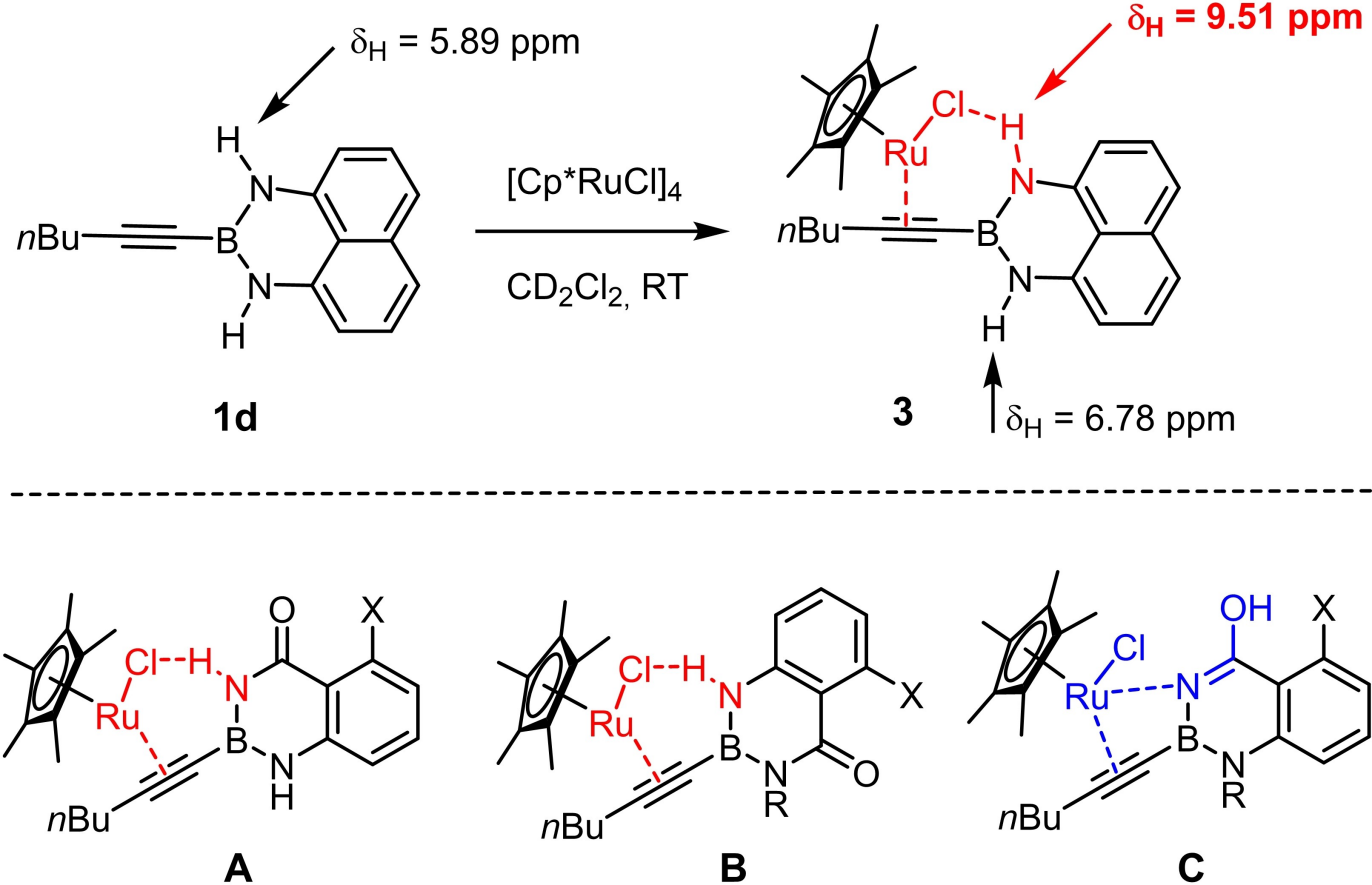
NMR‐spectroscopic evidence for interligand hydrogen bonding in the complex formed from the alkynyl−B(dan) derivative **1 d** and [Cp*RuCl]_4_; conceivable bonding motifs for alkynyl‐B(aam) derivatives.

To this end, we planned to resort to the directing effect exerted by protic functionality close to the triple bond. This idea was first tested with the corresponding ‐B(dan) derivative **1 d** (dan=naphthalene‐1,8‐diaminato),[^32^, ^33^, ^34^, ^35^, ^36^] which gave indeed a better outcome. An excellent *trans/cis* selectivity and an appreciable preference for the delivery of the Bu_3_Sn‐group to the C‐atom proximal to the protic site (α:β=85 : 15) were observed. The strong deshielding of the ^1^H NMR resonances of one of the two −NH protons (Δδ_H_≈3.6 ppm)^[37]^ of the substrate upon addition of [Cp*RuCl]_4_ (0.25 equiv.) in CD_2_Cl_2_ is strongly suggestive of the formation of the expected π‐complex featuring a lateral hydrogen bonding array, which in turn explains the improved selectivity (Scheme 2). Yet, this particular boron cap was soon recognized as non‐ideal since the substrate **1 d** itself is rather unstable and the resulting product **2 d** prone to partial protodestannation upon purification by flash chromatography; moreover, an even better regioselectivity was desirable and might be reached with a somewhat more acidic boron head group.

Indeed, the corresponding −B(aam) analogue **1 e** (aam=anthranilamidato) allowed all of these issues to be addressed. Although some virtues of the benzo‐1,3,2‐diazaborininone ring system have been clearly pointed out in the literature,[^35^, ^38^, ^39^, ^40^, ^41^, ^42^] it continues to be underutilized.^[43]^ In the present context, this particular boron heterocycle served our purpose very well: alkynyl‐B(aam) derivatives such as **1 e** are fairly easy to make on scale (see below) and bench‐stable for extended periods of time. The resulting *trans*‐addition product **2 e** is representative: it was obtained in 88 % yield with an impeccable *trans:cis* ratio (>99 : 1) and very high regioselectivity (α:β=96 : 4); the reaction scaled well, furnishing the same excellent results when performed on multi‐gram scale without need for further optimization. Unlike the corresponding −B(dan) analogue **2 d**, product **2 e** proved stable during flash chromatography. The stereochemical assignment follows from the characteristic ^3^ *J*_H,Sn_ coupling constant of 152.5 Hz and is consistent with nOe data (see the Supporting Information).

A number of control experiments was carried out to see if the favorable outcome is rooted in a synergistic interaction of the catalyst with the protic sites of the substrate. In contrast to our previous work with propargyl alcohols[^16^, ^18^, ^44^] as well as to the −B(dan) derivative **1 d** shown in Scheme 2, all attempts at characterizing the complex primarily formed from [Cp*RuCl] and **1 e** by spectroscopic and/or crystallographic means remained unsuccessful. Although addition of the precatalyst to a solution of **1 e** in CD_2_Cl_2_ caused an immediate color change to pink/purple, the color rapidly faded away even at low temperature. As previously described, π‐complex formation between a propargyl alcohol and the ruthenium catalyst is accompanied by the same characteristic appearance of a cherry red to pink color; the resulting adducts, however, had been found sufficiently stable to allow for full characterization by NMR and X‐ray diffraction.[^16^, ^18^] As the “loaded” complex formed from **1 e** and [Cp*RuCl] in the first place could not be characterized in this way, a stringent proof for the expected interligand hydrogen bonding between the protic boracycle and the polarized [Ru−Cl] bond of the catalyst is missing.

This lack of spectroscopic and/or crystallographic evidence is all the more regrettable since one can envisage different binding modes (Scheme 2). Rather than engaging the proton and the chloride ligand as shown in **A** and/or **B**, the tautomeric form of the heterocycle could come into play and ligate the metal center via the N‐atom by a conventional Lewis acid/base interaction.^[39a]^ A binding motif of type **C** also locks the substrate in place and could therefore also explain the observed regioselectivity.

In the absence of any direct information on the structure of this first reactive intermediate, it is not trivial to distinguish these different binding modes. In the starting material, the H‐atoms in question definitely reside on nitrogen as they were localized on the difference Fourier map of compound **22**; analysis of the bond lengths confirms the “amide‐like” character (see below). The ^1^ *J*_H,N_ coupling constants of 82/87 Hz for the two N‐atoms of the boracycle, as deduced from ^1^H,^15^N HSQC experiments at 25 °C as well as −50 °C, suggest that the same is true for solutions of **1 e** in CDCl_3_ (for details, see the Supporting Information).^[45]^ This fact, however, is no firm proof that they stay there once the strong π‐acid binds the triple bond. Likewise, either model explains why methylation of both N‐atoms results in a dramatic loss of selectivity and reactivity;^[46]^ actually, the conversion stalled at ca. 50 % and significant amounts of unknown byproducts were formed (Table 1, entry 7). N‐Methylation solely of the anthranilic amide N‐atom will prevent an interaction of type **A** from occurring but still allows for H‐bonding with the aniline N‐atom (**B**, R=Me). As the latter is less acidic, a poorer regioselectivity is expected but the *alpha,trans*‐product should still prevail (compare the outcome with the −B(dan) derivative **1 d**); this is indeed the case (entry 8). Exclusive N‐methylation of the aniline N‐atom, in contrast, renders the diazaborininone ring gradually more electron rich, which might (slightly) strengthen – but certainly not weaken – a Lewis acid/base interaction of type **C** (R=Me); yet, selectivity and reactivity drop again to a notable extent (entry 9). This observation can be taken as indirect evidence against a binding motif of type **C** being operative. This notion is supported by two additional control experiments, in which a fluoride substituent was placed at different positions on the annulated phenyl ring. For the electron withdrawal in the σ‐frame, the N−H acidity is gently up‐regulated whereas the donor strength of the N‐atom in the mesomeric form **C** either remain unchanged or will be reduced, if anything. As seen from entries 10 and 11, the system responds with an increase in selectivity; the almost perfect reaction outcome speaks for model **A** (for an additional example, in which a fluorinated aam unit entails higher selectivity, see Table 2). Whether this tentative conclusion pertains to the reaction of **1 e** with the cationic precatalyst [Cp*Ru(MeCN)_3_]PF_6_ too, which gave a surprisingly good isomer ratio but is less clean otherwise (entry 6),^[47]^ cannot be decided at this point.

**Table 2:** Formation and subsequent *trans*‐hydrostannation of alkynyl‐B(aam) derivatives.^[a,b]^

Entry	Alkynyl‐B(aam)		Yield (%)	Product^[c]^		Yield (%)	α:β^[d]^
1	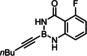	**1 i**	68	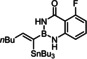	**2 i**	87	98 : 2
2	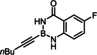	**1 j**	77	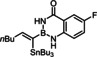	**2 j**	99	98 : 2
3	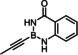	**4**	23	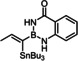	**5**	quant.	99 : 1
4	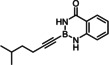	**6**	88	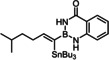	**7**	92	97 : 3
5	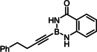	**8**	37	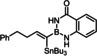	**9**	89	91 : 9
6	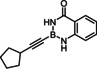	**10 a**	79	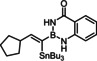	**11 a**	84	89 : 11 (97 : 3)^[e]^
7	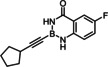	**10 b**	87	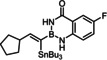	**11 b**	85	94 : 6 (99 : 1)^[e]^
8	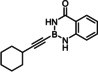	**12**	84	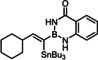	**13**	75	87 : 13 (95 : 5)^[e]^
9	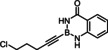	**14**	85	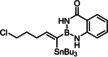	**15**	93	92 : 8
10	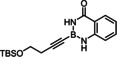	**16**	93	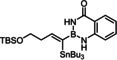	**17**	80	99 : 1
11	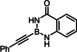	**18**	39	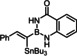	**19**	98	99 : 1
12	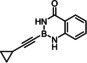	**20**	45	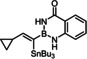	**21**	84	99 : 1
13	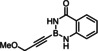	**22**	33	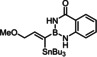	**23**	81	98 : 2
14	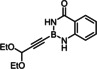	**24**	63	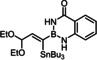	**25**	82	99 : 1
15	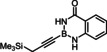	**26 a**	72	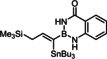	**27 a**	81	79 : 8 (+13)^[f]^
16	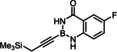	**26 b**	70	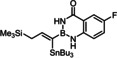	**27 b**	74	82 : 7 (+11)^[f]^
17	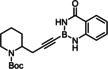	**28**	78	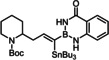	**29**	76	85 : 15^[g]^
18	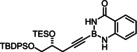	**30**	91	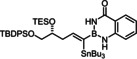	**31**	91	98 : 2
19	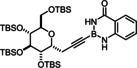	**32**	89	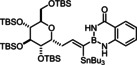	**33**	90	99 : 1

**Scheme 3:**
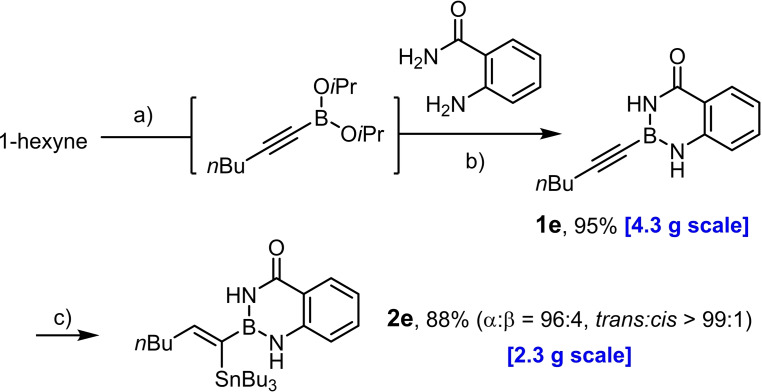
a) (i) *n*BuLi, THF, −78 °C; (ii) B(O*i*Pr)_3_, −78 °C; (iii) HCl in Et_2_O (2 M), −78 °C to RT; b) anthranilamide, toluene, reflux; c) Bu_3_SnH, [Cp*RuCl]_4_ (2.5 mol%), CH_2_Cl_2_.

[a] All reactions were carried out under the conditions shown in Scheme 3; [b] the *trans:cis* ratio, as determined by ^1^H NMR of the crude products, was ≥99 : 1, unless stated otherwise; [c] only the major *α,trans*‐isomer is shown; [d] α : β ratio as determined by ^1^H NMR of the crude products; [e] isomer ratio after flash chromatography; [f] the crude product contained the β*,cis*‐adduct (ratio in brackets); [g] after chromatographic removal of 15 % of the β,*cis*‐adduct

### Improved synthesis of alkynyl‐B(aam) derivatives and investigation into the scope of the reaction

**Figure 1:**
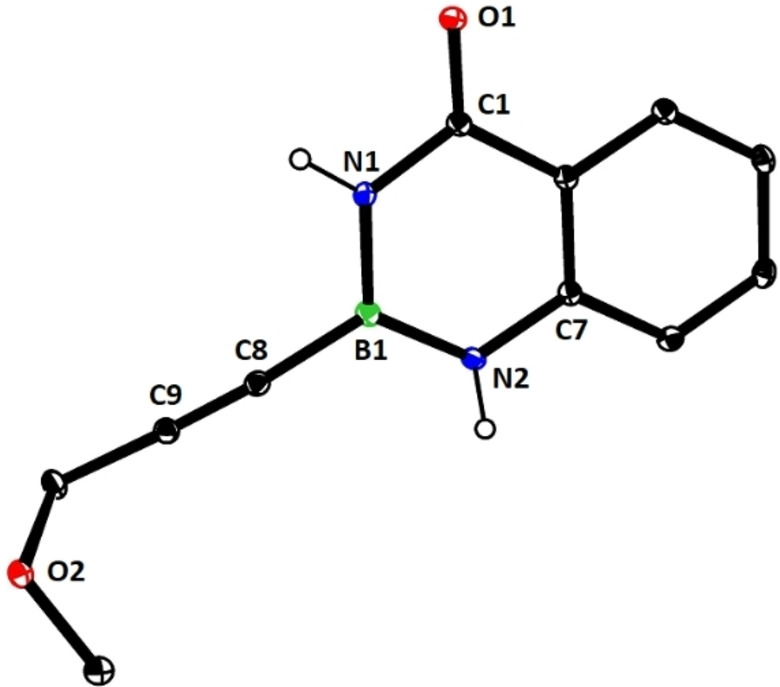
Structure of compound **22** in the solid state;^[52]^ the N−H atoms were localized on the difference Fourier map, all other H‐atoms are omitted for clarity; selected bond lengths (Å): C1‐O1 1.239(1), C1‐N1 1.370(1), B1‐N1 1.432(2), B1‐N2 1.409(2), B1‐C8 1.540(2), C8‐C9 1.204(2).

Model compound **1 e** had initially been prepared by adaptation of a literature procedure, in which stable trifluoroborate salts are chosen as precursors that are subjected to ligand exchange promoted by TMSCl (or silica) as the fluoride‐scavenging agent.^[48]^ However, the yield never exceeded 30 %, despite attempted optimization. Therefore a more direct route was pursued, in which the terminal alkyne was deprotonated with *n*BuLi in THF at −78 °C, the lithium acetylide quenched with B(O*i*Pr)_3_,^[49]^ and the crude boronate ester formed upon hydrolytic work‐up directly subjected to ligand exchange with cheap anthranilamide in refluxing toluene (Scheme 3 and Table 2).^[50]^ Evaporation of the solvent followed by washing of the product with pentane furnished the corresponding alkynyl‐B(aam) derivative in analytically pure form and good yield in most cases;^[51]^ only a few examples required purification by flash chromatography. This convenient procedure scales well, as described in the Supporting Information. The structure of compound **22** in the solid state (Figure 1) clearly shows the presence of the −NH rather than the −OH tautomer.

The examples compiled in Table 2 illustrate the wide scope and functional group tolerance of the subsequent *trans*‐hydrostannation. In most cases, excellent isomeric purity was reached, bearing witness of the strong directing effect of the benzo‐1,3,2‐diazaborininone ring system. In line with previous experiences with *trans*‐hydrometalations, branching at the propargylic position tends to decrease the regioselectivity,[^14^, ^16^] as manifested in the results obtained with the cyclopentyl and the cyclohexyl derivatives **10 a** and **12**, respectively; gratifyingly though, the samples were enriched by flash chromatography in the desired *alpha,trans*‐isomers **11 a** and **13**, respectively,. Alternatively, one can resort to the use of the fluorinated anthranilamide derivative **10 b**, which provides another solution to this problem: the comparison of entries 6 and 7 confirms the notion that the fluorinated boron‐heterocycle exerts a stronger directing effect.

Lower selectivities were observed with the N‐Boc protected piperidine derivative **28** and the propargyl silane **26 a**. Noticeable amounts (ca. 10–15 %) of the corresponding *beta,cis*‐addition products were present in the crude materials, an isomer which is virtually absent in all other cases investigated. Whereas this impurity could be removed by flash chromatography from the fairly polar adduct **29**, it was not possible to enrich **27 a** analogously. Use of a fluorinated directing group led again to some improvement but the isomer distribution remained more modest. The electronic bias imposed on the triple bond by the hyperconjugated Me_3_Si‐group is hence an important and partly antagonistic selectivity‐determining factor (compare entries 15/16). The lower isomeric purity is regrettable as building blocks of type **27** feature three different metalloid centers that might be chemoselectively addressed and could hence allow for interesting follow‐up chemistry.

### Limitations

**Figure 2:**
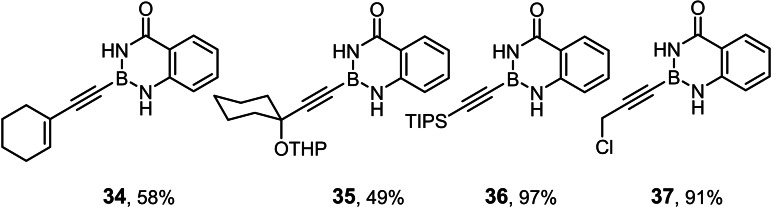
Additional alkynyl‐B(aam) derivatives which failed to undergo *trans*‐hydrostannation; THP=2‐tetrahydropyranyl; TIPS=tri(isopropyl)silyl.

The compounds shown in Figure 2 failed to undergo productive *trans*‐hydrostannation under the standard reaction conditions. The lack of reactivity reiterates earlier findings that 1,3‐enynes and acetylenes flanked by very bulky tertiary substituents are problematic. Specifically, η^4^‐binding of the enyne unit of **34** to [Cp*RuCl] seems to afford a kinetically stable and coordinatively saturated 18‐electron adduct, which is incompetent for *trans*‐hydrometalation.[^16^, ^18^, ^53^] In case of alkynes such as **35** carrying very bulky substituents, binding of the catalyst to the triple bond is impeded on steric grounds.^[18]^ A similar argument is thought to pertain to compound **36** too, in which the altered electronics of the triple bond may be an additional handicap.^[30]^ The propargyl chloride in **37**, in contrast, led to decomposition of this compound.

The analogous *trans*‐hydrosilylation of **1 e** with BnMe_2_SiH as well as *trans*‐hydroboration with H−B(pin) were sluggish (50–70 % yield in 18 h (NMR)) and showed quite modest selectivities (α:β≈80 : 20).^[31]^ Attempted *trans*‐hydrogenation of this substrate failed to afford any product. While similar trends have been observed in the propargyl alcohol series, with *trans*‐hydrostannation being the fastest and most selective of the ruthenium‐catalyzed addition reactions, the differences are less pronounced there.[^16^, ^18^] The exact reasons why alkynyl‐B(aam) derivatives undergo *trans*‐hydrostannation so easily but are reluctant to succumb to any of the other ruthenium catalyzed reactions remain to be fully elucidated. It is not unreasonable, however, to assume that the short lifetime of the complex initially formed between the substrate and the ruthenium catalysts (see above) might be a critical factor; only the activated Sn−H bond seems to meet this kinetic selection criterion.

### Boron‐selective cross coupling

**Scheme 4:**
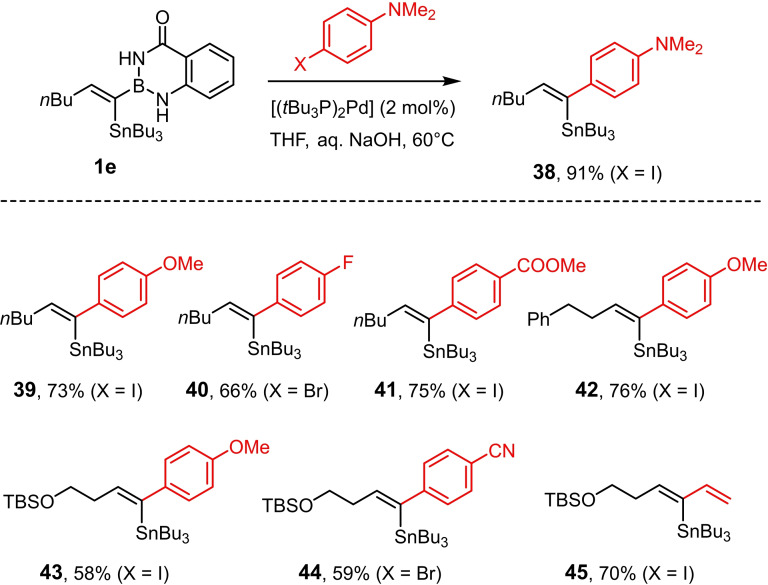
Chemoselective cross coupling of −B(aam) groups in the presence of tributylstannane entities; TBS=*tert*‐butyldimethylsilyl.

Building blocks containing two different metalloid substituents hold the promise of rich downstream chemistry.^[27]^ In the present context, however, one has to keep in mind that the use of −B(aam) derivatives for Suzuki‐Miyaura cross coupling reactions is uncommon. Yet, a recent investigation provides encouraging precedent in that it showed that aryl‐B(aam) derivatives allow for direct cross coupling without need for prior unmasking of the corresponding boronic acid.[^35^, ^41^] The results compiled in Scheme 4 do not only confirm this result: it is actually stunning to find that the Suzuki‐Miyaura reaction outcompetes a Stille cross coupling under the chosen conditions;^[54]^ in the basic aqueous medium, protodestannylation did not interfere. This somewhat unusual yet gratifying “inverse” order of reactivity brings functionalized alkenylstannanes into reach in isomerically pure form which would be difficult to prepare otherwise.

**Scheme 5:**
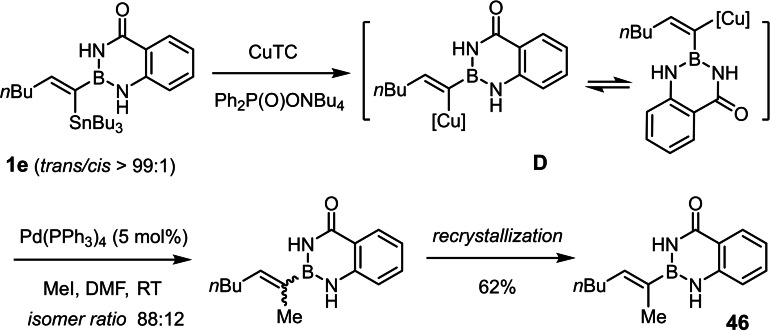
Example of a Stille reaction accompanied by partial double bond isomerization; CuTC=copper thiophene‐2‐carboxylate.

Quite perplexing was the observation that selective Stille coupling would not proceed well under anhydrous conditions either.[^55^, ^56^] Even the reaction with highly activated partners such as 1‐iodo‐4‐nitrobenzene or methyl (*Z*)‐3‐iodo‐acrylate proved problematic under palladium/copper co‐catalyzed conditions that had excelled in the past in many challenging cases.[^57^, ^58^] When applied to **2 e**, the reactions were unusually slow and led to partial isomerization of the double bond as judged by NMR inspection of the crude material. The same is true for a Stille‐type methylation,^[59]^ which furnished the desired product **46** in 62 % yield after recrystallization, but the crude material was again contaminated with isomerized and protodestannlyated by‐products (Scheme 5). Since double bond isomerization is extremely rare in palladium catalyzed cross coupling in general and Stille coupling in particular,^[55]^ we cannot help but assume that the peculiar vinylidene‐type character of the α‐borylated organometallic intermediate **D** (presumably at the copper‐ rather than the palladium stage)[^60^, ^61^] primarily formed by transmetalation of the alkenyltin moiety accounts for this complication.

### Tin/halogen exchange and follow‐up chemistry

**Scheme 6:**
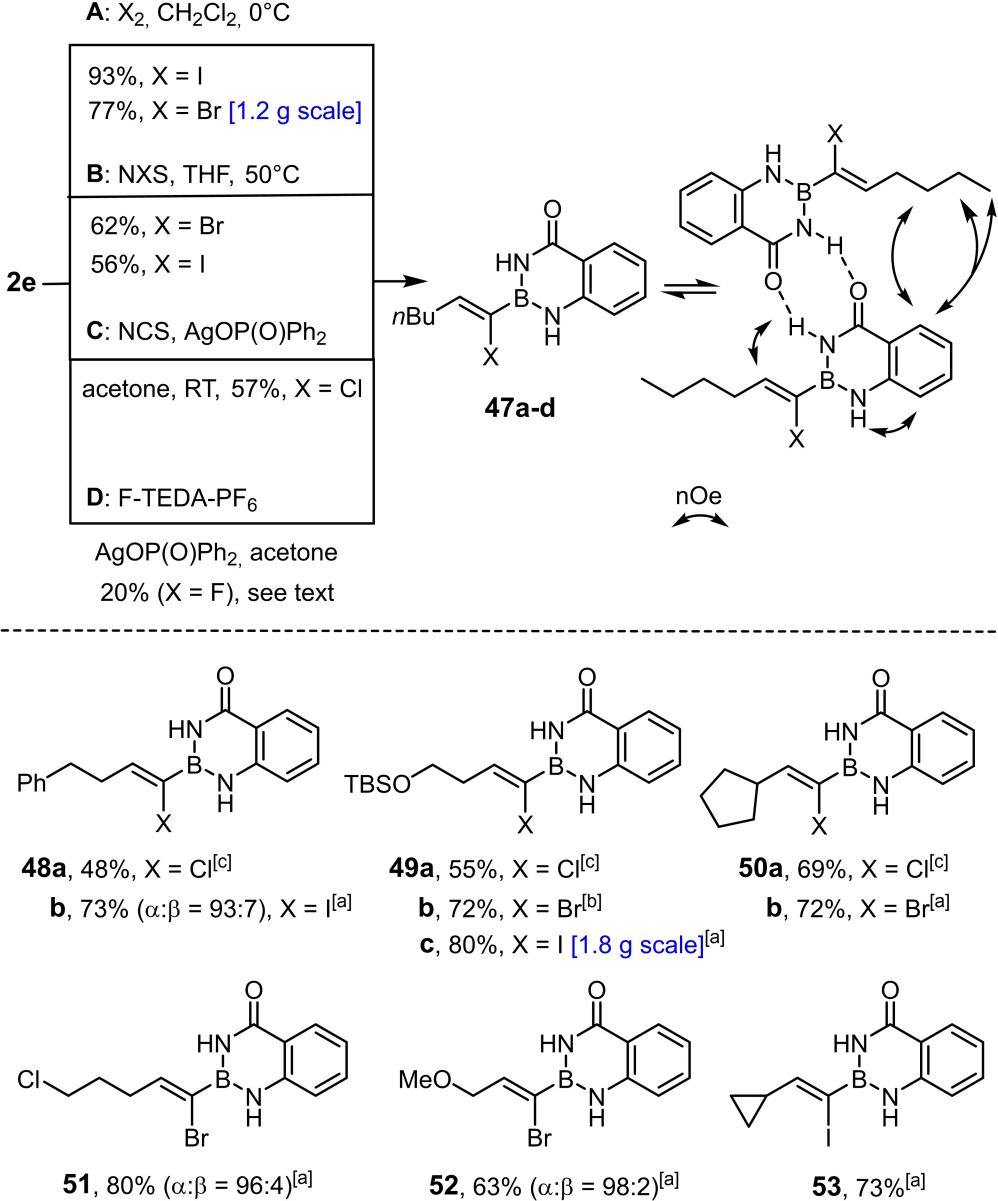
Selective tin/halogen exchange: [a] conditions **A**; [b] conditions **B**; [c] conditions **C**; unless stated otherwise, the α:β‐ratio was ≥99 : 1.

The surprising reluctance of α‐stannylated alkenyl‐B(aam) derivatives to undergo stereoretentive Stille reactions can be circumvented by first subjecting them to tin/halogen exchange (Scheme 6). Formation of the corresponding α‐borylated alkenyl iodides and bromides is particularly facile with X_2_ (X=Br, I) in CH_2_Cl_2_ at 0 °C, but also works with NXS at 50 °C; no scrambling was observed under these conditions.^[62]^ For the sake of convenience, hydrostannation and tin/halogen exchange can be carried out in “one pot” (see the Supporting Information). The analogous reaction with NCS, in contrast, gave substantial amounts of proto‐destannylated product despite the use of freshly recrystallized NCS and rigorously dried solvents. This problem was fixed by supplementing the mixture with AgOP(=O)Ph_2_ as an essentially neutral and π‐affine promoter and tin scavenger; in its presence, the corresponding alkenyl chlorides were obtained in isomerically pure form in respectable yields. Although the analogous tin/fluoride exchange with AgOP(=O)Ph_2_ in combination with F‐TEDA‐PF_6_ is possible,^[63]^ competing protodestannation could not be completely suppressed; although the desired alkenyl fluoride **47 b** is the major compound (75 : 25), the separation proved tedious and resulted in loss of material, such that no more than 20 % of pure **47 d** could be obtained (for the structure of this compound in the solid state confirming the assigned configuration, see the Supporting Information).^[52]^ The NMR spectra of all halogenated products of this series are concentration‐dependent, but the effect is most pronounced for the corresponding chloride derivatives. Characteristic nOe's suggest that the phenomenon is due to reversible formation of H‐bonded dimers.

**Scheme 7:**
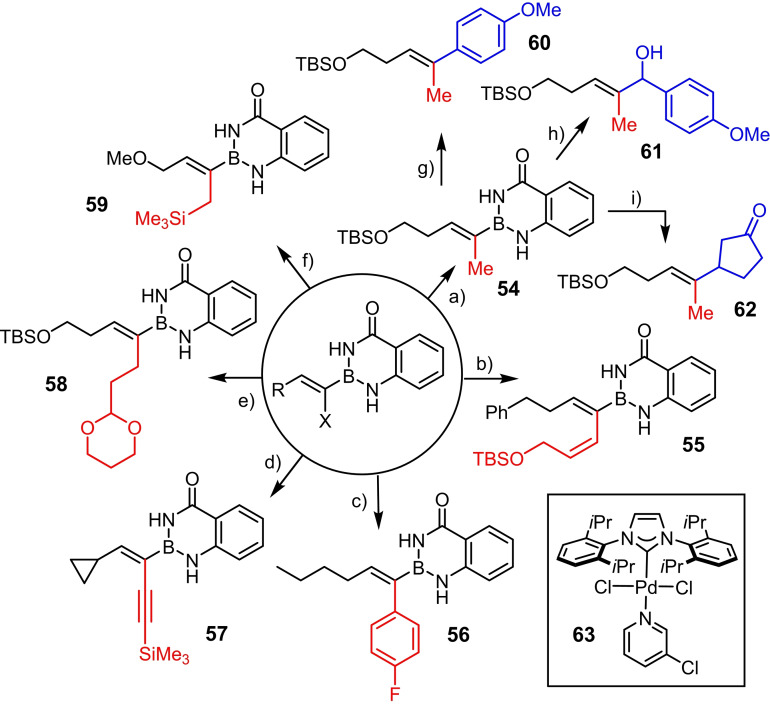
Negishi cross coupling reactions in presence of protic −B(aam) groups and downstream functionalization of the resulting alkenyl‐B(aam) derivatives: a) Me_2_Zn, **63** (2 mol%), THF, RT, 84 % (X=I); b) (i) (*Z*)‐*tert*‐butyl(3‐iodoallyl)oxy)dimethylsilane, *t*BuLi, Et_2_O, −78 °C, then ZnBr_2_; (ii) **63** (2 mol%), THF, RT, 48 % (X=I); c) (i) FC_6_H_4_MgBr, ZnBr_2_, THF, 0 °C; (ii) [(*t*Bu_3_P)_2_Pd] (2 mol%), THF, RT, 87 % (X=Br); d) (i) trimethylsilylacetylene, *n*BuLi, THF, −78 °C, then ZnBr_2_; (ii) [(*t*Bu_3_P)_2_Pd] (2 mol%), THF, RT, 80 % (X=I); e) (i) (2‐(1,3‐dioxan‐2‐yl)ethyl)magnesium bromide, ZnBr_2_, THF, 0 °C; (ii) **63** (2 mol%), THF, RT, 77 % (X=I); f) (i) Me_3_SiCH_2_MgCl, ZnBr_2_, THF, 0 °C; (ii) **63** (2 mol%), THF, RT, 71 % (X=Br); g) Pd(PPh_3_)_4_ (5 mol%), *t*BuOK, 1,4‐dioxane, 100 °C, 68 %; h) *p*‐MeOC_6_H_4_CHO, [(cod)RhCl]_2_ (3 mol%), K_3_PO_4_, 1,4‐dioxane/H_2_O, 140 °C (microwave), 53 %; i) 2‐cyclopentenone, [(cod)RhCl]_2_ (3 mol%), K_3_PO_4_, 1,4‐dioxane/H_2_O, 140 °C (microwave), 61 %; cod=1,5‐cyclooctadiene.

The α‐borylated alkenyl halides undergo Negishi cross coupling reactions with ease;[^64^, ^65^] representative examples are compiled in Scheme 7; in no case has any isomerization of the double bond been observed. The fact that a slight excess of the organozinc reagents (1.2 equiv.) sufficed to reach full conversion implies that the two protic sites of the benzo‐1,3,2‐diazaborininone ring do not quench the C−Zn bond. In view of the results reported above, it does not come as a surprise that the −B(aam) moiety of the resulting products also participated well in subsequent Miyaura‐Suzuki reactions and in rhodium‐catalyzed 1,2‐ as well as 1,4‐additions to aldehydes and enones, respectively, as exemplified by the transformation of compound **54** into the functionalized trisubstituted alkene derivatives **60**–**62**.[^41b^, ^66^, ^67^]

## Conclusions

Combined experimental and computational studies had previously shown that the unprotected −OH group of a propargyl alcohol is able to steer the ruthenium catalyzed *trans*‐hydrostannation of the triple bond by virtue of interligand hydrogen bonding with a polarized [Ru−Cl] subunit of the catalyst. The current paper extents this principle to a very different class of protic substrates: specifically, alkynyl‐B(aam) (aam=anthranilamidato) derivatives are shown to undergo highly regio‐ and stereoselective addition of Bu_3_SnH, provided the reaction is catalyzed with [Cp*RuCl]_4_ in CH_2_Cl_2_. Indirect evidence suggests that the observed *alpha,trans*‐delivery of the tin moiety to the C‐atom flanking the boracycle is indeed largely rooted in a hydrogen bonding array in the loaded catalyst rather than in conventional Lewis acid/base interactions. The required substrates are bench‐stable compounds that are readily prepared on scale; the resulting products provide numerous opportunities for downstream functionalization. Most notably, the −B(aam) group allows for Suzuki‐Miyaura coupling without need for hydrolysis of the boron heterocycle in a separate step; actually, this reaction outcompetes a conceivable Stille coupling of the flanking organotin moiety; the latter, however, is amenable to selective metal/halogen exchange. Overall, the regio‐ and stereochemically unorthodox *trans*‐hydrostannation of alkynyl‐B(aam) derivatives described herein opens a practical gateway to *gem*‐heterobimetallic building blocks that are difficult to make otherwise. One application pertains to the formation of isomerically pure trisubstituted alkenes by sequential functionalization of the two different organometallic residues at their terminus.

## Conflict of interest

The authors declare no conflict of interest.

## Supporting information

As a service to our authors and readers, this journal provides supporting information supplied by the authors. Such materials are peer reviewed and may be re‐organized for online delivery, but are not copy‐edited or typeset. Technical support issues arising from supporting information (other than missing files) should be addressed to the authors.

Supporting Information
