# Supplementary material for: Regioselective trans‐Hydrostannation of Boron‐Capped Alkynes
Source: Chemistry. 2021 Aug 4;27(68):17002–11. doi: 10.1002/chem.202101901 (PMC9291331; doi:10.1002/chem.202101901)
Supplement: Supplementary file 1 — Supporting Information [file CHEM-27-17002-s001.pdf]

Chemistry–A European Journal

Supporting Information

Regioselective *trans*-Hydrostannation of Boron-Capped Alkynes

Romain Melot, Tomas J. Saiegh, and Alois Fürstner*

Supporting Crystallographic Information

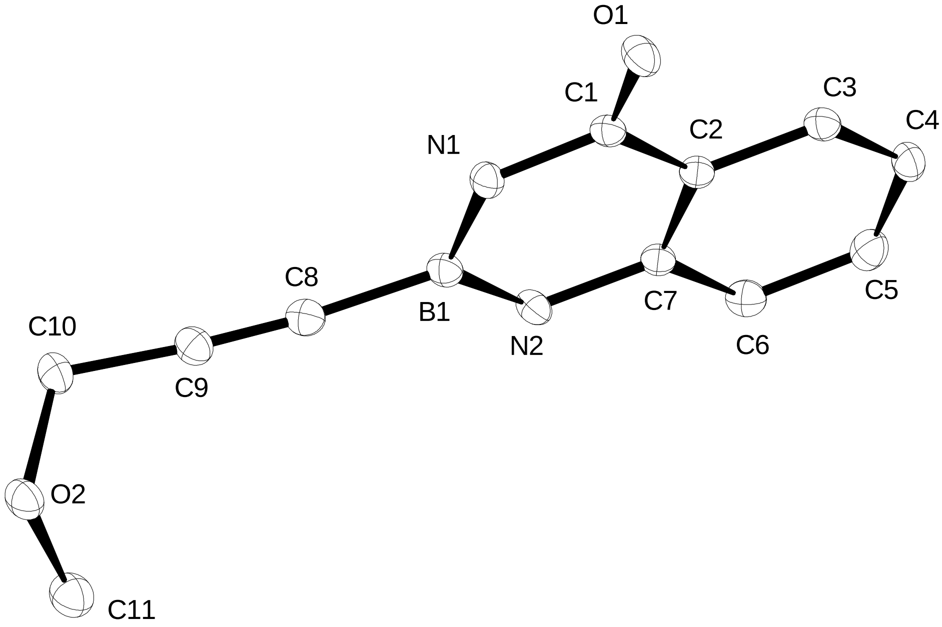

Figure S1. Structure of compound **22** in the solid state; arbitrary numbering.

X-ray Crystal Structure Analysis of Compound 22. $C_{11} H_{11} B N_2 O_2$, *Mr* = 214.03 g · mol^{-1}, colorless prism, crystal size 0.163 x 0.138 x 0.090 mm^3, monoclinic, space group $P2_1$/c, *a* = 7.4918(2) Å, *b* = 15.6775(5) Å, *c* = 9.0919(3) Å, β = 92.3000(10)°, *V* = 1067.01(6) Å^3, *T* = 100(2) K, *Z* = 4, D_{calc} = 1.332 g · cm^3, λ = 1.54178 Å, μ(*Cu-K*α) = 0.747 mm^{-1}, Gaussian absorption correction (T_{min} = 0.92, T_{max} = 0.95), Bruker-AXS Kappa Mach3 APEX-II diffractometer with FR591 rotating Cu-Anode, 5.628 < Θ < 71.430°, 32168 measured reflections, 2053 independent reflections, 1908 reflections with $I > 2\sigma(I)$, R_{int} = 0.0306.

The structure was solved by direct methods and refined by full-matrix least-squares against F^2 to R_1 = 0.033 [$I > 2\sigma(I)$], wR_2 = 0.092, 154 parameters. The hydrogens at N1 and N2 were found and refined the other hydrogens were refined using a riding model, *S* = 1.065, residual electron density 0.2 (0.92 Å from N2)/ –0.2 (0.81 Å from N2) e · Å^{-3}. **CCDC- 2083795**.

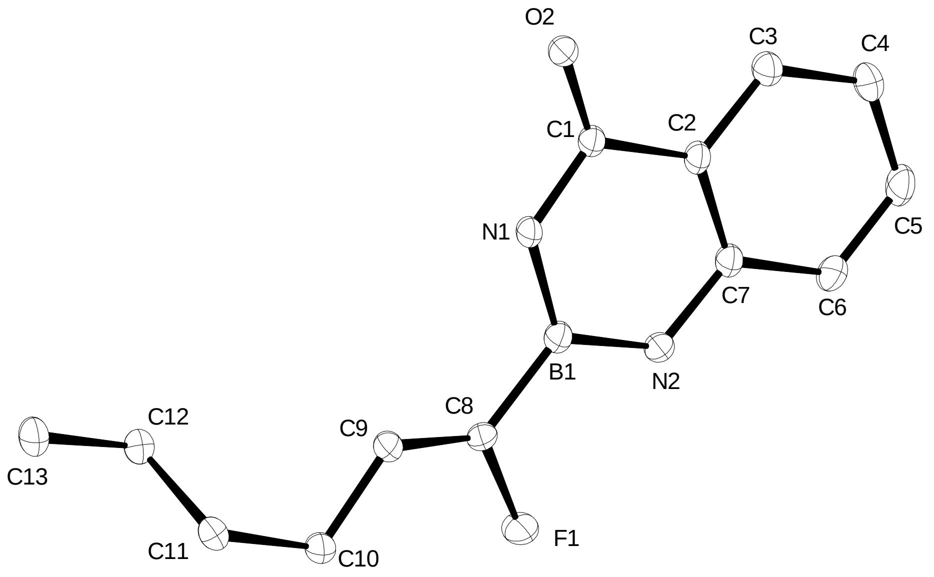

Figure S2. Structure of compound **47d** in the solid state; arbitrary numbering scheme.

X-ray Crystal Structure Analysis of Compound 47d. $C_{13}H_{16}BFN_2O$, *Mr* = 246.09 g · mol^{-1}, colorless needles, crystal size 0.24 x 0.06 x 0.05 mm^3, triclinic, space group $P\overline{1}$, *a* = 5.0306(9) Å, *b* = 9.5904(19) Å, *c* = 13.414(3) Å, α = 99.42(2)°, β = 98.56(2)°, γ = 101.383(13)°, *V* = 614.8(2) Å^3, *T* = 100(2) K, *Z* = 2, D_{calc} = 1.329 g · cm^3, λ = 0.71073 Å, μ(*Mo-K*α) = 0.094 mm^{-1}, Gaussian absorption correction (T_{min} = 0.99, T_{max} = 1.00), Bruker-AXS Kappa Mach3 APEX-II diffractometer with FR591 rotating Mo-anode, 2.954 < Θ < 33.101°, 11152 measured reflections, 4469 independent reflections, 3207 reflections with $I > 2\sigma(I)$, R_{int} = 0.0452.

The structure was solved by direct methods and refined by full-matrix least-squares against F^2 to R_1 = 0.053 [$I > 2\sigma(I)$], wR_2 = 0.146, 172 parameters. The hydrogens at N1 and N2 were found and refined the other hydrogens were refined using a riding model, *S* = 1.049, residual electron density 0.4 (0.71 Å from C12)/ –0.3 (0.65 Å from C1) e · Å^{-3}. **CCDC- 2083794**.

Supporting Spectroscopic Information

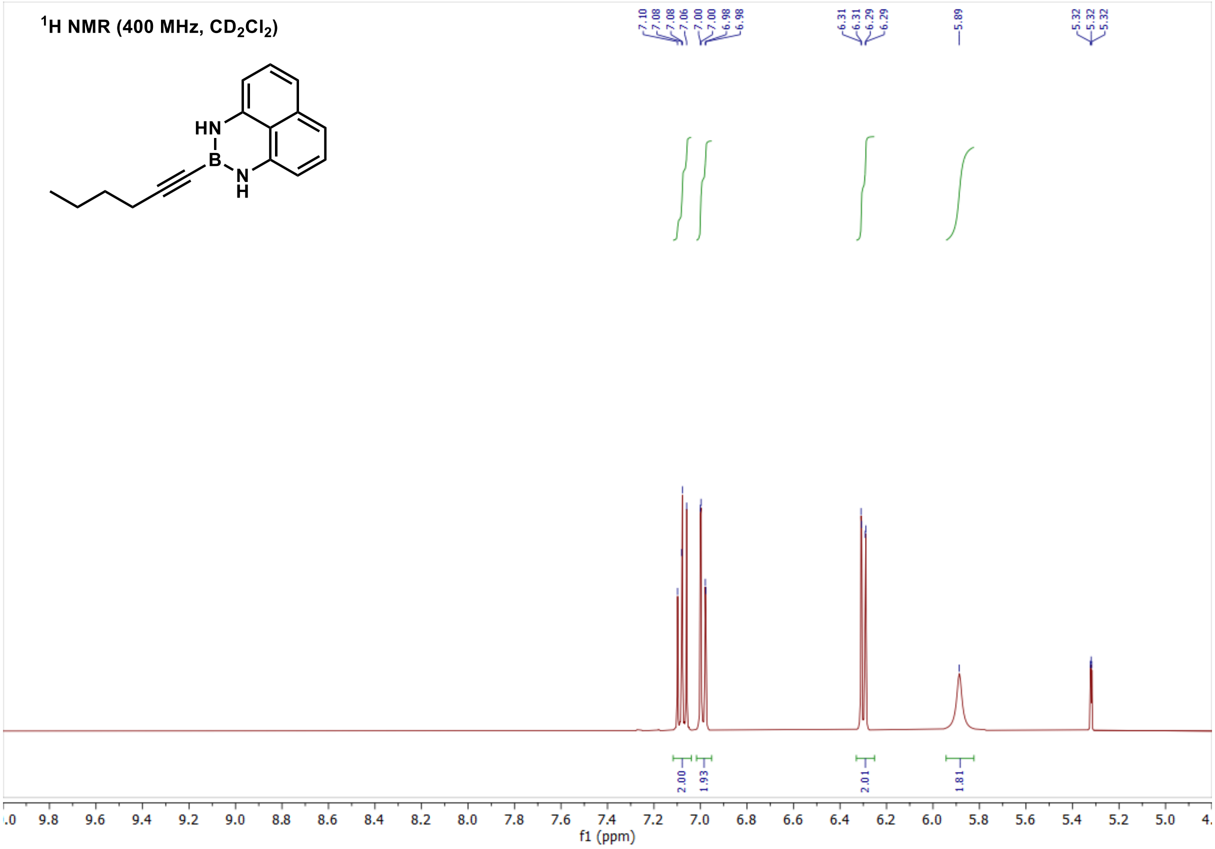

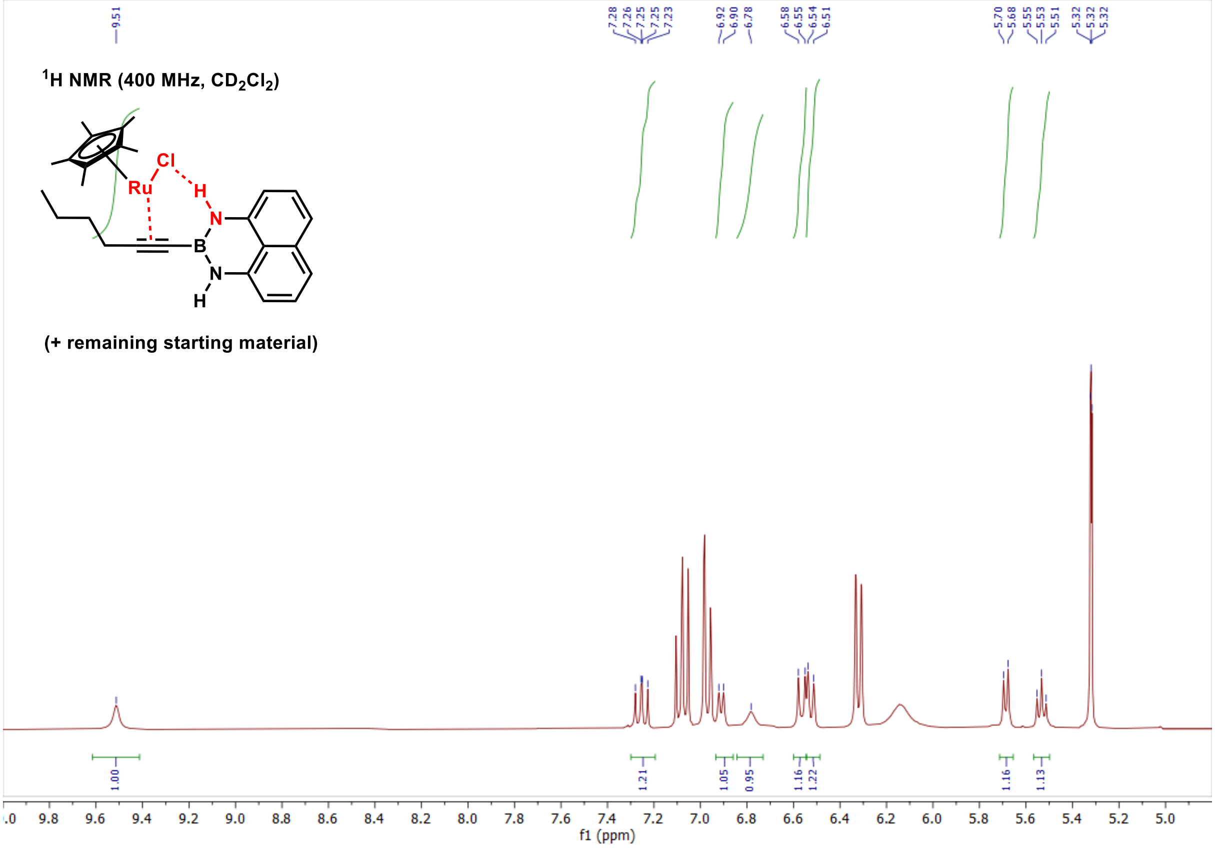

Figure S3. ^{1}H NMR spectra of the –B(dan) substrate **1d** and the derived ruthenium complex **3**

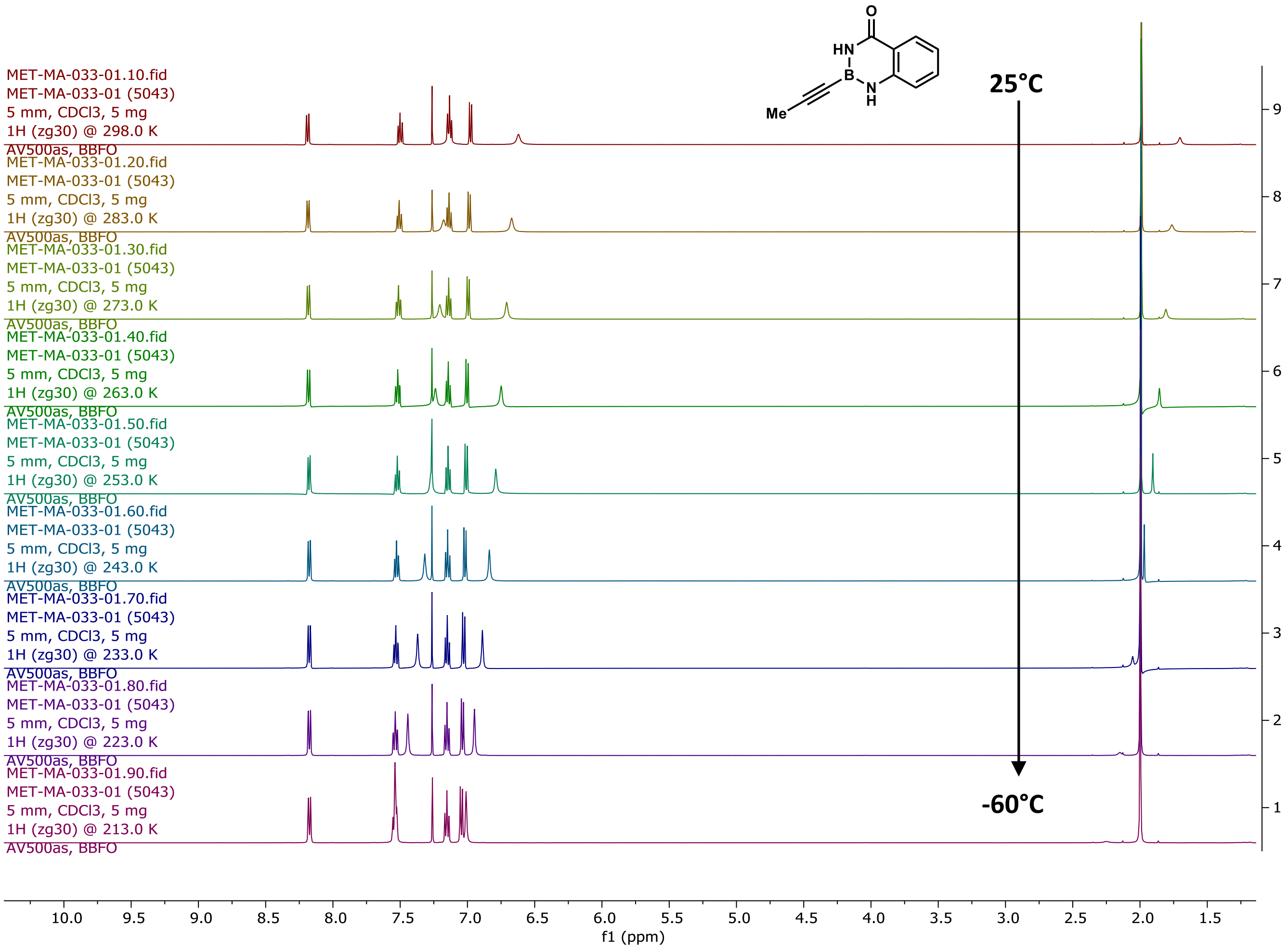

Figure S4. Temperature-dependence of the ^{1}H NMR spectrum ($CDCl_3$) of compound **4**: only a slight deshielding of the –NH signals is observed but no second signal set, as expected if two tautomeric forms of the heterocylic ring were present in solution

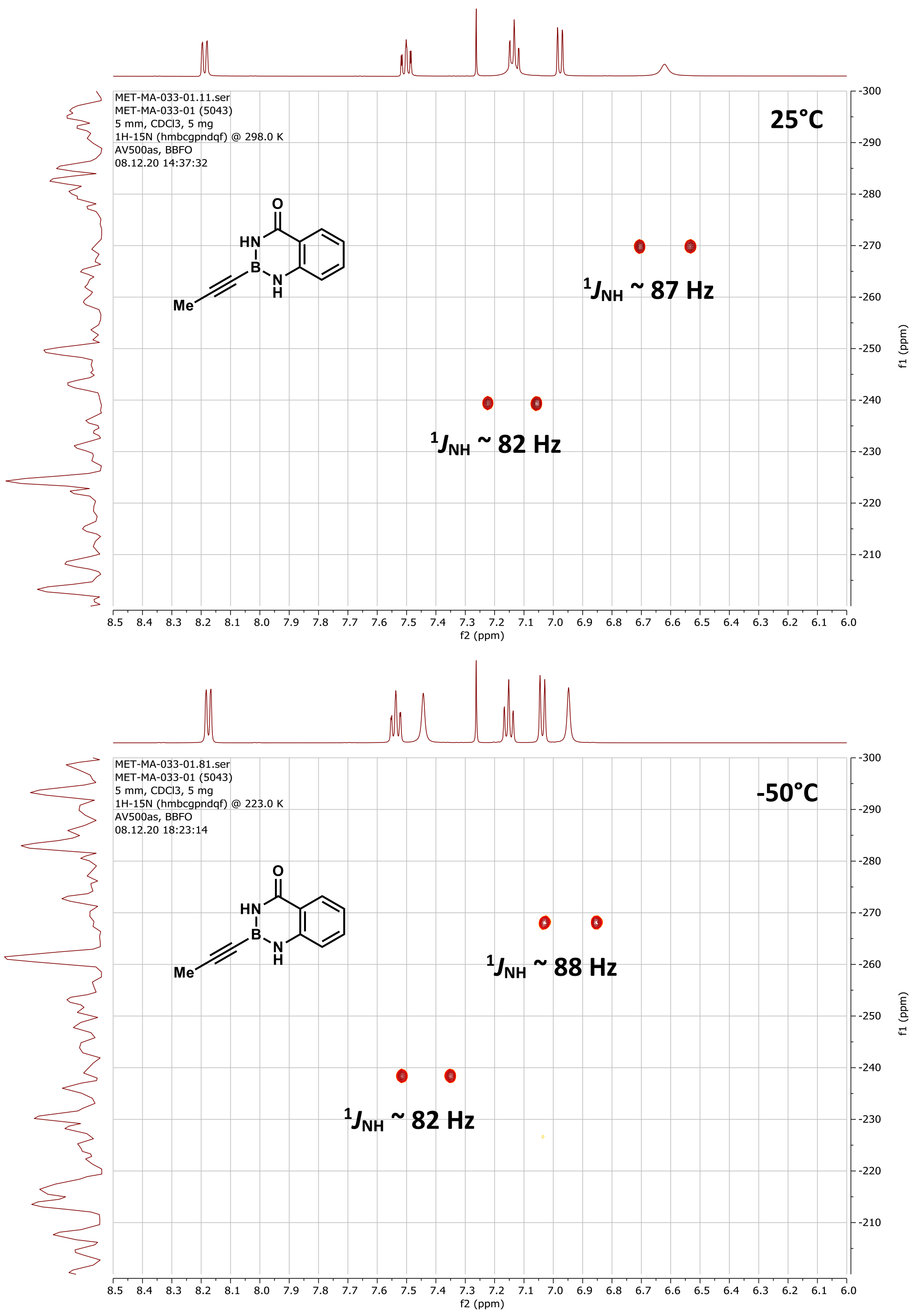

Figure S5. ^{1}H-^{15}N HSQC experiments at 25°C and –50°C

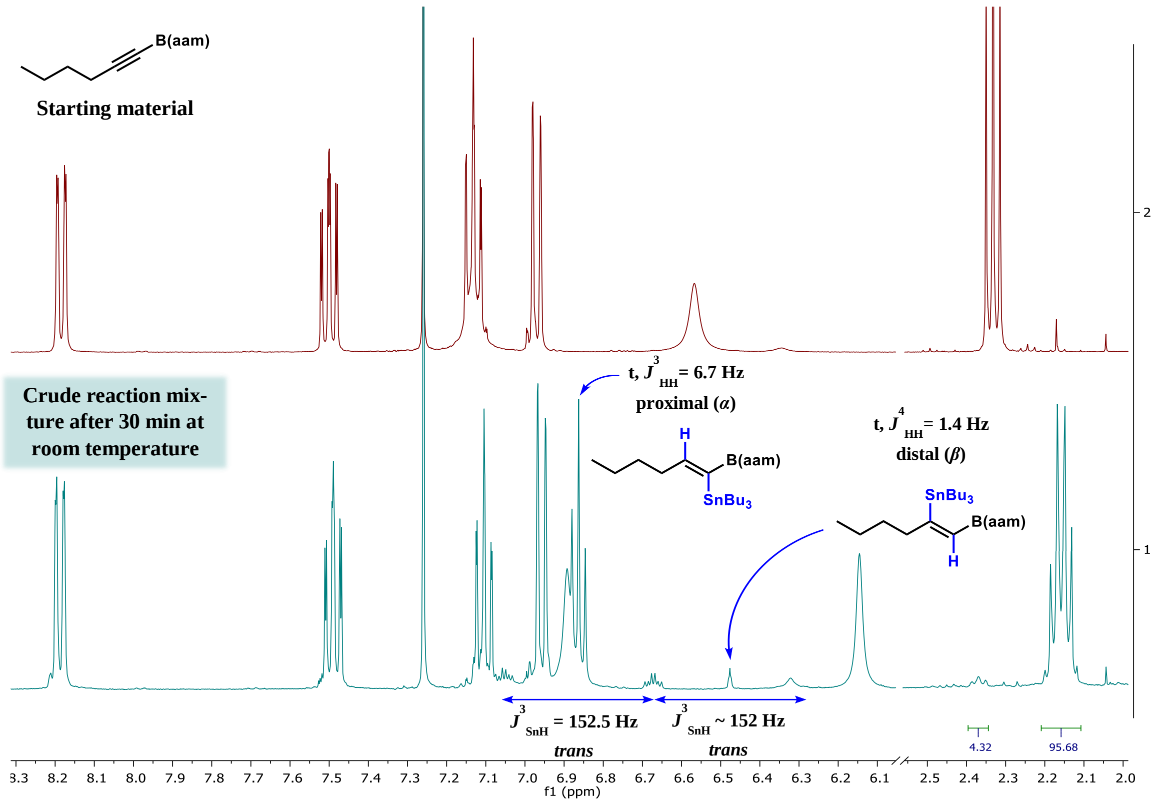

Figure S6. ¹H NMR spectra ($CDCl_3$) of model substrate **1e** and the resulting crude reaction mixture comprising the *alpha*,*trans*-addition product **2e** as the major component

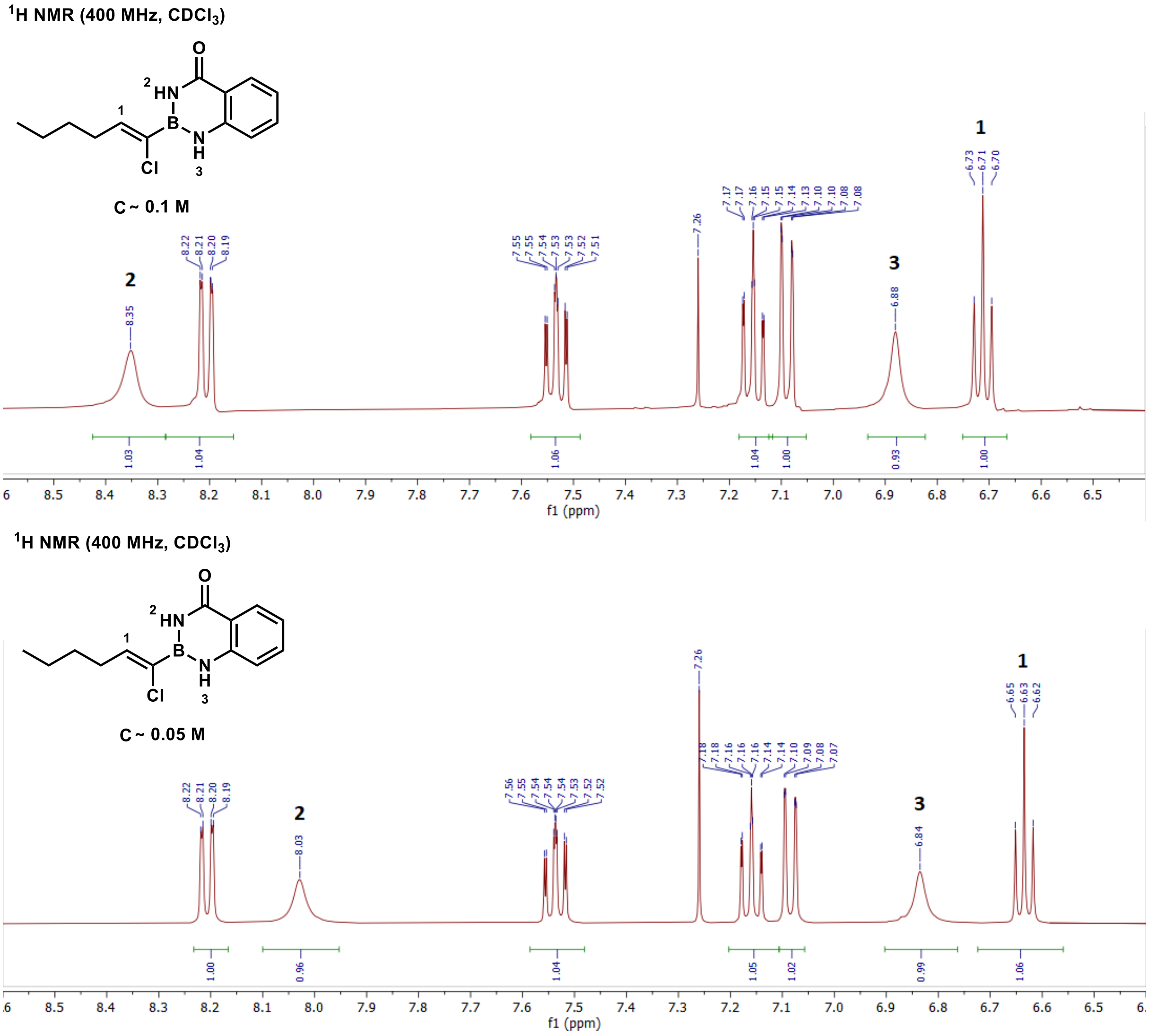

Figure S7. ^{1}H NMR spectrum of compound **47c** at two different concentrations

Table S1. Compilation of characteristic NMR data of boron capped alkynes *n*Bu**C**$_{\beta}$≡**C**$_{\alpha}$-BX$_2$

**-BX$_2$**	**δ NH (ppm)[a]**		**δC$_{\alpha}$ (ppm)[b]**	**δC$_{\beta}$ (ppm)[b]**	**Δδ C (ppm)**	**δB (ppm)**
-B(pin)	-		76.6	105.2	28.6	23.5
-B(dan)	5.77		79.8	105.2	25.4	21.6
-B(aam)	6.56	7.12	78.5	107.9	29.4	21.7
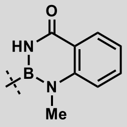	-	7.19	79.4	110.3	30.9	22.8
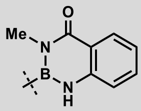	6.62	-	79.6	109.9	30.3	23.1
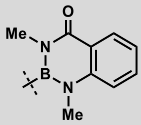	-	-	80.1	112.8	32.7	24.1
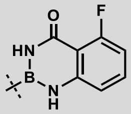	6.62	7.07	78.2	108.5	30.3	21.7
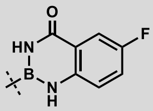	6.60	7.18	78.5	108.3	29.8	21.4

[a] *determined by ^{1}H NMR;* [b] *determined by ^{1}H-^{13}C HMBC NMR experiments.*

General Information

Techniques. Unless stated otherwise, all reactions were carried out under Argon in flame-dried glassware using Schlenk techniques, employing double-line argon-vacuum manifolds. Analytical thin layer chromatography (TLC) was performed using pre-coated polyester sheets (40 x 80 mm) POLYGRAM® SIL G/UV_{254} (0.20 mm silica gel 60 with fluorescent indicator). Visualization of the developed chromatogram was performed by UV absorbance (254 nm) and/or TLC stains ($KMnO_4$, *p*-anisaldehyde, phosphomolybdic acid). Flash chromatography was performed using Merck Geduran silica gel 60 (40 – 63μm) with the indicated solvent systems.

Chemicals. The following solvents and organic bases were purified by distillation over the indicated drying agents and were transferred under Ar: THF (Mg/anthracene); pentane, hexane, toluene (Na/K); CH_2Cl_2 (CaH_2); MeOH (Mg, stored over 3 Å MS). DMF, DMSO, 1,4-dioxane, MeCN and pyridine were dried by an adsorption solvent purification system based on molecular sieves. All other commercially available compounds (ABCR, Acros, Alfa Aesar, Aldrich, Fluka, STREM, TCI) were used as received, unless otherwise noted. *n*Bu–BPin, *n*Bu–BF_3K and *n*Bu–BF_3NBu_4 were prepared according to literature procedures.[1–3] Commercial Bu_3SnH is stabilized with 0.05% of 3,5-di-*tert*-butyl-4-hydroxytoluene, which was not removed in any of the reactions described herein. The following catalysts were prepared according to the cited literature: [Cp*Ru(CH_3CN)$_3$]PF_6,[4] [Cp*RuCl_2]n,[5] and [Cp*RuCl]$_4$.[5]

Instrumentation. NMR spectra were recorded on Bruker AV 300, AV 400, AV 500 or AVIII 600 spectrometers in the solvents indicated. Chemical shifts *(δ)* are reported in ppm relative to TMS; coupling constants (*J*) are given in Hz. Multiplets are indicated by the following abbreviations: s = singlet, d = doublet, t = triplet, q = quartet, quint = quintuplet, hex = hexuplet, sept = septuplet, m = multiplet. The abbreviation br indicates a broad signal. ^{11}B, ^{13}C, ^{19}F and ^{119}Sn spectra were recorded in {^1H}-decoupled manner and the values of the chemical shifts are rounded to one decimal point. Signal assignments were established using HSQC, HMBC, COSY, NOESY and other 2D experiments. ^{11}B NMR spectra were referenced to external $BF_3{\cdot}OEt_2$, ^{19}F NMR spectra were referenced to external $CFCl_3$, and ^{119}Sn NMR spectra were referenced to external $SnMe_4$.

Mass spectra were measured using the following devices: MS (EI): S-3 Finnigan MAT 8200 (70 eV), ESI-MS: Bruker ESQ3000, accurate mass determinations: Bruker APEX III FT-MS (7 T magnet) or MAT 95 (Finnigan).

IR spectra were recorded on an Alpha Platinum ATR spectrometer (Bruker) at ambient temperature, wavenumbers ($\tilde{\nu}$) are given in cm^{-1}.
Optical rotations were measured with an A-Krüss Otronic Model P8000-t polarimeter at a wavelength of 589 nm. The values are given as specific optical rotation with exact temperature, concent n (c in g/100 mL) and solvent.

Additional Information. Carbon centers directly bound to boron were often not observed in the corresponding ^{13}C NMR spectra due to line broadening but could usually be determined via HMBC experiments. Moreover, in the case of alkyne-B(aam) starting materials, even the signal of the distal (in regard of boron atom) alkynyl C-atom could, sometimes, not be observed directly by ^{13}C NMR and was also determined via HMBC experiments.
For clarity, Sn–H couplings of the olefinic protons were omitted in the multiplet analysis but are given in brackets (averaged over $^{117/119}Sn$). The of *α*- and *β*-isomer refer to the product containing the tributyltin unit proximal or distal to the boron atom respectively. Unless stated otherwise, the isomer ratio of the purified material was identical to that observed in the ^{1}H NMR of the crude product.

Substrates

General Procedure for the Prepa n of Boron-Capped Alkynes. A flame-dried Schlenk flask was charged with the selected alkyne (10 mmol) and THF (20 mL) under argon atmosphere. The resulting solution was cooled to −78°C before a solution of *n*-butyllithium (1.6 M in hexane, 6.25 mL, 10 mmol) was added dropwise to the vigorously stirred mixture. After 1 h at −78°C, triisopropyl borate (2.3 mL, 10 mmol) was added dropwise, causing the appearance of a white suspension. After stirring for 2 h at −78°C, a solution of hydrogen chloride in diethyl ether (2 M, 5.25 mL, 10.5 mmol) was introduced, leading to the formation of a clear solution. The dry ice bath was removed and stirring was continued for 30 min while the solution reached room temperature. The mixture was transferred into a round bottom flask (rinsing with *tert*-butyl methyl ether) and concentrated under reduced pressure at a bath temperature of 40°C. The residue was suspended in *tert*-butyl methyl ether and the suspension filtrated through a plug of Celite. The obtained filtrate was concentrated under reduced pressure to yield the crude alkynyl boronate which was immediately used in the next step (diisopropyl alkynyl boronates are prone to polymerization upon standing).

The crude alkynyl boronate was dissolved in toluene (50 mL) and anthranilamide (1.36 g, 10 mmol) was added. The mixture was stirred at 110°C in an open flask for 3h (at this point, ^{1}H NMR of an aliquot usually showed full conversion). The mixture was cooled to room temperature and concentrated under reduced pressure. The residue was washed with pentane and dried under high vacuum to give analytically pure compound. Alternatively, the crude product was purified by flash chromatography.

2-(Hex-1-yn-1-yl)-2,3-dihydro-1*H*-naphtho[1,8-*de*][1,3,2]diazaborinine (1d). Prepared analogously from 1-hexyne (1.15 mL, 821 mg, 10.00 mmol) and 1,8-diaminonaphthalene **(**instead of anthranilamide) as a colorless solid (1.77 g, 71%). This compound turned into a deep purple solid upon storage, even though ^{1}H NMR does not indicate significant degradation. ^{1}H NMR (400 MHz, $CDCl_3$): δ (ppm) 7.09 (dd, *J* = 8.4, 7.2 Hz, 2H), 7.01 (dd, *J* = 8.3, 1.1 Hz, 2H), 6.28 (dd, *J* = 7.4, 1.1 Hz, 2H), 5.77 (brs, 2H), 2.31 (t, *J* = 7.0 Hz, 2H), 1.60-1.52 (m, 2H), 1.51-1.41 (m, 2H), 0.95 (t, *J* = 7.2 Hz, 3H); ^{13}C NMR (101 MHz, $CDCl_3$): δ (ppm) 140.9, 136.4, 127.6, 119.9, 117.9, 105.8 (observed in HMBC experiment), 105.2, 30.6, 22.1, 19.5, 13.7; ^{11}B NMR (128 MHz, $CDCl_3$): δ (ppm) 21.6; IR (neat): $\tilde{\nu}$ (cm^{-1}) 3411, 3380, 2954, 2923, 1860, 2191, 1597, 1505, 1404, 1331, 1195, 1166, 1068, 817, 758; HRMS (ESI): calcd. for $C_{16}H_{18}BN_2$ $[M+H]^+$: 249.1558, found 249.1560.

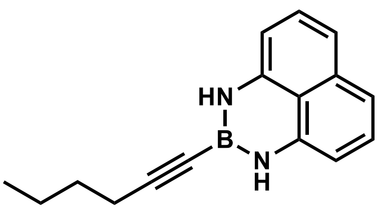

Chemical Formula: $C_{16}H_{17}BN_2$
Molecular Weight: 248,14

2-(Hex-1-yn-1-yl)-2,3-dihydrobenzo[*d*][1,3,2]diazaborinin-4(1*H*)-one (1e). Prepared according to this procedure from 1-hexyne (2.30 mL, 1.64 g, 20.00 mmol). This product was obtained as a colorless solid without flash chromatography (4.29 g, 95%). ^{1}H NMR (400 MHz, $CDCl_3$): δ (ppm) 8.18 (ddt, *J* = 8.0, 1.5, 0.7 Hz, 1H), 7.50 (ddd, *J* = 8.1, 7.3, 1.6 Hz, 1H), 7.13 (ddd, *J* = 8.1, 7.2, 1.0 Hz, 1H), 7.12 (brs, 1H), 6.97 (ddd, *J* = 8.1, 1.1, 0.5 Hz, 1H), 6.56 (brs, 1H), 2.33 (t, *J* = 7.0 Hz, 2H), 1.61-1.52 (m, 2H), 1.51-1.40 (m, 2H), 0.95 (t, *J* = 7.3 Hz, 3H); ^{13}C NMR (101 MHz, $CDCl_3$): δ (ppm) 166.1, 144.2, 134.0, 129.3, 122.1, 119.3, 117.4, 108.0 (observed in HMBC experiment), 30.4, 22.1, 19.5, 13.7; ^{11}B NMR (128 MHz, $CDCl_3$): δ (ppm) 21.7; IR (neat): $\tilde{\nu}$ (cm^{-1}) 3255, 2960, 2932, 2902, 2870, 2197, 1609, 1517, 1357, 1261, 758, 472; HRMS (EI): calcd. for $C_{13}H_{15}BNO$ $[M]^{+\bullet}$: 226.1272, found 226.1274.

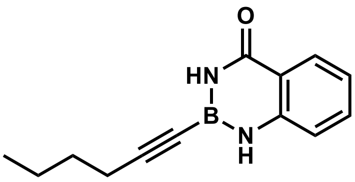

Chemical Formula: $C_{13}H_{15}BN_2O$
Molecular Weight: 226,09

2-(Hex-1-yn-1-yl)-1,3-dimethyl-2,3-dihydrobenzo[*d*][1,3,2]diazaborinin-4(1*H*)-one (1f).

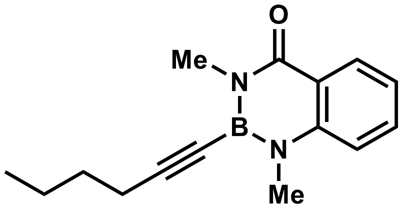

Chemical Formula: $C_{15}H_{19}BN_2O$
Molecular Weight: 254,14

Prepared analogously from 1-hexyne (1.15 mL, 821 g, 10.00 mmol) and *N*-methyl-2-(methylamino)benzamide (instead of anthranilamide) as a colorless solid (847 mg, 33%). ^{1}H NMR (400 MHz, $CDCl_3$): δ (ppm) 8.34 (dd, *J* = 8.2, 1.7 Hz, 1H), 7.59 (ddd, *J* = 8.4, 7.2, 1.6 Hz, 1H), 7.22-7.13 (m, 2H), 3.52 (s, 3H), 3.41 (s, 3H), 2.45 (t, *J* = 7.0 Hz, 2H), 1.70-1.56 (m, 2H), 1.59- 1.44 (m, 2H), 0.97 (t, *J* = 7.3 Hz, 3H); ^{13}C NMR (101 MHz, $CDCl_3$): δ (ppm) 166.6, 145.6, 133.4, 129.7, 121.7, 119.9, 114.2, 112.8, 35.5, 33.0, 30.6, 22.2, 19.8, 13.7; ^{11}B NMR (128 MHz, $CDCl_3$): δ (ppm) 24.1; IR (neat): $\tilde{\nu}$ (cm^{-1}) 2958, 2933, 2864, 2197, 1647, 1604, 1488, 1474, 1389, 1354, 1170, 754; HRMS (EI): calcd. for $C_{15}H_{19}BN_2O$ $[M]^{+\bullet}$: 254.1585, found 254.1588.

2-(Hex-1-yn-1-yl)-3-methyl-2,3-dihydrobenzo[*d*][1,3,2]diazaborinin-4(1*H*)-one (1g). Pre-

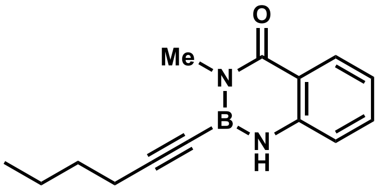

Chemical Formula: $C_{14}H_{17}BN_2O$
Molecular Weight: 240,11

pared analogously from 1-hexyne (1.15 mL, 821 mg, 10.00 mmol) and 2-methylaminobenzamide (instead of anthranilamide) as an off-white solid (1.48 g, 62%). ^{1}H NMR (400 MHz, $CDCl_3$): δ (ppm) 8.27- 8.20 (m, 1H), 7.47 (ddd, *J* = 8.1, 7.2, 1.6 Hz, 1H), 7.12 (ddd, *J* = 8.2, 7.2, 1.1 Hz, 1H), 6.98-6.91 (m, 1H), 6.62 (brs, 1H), 3.37 (s, 3H), 2.38 (t, *J* = 7.0 Hz, 2H), 1.67-1.55 (m, 2H), 1.54- 1.42 (m, 2H), 0.96 (t, *J* = 7.2 Hz, 3H); ^{13}C NMR (101 MHz, $CDCl_3$): δ (ppm) 166.9, 143.1, 133.2, 129.4, 121.9, 119.4, 116.9, 109.9, 32.5, 30.6, 22.1, 19.6, 13.7; ^{11}B NMR (128 MHz, $CDCl_3$): δ (ppm) 23.1; IR (neat): $\tilde{\nu}$ (cm^{-1}) 3316, 1957, 2192, 1628, 1612, 1424, 1314, 763, 701; HRMS (ESI): calcd. for $C_{14}H_{17}BN_2ONa$ $[M+Na]^{+}$: 263.1326, found 263.1324.

2-(Hex-1-yn-1-yl)-1-methyl-2,3-dihydrobenzo[*d*][1,3,2]diazaborinin-4(1*H*)-one (1h). Pre-

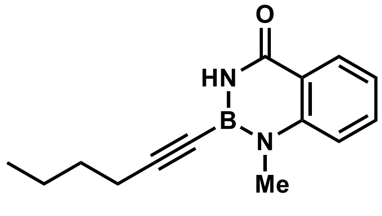

Chemical Formula: $C_{14}H_{17}BN_2O$
Molecular Weight: 240,11

pared analogously from 1-hexyne (1.15 mL, 821 mg, 10.00 mmol) and 2-amino-*N*-methylbenzamide (instead of anthranilamide) as a colorless solid (1.86 g, 77%). ^{1}H NMR (400 MHz, $CDCl_3$): δ (ppm) 8.27 (ddd, *J* = 7.7, 1.8, 0.6 Hz, 1H), 7.62 (ddd, *J* = 8.9, 7.2, 1.8 Hz, 1H), 7.19 (brs, 1H), 7.24-7.13 (m, 1H), 3.47 (s, 3H), 2.38 (t, *J* = 7.0 Hz, 2H), 1.64- 1.54 (m, 2H), 1.54-1.42 (m, 2H), 0.96 (t, *J* = 7.3 Hz, 3H); ^{13}C NMR (101 MHz, $CDCl_3$): δ (ppm) 165.7, 146.5,

134.0, 129.7, 121.7, 120.1, 114.6, 110.3, 35.1, 30.5, 22.1, 19.7, 13.7; ^{11}B NMR (128 MHz, CDCl$_3$): δ (ppm) 22.8; IR (neat): $\tilde{\nu}$ (cm^{-1}) 3182, 3077, 2933, 2199, 1660, 1605, 1482, 1392, 1316, 1283, 770, 634; HRMS (ESI): calcd. for $C_{14}H_{17}BN_2NaO$ [M+Na]$^+$: 263.1326, found 263.1326.

5-Fluoro-2-(hex-1-yn-1-yl)-2,3-dihydrobenzo[*d*][1,3,2]diazaborinin-4(1*H*)-one (1i). Prepared analogously from 1-hexyne (0.57 mL, 411 mg, 5.00 mmol) and 2-amino-6-fluorobenzamide (instead of anthranilamide) as a colorless solid (825 g, 68%). ^{1}H NMR (400 MHz, CDCl$_3$): δ (ppm) ^{1}H NMR (400 MHz, CDCl$_3$) δ 7.40 (td, *J* = 8.2, 5.4 Hz, 1H), 7.11 – 7.04 (m, 1H), 6.83 – 6.72 (m, 2H), 6.62 (m, 1H), 2.32 (t, *J* = 7.0 Hz, 2H), 1.59-1.51 (m, 2H), 1.50-1.39 (m, 2H), 0.94 (t, *J* = 7.3 Hz, 3H); ^{13}C NMR (101 MHz, CDCl$_3$): δ (ppm) 163.6 (d, *J* = 3.5 Hz), 163.6 (d, *J* = 263.0 Hz), 146.4 (d, *J* = 3.0 Hz), 134.3 (d, *J* = 11.5 Hz), 113.3 (d, *J* = 4.0 Hz), 109.6 (d, *J* = 22.6 Hz), 109.0(d, *J* = 7.5 Hz), 108.5, 30.4, 22.1, 19.5, 13.7; ^{19}F NMR (282 MHz, CDCl$_3$): δ (ppm) –109.9, ^{11}B NMR (128 MHz, CDCl$_3$): δ (ppm) 21.7; IR (neat): $\tilde{\nu}$ (cm^{-1}) 3315, 3199, 1960, 2201, 1625, 1519, 1359, 1194, 1050, 775, 459; HRMS (ESI): calcd. for $C_{13}H_{14}BFN_2ONa$ [M+Na]$^+$: 267.1075, found 267.1075.

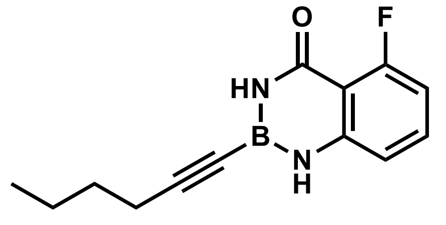

Chemical Formula: $C_{13}H_{14}BFN_2O$
Molecular Weight: 244,08

6-Fluoro-2-(hex-1-yn-1-yl)-2,3-dihydrobenzo[*d*][1,3,2]diazaborinin-4(1*H*)-one (1j). Prepared analogously from 1-hexyne (0.57 mL, 411 g, 5.00 mmol) and 2-amino-5-fluorobenzamide (instead of anthranilamide) as a colorless solid (939 g, 77%). ^{1}H NMR (400 MHz, CDCl$_3$): δ (ppm) 7.89-7.81 (m, 1H), 7.24 (ddd, *J* = 8.9, 7.8, 3.0 Hz, 1H), 7.18 (brs, 1H), 6.96 (dd, *J* = 8.9, 4.3 Hz, 1H), 6.60 (brs, 1H), 2.33 (t, *J* = 7.0 Hz, 2H), 1.62-1.52 (m, 2H), 1.51-1.39 (m, 2H), 0.94 (t, *J* = 7.3 Hz, 3H); ^{13}C NMR (101 MHz, CDCl$_3$): δ (ppm) 165.3 (d, *J* = 2.5 Hz), 158.0 (d, *J* = 241.4 Hz), 140.6 (d, *J* = 2.0 Hz), 121.9 (d, *J* = 24.1 Hz), 120.3 (d, *J* = 7.0 Hz), 118.9 (d, *J* = 7.5 Hz), 114.6 (d, *J* = 23.6 Hz), 108.3, 30.4, 22.1, 19.5, 13.7; ^{19}F NMR (282 MHz, CDCl$_3$): δ (ppm) –120.4, ^{11}B NMR (128 MHz, CDCl$_3$): δ (ppm) 21.4; IR (neat): $\tilde{\nu}$ (cm^{-1}) 3414, 3288, 2924, 2859, 2205, 1645, 1494, 1199, 1175, 825, 760, 715, 666; HRMS (ESI): calcd. for $C_{13}H_{14}BFN_2ONa$ [M+Na]$^+$: 267.1075, found 267.1073.

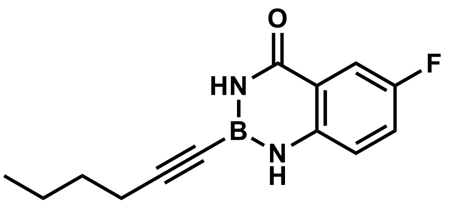

Chemical Formula: $C_{13}H_{14}BFN_2O$
Molecular Weight: 244,08

2-(Prop-1-yn-1-yl)-2,3-dihydrobenzo[*d*][1,3,2]diazaborinin-4(1*H*)-one (4). Prepared analogously from preformed propynyllithium (460 mg, 10.00 mmol) as a yellowish crystalline material after recrystallization form toluene (419 mg, 23%). ^{1}H NMR (400 MHz, $CDCl_3$): δ (ppm) 8.23-8.13 (m, 1H), 7.55-7.46 (m, 1H), 7.17 (brs, 1H), 7.16-7.08 (m, 1H), 6.98 (d, *J* = 8.1 Hz, 1H), 6.61 (brs, 1H), 1.99 (s, 3H); ^{13}C NMR (101 MHz, $CDCl_3$): δ (ppm) 166.2, 144.2, 134.0, 129.3, 122.2, 119.2, 117.4, 103.4 (observed in HMBC experiment), 4.9; ^{11}B NMR (128 MHz, $CDCl_3$): δ (ppm) 21.7; IR (neat): $\tilde{\nu}$ (cm^{-1}) 3271, 3196, 2208, 1609, 1517, 1357, 749, 474; HRMS (ESI): calcd. for $C_{10}H_{10}BN_2O$ $[M+H]^+$: 185.0881, found 185.0881.

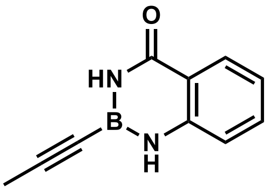

Chemical Formula: $C_{10}H_9BN_2O$
Molecular Weight: 184,01

2-(5-Methylhex-1-yn-1-yl)-2,3-dihydrobenzo[*d*][1,3,2]diazaborinin-4(1*H*)-one (6). Prepared analogously from 5-methyl-1-hexyne (962 mg, 10.00 mmol) as a colorless solid (2.11 g, 88%). ^{1}H NMR (400 MHz, $CDCl_3$): δ (ppm) 8.22-8.15 (m, 1H), 7.50 (ddd, *J* = 8.1, 7.2, 1.6 Hz, 1H), 7.14 (brs, 1H), 7.13 (ddd, *J* = 8.1, 7.2, 1.1 Hz, 1H), 6.97 (dd, *J* = 8.2, 1.1 Hz, 1H), 6.59 (brs, 1H), 2.33 (t, *J* = 7.4 Hz, 2H), 1.80-1.64 (m, 1H), 1.48 (q, *J* = 7.3 Hz, 2H), 0.93 (d, *J* = 6.6 Hz, 6H); ^{13}C NMR (101 MHz, $CDCl_3$): δ (ppm) 166.2, 144.2, 133.9, 129.3, 122.1, 119.3, 117.4, 108.1, 37.3, 27.4, 22.3, 17.8; ^{11}B NMR (128 MHz, $CDCl_3$): δ (ppm) 21.8; IR (neat): $\tilde{\nu}$ (cm^{-1}) 3293, 3200, 2949, 2870, 2201, 1611, 1485, 1356, 1260, 884, 757, 473; HRMS (EI): calcd. for $C_{14}H_{17}BN_2O$ $[M]^{+\bullet}$: 240.1428, found 240.1432.

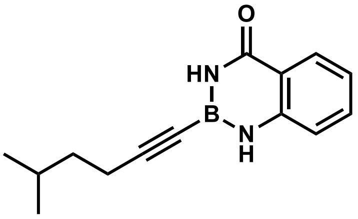

Chemical Formula: $C_{14}H_{17}BN_2O$
Molecular Weight: 240,11

2-(4-Phenylbut-1-yn-1-yl)-2,3-dihydrobenzo[*d*][1,3,2]diazaborinin-4(1*H*)-one (8). Prepared analogously from 4-phenyl-1-butyne (1.41 mL, 1.30 g, 10.00 mmol) as a colorless solid (1.01 g, 37%). ^{1}H NMR (400 MHz, $CDCl_3$): δ (ppm) 8.19 (dd, *J* = 7.9, 1.6 Hz, 1H), 7.50 (ddd, *J* = 8.6, 7.3, 1.6 Hz, 1H), 7.38-7.29 (m, 2H), 7.29-7.21 (m, 3H), 7.13 (ddd, *J* = 8.1, 7.2, 1.1 Hz, 1H), 7.09 (brs, 1H), 6.96 (dd, *J* = 8.1, 1.1 Hz, 1H), 6.54 (brs, 1H), 2.90 (t, *J* = 7.5 Hz, 2H), 2.63 (t, *J* = 7.5 Hz, 2H); ^{13}C NMR (101 MHz, $CDCl_3$): δ (ppm) 166.1, 144.1, 140.2, 134.0, 129.3, 128.7, 128.6, 126.7, 122.2, 119.3, 117.4, 106.8, 34.7, 22.0; ^{11}B NMR (128 MHz, $CDCl_3$): δ (ppm) 21.5; IR (neat): $\tilde{\nu}$ (cm^{-1}) 3233, 3023, 2199, 1608, 1518, 1359, 1259, 1192,

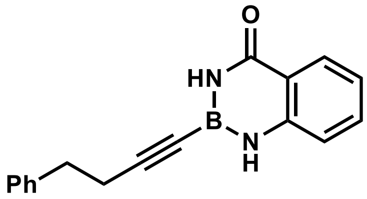

Chemical Formula: $C_{17}H_{15}BN_2O$
Molecular Weight: 274,13

868, 819, 755, 691, 473; HRMS (ESI): calcd. for $C_{17}H_{15}BN_2ONa$ $[M+Na]^+$: 297.1170, found 297.1169.

2-(Cyclopentylethynyl)-2,3-dihydrobenzo[*d*][1,3,2]diazaborinin-4(1*H*)-one (10a). Prepared analogously from cyclopentylacetylene (1.16 mL, 942 mg, 10.00 mmol) as an off-white solid (1.88 g, 79%). ^1H NMR (400 MHz, $CDCl_3$): δ (ppm) 8.21-8.15 (m, 1H), 7.50 (ddd, *J* = 8.2, 7.3, 1.6 Hz, 1H), 7.15 (brs, 1H), 7.13 (td, *J* = 7.7, 1.0 Hz, 1H), 7.02-6.93 (m, 1H), 6.60 (brs, 1H), 2.73 (quint, *J* = 7.6 Hz, 1H), 2.06-1.91 (m, 2H), 1.82-1.73 (m, 2H), 1.73-1.64 (m, 2H), 1.64-1.56 (m, 2H); ^{13}C NMR (101 MHz, $CDCl_3$): δ (ppm) 166.3, 144.3, 134.0, 129.3, 122.1, 119.1, 117.4, 112.3, 33.8, 31.0, 25.3; ^{11}B NMR (128 MHz, $CDCl_3$): δ (ppm) 21.8; IR (neat): $\tilde{\nu}$ (cm^{-1}) 3270, 2942, 2862, 2184, 1612, 1516, 1356, 1258, 757, 471; HRMS (ESI): calcd. for $C_{14}H_{15}BN_2ONa$ $[M+Na]^+$: 261.1170, found 261.1168.

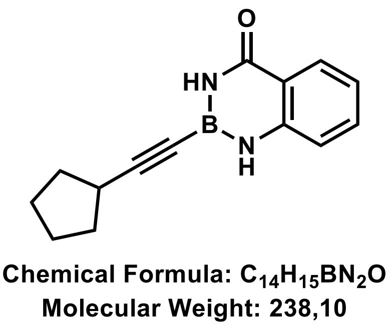

2-(Cyclopentylethynyl)-6-fluoro-2,3-dihydrobenzo[d][1,3,2]diazaborinin-4(1*H*)-one (10b). Prepared analogously from 1-hexyne (0.58 mL, 471 g, 5.00 mmol) and 2-amino-5-fluorobenzamide (instead of anthranilamide) as a yellow solid material (1.11 g, 87%). ^1H NMR (400 MHz, $CDCl_3$): δ (ppm) 7.85 (dd, *J* = 8.9, 3.1 Hz, 1H), 7.23 (ddd, *J* = 8.9, 7.9, 3.0 Hz, 1H), 7.16 (brs, 1H), 6.95 (dd, *J* = 8.9, 4.3 Hz, 1H), 6.59 (brs, 1H), 2.73 (p, *J* = 7.5 Hz, 1H), 2.03-1.92 (m, 2H), 1.81-1.72 (m, 2H), 1.72-1.63 (m, 2H), 1.63-1.55 (m, 2H); ^{13}C NMR (101 MHz, $CDCl_3$): δ (ppm) 165.3 (d, *J* = 3.0 Hz), 157.9 (d, *J* = 241.4 Hz), 140.6 (d, *J* = 2.0 Hz), 121.8 (d, *J* = 24.1 Hz), 120.2 (d, *J* = 7.0 Hz), 118.9 (d, *J* = 7.5 Hz), 114.6 (d, *J* = 23.6 Hz), 33.8, 30.9, 25.3; ^{19}F NMR (282 MHz, $CDCl_3$): δ (ppm) –120.5, ^{11}B NMR (128 MHz, $CDCl_3$): δ (ppm) 21.9; IR (neat): $\tilde{\nu}$ (cm^{-1}) 3414, 3284, 2948, 2869, 2198, 1646, 1494, 1200, 822, 759, 715, 665; HRMS (EI): calcd. for $C_{14}H_{14}BFN_2O$ $[M]^{+\bullet}$: 256.1178, found 256.1177.

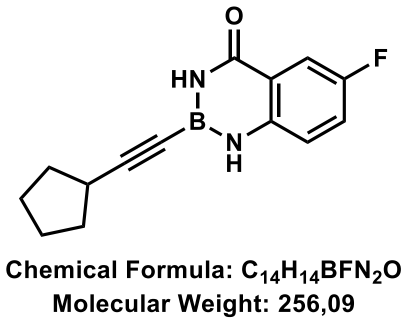

2-(Cyclohexylethynyl)-2,3-dihydrobenzo[*d*][1,3,2]diazaborinin-4(1*H*)-one (12). Prepared analogously from cyclohexylacetylene (1.31 mL, 1.08 g, 10.00 mmol) as an off-white solid material (2.12 g, 84%). ^{1}H NMR (400 MHz, $CDCl_3$): δ (ppm) 8.22-8.14 (m, 1H), 7.50 (ddd, *J* = 8.2, 7.2, 1.6 Hz, 1H), 7.14 (brs, 1H), 7.18-7.08 (m, 1H), 6.97 (d, *J* = 8.2 Hz, 1H), 6.60 (brs, 1H), 2.55-2.44 (m, 1H), 1.89-1.80 (m, 2H), 1.80-1.68 (m, 2H), 1.60-1.43 (m, 3H), 1.42-1.22 (m, 3H); ^{13}C NMR (101 MHz, $CDCl_3$): δ (ppm) 166.1, 144.2, 133.9, 129.3, 122.1, 119.3, 117.4, 112.0, 32.4, 30.0, 25.9, 24.9; ^{11}B NMR (128 MHz, $CDCl_3$): δ (ppm) 21.8; IR (neat): $\tilde{\nu}$ cm^{-1}) 3293, 3206, 2915, 2844, 2193, 1612, 1515, 1484, 1258, 886, 757, 471; HRMS (EI): calcd. for $C_{15}H_{17}BN_2O$ $[M]^{+\bullet}$: 252.1428, found 252.1431.

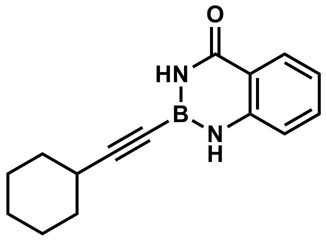

Chemical Formula: $C_{15}H_{17}BN_2O$
Molecular Weight: 252,12

2-(5-Chloropent-1-yn-1-yl)-2,3-dihydrobenzo[*d*][1,3,2]diazaborinin-4(1*H*)-one (14). Prepared analogously from 5-chloro-1-pentyne (1.06 mL, 1.03 g, 10.00 mmol) as an off-white solid (2.08 g, 85%). ^{1}H NMR (400 MHz, $CDCl_3$): δ (ppm) 8.23-8.15 (m, 1H), 7.51 (ddd, *J* = 8.2, 7.3, 1.6 Hz, 1H), 7.21 (brs, 1H), 7.14 (ddd, *J* = 8.1, 7.3, 1.0 Hz, 1H), 7.02-6.96 (m, 1H), 6.67 (brs, 1H), 3.69 (t, *J* = 6.3 Hz, 2H), 2.54 (t, *J* = 6.9 Hz, 2H), 2.03 (p, *J* = 6.7 Hz, 2H); ^{13}C NMR (101 MHz, $CDCl_3$): δ (ppm) 166.1, 144.1, 134.0, 129.3, 122.2, 119.4, 117.5, 105.5, 43.6, 31.0, 17.2; ^{11}B NMR (128 MHz, $CDCl_3$): δ (ppm) 21.6; IR (neat): $\tilde{\nu}$ (cm^{-1}) 3314, 3221, 2203, 1612, 1484, 1260, 759, 473; HRMS (ESI): calcd. for $C_{12}H_{12}BClN_2ONa$ $[M+Na]^{+}$: 269.0623, found 269.0621.

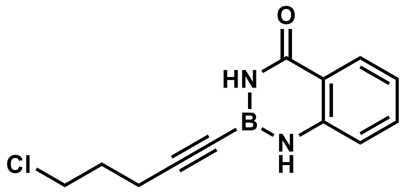

Chemical Formula: $C_{12}H_{12}BClN_2O$
Molecular Weight: 246,50

2-(4-(*tert*-Butyldimethylsilyloxy)but-1-yn-1-yl)-2,3-dihydrobenzo[*d*][1,3,2]diazaborinin-4(1*H*)-one (16). Prepared analogously from 4-(*tert*-butyldimethylsiloxy)-1-butyne (3.10 mL, 2.77 g, 15.00 mmol) without flash column chromatography as a colorless solid (4.56 g, 93%). ^{1}H NMR (400 MHz, $CDCl_3$): δ (ppm) 8.19 (ddd, *J* = 8.0, 1.5, 0.7 Hz, 1H), 7.50 (ddd, *J* = 8.1, 7.2, 1.6 Hz, 1H), 7.14 (ddd, *J* = 8.2, 7.2, 1.1 Hz, 1H), 7.12 (brs, 1H), 7.00-6.94 (m, 1H), 6.57 (brs, 1H), 3.79 (t, *J* = 6.9 Hz, 2H), 2.55 (t, *J* = 6.9 Hz, 2H), 0.92 (s, 9H), 0.10 (s, 6H); ^{13}C NMR (101 MHz, $CDCl_3$): δ (ppm) 166.0, 144.1, 134.0, 129.3, 122.2, 119.4, 117.4, 104.8, 61.6, 26.0, 24.3, 18.5, –5.1; ^{11}B NMR (128 MHz, $CDCl_3$): δ (ppm) 21.6; IR (neat): $\tilde{\nu}$ (cm^{-1}) 3373, 3214, 2952, 2927,

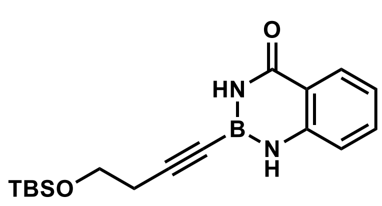

Chemical Formula: $C_{17}H_{25}BN_2O_2Si$
Molecular Weight: 328,29

2855, 2210, 1648, 1613, 1522, 1094, 831, 776, 748; HRMS (ESI): calcd. for $C_{17}H_{25}BN_2O_2SiNa$ $[M+Na]^+$: 351.1671, found 351.1666.

2-(Phenylethynyl)-2,3-dihydrobenzo[*d*][1,3,2]diazaborinin-4(1*H*)-one (18). Prepared analogously from preformed (phenylethynyl)lithium (1.08 g, 10.00 mmol) as a yellowish solid material (956 mg, 39%). ^1H NMR (400 MHz, $CDCl_3$): δ (ppm) 8.25-8.19 (m, 1H), 7.57-7.51 (m, 3H), 7.42-7.34 (m, 3H), 7.31 (brs, 1H), 7.17 (ddd, *J* = 8.1, 7.3, 1.0 Hz, 1H), 7.08-7.00 (m, 1H), 6.75 (brs, 1H); ^{13}C NMR (101 MHz, $CDCl_3$): δ (ppm) 166.1, 144.1, 134.1, 132.4, 129.8, 129.4, 128.7, 122.4, 121.9, 119.4, 117.5, 104.9 (observed in HMBC experiment); ^{11}B NMR (128 MHz, $CDCl_3$): δ (ppm) 22.1; IR (neat): $\tilde{\nu}$ (cm^{-1}) 3405, 3305, 3234, 2186, 1660, 1513, 1482, 1269, 1148, 754, 685, 469; HRMS (ESI): calcd. for $C_{15}H_{11}BN_2ONa$ $[M+Na]^+$: 269.0857, found 269.0855.

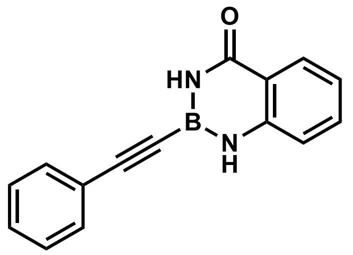

Chemical Formula: $C_{15}H_{11}BN_2O$
Molecular Weight: 246,08

2-(Cyclopropylethynyl)-2,3-dihydrobenzo[*d*][1,3,2]diazaborinin-4(1*H*)-one (20). Prepared analogously from cyclopropylacetylene (0.85 mL, 661 mg, 10.00 mmol) as a colorless solid (944 mg, 45%). ^1H NMR (400 MHz, $CDCl_3$): δ (ppm) 8.21-8.14 (m, 1H), 7.53-7.44 (m, 1H), 7.15-7.10 (m, 1H), 7.11 (brs, 1H), 6.96 (d, *J* = 8.2 Hz, 1H), 6.58 (brs, 1H), 1.43-1.31 (m, 1H), 0.95-0.84 (m, 2H), 0.87-0.78 (m, 2H); ^{13}C NMR (101 MHz, $CDCl_3$): δ (ppm) 166.1, 144.2, 133.9, 129.3, 122.1, 119.3, 117.4, 110.6, 9.2, 0.4; ^{11}B NMR (128 MHz, $CDCl_3$): δ (ppm) 21.6; IR (neat): $\tilde{\nu}$ (cm^{-1}) 3395, 3384, 3285, 2197, 1646, 1611, 1517, 1483, 1354, 1259, 760, 738; HRMS (ESI): calcd. for $C_{12}H_{11}BN_2ONa$ $[M+Na]^+$: 233.0857, found 233.0856.

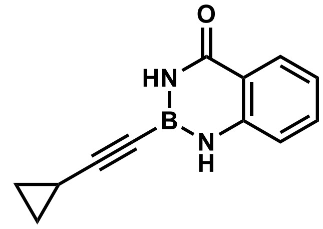

Chemical Formula: $C_{12}H_{11}BN_2O$
Molecular Weight: 210,04

2-(3-Methoxyprop-1-yn-1-yl)-2,3-dihydrobenzo[*d*][1,3,2]diazaborinin-4(1*H*)-one (22). Prepared analogously from methyl propargyl ether (0.84 mL, 700 mg, 10.00 mmol) as an off-white solid (696 mg, 33%). ^1H NMR (400 MHz, $CDCl_3$): δ (ppm) 8.23-8.16 (m, 1H), 7.52 (ddd, *J* = 8.1, 7.2, 1.6 Hz, 1H), 7.21 (brs, 1H), 7.16 (ddd, *J* = 8.2, 7.2, 1.1 Hz, 1H), 7.00 (d, *J* = 8.1 Hz, 1H), 6.69 (brs, 1H), 4.21 (s, 2H), 3.44 (s, 3H); ^{13}C NMR (101 MHz, $CDCl_3$): δ (ppm) 165.9, 143.9, 134.1, 129.4, 122.4, 119.5, 117.5, 101.5, 60.4, 58.2; ^{11}B NMR (128 MHz,

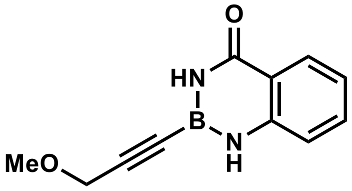

Chemical Formula: $C_{11}H_{11}BN_2O_2$
Molecular Weight: 214,03

$CDCl_3$): δ (ppm) 21.8; IR (neat): $\tilde{\nu}$ (cm^{-1}) 3334, 3184, 2205, 1658, 1614, 1521, 1486, 1179, 1093, 904, 771, 472; HRMS (ESI): calcd. for $C_{11}H_{11}BN_2O_2Na$ $[M+Na]^+$: 237.0806, found 237.0804.

2-(3,3-Diethoxyprop-1-yn-1-yl)-2,3-dihydrobenzo[*d*][1,3,2]diazaborinin-4(1*H*)-one (24).

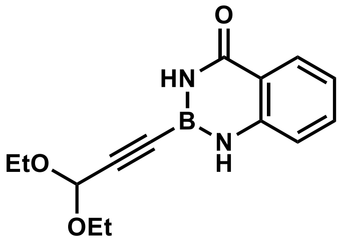

Chemical Formula: $C_{14}H_{17}BN_2O_3$
Molecular Weight: 272,11

Prepared analogously from propargylaldehyde diethyl acetal (1.00 mL, 897 mg, 7.00 mmol) as a yellow solid material (1.20 g, 63%). ^1H NMR (400 MHz, $CDCl_3$): δ (ppm) 8.23-8.16 (m, 1H), 7.52 (ddd, *J* = 8.1, 7.2, 1.6 Hz, 1H), 7.21 (brs, 1H), 7.16 (ddd, *J* = 8.2, 7.3, 1.1 Hz, 1H), 7.02-6.95 (m, 1H), 6.70 (brs, 1H), 5.35 (s, 1H), 3.79 (dq, *J* = 9.4, 7.1 Hz, 2H), 3.64 (dq, *J* = 9.4, 7.0 Hz, 2H), 1.27 (t, *J* = 7.1 Hz, 6H); ^{13}C NMR (101 MHz, $CDCl_3$): δ (ppm) 165.7, 143.8, 134.1, 129.4, 122.6, 119.5, 117.6, 100.1, 91.3, 61.4, 15.2; ^{11}B NMR (128 MHz, $CDCl_3$): δ (ppm) 21.6; IR (neat): $\tilde{\nu}$ (cm^{-1}) 3271, 2927, 2224, 1612, 1519, 1485, 1360, 1180, 1046, 1005, 803, 761, 475; HRMS (ESI): calcd. for $C_{14}H_{17}BN_2O_3Na$ $[M+Na]^+$: 295.1224, found 295.1228.

2-(3-(Trimethylsilyl)prop-1-yn-1-yl)-2,3-dihydrobenzo[*d*][1,3,2]diazaborinin-4(1*H*)-one (26a).

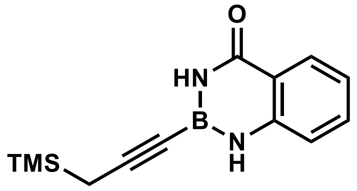

Chemical Formula: $C_{13}H_{17}BN_2OSi$
Molecular Weight: 256,19

Prepared analogously from trimethyl(propargyl)silane (0.60 mL, 449 mg, 4.00 mmol) as a colorless solid (742 mg, 72%). ^1H NMR (400 MHz, $CDCl_3$): δ (ppm) 8.22-8.14 (m, 1H), 7.49 (dd, *J* = 1.6, 0.9 Hz, 1H), 7.12 (ddd, *J* = 8.2, 7.2, 1.1 Hz, 1H), 7.11 (brs, 1H), 7.00-6.93 (m, 1H), 6.52 (brs, 1H), 1.66 (s, 2H), 0.16 (s, 9H); ^{13}C NMR (101 MHz, $CDCl_3$): δ (ppm) 166.3, 144.3, 133.9, 129.3, 122.0, 119.2, 117.3, 106.7, 9.0, –1.9; ^{11}B NMR (128 MHz, $CDCl_3$): δ (ppm) 21.9; IR (neat): $\tilde{\nu}$ (cm^{-1}) 3328, 3260, 2954, 2199, 2168, 1649, 1613, 1521, 1487, 1357, 1249, 843; HRMS (EI): calcd. for $C_{13}H_{17}BN_2OSi$ $[M]^{+\bullet}$: 256.1198, found 256.1197.

6-Fluoro-2-(3-(trimethylsilyl)prop-1-yn-1-yl)-2,3-dihydrobenzo[d][1,3,2]diazaborinin-4(1*H*)-one (26b). Prepared analogously from trimethyl(propargyl)silane (0.82 mL, 620 mg, 5.52 mmol) and 2-amino-5-fluorobenzamide (instead of anthranilamide) as a pale yellow solid material (1.06 g, 70%). ^{1}H NMR (400 MHz, $CDCl_3$): δ (ppm) 7.84 (dd, *J* = 8.9, 3.1 Hz, 1H), 7.22 (ddd, *J* = 8.8, 7.8, 3.0 Hz, 1H), 7.17 (br s, 1H), 6.95 (dd, *J* = 8.8, 4.3 Hz, 1H), 6.57 (br s, 1H), 1.66 (s, 2H), 0.15 (s, 9H); ^{13}C NMR (101 MHz, $CDCl_3$): δ (ppm) 165.4 (d, *J* = 2.8 Hz), 157.8 (d, *J* = 241.4 Hz), 140.7 (d, *J* = 1.9 Hz), 121.8 (d, *J* = 24.1 Hz), 120.2 (d, *J* = 7.3 Hz), 118.8 (d, *J* = 7.5 Hz), 114.5 (d, *J* = 23.6 Hz), 106.9, 9.0, –1.9; ^{19}F NMR (282 MHz, $CDCl_3$): δ (ppm) –120.7; ^{11}B NMR (128 MHz, $CDCl_3$): δ (ppm) 21.7; IR (neat): $\tilde{\nu}$ (cm^{-1}) 3290 (br), 2957, 2194, 2173, 1659, 1598, 1520, 1498, 1340, 1251, 850; HRMS (ESI): calcd. for $C_{13}H_{15}BFN_2OSi$ [M-H]$^-$: 273.1036, found 273.1036.

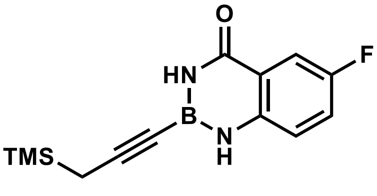

Chemical Formula: $C_{13}H_{16}BFN_2OSi$
Molecular Weight: 274.18

***tert*-Butyl 2-(3-(4-oxo-3,4-dihydrobenzo[*d*][1,3,2]diazaborinin-2(1*H*)-yl)prop-2-yn-1-yl)-piperidine-1-carboxylate (28).** Prepared analogously from *tert*-butyl 2-(prop-2-yn-1-yl)piperidine-1-carboxylate (1.12 g, 5.00 mmol)[8] as a colorless solid material (1.43 g, 78%). $[\alpha]_D^{20}$ = +4.5° (c = 1.21, $CHCl_3$). ^{1}H NMR (400 MHz, $CDCl_3$): δ (ppm) 8.21-8.14 (m, 1H), 7.50 (ddd, *J* = 8.2, 7.2, 1.6 Hz, 1H), 7.13 (ddd, *J* = 8.1, 7.2, 1.0 Hz, 1H), 7.12 (brs, 1H), 7.01-6.94 (m, 1H), 6.67 (brs, 1H), 4.52-4.47 (m, 1H), 4.08-3.96 (m, 1H), 2.81-2.70 (m, 1H), 2.58 (dd, *J* = 7.8, 2.9 Hz, 2H), 1.87-1.78 (m, 1H), 1.71-1.59 (m, 4H), 1.46 (s, 9H), 1.52-1.41 (m, 1H); ^{13}C NMR (101 MHz, $CDCl_3$): δ (ppm)b 166.1, 155.2, 144.2, 134.0, 129.3, 122.1, 119.3, 117.4, 104.6, 79.9, 49.5, 39.3, 28.6, 27.3, 25.3, 21.1, 18.8; ^{11}B NMR (128 MHz, $CDCl_3$): δ (ppm) 21.6; IR (neat): $\tilde{\nu}$ (cm^{-1}) 3257, 2944, 2212, 1680, 1651, 1613, 1488, 1416, 1266, 1162, 875, 759, 478; HRMS (ESI): calcd. for $C_{20}H_{26}BN_3O_3Na$ [M+Na]$^+$: 390.1959, found 390.1960; IR (neat): $\tilde{\nu}$ (cm^{-1}) 3291, 2954, 2932, 2875, 2207, 1657, 1614, 1512, 1486, 1109, 734, 700, 503;

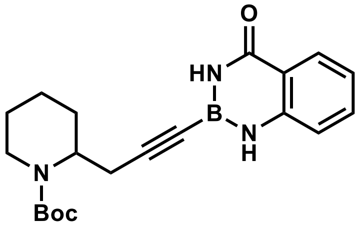

Chemical Formula: $C_{20}H_{26}BN_3O_3$
Molecular Weight: 367,26

(*R*)-2-(5-(*tert*-Butyldiphenylsilyloxy)-4-(triethylsilyloxy)pent-1-yn-1-yl)-2,3-dihydro-benzo[*d*][1,3,2]diazaborinin-4(1*H*)-one (30). Prepared analogously from (*R*)-5-((*tert*-butyldiphenylsilyl)oxy)-4-(triethylsilyloxy)-1-pentyne (1.36 g, 3.00 mmol)[6] as a yellow wax (1.62 g, 91%). ^{1}H NMR (400 MHz, $CDCl_3$): δ (ppm) 8.20 (dd, *J* = 8.0, 1.6 Hz, 1H), 7.74-6.63 (m, 4H), 7.51 (ddd, *J* = 8.5, 7.2, 1.6 Hz, 1H), 7.47-7.34 (m, 6H), 7.18-7.10 (m, 1H), 7.07 (brs, 1H), 6.95 (dd, *J* = 8.2, 1.1 Hz, 1H), 6.50 (brs, 1H), 3.98-3.87 (m, 1H), 3.65 (qd, *J* = 10.1, 5.6 Hz, 2H), 2.75 (dd, *J* = 17.0, 5.4 Hz, 1H), 2.55 (dd, *J* = 17.0, 5.8 Hz, 1H), 1.07 (s, 9H), 0.94 (t, *J* = 8.0 Hz, 9H), 0.58 (q, *J* = 8.1 Hz, 6H); ^{13}C NMR (101 MHz, $CDCl_3$): δ (ppm) 166.0, 144.1, 135.8, 135.7, 133.9, 133.5, 133.5, 129.9, 129.3, 127.9, 127.8, 122.1, 119.3, 117.4, 104.8, 71.4, 67.0, 27.0, 25.8, 19.4, 7.0, 5.0; ^{11}B NMR (128 MHz, $CDCl_3$): δ (ppm) 21.5. HRMS (ESI): calcd. for $C_{34}H_{45}BN_2O_3Si_2Na$ $[M+Na]^+$: 619.2954, found 619.2963.

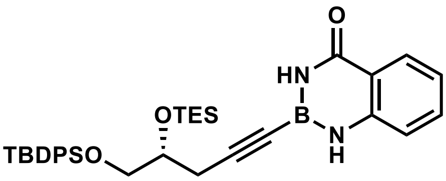

Chemical Formula: $C_{34}H_{45}BN_2O_3Si_2$
Molecular Weight: 596.73

2-(3-(2,3,4,6-Tetra-*O*-(*tert*-butyldimethylsilyl)-*α*-*D*-glucopyranosyl)prop-1-yn-1-yl)-2,3-dihydrobenzo[*d*][1,3,2]diazaborinin-4(1*H*)-one (32). Prepared analogously from 3-(2,3,4,6-tetra-*O*-(*tert*-butyldimethylsilyl)-*α*-*D*-glucopyranosyl)-1-propyne (357 mg, 0.54 mmol)[7] as a colorless solid (387 mg, 89%). $[\alpha]_D^{20}$ = +16.3° (c = 0.78, $CHCl_3$). ^{1}H NMR (400 MHz, $CDCl_3$): δ (ppm) 8.22-8.15 (m, 1H), 7.51 (ddd, *J* = 8.1, 7.2, 1.6 Hz, 1H), 7.14 (ddd, *J* = 8.1, 7.2, 1.1 Hz, 1H), 7.04 (brs, 1H), 6.99-6.92 (m, 1H), 6.53 (brs, 1H), 4.01 (td, *J* = 7.7, 7.2, 2.0 Hz, 1H), 3.90-3.80 (m, 3H), 3.79-3.72 (m, 3H), 2.61 (dd, *J* = 7.3, 1.7 Hz, 2H), 0.94 (s, 9H), 0.91 (s, 9H), 0.89 (s, 9H), 0.89 (s, 9H), 0.14 (s, 6H), 0.13 (s, 6H), 0.10 (s, 3H), 0.08 (s, 3H), 0.06 (s, 6H); ^{13}C NMR (101 MHz, $CDCl_3$): δ (ppm) 166.0, 144.1, 134.0, 129.4, 122.2, 119.4, 117.4, 104.8, 78.0, 74.7, 71.0, 70.5, 69.0, 62.6, 26.3, 26.1, 26.1, 25.9, 22.8, 18.5, 18.5, 18.3, 18.1, –3.4, –3.9, –3.9, –4.5, –4.6, –4.8, –4.8, –5.1; ^{11}B NMR (128 MHz, $CDCl_3$): δ (ppm) 21.6; IR (neat): $\tilde{\nu}$ (cm^{-1}) 2952, 2928, 2886, 2856, 2206, 1661, 1252, 1086, 831, 774; HRMS (ESI): calcd. for $C_{40}H_{75}BN_2O_6Si_4Na$ $[M+Na]^+$: 825.4688, found 825.4696.

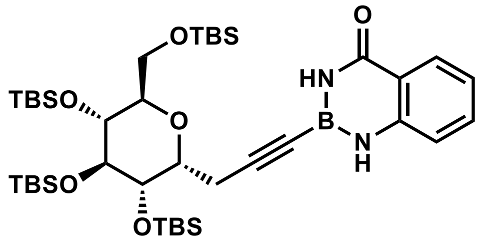

Chemical Formula: $C_{40}H_{75}BN_2O_6Si_4$
Molecular Weight: 803,20

2-(Cyclohex-1-en-1-ylethynyl)-2,3-dihydrobenzo[*d*][1,3,2]diazaborinin-4(1*H*)-one (34).

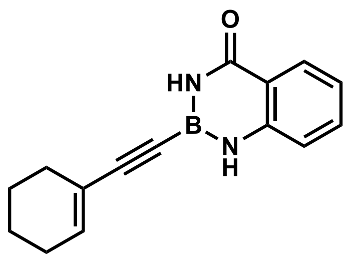

Chemical Formula: $C_{15}H_{15}BN_2O$
Molecular Weight: 250,11

Prepared analogously from 1-ethynyl-1-cyclohexene (1.18 mL, 1.06 g, 10.00 mmol) as a colorless solid material (1.46 mg, 58%). ^{1}H NMR (400 MHz, $CDCl_3$): δ (ppm) 8.19 (dd, *J* = 8.0, 1.6 Hz, 1H), 7.50 (ddd, *J* = 8.6, 7.3, 1.6 Hz, 1H), 7.14 (brs, 1H), 7.14 (ddd, *J* = 8.1, 7.2, 1.1 Hz, 1H), 6.98 (dd, *J* = 8.1, 1.0 Hz, 1H), 6.60 (brs, 1H), 6.32 (tt, *J* = 3.8, 1.7 Hz, 1H), 2.24-2.10 (m, 4H), 1.73-1.56 (m, 4H); ^{13}C NMR (101 MHz, $CDCl_3$): δ (ppm) 166.0, 144.2, 139.0, 134.0, 129.3, 122.2, 120.2, 119.4, 117.4, 107.1, 28.9, 26.0, 22.2, 21.4; ^{11}B NMR (128 MHz, $CDCl_3$): δ (ppm) 22.1; IR (neat): $\tilde{\nu}$ (cm^{-1}) 3274, 3189, 2930, 2859, 2822, 2170, 1616, 1515, 1483, 1259, 754, 474; HRMS (EI): calcd. for $C_{15}H_{15}BN_2O$ $[M]^{+\bullet}$: 250.1272, found 250.1275.

2-((1-((Tetrahydro-2*H*-pyran-2-yl)oxy)cyclohexyl)ethynyl)-2,3-dihydrobenzo[*d*][1,3,2]diazaborinin-4(1*H*)-one (35).

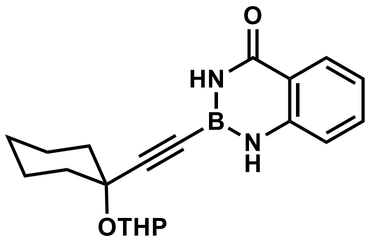

Chemical Formula: $C_{20}H_{25}BN_2O_3$
Molecular Weight: 352,24

Prepared analogously from 2-((1-ethynylcyclohexyl)oxy)tetrahydro-2*H*-pyran (1.04 g, 5.00 mmol)[9] as a colorless solid material (856 mg, 49%). ^{1}H NMR (400 MHz, $CDCl_3$): δ (ppm) 8.19 (dd, *J* = 8.0, 1.5 Hz, 1H), 7.52 (ddd, *J* = 8.5, 7.2, 1.6 Hz, 1H), 7.21 (brs, 1H), 7.18-7.11 (m, 1H), 7.00 (dd, *J* = 8.1, 1.1 Hz, 1H), 6.67 (brs, 1H), 5.16 (dd, *J* = 5.0, 3.1 Hz, 1H), 4.04-3.94 (m, 1H), 3.58-3.48 (m, 1H), 2.11-2.02 (m, 1H), 1.95-1.84 (m, 2H), 1.79-1.68 (m, 4H), 1.67-1.52 (m, 8H), 1.36-1.22 (m, 1H); ^{13}C NMR (101 MHz, $CDCl_3$): δ (ppm) 166.0, 144.0, 134.0, 129.3, 122.4, 122.3, 119.4, 117.5, 107.7, 95.7, 63.4, 39.7, 38.5, 32.2, 25.5, 25.3, 25.2, 23.3, 23.2, 23.2, 20.3; ^{11}B NMR (128 MHz, $CDCl_3$): δ (ppm) 21.8; IR (neat): $\tilde{\nu}$ (cm^{-1}) 3247, 2931, 2857, 2201, 1643, 1619, 1524, 1486, 1361, 1019, 986, 755, 475; HRMS (ESI): calcd. for $C_{20}H_{25}BN_2O_3Na$ $[M+Na]^+$: 375.1850, found 375.1854.

2-((Triisopropylsilyl)ethynyl)-2,3-dihydrobenzo[*d*][1,3,2]diazaborinin-4(1*H*)-one (36).

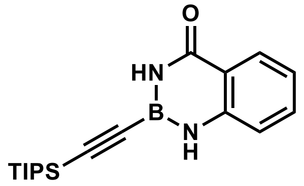

Chemical Formula: $C_{18}H_{27}BN_2OSi$
Molecular Weight: 326,32

Prepared analogously from (triisopropylsilyl)acetylene (1.12 mL, 912 mg, 5.00 mmol) as a colorless solid material (1.58 g, 97%). ^{1}H NMR (400 MHz, $CDCl_3$): δ (ppm) 8.28-8.05 (m, 1H), 7.52 (ddd, *J* = 8.1, 7.2, 1.6 Hz, 1H), 7.20 (brs, 1H), 7.15 (ddd, *J* = 8.2, 7.2, 1.1 Hz, 1H), 7.00 (dt, *J* = 8.1, 0.8 Hz, 1H), 6.63 (brs, 1H), 1.12 (s, 21H); ^{13}C NMR (101 MHz, $CDCl_3$): δ (ppm) 166.0, 144.0, 134.0, 129.3, 122.3, 119.5, 117.5, 111.0, 18.7, 11.2; ^{11}B NMR (128 MHz, $CDCl_3$): δ (ppm) 21.1; IR (neat): $\tilde{\nu}$ (cm^{-1}) 3446, 3393, 3189, 2941, 2862, 1618, 1510, 1486, 1157, 881. 677, 630, 473; HRMS (EI): calcd. for $C_{20}H_{25}BN_2O_3Na$ $[M]^{+\bullet}$: 326.1980, found 326.1978.

2-(3-Chloroprop-1-yn-1-yl)-2,3-dihydrobenzo[*d*][1,3,2]diazaborinin-4(1*H*)-one (37). Prepared analogously from a solution of propargyl chloride in toluene (70% *w/w*, 1.11 mL, 1.06 g, 10.00 mmol) as a yellow solid material (1.98 g, 91%). ^{1}H NMR (400 MHz, $CDCl_3$): δ (ppm) 8.24- 8.17 (m, 1H), 7.53 (ddd, *J* = 8.1, 7.2, 1.6 Hz, 1H), 7.26 (brs, 1H), 7.17 (ddd, *J* = 8.2, 7.2, 1.1 Hz, 1H), 7.06- 6.98 (m, 1H), 6.77 (brs, 1H), 4.23 (s, 2H); ^{13}C NMR (101 MHz, $CDCl_3$): δ (ppm) 165.8, 143.8, 134.1, 129.4, 122.6, 119.5, 117.6, 99.6, 30.3; ^{11}B NMR (128 MHz, $CDCl_3$): δ (ppm) 21.7; IR (neat): $\tilde{\nu}$ (cm^{-1}) 3278, 3193, 2216, 1612, 1520, 1485, 1363, 1261, 756, 701, 474; HRMS (EI): calcd. for $C_{12}H_{11}BN_2ONa$ $[M]^{+\bullet}$: 218.0427, found 218.0415.

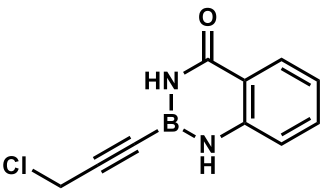

Chemical Formula: $C_{10}H_8BClN_2O$
Molecular Weight: 218,45

***trans*-Selective Hydrostannation of Alkynyl-B(aam) Derivatives. General Procedure (Small Scale).** A 10 mL flame-dried Schlenk tube was charged under argon with the selected alkyne-B(aam) derivative (0.5 mmol), $[Cp^*RuCl]_4$ (13.6 mg, 12.5 µmol, 2.5 mol%) and CH_2Cl_2 (2.5 mL). Bu_3SnH (0.14 mL, 0.525 mmol) was then slowly added (ca. one drop every 5 sec). Once the addition was complete, the mixture was stirred for 30 min and the conversion was checked by TLC. The mixture was concentrated under reduced pressure and the crude mixture analyzed by ^{1}H NMR to determine the isomer . The residue was purified by flash chromatography (hexane/EtOAc) to yield the desired hydrostannated product.

Larger Scale Experiments. A 50 mL flame-dried Schlenk tube was charged under argon atmosphere with the selected alkyne-B(aam) derivative (5.0 mmol), $[Cp^*RuCl]_4$ (136 mg, 125

µmol, 2.5 mol%) and CH_2Cl_2 (25 mL). The resulting solution was cooled to 0°C in an ice bath before Bu_3SnH (1.4 mL, 5.25 mmol) was added over the course of 5 min via syringe pump. Once the addition was complete, the reaction was stirred for 30 min at 0°C and the conversion was checked by TLC. The mixture was then transferred in a 100 mL flask (ringing with CH_2Cl_2) and concentrated under reduced pressure. The crude product was analyzed by ^{1}H NMR to determine the isomer . The residue was suspended in pentane, sonicated and the mixture filtrated through a pad of Celite® in order to remove most of the catalyst decomposition products. The filtrate was concentrated and the residue purified by flash chromatography (hexane/EtOAc) to yield the desired hydrostannated product.

(*Z*)-2-(1-(Tributylstannyl)hex-1-en-1-yl)-2,3-dihydrobenzo[*d*][1,3,2]diazaborinin-4(1*H*)-one (2e). Prepared accordingly as a brownish oil (2.26 g, 4.4 mmol, 88%, α/β = 96:4, *Z*/*E* > 99:1). ^{1}H NMR (400 MHz, $CDCl_3$): δ (ppm) 8.21-8.16 (m, 1H), 7.49 (ddd, *J* = 8.1, 7.2, 1.6 Hz, 1H), 7.11 (ddd, *J* = 8.1, 7.3, 1.0 Hz, 1H), 6.98-6.93 (m, 1H), 6.91 (brs, 1H), 6.86 (t, *J* = 6.7 Hz, J_{Sn-H} = 152.5 Hz, 1H), 6.15 (brs, 1H), 2.22-2.10 (m, 2H), 1.53-1.24 (m, 16H), 1.02-0.91 (m, 9H), 0.87 (t, *J* = 7.3 Hz, 9H); ^{13}C NMR (101 MHz, $CDCl_3$): δ (ppm) 166.7, 156.9, 144.6, 137.8 (C(sp^2)-B by HMBC), 133.8, 129.3, 121.5, 118.7, 117.4, 39.0, 31.8, 29.4, 27.5, 22.7, 14.2, 13.8, 11.2; ^{11}B NMR (128 MHz, $CDCl_3$): δ (ppm) 30.8, ^{119}Sn NMR (149 MHz, $CDCl_3$): δ (ppm) –52.0; IR (neat): $\tilde{\nu}$ (cm^{-1}) 3305, 2954, 2923, 2853, 1654, 1613, 1509, 1485, 1341, 1162, 759; HRMS (ESI): calcd. for $C_{25}H_{43}BN_2OSnNa$ [M+Na]$^+$: 541.2383, found 541.2380.

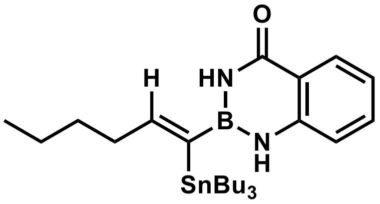

Chemical Formula: $C_{25}H_{43}BN_2OSn$
Molecular Weight: 517,15

(*Z*)-5-Fluoro-2-(1-(tributylstannyl)hex-1-en-1-yl)-2,3-dihydrobenzo[*d*][1,3,2]diazaborinin-4(1*H*)-one (2i). Prepared analogously as a brownish oil (232.9 mg, 87%, α/β = 98:2, *Z*/*E* > 99:1). ^{1}H NMR (400 MHz, $CDCl_3$): δ (ppm) 7.39 (td, *J* = 8.1, 5.3 Hz, 1H), 6.85 (t, *J* = 6.7 Hz, 1H), 6.84 (brs, 1H), 6.77-6.72 (m, 2H), 6.20 (brs, 1H), 2.21-2.08 (m, 2H), 1.51-1.43 (m, 6H), 1.42-1.34 (m, 4H), 1.32-1.27 (m, 6H), 1.00-0.89 (m, 9H), 0.87 (t, *J* = 7.4 Hz, 9H); ^{13}C NMR (101 MHz, $CDCl_3$): δ (ppm) 164.3 (d, *J* = 3.5 Hz), 163.6 (d, *J* = 262.5 Hz), 157.3, 146.9 (d, *J* = 3.0 Hz), 137.1 (C(sp^2)-B by HMBC), 134.1 (d, *J* = 11.5 Hz), 113.2 (d, *J* = 4.0 Hz), 108.9 (d, *J* = 22.1 Hz), 108.3 (d, *J* = 8.0 Hz), 39.0, 31.7, 29.4, 27.5, 22.7, 14.2,

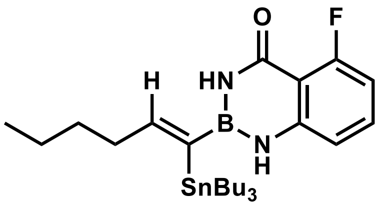

Chemical Formula: $C_{25}H_{42}BFN_2OSn$
Molecular Weight: 535,14

13.8, 11.1; ^{19}F NMR (282 MHz, $CDCl_3$): δ (ppm) −110.4, ^{11}B NMR (128 MHz, $CDCl_3$): δ (ppm) 30.7; ^{119}Sn NMR (149 MHz, $CDCl_3$): δ (ppm) −51.9; IR (neat): $\tilde{\nu}$ (cm^{-1}) 3407, 3311, 2955, 2923, 2871, 2854, 1656, 1626, 1515, 1044, 817, 666, 459; HRMS (ESI): calcd. for $C_{25}H_{41}BFN_2OSn$ $[M\text{-}H]^-$: 535.2323, found 535.2325.

(*Z*)-6-Fluoro-2-(1-(tributylstannyl)hex-1-en-1-yl)-2,3-dihydrobenzo[*d*][1,3,2]diazaborinin-4(1*H*)-one (2j). Prepared analogously as a brownish oil (266.1 mg, 99%, *α*/*β* = 98:2, *Z*/*E* > 99:1). ^1H NMR (400 MHz, $CDCl_3$): δ (ppm) 7.85 (dd, *J* = 8.9, 3.1 Hz, 1H), 7.22 (ddd, *J* = 8.9, 7.9, 3.0 Hz, 1H), 7.01 (brs, 1H), 6.94 (dd, *J* = 8.9, 4.3 Hz, 1H), 6.86 (t, *J* = 6.8 Hz, 1H), 6.19 (brs, 1H), 2.21-2.10 (m, 2H), 1.52-1.44 (m, 6H), 1.45-1.33 (m, 4H), 1.33-1.25 (m, 6H), 1.00-0.91 (m, 9H), 0.86 (t, *J* = 7.3 Hz, 9H); ^{13}C NMR (101 MHz, $CDCl_3$): δ (ppm) 165.9 (d, *J* = 2.5 Hz), 157.6 (d, *J* = 240.4 Hz), 157.0, 141.0, 137.5 (C(sp^2)-B by HMBC), 121.6 (d, *J* = 24.1 Hz), 119.6 (d, *J* = 7.5 Hz), 118.8 (d, *J* = 7.5 Hz), 114.4 (d, *J* = 23.6 Hz), 39.0, 31.7, 29.4, 27.5, 22.7, 14.2, 13.8, 11.1; ^{19}F NMR (282 MHz, $CDCl_3$): δ (ppm) −121.5, ^{11}B NMR (128 MHz, $CDCl_3$): δ (ppm) 30.7; ^{119}Sn NMR (149 MHz, $CDCl_3$): δ (ppm) −51.9; IR (neat): $\tilde{\nu}$ (cm^{-1}) 3440, 3405, 3306, 2955, 2923, 2871, 2853, 1652, 1597, 1497, 827; HRMS (ESI): calcd. for $C_{25}H_{41}BFN_2OSn$ $[M\text{-}H]^-$: 535.2323, found 535.2325.

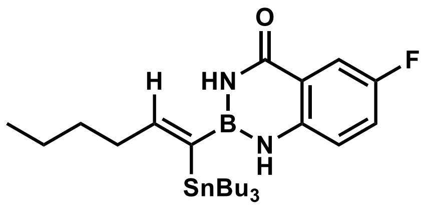

Chemical Formula: $C_{25}H_{42}BFN_2OSn$
Molecular Weight: 535,14

(*Z*)-2-(1-(Tributylstannyl)prop-1-en-1-yl)-2,3-dihydrobenzo[*d*][1,3,2]diazaborinin-4(1*H*)-one (5). Prepared analogously as a brownish oil (237.5 mg, quant., *α*/*β* > 99:1, *Z*/*E* > 99:1). ^1H NMR (400 MHz, $CDCl_3$): δ (ppm) 8.22-8.15 (m, 1H), 7.48 (ddd, *J* = 8.2, 7.2, 1.6 Hz, 1H), 7.10 (td, *J* = 7.7, 1.0 Hz, 1H), 6.97 (q, *J* = 6.3 Hz, $J_{Sn\text{-}H}$ = 151.3 Hz, 1H), 6.98-6.94 (m, 1H), 6.94 (brs, 1H), 6.20 (brs, 1H), 1.92 (d, *J* = 6.3 Hz, 3H), 1.58-1.42 (m, 6H), 1.45-1.21 (m, 6H), 1.04-0.95 (m, 6H), 0.87 (t, *J* = 7.3 Hz, 9H); ^{13}C NMR (101 MHz, $CDCl_3$): δ (ppm) 166.7, 150.8, 144.6, 139.5 (C(sp^2)-B by HMBC), 133.8, 129.3, 121.5, 118.7, 117.4, 29.4, 27.5, 24.3, 13.8, 11.0; ^{11}B NMR (128 MHz, $CDCl_3$): δ (ppm) 30.8, ^{119}Sn NMR (149 MHz, $CDCl_3$): δ (ppm) −51.9; IR (neat): $\tilde{\nu}$ (cm^{-1}) 3305, 2955, 2922, 2852, 1654, 1612, 1509, 1484, 758; HRMS (ESI): calcd. for $C_{22}H_{38}BN_2OSn$ $[M+H]^+$: 477.2094, found 477.2094.

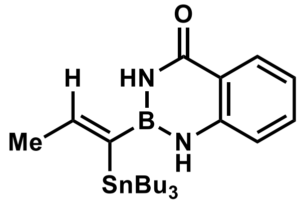

Chemical Formula: $C_{22}H_{37}BN_2OSn$
Molecular Weight: 475,07

(*Z*)-2-(5-Methyl-1-(tributylstannyl)hex-1-en-1-yl)-2,3-dihydrobenzo[*d*][1,3,2]diazaborinin-4(1*H*)-one (7). Prepared analogously as a brownish oil (243.2 mg, 92%, α/β = 97:3, *Z*/*E* > 99:1). ^{1}H NMR (400 MHz, $CDCl_3$): δ (ppm) 8.22-8.15 (m, 1H), 7.48 (ddd, *J* = 8.1, 7.2, 1.6 Hz, 1H), 7.10 (ddd, *J* = 8.1, 7.2, 1.1 Hz, 1H), 6.96 (dd, *J* = 8.1, 1.1 Hz, 1H), 6.94 (brs, 1H), 6.85 (t, *J* = 6.7 Hz, $J_{Sn\text{-}H}$ = 152.5 Hz, 1H), 6.19 (brs, 1H), 2.22-2.09 (m, 2H), 1.68-1.52 (m, 2H), 1.55-1.39 (m, 6H), 1.39-1.18 (m, 8H), 1.01-0.95 (m, 6H), 0.92 (d, *J* = 6.6 Hz, 6H), 0.87 (t, *J* = 7.3 Hz, 9H); ^{13}C NMR (101 MHz, $CDCl_3$): δ (ppm) 166.7, 157.0, 144.6, 137.4 (C(sp^2)-B by HMBC), 133.8, 129.3, 121.5, 118.7, 117.4, 38.7, 37.2, 29.4, 28.2, 27.5, 22.7, 13.8, 11.1; ^{11}B NMR (128 MHz, $CDCl_3$): δ (ppm) 30.7, ^{119}Sn NMR (149 MHz, $CDCl_3$): δ (ppm) −52.2; IR (neat): $\tilde{\nu}$ (cm^{-1}) 3306, 2954, 2923, 2870, 2852, 1654, 1613, 1510, 1485, 1261, 1164, 759; HRMS (ESI): calcd. for $C_{26}H_{45}BN_2OSnNa$ [M+Na]$^+$: 555.2539, found 555.2537.

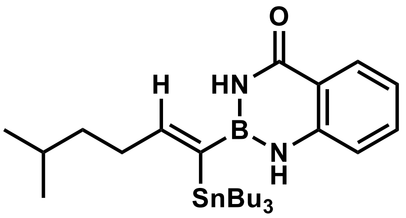

Chemical Formula: $C_{26}H_{45}BN_2OSn$
Molecular Weight: 531.18

(*Z*)-2-(4-Phenyl-1-(tributylstannyl)but-1-en-1-yl)-2,3-dihydrobenzo[*d*][1,3,2]diazaborinin-4(1*H*)-one (9). Prepared analogously as a brownish oil (250.8 mg, 89%, α/β = 91:9, *Z*/*E* > 99:1). ^{1}H NMR (400 MHz, $CDCl_3$): δ (ppm) 8.23-8.15 (m, 1H), 7.50 (ddd, *J* = 8.0, 7.2, 1.6 Hz, 1H), 7.36-7.27 (m, 2H), 7.24-7.17 (m, 3H), 7.11 (ddd, *J* = 8.1, 7.2, 1.1 Hz, 1H), 7.02-6.95 (m, 1H), 6.90 (brs, 1H), 6.89 (t, *J* = 6.7 Hz, $J_{Sn\text{-}H}$ = 149.7 Hz, 1H), 6.15 (brs, 1H), 2.77 (dd, *J* = 9.0, 6.5 Hz, 2H), 2.52-2.44 (m, 2H), 1.52-1.41 (m, 6H), 1.35-1.23 (m, 6H), 1.00-0.93 (m, 6H), 0.86 (t, *J* = 7.3 Hz, 9H); ^{13}C NMR (101 MHz, $CDCl_3$): δ (ppm) 166.7, 155.2, 144.5, 141.4, 139.0 (C(sp^2)-B by HMBC), 133.8, 129.3, 128.6, 128.6, 126.2, 121.6, 118.7, 117.4, 40.9, 35.8, 29.4, 27.5, 13.8, 11.1; ^{11}B NMR (128 MHz, $CDCl_3$): δ (ppm) 30.8, ^{119}Sn NMR (149 MHz, $CDCl_3$): δ (ppm) −52.1; IR (neat): $\tilde{\nu}$ (cm^{-1}) 3306, 2954, 2922, 2869, 2852, 1654, 1612, 1508, 1484, 1151, 759, 696; HRMS (ESI): calcd. for $C_{29}H_{42}BN_2OSnNa$ [M-H]$^-$: 565.2418, found 565.2423

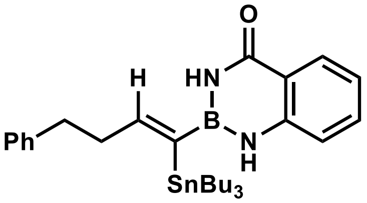

Chemical Formula: $C_{29}H_{43}BN_2OSn$
Molecular Weight: 565,20

(*Z*)-2-(2-Cyclopentyl-1-(tributylstannyl)vinyl)-2,3-dihydrobenzo[*d*][1,3,2]diazaborinin-4(1*H*)-one (11a). Prepared analogously as a brownish oil (221.8 mg, 0.42 mmol, 84%, *α*/*β* = 97:3, *Z*/*E* > 99:1). The isomer s of the crude material were *α*/*β* = 89:11 and *Z*/*E* > 99:1. ^{1}H NMR (400 MHz, $CDCl_3$): δ (ppm) 8.22-8.15 (m, 1H), 7.49 (ddd, *J* = 8.2, 7.2, 1.6 Hz, 1H), 7.15-7.06 (m, 1H), 7.00-6.90 (m, 1H), 6.95 (brs, 1H), 6.72 (d, *J* = 9.3 Hz, $J_{Sn\text{-}H}$ = 151.5 Hz, 1H), 6.18 (brs, 1H), 2.40-2.25 (m, 1H), 1.85-1.74 (m, 2H), 1.74-1.55 (m, 2H), 1.65-1.55 (m, 2H), 1.54-1.44 (m, 6H), 1.44-1.36 (m, 2H), 1.36-1.24 (m, 6H), 1.04-0.91 (m, 6H), 0.87 (t, *J* = 7.3 Hz, 9H); ^{13}C NMR (101 MHz, $CDCl_3$): δ (ppm) ^{13}C NMR (101 MHz, $CDCl_3$) δ 166.7, 161.4, 144.6, 135.0 (C(sp^2)-B by HMBC), 133.8, 129.3, 121.5, 118.7, 117.4, 50.2, 33.7, 29.4, 27.5, 25.9, 13.8, 11.3; ^{11}B NMR (128 MHz, $CDCl_3$): δ (ppm) 30.7, ^{119}Sn NMR (149 MHz, $CDCl_3$): δ (ppm) –50.1; IR (neat): $\tilde{\nu}$ (cm^{-1}) 3306, 2953, 2924, 1654, 1613, 1507, 1485, 1153, 757; HRMS (ESI): calcd. for $C_{26}H_{43}BN_2OSnNa$ [M+Na]$^+$: 553.2383, found 553.2387.

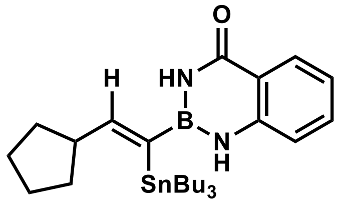

Chemical Formula: $C_{26}H_{43}BN_2OSn$
Molecular Weight: 529,16

(*Z*)-2-(2-Cyclopentyl-1-(tributylstannyl)vinyl)-6-fluoro-2,3-dihydrobenzo[*d*][1,3,2]diazaborinin-4(1*H*)-one (11b). Prepared analogously as a brownish oil (233.5 mg, 85%, *α*/*β* = 99:1, *Z*/*E* > 99:1). The isomer s of the crude product were *α*/*β* = 94:6, *Z*/*E* > 99:1. ^{1}H NMR (400 MHz, $CDCl_3$): δ (ppm) 7.85 (dd, *J* = 9.0, 3.0 Hz, 1H), 7.22 (ddd, *J* = 8.9, 7.9, 3.0 Hz, 1H), 7.01 (brs, 1H), 6.94 (dd, *J* = 8.8, 4.2 Hz, 1H), 6.71 (d, *J* = 9.4 Hz, 1H), 6.18 (brs, 1H), 2.39-2.25 (m, 1H), 1.84-1.74 (m, 2H), 1.74-1.67 (m, 2H), 1.64-1.56 (m, 2H), 1.51-1.44 (m, 6H), 1.43-1.36 (m, 2H), 1.33-1.26 (m, 6H), 0.99-0.92 (m, 6H), 0.86 (t, *J* = 7.3 Hz, 9H); ^{13}C NMR (101 MHz, $CDCl_3$): δ (ppm) 165.9 (d, *J* = 2.5 Hz), 161.6, 157.6 (d, *J* = 240.9 Hz), 141.0 (d, *J* = 2.0 Hz), 134.9 (C(sp^2)-B by HMBC), 121.6 (d, *J* = 24.1 Hz), 119.6 (d, *J* = 7.0 Hz), 118.8 (d, *J* = 7.5 Hz), 114.4 (d, *J* = 23.6 Hz), 50.2, 33.7, 29.4, 27.5, 25.9, 13.8, 11.3; ^{19}F NMR (282 MHz, $CDCl_3$): δ (ppm) –121.5, ^{11}B NMR (128 MHz, $CDCl_3$): δ (ppm) 30.8; ^{119}Sn NMR (149 MHz, $CDCl_3$): δ (ppm) –50.0; IR (neat): $\tilde{\nu}$ (cm^{-1}) 3440, 3406, 3305, 2953, 2923, 2869, 2854, 1651, 1597, 1496, 1334, 825; HRMS (ESI): calcd. for $C_{26}H_{41}BFN_2OSn$ [M-H]$^-$: 547.2323, found 547.2326.

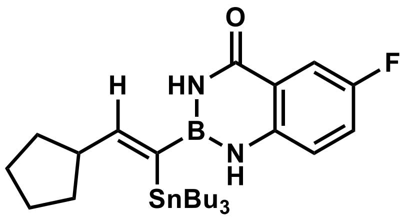

Chemical Formula: $C_{26}H_{42}BFN_2OSn$
Molecular Weight: 547.15

(*Z*)-2-(2-Cyclohexyl-1-(tributylstannyl)vinyl)-2,3-dihydrobenzo[*d*][1,3,2]diazaborinin-4(1*H*)-one (13). Prepared analogously as a brownish oil (203.3 mg, 75%, *α*/*β* = 95:5, *Z*/*E* > 99:1). The isomer s of the crude material were *α*/*β* = 87:13, *Z*/*E* > 99:1. ^{1}H NMR (400 MHz, $CDCl_3$): δ (ppm) 8.18 (dd, *J* = 7.9, 1.6 Hz, 1H), 7.48 (ddd, *J* = 8.1, 7.2, 1.6 Hz, 1H), 7.10 (ddd, *J* = 8.1, 7.2, 1.1 Hz, 1H), 6.96 (dd, *J* = 8.2, 1.0 Hz, 1H), 6.94 (brs, 1H), 6.64 (d, *J* = 9.2 Hz, $J_{Sn\text{-}H}$ = 152.5 Hz, 1H), 6.18 (brs, 1H), 1.94-1.80 (m, 1H), 1.81-1.74 (m, 2H), 1.74-1.59 (m, 4H), 1.54-1.44 (m, 6H), 1.36-1.26 (m, 6H), 1.27-1.13 (m, 4H), 1.02-0.93 (m, 6H), 0.87 (t, *J* = 7.3 Hz, 9H); ^{13}C NMR (101 MHz, $CDCl_3$): δ (ppm) 166.8, 162.1, 144.6, 135.1 (C(sp^2)-B by HMBC), 133.8, 129.3, 121.5, 118.7, 117.4, 48.9, 32.9, 29.4, 27.5, 25.9, 25.8, 13.8, 11.3; ^{11}B NMR (128 MHz, $CDCl_3$): δ (ppm) 30.8, ^{119}Sn NMR (149 MHz, $CDCl_3$): δ (ppm) –51.8; IR (neat): $\tilde{\nu}$ (cm^{-1}) 3306, 2854, 2921, 2869, 2849, 1653, 1612, 1508, 1485, 759, 736; HRMS (ESI): calcd. for $C_{27}H_{45}BN_2O$-SnNa $[M+Na]^+$: 567.2539, found 567.2537.

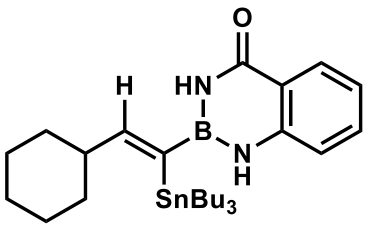

Chemical Formula: $C_{27}H_{45}BN_2OSn$
Molecular Weight: 543.19

(*Z*)-2-(5-Chloro-1-(tributylstannyl)pent-1-en-1-yl)-2,3-dihydrobenzo[*d*][1,3,2]diazaborinin-4(1*H*)-one (15). Prepared analogously as a brownish oil (248.9 mg, 93%, *α*/*β* = 92:8, *Z*/*E* > 99:1). ^{1}H NMR (400 MHz, $CDCl_3$): δ (ppm) 8.19 (dd, *J* = 8.0, 1.6 Hz, 1H), 7.50 (ddd, *J* = 8.1, 7.2, 1.6 Hz, 1H), 7.11 (ddd, *J* = 8.2, 7.2, 1.1 Hz, 1H), 7.01-6.94 (m, 1H), 6.93 (brs, 1H), 6.82 (t, *J* = 6.7 Hz, $J_{Sn\text{-}H}$ = 147.8 Hz, 1H), 6.17 (brs, 1H), 3.59 (t, *J* = 6.5 Hz, 2H), 2.39-2.26 (m, 2H), 1.99-1.90 (m, 2H), 1.57-1.40 (m, 6H), 1.36-1.24 (m, 6H), 1.05-0.96 (m, 6H), 0.87 (t, *J* = 7.3 Hz, 9H); ^{13}C NMR (101 MHz, $CDCl_3$): δ (ppm) 166.7, 154.0, 144.5, 140.1 (C(sp^2)-B by HMBC), 133.9, 129.3, 121.6, 118.7, 117.4, 44.6, 36.2, 32.4, 29.4, 27.5, 13.8, 11.1; ^{11}B NMR (128 MHz, $CDCl_3$): δ (ppm) 30.7, ^{119}Sn NMR (149 MHz, $CDCl_3$): δ (ppm) –51.5; IR (neat): $\tilde{\nu}$ (cm^{-1}) 3304, 2955, 2923, 2870, 2852, 1656, 1612, 1507, 1485, 756; HRMS (ESI): calcd. for $C_{24}H_{40}BClN_2OSnNa$ $[M+Na]^+$: 561.1836, found 561.1837.

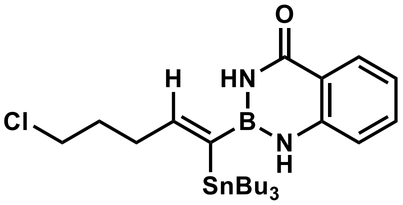

Chemical Formula: $C_{24}H_{40}BClN_2OSn$
Molecular Weight: 537,57

(*Z*)-2-(4-(*tert*-Butyldimethylsiloxy)-1-(tributylstannyl)but-1-en-1-yl)-2,3-dihydro-benzo[*d*][1,3,2]diazaborinin-4(1*H*)-one (17).

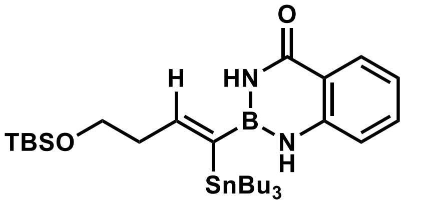

Chemical Formula: $C_{29}H_{53}BN_2O_2SiSn$
Molecular Weight: 619,36

Prepared analogously as a brownish oil (2.47 g, 80%, *α*/*β* > 99:1, *Z*/*E* > 99:1). ^{1}H NMR (400 MHz, $CDCl_3$): δ (ppm) 8.23-8.15 (m, 1H), 7.49 (ddd, *J* = 8.1, 7.2, 1.6 Hz, 1H), 7.11 (ddd, *J* = 8.1, 7.2, 1.1 Hz, 1H), 6.99-6.92 (m, 1H), 6.88 (brs, 1H), 6.87 (t, *J* = 6.6 Hz, $J_{Sn\text{-}H}$ = 150.5 Hz, 1H), 6.15 (brs, 1H), 3.72 (t, *J* = 6.7 Hz, 2H), 2.40 (q, *J* = 6.7 Hz, 2H), 1.54-1.42 (m, 6H), 1.37-1.22 (m, 6H), 1.04-0.93 (m, 6H), 0.91 (s, 9H), 0.87 (t, *J* = 7.3 Hz, 9H), 0.07 (s, 6H); ^{13}C NMR (101 MHz, $CDCl_3$): δ (ppm) 166.6, 152.8, 144.6, 140.5 (C(sp^2)-B by HMBC), 133.8, 129.3, 121.5, 118.7, 117.4, 62.7, 42.3, 29.4, 27.5, 26.1, 18.5, 13.8, 11.1, –5.1; ^{11}B NMR (128 MHz, $CDCl_3$): δ (ppm) 30.7, ^{119}Sn NMR (149 MHz, $CDCl_3$): δ (ppm) –52.4; IR (neat): $\tilde{\nu}$ (cm^{-1}) 3307, 2954, 2926, 2855, 1657, 1613, 1508, 1485, 1256, 1091, 835, 759; HRMS (ESI): calcd. for $C_{29}H_{53}BN_2O_2SiSnNa$ [M+Na]$^+$: 643.2884, found 643.2895.

(*Z*)-2-(2-Phenyl-1-(tributylstannyl)vinyl)-2,3-dihydrobenzo[*d*][1,3,2]diazaborinin-4(1*H*)-one (19).

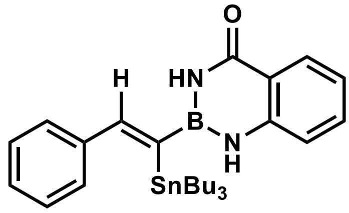

Chemical Formula: $C_{27}H_{39}BN_2OSn$
Molecular Weight: 537,14

Prepared analogously as a brownish oil (263.6 mg, 98%, *α*/*β* > 99:1, *Z*/*E* > 99:1). ^{1}H NMR (400 MHz, $CDCl_3$): δ (ppm) 8.23 (dd, *J* = 7.9, 1.5 Hz, 1H), 7.96 (s, $J_{Sn\text{-}H}$ = 145.4 Hz, 1H), 7.52 (ddd, *J* = 8.1, 7.2, 1.6 Hz, 1H), 7.34 (tt, *J* = 6.9, 6.0 Hz, 3H), 7.29-7.24 (m, 2H), 7.14 (ddd, *J* = 8.1, 7.3, 1.0 Hz, 1H), 7.04 (brs, 1H), 7.02 (d, *J* = 8.1 Hz, 1H), 6.31 (brs, 1H), 1.39-1.31 (m, 6H), 1.27-1.13 (m, 6H), 0.80 (t, *J* = 7.3 Hz, 9H), 0.81-0.76 (m, 6H); ^{13}C NMR (101 MHz, $CDCl_3$): δ (ppm) 166.7, 153.4, 144.5, 143.7 (C(sp^2)-B by HMBC), 142.3, 133.9, 129.3, 128.4, 128.1, 127.4, 121.7, 118.8, 117.5, 29.3, 27.4, 13.7, 11.7; ^{11}B NMR (128 MHz, $CDCl_3$): δ (ppm) 31.2; ^{119}Sn NMR (149 MHz, $CDCl_3$): δ (ppm) –48.1; IR (neat): $\tilde{\nu}$ (cm^{-1}) 3304, 2954, 2921, 2852, 1655, 1509, 1485, 1153, 758, 695; HRMS (ESI): calcd. for $C_{27}H_{39}BN_2OSnNa$ [M+Na]$^+$: 561.2070, found 561.2076.

(*Z*)-2-(2-Cyclopropyl-1-(tributylstannyl)vinyl)-2,3-dihydrobenzo[*d*][1,3,2]diazaborinin-4(1*H*)-one (21). Prepared analogously as a brownish oil (210.2 mg, 0.42 mmol, 84%, *α*/*β* > 99:1, *Z*/*E* > 99:1). ^{1}H NMR (400 MHz, $CDCl_3$): δ (ppm) 8.18 (dd, *J* = 7.9, 1.7 Hz, 1H), 7.48 (ddd, *J* = 8.1, 7.2, 1.6 Hz, 1H), 7.09 (ddd, *J* = 8.1, 7.2, 1.1 Hz, 1H), 6.95 (dd, *J* = 8.2, 1.1 Hz, 1H), 6.92 (brs, 1H), 6.16 (brs, 1H), 6.15 (d, *J* = 9.4 Hz, J_{Sn-H} = 146.7 Hz, 1H), 1.58-1.46 (m, 6H), 1.37-1.26 (m, 7H), 1.07-0.98 (m, 6H), 0.91-0.85 (m, 2H), 0.87 (t, *J* = 7.3 Hz, 9H), 0.62-0.54 (m, 2H); ^{13}C NMR (101 MHz, $CDCl_3$): δ (ppm) 166.8, 160.7, 144.7, 133.8, 133.3 (C(sp^2)-B by HMBC), 129.3, 121.4, 118.7, 117.4, 29.4, 27.5, 19.5, 13.8, 11.2, 8.4; ^{11}B NMR (128 MHz, $CDCl_3$): δ (ppm) 30.7, ^{119}Sn NMR (149 MHz, $CDCl_3$): δ (ppm) –46.2; IR (neat): $\tilde{\nu}$ (cm^{-1}) 3307, 2955, 2922, 2851, 1655, 1613, 1508, 1485, 1153, 757; HRMS (ESI): calcd. for $C_{24}H_{40}BN_2OSn$ [M+H]$^+$: 503.2250, found 503.2252.

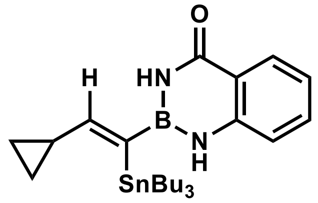

Chemical Formula: $C_{24}H_{39}BN_2OSn$
Molecular Weight: 501,11

(*Z*)-2-(3-Methoxy-1-(tributylstannyl)prop-1-en-1-yl)-2,3-dihydrobenzo[*d*][1,3,2]diazaborinin-4(1*H*)-one (23). Prepared analogously as a brownish oil (203.3 mg, 81%, *α*/*β* = 98:2, *Z*/*E* > 99:1). ^{1}H NMR (400 MHz, $CDCl_3$): δ (ppm) 8.19 (ddd, *J* = 8.0, 1.5, 0.7 Hz, 1H), 7.49 (ddd, *J* = 8.1, 7.2, 1.6 Hz, 1H), 7.11 (ddd, *J* = 8.1, 7.2, 1.1 Hz, 1H), 6.98-6.95 (m, 1H), 6.92 (t, *J* = 4.9 Hz, J_{Sn-H} = 146.4 Hz, 1H), 6.90 (brs, 1H), 6.21 (brs, 1H), 3.99 (d, *J* = 4.9 Hz, 2H), 3.38 (s, 3H), 1.53-1.41 (m, 6H), 1.34-1.22 (m, 6H), 0.99-0.92 (m, 6H), 0.86 (t, *J* = 7.3 Hz, 9H); ^{13}C NMR (101 MHz, $CDCl_3$): δ (ppm) 166.6, 150.3, 144.5, 141.3 (C(sp^2)-B by HMBC), 133.9, 129.3, 121.6, 118.7, 117.4, 76.1, 58.5, 29.4, 27.5, 13.8, 11.6; ^{11}B NMR (128 MHz, $CDCl_3$): δ (ppm) 31.3, ^{119}Sn NMR (149 MHz, $CDCl_3$): δ (ppm) –52.2; IR (neat): $\tilde{\nu}$ (cm^{-1}) 3307, 2954, 2921, 2870, 2852, 1655, 1612, 1511, 1484, 1162, 1117, 759; HRMS (ESI): calcd. for $C_{23}H_{39}BN_2O_2SnNa$ [M+Na]$^+$: 529.2019, found 529.2019.

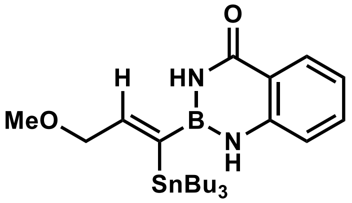

Chemical Formula: $C_{23}H_{39}BN_2O_2Sn$
Molecular Weight: 505,10

(*Z*)-2-(3,3-Diethoxy-1-(tributylstannyl)prop-1-en-1-yl)-2,3-dihydrobenzo[*d*][1,3,2]diazaborinin-4(1*H*)-one (25). Prepared analogously as a brownish oil (232.1 mg, 0.41 mmol, 82%, *α*/*β* > 99:1, *Z*/*E* > 99:1). ^{1}H NMR (400 MHz, $CDCl_3$): δ (ppm) 8.19 (ddd, *J* = 8.0, 1.6, 0.7 Hz, 1H), 7.50 (ddd, *J* = 8.1, 7.2, 1.6 Hz, 1H), 7.12 (ddd, *J* = 8.1, 7.2, 1.0 Hz, 1H), 7.00-6.93 (m, 1H), 6.89 (brs, 1H), 6.79 (d, *J* = 5.4 Hz, 1H), 6.22 (brs, 1H), 4.81 (d, *J* = 5.4 Hz, $J_{Sn\text{-}H}$ = 143.0 Hz, 1H), 3.68 (dq, *J* = 9.4, 7.1 Hz, 2H), 3.54 (dq, *J* = 9.4, 7.0 Hz, 2H), 1.53-1.41 (m, 6H), 1.35-1.21 (m, 12H), 1.11-0.91 (m, 6H), 0.85 (t, *J* = 7.3 Hz, 9H); ^{13}C NMR (101 MHz, $CDCl_3$): δ (ppm) 166.5, 150.4, 144.4, 144.4 (C(sp^2)-B by HMBC), 133.9, 129.3, 121.7, 118.7, 117.4, 102.9, 61.0, 29.4, 27.5, 15.5, 13.8, 11.6; ^{11}B NMR (128 MHz, $CDCl_3$): δ (ppm) 31.4, ^{119}Sn NMR (149 MHz, $CDCl_3$): δ (ppm) –52.7; IR (neat): $\tilde{\nu}$ (cm^{-1}) 3307, 2955, 2922, 2871, 2853, 1658, 1613, 1510, 1485, 1117, 1047, 760; HRMS (ESI): calcd. for $C_{26}H_{45}BN_2O_3SnNa$ [M+Na]$^+$: 587.2437, found 587.2438.

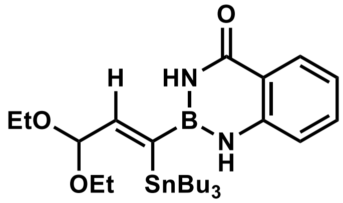

Chemical Formula: $C_{26}H_{45}BN_2O_3Sn$
Molecular Weight: 563,18

(*Z*)-2-(1-(Tributylstannyl)-3-(trimethylsilyl)prop-1-en-1-yl)-2,3-dihydrobenzo[*d*][1,3,2]-diazaborinin-4(1*H*)-one (27a). Prepared analogously as a brownish oil (222.2 mg, 81%, *α-Z*/*β-Z*/*β-E* = 79:8:13). The isomer of the crude material was *α-Z*/*β-Z*/*β-E* = 75:10:15. *Data of the major α-Z-isomer*: ^{1}H NMR (400 MHz, $CDCl_3$): δ (ppm) 8.18 (dd, *J* = 7.9, 1.6 Hz, 1H), 7.48 (ddd, *J* = 8.1, 7.2, 1.5 Hz, 1H), 7.09 (ddd, *J* = 8.1, 7.2, 1.1 Hz, 1H), 7.00-6.90 (m, 3H), 6.16 (brs, 1H), 1.80-1.72 (m, 2H), 1.57-1.44 (m, 6H), 1.36-1.26 (m, 6H), 1.03-0.94 (m, 6H), 0.87 (t, *J* = 7.3 Hz, 9H), 0.07 (s, 9H); ^{13}C NMR (101 MHz, $CDCl_3$): δ (ppm) 166.8, 153.5, 144.7, 135.1 (C(sp^2)-B by HMBC), 133.8, 129.3, 121.4, 118.7, 117.4, 31.4, 29.4, 27.6, 13.8, 11.2, –1.4; ^{11}B NMR (128 MHz, $CDCl_3$): δ (ppm) 30.5, ^{119}Sn NMR (149 MHz, $CDCl_3$): δ (ppm) –53.4; IR (neat): $\tilde{\nu}$ (cm^{-1}) 3306, 2954, 2923, 2853, 1654, 1614, 1509, 1485, 1247, 838, 759, 692; HRMS (ESI): calcd. for $C_{25}H_{44}BN_2OSiSn$ [M-H]$^-$: 547.2343, found 547.2347.

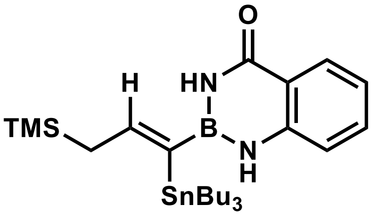

Chemical Formula: $C_{25}H_{45}BN_2OSiSn$
Molecular Weight: 547.25

(*Z*)-6-Fluoro-2-[1-(tributylstannyl)-3-(trimethylsilyl)prop-1-en-1-yl]-2,3-dihydro-benzo[d][1,3,2]diazaborinin-4(1*H*)-one (27b). Prepared analogously as a brownish oil (210 mg, 74%, *α-Z*/*β-Z*/*β-E* = 82:7:11). *Spectral data of the major α-Z-isomer:* ^{1}H NMR (400 MHz, $CDCl_3$): δ (ppm) 7.85 (dd, *J* = 9.0, 3.0 Hz, 1H), 7.22 (ddd, *J* = 8.8, 7.8, 3.0 Hz, 1H), 6.98–6.88 (m, 3H), 6.11 (br s, 1H), 1.76 (d, *J* = 8.0 Hz, 2H), 1.53–1.44 (m, 6H), 1.38–1.29 (m, 6H), 1.01–0.94 (m, 6H), 0.87 (t, *J* = 7.3 Hz, 9H), 0.07 (s, 9H); ^{13}C NMR (101 MHz, $CDCl_3$): δ (ppm) 166.0, 157.6 (d, *J* = 240.5 Hz), 153.7, 141.0, 134.8 (C(sp^2)-B by HMBC), 121.6 (d, *J* = 24.4 Hz), 119.6 (d, *J* = 7.4 Hz), 118.7 (d, *J* = 7.4 Hz), 114.5 (d, *J* = 23.5 Hz), 31.5, 29.4, 27.6, 13.8, 11.2, –1.3; ^{19}F NMR (282 MHz, $CDCl_3$): δ (ppm) –121.6; ^{11}B NMR (128 MHz, $CDCl_3$): δ (ppm) 30.2; ^{119}Sn NMR (149 MHz, $CDCl_3$): δ (ppm) –53.3; IR (neat): $\tilde{\nu}$ (cm^{-1}) 3308 (br), 2955, 2923, 2871, 2825, 1655, 1499, 1336, 1249, 1137, 848, 734; HRMS (ESI): calcd. for $C_{25}H_{45}BFN_2OSiSn$ [M+H]$^+$: 567.2394; found: 567.2392.

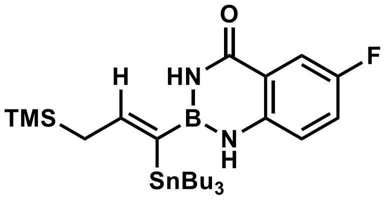

Chemical Formula: $C_{25}H_{44}BFN_2OSiSn$
Molecular Weight: 565.24

***tert*-Butyl (*Z*)-2-(3-(4-oxo-3,4-dihydrobenzo[*d*][1,3,2]diazaborinin-2(1*H*)-yl)-3-(tributylstannyl)allyl)piperidine-1-carboxylate and *tert*-butyl (*E*)-2-(3-(4-oxo-3,4-dihydrobenzo[*d*][1,3,2]diazaborinin-2(1*H*)-yl)-2-(tributylstannyl)allyl)piperidine-1-carboxylate (29).** Prepared analogously as a brownish oil (249.1 mg, 76%, *α*/*β* = 85:15, *Z*/*E* > 99:1); the corresponding *β-E* isomer was separated by flash chromatography and obtained as a brownish oil (48.2 mg, 15%, *α*/*β* = 1:99, *Z*/*E* = 1:99). The isomer in the crude product was *α-Z*/*β-Z*/*β-E* = 70:12:18. *Spectroscopic data of the major α-Z-isomer*: ^{1}H NMR (400 MHz, $CDCl_3$): δ (ppm) 8.17 (dd, *J* = 8.0, 1.7 Hz, 1H), 7.47 (ddd, *J* = 8.6, 7.2, 1.6 Hz, 1H), 7.09 (ddd, *J* = 8.2, 7.2, 1.1 Hz, 1H), 6.95 (dd, *J* = 8.2, 1.2 Hz, 1H), 6.89 (brs, 1H), 6.81 (t, *J* = 6.4 Hz, $J_{Sn\text{-}H}$ = 152.2 Hz, 1H), 6.27 (brs, 1H), 4.51-4.38 (m, 1H), 4.09-3.94 (m, 1H), 2.84-2.70 (m, 1H), 2.62 (ddd, *J* = 14.7, 9.0, 5.8 Hz, 1H), 2.23 (dt, *J* = 14.8, 6.6 Hz, 1H), 1.67-1.38 (m, 12H), 1.44 (s, 9H), 1.37-1.24 (m, 6H), 1.06-0.93 (m, 6H), 0.87 (t, *J* = 7.3 Hz, 9H); ^{13}C NMR (101 MHz, $CDCl_3$): δ (ppm) 166.6, 155.2, 153.5, 144.6, 139.6 (C(sp^2)-B by HMBC), 133.8, 129.2, 121.4, 118.7, 117.4, 79.6, 50.6, 39.2, 39.0, 29.4, 28.7, 28.6, 27.5, 25.6, 19.3, 13.8, 11.1; ^{11}B NMR (128 MHz, $CDCl_3$): δ (ppm) 30.7, ^{119}Sn NMR (149 MHz, $CDCl_3$): δ (ppm) –52.2; IR (neat): $\tilde{\nu}$ (cm^{-1}) 3274, 2954, 1677, 1648, 1613, 1516, 1485, 1409, 1356,

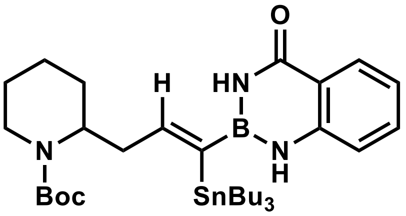

Chemical Formula: $C_{32}H_{54}BN_3O_3Sn$
Molecular Weight: 658.32

1262, 1159, 759; HRMS (ESI): calcd. for $C_{32}H_{54}BN_3O_3SnNa$ [M+Na]$^+$: 682.3172, found 682.3177.

Spectroscopic data of the β-E isomer : ^{1}H NMR (400 MHz, $CDCl_3$): δ (ppm) 8.18 (dd, *J* = 7.9, 1.4 Hz, 1H), 7.50 (ddd, *J* = 8.6, 7.0, 1.6 Hz, 1H), 7.44 (brs, 1H), 7.14-7.08 (m, 2H), 6.03 (s, 1H), 4.36-4.25 (m, 1H), 3.97-3.87 (m, 1H), 2.83-2.64 (m, 3H), 1.51 (s, 9H), 1.59-1.45 (m, 12H), 1.37-1.29 (m, 6H), 1.00-0.94 (m, 6H), 0.91 (t, *J* = 7.3 Hz, 9H); ^{13}C NMR (101 MHz, $CDCl_3$): δ (ppm) 168.3 (C(sp^2)-Sn by HMBC), 167.0, 155.7, 145.4, 140.0, 133.6, 128.8, 121.5, 118.9, 118.4, 80.0, 51.8, 40.2, 38.6, 29.2, 28.7, 27.6, 25.5, 19.4, 17.7, 13.8, 10.4; ^{11}B NMR (128 MHz, $CDCl_3$): δ (ppm) 26.6, ^{119}Sn NMR (149 MHz, $CDCl_3$): δ (ppm) –38.2; IR (neat): $\tilde{\nu}$ (cm^{-1}) 3273, 2957, 1677, 1650, 1613, 1518, 1485, 1356, 1262, 1159, 759, 647; HRMS (ESI): calcd. for $C_{32}H_{54}BN_3O_3SnNa$ [M+Na]$^+$: 682.3172, found 682.3174.

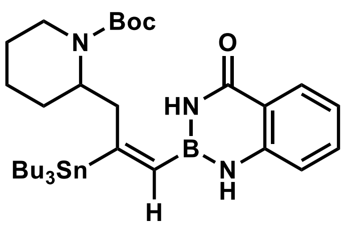

Chemical Formula: $C_{32}H_{54}BN_3O_3Sn$
Molecular Weight: 658,32

(*R*,*Z*)-2-(5-(*tert*-Butyldiphenylsiloxy)-1-(tributylstannyl)-4-((triethylsiloxy)pent-1-en-1-yl)-2,3-dihydrobenzo[*d*][1,3,2]diazaborinin-4(1*H*)-one (31). Prepared analogously as a brownish oil (404.9 mg, 91%, *α*/*β* = 98:2, *Z*/*E* > 99:1). $[\alpha]_D^{20}$ = +11.4° (c = 1.48, $CHCl_3$). ^{1}H NMR (400 MHz, $CDCl_3$): δ (ppm) 8.21 (dd, *J* = 8.0, 1.6 Hz, 1H), 7.71-7.64 (m, 4H), 7.50 (ddd, *J* = 8.1, 7.2, 1.6 Hz, 1H), 7.43-7.33 (m, 6H), 7.12 (ddd, *J* = 8.1, 7.2, 1.1 Hz, 1H), 6.93 (dd, *J* = 8.2, 1.0 Hz, 1H), 6.91 (t, *J* = 6.4 Hz, $J_{Sn\text{-}H}$ = 151.6 Hz, 1H), 6.84 (brs, 1H), 6.10 (brs, 1H), 3.88 (tt, *J* = 7.1, 4.7 Hz, 1H), 3.62 (dd, *J* = 10.1, 5.0 Hz, 1H), 3.49 (dd, *J* = 10.1, 6.6 Hz, 1H), 2.59 (ddd, *J* = 14.9, 6.3, 4.6 Hz, 1H), 2.34 (dt, *J* = 14.4, 7.0 Hz, 1H), 1.53-1.41 (m, 6H), 1.35-1.21 (m, 6H), 1.06 (s, 9H), 1.04-0.96 (m, 6H), 0.90 (t, *J* = 8.0 Hz, 9H), 0.85 (t, *J* = 7.3 Hz, 9H), 0.53 (q, *J* = 7.9 Hz, 6H); ^{13}C NMR (101 MHz, $CDCl_3$): δ (ppm) 166.6, 153.2, 144.6, 140.1 (C(sp^2)-B by HMBC), 135.8, 135.7, 133.8, 133.6, 133.5, 129.9, 129.3, 127.8, 121.5, 118.7, 117.4, 72.7, 68.3, 43.8, 29.4, 27.5, 27.0, 19.3, 13.8, 11.1, 7.0, 5.1; ^{11}B NMR (128 MHz, $CDCl_3$): δ (ppm) 30.6, ^{119}Sn NMR (149 MHz, $CDCl_3$): δ (ppm) –52.9; IR (neat): $\tilde{\nu}$ (cm^{-1}) 3306, 2954, 2928, 2872, 2857, 1665, 1613, 1506, 1486, 1112, 737, 700, 503; HRMS (ESI): calcd. for $C_{46}H_{74}BN_2O_3Si_2Sn$ [M+H]$^+$: 889.4348, found 889.4344.

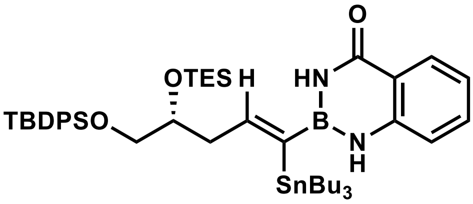

$C_{46}H_{73}BN_2O_3Si_2Sn$
Molecular Weight: 887.79

(*Z*)-2-(3-(2,3,4,6-Zetra-*O*-(*tert*-butyldimethylsilyl)-*α*-*D*-glucopyranosyl)-1-(tributyl-stannyl)prop-1-en-1-yl)-2,3-dihydrobenzo[*d*][1,3,2]diazaborinin-4(1*H*)-one (33). Prepared analogously as a brownish oil (245.1 mg, 90%, *α*/*β* > 99:1, *Z*/*E* > 99:1). $[\alpha]_D^{20}$ = +28.4° (c = 0.92, $CHCl_3$). ^1H NMR (400 MHz, $CDCl_3$): δ (ppm) 8.19 (dd, *J* = 7.9, 1.6 Hz, 1H), 7.48 (ddd, *J* = 8.1, 7.2, 1.6 Hz, 1H), 7.10 (ddd, *J* = 8.2, 7.3, 1.1 Hz, 1H), 7.08 (dd, *J* = 7.8, 5.3 Hz $J_{Sn\text{-}H}$ = 153.5 Hz, 1H), 6.94 (dd, *J* = 8.1, 1.0 Hz, 1H), 6.84 (brs, 1H), 6.20 (brs, 1H), 3.88-3.81 (m, 3H), 3.79-3.75 (m, 2H), 3.73 (dt, *J* = 4.7, 1.2 Hz, 1H), 3.53-3.49 (m, 1H), 2.68 (ddd, *J* = 14.6, 10.3, 5.4 Hz, 1H), 2.04-1.92 (m, 1H), 1.53-1.42 (m, 6H), 1.34-1.23 (m, 6H), 1.01-0.96 (m, 6H), 0.94 (s, 9H), 0.90 (s, 9H), 0.89 (s, 9H), 0.86 (t, *J* = 7.3 Hz, 9H), 0.83 (s, 9H), 0.13 (s, 3H), 0.12 (s, 3H), 0.09 (s, 9H), 0.08 (s, 3H), 0.02 (s, 3H), 0.00 (s, 3H); ^{13}C NMR (101 MHz, $CDCl_3$): δ (ppm) 166.6, 154.3, 144.7, 139.2 (C(sp^2)-B by HMBC), 133.7, 129.3, 121.4, 118.7, 117.4, 77.6, 74.8, 72.4, 71.0, 70.1, 62.4, 40.8, 29.4, 27.5, 26.3, 26.2, 26.0, 25.9, 18.5, 18.4, 18.3, 18.0, 13.8, 11.1, –3.4, –3.9, –4.0, –4.5, –4.6, –4.8, –5.1; ^{11}B NMR (128 MHz, $CDCl_3$): δ (ppm) 30.7, ^{119}Sn NMR (149 MHz, $CDCl_3$): δ (ppm) –54.0; IR (neat): $\tilde{\nu}$ (cm^{-1}) 2954, 2928, 2856, 1667, 1252, 1084, 831, 773, 666; HRMS (ESI): calcd. for $C_{52}H_{104}BN_2O_6Si_4Sn$ $[M+H]^+$: 1095.6081, found 1095.6092.

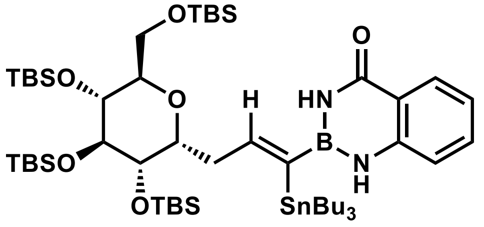

Chemical Formula: $C_{52}H_{103}BN_2O_6Si_4Sn$
Molecular Weight: 1094.26

Copper Mediated Methylation. (*Z*)-2-(Hept-2-en-2-yl)-2,3-dihydrobenzo[*d*][1,3,2]dia-zaborinin-4(1*H*)-one (46).[10] A 25 mL flame-dried Schlenk tube was charged under Ar with compound **1e** (226.1 mg, 1.0 mmol), $[Cp^*RuCl]_4$ (27.2 mg, 25 µmol, 2.5 mol%) and CH_2Cl_2 (5 mL). Bu_3SnH (0.14 mL, 0.525 mmol) was added at a rate of one drop every 5 seconds at 0°C. Once the addition was complete, the mixture was stirred for 30 min before the solvent was evaporated on a Schlenk line.

In a second Schlenk tube, $(Ph_2PO_2)NBu_4$ (250 mg, 544 µmol) was flame dried and, after cooling to room temperature, dissolved in DMSO (5 mL). This solution was added to the crude hydro-stannation product. MeI (0.19 mL, 3 mmol) was then added, immediately followed – within maximum 30 sec – by CuTC (200 mg, 1.05 mmol). The resulting black suspension was stirred for 1 h before the reaction was quenched by the addition of Et_3N (ca. 0.1 mL). The mixture was diluted with ethyl acetate (30 mL) and poured into a mixture of 25% aq. NH_4OH/sat. aq. NH_4Cl solution (1:9, 20 mL). The phases were separated and the clear, bright blue aqueous phase was

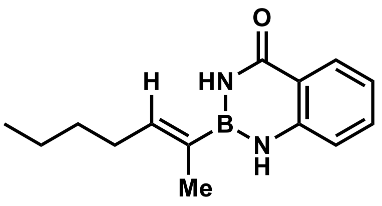

Chemical Formula: $C_{14}H_{19}BN_2O$
Molecular Weight: 242.13

extracted with ethyl acetate (2 x 30 mL). The combined extracts were dried over $MgSO_4$, filtered and concentrated under reduced pressure. Purification of the residue by flash chromatography (CH_2Cl_2/EtOAc, 90:10) afforded the title compound contaminated by some isomers as well as the protodestannylated compound. Pure material was obtained by recrystallization from hexane/ethyl acetate as a colorless solid (149.5 mg, 62%, α/β > 99:1, Z/E > 99:1). ^{1}H NMR (400 MHz, $CDCl_3$): δ (ppm) 8.19 (dd, J = 7.9, 1.7 Hz, 1H), 7.50 (ddd, J = 8.1, 7.1, 1.5 Hz, 1H), 7.28 (brs, 1H), 7.11 (ddd, J = 8.1, 7.2, 1.1 Hz, 1H), 7.06-6.99 (m, 1H), 6.47 (brs, 1H), 6.17 (tq, J = 6.8, 1.6 Hz, 1H), 2.26-2.17 (m, 2H), 1.82-1.80 (m, 3H), 1.48-1.30 (m, 4H), 0.93 (t, J = 7.2 Hz, 3H); ^{13}C NMR (101 MHz, $CDCl_3$): δ (ppm) 166.8, 144.5, 143.4, 133.9, 129.2, 128.7 (C(sp^2)-B by HMBC), 121.6, 118.9, 117.5, 31.3, 28.6, 22.6, 14.1, 14.0; ^{11}B NMR (128 MHz, $CDCl_3$): δ (ppm) 29.0; IR (neat): $\tilde{\nu}$ (cm^{-1}) 3368, 3332, 3249, 2949, 2924, 2867, 1626, 1611, 1486, 1268, 758, 478; HRMS (ESI): calcd. for $C_{14}H_{20}BN_2O$ [M+H]$^+$: 243.1663, found 243.1666.

Suzuki-Miyaura Coupling Reactions under Preservation of the Adjacent Organotin Moiety.[11] A 10 mL flame-dried Schlenk tube was charged under argon atmosphere with the alkenyl-B(aam) substrate (0.1 mmol) and THF (2 mL). A solution of NaOH (3 M, 0.17 mL, 0.5 mmol) was added and the mixture was stirred at room temperature for 10 min. The corresponding aryl halide (1 equiv) and Pd(P^{t}Bu$_3$)$_2$ (1.0 mg, 2.0 μmol, 2 mol%) were then added and the mixture was stirred at 60 °C for 4 h. The reaction was cooled to room temperature and the mixture diluted with water and *tert*-butyl methyl ether. The layers were separated and the aqueous phase was extracted two times with *tert*-butyl methyl ether. The combined organic layers were dried over $MgSO_4$, filtrated, and concentrated under reduced pressure. The crude material was purified by flash chromatography (hexane/EtOAc, 95:5) to yield the desired product.

***(Z)*-*N,N*-Dimethyl-4-[1-(tributylstannyl)hex-1-en-1-yl]aniline (38).** Prepared analogously, using 4-iodo-*N,N*-dimethylaniline as the coupling partner; brown oil (33 mg, 91%). ^{1}H NMR (400 MHz, $CDCl_3$): δ (ppm) 6.93 (m, app. br d, J = 8.7 Hz, 2H), 6.67 (m, app. br d, J = 8.7 Hz, 2H), 6.19 (t, J = 7.2 Hz, $J_{Sn\text{-}H}$ = 127.6 Hz, 1H), 2.92 (s, 6H), 2.18–2.09 (m, 2H), 1.50–1.40 (m, 10H), 1.34–1.24 (m, 6H), 0.95–0.89 (m, 9H), 0.86 (t, J = 7.3 Hz, 9H); ^{13}C NMR (101 MHz, $CDCl_3$): δ (ppm) 148.9,

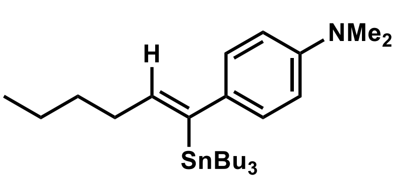

Chemical Formula: $C_{26}H_{47}NSn$
Molecular Weight: 492.38

144.3, 143.0, 136.7, 127.8, 112.9, 41.1, 35.1, 32.6, 29.3, 27.5, 22.7, 14.3, 13.8, 11.2; ^{119}Sn NMR (149 MHz, $CDCl_3$): δ (ppm) –51.5; IR (neat): $\tilde{\nu}$ (cm^{-1}) 2955, 2926, 2871, 2853, 1608, 1514, 1463, 1343, 1164, 1061, 866, 813; HRMS (ESI): calcd. for $C_{26}H_{48}NSn$ $[M+H]^+$: 494.2803, found 494.2805.

***(Z)*-Tributyl[1-(4-methoxyphenyl)hex-1-en-1-yl]stannane (39).** Prepared analogously, using 4-iodoanisole as the coupling partner; brown oil (35 mg, 73%). ^{1}H NMR (400 MHz, $CDCl_3$): δ (ppm) 6.95 (m, app. br d, J = 8.7 Hz, 2H), 6.80 (m, app. br d, J = 8.7 Hz, 2H), 6.19 (t, J = 7.2 Hz, $J_{Sn\text{-}H}$ = 124.6 Hz, 1H), 3.79 (s, 3H), 2.20–2.09 (m, 2H), 1.50–1.34 (m, 10H), 1.33–1.22 (m, 6H), 0.96–0.89 (m, 9H), 0.86 (t, J = 7.3 Hz, 9H); ^{13}C NMR (101 MHz, $CDCl_3$): δ (ppm) 157.6, 144.3, 144.0, 140.6, 128.0, 113.5, 55.4, 35.1, 32.5, 29.3, 27.5, 22.7, 14.2, 13.8, 11.2; ^{119}Sn NMR (149 MHz, $CDCl_3$): δ (ppm) –51.0; IR (neat): $\tilde{\nu}$ (cm^{-1}) 2955, 2926, 2871, 2854, 1604, 1505, 1463, 1283, 1243, 1173, 1040, 826; HRMS (CI): calcd. for $C_{25}H_{45}OSn$ $[M+H]^+$: 481.2487, found 481.2483.

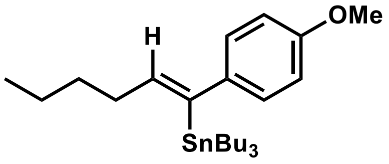

Chemical Formula: $C_{25}H_{44}OSn$
Molecular Weight: 479.34

***(Z)*-Tributyl[1-(4-fluorophenyl)hex-1-en-1-yl]stannane (40).** Prepared analogously, using 4-fluorobromobenzene as the coupling partner; colorless oil (31 mg, 66%). ^{1}H NMR (400 MHz, $CDCl_3$): δ (ppm) 6.97–6.91 (m, 4H), 6.19 (t, J = 7.2 Hz, $J_{Sn\text{-}H}$ = 116.8 Hz, 1H), 2.19–2.09 (m, 2H), 1.49–1.37 (m, 10H), 1.32–1.23 (m, 6H), 0.94–0.89 (m, 9H), 0.86 (t, J = 7.3 Hz, 9H); ^{13}C NMR (101 MHz, $CDCl_3$): δ (ppm) 161.2 (d, $J_{C\text{-}F}$ = 243.3 Hz), 149.5, 144.9, 144.0, 128.3 (d, $J_{C\text{-}F}$ = 7.9 Hz), 114.8 (d, $J_{C\text{-}F}$ = 21.4 Hz), 35.1, 32.4, 29.2, 27.5, 22.7, 14.2, 13.8, 11.1; ^{19}F NMR (282 MHz, $CDCl_3$): δ (ppm) –118.8, ^{119}Sn NMR (149 MHz, $CDCl_3$): δ (ppm) –50.2; IR (neat): $\tilde{\nu}$ (cm^{-1}) 2956, 2925, 2872, 2854, 1599, 1502, 1463, 1377, 1228, 1155, 1072, 830; HRMS (CI): calcd. for $C_{24}H_{42}FSn$ $[M+H]^+$: 469.2287, found 469.2287.

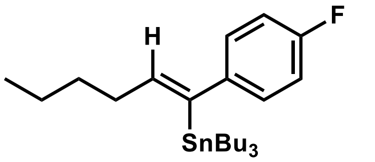

Chemical Formula: $C_{24}H_{41}FSn$
Molecular Weight: 467.30

Ethyl *(Z)*-4-(1-(tributylstannyl)hex-1-en-1-yl)benzoate (41). Prepared analogously, using ethyl 4-iodobenzoate as the coupling partner; brown oil (118 mg, 75%). ^{1}H NMR (400 MHz, $CDCl_3$): δ (ppm) 7.93 (m, app. br d, J = 8.3 Hz, 2H), 7.05 (m, app. br d, J = 8.4 Hz, 2H), 6.24 (t, J = 7.2 Hz, $J_{Sn\text{-}H}$ = 118.0 Hz, 1H), 4.36 (q, J =

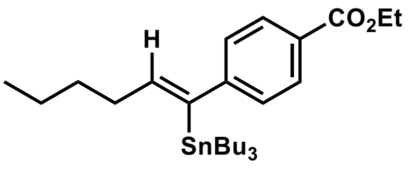

Chemical Formula: $C_{27}H_{46}O_2Sn$
Molecular Weight: 521.37

7.1 Hz, 2H), 2.17 (app. q, J = 7.3 Hz, 2H), 1.48–1.41 (m, 10H), 1.39 (t, J = 7.1 Hz, 3H), 1.32–1.21 (m, 6H), 0.96–0.89 (m, 9H), 0.85 (t, J = 7.3 Hz, 9H); ^{13}C NMR (101 MHz, $CDCl_3$): δ (ppm) 167.0, 153.2, 145.7, 144.7, 129.5, 127.3, 127.0, 60.8, 35.3, 32.3, 29.2, 27.4, 22.7, 14.5, 14.2, 13.8, 11.2; ^{119}Sn NMR (149 MHz, $CDCl_3$): δ (ppm) –48.7; IR (neat): $\tilde{\nu}$ (cm^{-1}) 2956, 2927, 2871, 2854, 1719, 1603, 1463, 1367, 1271, 1175, 1100, 1021, 770; HRMS (ESI): calcd. for $C_{27}H_{47}O_2Sn$ [M+H]$^+$: 523.2592, found 523.2593.

***(Z)*-Tributyl[1-(4-methoxyphenyl)-4-phenylbut-1-en-1-yl]stannane (42).** Prepared analogously, using 4-iodoanisole as the coupling partner; brown oil (40 mg, 76%). ^{1}H NMR (400 MHz, $CDCl_3$): δ (ppm) 7.34–7.27 (m, 2H), 7.25–7.18 (m, 3H), 6.95 (m, app. br d, J = 8.7 Hz, 2H), 6.81 (m, app. br d, J = 8.7 Hz, 2H), 6.24 (t, J = 7.2 Hz, J_{Sn-H} = 122.4 Hz, 1H), 3.80 (s, 3H), 2.82–2.71 (m, 2H), 2.52–2.41 (m, 2H), 1.47–1.39 (m, 6H), 1.32–1.21 (m, 6H), 0.93–0.88 (m, 6H), 0.85 (t, J = 7.3 Hz, 9H); ^{13}C NMR (101 MHz, $CDCl_3$): δ (ppm) 157.7, 145.4, 142.7, 142.0, 140.4, 128.6, 128.5, 128.0, 126.0, 113.5, 55.4, 37.3, 36.6, 29.3, 27.5, 13.8, 11.2; ^{119}Sn NMR (149 MHz, $CDCl_3$): δ (ppm) –50.7; IR (neat): $\tilde{\nu}$ (cm^{-1}) 2954, 2922, 2870, 2852, 1603, 1504, 1454, 1283, 1242, 1173, 1038, 824, 746, 697; HRMS (ESI): calcd. for $C_{29}H_{44}OSnNa$ [M+Na]$^+$: 551.2306, found 551.2304.

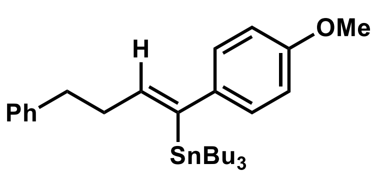

Chemical Formula: $C_{29}H_{44}OSn$
Molecular Weight: 527.38

***(Z)-tert*-Butyl{[4-(4-methoxyphenyl)-4-(tributylstannyl)but-3-en-1-yl]oxy}dimethylsilane (43).** Prepared analogously, using 4-iodoanisole as the coupling partner; brown oil (34 mg, 58%). ^{1}H NMR (400 MHz, $CDCl_3$): δ (ppm) 6.94 (m, app. br d, J = 8.7 Hz, 2H), 6.81 (m, app. br d, J = 8.8 Hz, 2H), 6.18 (t, J = 7.2 Hz, J_{Sn-H} = 122.8 Hz, 1H), 3.79 (s, 3H), 3.70 (t, J = 7.0 Hz, 1H), 2.44–2.35 (m, 2H), 1.49–1.41 (m, 6H), 1.33–1.22 (m, 6H), 0.96–0.91 (m, 6H), 0.91 (s, 9H), 0.86 (t, J = 7.3 Hz, 9H), 0.07 (s, 6H); ^{13}C NMR (101 MHz, $CDCl_3$): δ (ppm) 157.7, 146.9, 140.5, 139.7, 127.9, 113.5, 63.4, 55.4, 38.8, 29.2, 27.5, 26.1, 18.5, 13.8, 11.2, –5.1; ^{119}Sn NMR (149 MHz, $CDCl_3$): δ (ppm) –51.1; IR (neat): $\tilde{\nu}$ (cm^{-1}) 2954, 2927, 2855, 1604, 1504, 1463, 1284, 1243, 1173, 1093, 1040, 834, 774; HRMS (ESI): calcd. for $C_{28}H_{52}O_2SiSnNa$ [M+Na]$^+$: 605.2807, found 605.2807.

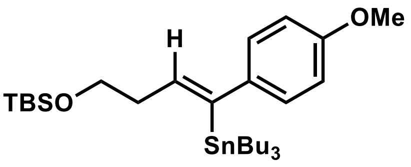

Chemical Formula: $C_{29}H_{54}O_2SiSn$
Molecular Weight: 581.54

***(Z)*-4-{4-[(*tert*-Butyldimethylsilyl)oxy]-1-(tributylstannyl)but-1-en-1-yl}benzonitrile (44).**

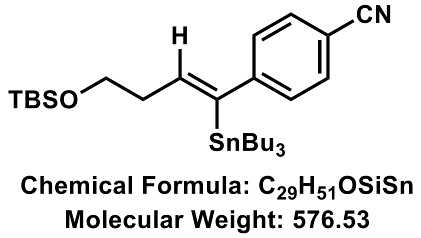

Prepared analogously, using 4-bromobenzonitrile as the coupling partner; colorless oil (34 mg, 59%). ^{1}H NMR (400 MHz, $CDCl_3$): δ (ppm) 7.54 (m, app. br d, J = 8.4 Hz, 2H), 7.06 (m, app. br d, J = 8.4 Hz, 2H), 6.23 (t, J = 7.1 Hz, $J_{Sn\text{-}H}$ = 112.7 Hz, 1H), 3.72 (t, J = 6.7 Hz, 2H), 2.45–2.36 (m, 2H), 1.45–1.38 (m, 6H), 1.30–1.23 (m, 6H), 0.96–0.91 (m, 6H), 0.90 (s, 9H), 0.85 (t, J = 7.3 Hz, 9H), 0.06 (s, 6H); ^{13}C NMR (101 MHz, $CDCl_3$): δ (ppm) 153.3, 146.7, 142.6, 132.0, 127.6, 119.5, 108.8, 63.0, 38.8, 29.1, 27.4, 26.1, 18.5, 13.8, 11.2, –5.1; ^{119}Sn NMR (149 MHz, $CDCl_3$): δ (ppm) –47.4; IR (neat): $\tilde{\nu}$ (cm^{-1}) 2956, 2828, 2856, 2227, 1600, 1463, 1255, 1096, 906, 835, 733; HRMS (ESI): calcd. for $C_{29}H_{51}NOSiSnNa$ [M+Na]$^{+}$: 600.2654, found 600.2652.

***(Z)-tert*-Butyldimethyl{[4-(tributylstannyl)hexa-3,5-dien-1-yl]oxy}silane(45).** Prepared analogously, using vinyl iodide as the coupling partner; brown solid material (35 mg, 70%). ^{1}H NMR (400 MHz, $CDCl_3$): δ (ppm) 6.50 (ddd, J = 17.3, 10.4, 1.1 Hz, 1H), 6.29 (td, J = 7.3, 1.2 Hz, 1H), 4.98 (dd, J = 17.1, 1.3 Hz, 1H), 4.88 (dd, J = 10.4, 1.4 Hz, 1H), 3.64 (t, J = 7.0 Hz, 2H), 2.37–2.29 (m, 2H), 1.54–1.47 (m, 6H), 1.36–1.27 (m, 6H), 1.02–0.95 (m, 6H), 0.92–0.85 (m, 18H), 0.05 (s, 6H); ^{13}C NMR (101 MHz, $CDCl_3$): δ (ppm) 145.2, 144.5, 142.4, 113.5, 63.3, 37.8, 29.3, 27.5, 26.1, 18.5, 13.8, 11.3, –5.1; ^{119}Sn NMR (149 MHz, $CDCl_3$): δ (ppm) –54.6; IR (neat): $\tilde{\nu}$ (cm^{-1}) 2955, 2928, 2857, 1611, 1463, 1254, 1173, 1101, 836, 776; HRMS (ESI): calcd. for $C_{24}H_{50}OSiSnNa$ [M+Na]$^{+}$: 525.2544, found 525.2539.

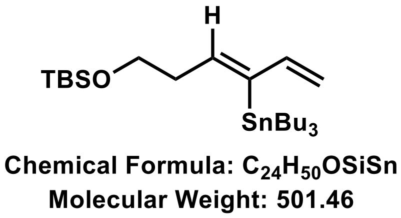

Silver-Mediated Chlorination of B(aam)-Capped Alkenylstannanes. $AgOP(O)Ph_2$ (65 mg, 0.2 mmol) and NCS (27 mg, 0.2 mmol) were stirred under Ar in an oven-dried Schlenk flask for 10 min until a homogenous greyish powder had formed. This material was suspended in dry acetone (1.5 mL). A solution of the alkenyl-B(aam) substrate (0.1 mmol, 1.0 equiv) in dry acetone (0.5 mL) was added over 60 min via syringe pump to this suspension. Once the addition was complete, the mixture was stirred for an additional 1 h at room temperature. The mixture was diluted with *tert*-butyl methyl ether and the reaction quenched with saturated ammonium chloride solution. The mixture was extracted twice with *tert*-butyl methyl ether, the combined extracts were washed with brine, dried over magnesium sulfate and concentrated under reduced

pressure. The crude material was purified by flash chromatography (hexane/EtOAc, 80:20) to yield the desired chlorinated alkene.

***(Z)*-2-(1-Chlorohex-1-en-1-yl)-2,3-dihydrobenzo[d][1,3,2]diazaborinin-4(1*H*)-one (47c).**

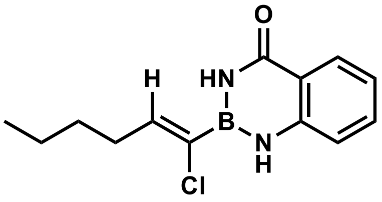

Chemical Formula: $C_{13}H_{16}BClN_2O$
Molecular Weight: 262.54

Prepared analogously as a white solid (15 mg, 57%). ^{1}H NMR (400 MHz, $CDCl_3$): δ (ppm) 8.35 (br s, 1H), 8.21 (dd, *J* = 8.0, 1.6 Hz, 1H), 7.53 (ddd, *J* = 8.5, 7.2, 1.6 Hz, 1H), 7.15 (ddd, *J* = 8.1, 7.2, 1.0 Hz, 1H), 7.09 (dd, *J* = 8.1, 1.0 Hz, 1H), 6.88 (br s, 1H), 6.71 (t, *J* = 6.8 Hz, 1H), 2.45 (app. q, *J* = 7.2 Hz, 2H), 1.57–1.47 (m, 2H), 1.47–1.36 (m, 2H), 0.95 (t, *J* = 7.2 Hz, 3H); ^{13}C NMR (101 MHz, $CDCl_3$): δ (ppm) 167.0, 144.0, 143.7, 134.1, 129.2, 129.0 (C(sp^2)-B by HMBC), 122.2, 119.1, 117.9, 30.4, 29.5, 22.5, 14.0; ^{11}B NMR (128 MHz, $CDCl_3$): δ (ppm) 26.6; IR (neat): $\tilde{\nu}$ (cm^{-1}) 3405, 3202 (br), 3101, 2950, 2926, 2867, 1656, 1616, 1513, 1490, 1414, 1339, 1306, 1264, 873, 757; HRMS (EI): calcd. for $C_{13}H_{16}BClN_2O$ $[M]^{+\bullet}$: 262.1039, found 262.1039.

***(Z)*-2-(1-Chloro-4-phenylbut-1-en-1-yl)-2,3-dihydrobenzo[d][1,3,2]diazaborinin-4(1*H*)-one (48a).**

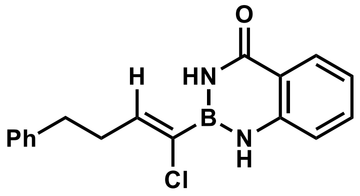

Chemical Formula: $C_{17}H_{16}BClN_2O$
Molecular Weight: 310.59

Prepared analogously as a white solid (15 mg, 48%). ^{1}H NMR (400 MHz, $CDCl_3$): δ (ppm) *δ* 8.21 (dd, *J* = 8.0, 1.6 Hz, 1H), 8.17 (br s, 1H), 7.54 (ddd, *J* = 8.5, 7.2, 1.6 Hz, 1H), 7.35–7.28 (m, 2H), 7.26–7.20 (m, 3H), 7.16 (ddd, *J* = 8.2, 7.2, 1.1 Hz, 1H), 7.08 (dd, *J* = 8.1, 1.0 Hz, 1H), 6.86 (br s, 1H), 6.70 (t, *J* = 6.4 Hz, 1H), 2.89–2.82 (m, 2H), 2.82–2.74 (m, 2H); ^{13}C NMR (101 MHz, $CDCl_3$): δ (ppm) 166.8, 143.9, 142.2, 141.0, 134.1, 129.6 (C(sp^2)-B by HMBC), 129.2, 128.7, 128.5, 126.4, 122.3, 119.1, 117.9, 34.2, 31.4; ^{11}B NMR (128 MHz, $CDCl_3$): δ (ppm) 26.5; IR (neat): $\tilde{\nu}$ (cm^{-1}) 3389, 3201 (br), 3025, 2921, 1655, 1615, 1523, 1491, 1416, 1298, 1264, 870, 763; HRMS (ESI): calcd. for $C_{17}H_{15}BClN_2O$ $[M-H]^-$: 309.0971, found 309.0971.

***(Z)*-2-{4-[(*tert*-Butyldimethylsilyl)oxy]-1-chlorobut-1-en-1-yl}-2,3-dihydrobenzo[d][1,3,2]diazaborinin-4(1*H*)-one (49a).** Prepared analogously as a white solid (20 mg, 55%). ^{1}H NMR (400 MHz, $CDCl_3$): δ (ppm) 8.21 (dd, *J* = 8.0, 1.6 Hz, 1H), 7.85 (br s, 1H), 7.54 (ddd, *J* = 8.1, 7.2, 1.6 Hz, 1H), 7.16 (ddd, *J* = 8.2, 7.3, 1.1 Hz, 1H), 7.08 (dd, *J* = 8.1, 0.6 Hz, 1H), 6.83 (br s, 1H), 6.67 (t, *J* = 6.6 Hz, 1H), 3.79 (t, *J* = 6.5 Hz, 2H), 2.67 (app. q, *J* = 6.6 Hz, 2H), 0.91 (s, 9H), 0.08 (s, 6H); ^{13}C NMR (101 MHz, $CDCl_3$): δ (ppm) *δ* 166.6, 143.8, 139.8, 134.1, 130.5 (C(sp^2)-B by HMBC), 129.3, 122.3, 119.1, 117.9, 61.4, 33.6, 26.1, 18.5, –5.1; ^{11}B NMR (128 MHz, $CDCl_3$): δ (ppm) 26.4; IR (neat): $\tilde{\nu}$ (cm^{-1}) 3410, 3378, 32085 (br), 2953, 2930, 1651, 1617, 1527, 1254, 1111, 836, 777, 755, 657; HRMS (ESI): calcd. for $C_{17}H_{26}BClN_2O_2SiNa$ [M+Na]$^+$: 387.1437, found 387.1438.

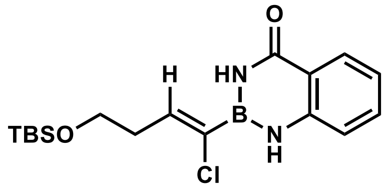

Chemical Formula: $C_{17}H_{26}BClN_2O_2Si$
Molecular Weight: 364.75

***(Z)*-2-(1-Chloro-2-cyclopentylvinyl)-2,3-dihydrobenzo[d][1,3,2]diazaborinin-4(1*H*)-one (50a).** Prepared analogously as a white solid (19 mg, 69 μmol, 69%). ^{1}H NMR (400 MHz, $CDCl_3$): δ (ppm) 8.20 (dd, *J* = 8.0, 1.6 Hz, 1H), 8.14 (br s, 1H), 7.53 (ddd, *J* = 8.5, 7.2, 1.6 Hz, 1H), 7.16 (ddd, *J* = 8.1, 7.2, 1.1 Hz, 1H), 7.08 (dd, *J* = 8.1, 1.0 Hz, 1H), 6.84 (br s, 1H), 6.59 (d, *J* = 8.6 Hz, 1H), 3.18 (app. h, *J* = 8.2 Hz, 1H), 2.03–1.90 (m, 2H), 1.81–1.71 (m, 2H), 1.71–1.60 (m, 2H), 1.47–1.34 (m, 2H); ^{13}C NMR (101 MHz, $CDCl_3$): δ (ppm) 166.8, 148.7, 144.0, 134.1, 129.2, 128.0 (C(sp^2)-B by HMBC), 122.2, 119.1, 117.9, 40.4, 32.7, 25.6; ^{11}B NMR (128 MHz, $CDCl_3$): δ (ppm) 26.6; IR (neat): $\tilde{\nu}$ (cm^{-1}) 3397, 3196 (br), 2946, 1657, 1618, 1523, 1492, 1415, 1310, 1265, 874, 756; HRMS (ESI): calcd. for $C_{14}H_{16}BClN_2ONa$ [M+Na]$^+$: 297.0936, found 297.0939.

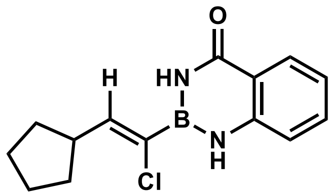

Chemical Formula: $C_{14}H_{16}BClN_2O$
Molecular Weight: 274.56

***(Z)*-2-(1-Fluorohex-1-en-1-yl)-2,3-dihydrobenzo[d][1,3,2]diazaborinin-4(1H)-one (47d).**[12] AgOP(O)Ph_2 (65 mg, 0.2 mmol, 2.0 equiv) and F-TEDA-PF_6 (94 mg, 0.2 mmol, 2.0 equiv) were stirred under argon in an flame-dried Schlenk flask at room temperature for 10 min until a homogenous greyish powder had formed. This material was suspended in dry acetone (1.5 mL). A solution of the alkenyl-B(aam) derivative **2e** (52 mg, 0.1 mmol) in dry acetone (0.5 mL) was added over 60 min via syringe pump to this suspension. Once the addition was complete, the mixture was stirred for an additional 2 h at room temperature. The mixture was diluted with *tert*-butyl methyl ether and the reaction quenched with saturated ammonium chloride solution.

The mixture was extracted twice with *tert*-butyl methyl ether, the combined extracts were washed with brine, dried over magnesium sulfate and concentrated under reduced pressure. The crude product was purified by flash chromatography (hexane/EtOAc, 70:30) to yield the title compound as a white solid (5.0 mg, 20%). ^{1}H NMR (400 MHz, $CDCl_3$): δ (ppm) 8.21 (dd, *J* = 8.0, 1.7 Hz, 1H), 7.74 (br s, 1H), 7.53 (ddd, *J* = 8.1, 7.2, 1.6 Hz, 1H), 7.16 (ddd, *J* = 8.1, 7.2, 1.1 Hz, 1H), 7.05 (dd, *J* = 8.3, 1.0 Hz, 1H), 6.70 (br s, 1H), 5.67 (dt, *J* = 45.8, 7.5 Hz, 1H), 2.31 (app. qd, *J* = 7.3, 1.7 Hz, 2H), 1.50–1.32 (m, 4H), 0.94 (t, *J* = 7.1 Hz, 3H); ^{13}C NMR (101 MHz, $CDCl_3$): δ (ppm) 166.5, 143.9, 134.1, 129.3, 124.9 (d, J_{C-F} = 8.1 Hz), 122.2, 119.3, 117.8, 31.1, 23.8 (d, J_{C-F} = 7.5 Hz), 22.4, 14.0; ^{19}F NMR (282 MHz, $CDCl_3$): δ (ppm) –131.3, ^{11}B NMR (128 MHz, $CDCl_3$): δ (ppm) 25.8; IR (neat): $\tilde{\nu}$ (cm^{-1}) 3425, 3198 (br), 2952, 2926, 2866, 1654, 1616, 1529, 1493, 1417, 1345, 1147, 887, 755; HRMS (EI): calcd. for $C_{13}H_{16}BFN_2O$ $[M]^{+\bullet}$: 246.1334, found 246.1335.

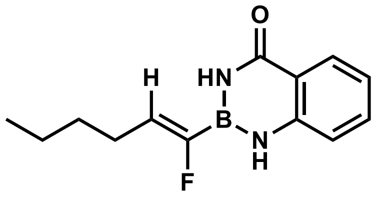

Chemical Formula: $C_{13}H_{16}BFN_2O$
Molecular Weight: 246.09

One-Pot *trans*-Selective Hydrostannation/Halodestannation.[13] A 25 mL flame-dried Schlenk tube was charged under Ar with the respective alkyne (1.0 mmol), $[Cp^*RuCl]_4$ (27.2 mg, 25 µmol, 2.5 mol%) and CH_2Cl_2 (5 mL). Bu_3SnH (0.14 mL, 0.525 mmol) was added at a rate of one drop every 5 seconds at 0°C.[1] Once the addition was complete, the mixture was stirred for 30 min. At this point, a solution of X_2 (Br_2, 28 µL or I_2, 139.6 mg, 0.55 mmol) in CH_2Cl_2 (5 mL) was added and stirring was continued for 30 min at 0°C. The reaction was quenched with aq. $Na_2S_2O_3$ (half-saturated, 4 mL), the organic layer was separated, and the aqueous layer extracted with CH_2Cl_2 (3 x 2 mL). The combined organic phases were dried under Mg_2SO_4, filtrated and concentrated under reduced pressure. The crude product was used to analyze the isomer ratio by ^{1}H NMR. The residue was suspended in pentane, sonicated, and the mixture filtered. The obtained brown solid was carefully rinsed with pentane to remove the tributyltin halide byproduct. The solid was dissolved in CH_2Cl_2, the solution was concentrated under reduced pressure and the residue purified by flash chromatography (CH_2Cl_2/AcOEt, 90:10) to yield the desired product. In some case, as precised later, a simple wash of the obtained solid with chilled CH_2Cl_2 to remove brown impurity sufficed to give product of ≥ 95% purity.

---

[1] When performed on 5 mmol scale, the Bu_3SnH (1.4 mL, 5.25 mmol, 1.05 equiv) was added over 5 min via syringe pump.

(*Z*)-2-(1-Iodohex-1-en-1-yl)-2,3-dihydrobenzo[*d*][1,3,2]diazaborinin-4(1*H*)-one (47a). Prepared analogously as a colorless solid (164.1 mg, 93%, *α*/*β* > 99:1, *Z*/*E* > 99:1) without flash column chromatography. ^{1}H NMR (400 MHz, $CDCl_3$): δ (ppm) 8.20 (dd, *J* = 8.0, 1.6 Hz, 1H), 7.83 (brs, 1H), 7.54 (ddd, *J* = 8.1, 7.2, 1.6 Hz, 1H), 7.16 (ddd, *J* = 8.1, 7.2, 1.0 Hz, 1H), 7.10 (dd, *J* = 8.2, 1.1 Hz, 1H), 6.76 (brs, 1H), 6.70 (t, *J* = 6.3 Hz, 1H), 2.42-2.32 (m, 2H), 1.60-1.48 (m, 2H), 1.47-1.35 (m, 2H), 0.96 (t, *J* = 7.2 Hz, 3H); ^{13}C NMR (101 MHz, $CDCl_3$): δ (ppm) 166.7, 152.1, 143.9, 134.1, 129.3, 122.3, 119.0, 117.9, 101.8 (C(sp^2)-B by HMBC), 39.0, 30.0, 22.5, 14.1; ^{11}B NMR (128 MHz, $CDCl_3$): δ (ppm) 27.0; IR (neat): $\tilde{\nu}$ (cm^{-1}) 3395, 3191, 3095, 2945, 2925, 2864, 1655, 1606, 1509, 1486, 871, 756, 641; HRMS (ESI): calcd. for $C_{13}H_{16}BIN_2ONa$ [M+Na]$^+$: 377.0293, found 377.0295.

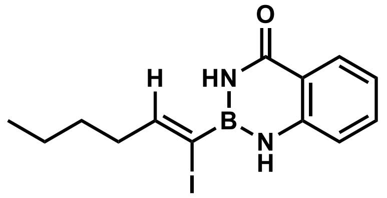

Chemical Formula: $C_{13}H_{16}BIN_2O$
Molecular Weight: 354.00

(*Z*)-2-(1-Bromohex-1-en-1-yl)-2,3-dihydrobenzo[*d*][1,3,2]diazaborinin-4(1*H*)-one (47b). Prepared analogously as a colorless solid (1.18 g, 77%, *α*/*β* > 99:1, *Z*/*E* > 99:1) without flash column chromatography. ^{1}H NMR (400 MHz, $CDCl_3$): δ (ppm) 8.20 (dd, *J* = 8.0, 1.8 Hz, 1H), 7.91 (brs, 1H), 7.54 (ddd, *J* = 8.5, 7.1, 1.5 Hz, 1H), 7.16 (ddd, *J* = 8.2, 7.3, 1.1 Hz, 1H), 7.09 (dd, *J* = 8.2, 1.1 Hz, 1H), 6.84 (t, *J* = 6.7 Hz, 1H), 6.83 (brs, 1H), 2.48-2.38 (m, 2H), 1.59-1.47 (m, 2H), 1.47-1.34 (m, 2H), 0.95 (t, *J* = 7.2 Hz, 3H); ^{13}C NMR (101 MHz, $CDCl_3$): δ (ppm) 166.7, 146.3, 143.9, 134.1, 129.3, 122.3, 121.7 (C(sp^2)-B by HMBC), 119.0, 117.9, 32.9, 30.2, 22.5, 14.0; ^{11}B NMR (128 MHz, $CDCl_3$): δ (ppm) 26.6; IR (neat): $\tilde{\nu}$ (cm^{-1}) 3406, 3217, 2951, 2917, 2853, 1651, 1609, 1509, 1486, 761, 642; HRMS (ESI): calcd. for $C_{13}H_{16}B^{79}BrN_2ONa$ [M+Na]$^+$: 329.0431, found 329.0431.

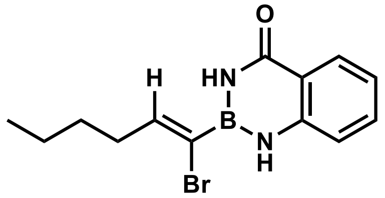

Chemical Formula: $C_{13}H_{16}BBrN_2O$
Molecular Weight: 307.00

(*Z*)-2-(1-Iodo-4-phenylbut-1-en-1-yl)-2,3-dihydrobenzo[*d*][1,3,2]diazaborinin-4(1*H*)-one (48b). Prepared analogously as a colorless solid (147.3 mg, 73%, *α*/*β* = 97:3, *Z*/*E* > 99:1) without flash column chromatography. ^{1}H NMR (400 MHz, $CDCl_3$): δ (ppm) 8.20 (dd, *J* = 7.9, 1.7 Hz, 1H), 7.62 (brs, 1H), 7.54 (ddd, *J* = 8.1, 7.2, 1.6 Hz, 1H), 7.35-7.29 (m, 2H), 7.26-7.20 (m, 3H), 7.16 (ddd, *J* = 8.1, 7.2, 1.1 Hz, 1H), 7.10-7.06 (m, 1H), 6.72 (brs, 1H), 6.69

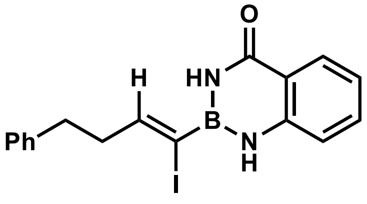

Chemical Formula: $C_{17}H_{16}BIN_2O$
Molecular Weight: 402.04

(t, J = 6.3 Hz, 1H), 2.86 (dd, J = 8.7, 6.7 Hz, 2H), 2.73-2.64 (m, 2H); ^{13}C NMR (101 MHz, $CDCl_3$): δ (ppm) 166.6, 150.5, 143.8, 140.7, 134.2, 129.3, 128.7, 128.5, 126.5, 122.4, 118.9, 117.9, 102.5 (C(sp^2)-B by HMBC), 40.7, 33.8; ^{11}B NMR (128 MHz, $CDCl_3$): δ (ppm) 26.9; IR (neat): $\tilde{\nu}$ (cm^{-1}) 3398, 3198, 3096, 2941, 2925, 1667, 1609, 1508, 1487, 1410, 876, 757, 744, 635; HRMS (ESI): calcd. for $C_{17}H_{17}BIN_2O$ $[M+H]^+$: 403.0473, found 403.0472.

(*Z*)-2-(4-(*tert*-Butyldimethylsiloxy)-1-iodobut-1-en-1-yl)-2,3-dihydrobenzo[*d*][1,3,2]diazaborinin-4(1*H*)-one (49c).

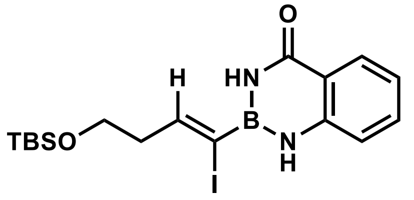

Chemical Formula: $C_{17}H_{26}BIN_2O_2Si$
Molecular Weight: 456.21

Prepared analogously as a colorless solid (1.83 g, 80%, α/β > 99:1, Z/E > 99:1) without flash column chromatography. ^{1}H NMR (400 MHz, $CDCl_3$): δ (ppm) 8.24-8.17 (m, 1H), 7.54 (ddd, J = 8.1, 7.2, 1.6 Hz, 1H), 7.48 (brs, 1H), 7.17 (ddd, J = 8.1, 7.2, 1.0 Hz, 1H), 7.09 (ddd, J = 8.1, 1.1, 0.5 Hz, 1H), 6.74 (t, J = 6.3 Hz, 1H), 6.72 (brs, 1H), 3.80 (t, J = 6.3 Hz, 2H), 2.57 (q, J = 6.3 Hz, 2H), 0.91 (s, 9H), 0.09 (s, 6H); ^{13}C NMR (101 MHz, $CDCl_3$): δ (ppm) 166.4, 148.6, 143.8, 134.1, 129.3, 122.4, 119.0, 117.9, 103.1 (C(sp^2)-B by HMBC), 61.0, 42.7, 26.1, 18.5, –5.1; ^{11}B NMR (128 MHz, $CDCl_3$): δ (ppm) 27.0; IR (neat): $\tilde{\nu}$ (cm^{-1}) 3347, 3221, 2927, 2856, 1644, 1612, 1487, 1098, 1069, 833, 756, 666; HRMS (ESI): calcd. for $C_{17}H_{26}BIN_2O_2SiNa$ $[M+Na]^+$: 479.0794, found 479.0798.

(*Z*)-2-(1-Bromo-2-cyclopentylvinyl)-2,3-dihydrobenzo[*d*][1,3,2]diazaborinin-4(1*H*)-one (50b).

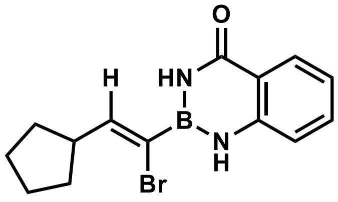

Chemical Formula: $C_{14}H_{16}BBrN_2O$
Molecular Weight: 319.01

Prepared analogously as a colorless solid (114.3 mg, 72%, α/β > 99:1, Z/E > 99:1). ^{1}H NMR (400 MHz, $CDCl_3$): δ (ppm) 8.20 (dd, J = 8.0, 1.6 Hz, 1H), 7.85 (brs, 1H), 7.54 (ddd, J = 8.1, 7.2, 1.6 Hz, 1H), 7.16 (ddd, J = 8.1, 7.2, 1.0 Hz, 1H), 7.09 (dd, J = 8.2, 1.3 Hz, 1H), 6.81 (brs, 1H), 6.74 (d, J = 8.4 Hz, 1H), 3.12 (hex, J = 8.4 Hz, 1H), 2.06-1.92 (m, 2H), 1.83-1.68 (m, 2H), 1.73-1.59 (m, 2H), 1.48-1.31 (m, 2H); ^{13}C NMR (101 MHz, CDCl3): δ (ppm) 166.7, 151.2, 143.9, 134.2, 129.3, 122.3, 119.7 (C(sp^2)-B by HMBC), 118.9, 117.9, 43.7, 32.5, 25.7; ^{11}B NMR (128 MHz, $CDCl_3$): δ (ppm) 26.6; IR (neat): $\tilde{\nu}$ (cm^{-1}) 3386, 3194, 2945, 2862, 1652, 1610, 1510, 1488, 1412, 1154, 869, 754; HRMS (ESI): calcd. for $C_{14}H_{17}B^{79}BrN_2O$ $[M+H]^+$: 319.0612, found 319.0611.

(*Z*)-2-(1-Bromo-5-chloropent-1-en-1-yl)-2,3-dihydrobenzo[*d*][1,3,2]diazaborinin-4(1*H*)-one (51). Prepared analogously as a colorless solid (130.9 mg, 80%, *α*/*β* = 96:4, *Z*/*E* > 99:1). ^{1}H NMR (400 MHz, $CDCl_3$): δ (ppm) 8.40 (brs, 1H), 8.24-8.17 (m, 1H), 7.55 (ddd, *J* = 8.1, 7.1, 1.5 Hz, 1H), 7.18 (ddd, *J* = 8.1, 7.1, 1.0 Hz, 1H), 7.10 (dd, *J* = 8.1, 1.3 Hz, 1H), 6.96 (t, *J* = 6.7 Hz, 1H), 6.88 (brs, 1H), 3.63 (t, *J* = 6.6 Hz, 2H), 2.66-2.55 (m, 2H), 2.05 (dt, *J* = 8.0, 6.8 Hz, 2H); ^{13}C NMR (101 MHz, $CDCl_3$): δ (ppm) 167.0, 144.2, 143.9, 134.2, 129.2, 123.1 (C(sp^2)-B by HMBC), 122.4, 119.1, 118.0, 44.3, 30.9, 30.5; ^{11}B NMR (128 MHz, $CDCl_3$): δ (ppm) 26.4; IR (neat): $\tilde{\nu}$ (cm^{-1}) 3408, 3213, 1651, 1608, 1507, 1486, 763, 641 HRMS (ESI): calcd. for $C_{12}H_{13}B^{79}Br^{35}ClN_2ONa$ [M+Na]$^+$: 348.9885, found 348.9885.

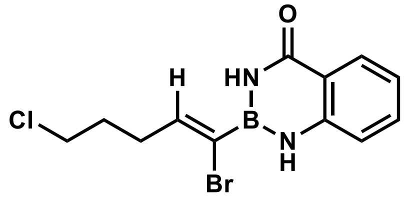

Chemical Formula: $C_{12}H_{13}BBrClN_2O$
Molecular Weight: 327.41

(*Z*)-2-(1-Bromo-3-methoxyprop-1-en-1-yl)-2,3-dihydrobenzo[*d*][1,3,2]diazaborinin-4(1*H*)-one (52). Prepared analogously as a yellow solid (93.2 mg, 63%, *α*/*β* = 98:2, *Z*/*E* > 99:1). ^{1}H NMR (400 MHz, $CDCl_3$): δ (ppm) 8.26-8.19 (m, 1H), 7.95 (brs, 1H), 7.55 (ddd, *J* = 8.1, 7.2, 1.5 Hz, 1H), 7.18 (ddd, *J* = 8.2, 7.2, 1.1 Hz, 1H), 7.12-7.08 (m, 1H), 7.05 (t, *J* = 4.8 Hz, 1H), 6.85 (brs, 1H), 4.27 (d, *J* = 4.7 Hz, 2H), 3.44 (s, 3H); ^{13}C NMR (101 MHz, $CDCl_3$): δ (ppm) 166.6, 143.7, 143.2, 134.2, 129.4, 122.5, 121.3 (C(sp^2)-B by HMBC), 119.1, 117.9, 73.3, 58.9; ^{11}B NMR (128 MHz, $CDCl_3$): δ (ppm) 26.4; IR (neat): $\tilde{\nu}$ (cm^{-1}) 3399, 3177, 3090, 2928, 2816, 1652, 1612, 1511, 1488, 1267, 1118, 768; HRMS (ESI): calcd. for $C_{11}H_{12}B^{79}BrN_2ONa$ [M+Na]$^+$: 317.0068, found 317.0066.

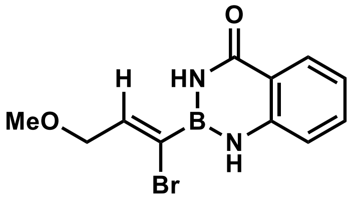

Chemical Formula: $C_{11}H_{12}BBrN_2O_2$
Molecular Weight: 294.94

(*Z*)-2-(2-Cyclopropyl-1-iodovinyl)-2,3-dihydrobenzo[*d*][1,3,2]diazaborinin-4(1*H*)-one (53). Prepared analogously as a colorless solid (124.1 mg, 73%, *α*/*β* > 99:1, *Z*/*E* > 99:1) without flash column chromatography. ^{1}H NMR (400 MHz, $CDCl_3$): δ (ppm) 8.17 (dd, *J* = 7.9, 1.7 Hz, 1H), 7.72 (brs, 1H), 7.53 (ddd, *J* = 8.1, 7.2, 1.6 Hz, 1H), 7.15 (ddd, *J* = 8.1, 7.2, 1.1 Hz, 1H), 7.10-7.02 (m, 1H), 6.70 (brs, 1H), 6.04 (d, *J* = 8.9 Hz, 1H), 1.98 (dtt, *J* = 9.1, 8.0, 4.6 Hz, 1H), 1.12-1.00 (m, 2H), 0.81-0.72 (m, 2H); ^{13}C NMR (101 MHz, $CDCl_3$): δ (ppm) 166.7, 155.8, 144.0, 134.1, 129.2, 122.2, 118.9, 117.9, 22.0, 8.5; ^{11}B NMR (128 MHz, $CDCl_3$):

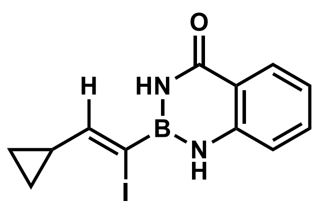

Chemical Formula: $C_{12}H_{12}BIN_2O$
Molecular Weight: 337.96

δ (ppm) 27.0; IR (neat): $\tilde{\nu}$ (cm^{-1}) 3413, 3216, 1662, 1617, 1604, 1506, 1487, 1400, 1305, 1157, 755, 634; HRMS (ESI): calcd. for $C_{12}H_{12}BIN_2ONa$ $[M+Na]^+$: 360.9980, found 360.9981.

Post-Functionalization of the Halide via Negishi Cross Coupling.[14,15] A 10 mL flame-dried Schlenk tube was charged under argon atmosphere with the selected alkenyl halide (0.2 mmol), the palladium catalyst (4 µmol, 2 mol%), and THF (1 mL). Next, the solution of the selected organozinc reagent (0.24 mmol, 1.2 equiv.) was added dropwise and stirring was continued until TLC showed full conversion. The mixture was concentrated under reduced pressure and the residue purified by flash chromatography (CH_2Cl_2/EtOAc, 90:10) to yield the desired product.

(*Z*)-2-(5-(*tert*-butyldimethylsiloxy)pent-2-en-2-yl)-2,3-dihydrobenzo[*d*][1,3,2]diazaborinin-4(1*H*)-one (54). Prepared using PEPPSI™-IPr as the catalyst; a colorless solid material (58.0 mg, 84%, *α*/*β* > 99:1, *Z*/*E* > 99:1). ^{1}H NMR (400 MHz, $CDCl_3$): δ (ppm) 8.24-8.15 (m, 1H), 7.50 (ddd, *J* = 8.1, 7.2, 1.6 Hz, 1H), 7.32 (brs, 1H), 7.11 (ddd, *J* = 8.1, 7.2, 1.1 Hz, 1H), 7.06-6.99 (m, 1H), 6.49 (brs, 1H), 6.19 (tq, *J* = 7.0, 1.7 Hz, 1H), 3.71 (t, *J* = 6.8 Hz, 2H), 2.51-2.41 (m, 2H), 1.85-1.82 (m, 3H), 0.90 (s, 9H), 0.07 (s, 6H); ^{13}C NMR (101 MHz, $CDCl_3$): δ (ppm) 166.8, 144.5, 139.0, 133.9, 130.7 (C(sp^2)-B by HMBC), 129.2, 121.7, 118.9, 117.6, 62.3, 32.7, 26.1, 18.5, 14.3, −5.1; ^{11}B NMR (128 MHz, $CDCl_3$): δ (ppm) 28.7; IR (neat): $\tilde{\nu}$ (cm^{-1}) 3350, 3247, 2951, 2926, 2884, 2856, 1629, 1615, 1525, 1103, 1037, 833, 757, 478; HRMS (ESI): calcd. for $C_{18}H_{30}BN_2O_2Si$ $[M+H]^+$: 345.2164, found 345.2165.

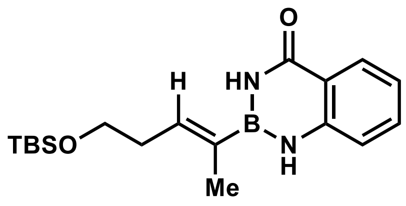

Chemical Formula: $C_{18}H_{29}BN_2O_2Si$
Molecular Weight: 344.34

2-((2*Z*,4*Z*)-1-(*tert*-Butyldimethylsiloxy)-7-phenylhepta-2,4-dien-4-yl)-2,3-dihydrobenzo[*d*][1,3,2]diazaborinin-4(1*H*)-one (55). The organozinc reagent was prepared as follow: A 10 mL flame-dried Schlenk tube was charged under argon atmosphere with (*Z*)-*tert*-butyl((3-iodoallyl)oxy)dimethylsilane (71.6 mg, 0.24 mmol, 1.2 equiv) and Et_2O (0.4 mL). The solution was cooled to −78°C before *tert*-butyllithium (1.6 M in pentane, 0.30 mL, 0.24 mmol) was added dropwise. After stirring for 1 h at −78°C, a solution of dry $ZnBr_2$ (54 mg, 0.24 mmol) in THF (0.3 mL) was added dropwise. Stirring was continued at −78°C for 15 min before the mixture was allowed to reach room temperature over the course of 30 min.

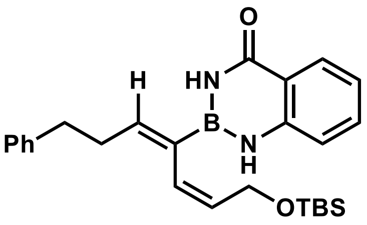

Chemical Formula: $C_{26}H_{35}BN_2O_2Si$
Molecular Weight: 446.47

The cross coupling was performed as described above, using this solution of the organozinc reagent and PEPPSI™-IPr as catalyst. The title compound was obtained as a yellow solid material (42.4 mg, 48%, *α*/*β* = 97:3, *Z*/*E* > 99:1). ^{1}H NMR (400 MHz, $CDCl_3$): δ (ppm) 8.20 (dd, *J* = 7.9, 1.7 Hz, 1H), 7.48 (ddd, *J* = 8.1, 7.2, 1.6 Hz, 1H), 7.45 (brs, 1H), 7.32-7.26 (m, 2H), 7.23-7.16 (m, 3H), 7.11 (ddd, *J* = 8.1, 7.2, 1.1 Hz, 1H), 6.99 (dd, *J* = 8.2, 1.2 Hz, 1H), 6.70 (brs, 1H), 6.32-6.24 (m, 1H), 6.09 (ddt, *J* = 11.2, 1.9, 1.2 Hz, 1H), 5.79 (dtd, *J* = 11.3, 6.8, 1.0 Hz, 1H), 3.99 (dd, *J* = 6.8, 1.3 Hz, 2H), 2.75 (t, *J* = 7.7 Hz, 2H), 2.58-2.43 (m, 2H), 0.84 (s, 9H), –0.01 (s, 6H); ^{13}C NMR (101 MHz, $CDCl_3$): δ (ppm) 166.8, 144.5, 143.8, 141.4, 133.8, 132.1, 129.2, 128.6, 128.5, 128.0, 126.2, 121.7, 119.0, 117.8, 60.3, 35.0, 32.1, 26.1, 18.5, –5.1; ^{11}B NMR (128 MHz, $CDCl_3$): δ (ppm) 27.9; IR (neat): $\tilde{\nu}$ (cm^{-1}) 3406, 3215, 2951, 2927, 2855, 1651, 1613, 1515, 1488, 1253, 1058, 833, 756; HRMS (ESI): calcd. for $C_{26}H_{35}BN_2O_2SiNa$ $[M+Na]^+$: 469.2453, found 469.2457.

(*Z*)-2-(1-(4-Fluorophenyl)hex-1-en-1-yl)-2,3-dihydrobenzo[*d*][1,3,2]diazaborinin-4(1*H*)-one (56). The organozinc reagent was prepared as follow: A 10 mL flame-dried Schlenk tube was charged under argon atmosphere with $ZnBr_2$ (135.1 mg, 0.60 mmol) and THF (2.2 mL). A solution of 4-fluorophenylmagnesium bromide (2 M in THF, 0.30 mL, 0.60 mmol) was added dropwise at 0°C and the resulting mixture was stirred at 0°C for 30 min.

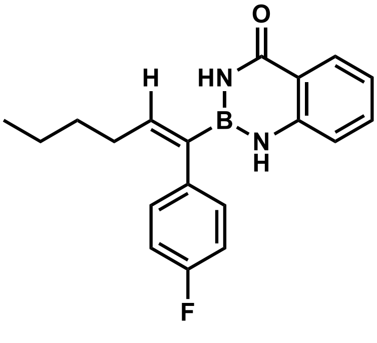

Chemical Formula: $C_{19}H_{20}BFN_2O$
Molecular Weight: 322.19

The cross coupling was performed as described above, using this solution of the organozinc reagent and $Pd(P^tBu_3)_2$ as the catalyst. The title compound was obtained as a yellow solid material (139.8 mg, 87%, *α*/*β* > 99:1, *Z*/*E* = 95:5). ^{1}H NMR (400 MHz, $CDCl_3$): δ (ppm) 8.18 (dd, *J* = 7.9, 1.7 Hz, 1H), 7.47 (ddd, *J* = 8.5, 7.2, 1.6 Hz, 1H), 7.26 (brs, 1H), 7.14-7.03 (m, 5H), 6.93 (dd, *J* = 8.1, 0.9 Hz, 1H), 6.45 (t, *J* = 7.2 Hz, 1H), 6.19 (brs, 1H), 2.09 (q, *J* = 7.2 Hz, 2H), 1.45-1.34 (m, 2H), 1.33-1.22 (m, 2H), 0.85 (t, *J* = 7.3 Hz, 3H); ^{13}C NMR (101 MHz, $CDCl_3$): δ (ppm) 166.7, 161.9 (d, *J* = 245.4 Hz), 145.6, 144.3, 136.1 (C(sp^2)-B by HMBC), 135.8 (d, *J* = 3.5 Hz), 133.9, 130.4 (d, *J* = 7.5 Hz), 129.3, 121.9, 118.9, 117.6, 115.9 (d, *J* = 21.1 Hz), 31.5, 30.0, 22.5, 14.0; ^{19}F NMR (282 MHz, $CDCl_3$): δ (ppm) –115.9, ^{11}B NMR (128 MHz, $CDCl_3$): δ (ppm) 28.6; IR

(neat): $\tilde{\nu}$ (cm^{-1}) 3421, 3185, 3103, 2948, 2924, 2865, 1657, 1608, 1504, 1487, 1401, 1207, 1152, 759; HRMS (ESI): calcd. for $C_{19}H_{19}BFN_2O$ [M-H]$^-$: 321.1580, found 321.1586.

(*Z*)-2-(1-Cyclopropyl-4-(trimethylsilyl)but-1-en-3-yn-2-yl)-2,3-dihydrobenzo[*d*][1,3,2]diazaborinin-4(1*H*)-one (57). The used alkynyl zinc bromide solution was prepared as follow: A 10 mL flame-dried Schlenk tube was charged under argon atmosphere with ethynyl-trimethylsilan (34 µL, 23.6 mg, 0.24 mmol) and THF (0.5 mL). The resulting solution was cooled to −78°C before *n*-butyllithium (1.6 M in hexane, 0.15 mL, 0.24 mmol) was added dropwise. The mixture was stirred for 1 h at −78°C before a solution of dry $ZnBr_2$ (54 mg, 0.24 mmol, 1.2 equiv) in THF (0.35 mL) was added dropwise. Stirring was continued at −78°C for 15 min before the mixture was allowed to reach room temperature.

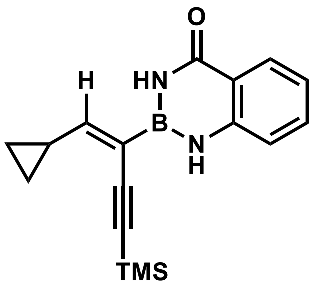

Chemical Formula: $C_{17}H_{21}BN_2OSi$
Molecular Weight: 308.26

The cross coupling was performed as described above, using this alkynyl zinc reagent and Pd(P^{t}Bu$_3$)$_2$ as the catalyst. The title compound was obtained as a colorless solid (49.1 mg, 80%, *α*/*β* > 99:1, *Z*/*E* > 99:1). ^{1}H NMR (400 MHz, $CDCl_3$): δ (ppm) 8.17 (dd, *J* = 7.9, 1.7 Hz, 1H), 7.79 (brs, 1H), 7.51 (ddd, *J* = 8.1, 7.2, 1.5 Hz, 1H), 7.13 (ddd, *J* = 8.1, 7.2, 1.1 Hz, 1H), 7.02 (dd, *J* = 8.2, 1.2 Hz, 1H), 6.68 (brs, 1H), 6.02 (d, *J* = 10.1 Hz, 1H), 2.29-2.16 (m, 1H), 1.12-1.06 (m, 2H), 0.77-0.69 (m, 2H), 0.28 (s, 9H); ^{13}C NMR (101 MHz, $CDCl_3$): δ (ppm) 166.9, 159.9, 144.2, 133.9, 129.2, 121.8, 119.0, 117.7, 113.6 (C(sp^2)-B by HMBC), 103.7, 100.6, 16.1, 9.5, 0.4; ^{11}B NMR (128 MHz, $CDCl_3$): δ (ppm) 27.5; IR (neat): $\tilde{\nu}$ (cm^{-1}) 3413, 1394, 2956, 2129, 1672, 1607, 1511, 1409, 1244, 837, 751; HRMS (ESI): calcd. for $C_{17}H_{22}BN_2OSi$ [M+H]$^+$: 309.1589, found 309.1587.

(*Z*)-2-(6-(*tert*-Butyldimethylsiloxy)-1-(1,3-dioxan-2-yl)hex-3-en-3-yl)-2,3-dihydrobenzo-[*d*][1,3,2]diazaborinin-4(1*H*)-one (58). The organozinc reagent was prepared as follow: A 10 mL flame-dried Schlenk tube was charged under argon atmosphere with $ZnBr_2$ (54 mg, 0.24 mmol) and THF (0.52 mL). A solution of (1,3-dioxan-2-ylethyl)magnesium bromide (0.5 M in THF, 0.24 mL, 0.48 mmol) was added dropwise at 0°C and the resulting mixture was stirred at 0°C for 30 min.

The cross coupling was performed as described above, using this solution of the organozinc reagent and PEPPSI™-IPr as the catalyst. The title compound was obtained as a yellow solid

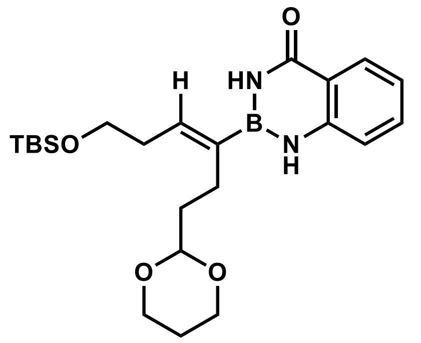

Chemical Formula: $C_{23}H_{37}BN_2O_4Si$
Molecular Weight: 444.45

material (77.2 mg, 77%, *α*/*β* > 99:1, *Z*/*E* > 99:1). ^{1}H NMR (400 MHz, $CDCl_3$): δ (ppm) 8.19 (dd, *J* = 7.9, 1.7 Hz, 1H), 7.49 (ddd, *J* = 8.1, 7.2, 1.6 Hz, 1H), 7.45 (brs, 1H), 7.11 (ddd, *J* = 8.1, 7.2, 1.1 Hz, 1H), 7.00 (brs, 1H), 6.98 (dd, *J* = 8.1, 1.4 Hz, 1H), 6.19 (t, *J* = 7.0 Hz, 1H), 4.55 (t, *J* = 4.9 Hz, 1H), 4.16 (ddd, *J* = 11.8, 4.9, 1.3 Hz, 2H), 3.81-3.73 (m, 2H), 3.71 (t, *J* = 6.7 Hz, 2H), 2.46 (q, *J* = 6.7 Hz, 2H), 2.39 (t, *J* = 7.4 Hz, 2H), 2.20-2.03 (m, 1H), 1.73 (ddd, *J* = 8.0, 7.0, 4.9 Hz, 2H), 1.37 (ddt, *J* = 13.4, 2.5, 1.2 Hz, 1H), 0.90 (s, 9H), 0.07 (s, 6H); ^{13}C NMR (101 MHz, $CDCl_3$): δ (ppm) 166.9, 144.7, 139.6, 135.5 (C(sp^2)-B by HMBC), 133.8, 129.2, 121.5, 119.0, 117.6, 101.3, 67.0, 62.6, 35.2, 32.5, 26.1, 25.8, 22.2, 18.5, −5.1; ^{11}B NMR (128 MHz, $CDCl_3$): δ (ppm) 28.5; IR (neat): $\tilde{\nu}$ (cm^{-1}) 3356, 3220, 2953, 2927, 2855, 1655, 1614, 1522, 1080, 835, 761; HRMS (ESI): calcd. for $C_{23}H_{37}BN_2O_4SiNa$ $[M+Na]^+$: 467.2508, found 467.2506.

(*Z*)-2-(4-Methoxy-1-(trimethylsilyl)but-2-en-2-yl)-2,3-dihydrobenzo[*d*][1,3,2]diazaborinin-4(1*H*)-one (59). The organozinc reagent was prepared as follow: A 10 mL flame-dried Schlenk tube was charged under argon atmosphere with $ZnBr_2$ (54 mg, 0.24 mmol, 1.2 equiv) and THF (0.76 mL). A solution of (trimethylsilyl)methylmagnesium chloride (1 M in THF, 0.24 mL, 0.24 mmol) was added dropwise at 0°C and the resulting mixture was stirred at 0°C for 30 min.

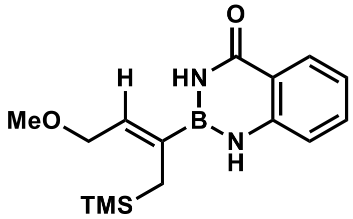

Chemical Formula: $C_{15}H_{23}BN_2O_2Si$
Molecular Weight: 302.26

The cross coupling was performed as described above, using this solution of the organozinc reagent and PEPPSI™-IPr as catalyst. The title compound was obtained as a yellow solid material (42.6 mg, 71%, *α*/*β* = 98:2, *Z*/*E* > 99:1). ^{1}H NMR (400 MHz, $CDCl_3$): δ (ppm) 8.26-8.12 (m, 1H), 7.51 (ddd, *J* = 8.1, 7.2, 1.5 Hz, 1H), 7.23 (brs, 1H), 7.13 (ddd, *J* = 8.1, 7.2, 1.0 Hz, 1H), 7.02 (ddd, *J* = 8.1, 1.1, 0.5 Hz, 1H), 6.47 (brs, 1H), 6.08 (tt, *J* = 5.8, 1.1 Hz, 1H), 4.06 (d, *J* = 5.7 Hz, 2H), 3.39 (s, 3H), 1.75 (s, 2H), 0.00 (s, 9H); ^{13}C NMR (101 MHz, $CDCl_3$): δ (ppm) 166.7, 144.3, 134.6, 134.0, 129.3, 121.9, 118.9, 117.6, 69.8, 58.6, 21.0, −1.1; ^{11}B NMR (128 MHz, $CDCl_3$): δ (ppm) 29.3; IR (neat): $\tilde{\nu}$ (cm^{-1}) 3320, 3289, 2947, 2912, 1650, 1615, 1518, 1486, 1247, 1145, 1082, 842, 754; HRMS (ESI): calcd. for $C_{15}H_{23}BN_2O_2SiNa$ $[M+Na]^+$: 325.1514, found 325.1514.

Further Post-Functionalizations of the B(aam) Part

(*E*)-*tert*-Butyl((4-(4-methoxyphenyl)pent-3-en-1-yl)oxy)dimethylsilane (60).[16–18] A 2-5 mL microwave Biotage tube was charged under argon with the the alkenyl-B(aam) derivative **54** (34.4 mg, 0.1 mmol), $Pd(PPh_3)_4$ (5.8 mg, 5 µmol, 5 mol%), and 1,4-dioxane (0.5 mL). 4-Bromo-anisol (13 µL, 18.7 mg, 0.1 mmol) was added, followed by a solution of potassium *tert*-butoxide in THF (1 M, 0.11 mL, 0.11 mmol). The tube was sealed and the mixture was vigorously stirred at 100°C for 2 h. The mixture was cooled to room temperature and concentrated under reduced pressure. The crude material was purified by flash chromatography (hexane/EtOAc, 90:10) to yield the title compound as a colorless liquid (20.8 mg, 68%). ^{1}H NMR (400 MHz, $CDCl_3$): δ (ppm) 7.35-7.29 (m, 2H), 6.87-6.82 (m, 2H), 5.70 (tq, *J* = 7.2, 1.4 Hz, 1H), 3.81 (s, 3H), 3.70 (t, *J* = 7.1 Hz, 2H), 2.48-2.38 (m, 2H), 2.07-1.99 (m, 3H), 0.91 (s, 9H), 0.07 (s, 6H); ^{13}C NMR (101 MHz, $CDCl_3$): δ (ppm) 158.6, 136.6, 135.9, 126.7, 122.9, 113.7, 63.0, 55.4, 32.8, 26.1, 18.5, 16.1, −5.1; IR (neat): $\tilde{\nu}$ (cm^{-1}) 2953, 2928, 2856, 1608, 1511, 1245, 1179, 1092, 1036, 935, 824, 773; HRMS (CI): calcd. for $C_{18}H_{31}O_2Si$ $[M+H]^+$: 307.2088, found 307.2087

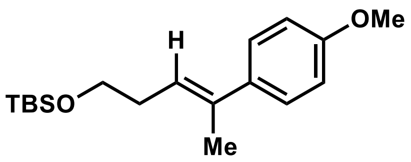

Chemical Formula: $C_{18}H_{30}O_2Si$
Molecular Weight: 306.52

(*E*)-5-(*tert*-Butyldimethylsiloxy)-1-(4-methoxyphenyl)-2-methylpent-2-en-1-ol (61).[19] A 2-5 mL microwave Biotage tube was charged under argon with the alkenyl-B(aam) derivative **54** (51.6 mg, 0.15 mmol), $[Rh(cod)Cl]_2$ (2.2 mg, 4.5 µmol, 3 mol%), K_3PO_4 (47.8 mg, 0.225 mmol), 1,4-dioxane (0.5 mL) and water (0.13 mL). *p*-Anisaldehyde (37 µL, 40.8 mg, 0.30 mmol) was added and the tube was sealed. The mixture was then heated under microwave irradiation at 140°C for 30 min, before it was cooled to room temperature and diluted with MeOH (1 mL). $NaBH_4$ (11.3 mg, 0.30 mmol) was added carefully and the resulting mixture was stirred at room temperature for 1 h. The reaction was quenched with water and the mixture extracted with ethyl acetate. The combined organic phases were dried over $MgSO_4$, filtrated and concentrated under reduced pressure. The residue was purified by flash chromatography (hexane/EtOAc, 80:20) to yield the title compound as a colorless liquid (26.8 mg, 53%). ^{1}H NMR (400 MHz, $CDCl_3$): δ (ppm) 7.30-7.26 (m, 2H), 6.89-6.84 (m, 2H), 5.64 (tq, *J* = 7.2, 1.3 Hz, 1H), 5.08 (s, 1H), 3.80 (s, 3H), 3.66 (t, *J* = 7.0 Hz, 2H), 2.35-2.27 (m, 2H), 1.78 (brs, 1H), 1.53-1.47 (m, 3H), 0.90 (s,

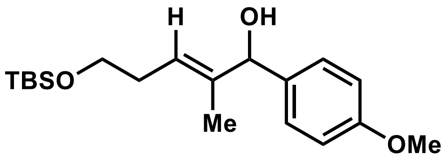

Chemical Formula: $C_{19}H_{32}O_3Si$
Molecular Weight: 336.55

9H), 0.06 (s, 6H); ^{13}C NMR (101 MHz, $CDCl_3$): δ (ppm) 159.1, 138.8, 134.7, 127.7, 122.7, 113.8, 78.9, 62.9, 55.4, 31.7, 26.1, 18.5, 12.6, –5.1; IR (neat): $\tilde{\nu}$ (cm^{-1}) 3428, 2953, 2929, 2898, 2856, 1611, 1510, 1247, 1093, 1037, 832, 810, 774; HRMS (CI): calcd. for $C_{19}H_{31}O_3Si$ $[M-H]^-$ 335.2037, found 335.2034.

(*E*)-3-(5-(*tert*-Butyldimethylsiloxy)pent-2-en-2-yl)cyclopentan-1-one (62).[14] **A** 2-5 mL microwave Biotage tube was charged under argon with the alkenyl-B(aam) derivative **54** (51.65 mg, 0.15 mmol), $[Rh(cod)Cl]_2$ (2.2 mg, 4.5 μmol, 3 mol%), K_3PO_4 (47.8 mg, 0.225 mmol), 1,4-dioxane (0.5 mL) and water (0.13 mL). 2-Cyclopentenone (25 μL, 24.6 mg, 0.30 mmol) was added and the tube was sealed. The mixture was then heated under microwave irradiation at 140°C for 30 min, before it was cooled to room temperature and filtrated through a short plug of silica, eluting with hexane/EtOAc (9:1). The combined filtrates were concentrated under reduced pressure and the residue was purified by flash chromatography (hexane/EtOAc, 90:10) to yield the title compound as a colorless liquid (25.8 mg, 61%). ^{1}H NMR (400 MHz, $CDCl_3$): δ (ppm) 5.23 (tquint, J = 7.1, 1.4 Hz, 1H), 3.60 (t, J = 6.8 Hz, 2H), 2.76 (ddd, J = 16.7, 11.2, 6.7 Hz, 1H), 2.39-2.29 (m, 2H), 2.26 (qt, J = 7.0, 0.9 Hz, 2H), 2.23-2.06 (m, 3H), 1.82-1.69 (m, 1H), 1.67-1.64 (m, 3H), 0.89 (s, 9H), 0.05 (s, 6H); ^{13}C NMR (101 MHz, $CDCl_3$): δ (ppm) 219.3, 137.4, 120.7, 63.0, 45.3, 43.6, 38.7, 31.8, 28.2, 26.1, 18.5, 14.8, –5.1; IR (neat): $\tilde{\nu}$ (cm^{-1}) 2954, 2928, 2895, 2856, 1743, 1253, 1093, 832, 773; HRMS (CI): calcd. for $C_{16}H_{31}O_2Si$ $[M+H]^+$: 283.2088, found 283.2085.

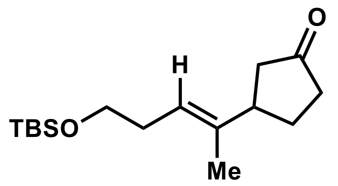

Chemical Formula: $C_{16}H_{30}O_2Si$
Molecular Weight: 282.50

References

1. Y. Nishihara, Y. Okada, J. Jiao, M. Suetsugu, M.-T. Lan, M. Kinoshita, M. Iwasaki, K. Takagi, *Angew. Chem. Int. Ed.* **2011**, *50*, 8660–8664.
2. M. D. Mbaye, B. Demerseman, J.-L. Renaud, L. Toupet, C. Bruneau, *Adv. Synth. Catal.* **2004**, *346*, 835–841.
3. G. A. Molander, B. W. Katona, F. Machrouhi, *J. Org. Chem.* **2002**, *67*, 8416–8423.
4. V. V. Bardin, N. Y. Adonin, H.-J. Frohn, *Z. Anorg. Allg. Chem.* **2012**, *638*, 565–579.
5. P. J. Fagan, M. D. Ward, J. C. Calabrese, *J. Am. Chem. Soc.* **1989**, *111*, 1698–1719.
6. J. Willwacher, B. Heggen, C. Wirtz, W. Thiel, A. Fürstner, *Chem. Eur. J.* **2015**, *21*, 10416–10430.
7. S. Größl, Total Synthesis of Belizentrin Methyl Ester : The Polyhydroxylated Sidechain, Dissertation, TU Dortmund, **2018**.
8. A. Fürstner, J. W. J. Kennedy, *Chem. Eur. J.* **2006**, *12*, 7398–7410.
9. K. Semba, T. Fujihara, J. Terao, Y. Tsuji, *Angew. Chem. Int. Ed.* **2013**, *52*, 12400–12403.
10. N. Huwyler, K. Radkowski, S. M. Rummelt, A. Fürstner, *Chem. Eur. J.* **2017**, *23*, 12412–12419.
11. R. S Coleman, X. Lu, *Chem. Commun.* **2006**, 423-425.
12. H. Sommer, A. Fürstner *Chem. Eur. J.* **2017**, *23*, 558-562.
13. R. N. Hanson, H. El-Wakil, *J. Org. Chem.* **1987**, *52*, 3687–3688.
14. C. Wang, Z. Xu, T. Tobrman, E. Negishi, *Adv. Synth. Catal.* **2010**, *352*, 627–631.
15. S. Xu, C.-T. Lee, H. Rao, E. Negishi, *Adv. Synth. Catal.* **2011**, *353*, 2981–2987.
16. Y. Mutoh, K. Yamamoto, S. Saito, *ACS Catal.* **2020**, *10*, 352–357.
17. K. Yamamoto, Y. Mohara, Y. Mutoh, S. Saito, *J. Am. Chem. Soc.* **2019**, *141*, 17042–17047.
18. H. Yoshida, M. Seki, S. Kamio, H. Tanaka, Y. Izumi, J. Li, I. Osaka, M. Abe, H. Andoh, T. Yajima, et al., *ACS Catal.* **2020**, *10*, 346–351.
19. S. Kamio, I. Kageyuki, I. Osaka, H. Yoshida, *Chem. Commun.* **2019**, *55*, 2624–2627.

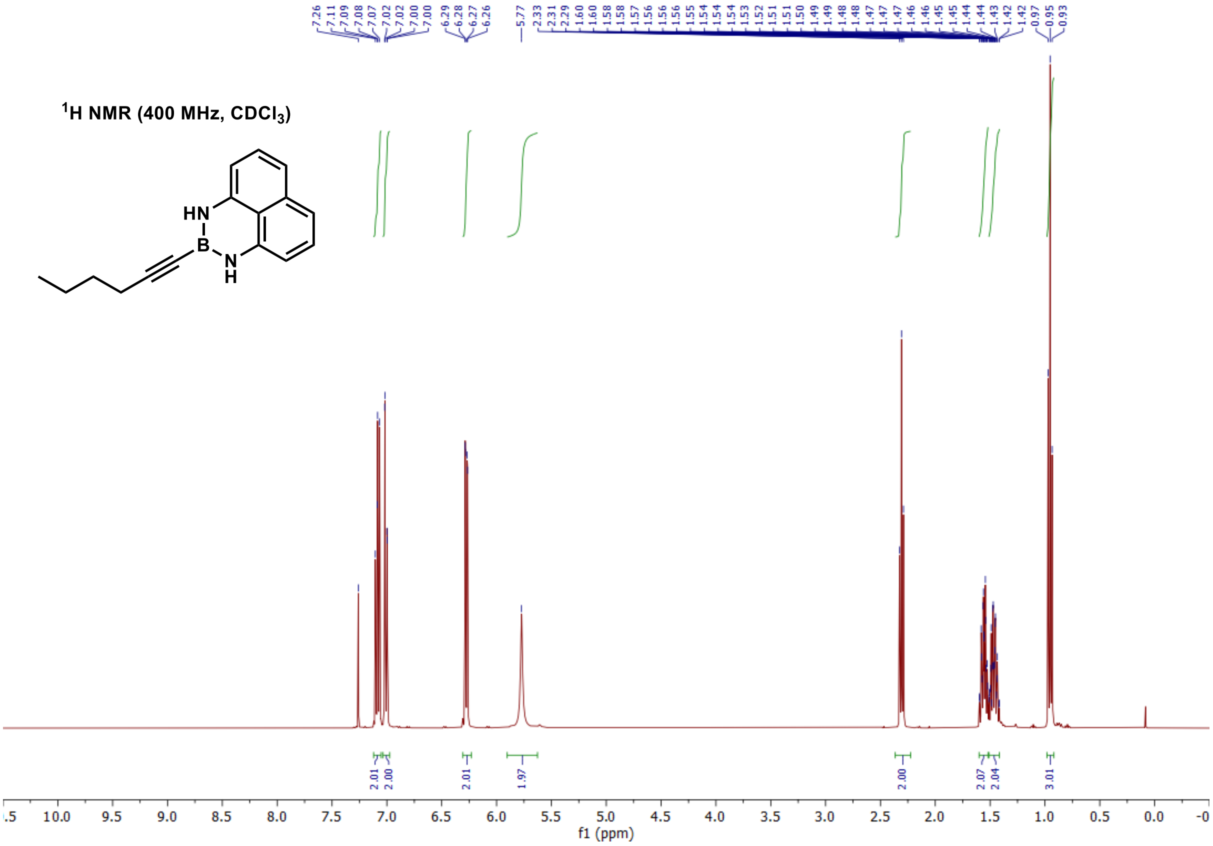
1H NMR (400 MHz, CDCl3)
HN
B
N
H
7.26 7.11 7.09 7.08 7.07 7.02 7.02 7.00 7.00 6.29 6.28 6.27 6.26 5.77 2.33 2.31 2.29 1.60 1.60 1.58 1.58 1.57 1.56 1.56 1.55 1.55 1.54 1.54 1.54 1.53 1.53 1.52 1.51 1.51 1.50 1.49 1.49 1.48 1.48 1.47 1.47 1.47 1.46 1.46 1.45 1.45 1.44 1.44 1.43 1.43 1.42 1.42 0.97 0.95 0.93
2.01 2.00 2.01 1.97 2.00 2.07 2.04 3.01
f1 (ppm)

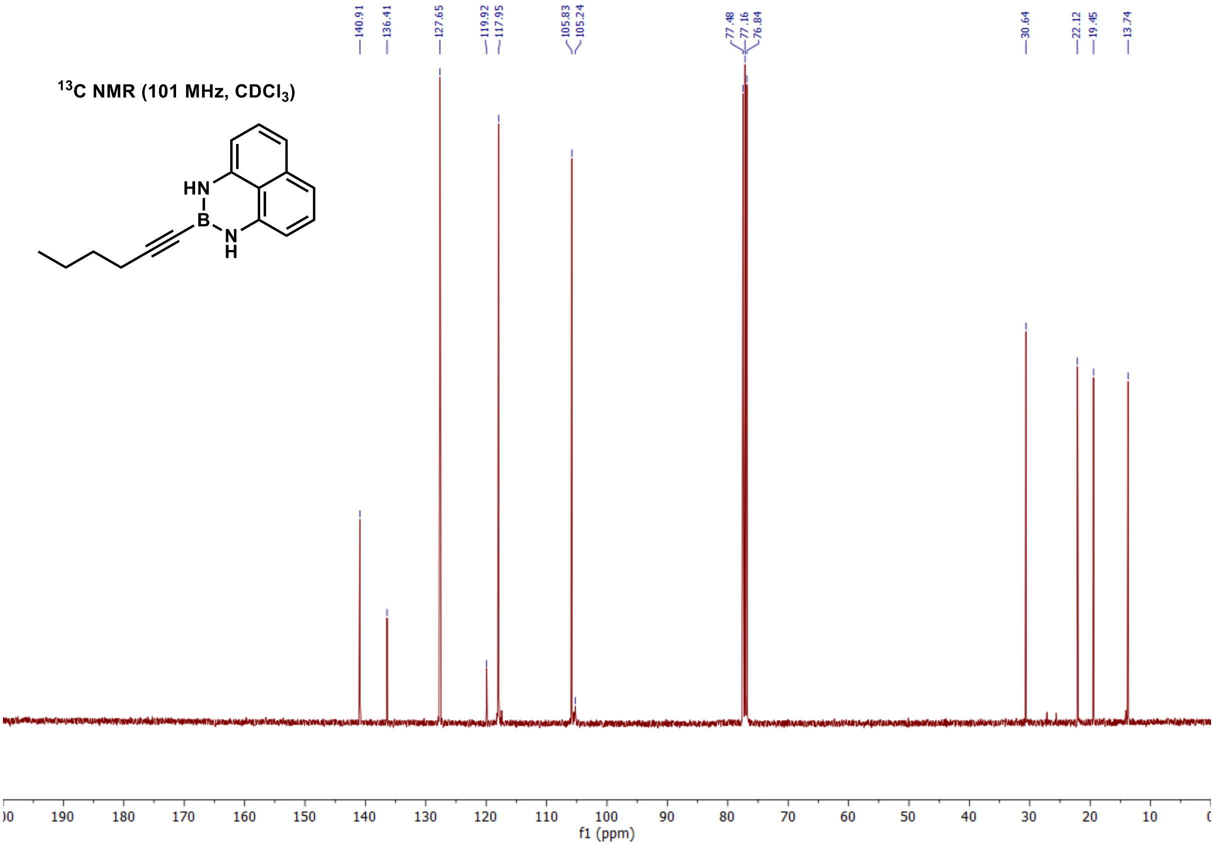
13C NMR (101 MHz, CDCl3)
HN
B
N
H
140.91 136.41 127.65 119.92 117.95 105.83 105.24 77.48 77.16 76.84 30.64 22.12 19.45 13.74
f1 (ppm)

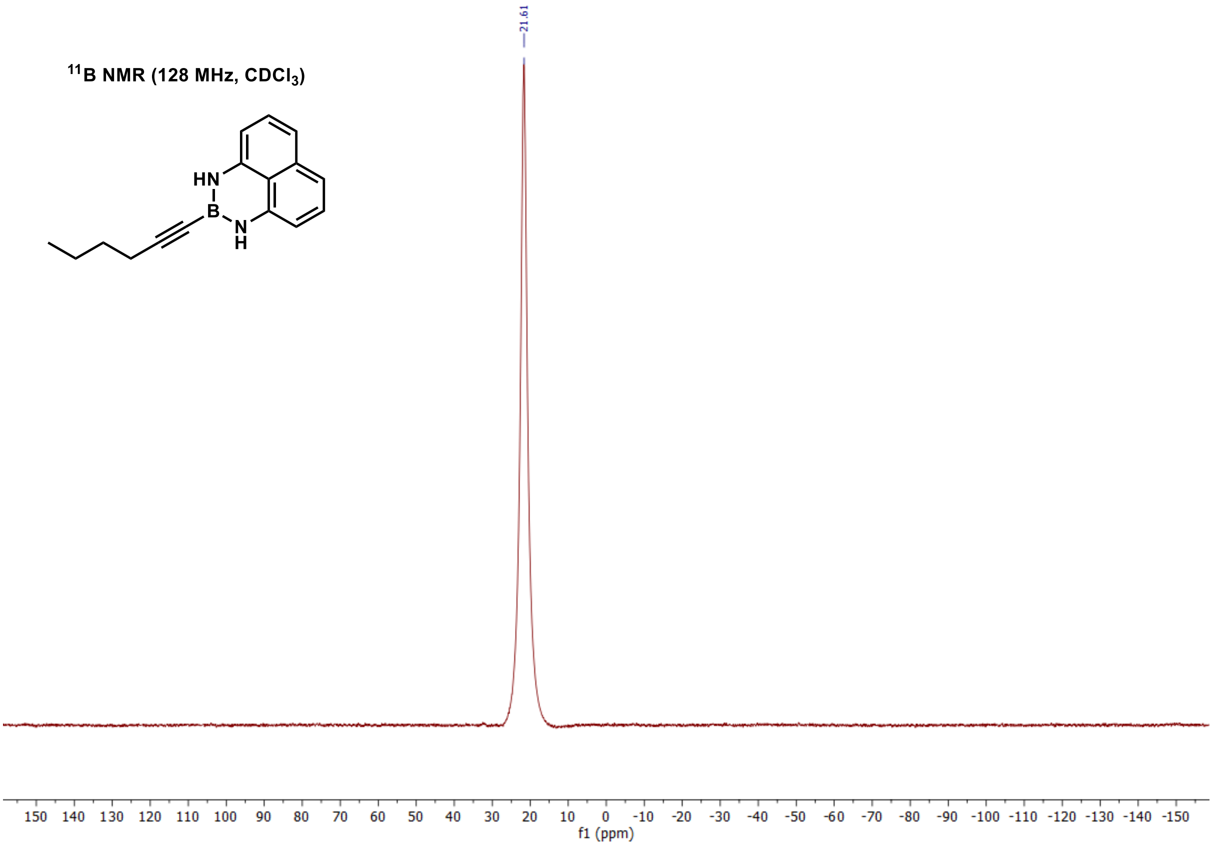

11B NMR (128 MHz, CDCl3)
HN
B
N
H
21.61
150 140 130 120 110 100 90 80 70 60 50 40 30 20 10 0 -10 -20 -30 -40 -50 -60 -70 -80 -90 -100 -110 -120 -130 -140 -150
f1 (ppm)

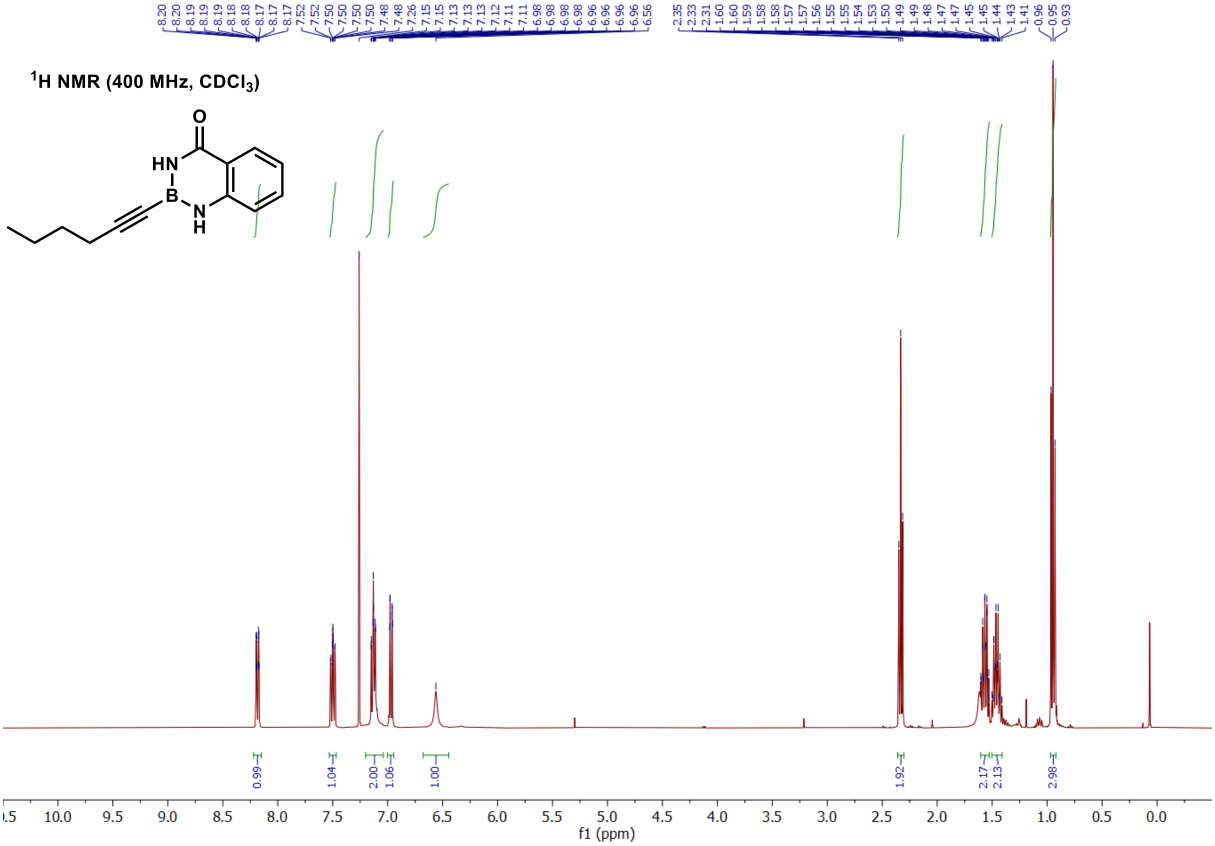
^{1}H NMR (400 MHz, $CDCl_3$)
f1 (ppm)

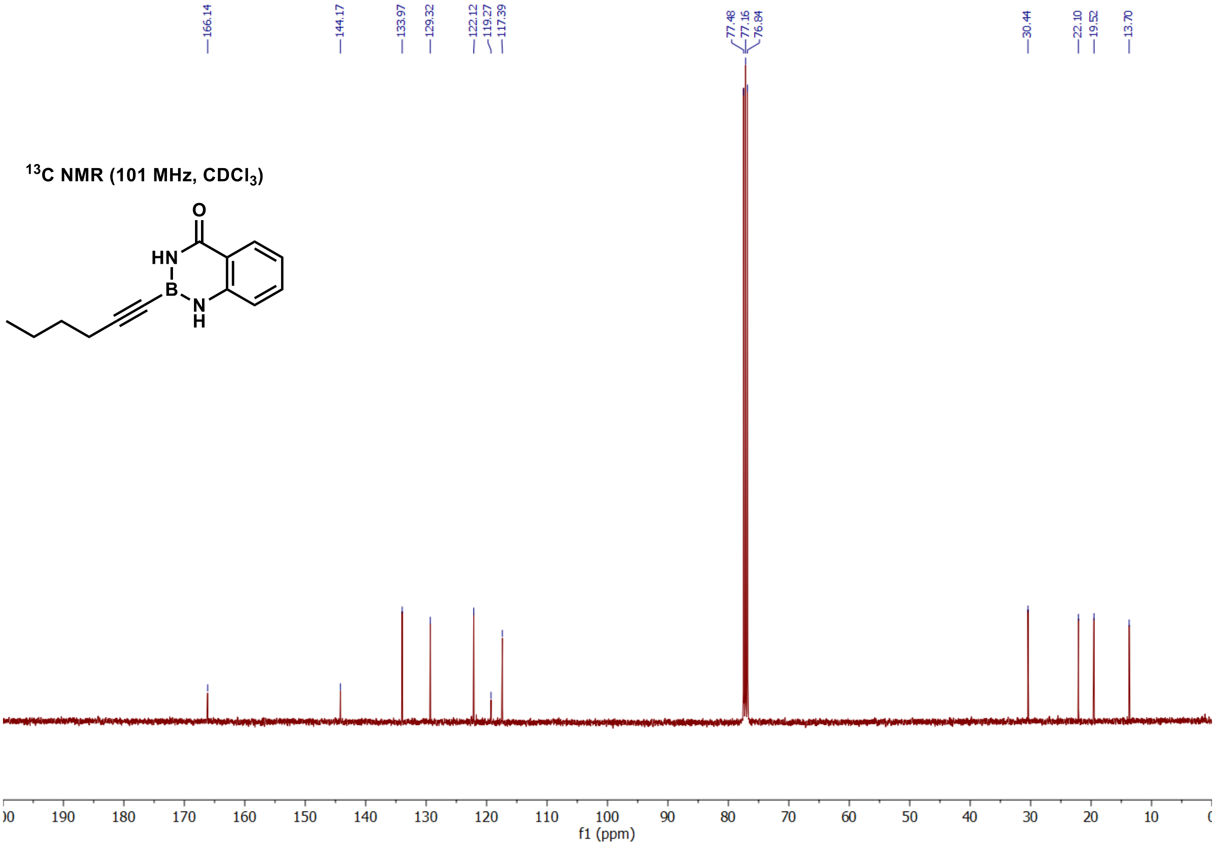
^{13}C NMR (101 MHz, $CDCl_3$)
f1 (ppm)

^{11}B NMR (128 MHz, $CDCl_3$)

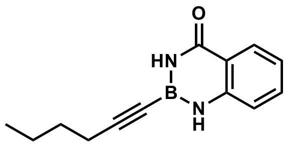

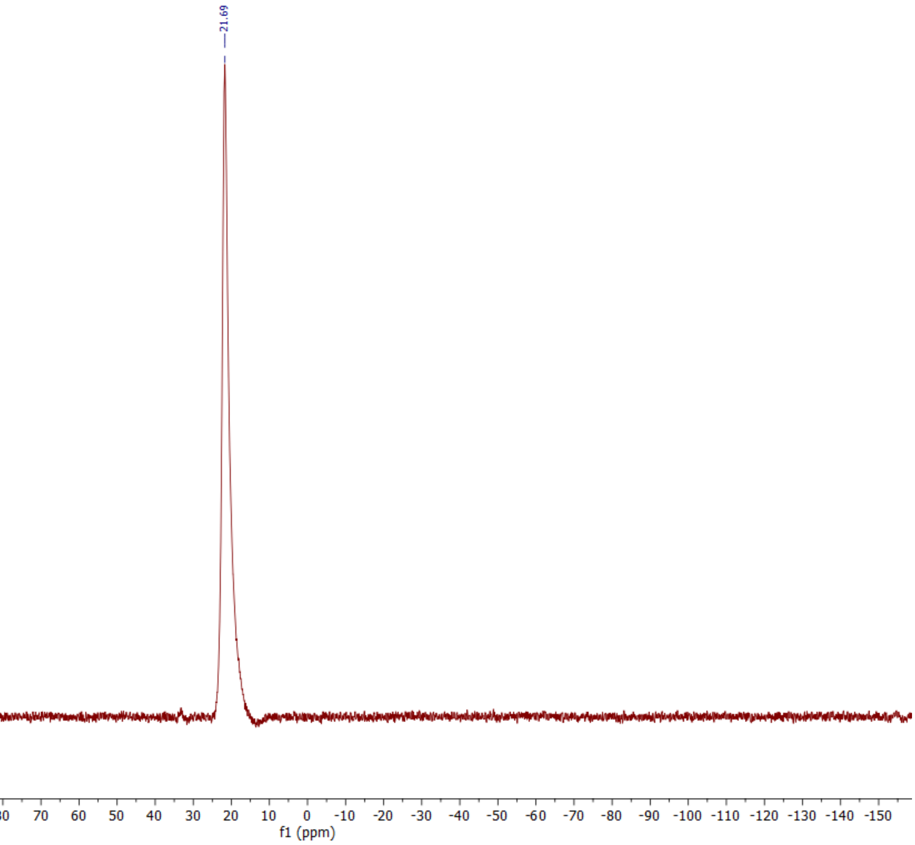

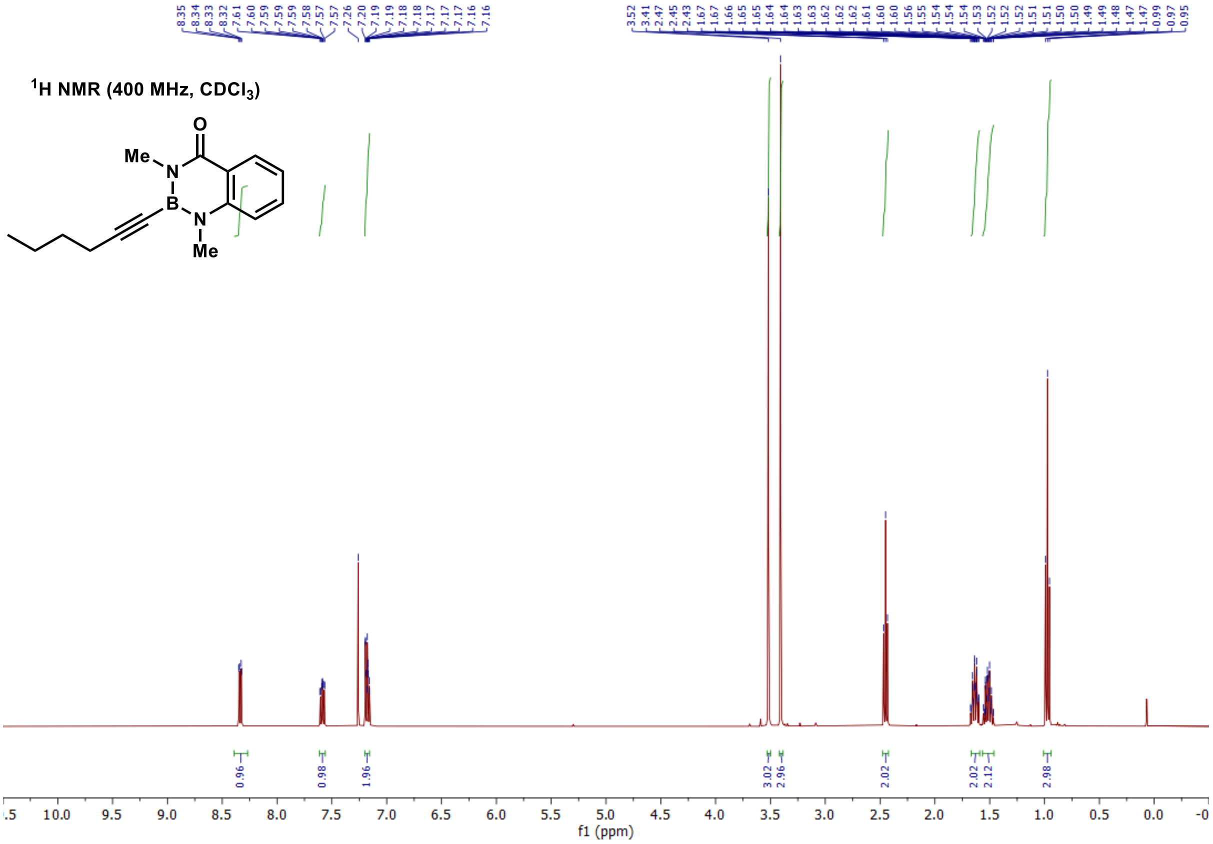
^{1}H NMR (400 MHz, CDCl$_3$)
Me
O
N
B
N
Me
f1 (ppm)

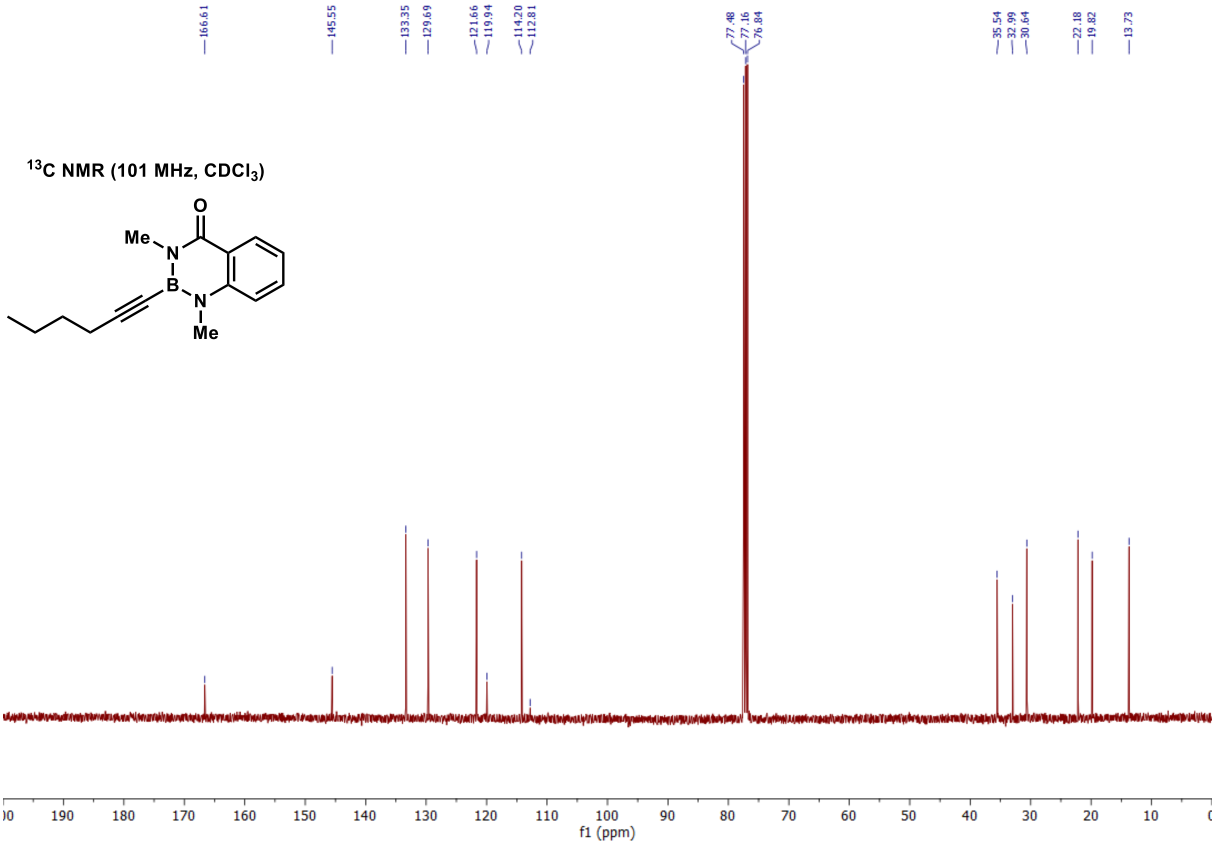
^{13}C NMR (101 MHz, CDCl$_3$)
Me
O
N
B
N
Me
f1 (ppm)

^{11}B NMR (128 MHz, $CDCl_3$)

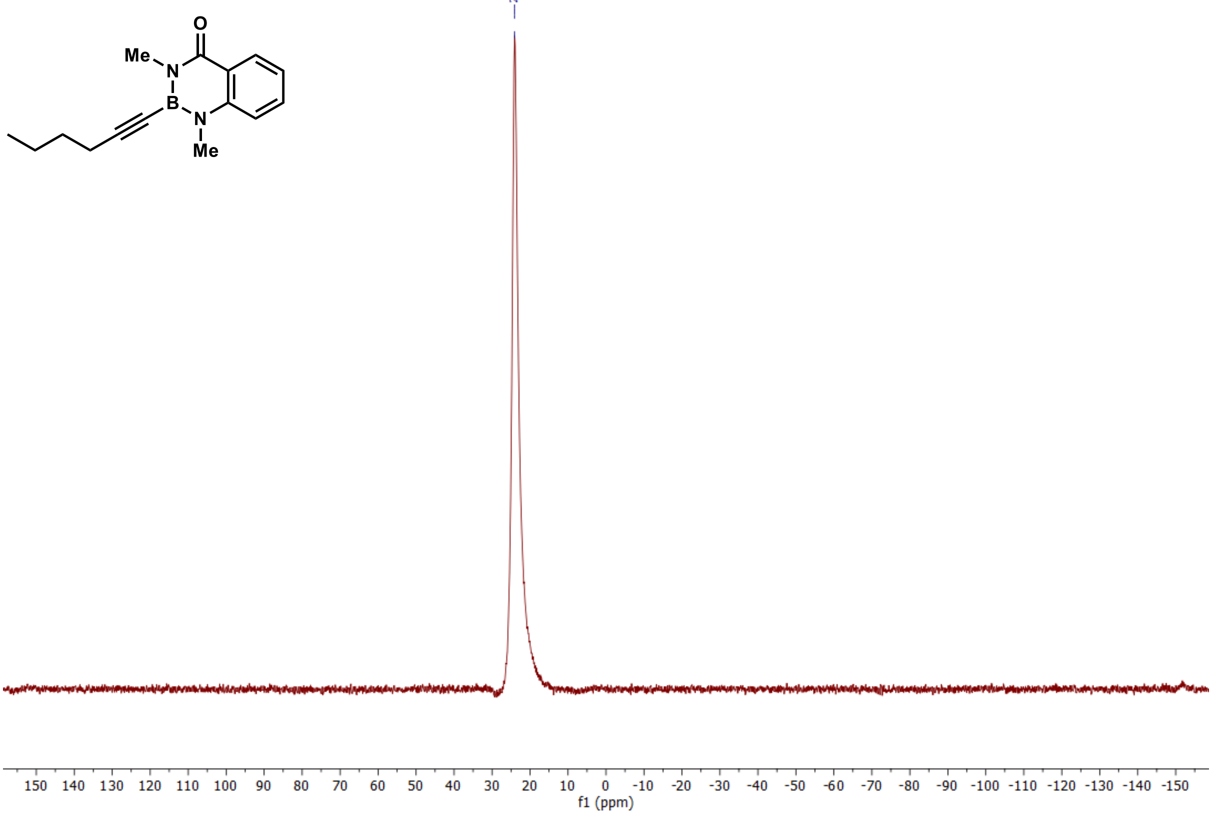

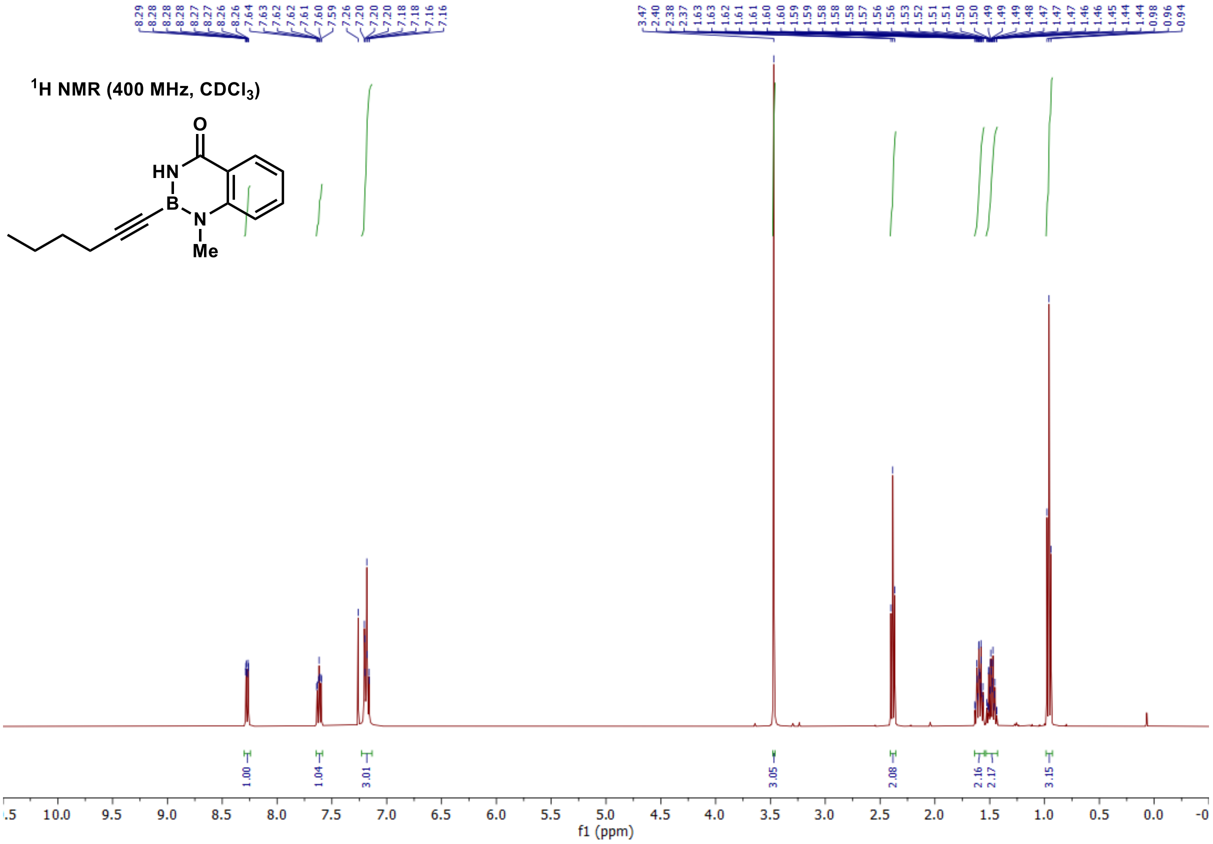
^{1}H NMR (400 MHz, CDCl$_3$)
f1 (ppm)

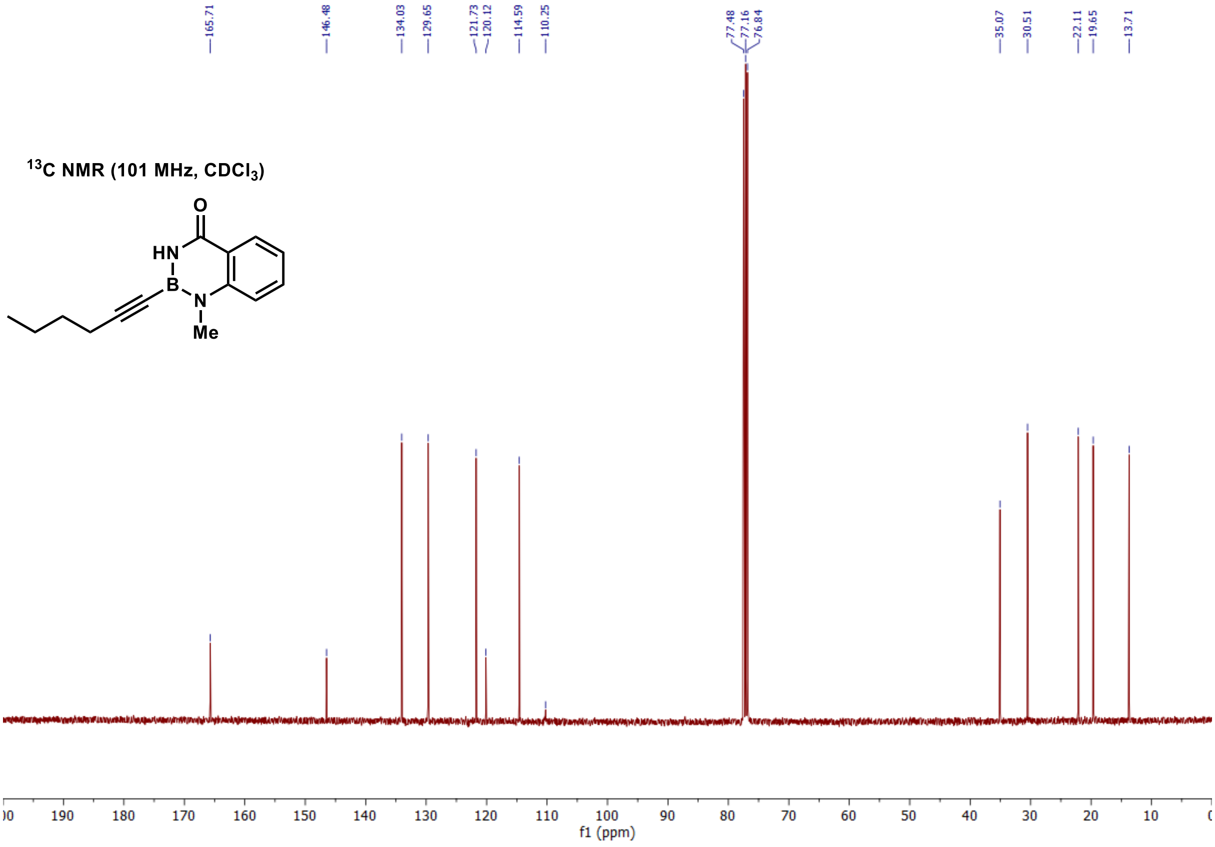
^{13}C NMR (101 MHz, CDCl$_3$)
f1 (ppm)

^{11}B NMR (128 MHz, $CDCl_3$)

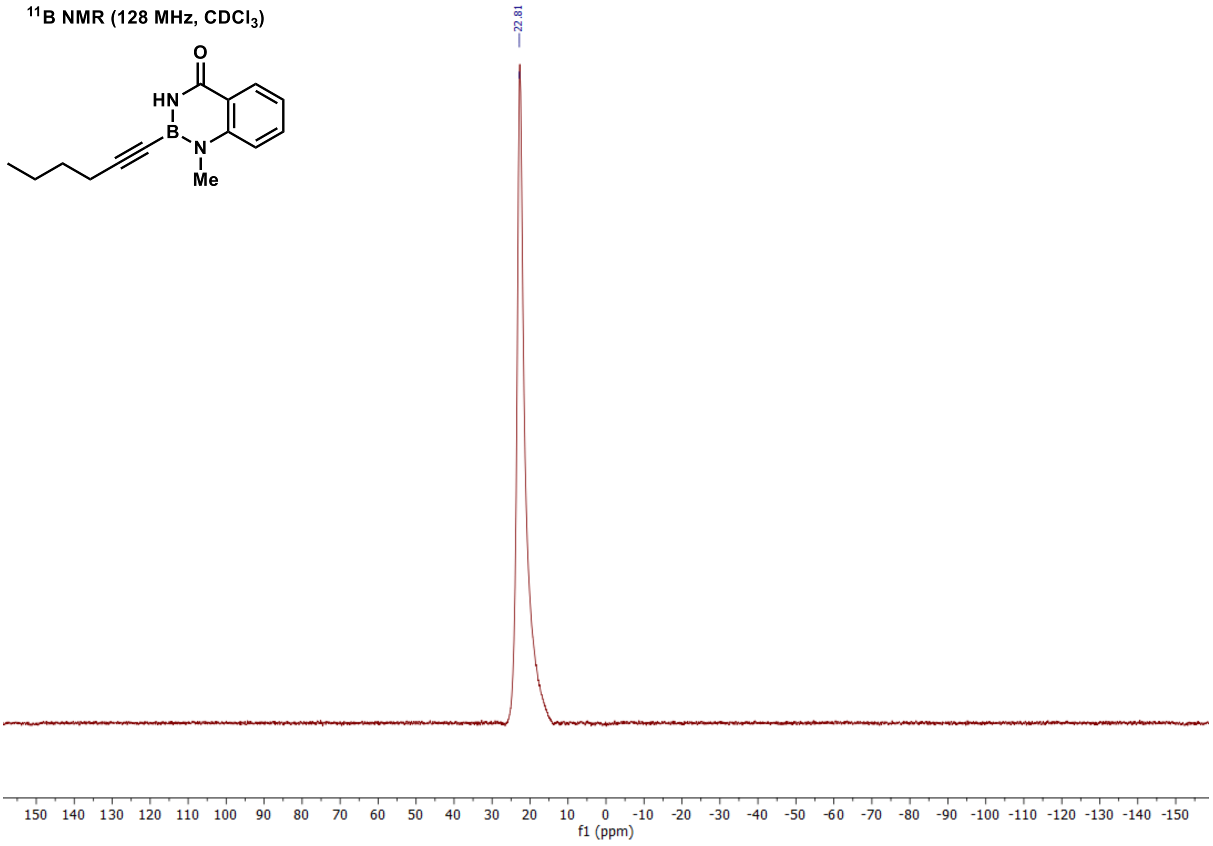

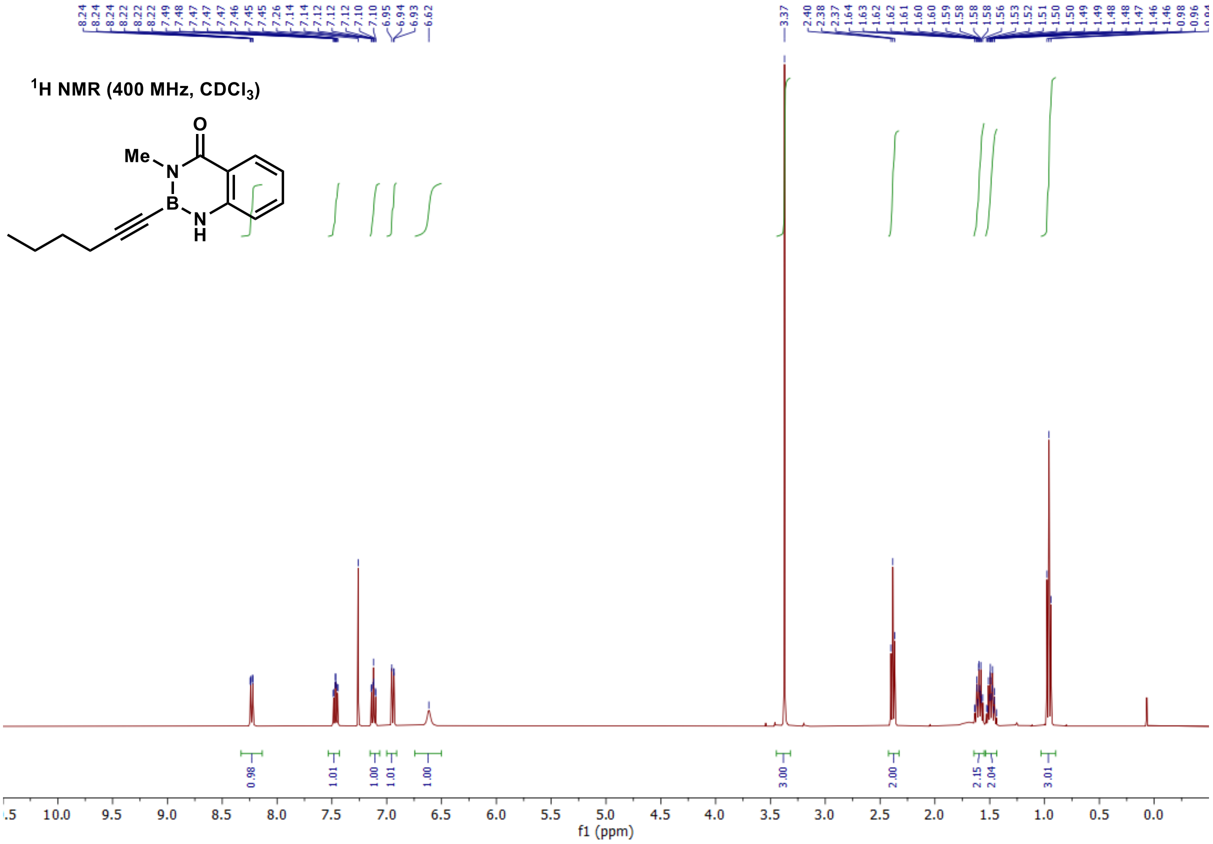

1H NMR (400 MHz, CDCl3)
O
Me
N
B
N
H
f1 (ppm)

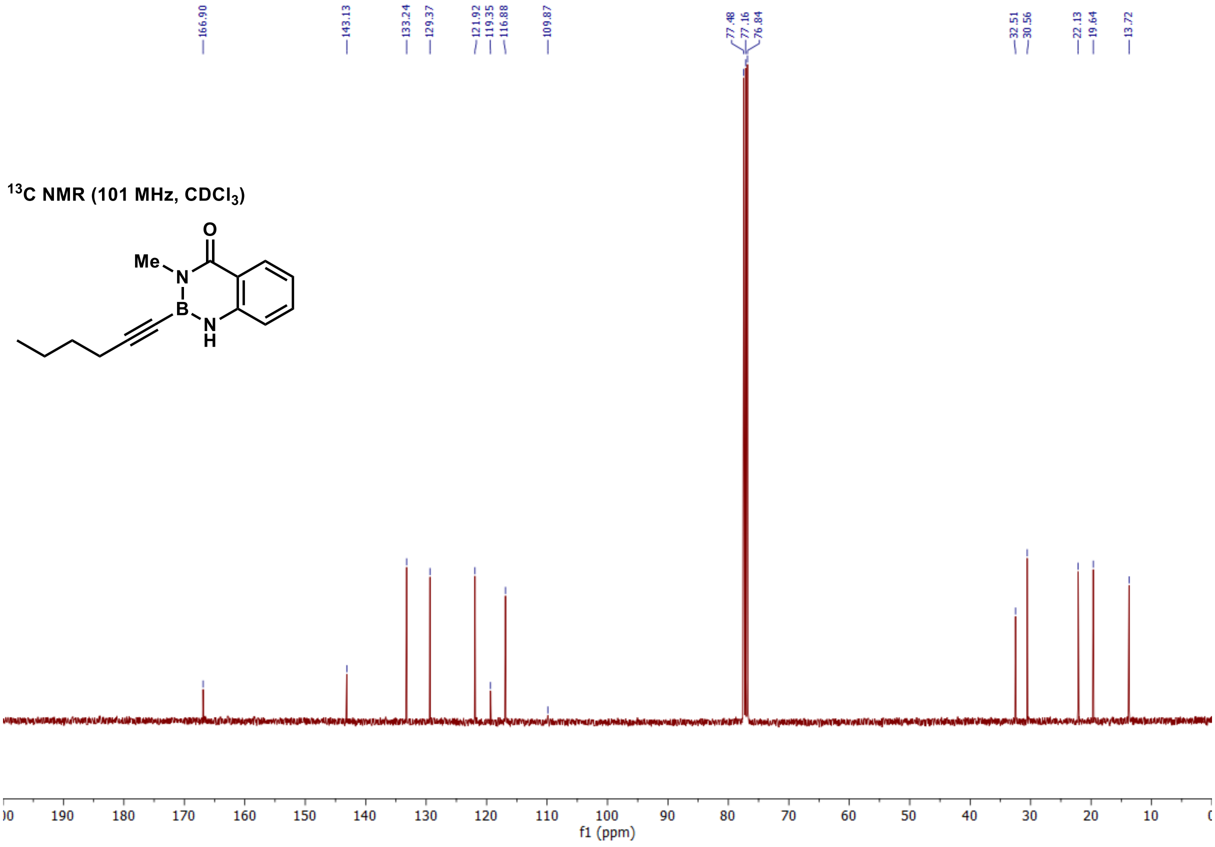

13C NMR (101 MHz, CDCl3)
O
Me
N
B
N
H
166.90
143.13
133.24
129.37
121.92
119.35
116.88
109.87
77.48
77.16
76.84
32.51
30.56
22.13
19.64
13.72
f1 (ppm)

^{11}B NMR (128 MHz, $CDCl_3$)

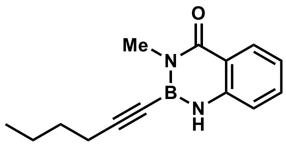

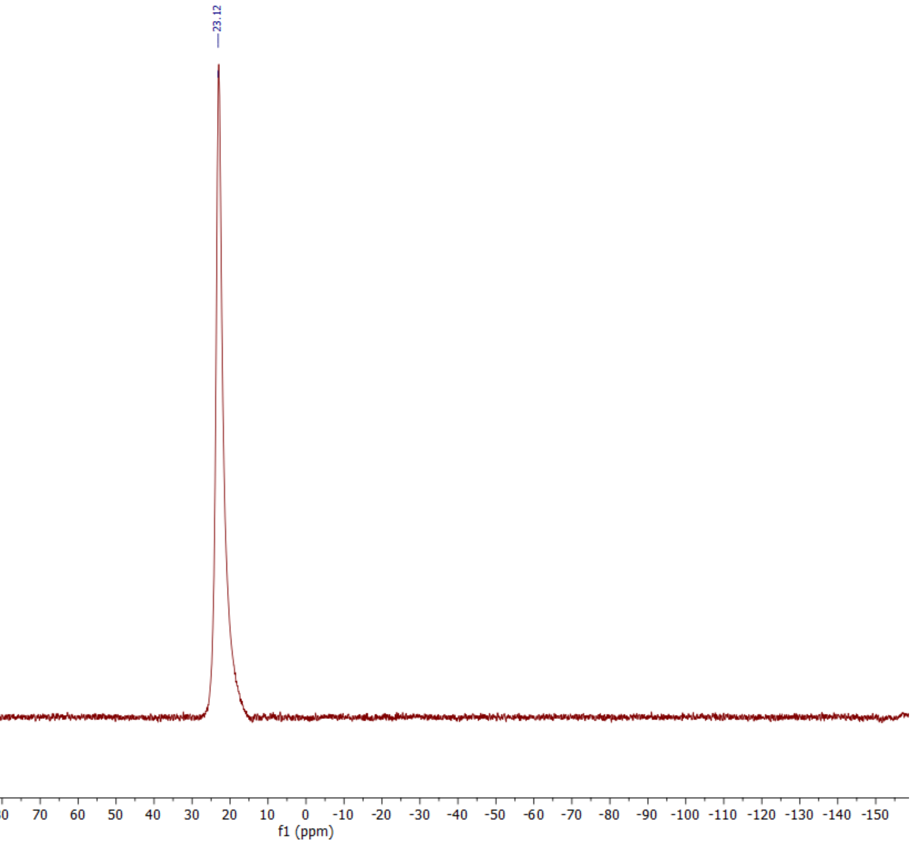

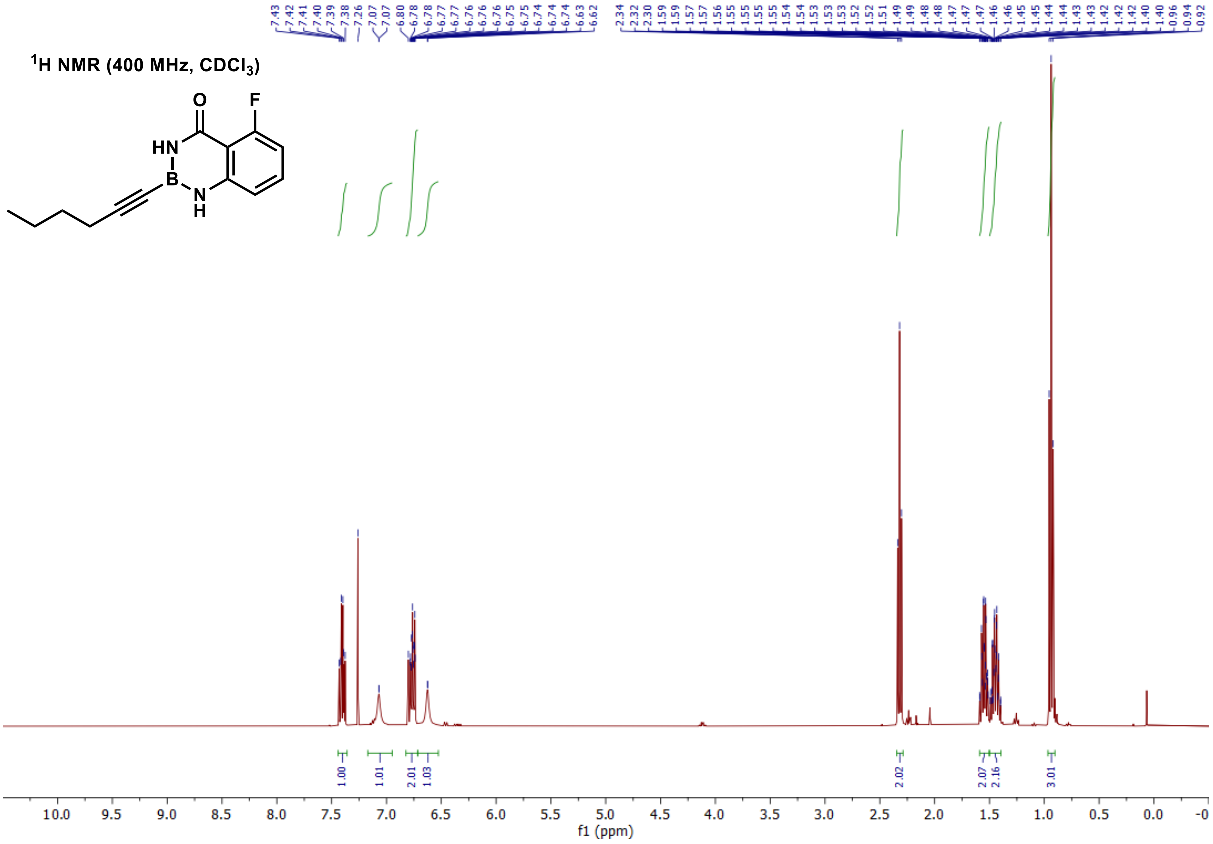
^{1}H NMR (400 MHz, CDCl$_3$)
f1 (ppm)

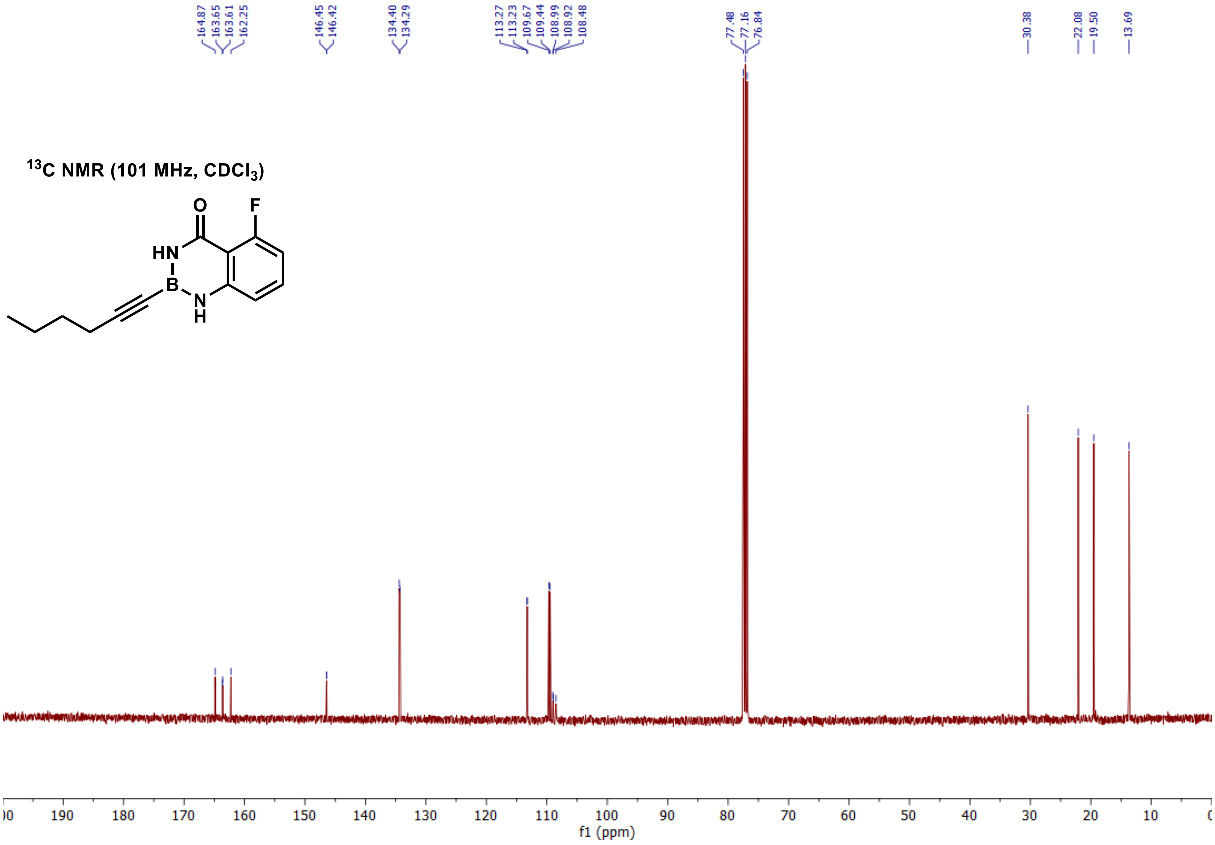
^{13}C NMR (101 MHz, CDCl$_3$)
f1 (ppm)

^{11}B NMR (128 MHz, $CDCl_3$)

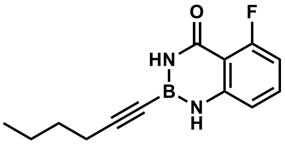

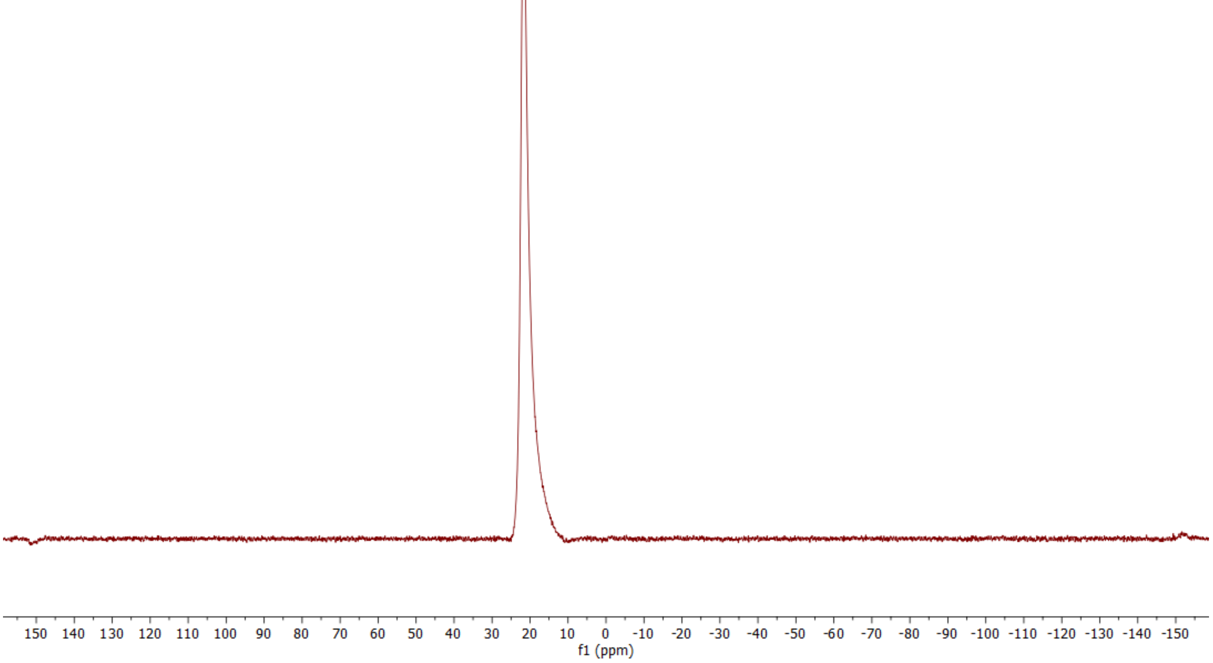

^{19}F NMR (282 MHz, $CDCl_3$)

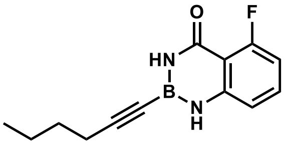

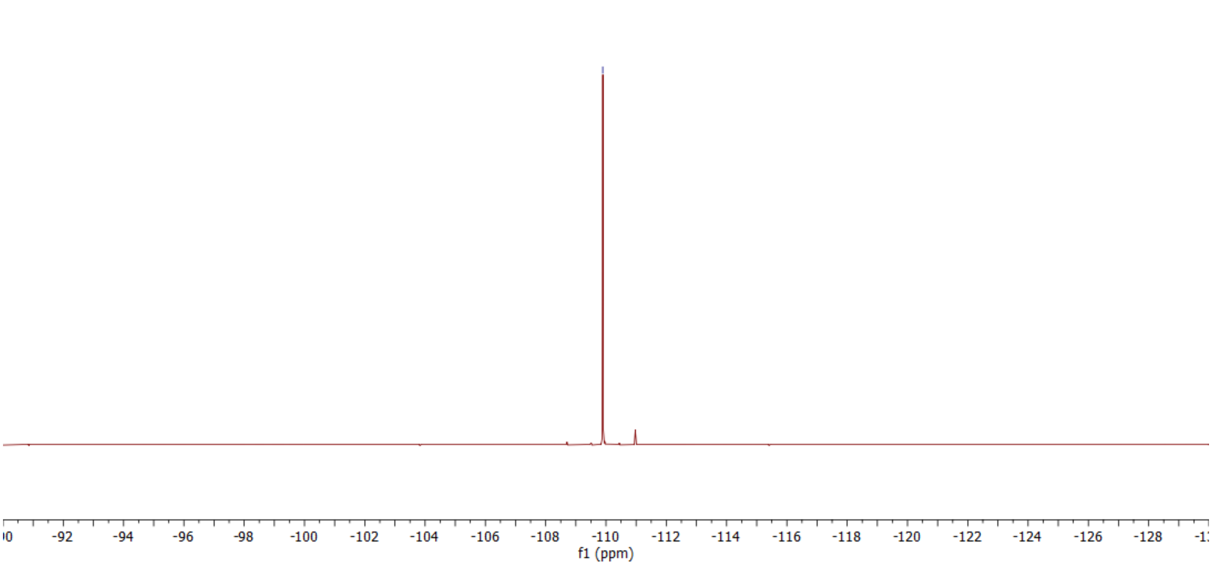

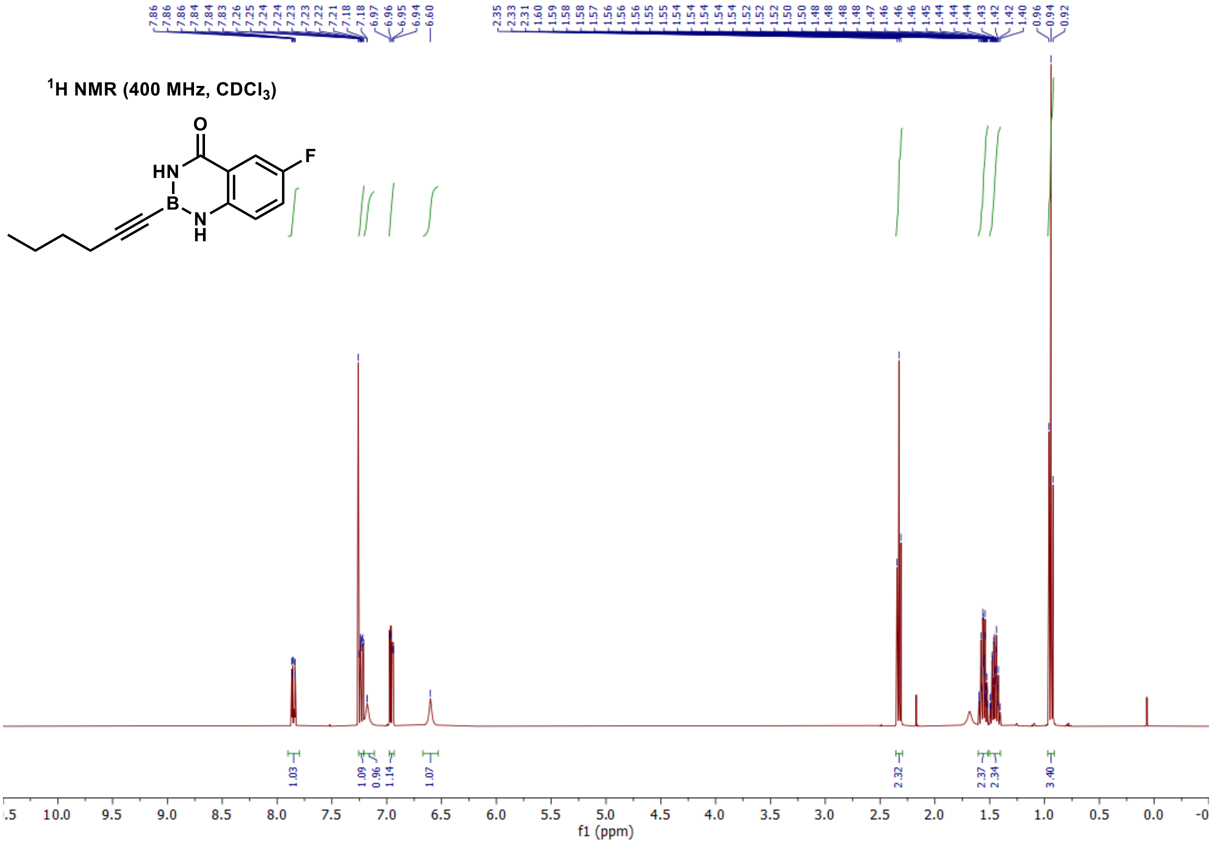
^{1}H NMR (400 MHz, $CDCl_3$)
O
HN
F
B
N
H
f1 (ppm)

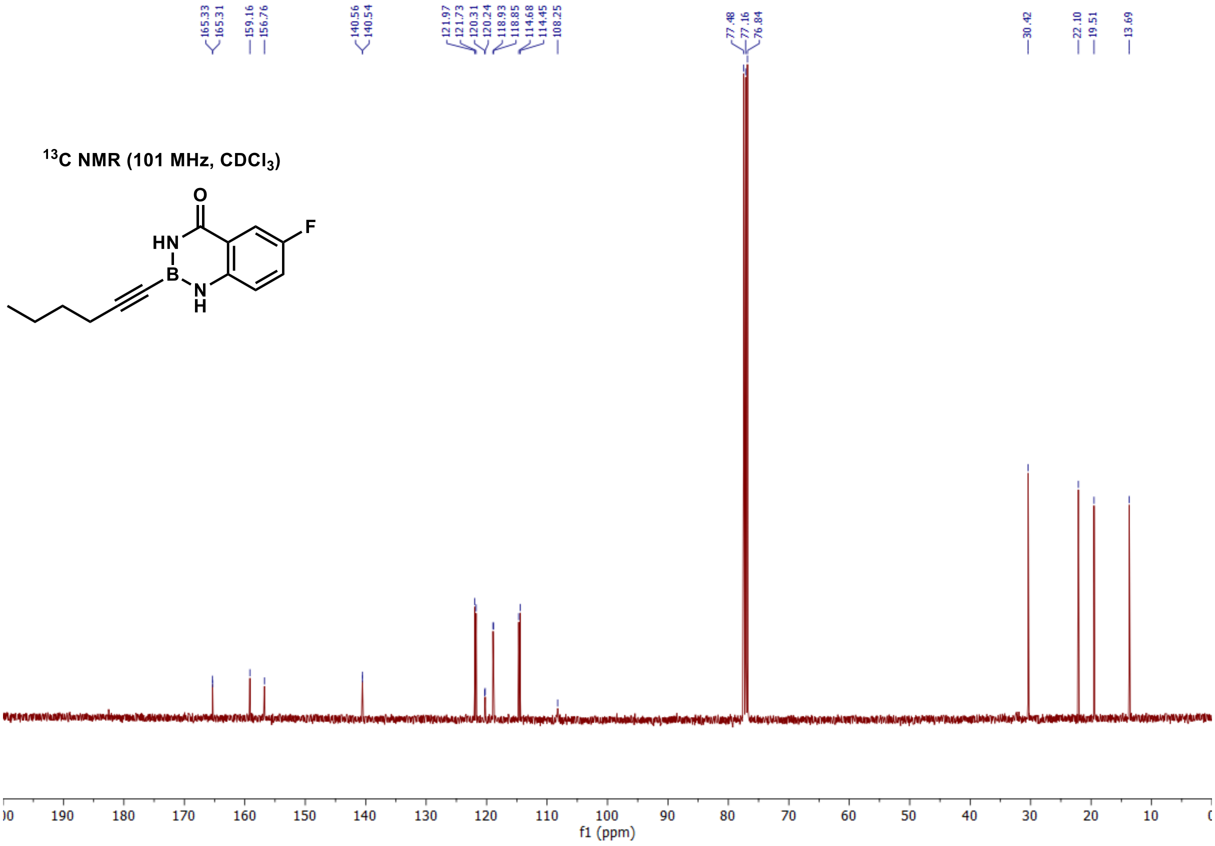
^{13}C NMR (101 MHz, $CDCl_3$)
O
HN
F
B
N
H
f1 (ppm)

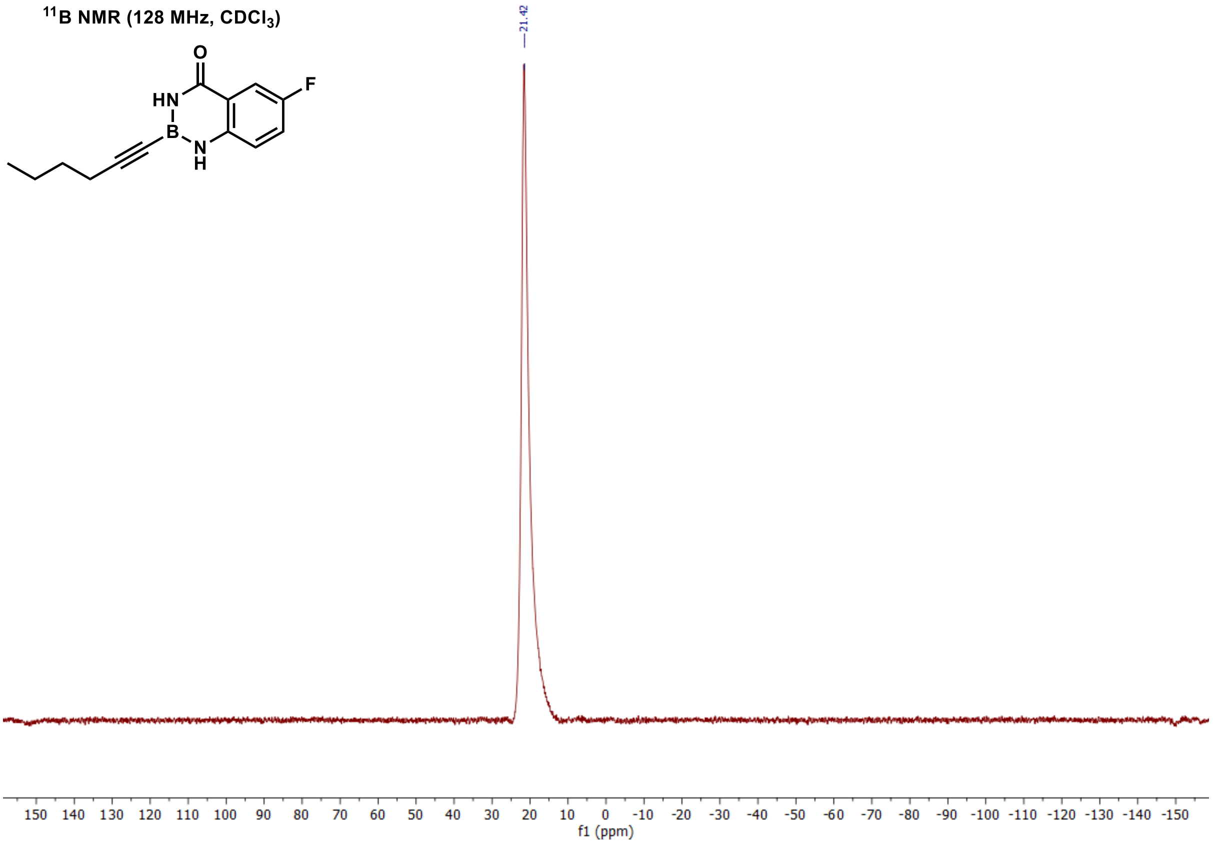
11B NMR (128 MHz, CDCl3)
21.42
f1 (ppm)

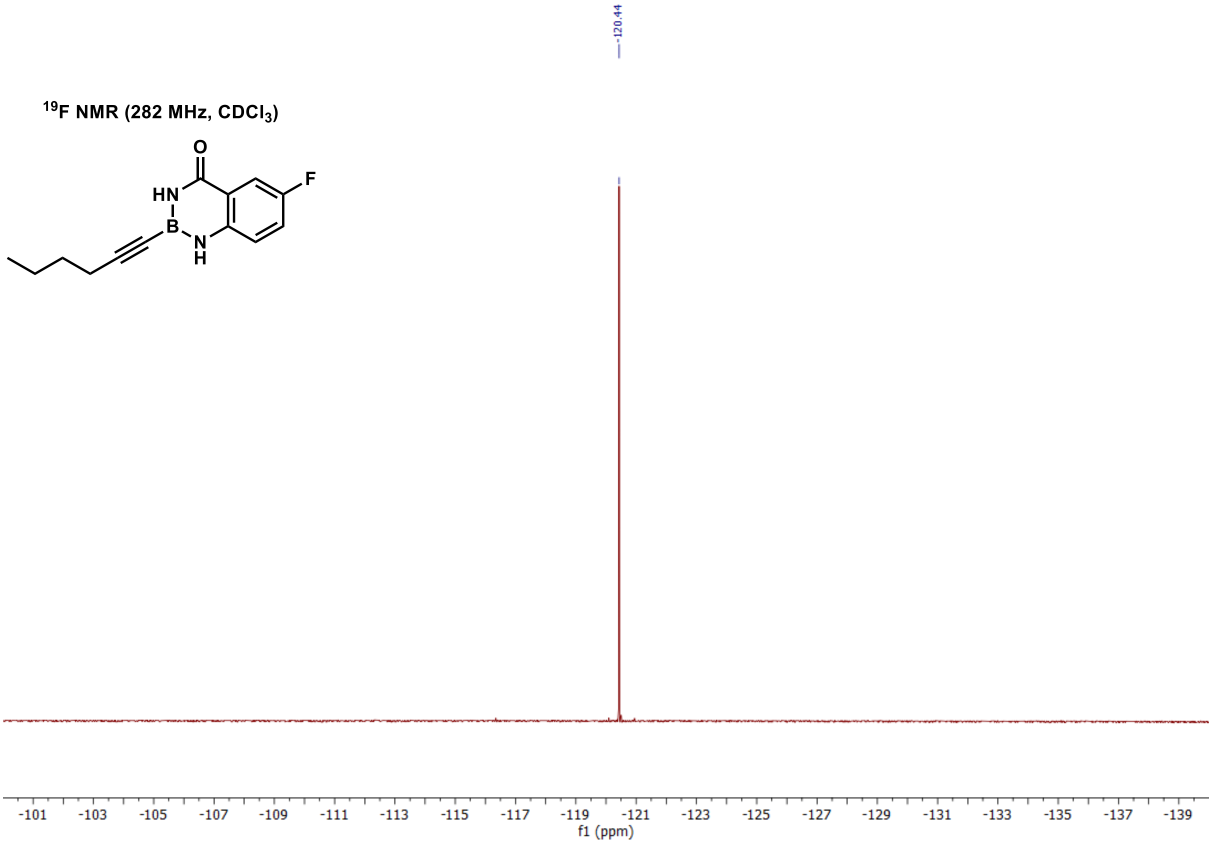
-120.44
19F NMR (282 MHz, CDCl3)
f1 (ppm)

^{1}H NMR (400 MHz, $CDCl_3$)

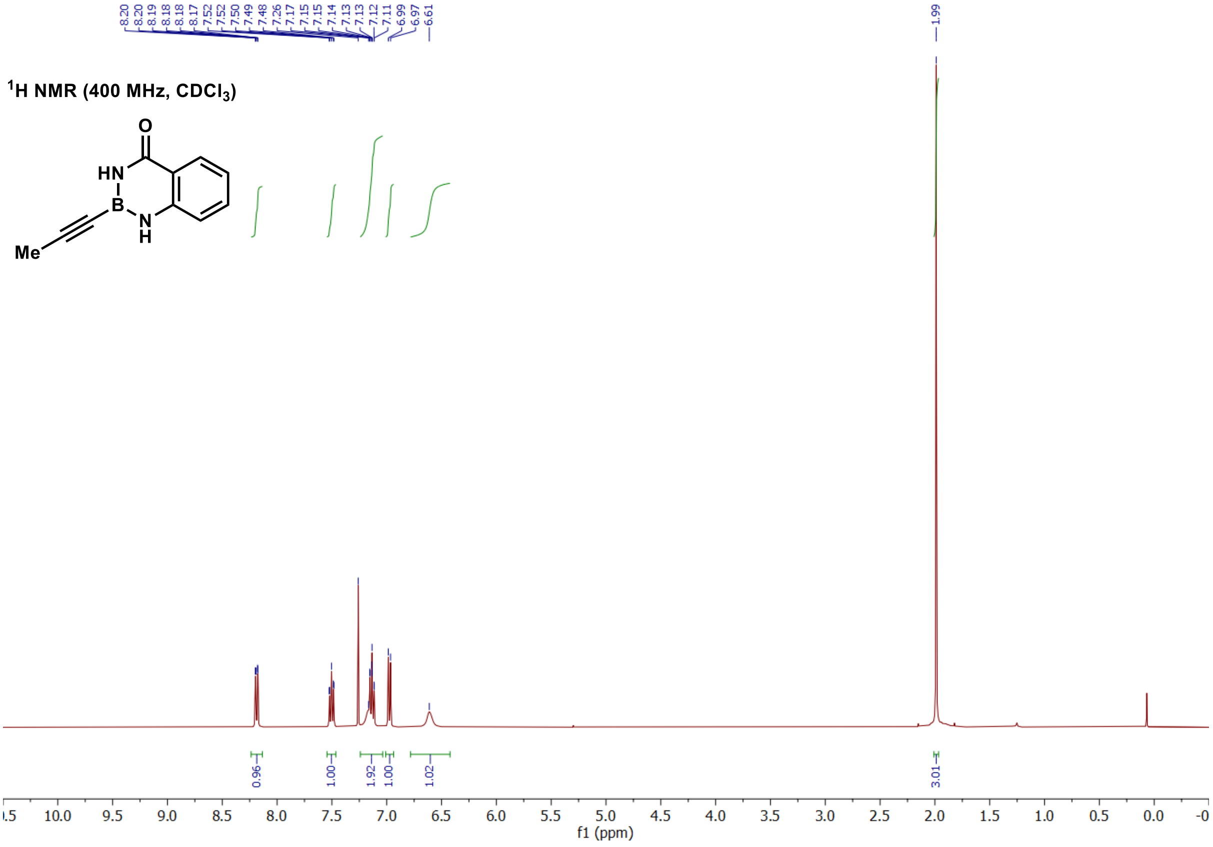

^{13}C NMR (101 MHz, $CDCl_3$)

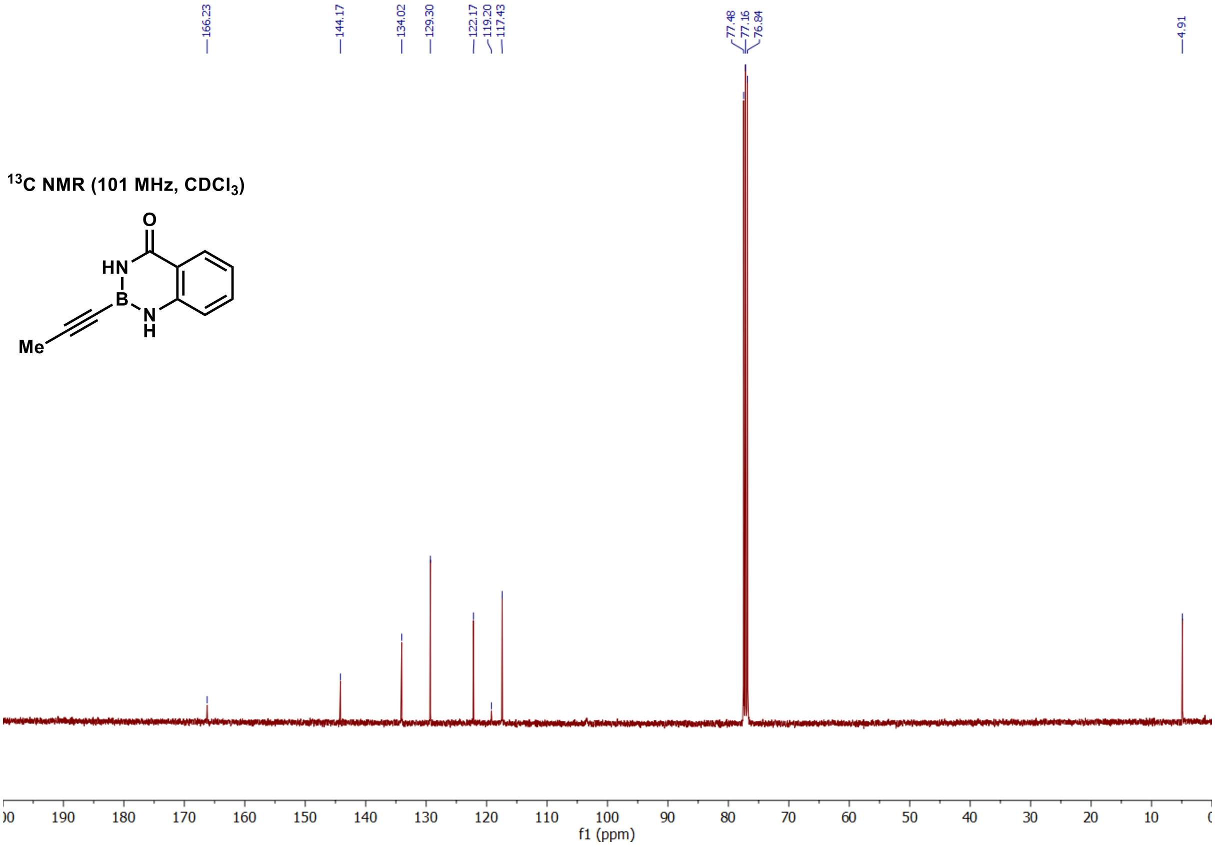

^{11}B NMR (128 MHz, $CDCl_3$)

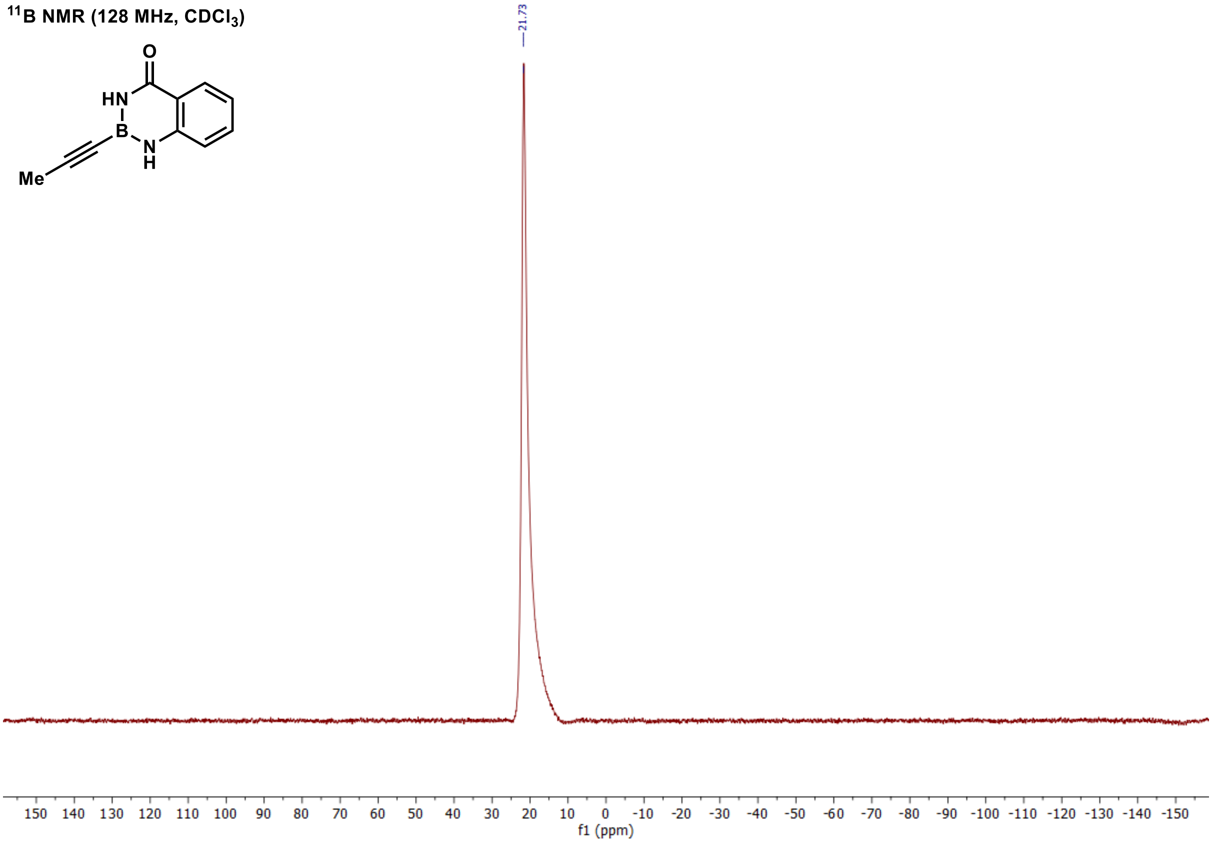

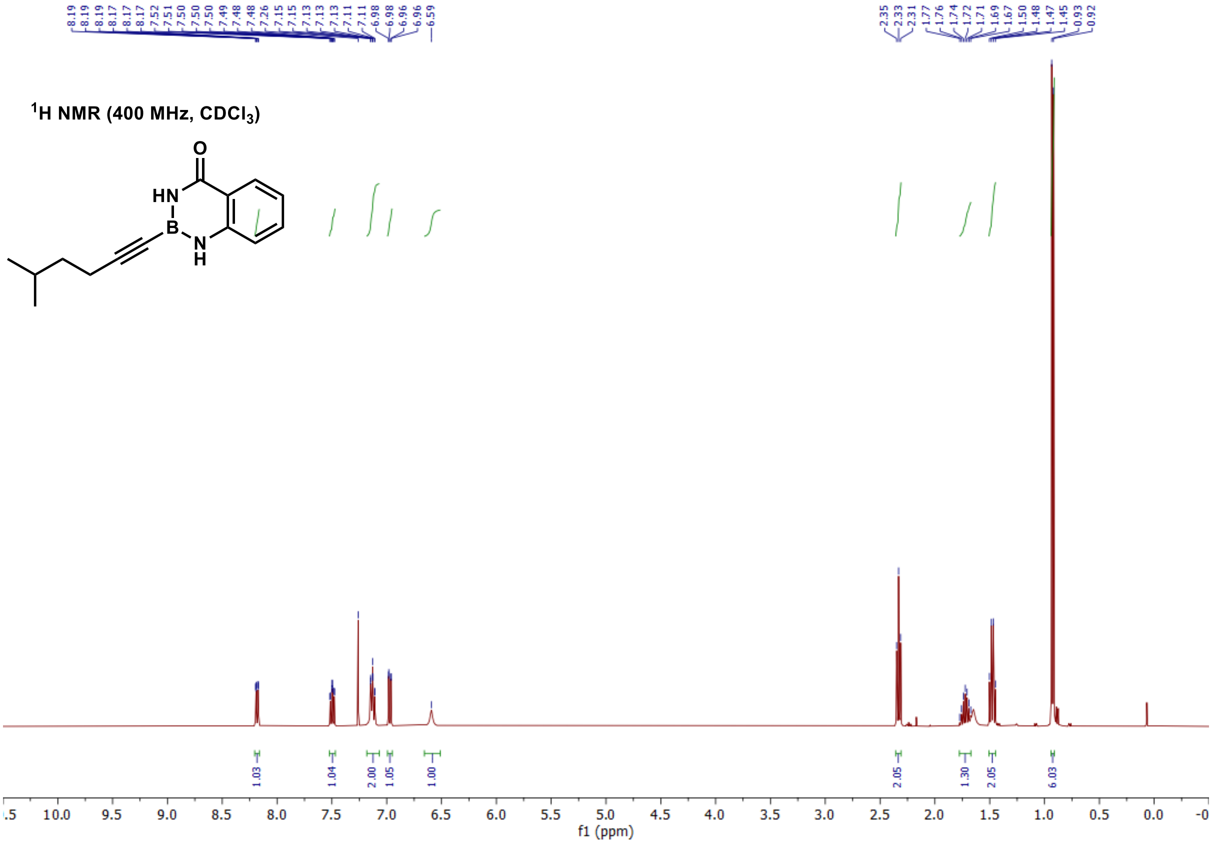
8.19 8.19 8.19 8.17 8.17 7.52 7.51 7.50 7.50 7.50 7.49 7.48 7.48 7.26 7.15 7.15 7.13 7.13 7.13 7.11 7.11 6.98 6.98 6.96 6.96 6.59
2.35 2.33 2.31 1.77 1.76 1.74 1.72 1.71 1.69 1.67 1.50 1.48 1.47 1.45 0.93 0.92
^{1}H NMR (400 MHz, $CDCl_3$)
O
HN
B
N
H
1.03
1.04
2.00
1.05
1.00
2.05
1.30
2.05
6.03
f1 (ppm)

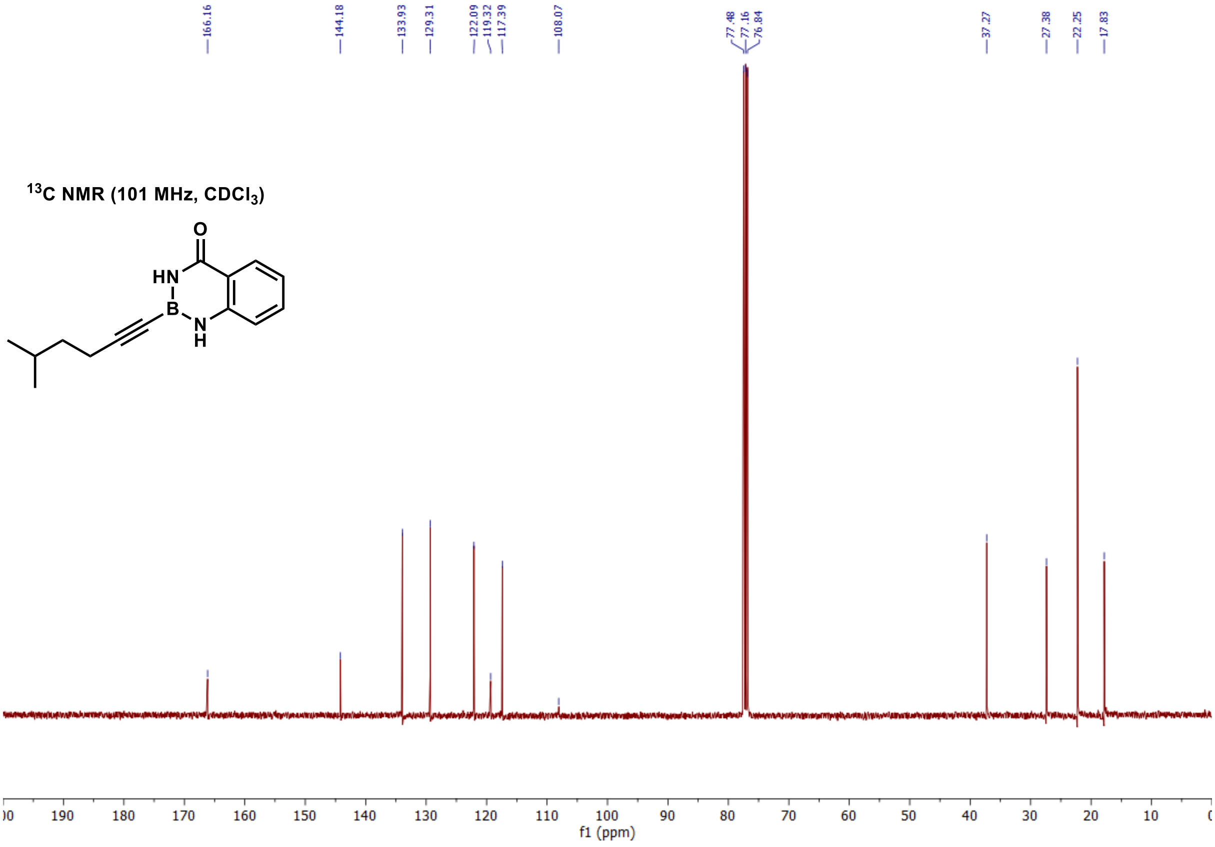
166.16
144.18
133.93
129.31
122.09
119.32
117.39
108.07
77.48 77.16 76.84
37.27
27.38
22.25
17.83
^{13}C NMR (101 MHz, $CDCl_3$)
O
HN
B
N
H
f1 (ppm)

^{11}B NMR (128 MHz, $CDCl_3$)

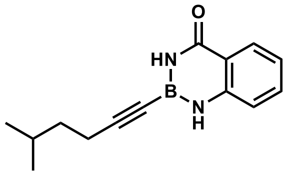

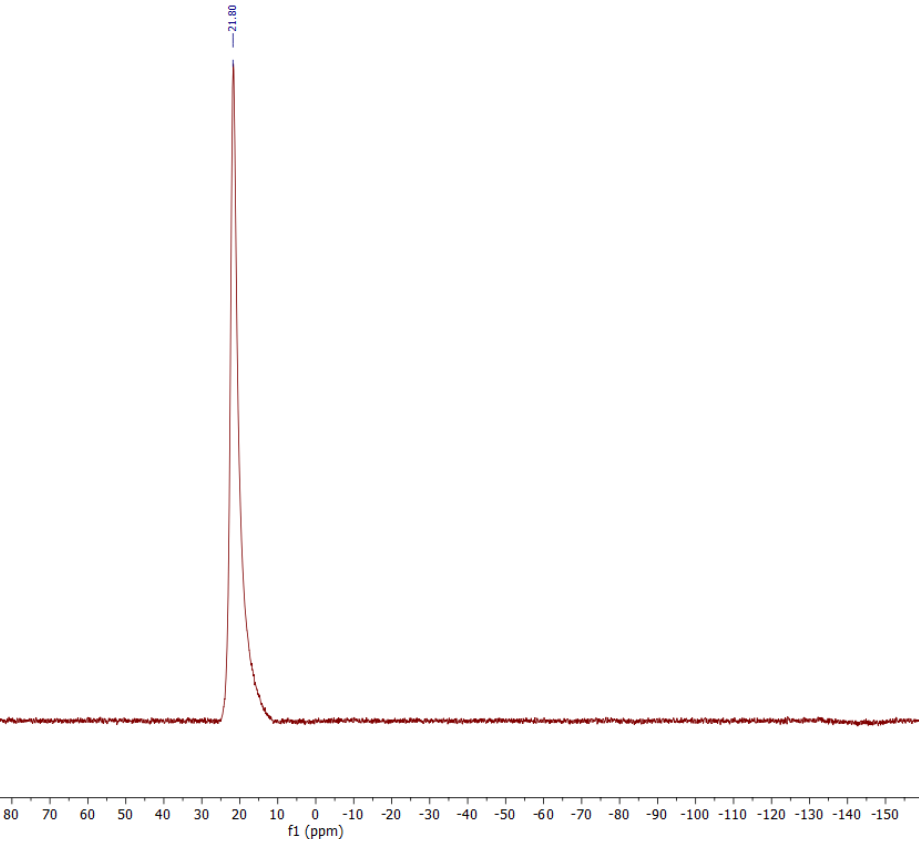

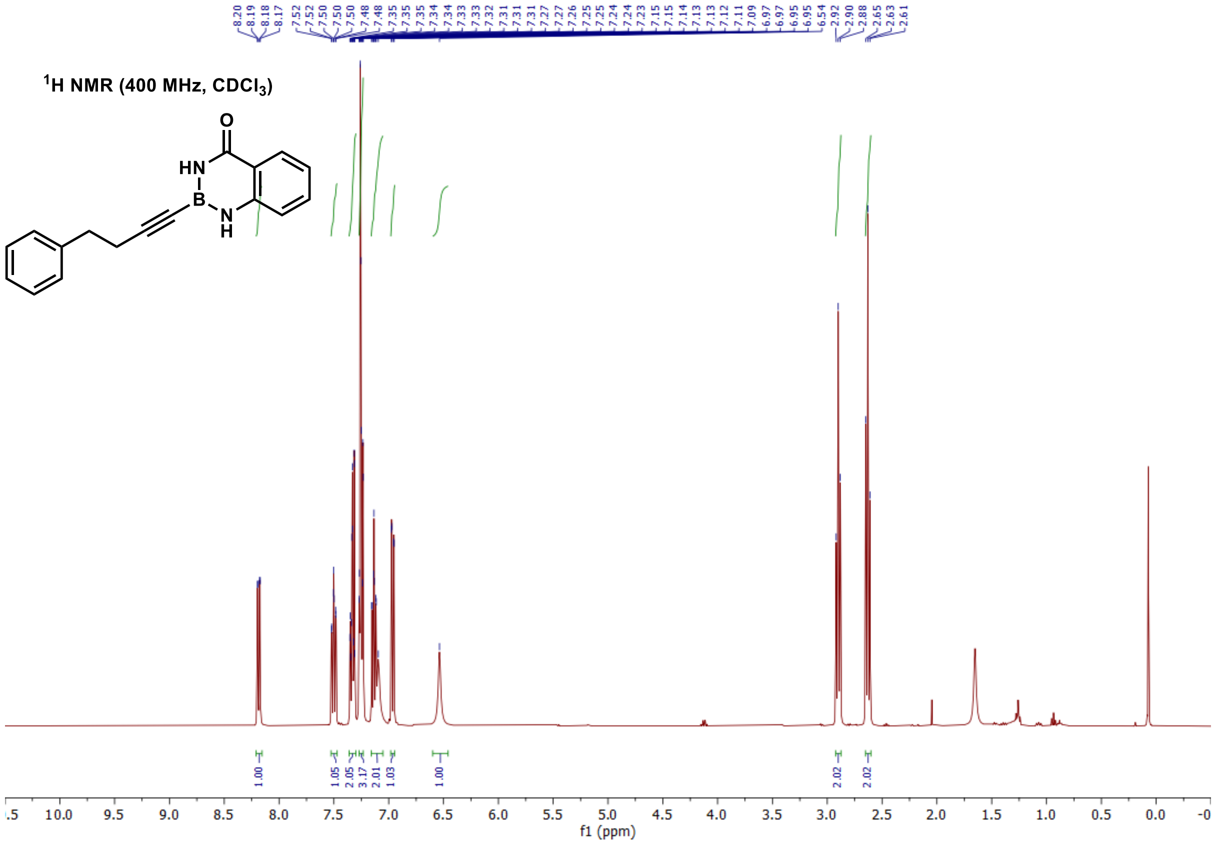
1H NMR (400 MHz, CDCl3)
f1 (ppm)

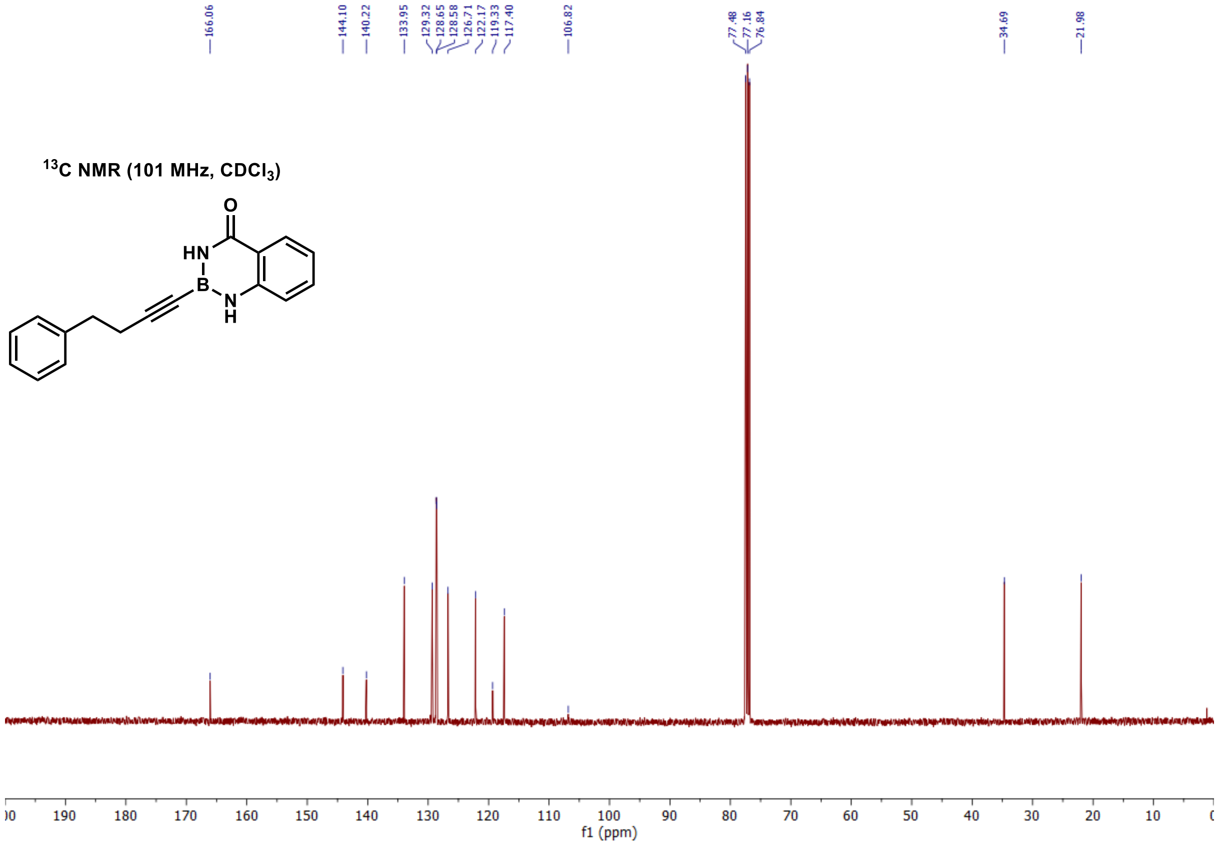
13C NMR (101 MHz, CDCl3)
f1 (ppm)

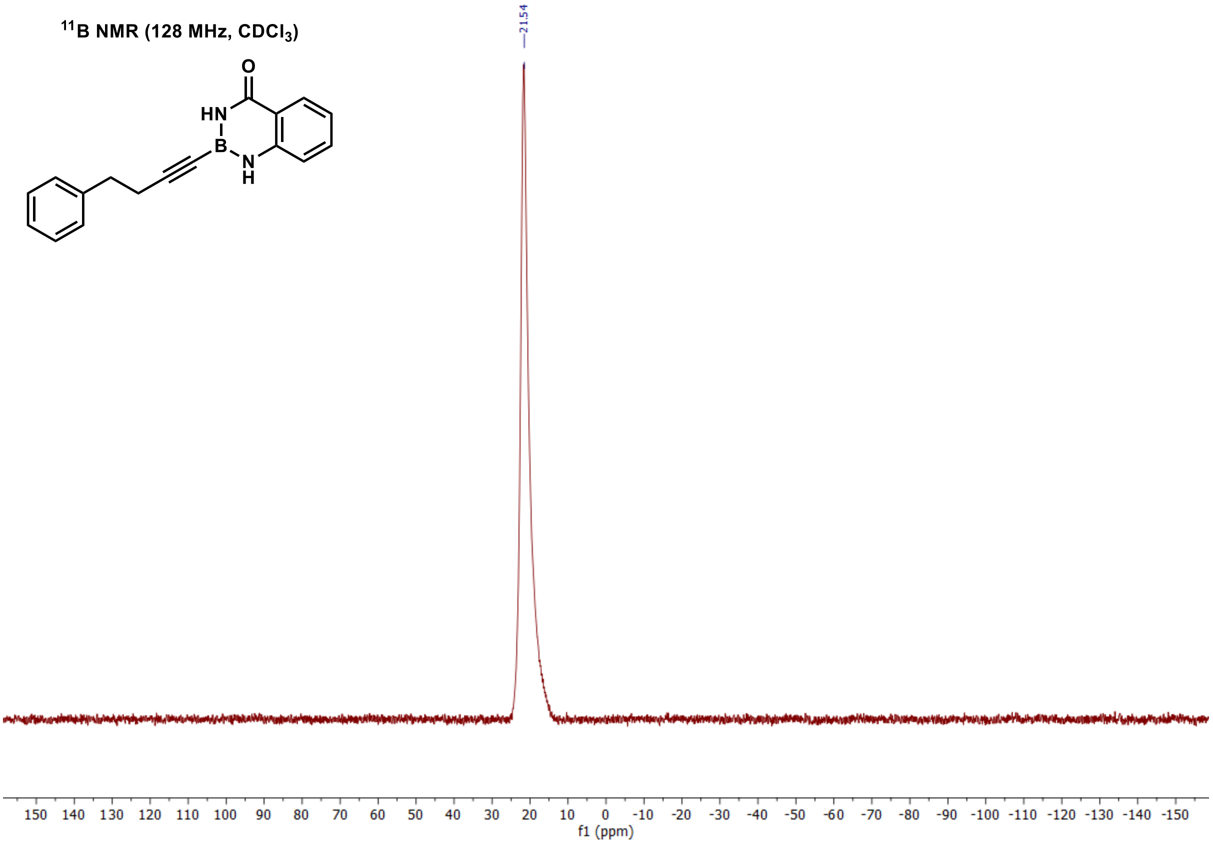

11B NMR (128 MHz, CDCl3)
O
HN
B
N
H
21.54
150 140 130 120 110 100 90 80 70 60 50 40 30 20 10 0 -10 -20 -30 -40 -50 -60 -70 -80 -90 -100 -110 -120 -130 -140 -150
f1 (ppm)

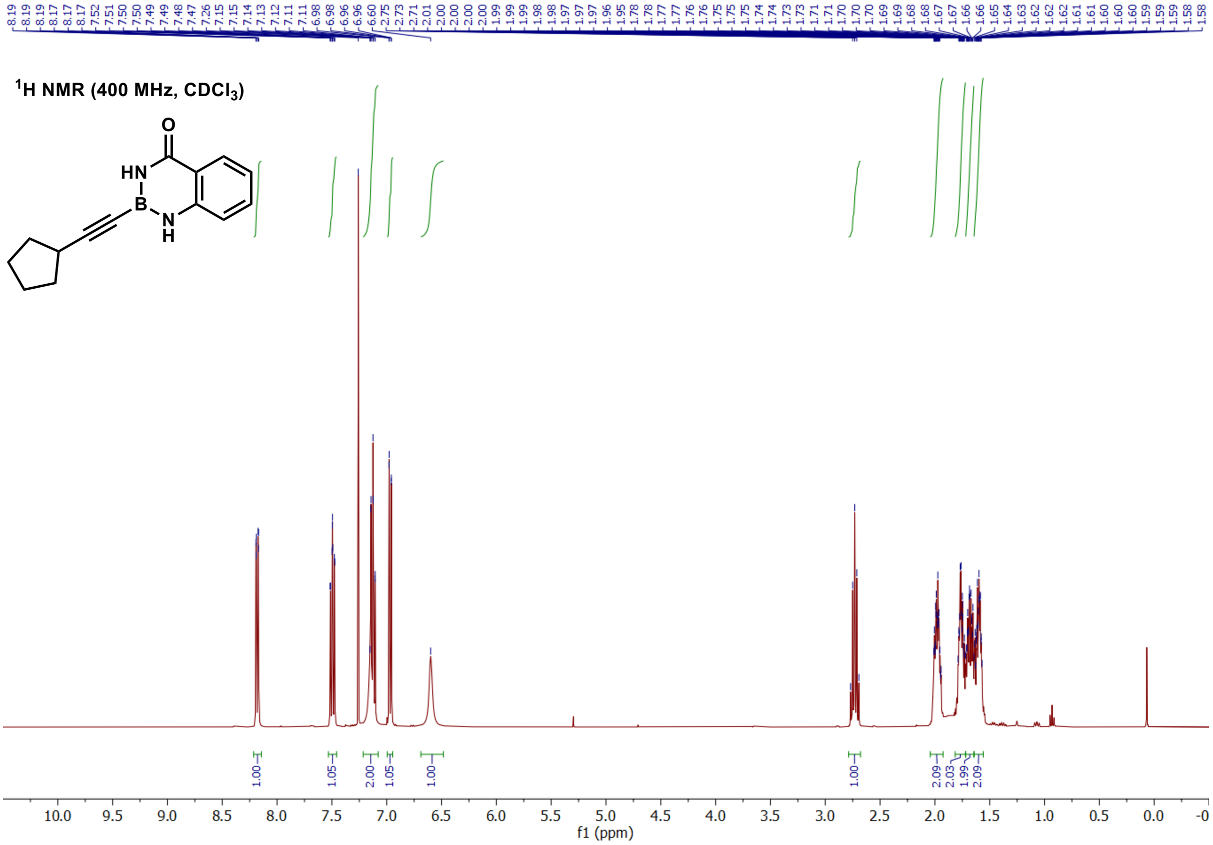
1H NMR (400 MHz, CDCl3)
O
HN
B
N
H
1.00
1.05
2.00
1.05
1.00
1.00
2.09
2.03
1.99
2.09
f1 (ppm)

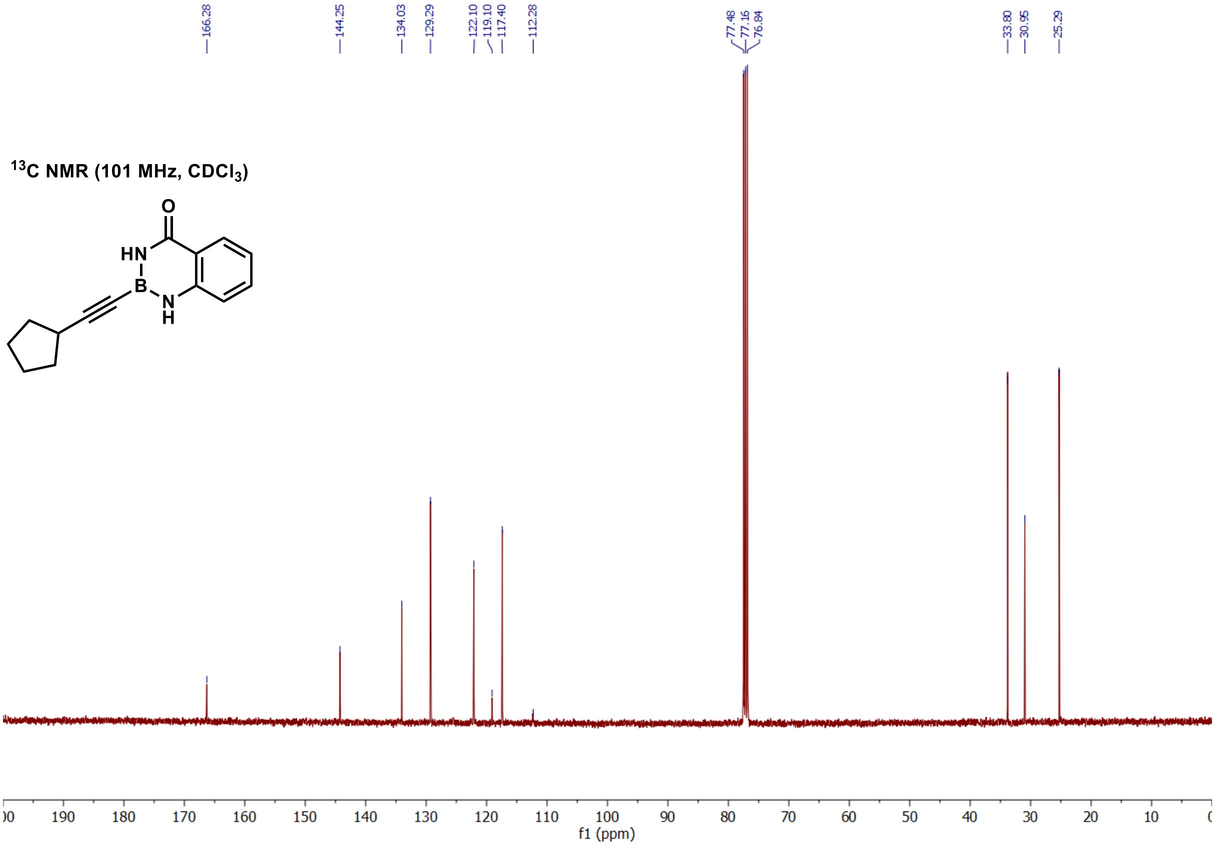
166.28
144.25
134.03
129.29
122.10
119.10
117.40
112.28
77.48
77.16
76.84
33.80
30.95
25.29
13C NMR (101 MHz, CDCl3)
O
HN
B
N
H
f1 (ppm)

^{11}B NMR (128 MHz, $CDCl_3$)

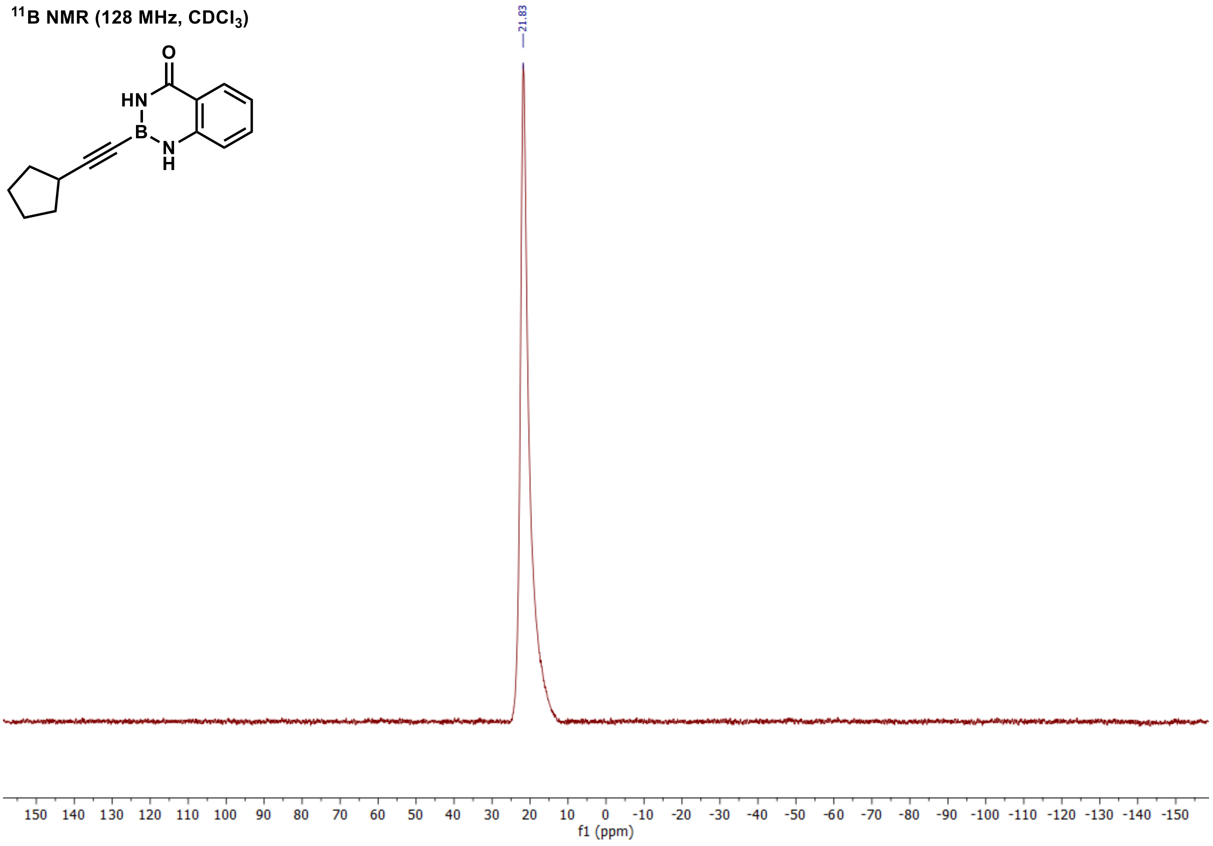

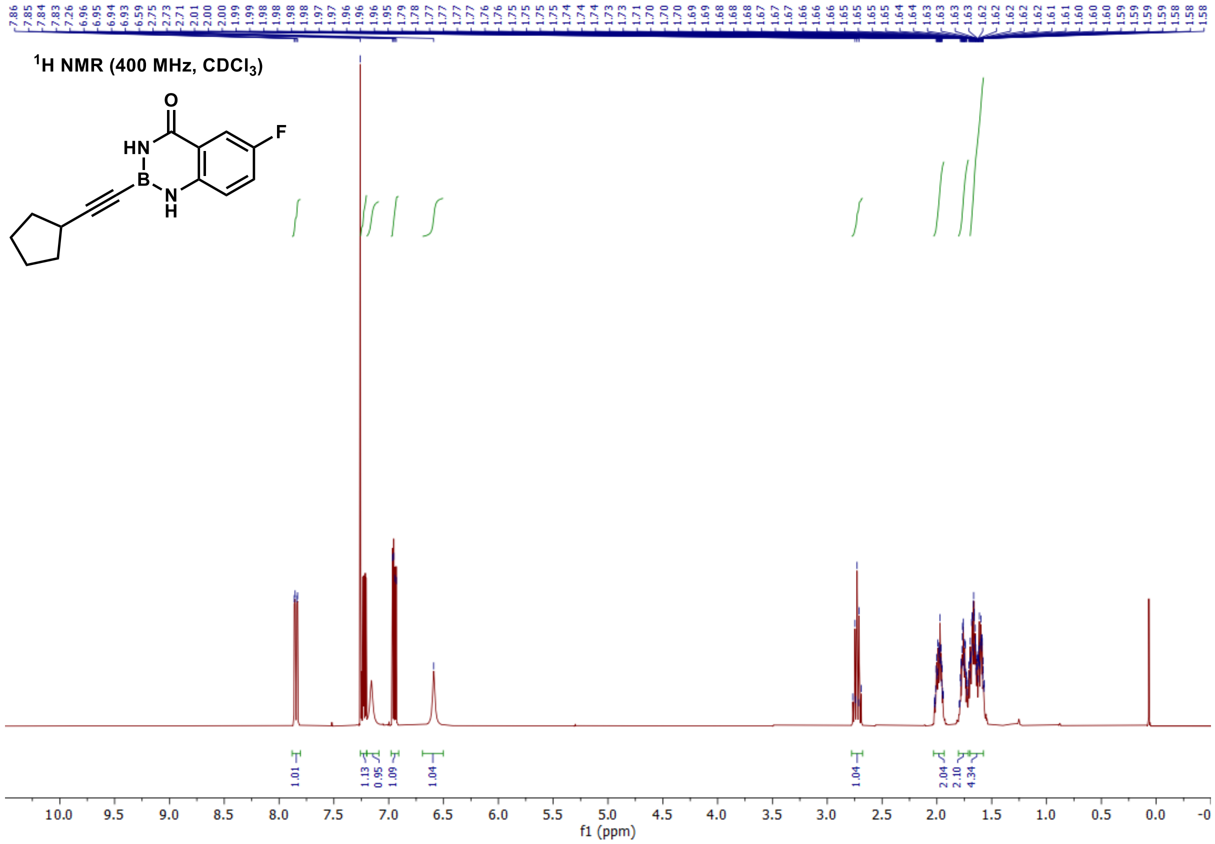

7.86 7.85 7.84 7.83 7.26 6.96 6.95 6.94 6.93 6.59 2.75 2.73 2.71 2.01 2.00 1.99 1.99 1.98 1.98 1.98 1.98 1.97 1.97 1.96 1.96 1.96 1.95 1.79 1.78 1.77 1.77 1.77 1.77 1.76 1.76 1.75 1.75 1.75 1.75 1.74 1.74 1.73 1.73 1.71 1.70 1.70 1.69 1.69 1.68 1.68 1.68 1.67 1.67 1.67 1.66 1.66 1.66 1.65 1.65 1.65 1.65 1.64 1.64 1.63 1.63 1.63 1.63 1.62 1.62 1.62 1.62 1.62 1.61 1.61 1.60 1.60 1.60 1.59 1.59 1.59 1.59 1.58 1.58 1.58
^{1}H NMR (400 MHz, CDCl$_3$)
O
HN
B
N
H
F
1.01
1.13
0.95
1.09
1.04
1.04
2.04
2.10
4.34
10.0 9.5 9.0 8.5 8.0 7.5 7.0 6.5 6.0 5.5 5.0 4.5 4.0 3.5 3.0 2.5 2.0 1.5 1.0 0.5 0.0
f1 (ppm)

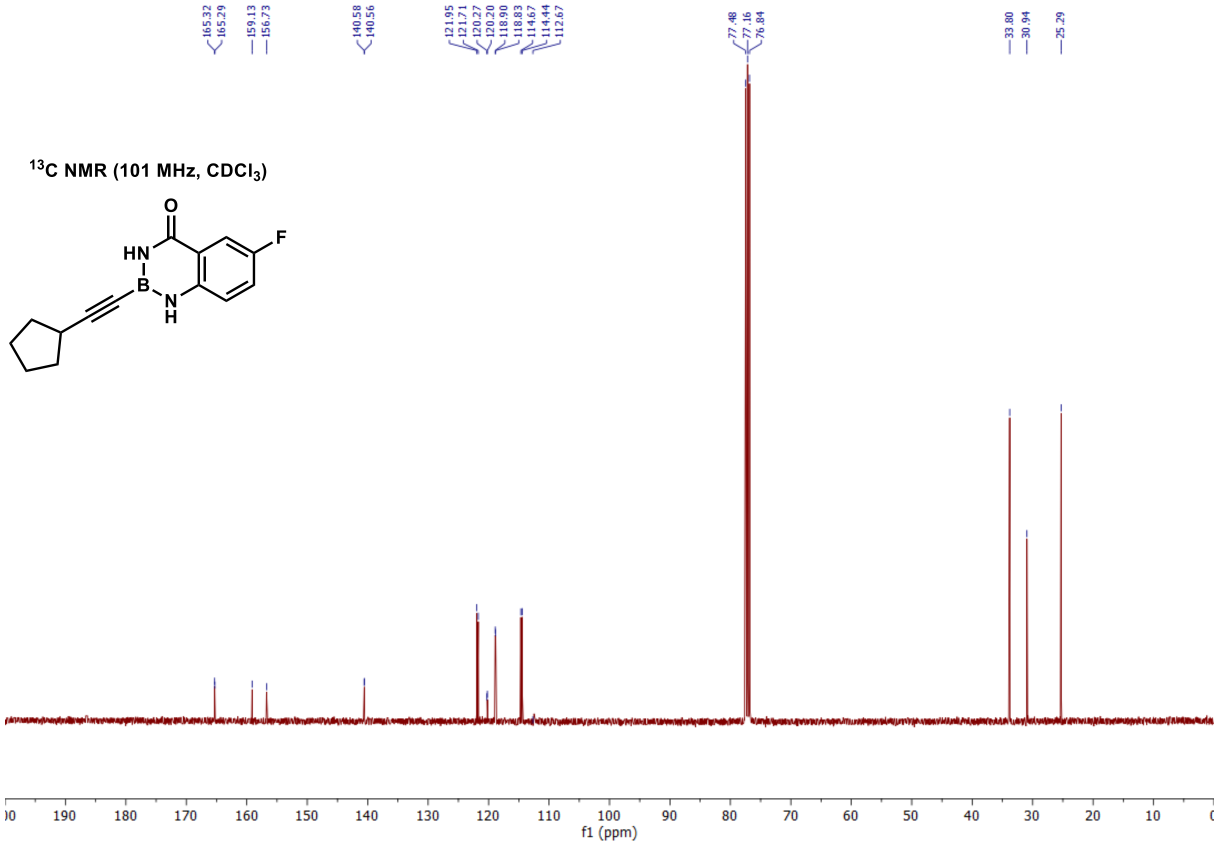

165.32 165.29 159.13 156.73 140.58 140.56 121.95 121.71 120.27 120.20 118.90 118.83 114.67 114.44 112.67 77.48 77.16 76.84 33.80 30.94 25.29
^{13}C NMR (101 MHz, CDCl$_3$)
O
HN
B
N
H
F
190 180 170 160 150 140 130 120 110 100 90 80 70 60 50 40 30 20 10
f1 (ppm)

^{11}B NMR (128 MHz, CDCl$_3$)

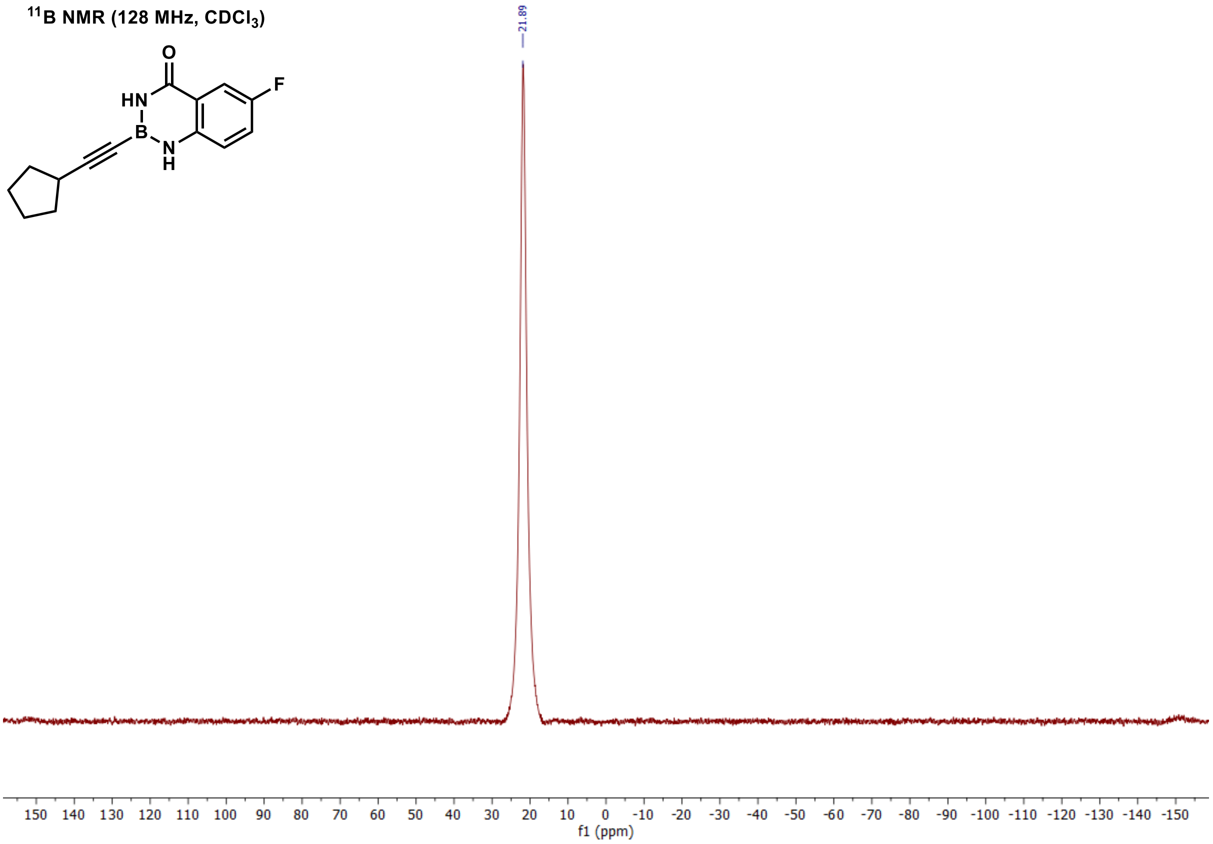

^{19}F NMR (282 MHz, CDCl$_3$)

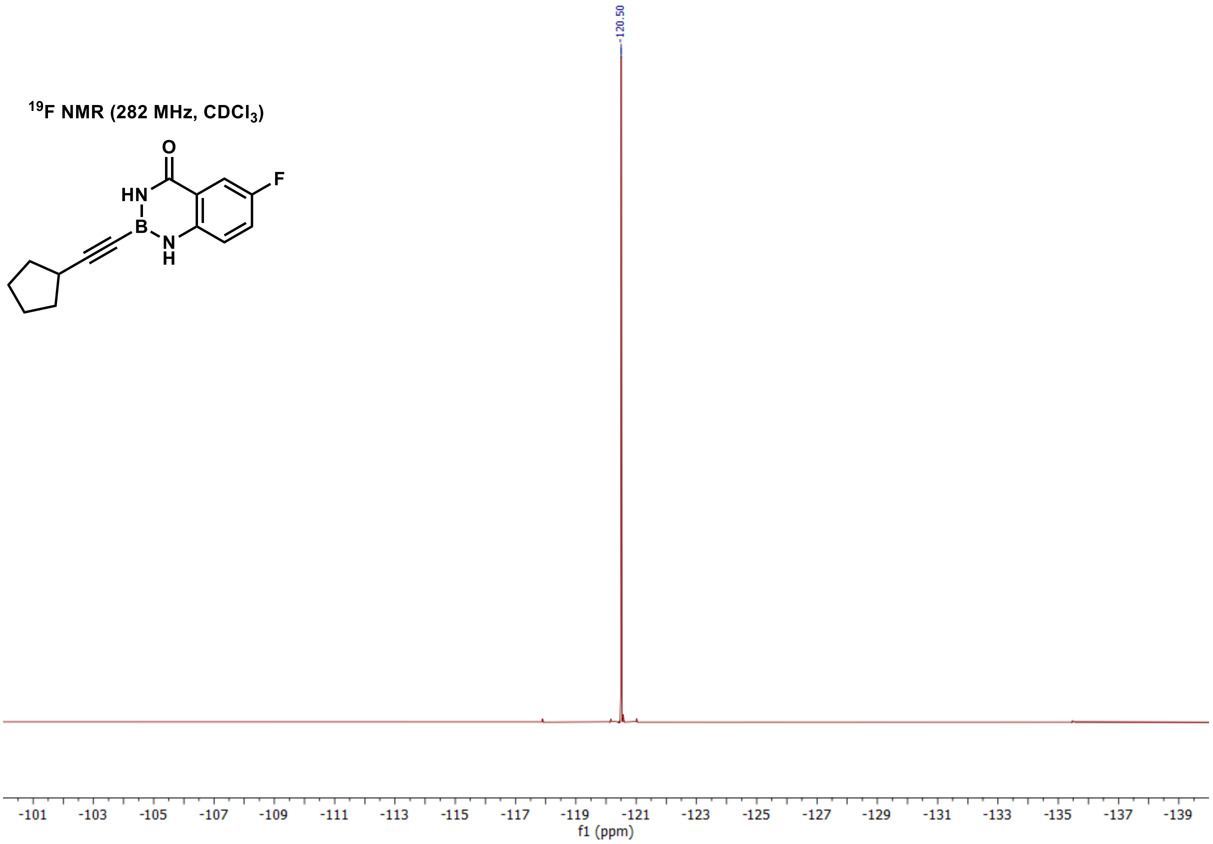

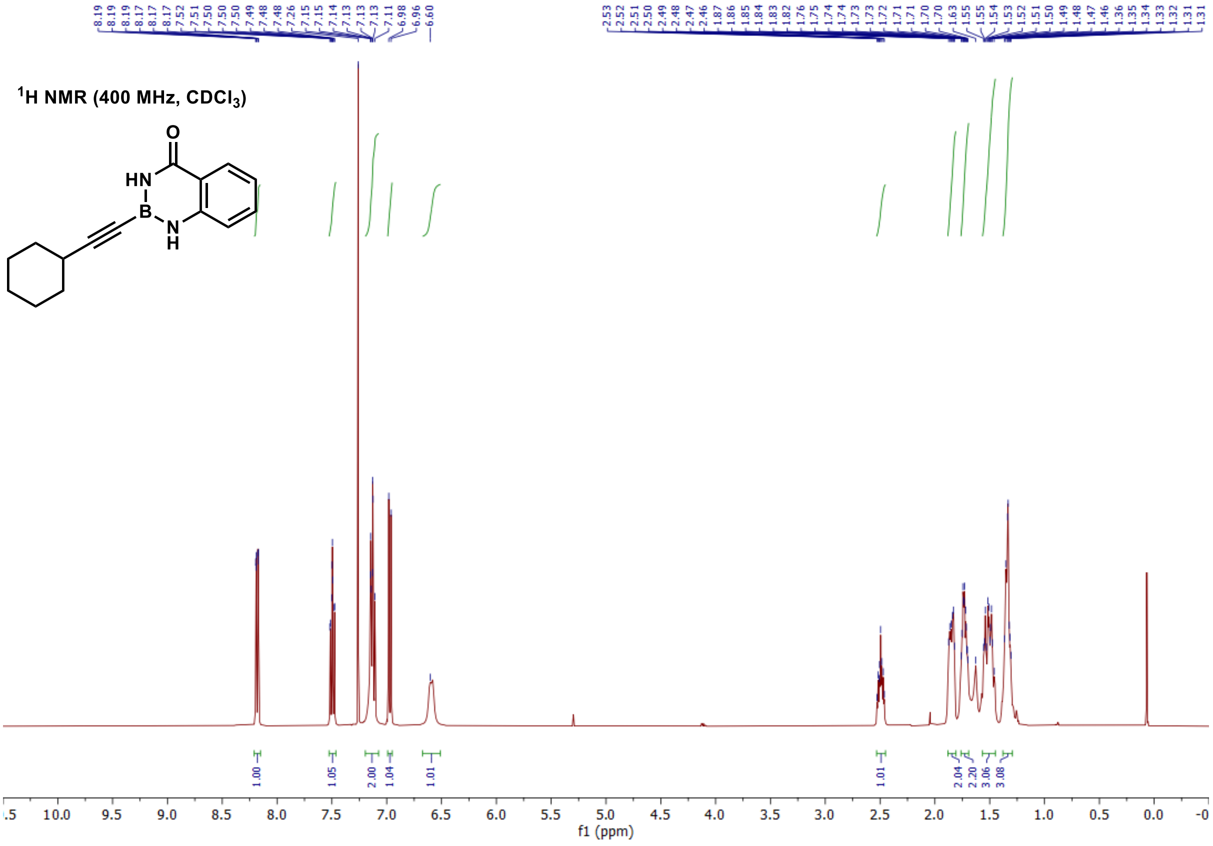

1H NMR (400 MHz, CDCl3)
O
HN
B
N
H
f1 (ppm)

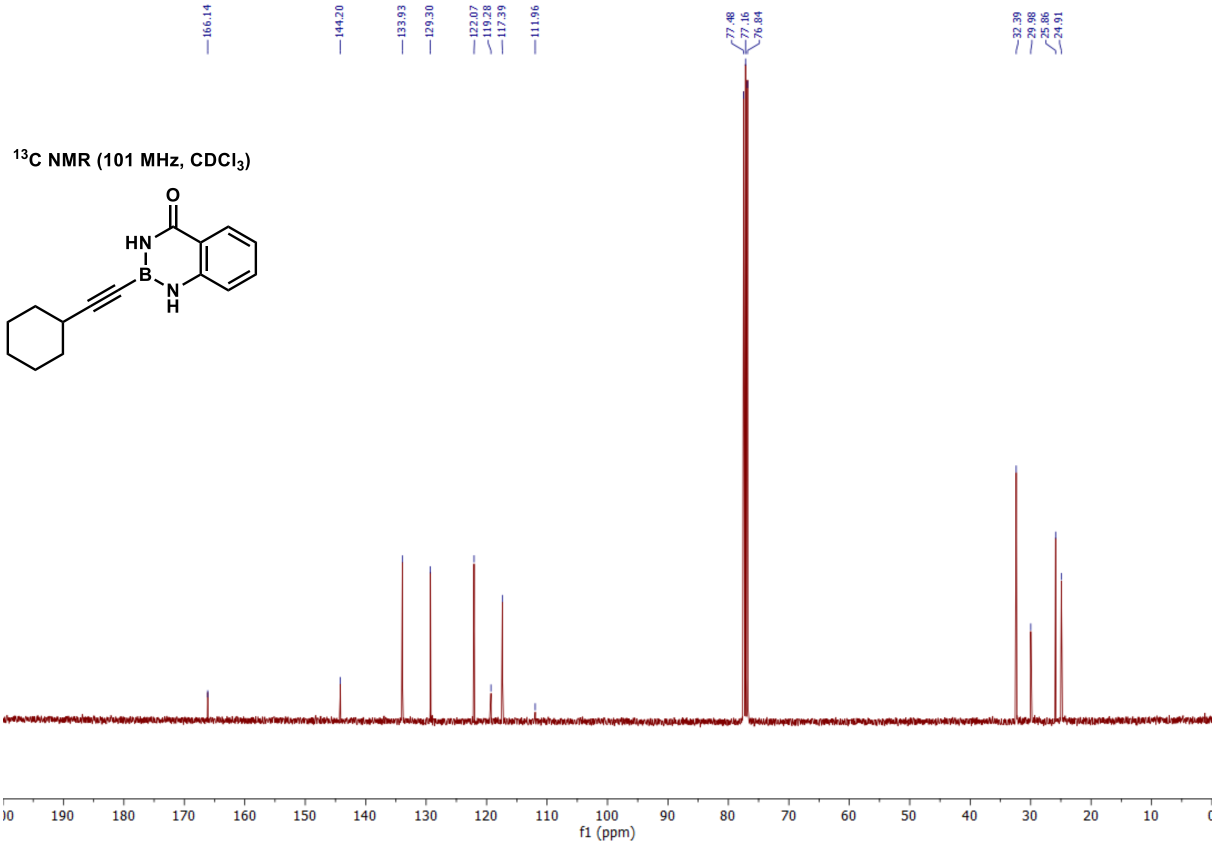

13C NMR (101 MHz, CDCl3)
O
HN
B
N
H
f1 (ppm)

^{11}B NMR (128 MHz, $CDCl_3$)

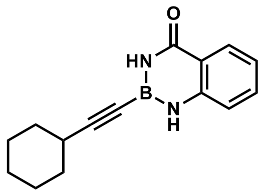

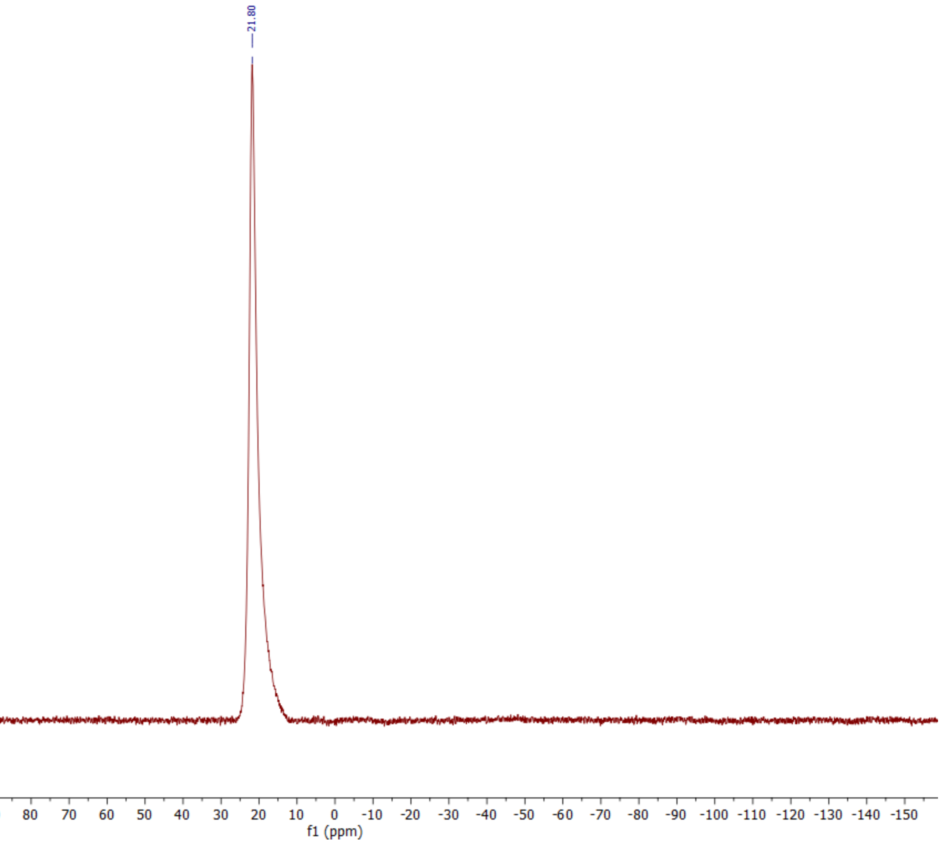

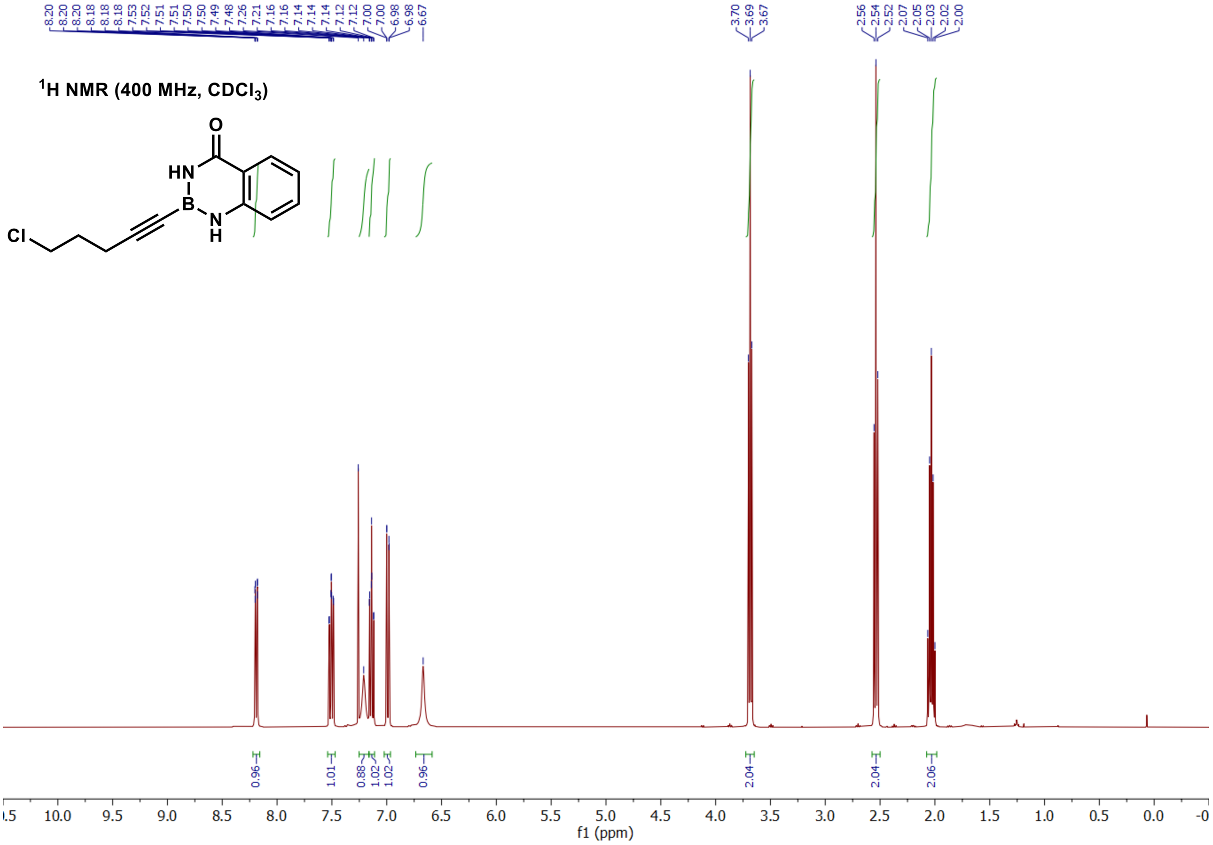

^{1}H NMR (400 MHz, CDCl$_3$)
HN
B
N
H
O
Cl
f1 (ppm)

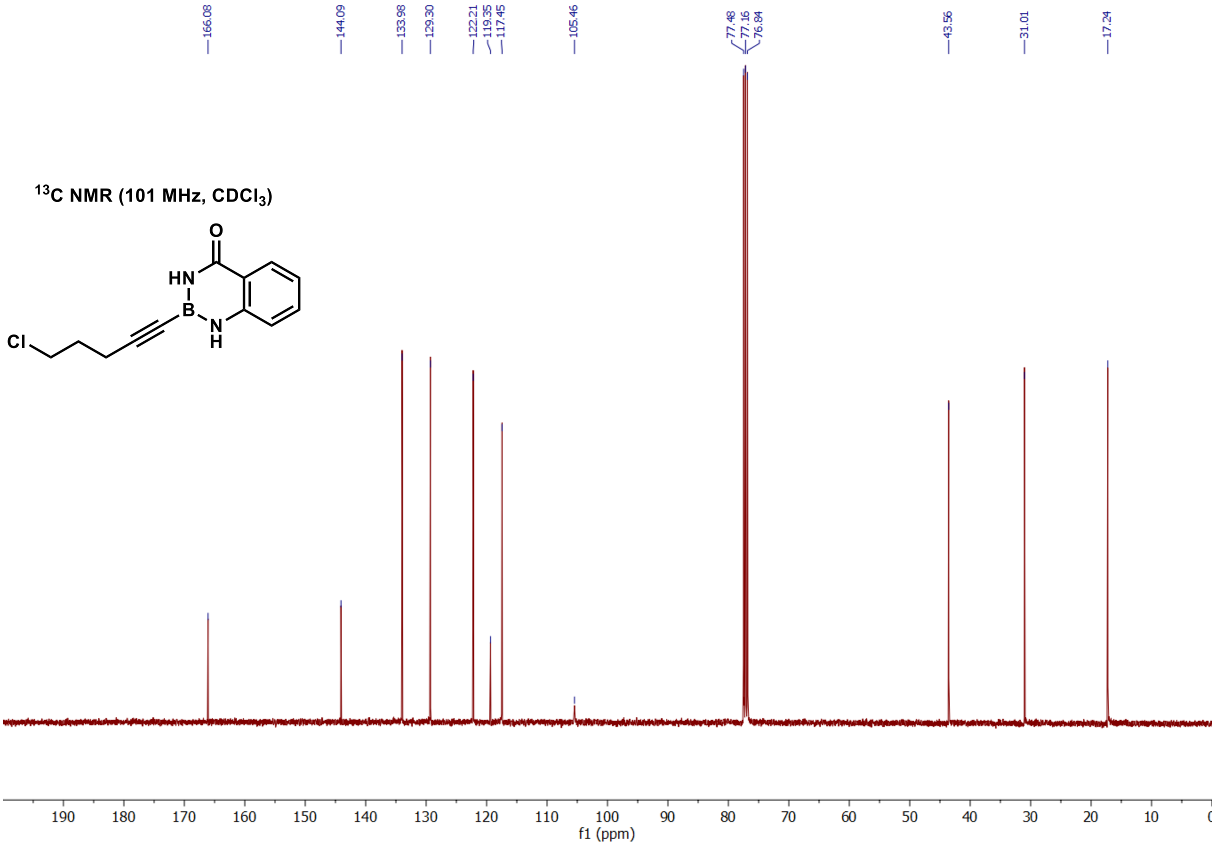

^{13}C NMR (101 MHz, CDCl$_3$)
HN
B
N
H
O
Cl
166.08
144.09
133.98
129.30
122.21
119.35
117.45
105.46
77.48
77.16
76.84
43.56
31.01
17.24
f1 (ppm)

^{11}B NMR (128 MHz, $CDCl_3$)

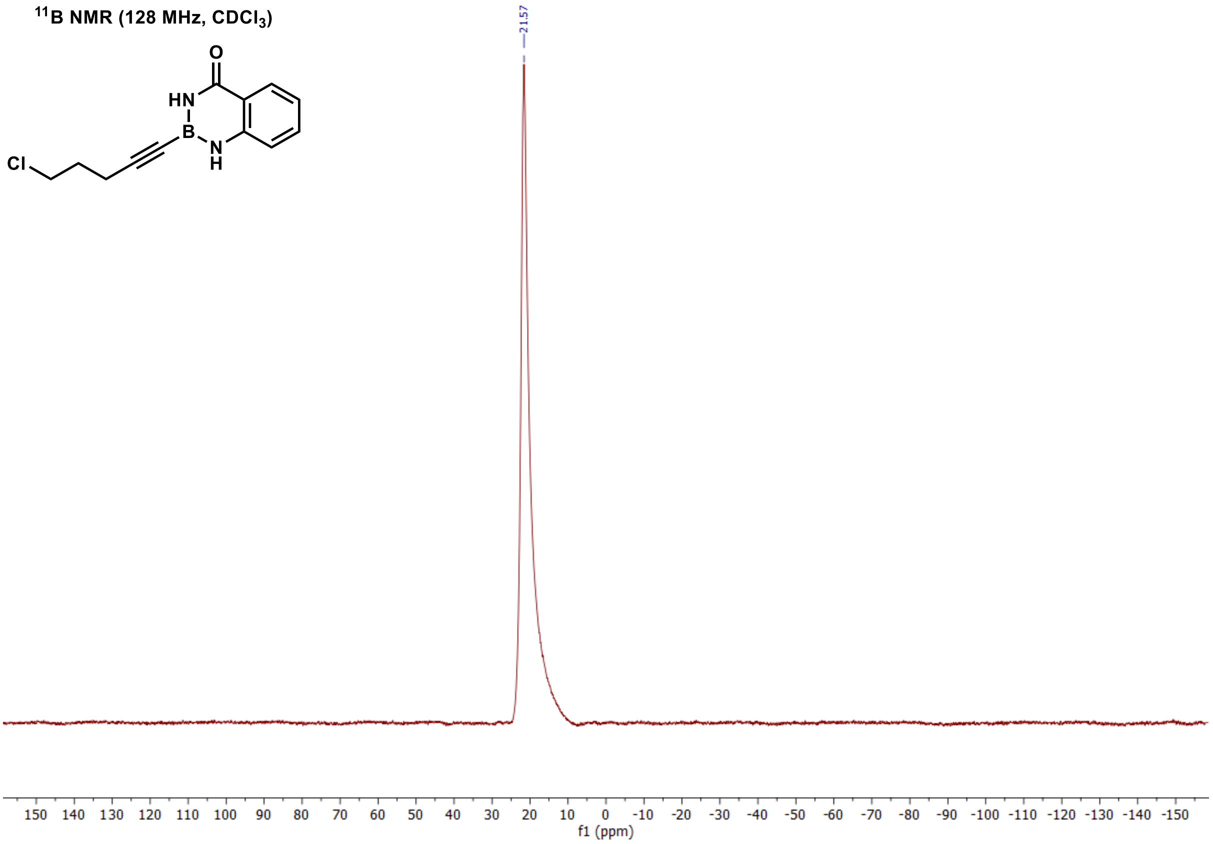

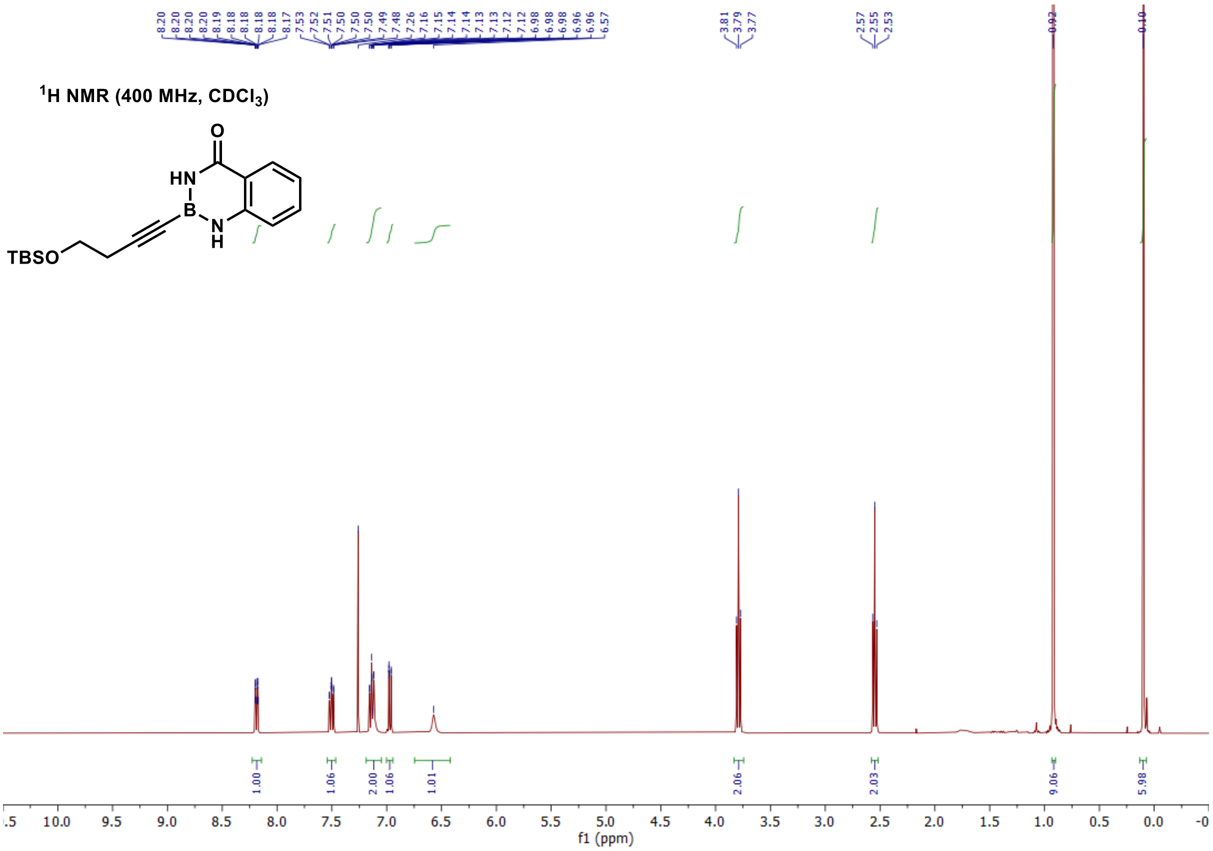

^{1}H NMR (400 MHz, $CDCl_3$)
HN
B
N
H
O
TBSO
f1 (ppm)

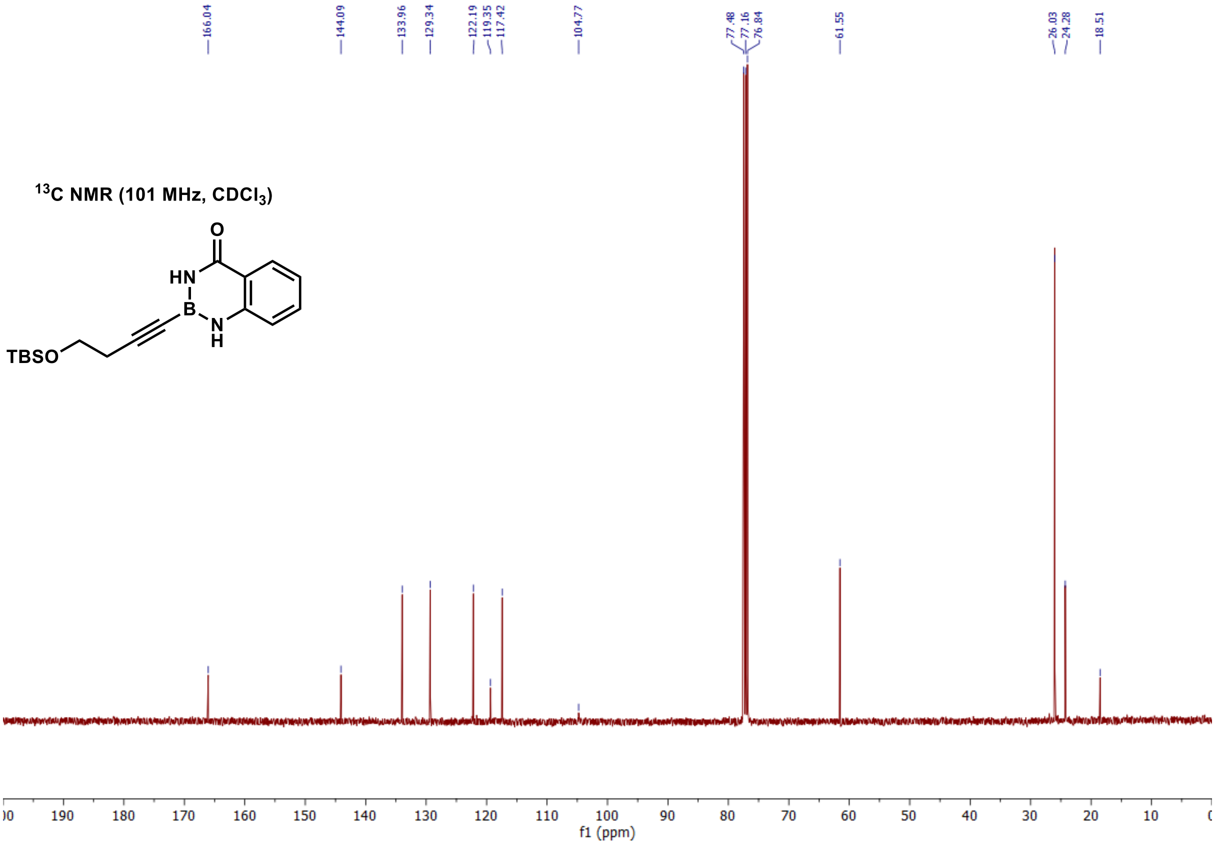

^{13}C NMR (101 MHz, $CDCl_3$)
HN
B
N
H
O
TBSO
f1 (ppm)

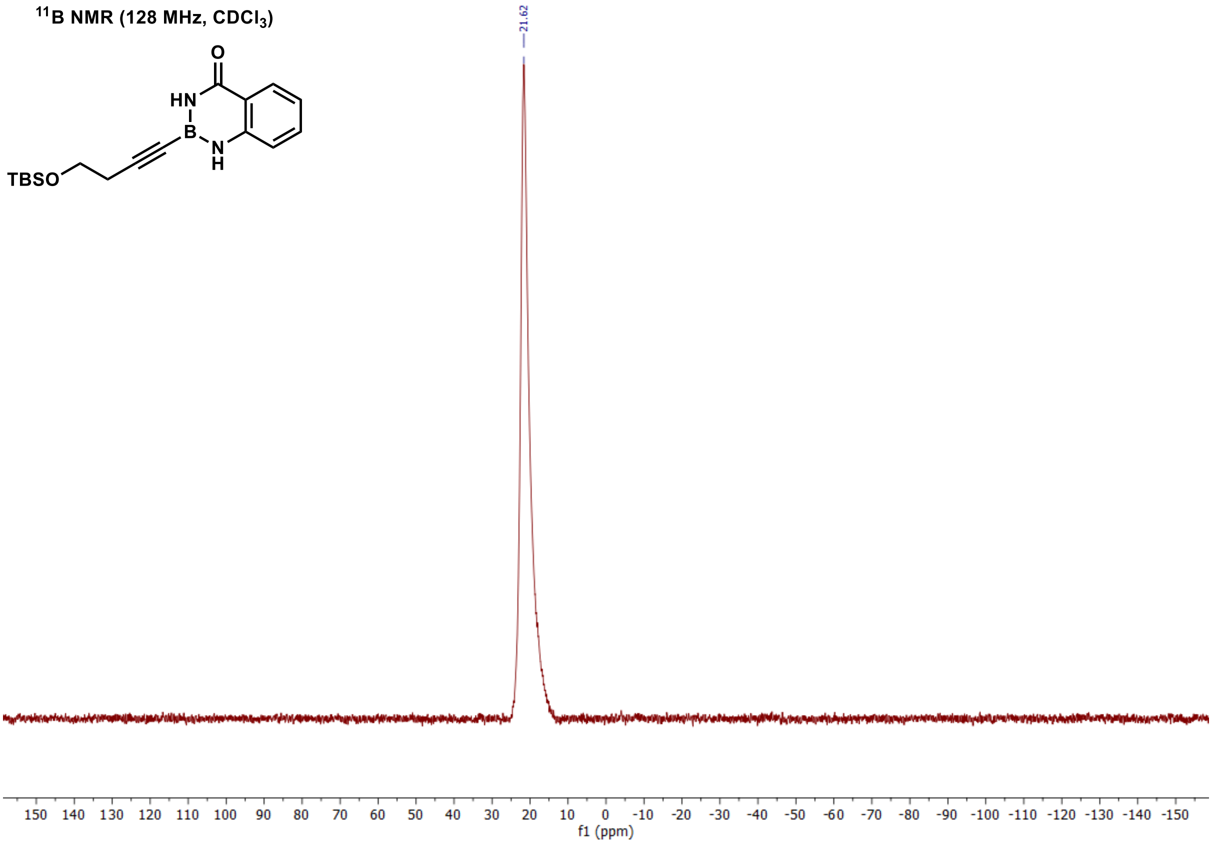

11B NMR (128 MHz, CDCl3)
O
HN
B
N
H
TBSO
21.62
150 140 130 120 110 100 90 80 70 60 50 40 30 20 10 0 -10 -20 -30 -40 -50 -60 -70 -80 -90 -100 -110 -120 -130 -140 -150
f1 (ppm)

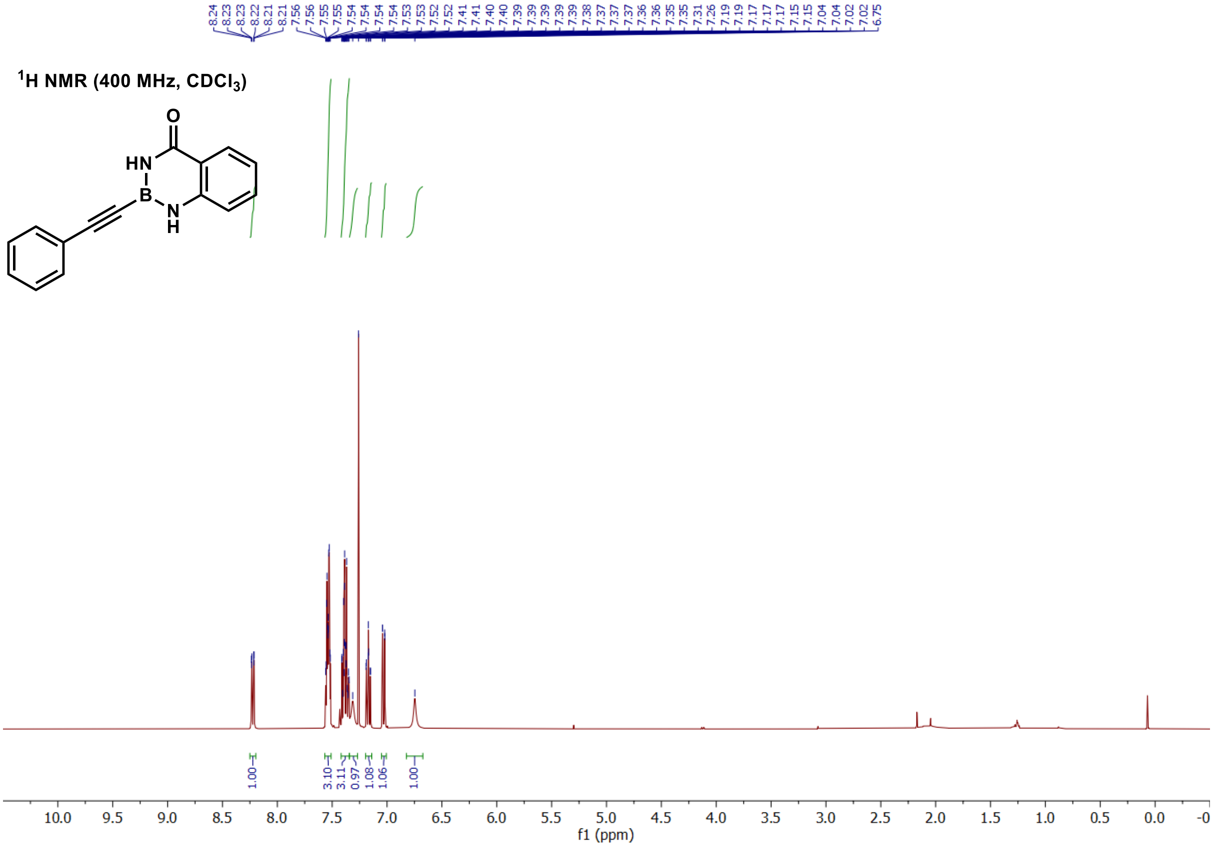

8.24 8.23 8.23 8.22 8.21 8.21 7.56 7.55 7.55 7.54 7.54 7.54 7.54 7.53 7.53 7.52 7.52 7.41 7.41 7.40 7.40 7.39 7.39 7.39 7.39 7.39 7.38 7.37 7.37 7.37 7.36 7.36 7.35 7.35 7.31 7.26 7.19 7.19 7.17 7.17 7.17 7.15 7.15 7.04 7.04 7.02 7.02 6.75
1H NMR (400 MHz, CDCl3)
O
HN
B
N
H
1.00
3.10
3.11
0.97
1.08
1.06
1.00
10.0 9.5 9.0 8.5 8.0 7.5 7.0 6.5 6.0 5.5 5.0 4.5 4.0 3.5 3.0 2.5 2.0 1.5 1.0 0.5 0.0
f1 (ppm)

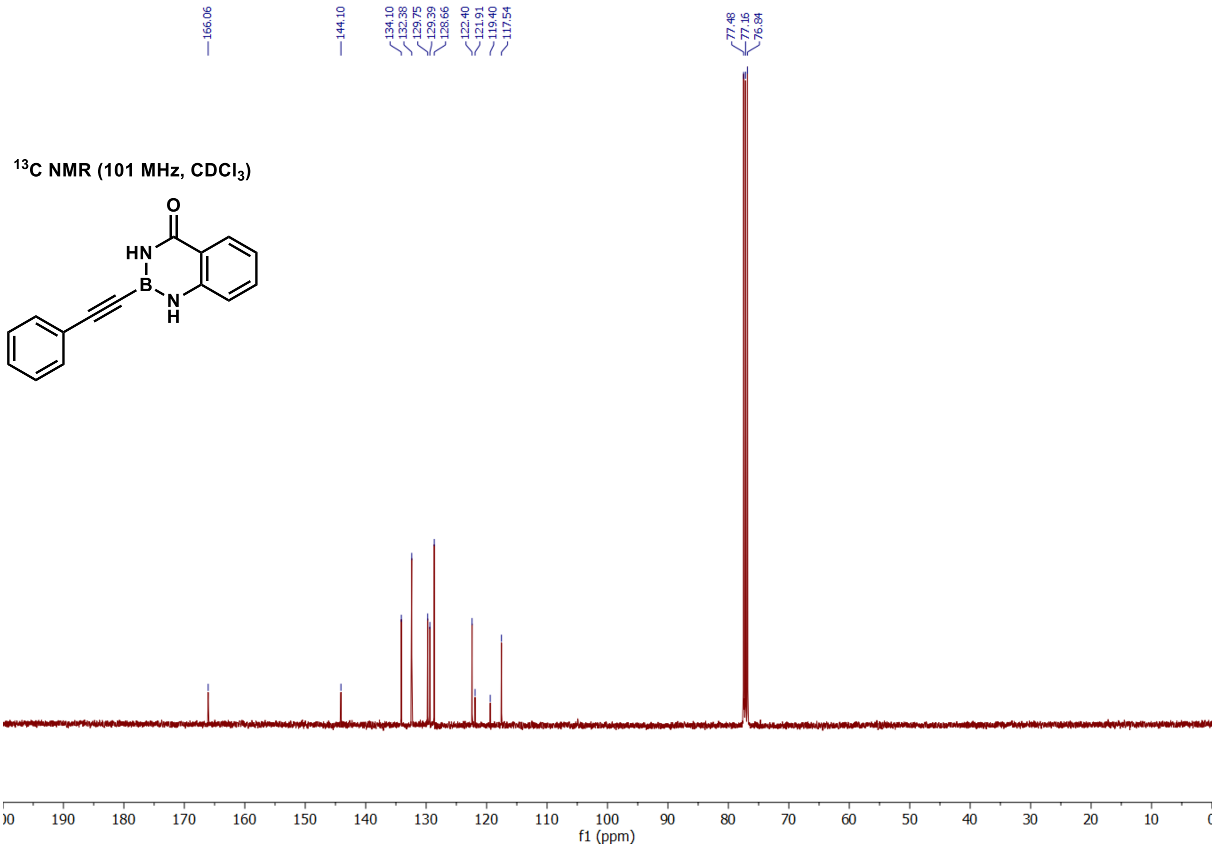

166.06
144.10
134.10 132.38 129.75 129.39 128.66 122.40 121.91 119.40 117.54
77.48 77.16 76.84
13C NMR (101 MHz, CDCl3)
O
HN
B
N
H
190 180 170 160 150 140 130 120 110 100 90 80 70 60 50 40 30 20 10
f1 (ppm)

^{11}B NMR (128 MHz, $CDCl_3$)

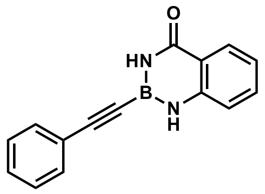

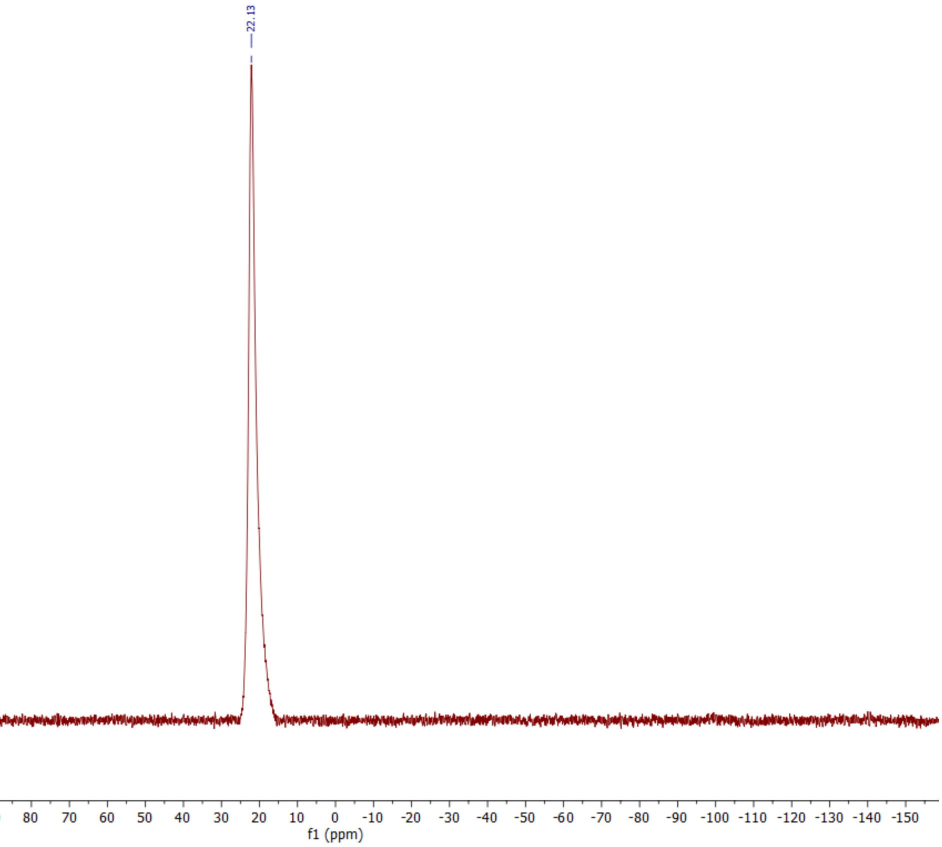

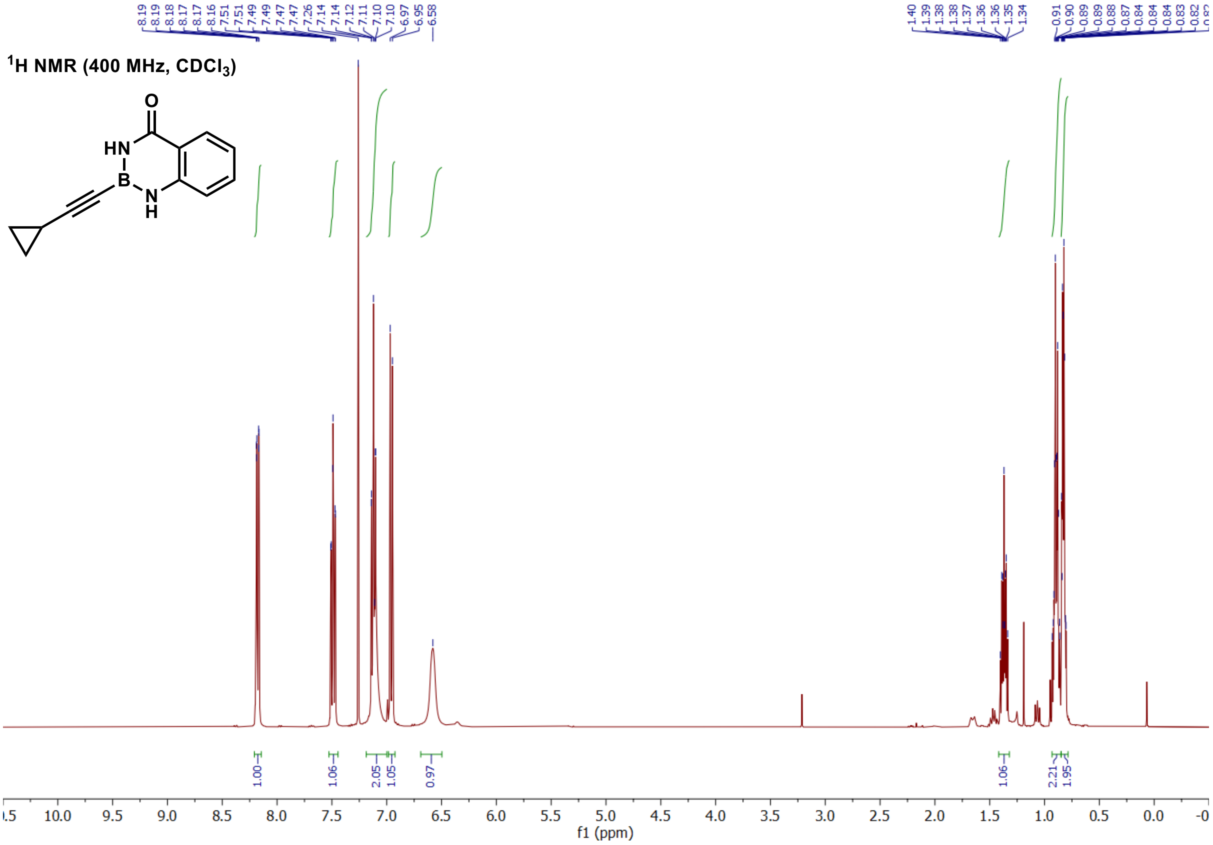

1H NMR (400 MHz, CDCl3)
O
HN
B
N
H
8.19 8.19 8.18 8.17 8.17 8.16 7.51 7.51 7.49 7.49 7.47 7.47 7.26 7.14 7.14 7.12 7.11 7.10 7.10 6.97 6.95 6.58
1.40 1.39 1.38 1.38 1.37 1.36 1.36 1.35 1.34
0.91 0.90 0.89 0.89 0.88 0.88 0.87 0.84 0.84 0.84 0.83 0.82
1.00 1.06 2.05 1.05 0.97 1.06 2.21 1.95
10.0 9.5 9.0 8.5 8.0 7.5 7.0 6.5 6.0 5.5 5.0 4.5 4.0 3.5 3.0 2.5 2.0 1.5 1.0 0.5 0.0
f1 (ppm)

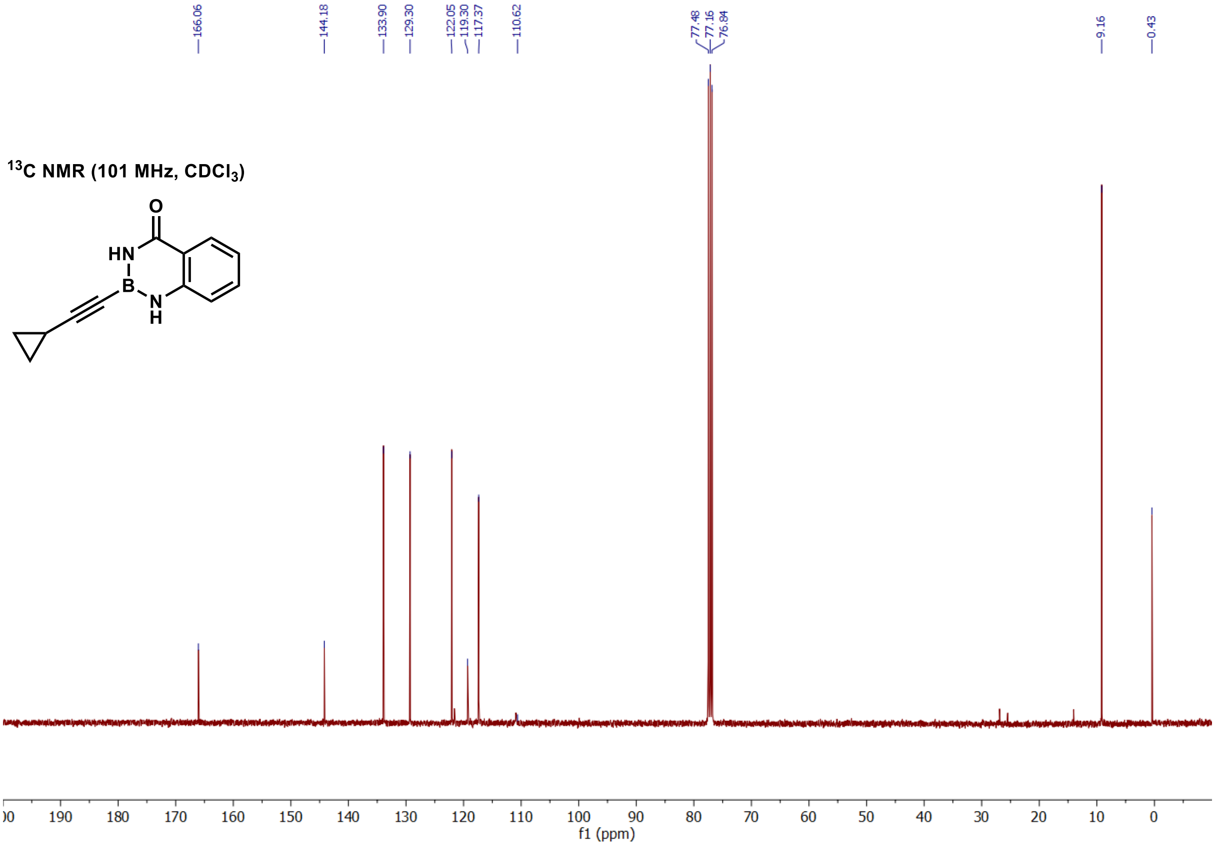

13C NMR (101 MHz, CDCl3)
O
HN
B
N
H
166.06
144.18
133.90
129.30
122.05
119.30
117.37
110.62
77.48
77.16
76.84
9.16
0.43
190 180 170 160 150 140 130 120 110 100 90 80 70 60 50 40 30 20 10 0
f1 (ppm)

^{11}B NMR (128 MHz, $CDCl_3$)

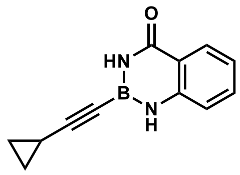

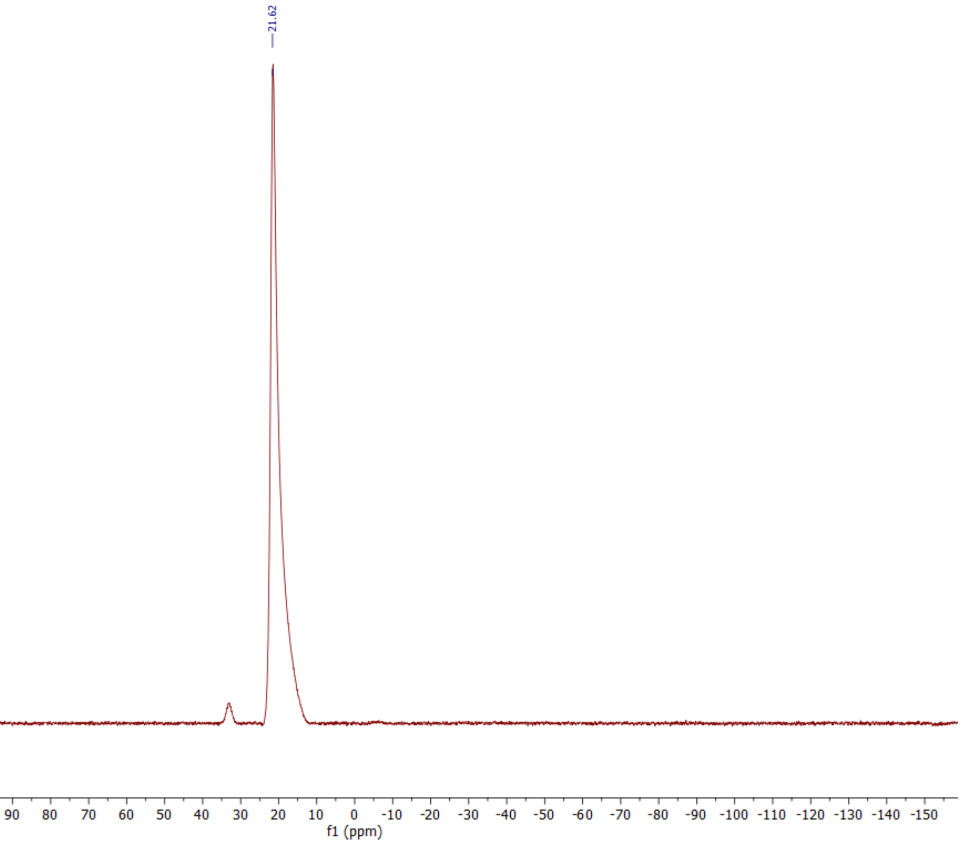

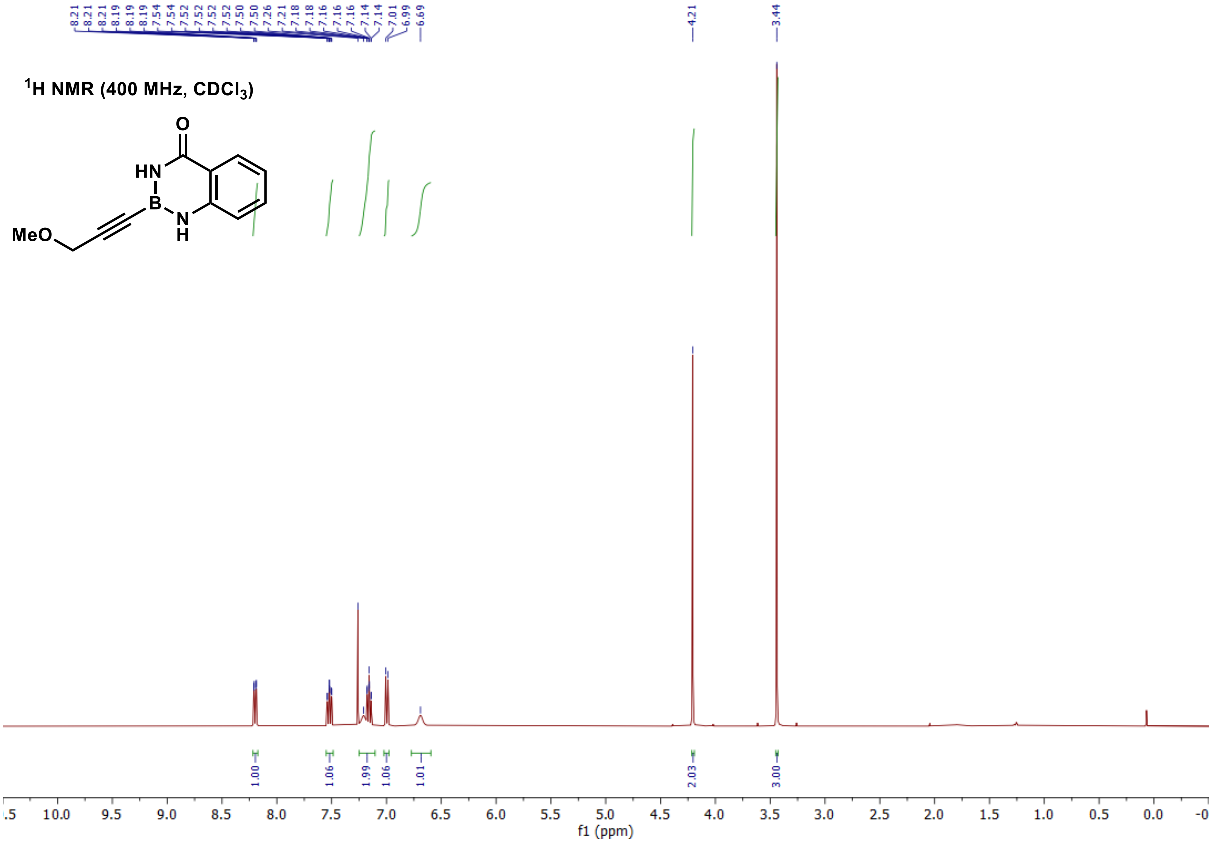
1H NMR (400 MHz, CDCl3)
MeO
HN
B
N
H
O
f1 (ppm)

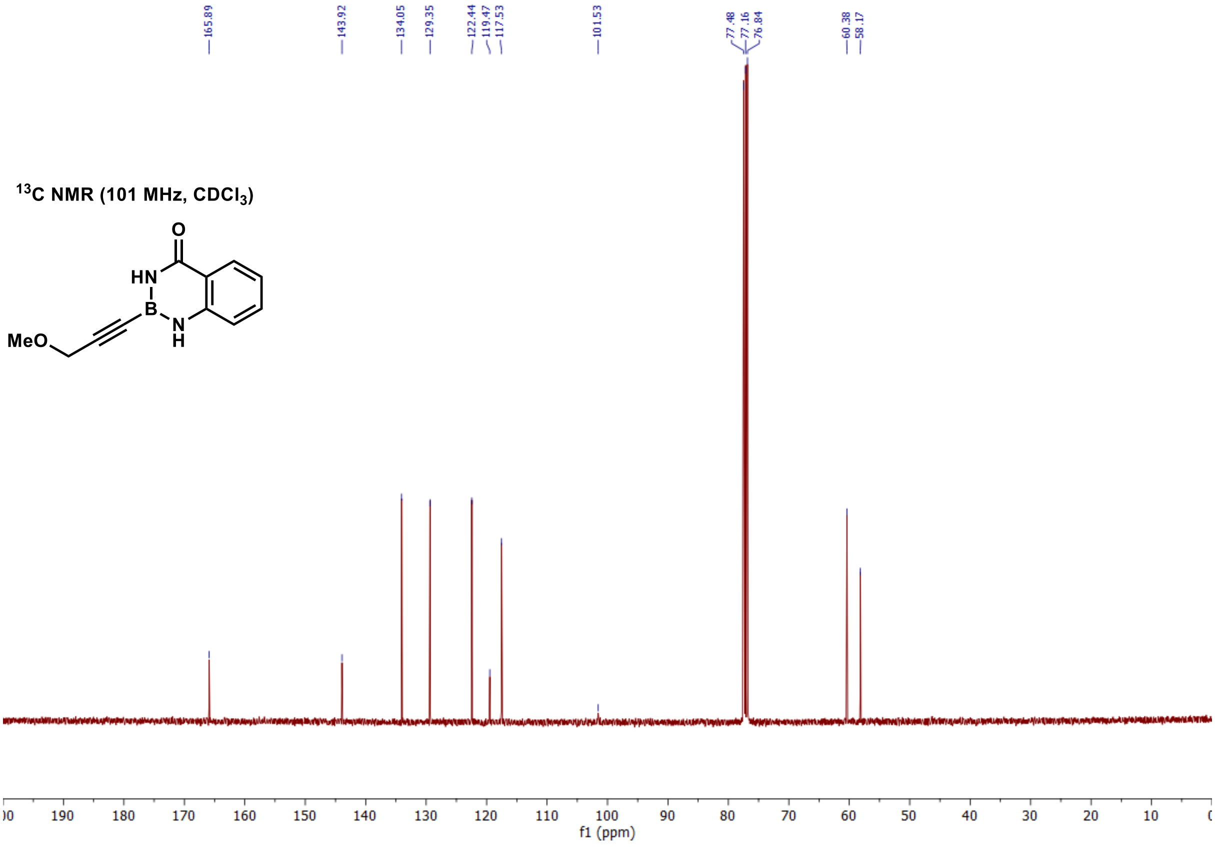
13C NMR (101 MHz, CDCl3)
MeO
HN
B
N
H
O
f1 (ppm)

^{11}B NMR (128 MHz, $CDCl_3$)

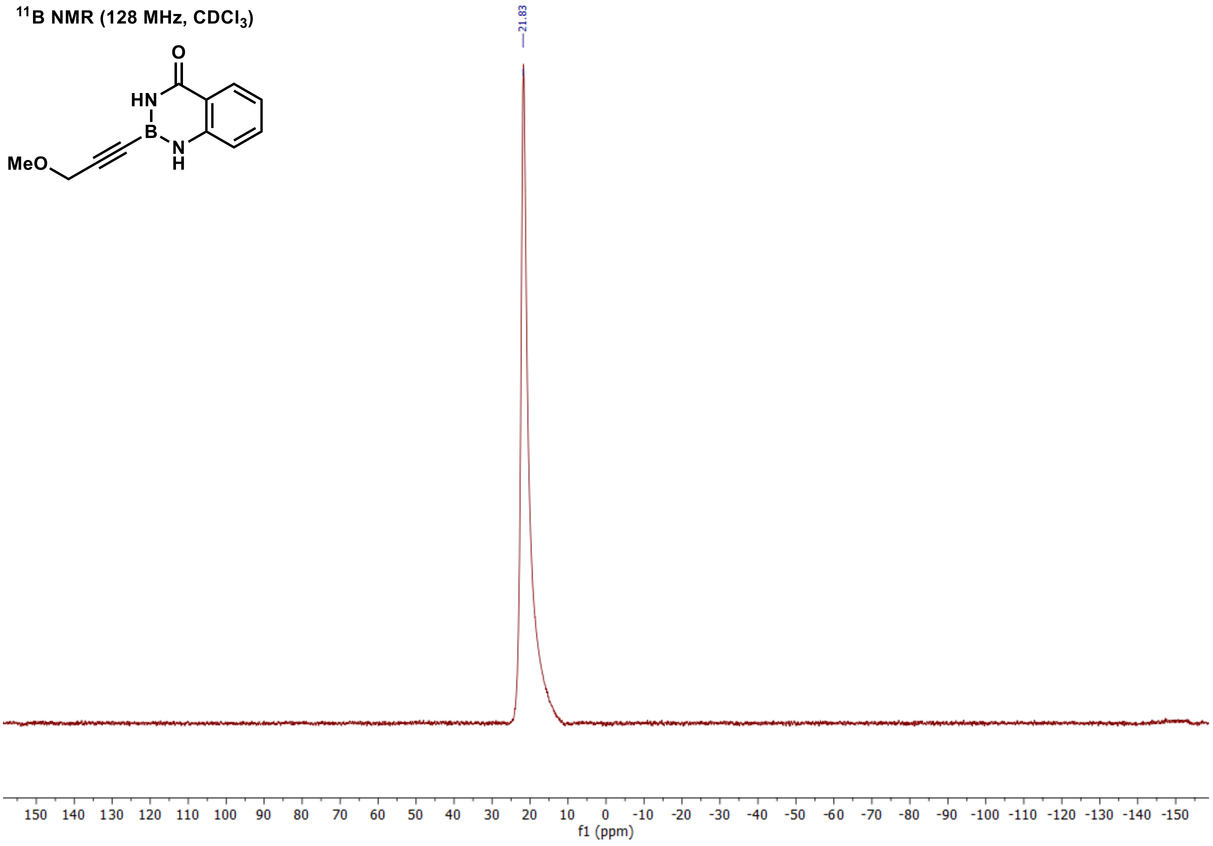

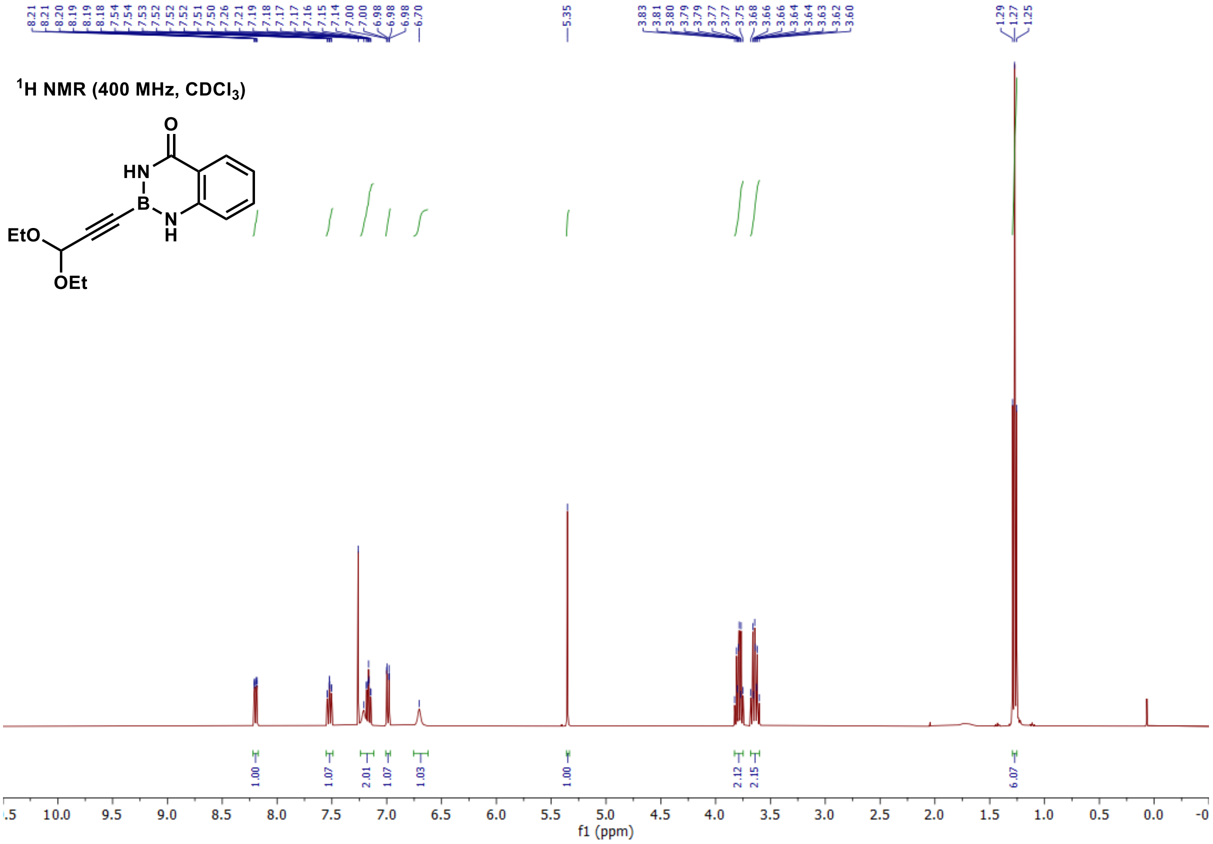
8.21
8.21
8.20
8.19
8.19
8.18
7.54
7.54
7.53
7.52
7.52
7.52
7.51
7.50
7.26
7.21
7.19
7.18
7.17
7.17
7.16
7.15
7.14
7.00
7.00
6.98
6.98
6.98
6.70
5.35
3.83
3.81
3.80
3.79
3.79
3.77
3.77
3.75
3.68
3.66
3.66
3.64
3.64
3.63
3.62
3.60
1.29
1.27
1.25
^{1}H NMR (400 MHz, $CDCl_3$)
O
HN
B
N
H
EtO
OEt
1.00
1.07
2.01
1.07
1.03
1.00
2.12
2.15
6.07
f1 (ppm)

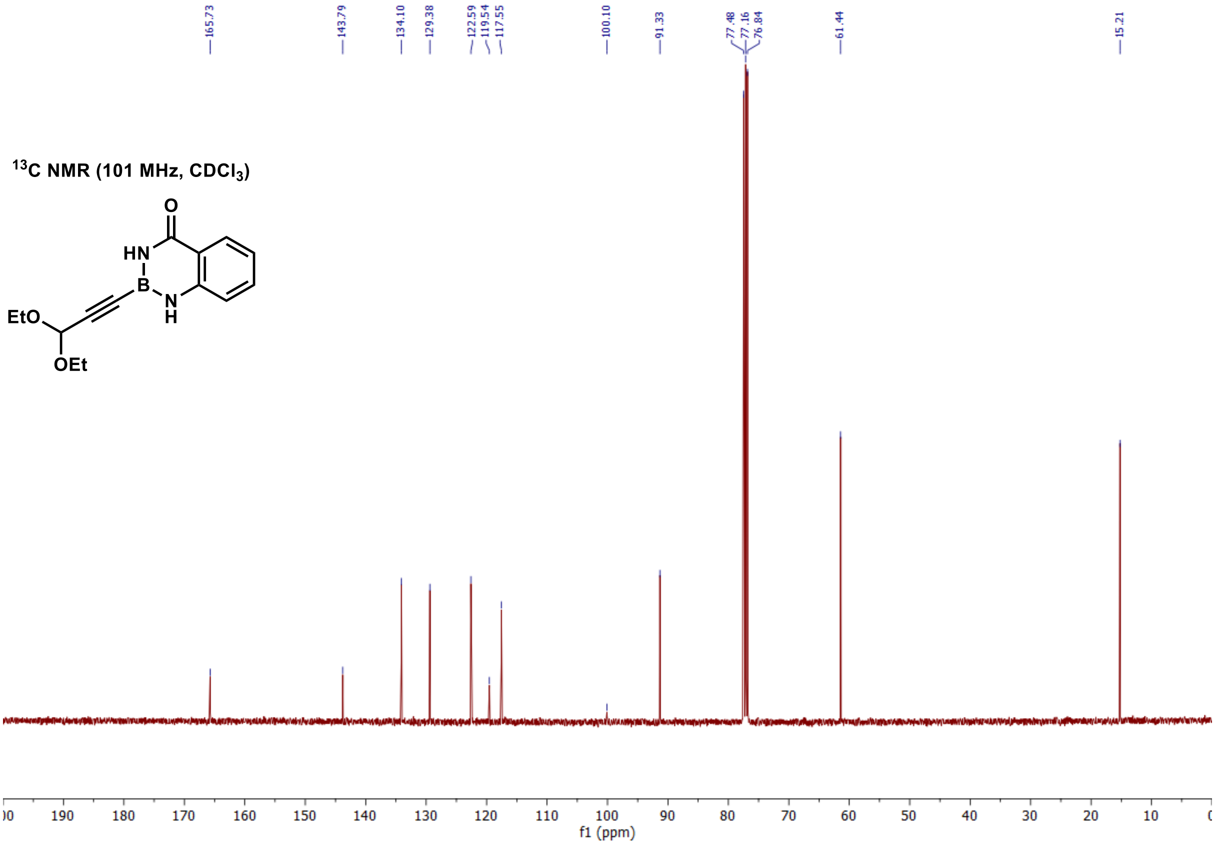
165.73
143.79
134.10
129.38
122.59
119.54
117.55
100.10
91.33
77.48
77.16
76.84
61.44
15.21
^{13}C NMR (101 MHz, $CDCl_3$)
O
HN
B
N
H
EtO
OEt
f1 (ppm)

^{11}B NMR (128 MHz, $CDCl_3$)

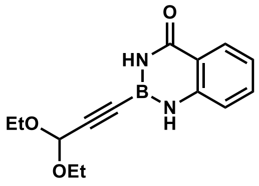

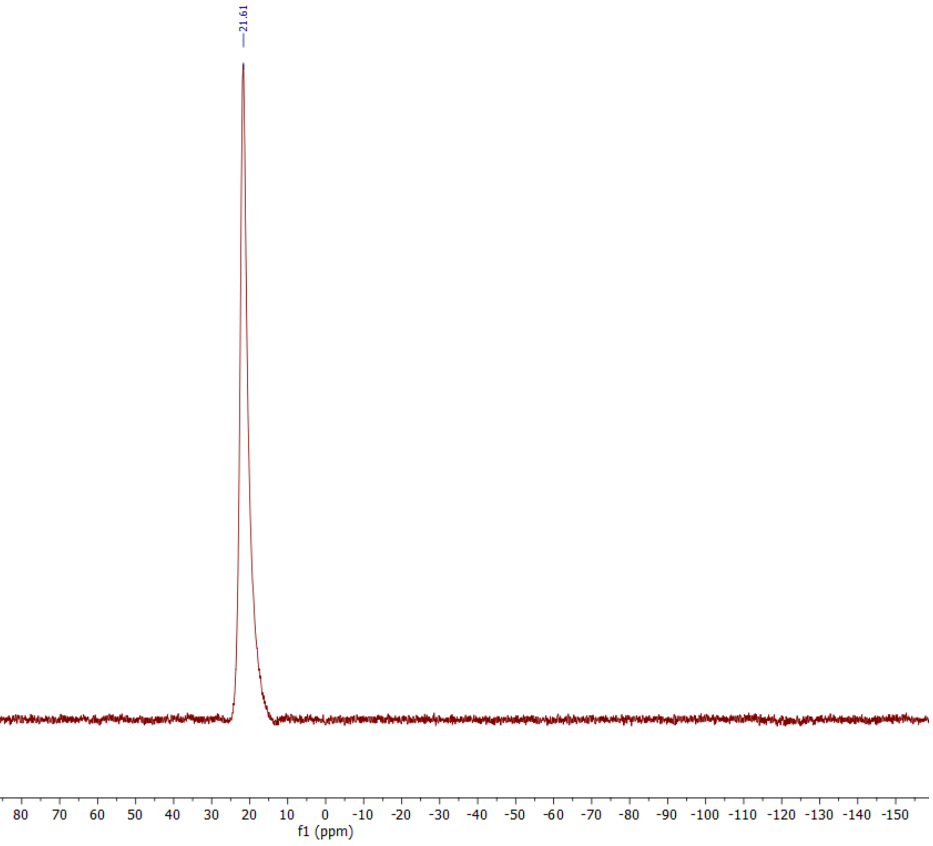

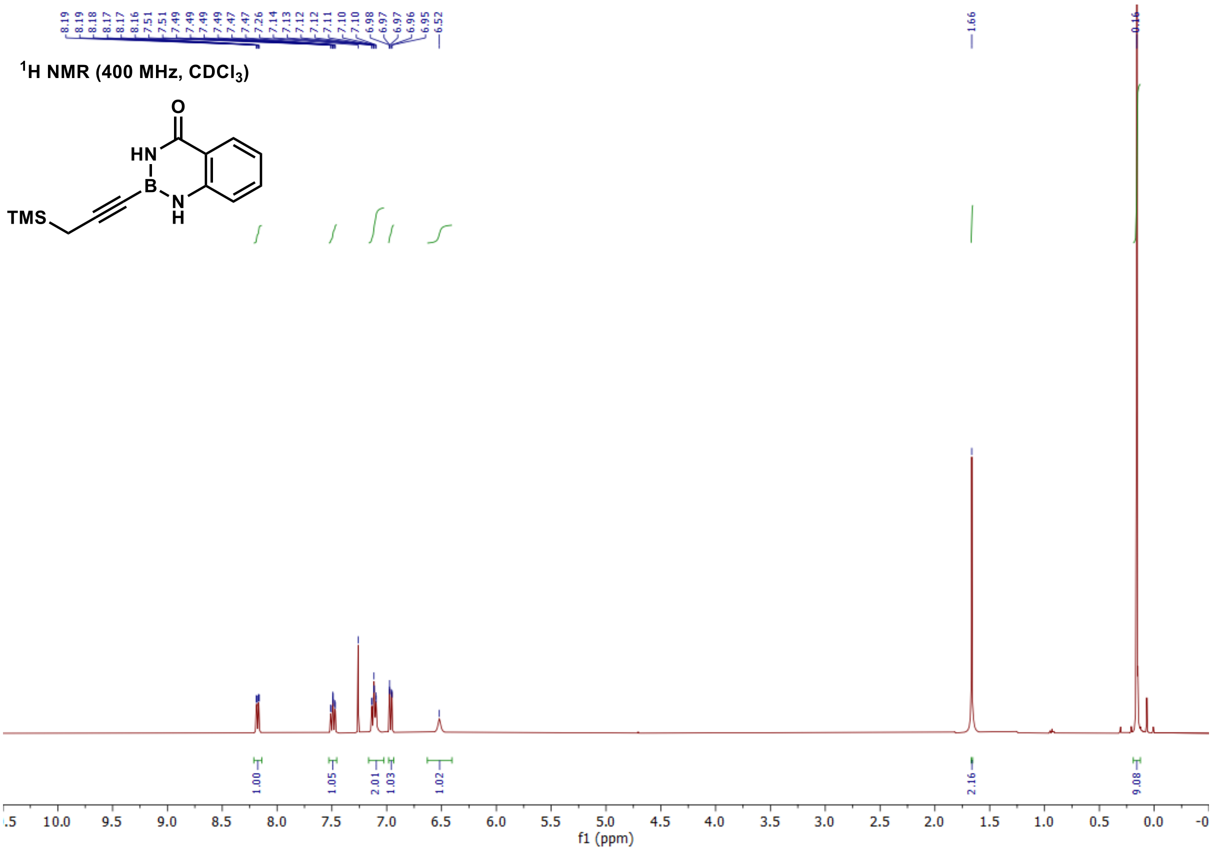
1H NMR (400 MHz, CDCl3)
TMS
HN
B
N
H
O
f1 (ppm)

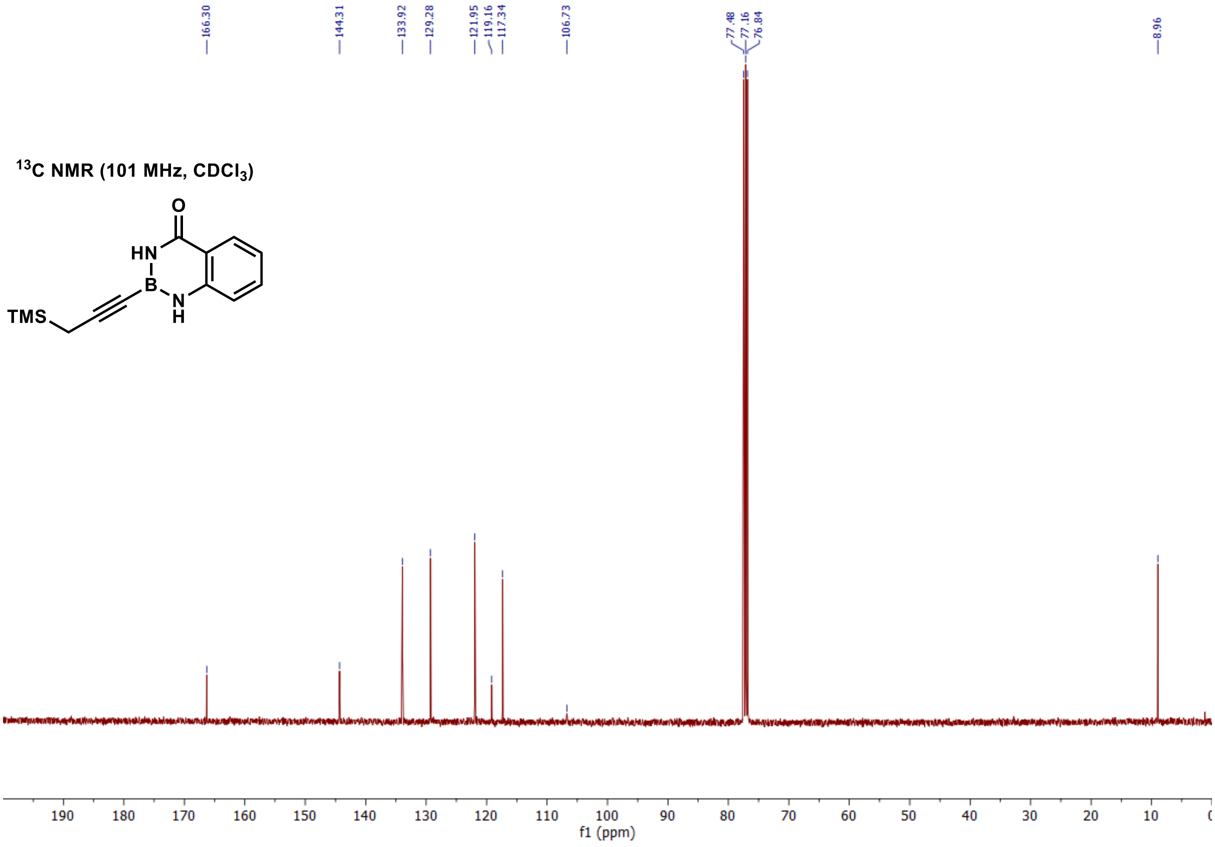
13C NMR (101 MHz, CDCl3)
TMS
HN
B
N
H
O
f1 (ppm)

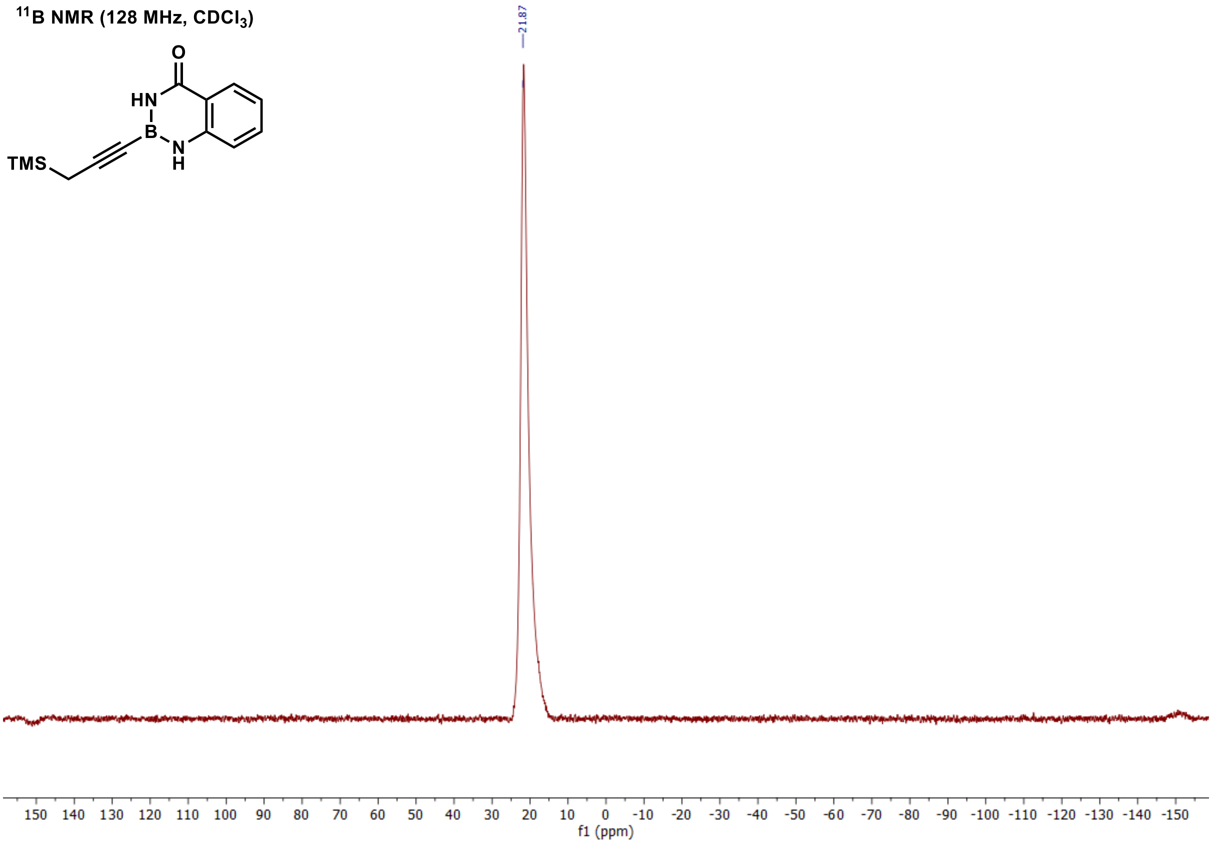

^{11}B NMR (128 MHz, $CDCl_3$)
O
HN
B
N
H
TMS
21.87
150 140 130 120 110 100 90 80 70 60 50 40 30 20 10 0 -10 -20 -30 -40 -50 -60 -70 -80 -90 -100 -110 -120 -130 -140 -150
f1 (ppm)

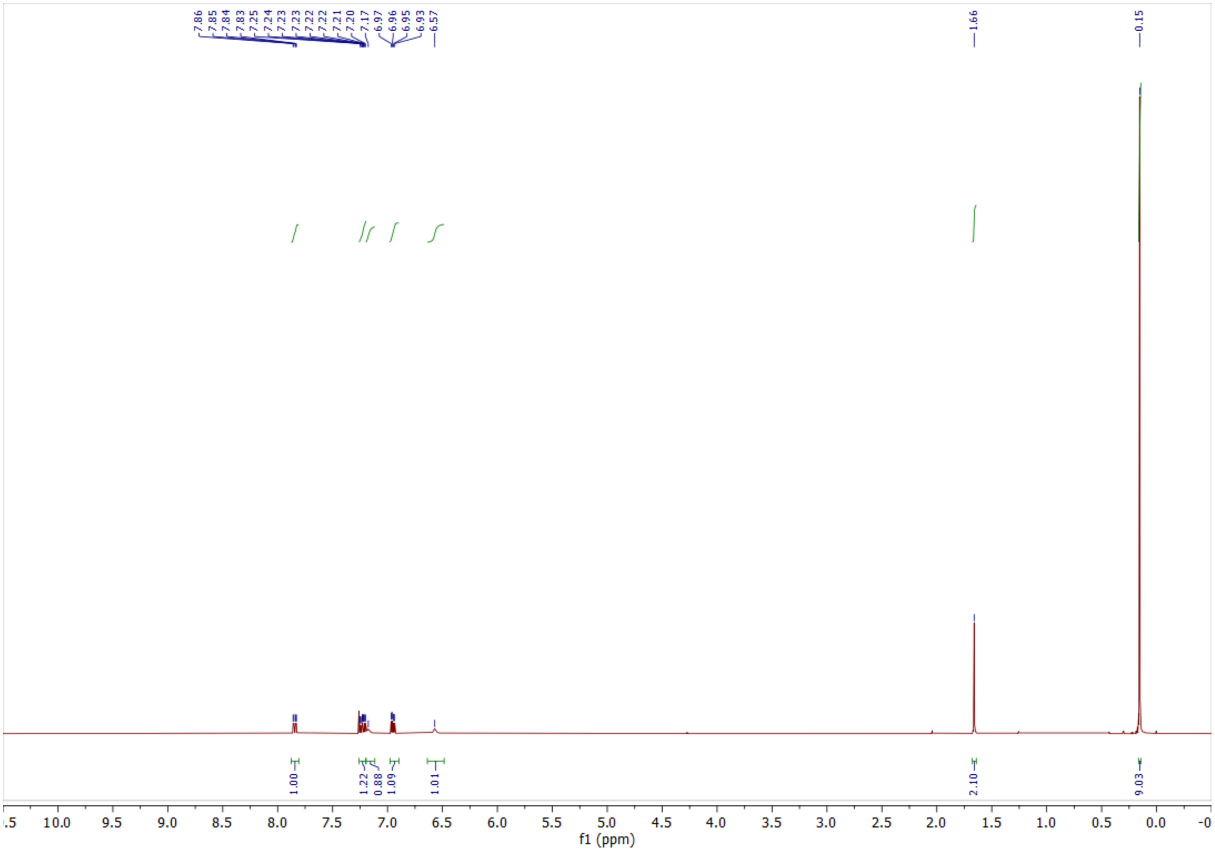

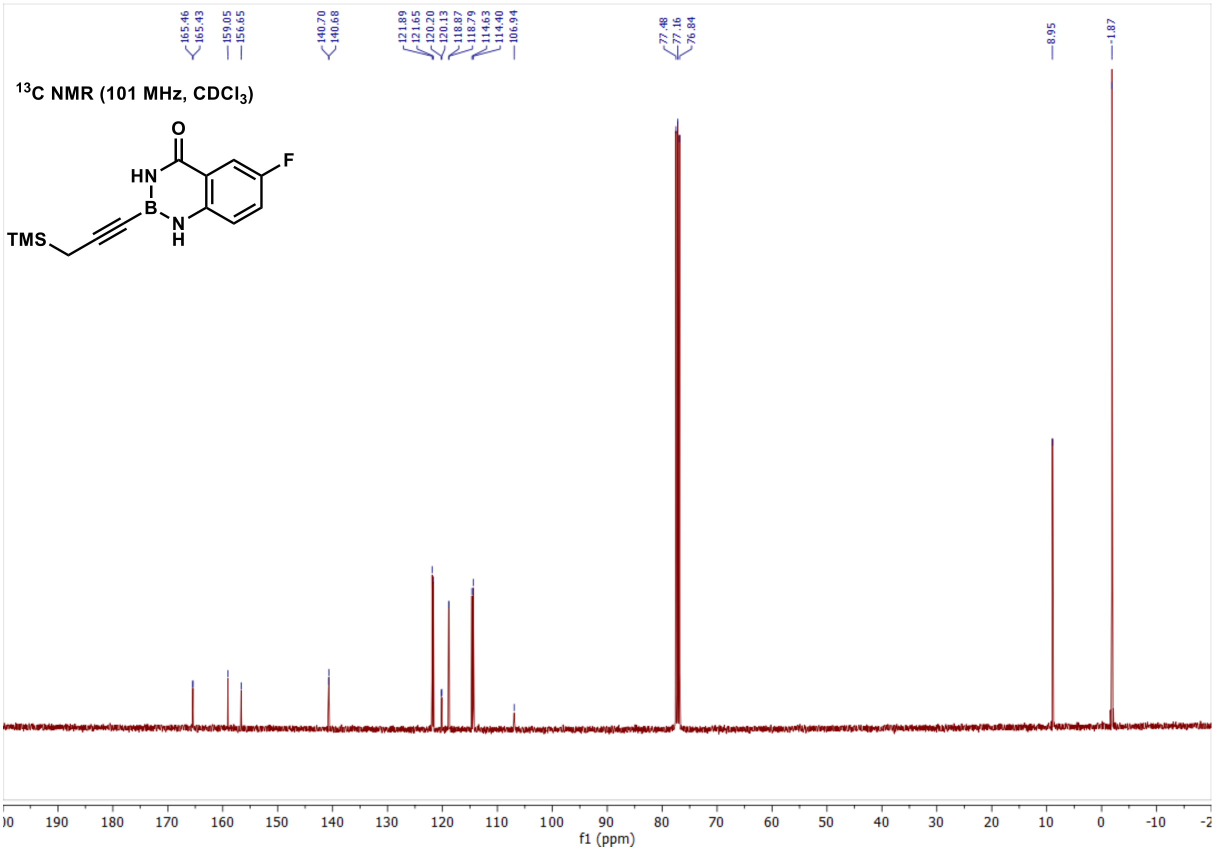
13C NMR (101 MHz, CDCl3)
TMS
HN
B
N
H
O
F
f1 (ppm)

^{11}B NMR (128 MHz, $CDCl_3$)

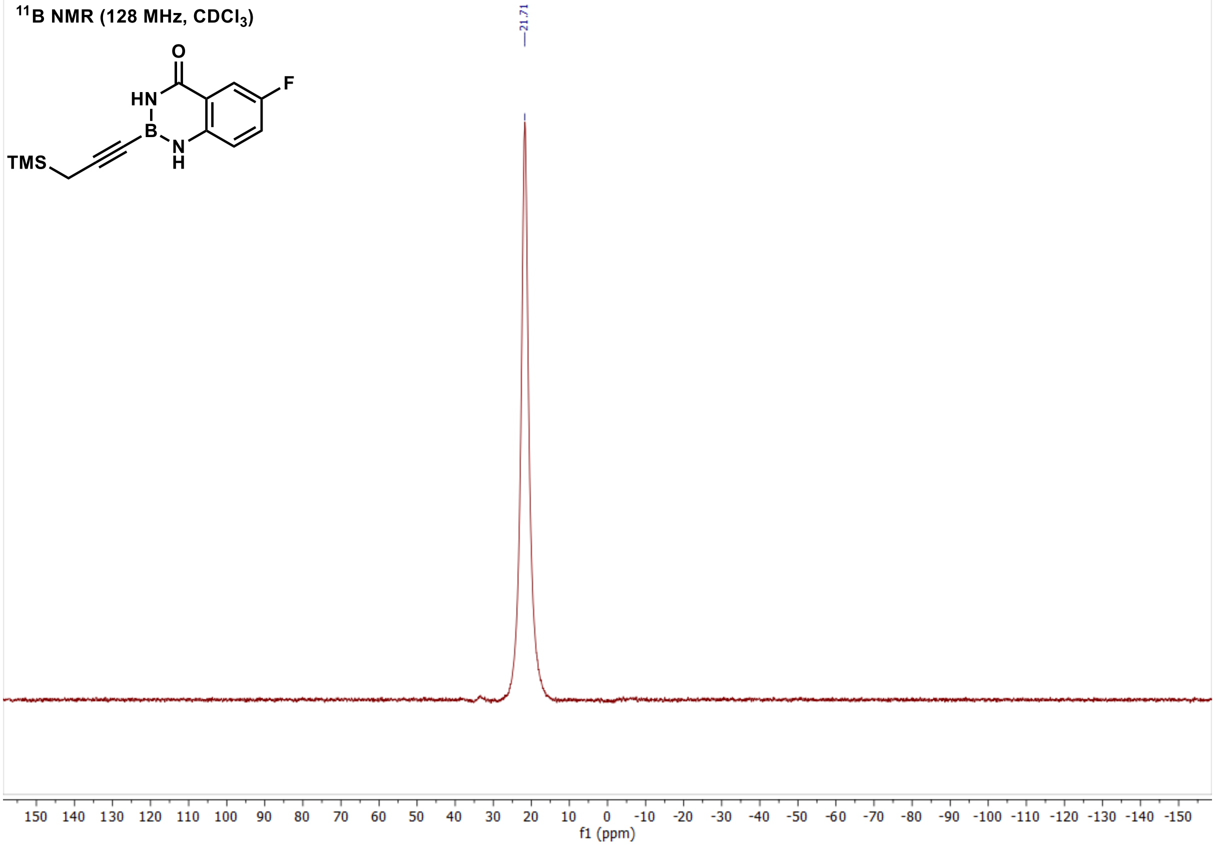

^{19}F NMR (282 MHz, $CDCl_3$)

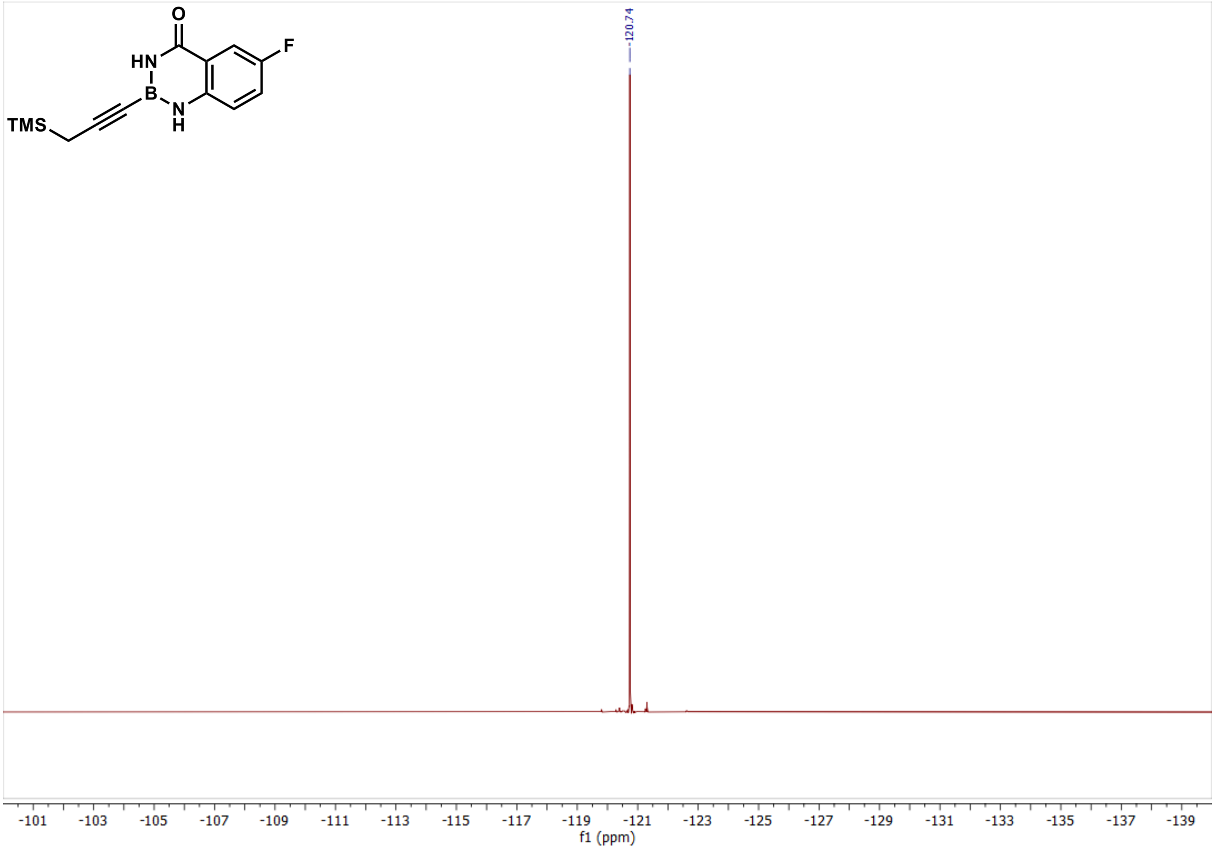

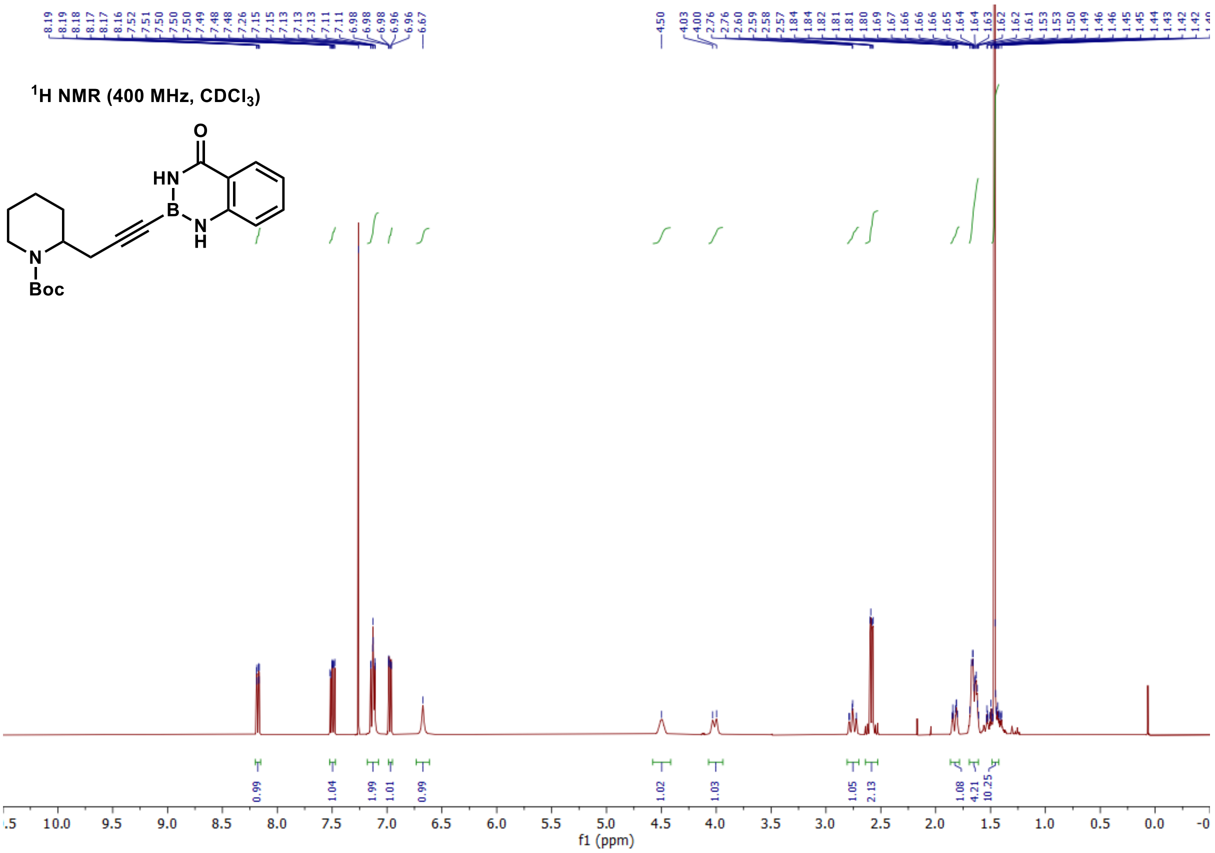

1H NMR (400 MHz, CDCl3)
O
HN
B
N
H
N
Boc
f1 (ppm)

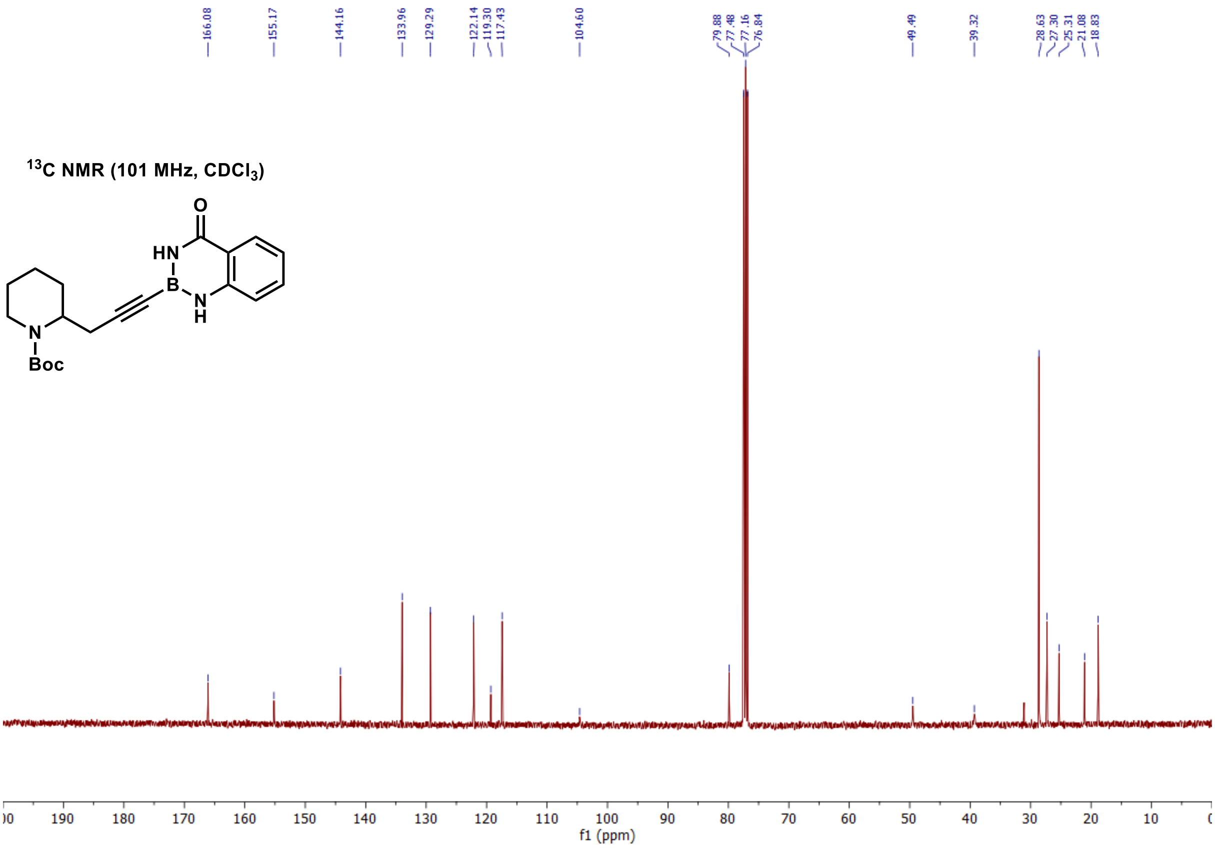

13C NMR (101 MHz, CDCl3)
O
HN
B
N
H
N
Boc
f1 (ppm)

^{11}B NMR (128 MHz, $CDCl_3$)

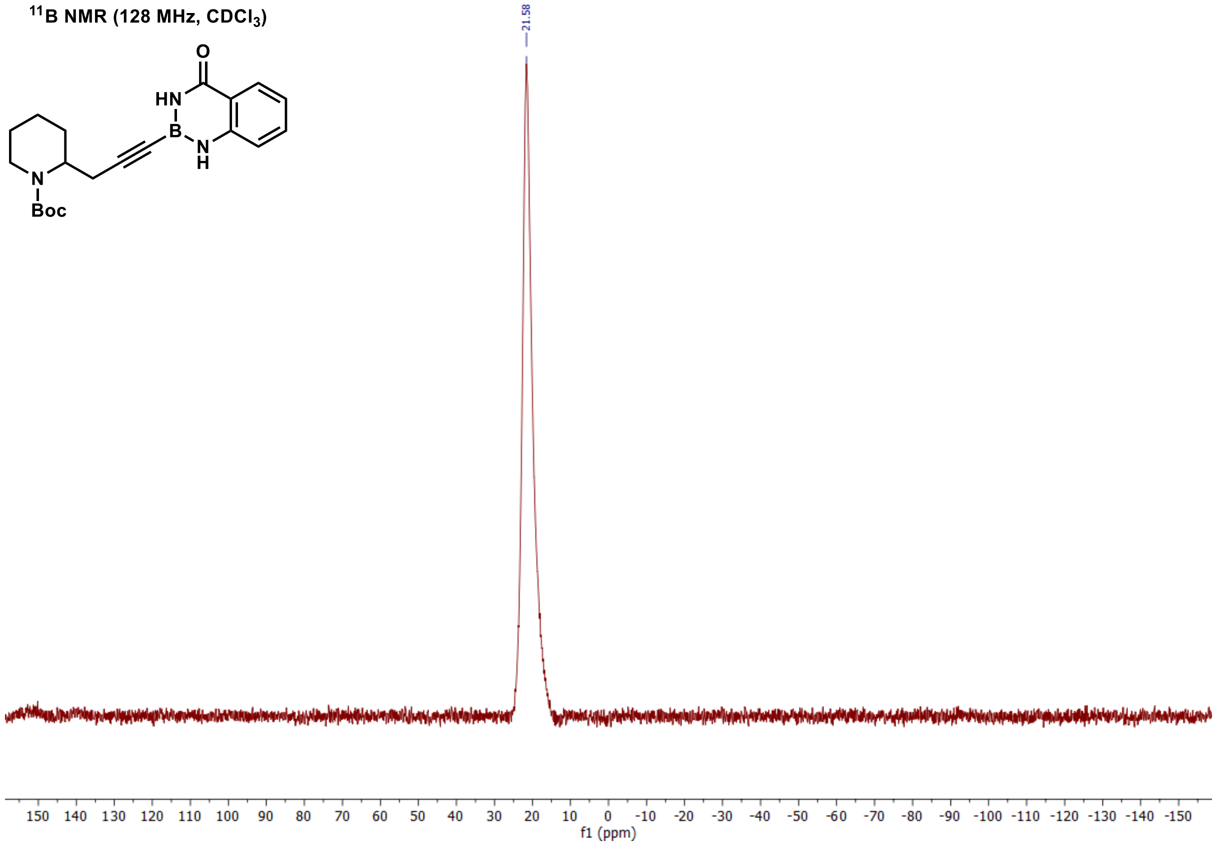

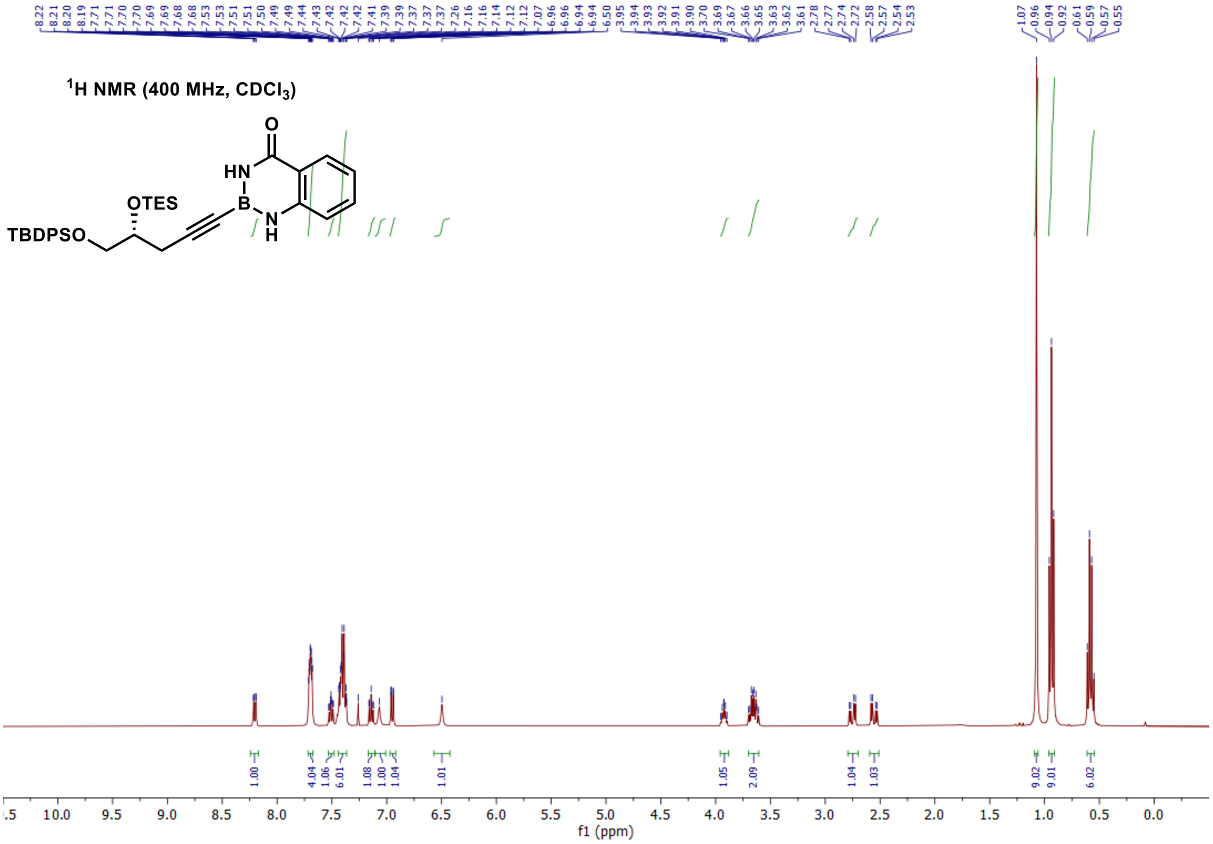
8.22 8.21 8.20 8.19 7.71 7.71 7.70 7.69 7.69 7.68 7.53 7.53 7.51 7.51 7.50 7.49 7.49 7.44 7.43 7.42 7.42 7.41 7.39 7.39 7.37 7.37 7.37 7.26 7.16 7.16 7.14 7.12 7.12 7.07 6.96 6.96 6.94 6.94 6.50 3.95 3.94 3.93 3.92 3.91 3.90 3.70 3.69 3.67 3.66 3.65 3.63 3.62 3.61 2.78 2.77 2.74 2.72 2.58 2.57 2.54 2.53 1.07 0.96 0.94 0.92 0.61 0.59 0.57 0.55
1H NMR (400 MHz, CDCl3)
O
HN
B
N
H
OTES
TBDPSO
1.00 4.04 1.06 6.01 1.08 1.00 1.04 1.01 1.05 2.09 1.04 1.03 9.02 9.01 6.02
f1 (ppm)

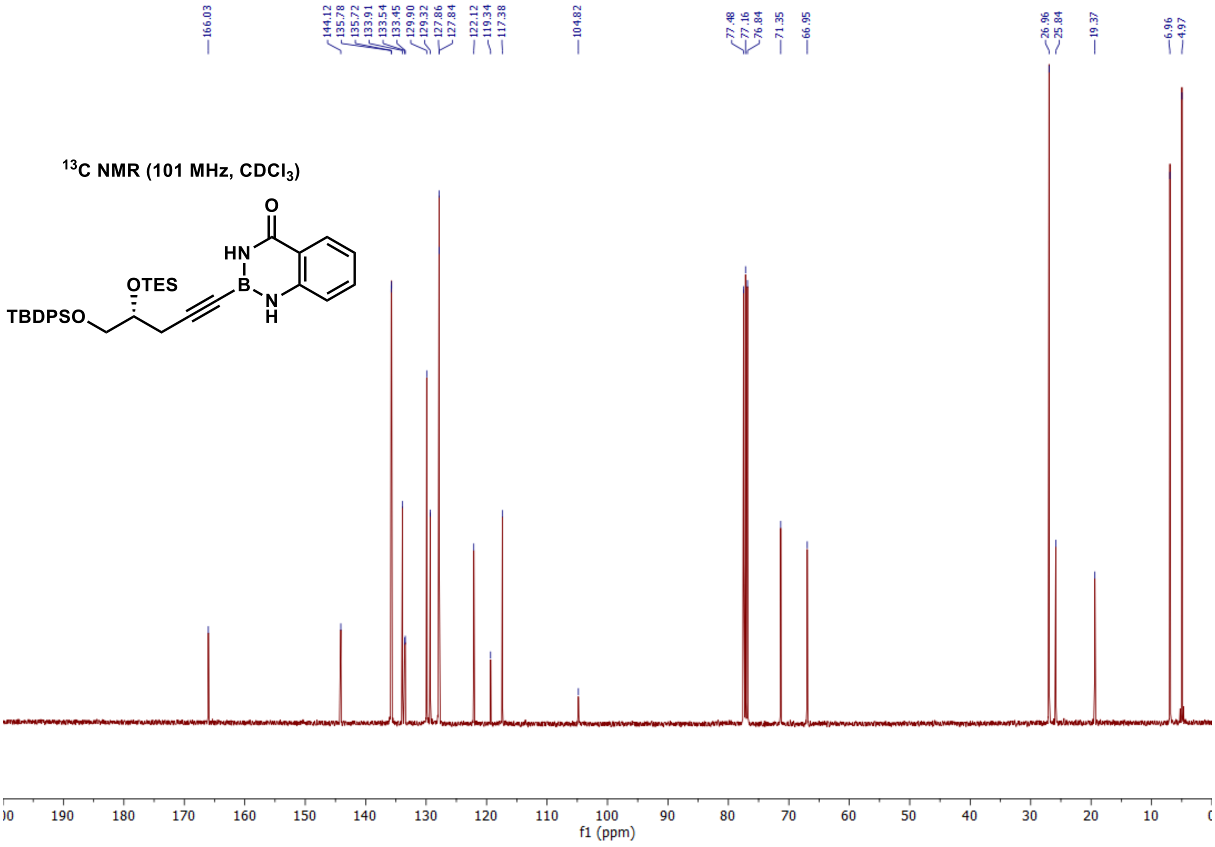
166.03 144.12 135.78 135.72 133.91 133.54 133.45 129.90 129.32 127.86 127.84 122.12 119.34 117.38 104.82 77.48 77.16 76.84 71.35 66.95 26.96 25.84 19.37 6.96 4.97
13C NMR (101 MHz, CDCl3)
O
HN
B
N
H
OTES
TBDPSO
f1 (ppm)

^{11}B NMR (128 MHz, $CDCl_3$)

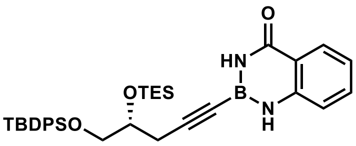

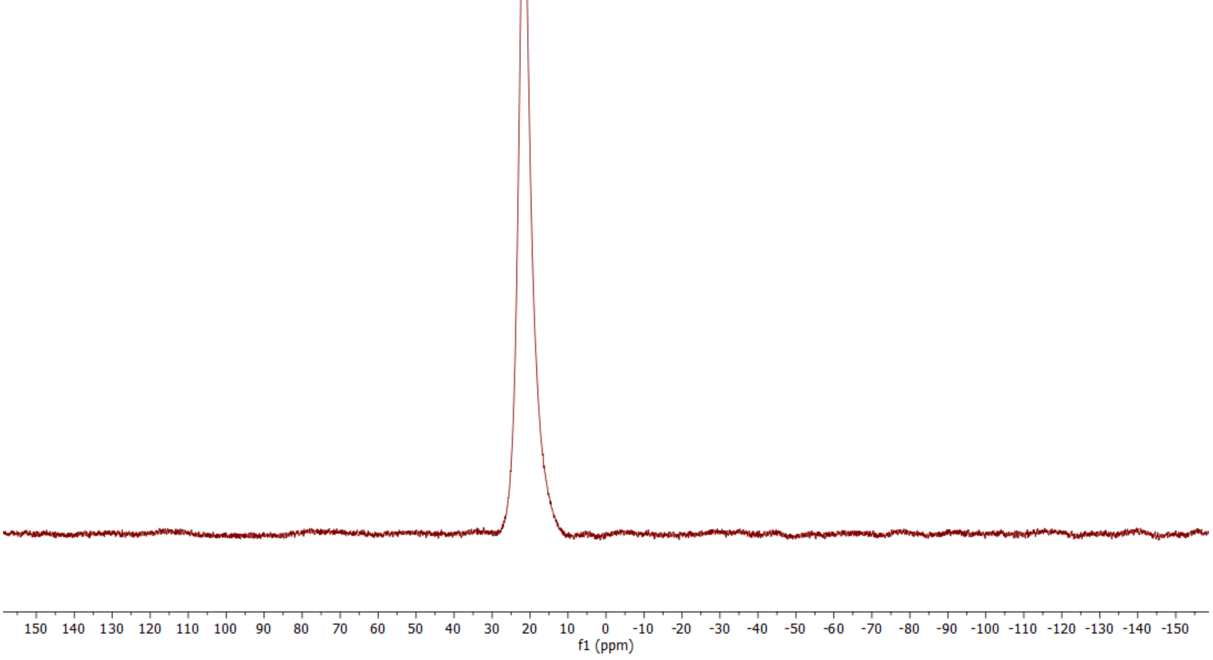

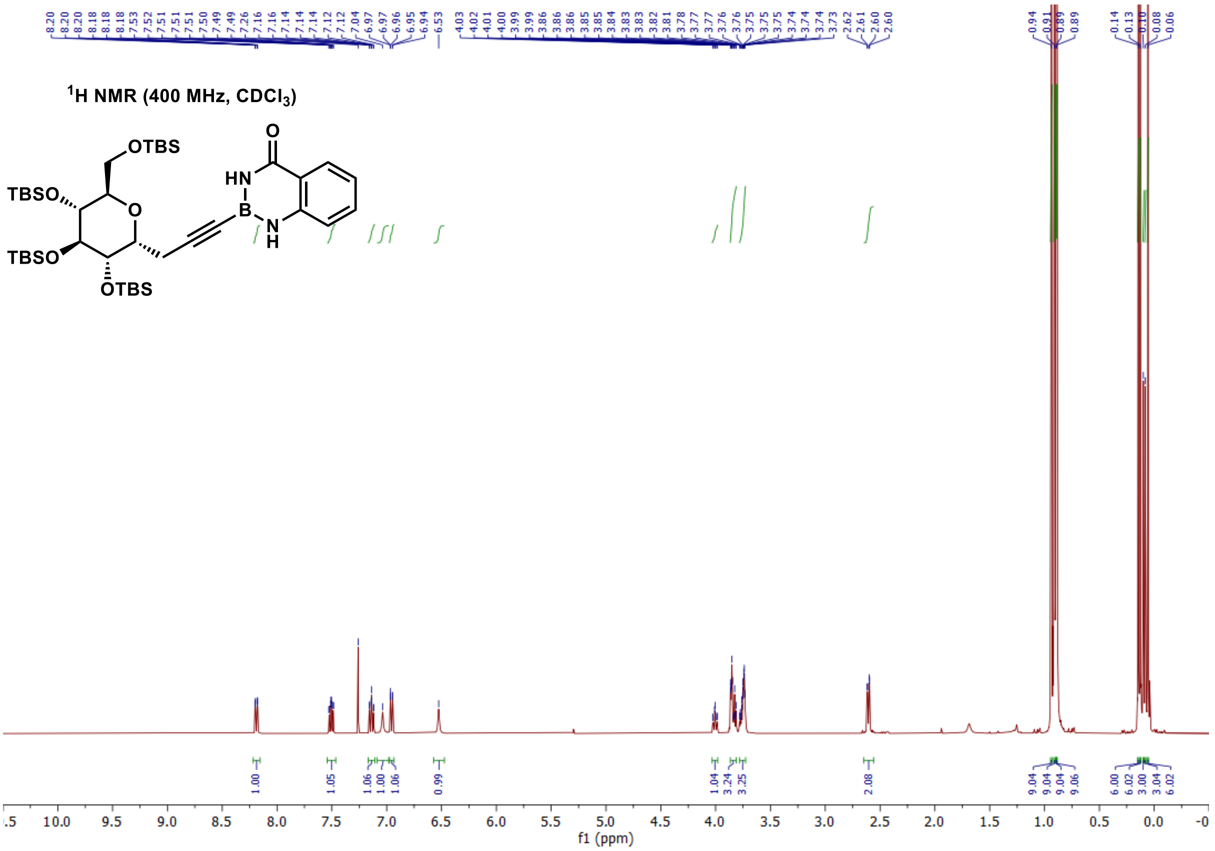
1H NMR (400 MHz, CDCl3)
OTBS
TBSO
TBSO
OTBS
O
HN
B
N
H
O
f1 (ppm)

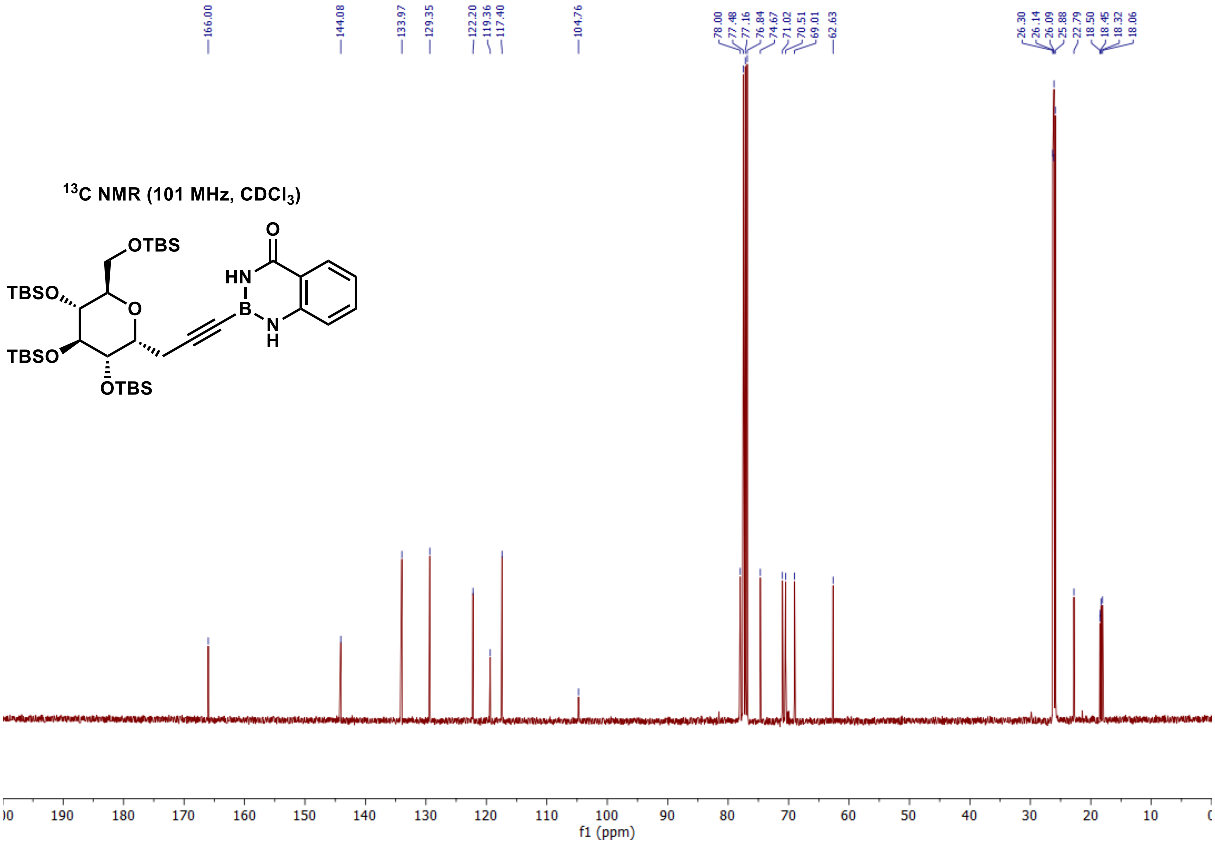
13C NMR (101 MHz, CDCl3)
OTBS
TBSO
TBSO
OTBS
O
HN
B
N
H
O
f1 (ppm)

^{11}B NMR (128 MHz, $CDCl_3$)

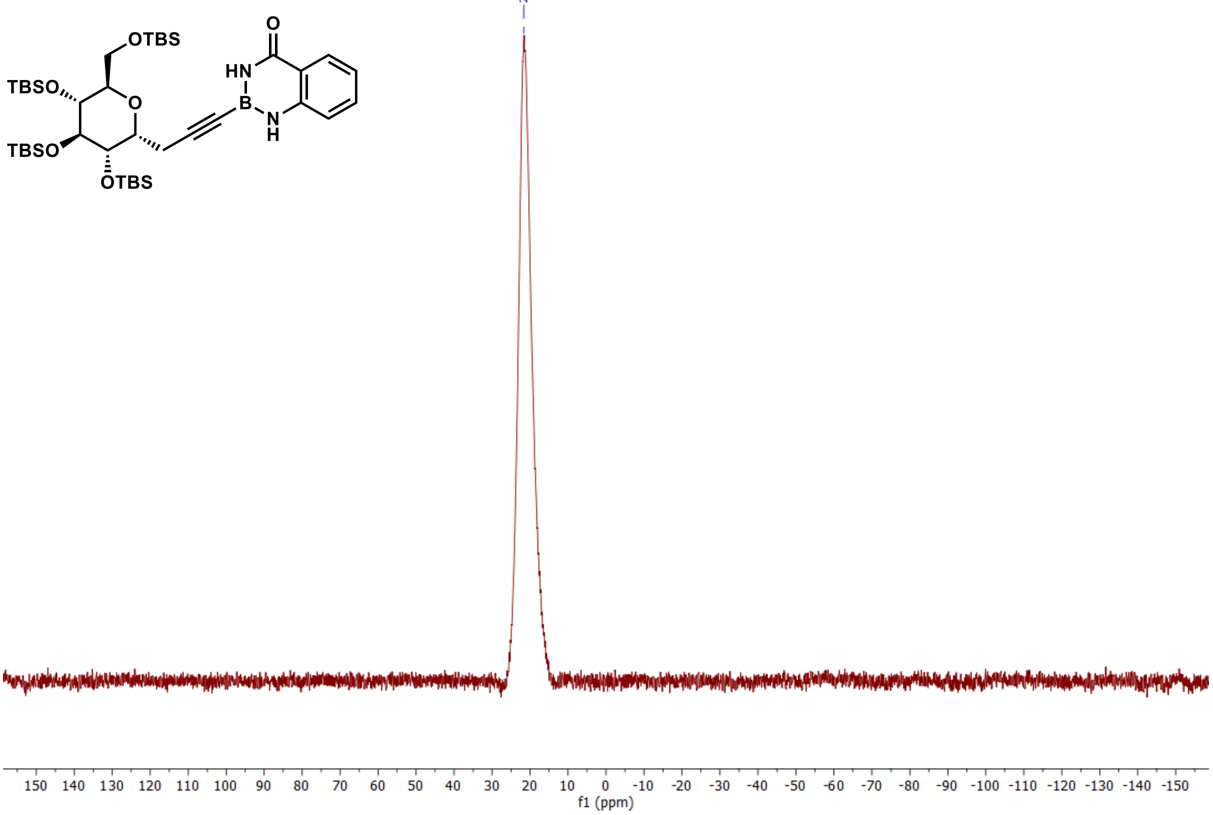

^{1}H NMR (400 MHz, $CDCl_3$)

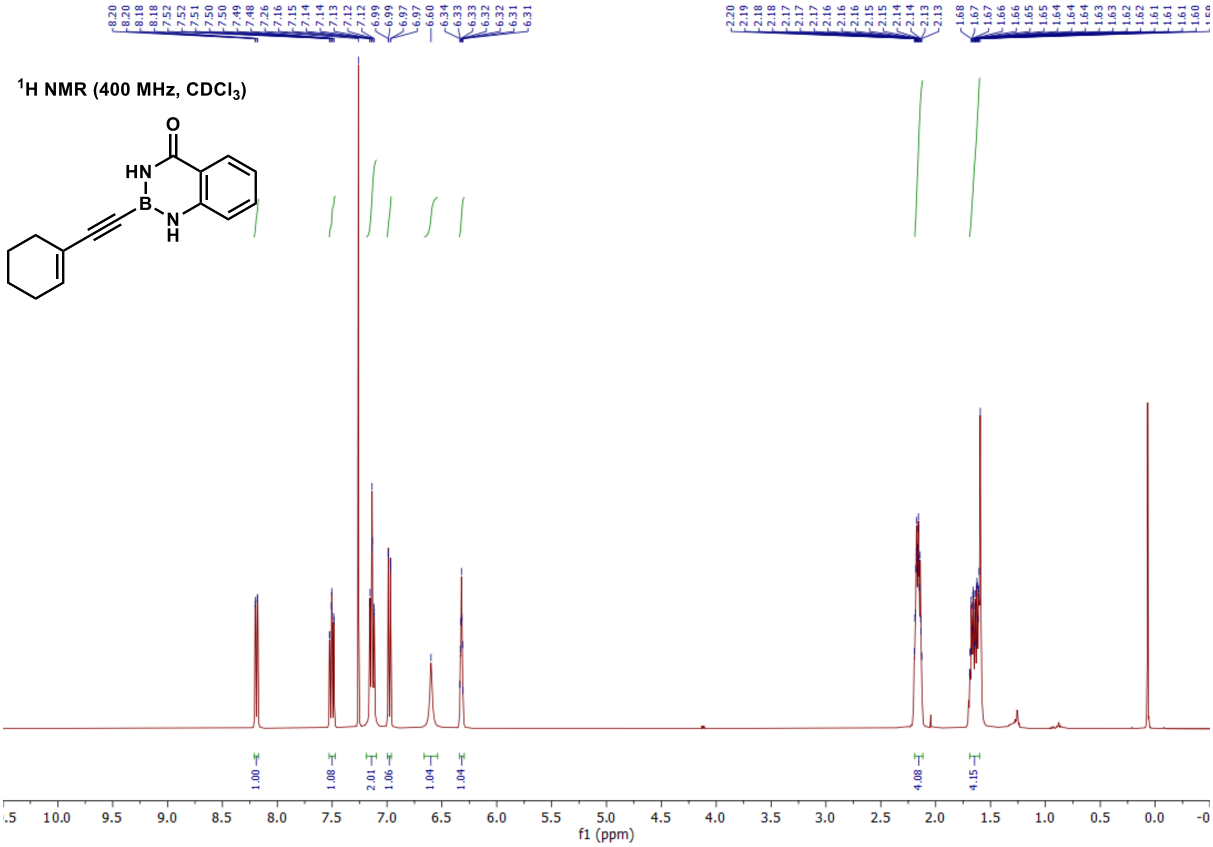

^{13}C NMR (101 MHz, $CDCl_3$)

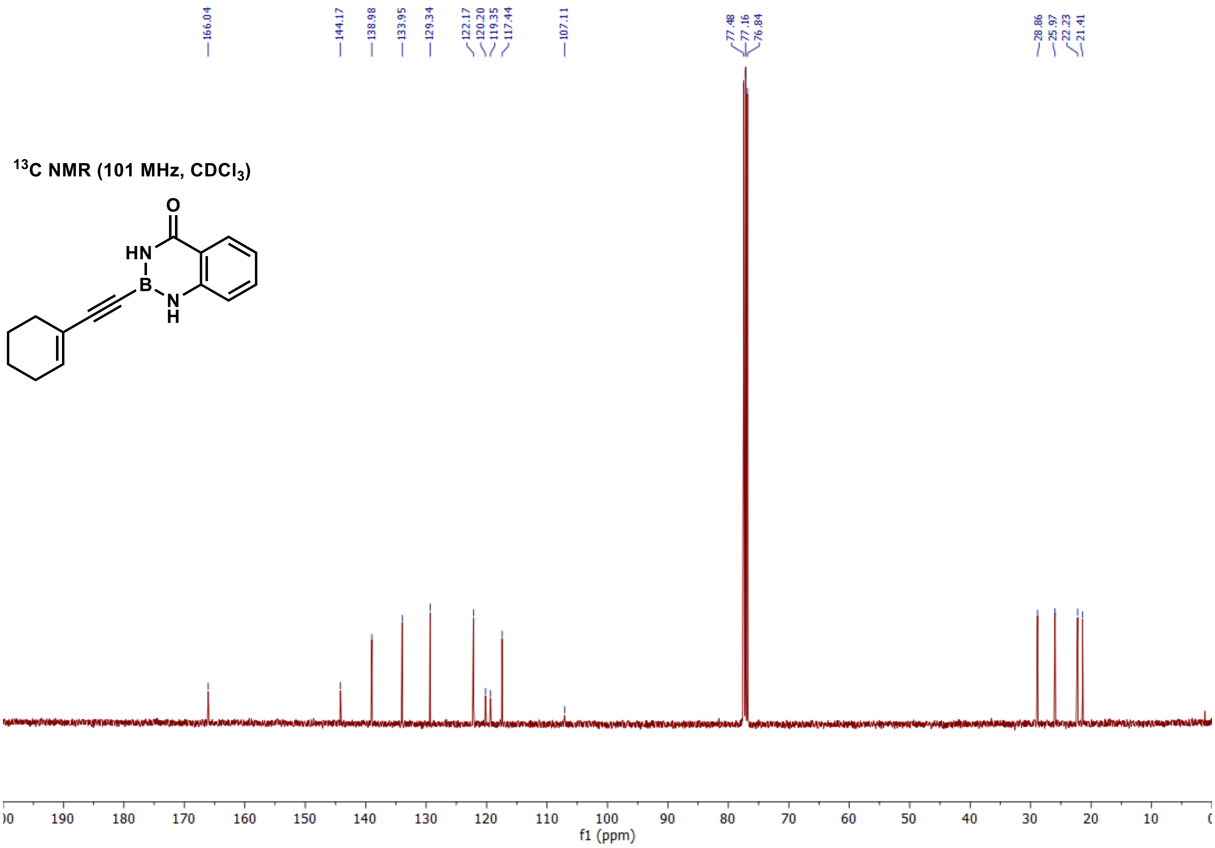

^{11}B NMR (128 MHz, $CDCl_3$)

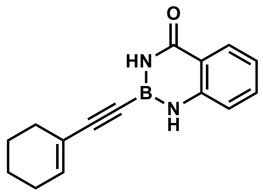

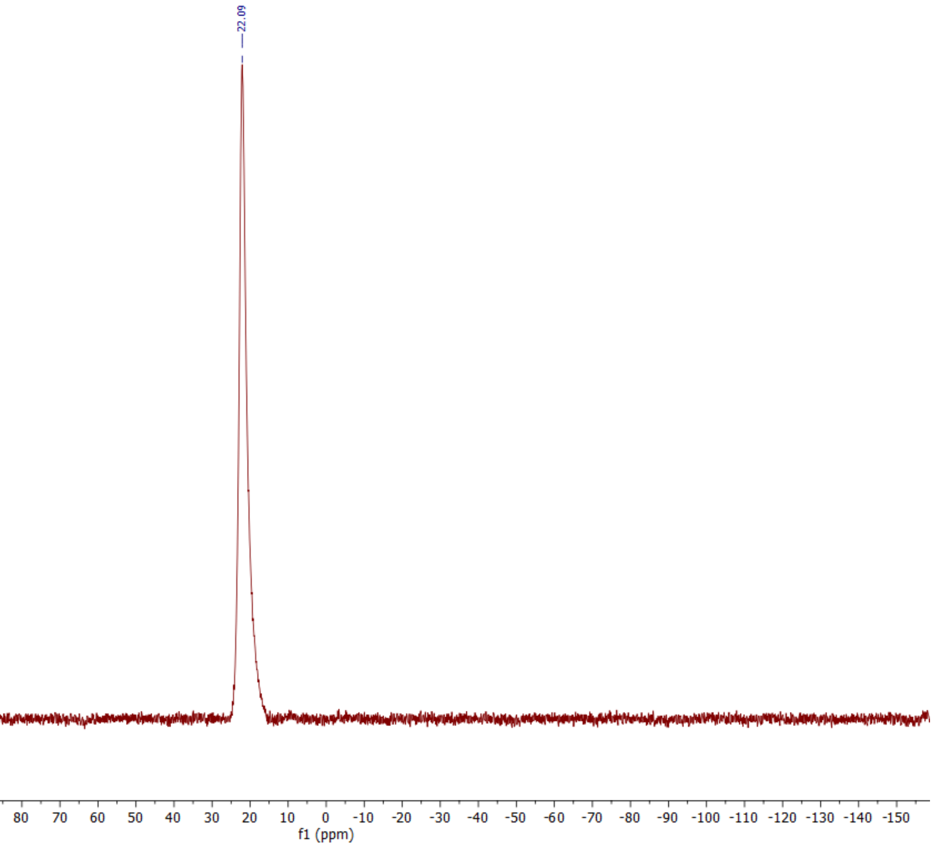

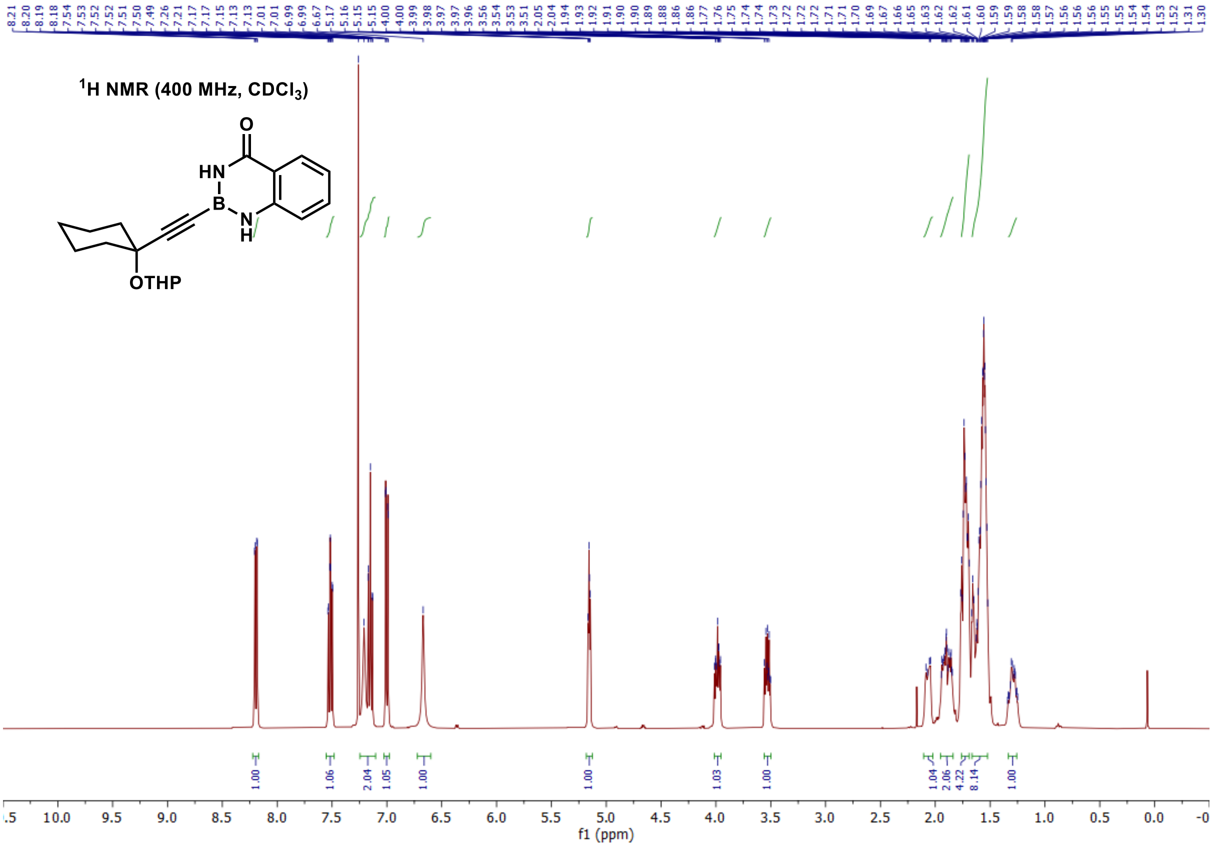
1H NMR (400 MHz, CDCl3)
O
HN
B
N
H
OTHP
f1 (ppm)

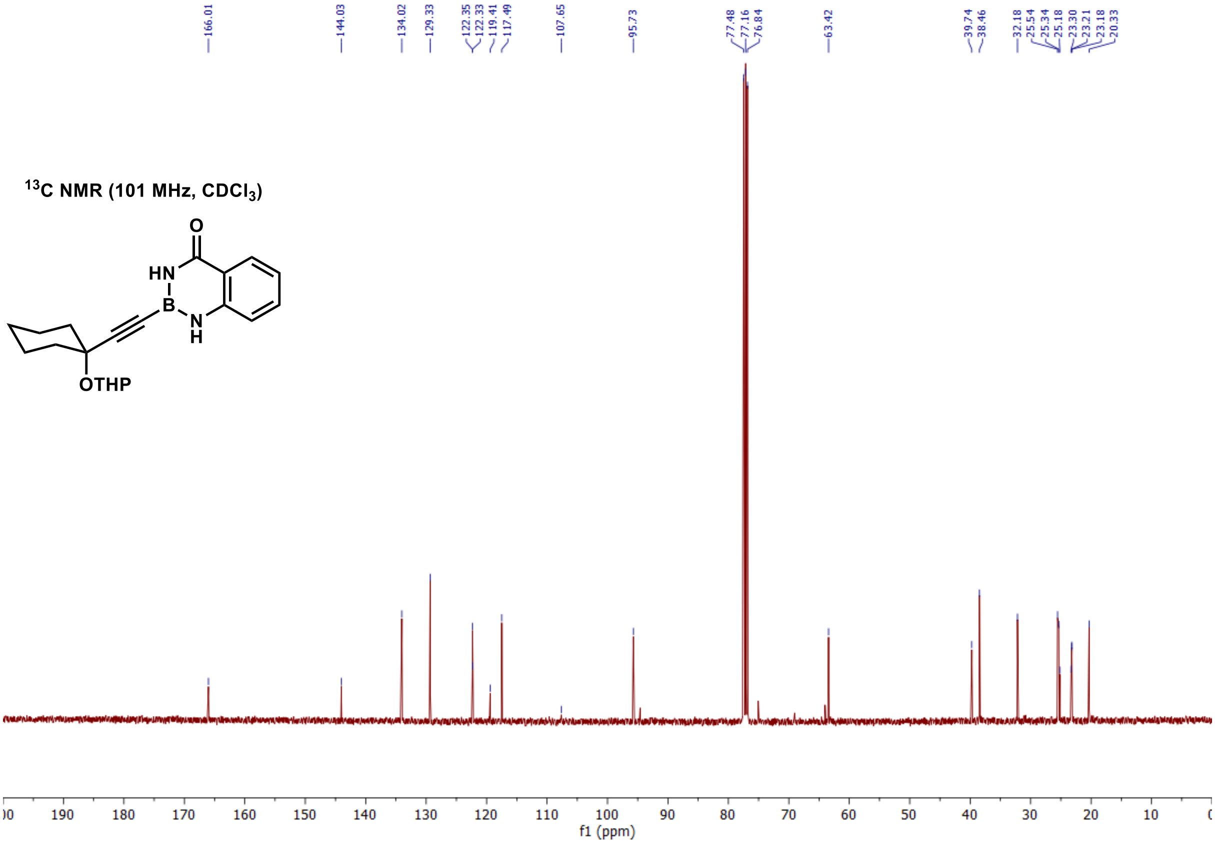
13C NMR (101 MHz, CDCl3)
O
HN
B
N
H
OTHP
f1 (ppm)

^{11}B NMR (128 MHz, $CDCl_3$)

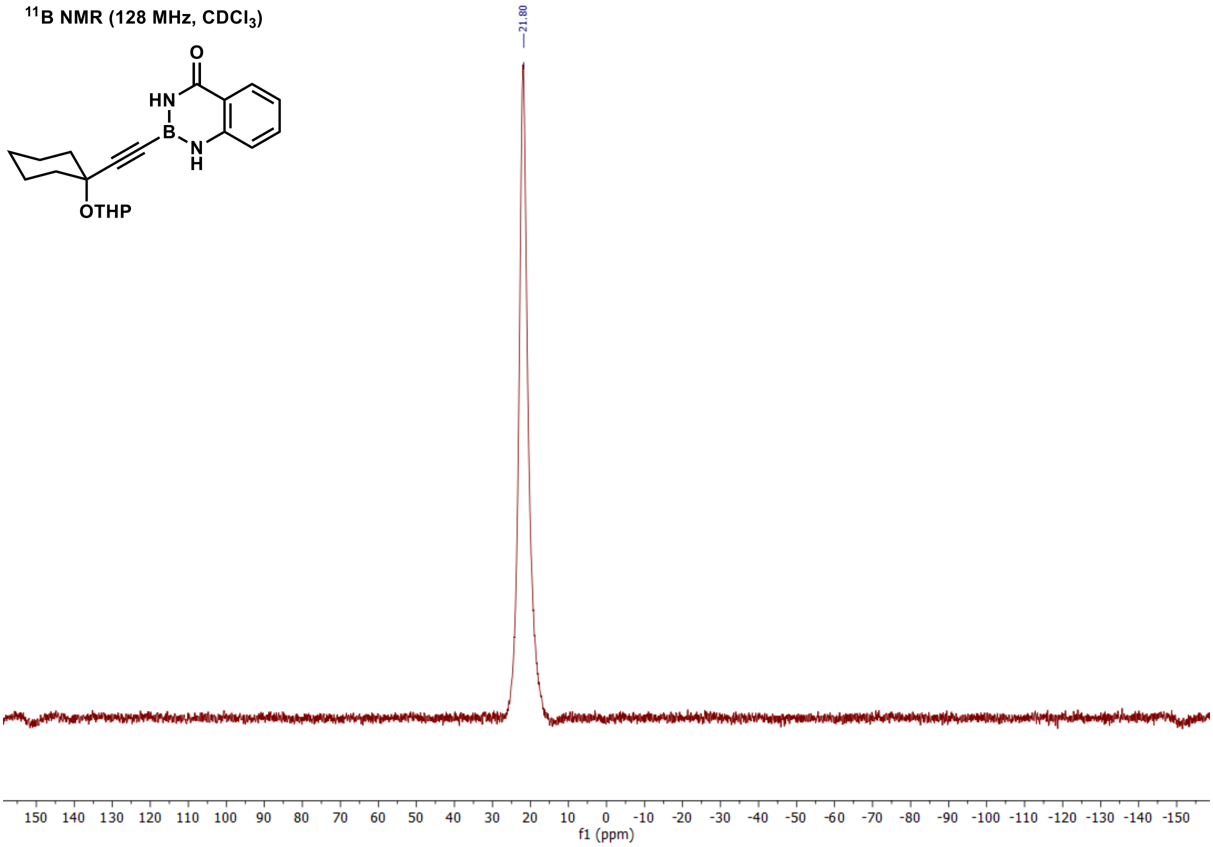

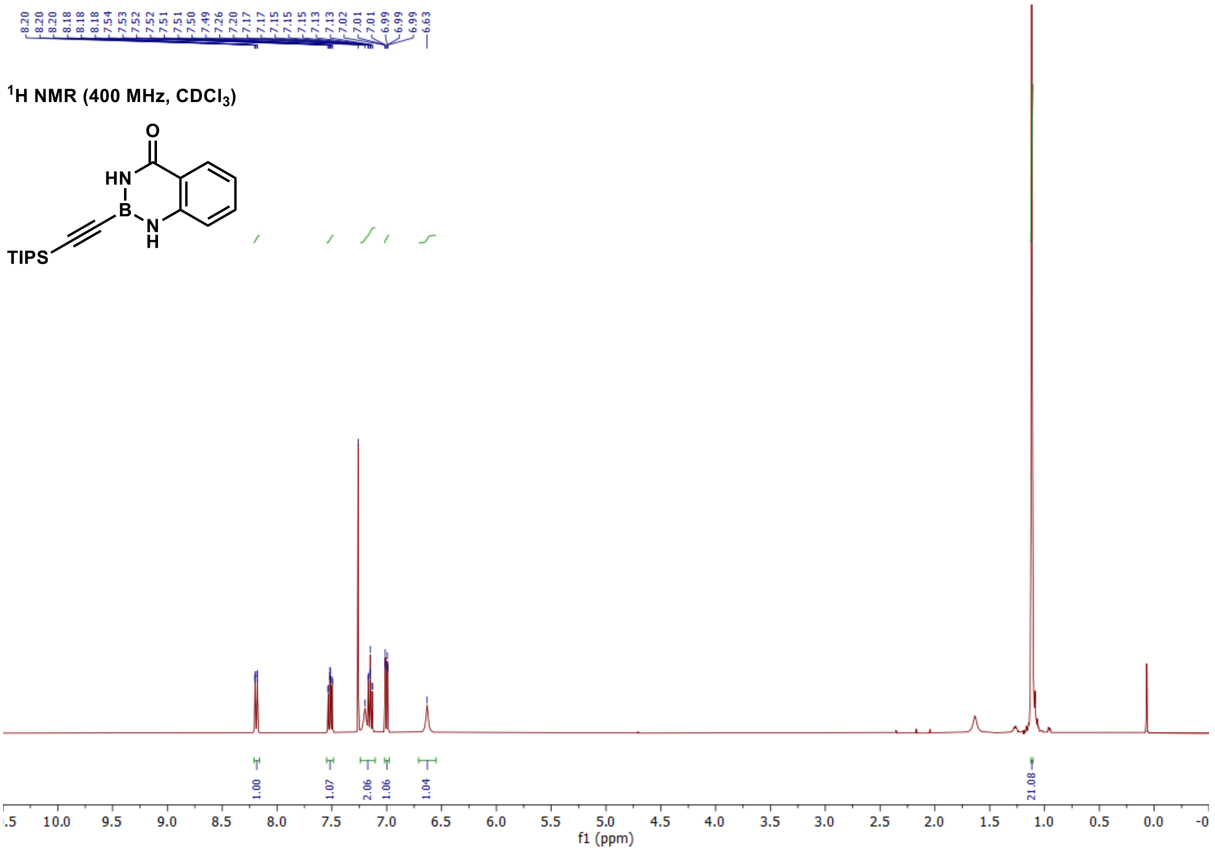
8.20
8.20
8.20
8.18
8.18
8.18
7.54
7.53
7.52
7.52
7.51
7.51
7.50
7.49
7.26
7.20
7.17
7.17
7.15
7.15
7.15
7.13
7.13
7.02
7.01
7.01
6.99
6.99
6.99
6.63
1H NMR (400 MHz, CDCl3)
O
HN
B
N
H
TIPS
1.00
1.07
2.06
1.06
1.04
21.08
f1 (ppm)

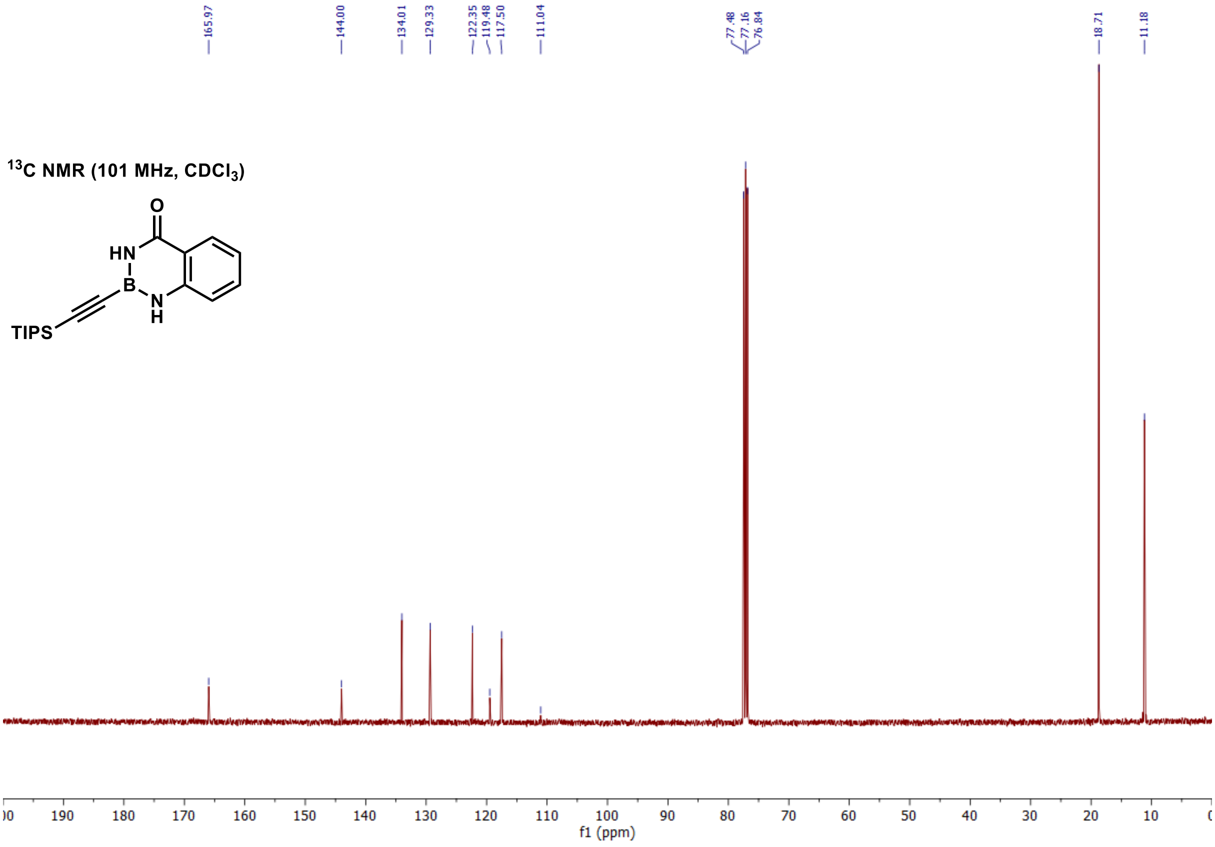
165.97
144.00
134.01
129.33
122.35
119.48
117.50
111.04
77.48
77.16
76.84
18.71
11.18
13C NMR (101 MHz, CDCl3)
O
HN
B
N
H
TIPS
f1 (ppm)

^{11}B NMR (128 MHz, $CDCl_3$)

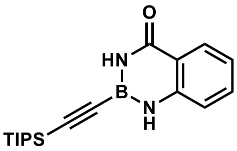

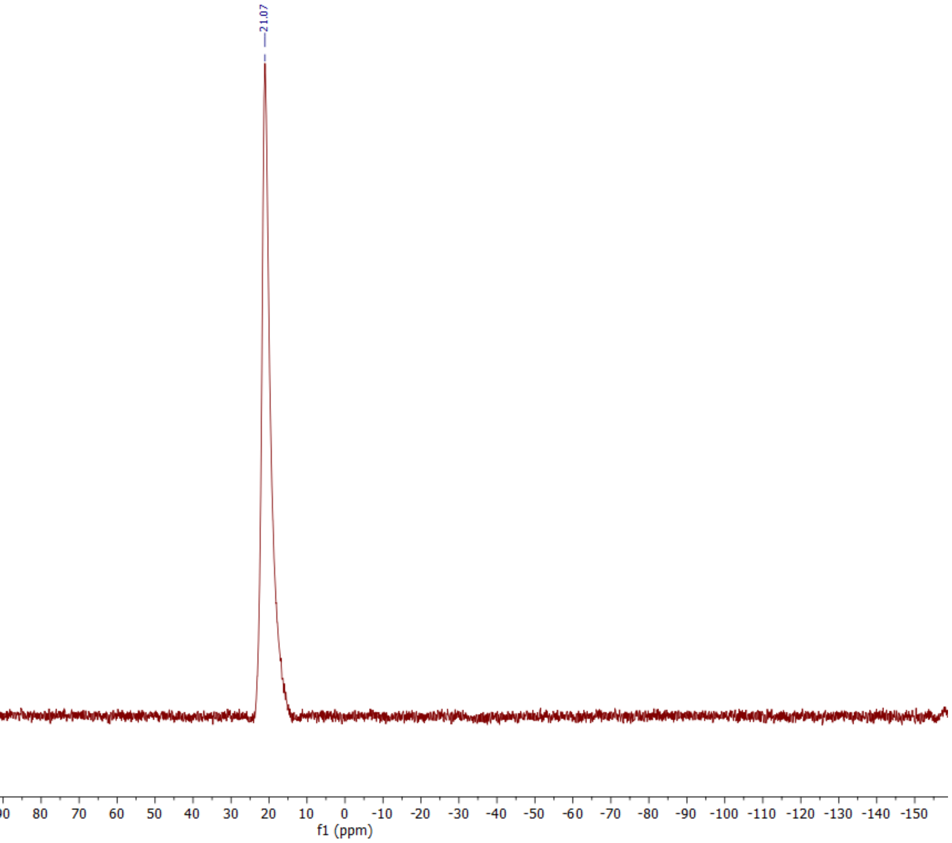

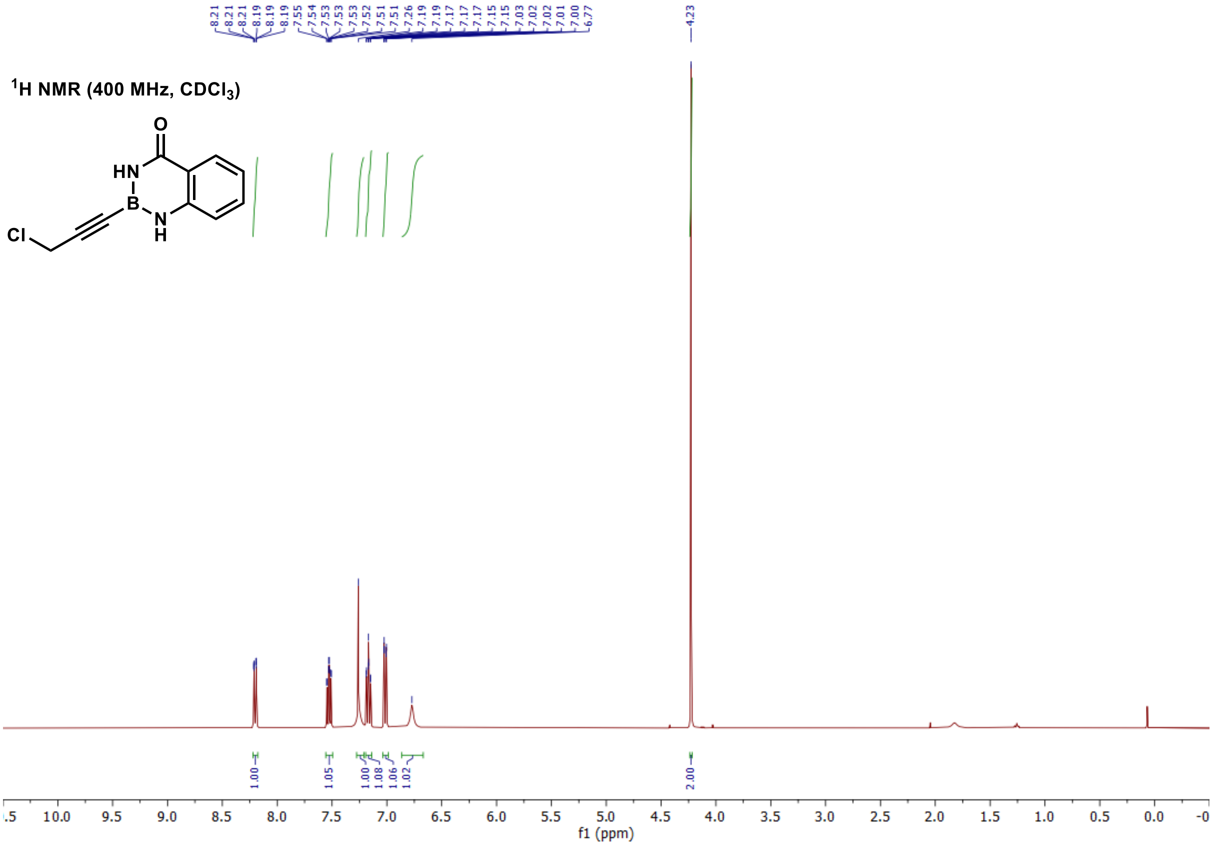
1H NMR (400 MHz, CDCl3)
f1 (ppm)

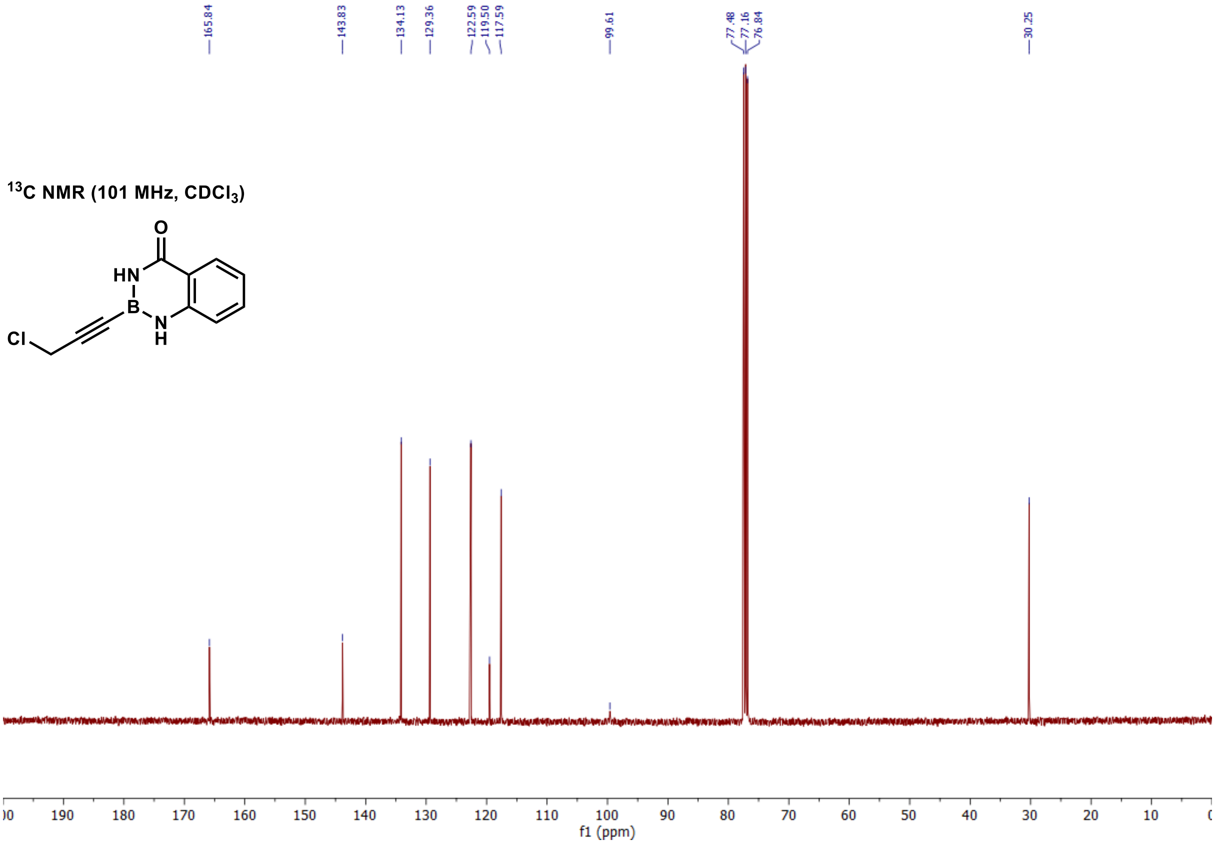
13C NMR (101 MHz, CDCl3)
f1 (ppm)

^{11}B NMR (128 MHz, $CDCl_3$)

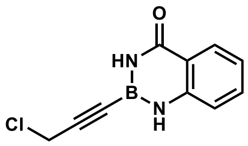

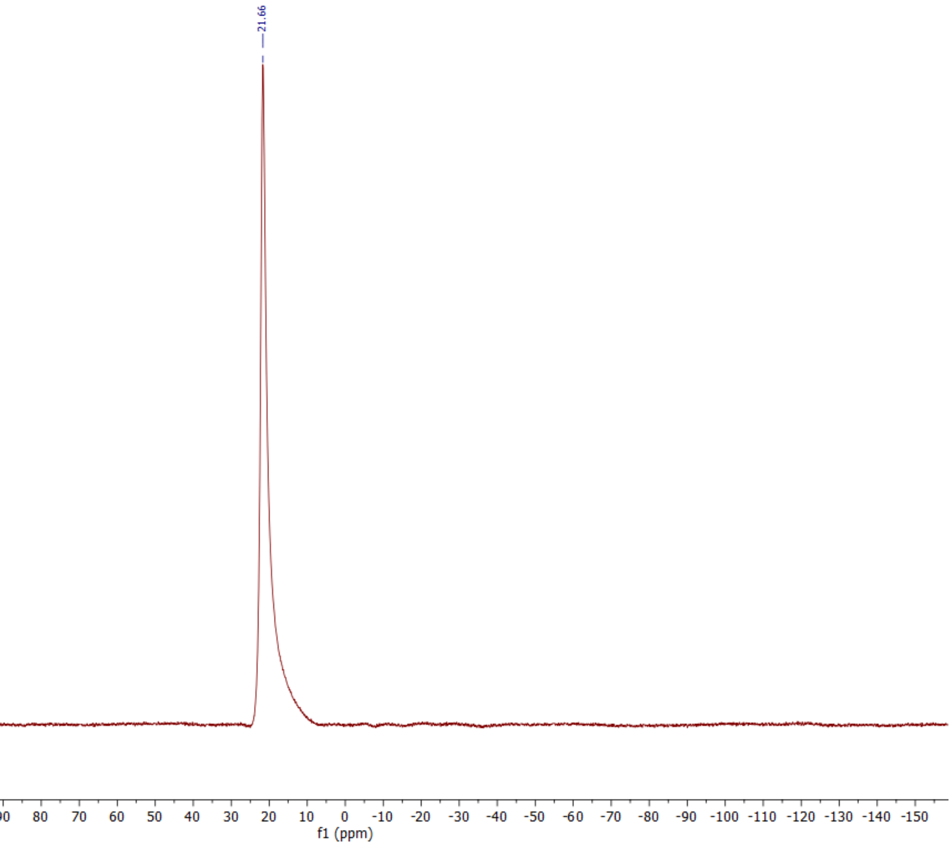

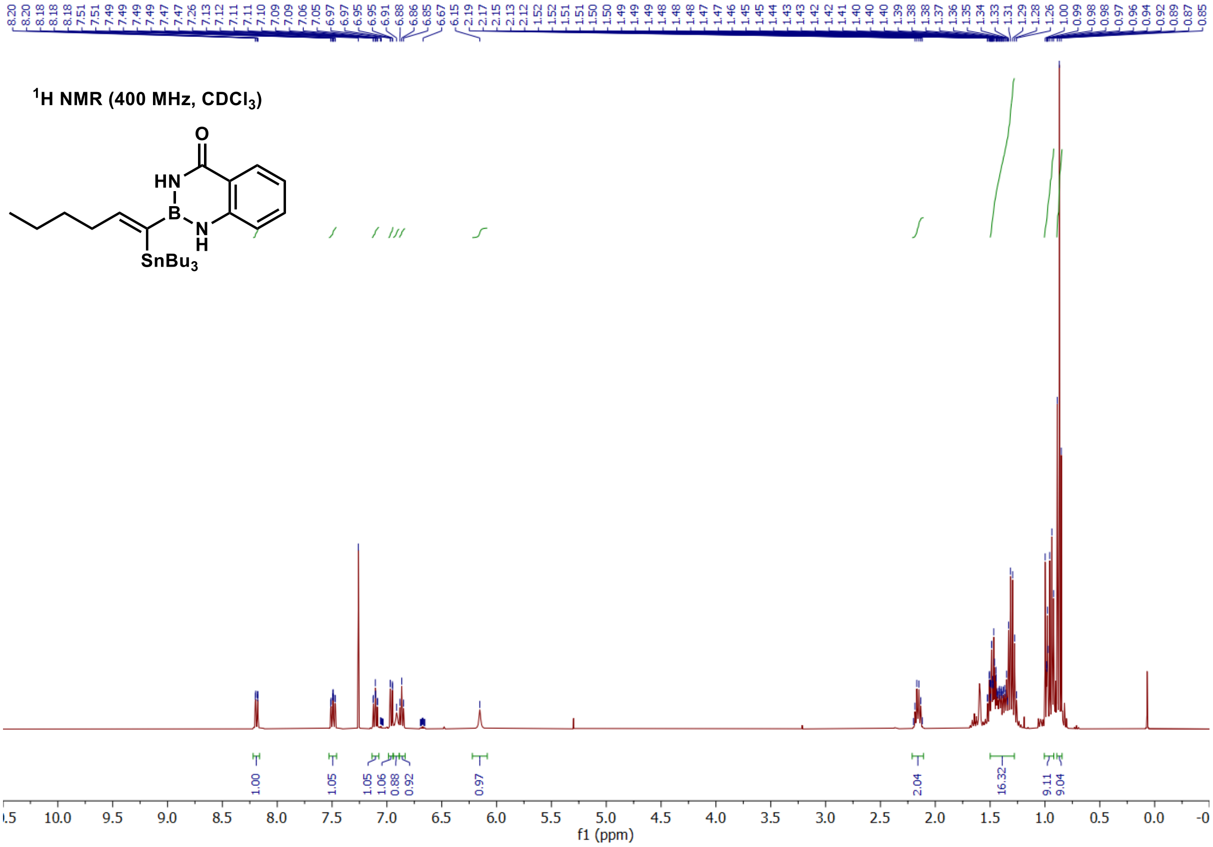

^{1}H NMR (400 MHz, CDCl$_3$)
O
HN
B
N
H
SnBu$_3$
1.00
1.05
1.05
1.06
0.88
0.92
0.97
2.04
16.32
9.11
9.04
f1 (ppm)

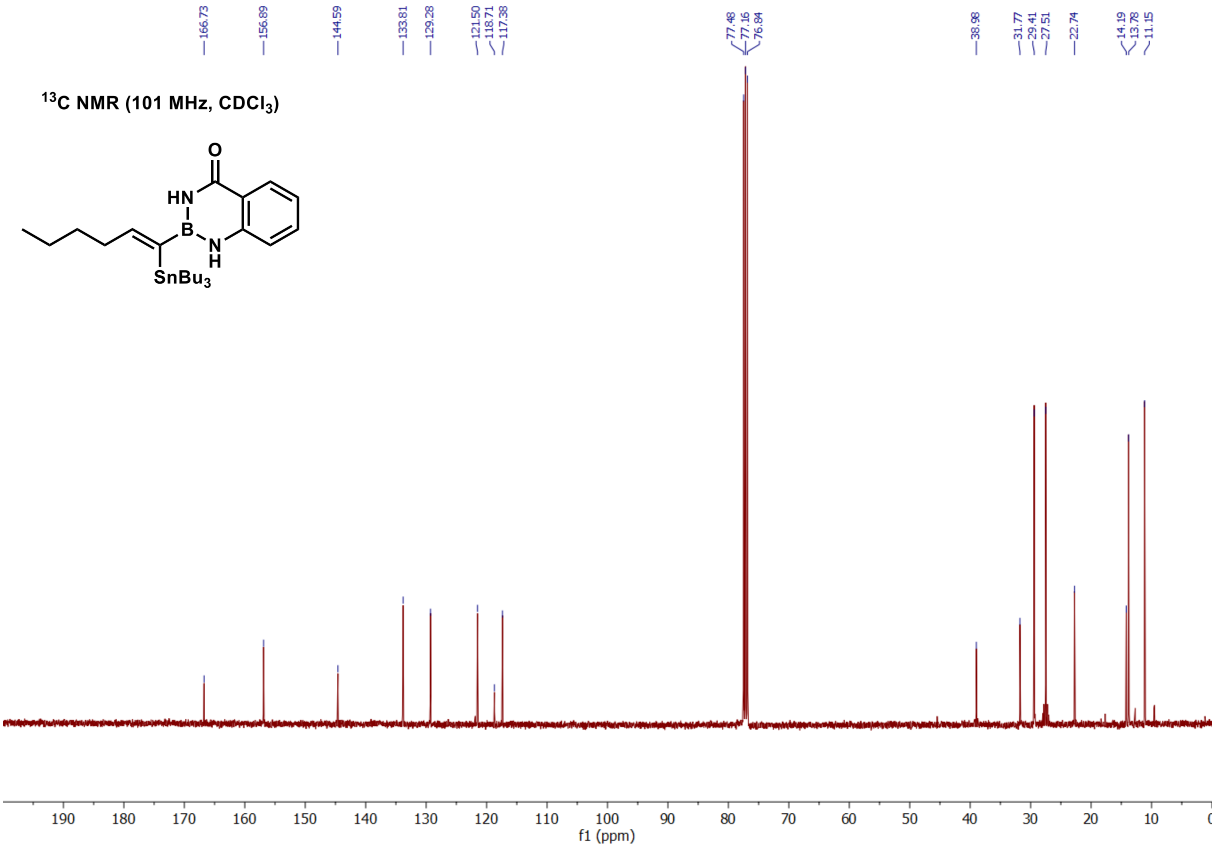

^{13}C NMR (101 MHz, CDCl$_3$)
166.73
156.89
144.59
133.81
129.28
121.50
118.71
117.38
77.48
77.16
76.84
38.98
31.77
29.41
27.51
22.74
14.19
13.78
11.15
O
HN
B
N
H
SnBu$_3$
f1 (ppm)

^{11}B NMR (128 MHz, $CDCl_3$)

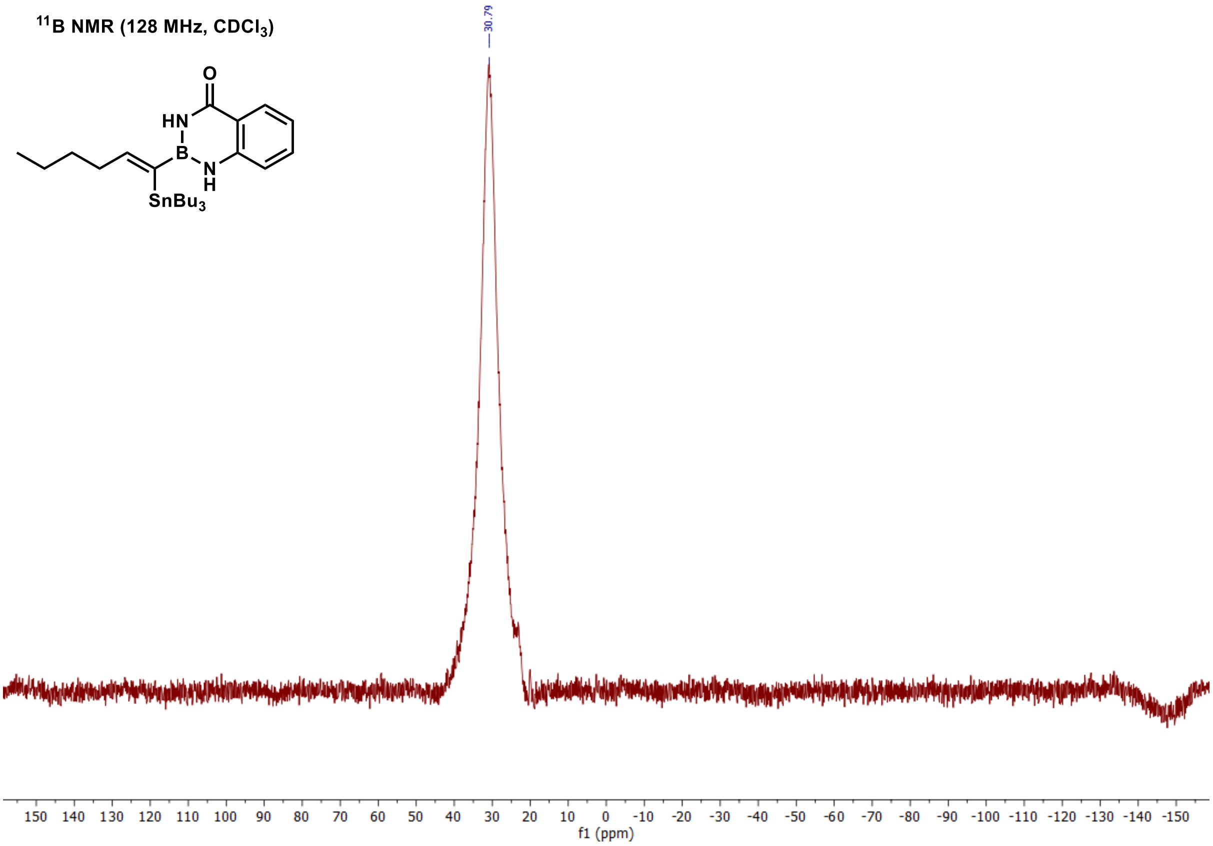

^{119}Sn NMR (149 MHz, $CDCl_3$)

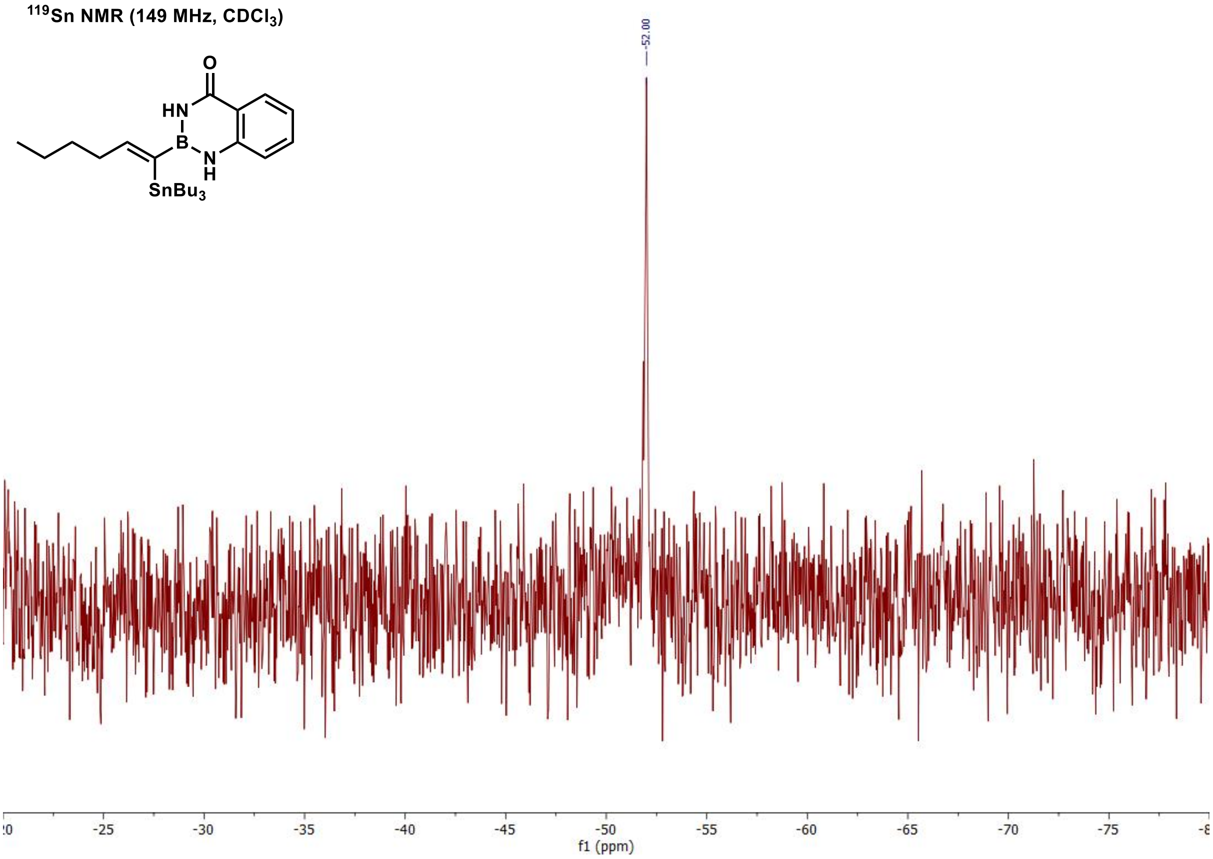

^{1}H NMR (400 MHz, $CDCl_3$)

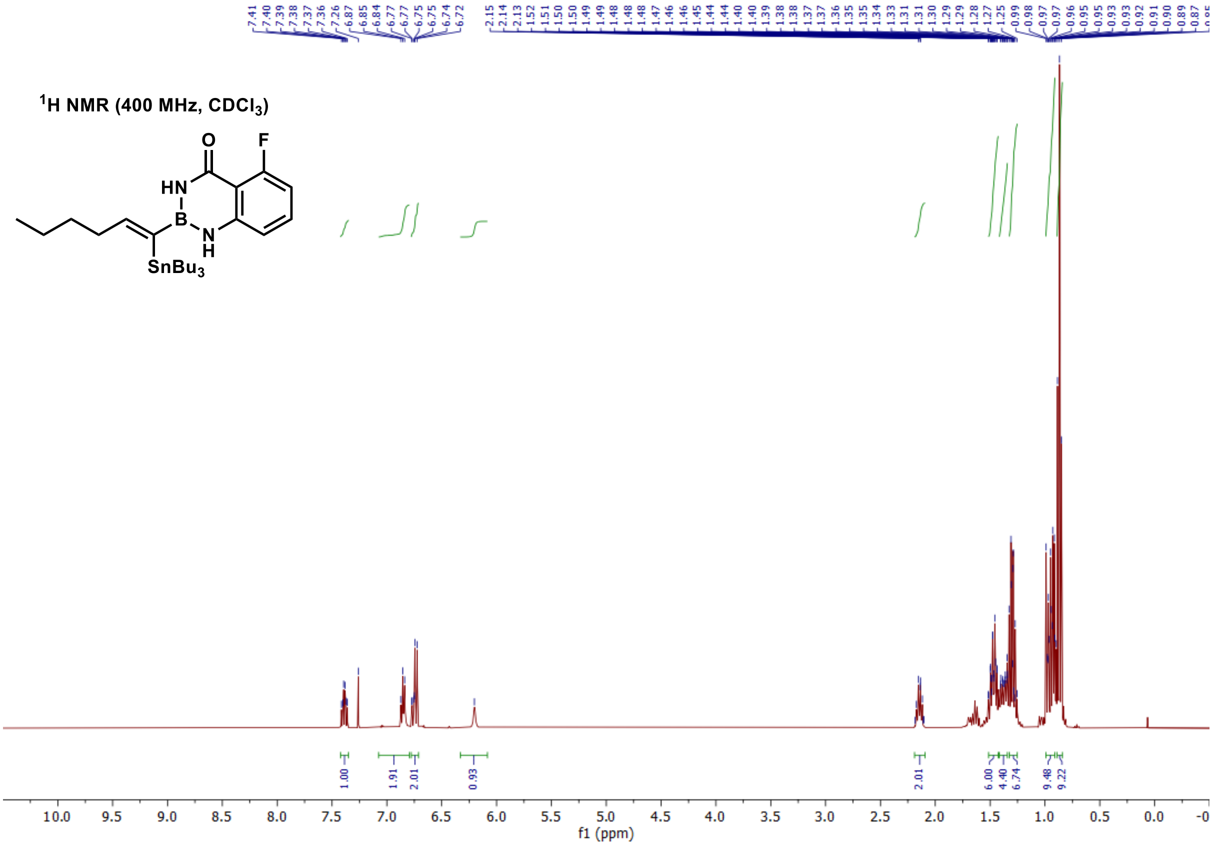

^{13}C NMR (101 MHz, $CDCl_3$)

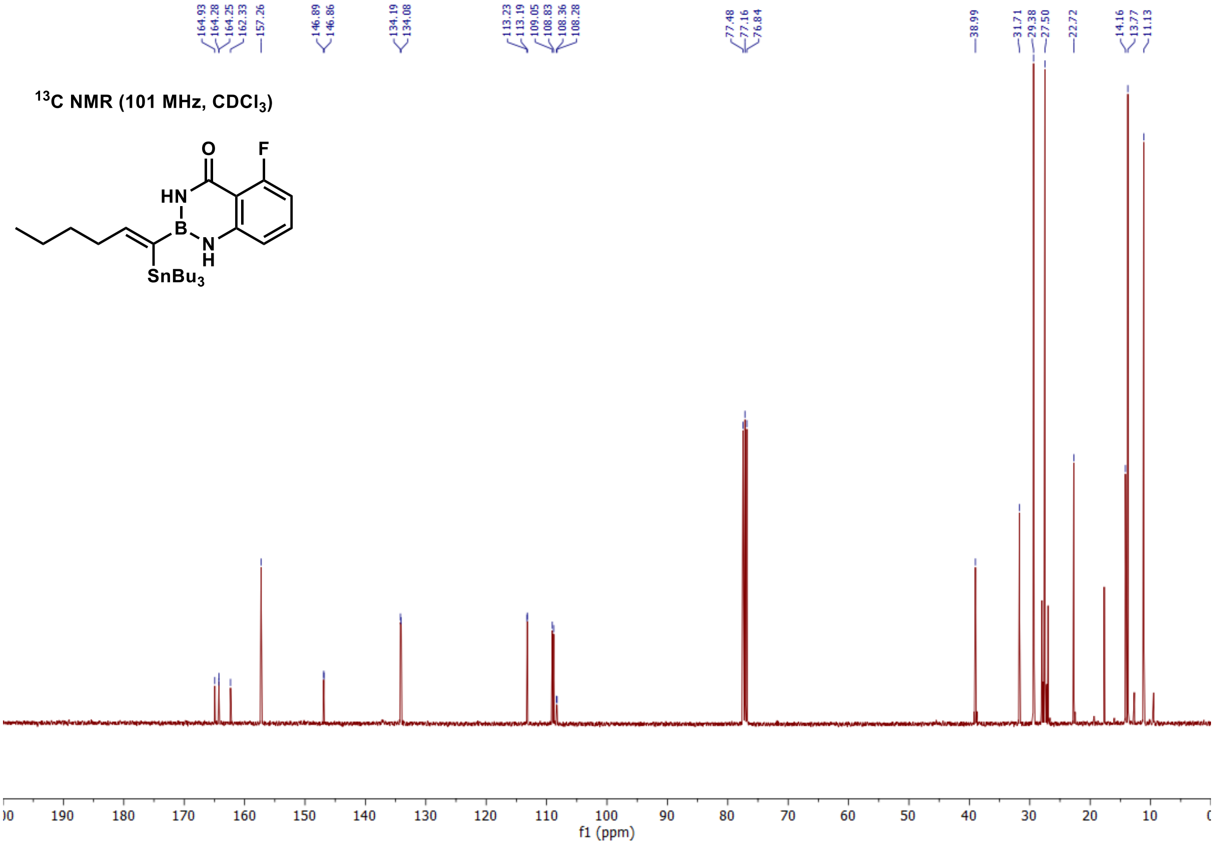

^{11}B NMR (128 MHz, $CDCl_3$)

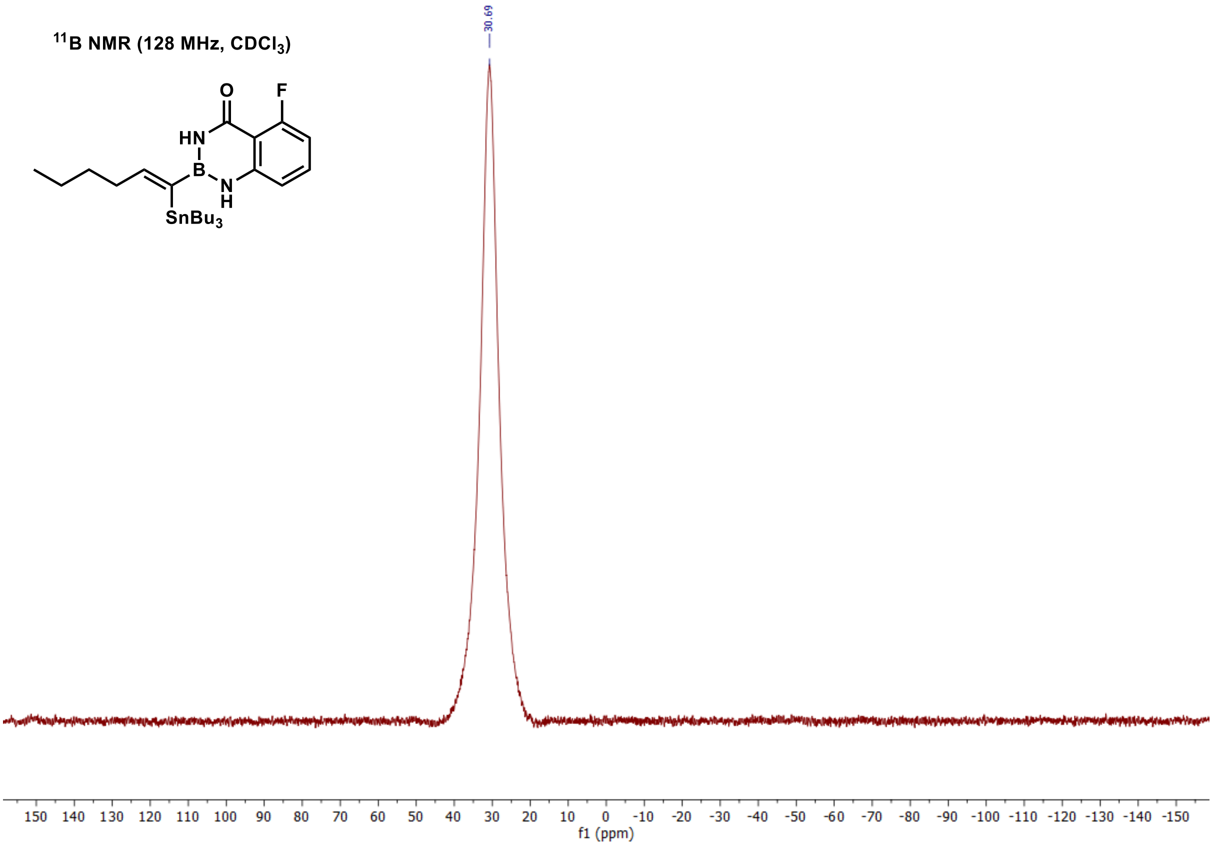

^{19}F NMR (282 MHz, $CDCl_3$)

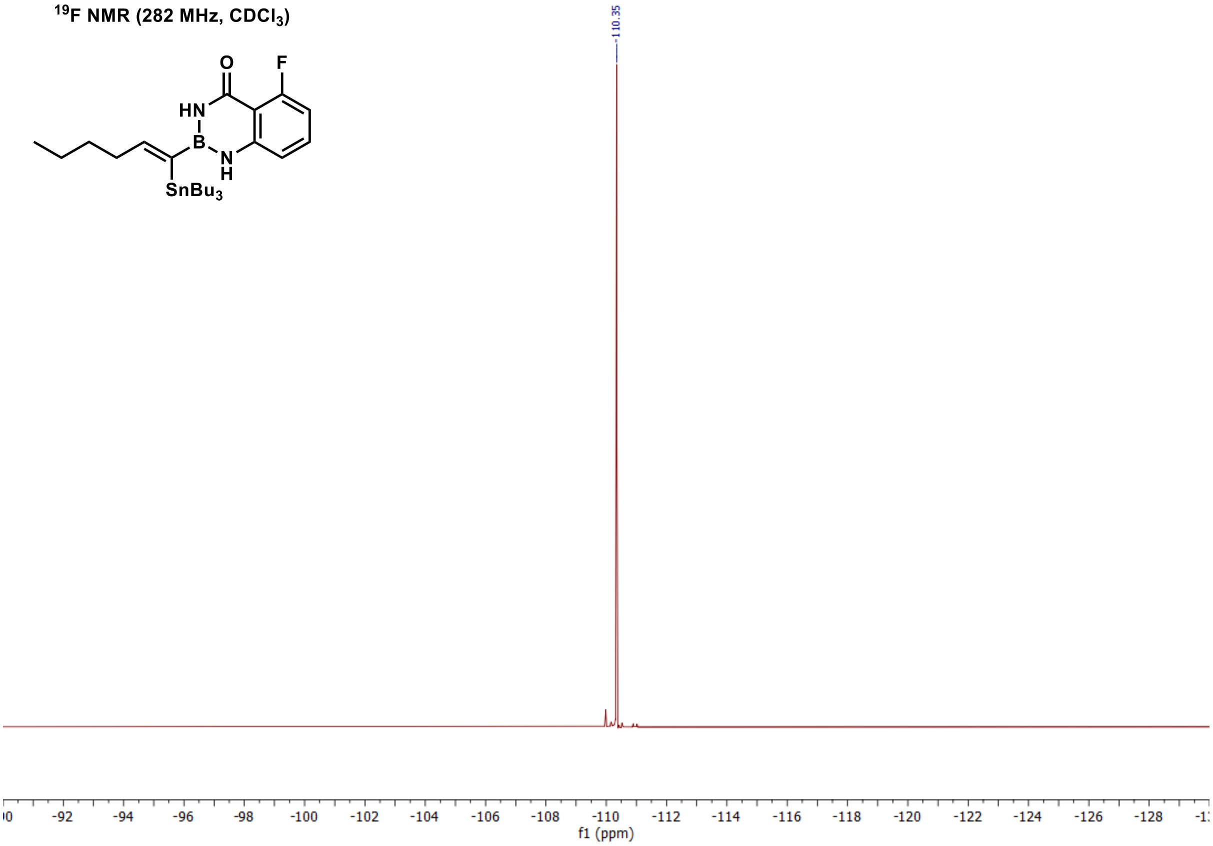

^{119}Sn NMR (149 MHz, $CDCl_3$)

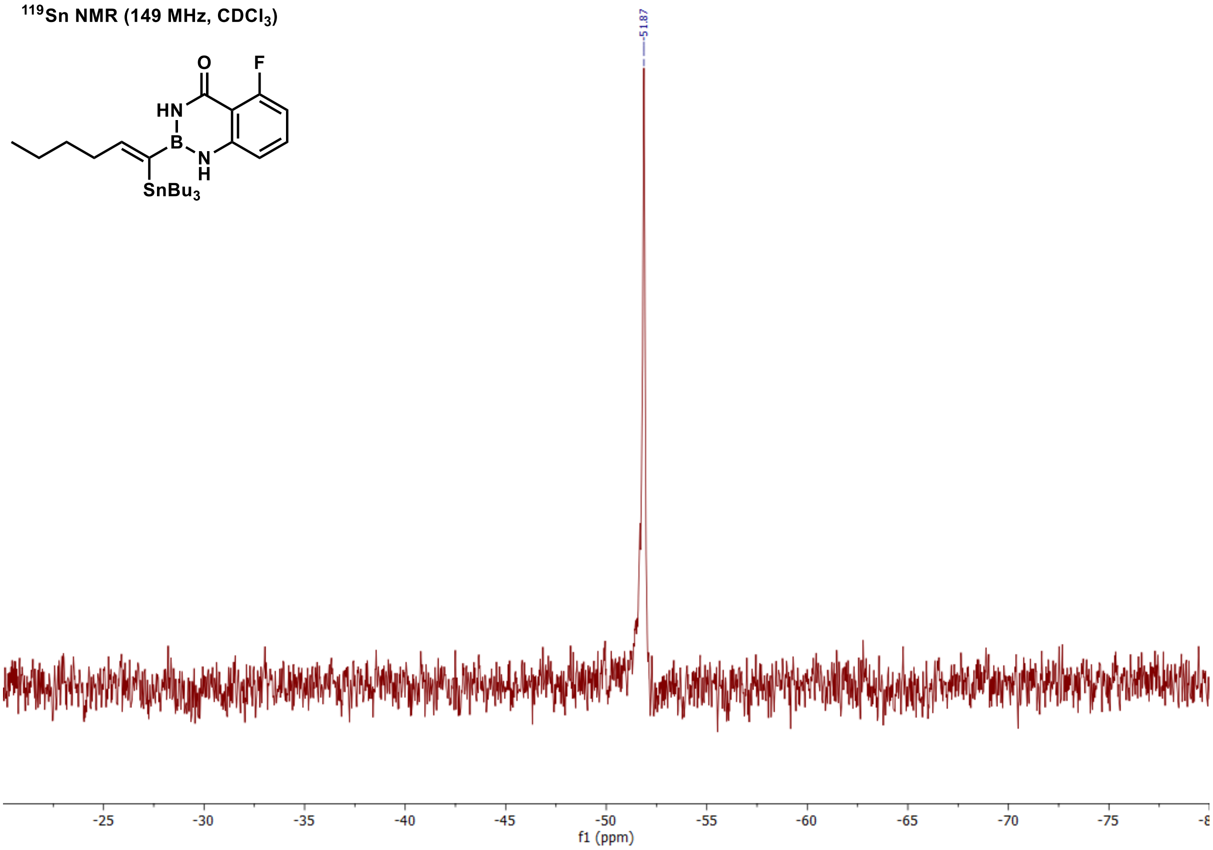

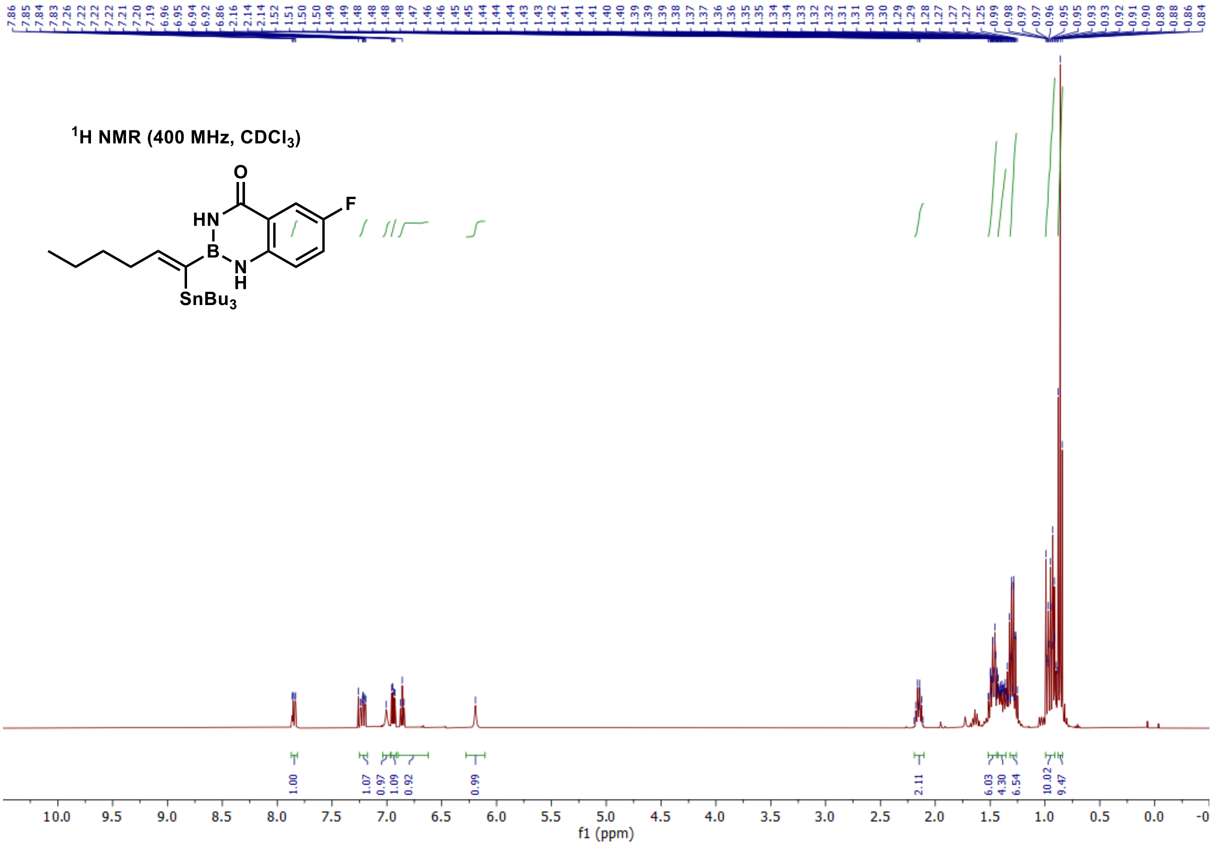

1H NMR (400 MHz, CDCl3)
HN
B
N
H
O
F
SnBu3
f1 (ppm)

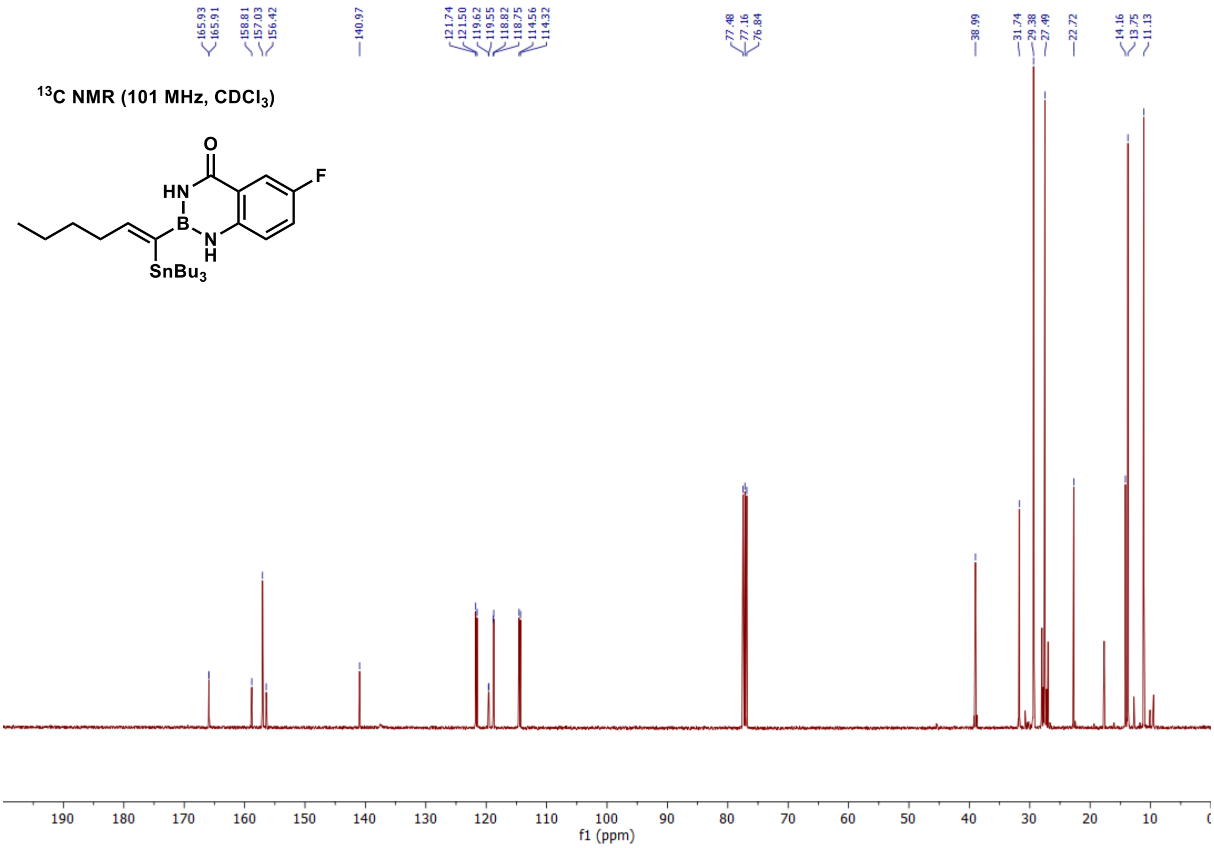

13C NMR (101 MHz, CDCl3)
HN
B
N
H
O
F
SnBu3
f1 (ppm)

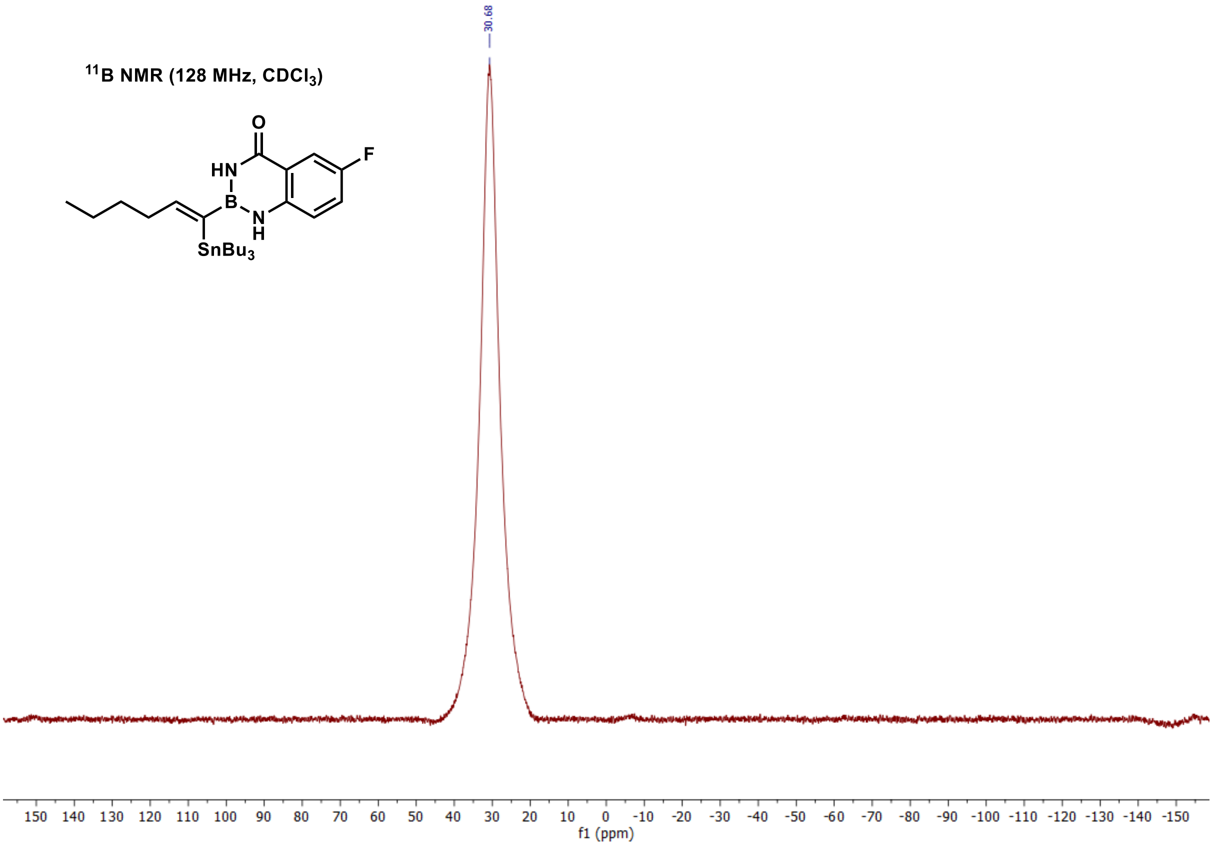

30.68
11B NMR (128 MHz, CDCl3)
O
HN
B
N
H
F
SnBu3
150 140 130 120 110 100 90 80 70 60 50 40 30 20 10 0 -10 -20 -30 -40 -50 -60 -70 -80 -90 -100 -110 -120 -130 -140 -150
f1 (ppm)

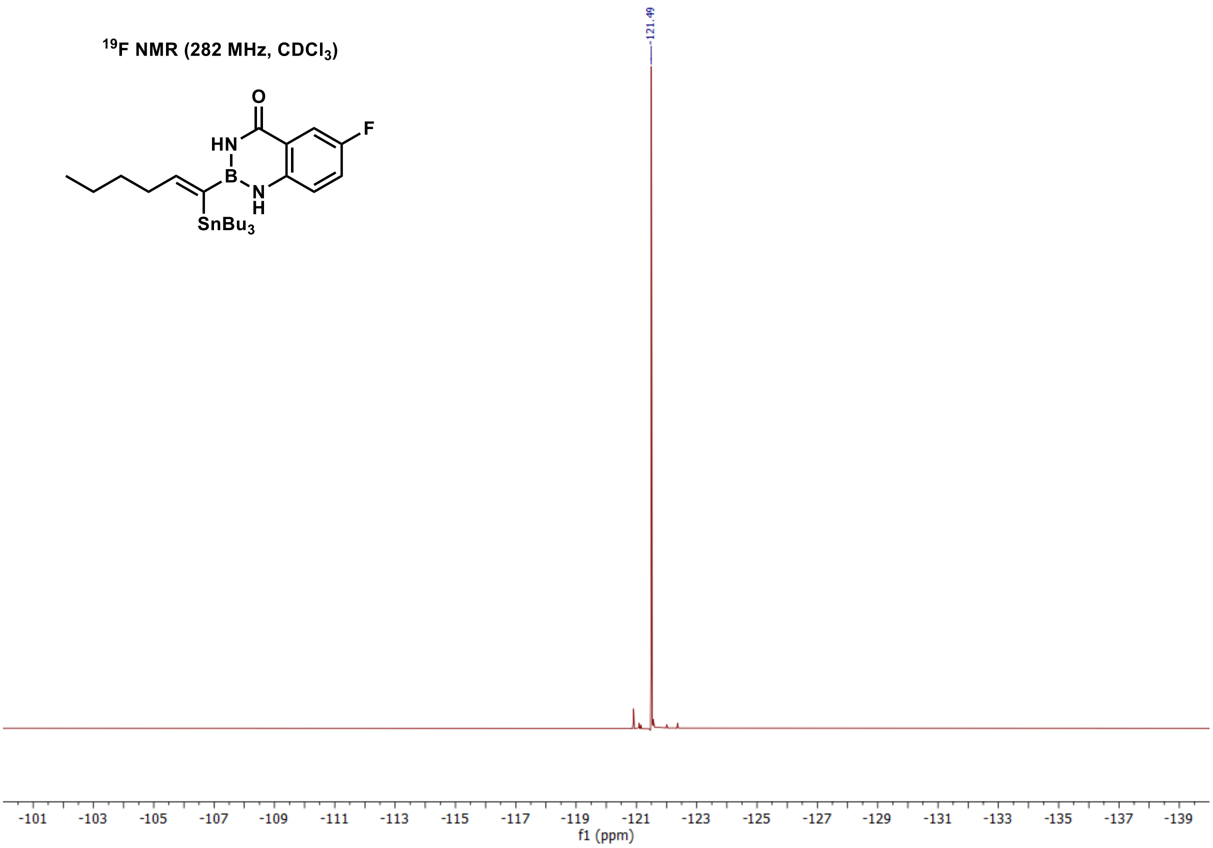

-121.49
19F NMR (282 MHz, CDCl3)
O
HN
B
N
H
F
SnBu3
-101 -103 -105 -107 -109 -111 -113 -115 -117 -119 -121 -123 -125 -127 -129 -131 -133 -135 -137 -139
f1 (ppm)

^{119}Sn NMR (149 MHz, $CDCl_3$)

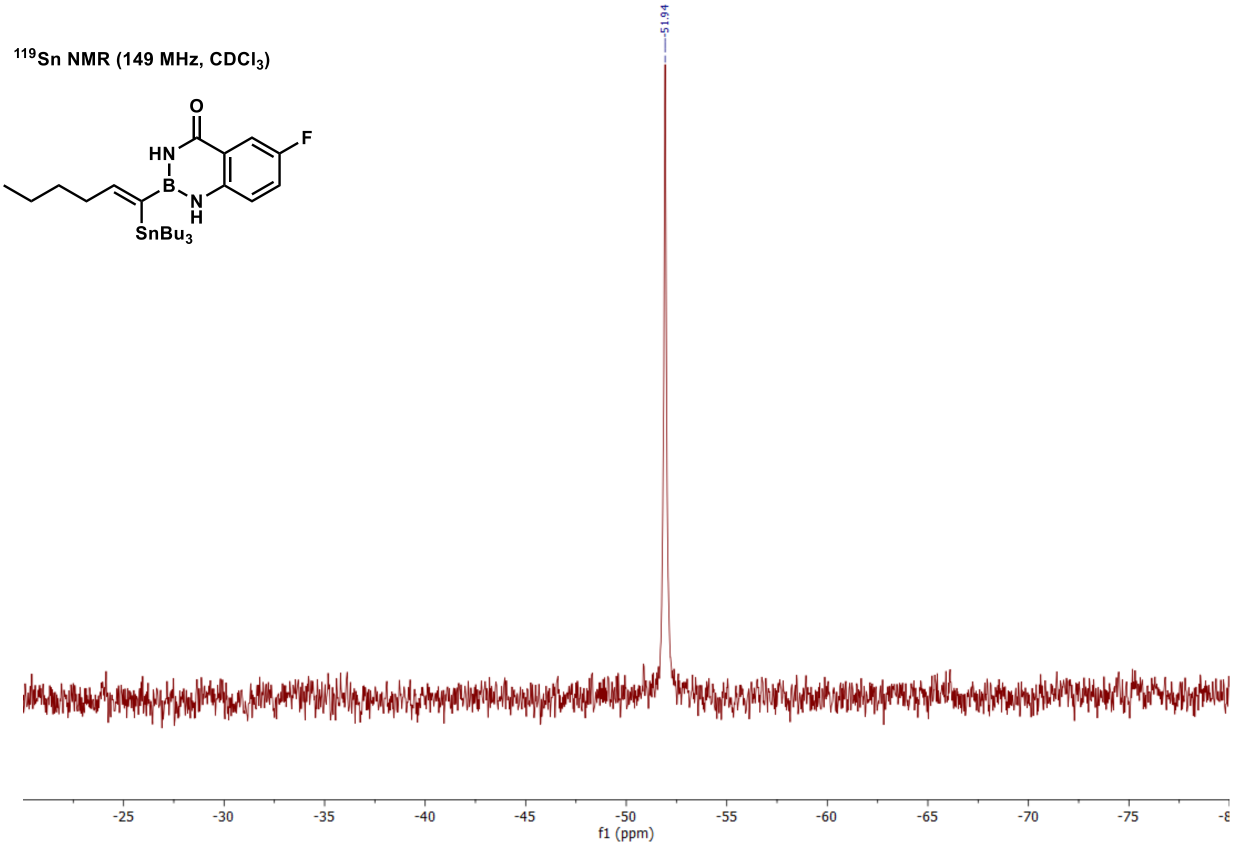

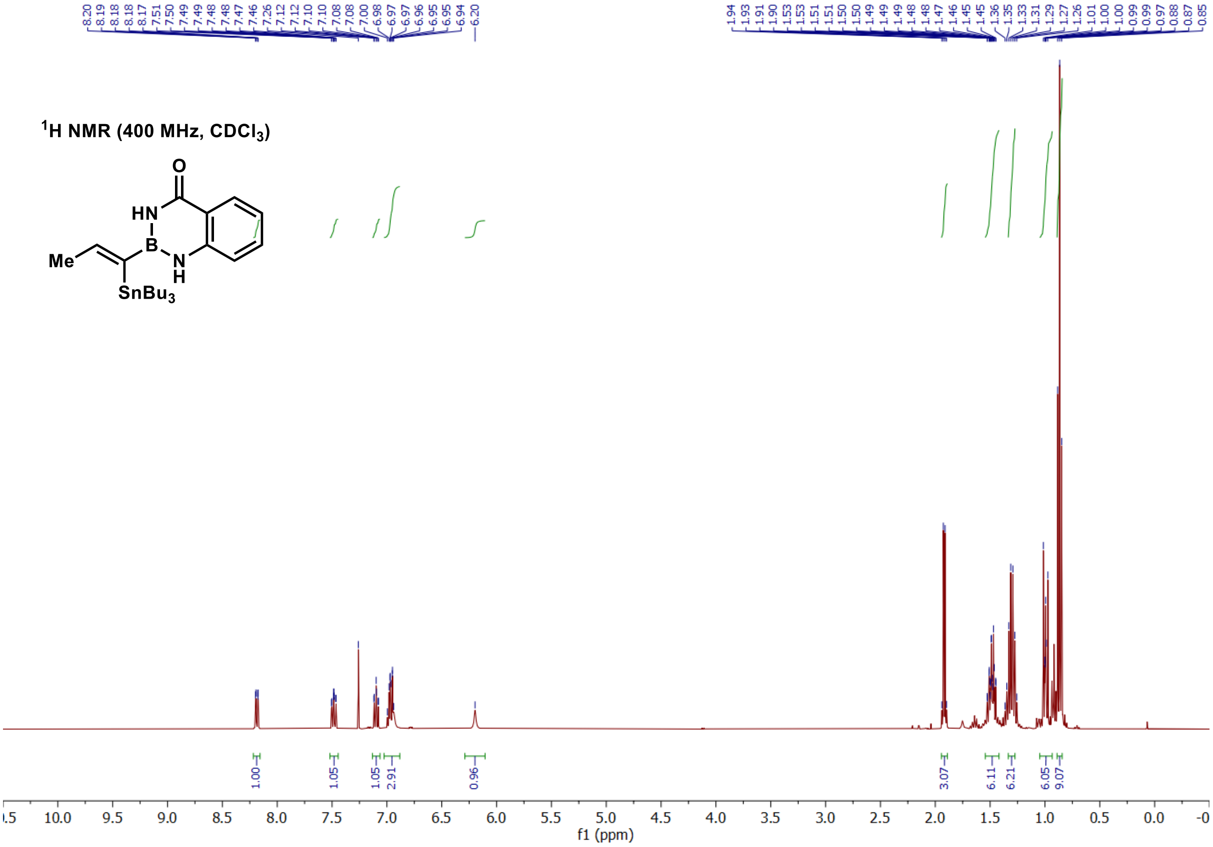

^{1}H NMR (400 MHz, CDCl$_3$)
Me
HN
B
N
H
O
SnBu$_3$
1.00
1.05
1.05
2.91
0.96
3.07
6.11
6.21
6.05
9.07
f1 (ppm)

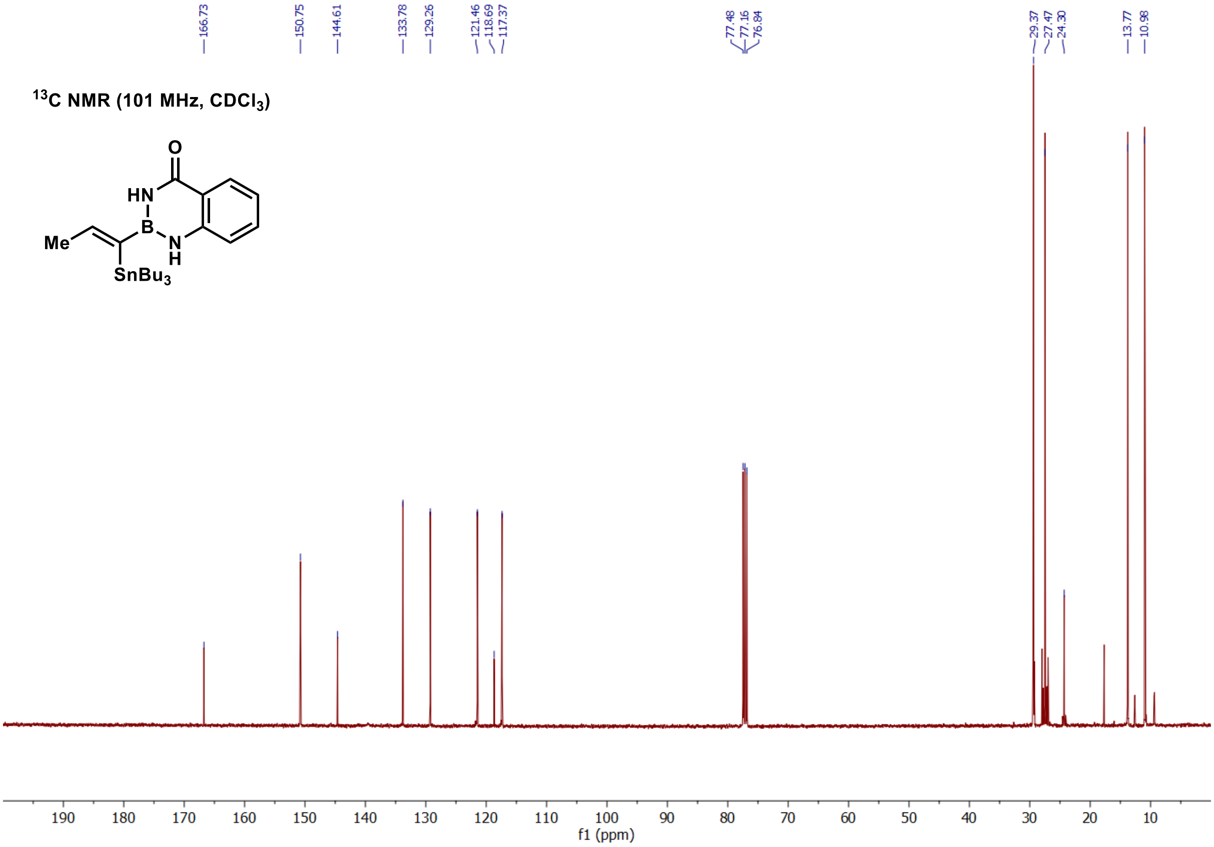

166.73
150.75
144.61
133.78
129.26
121.46
118.69
117.37
77.48
77.16
76.84
29.37
27.47
24.30
13.77
10.98
^{13}C NMR (101 MHz, CDCl$_3$)
Me
HN
B
N
H
O
SnBu$_3$
f1 (ppm)

^{11}B NMR (128 MHz, $CDCl_3$)

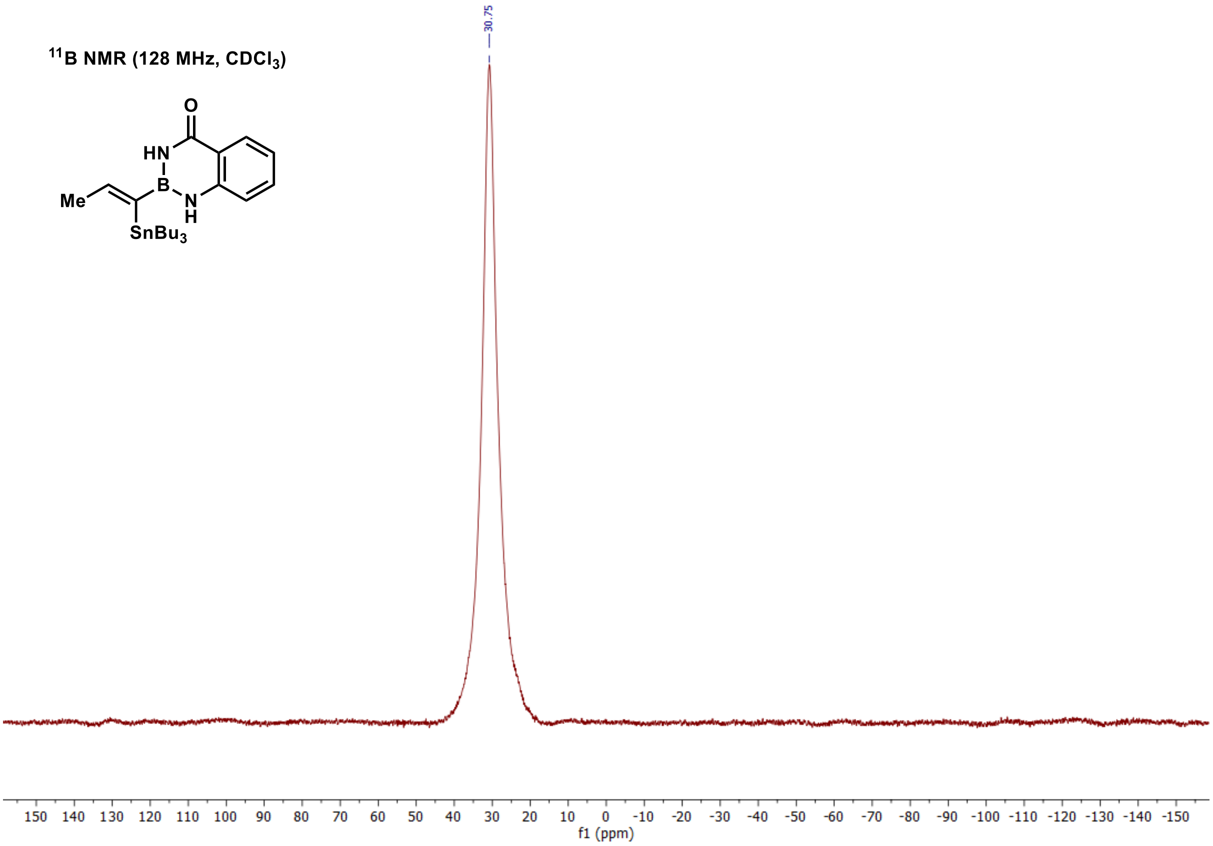

^{119}Sn NMR (149 MHz, $CDCl_3$)

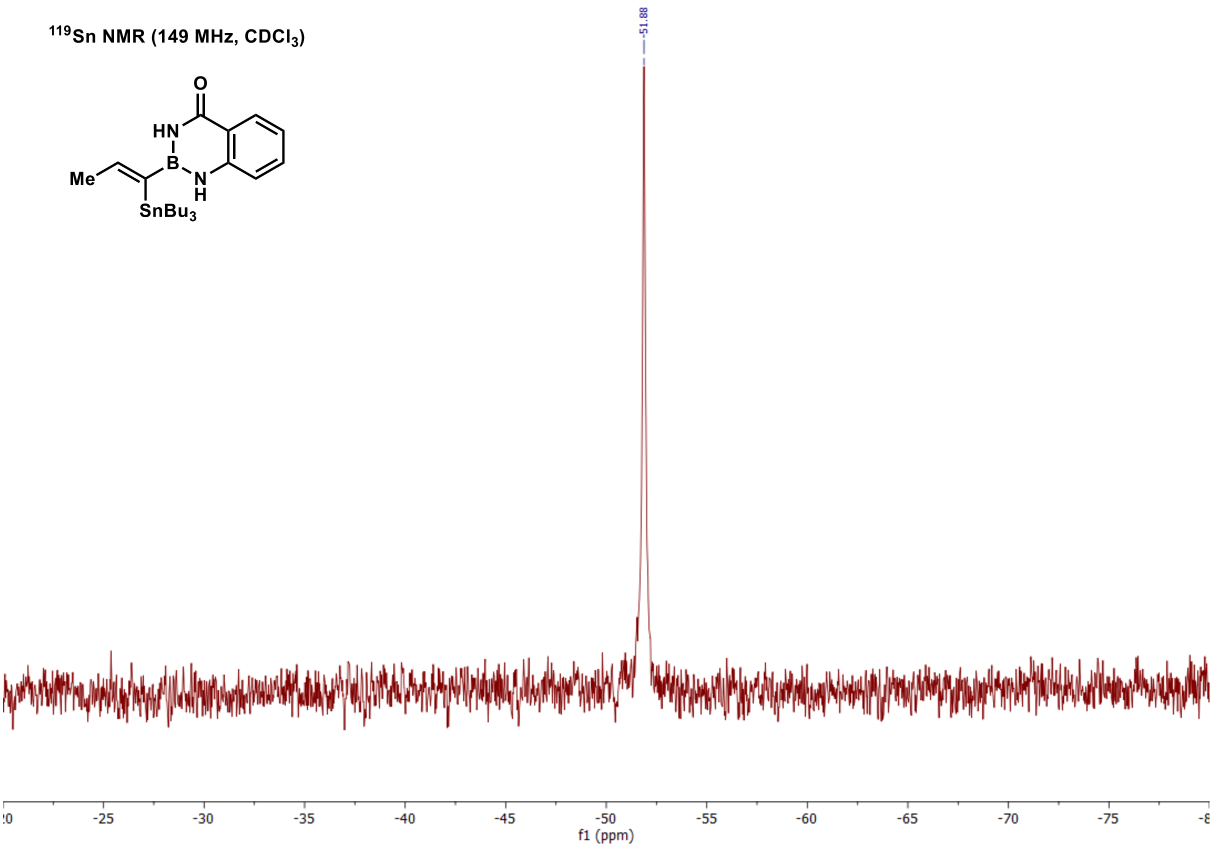

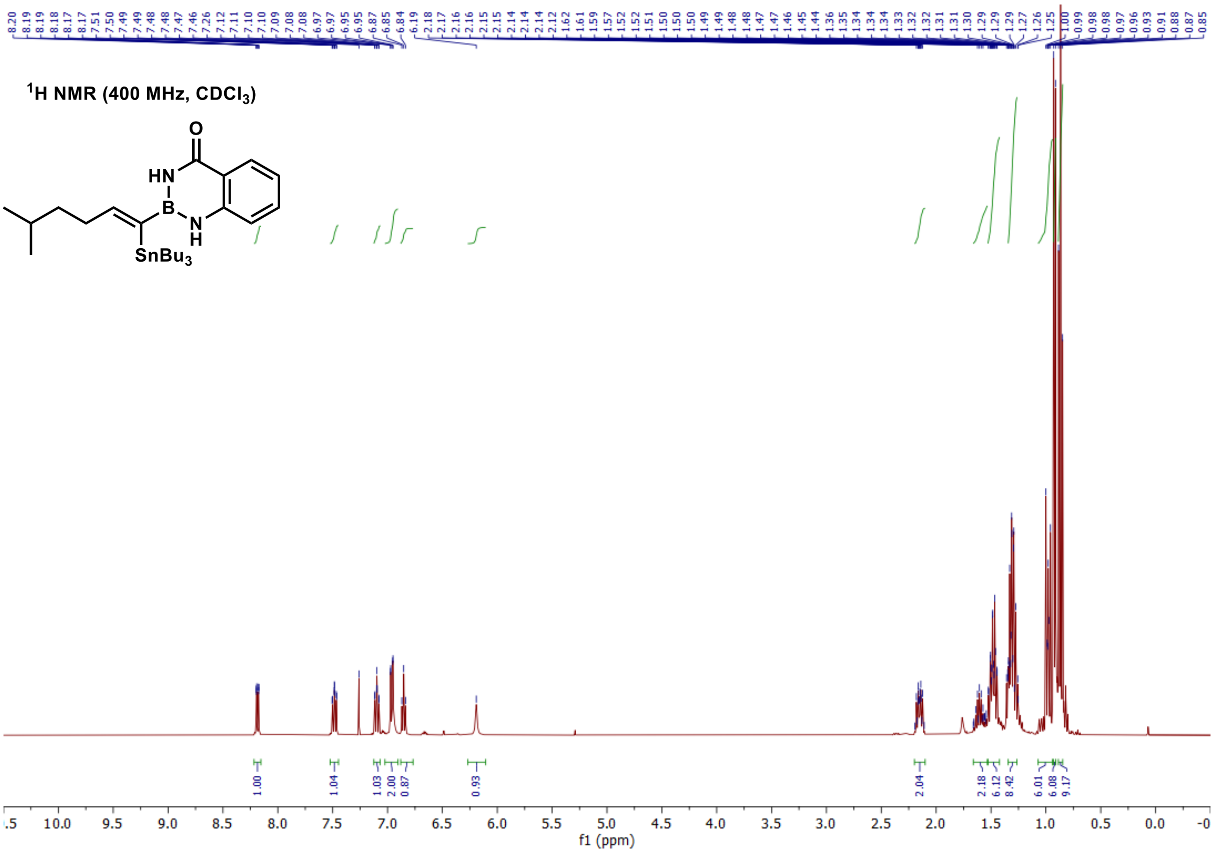
1H NMR (400 MHz, CDCl3)
HN
B
N
H
O
SnBu3
f1 (ppm)

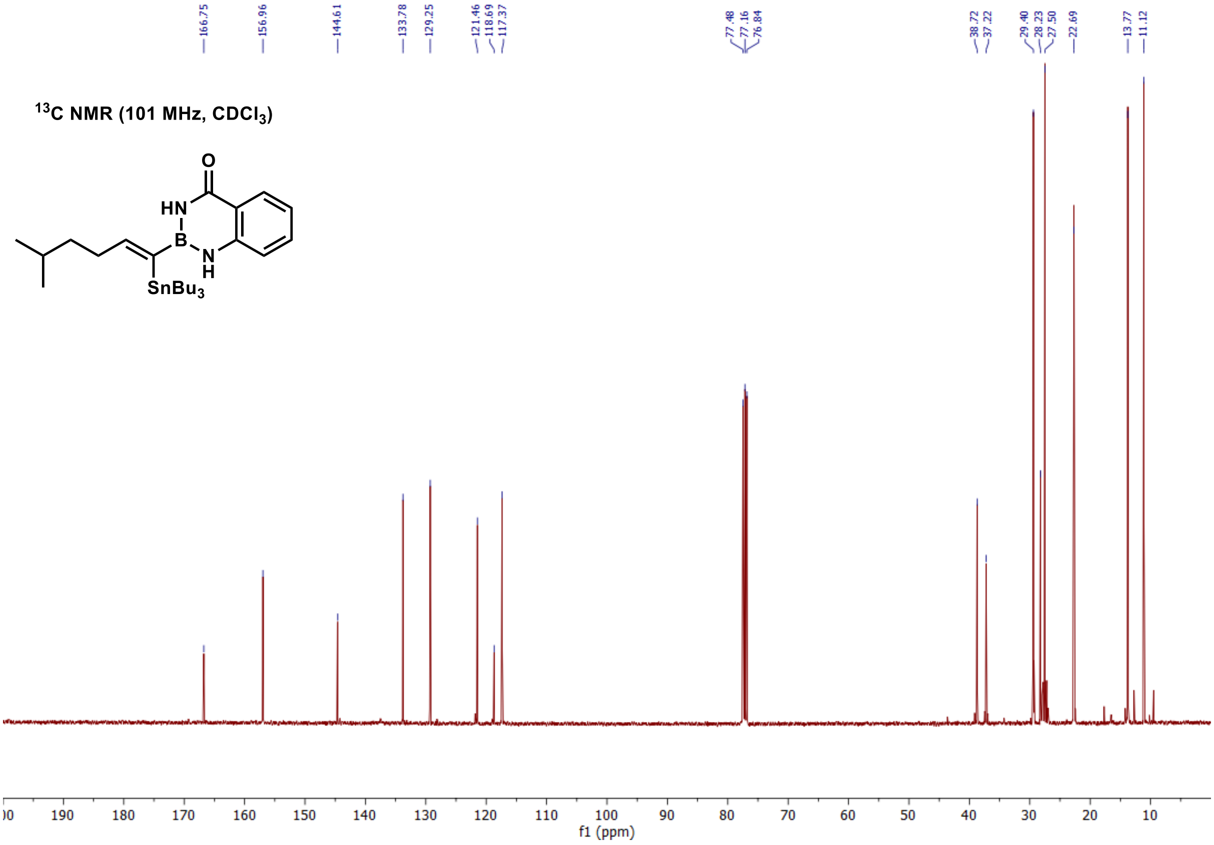
13C NMR (101 MHz, CDCl3)
HN
B
N
H
O
SnBu3
166.75
156.96
144.61
133.78
129.25
121.46
118.69
117.37
77.48
77.16
76.84
38.72
37.22
29.40
28.23
27.50
22.69
13.77
11.12
f1 (ppm)

^{11}B NMR (128 MHz, $CDCl_3$)

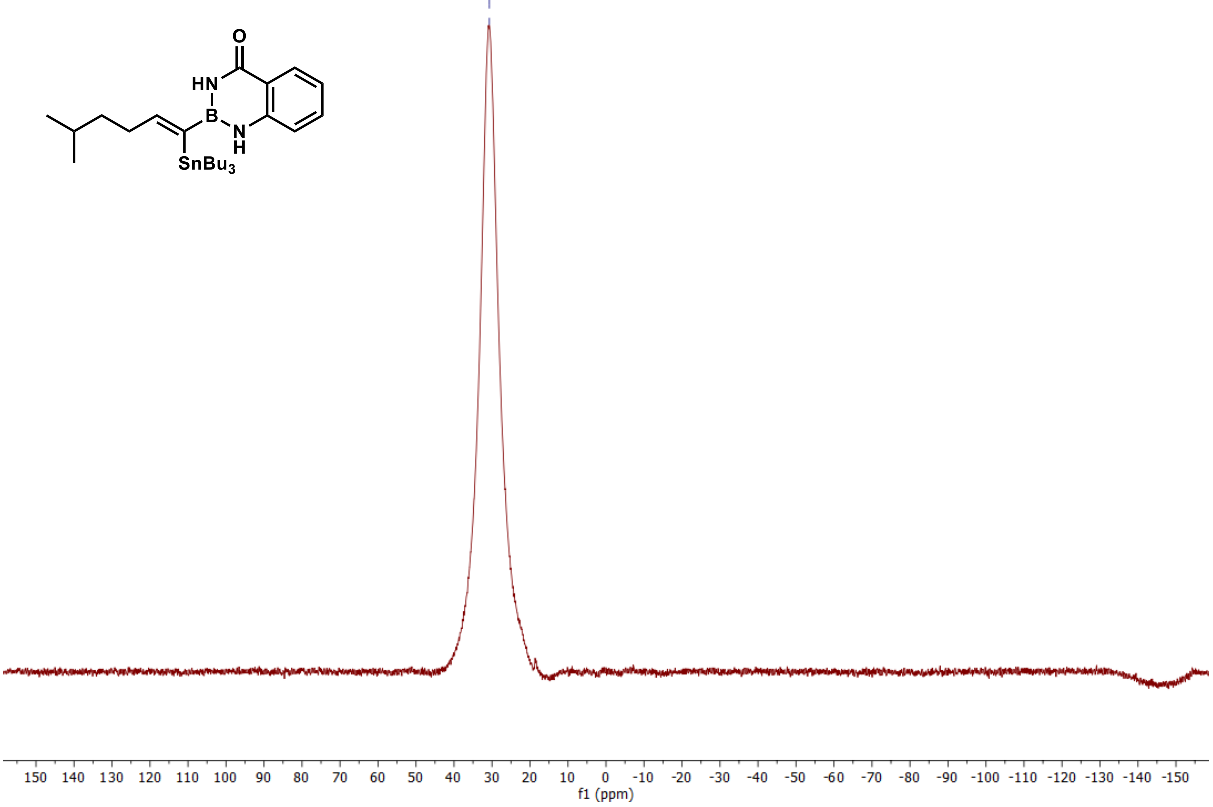

^{119}Sn NMR (149 MHz, $CDCl_3$)

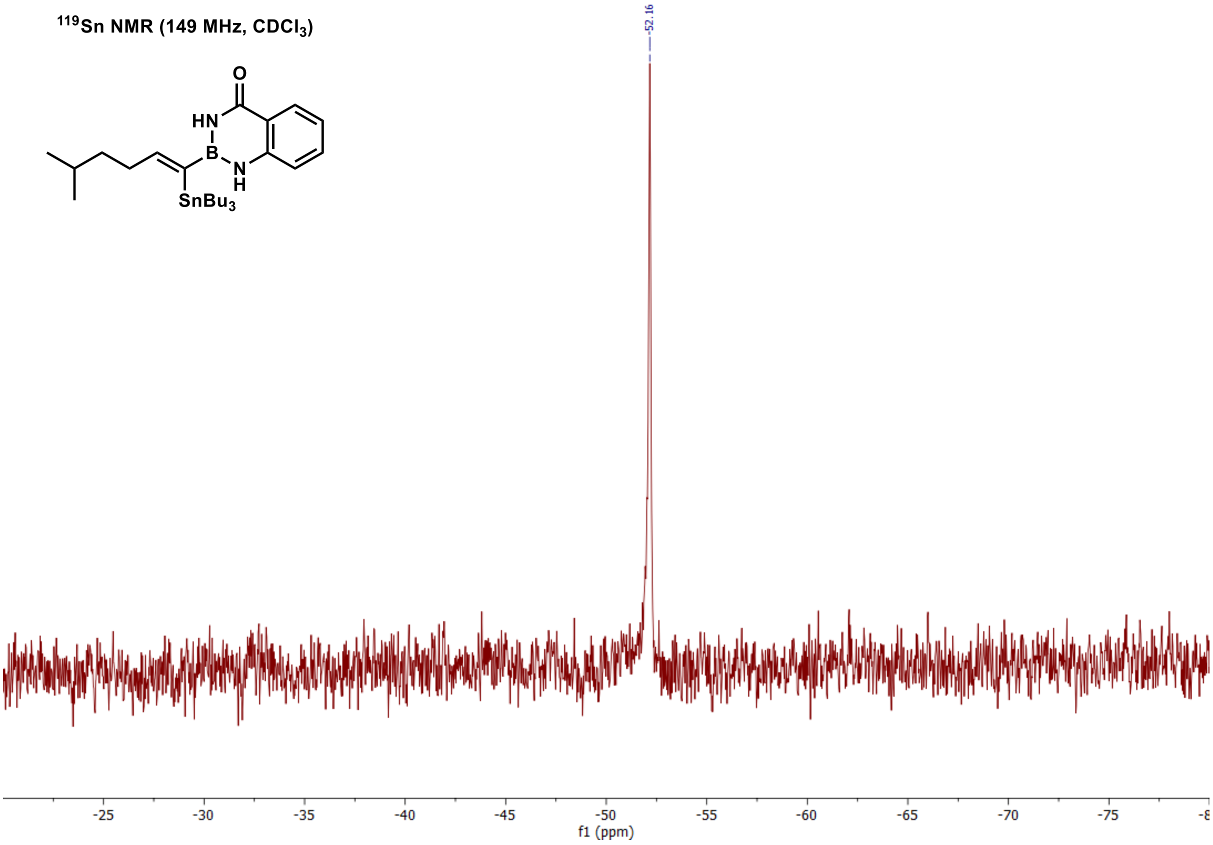

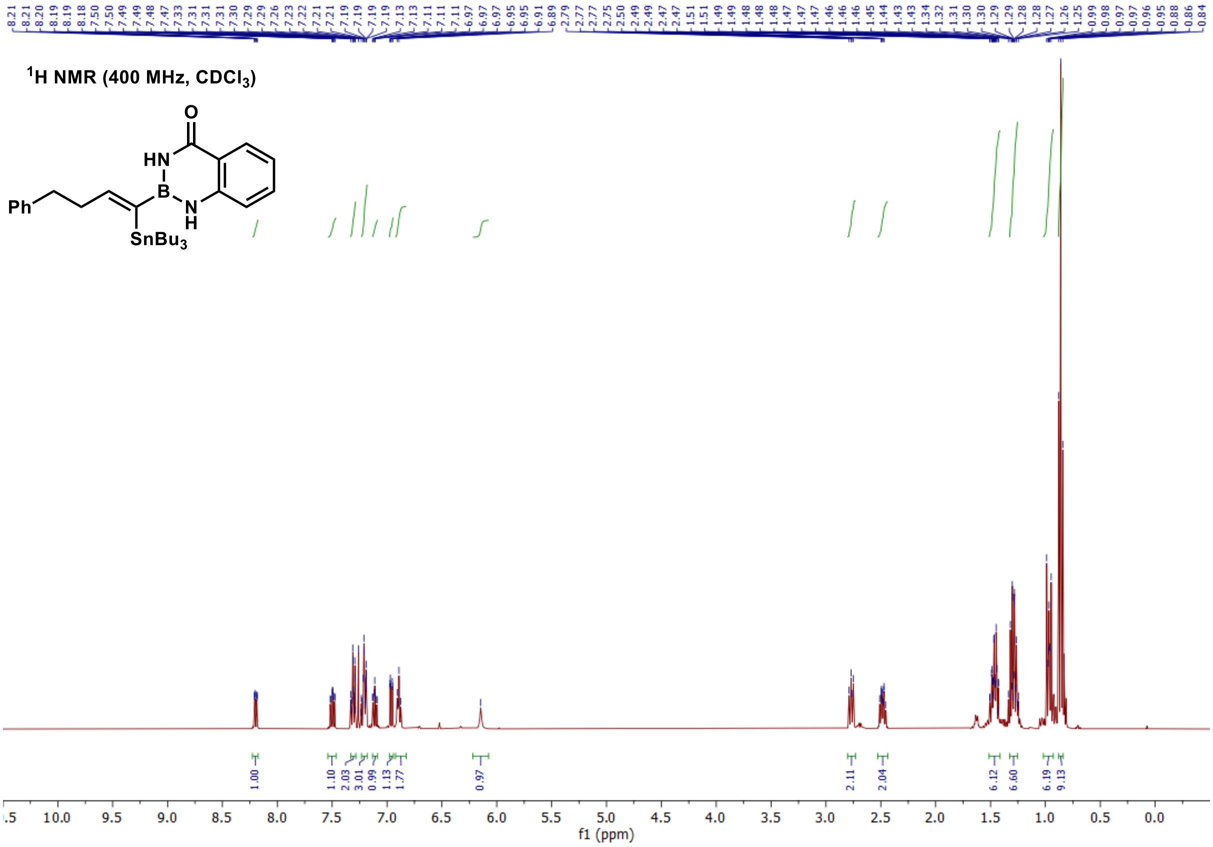
1H NMR (400 MHz, CDCl3)
Ph
SnBu3
f1 (ppm)

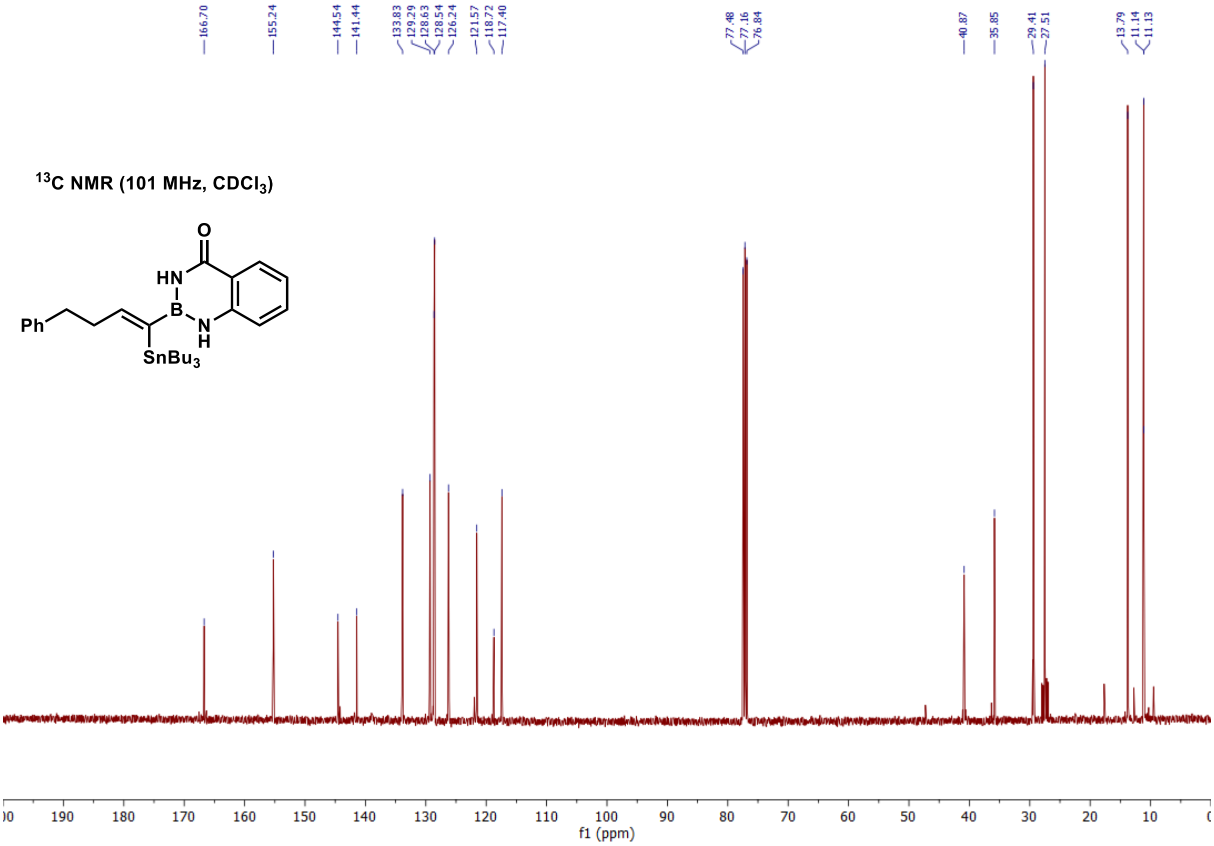
13C NMR (101 MHz, CDCl3)
Ph
SnBu3
f1 (ppm)

^{11}B NMR (128 MHz, $CDCl_3$)

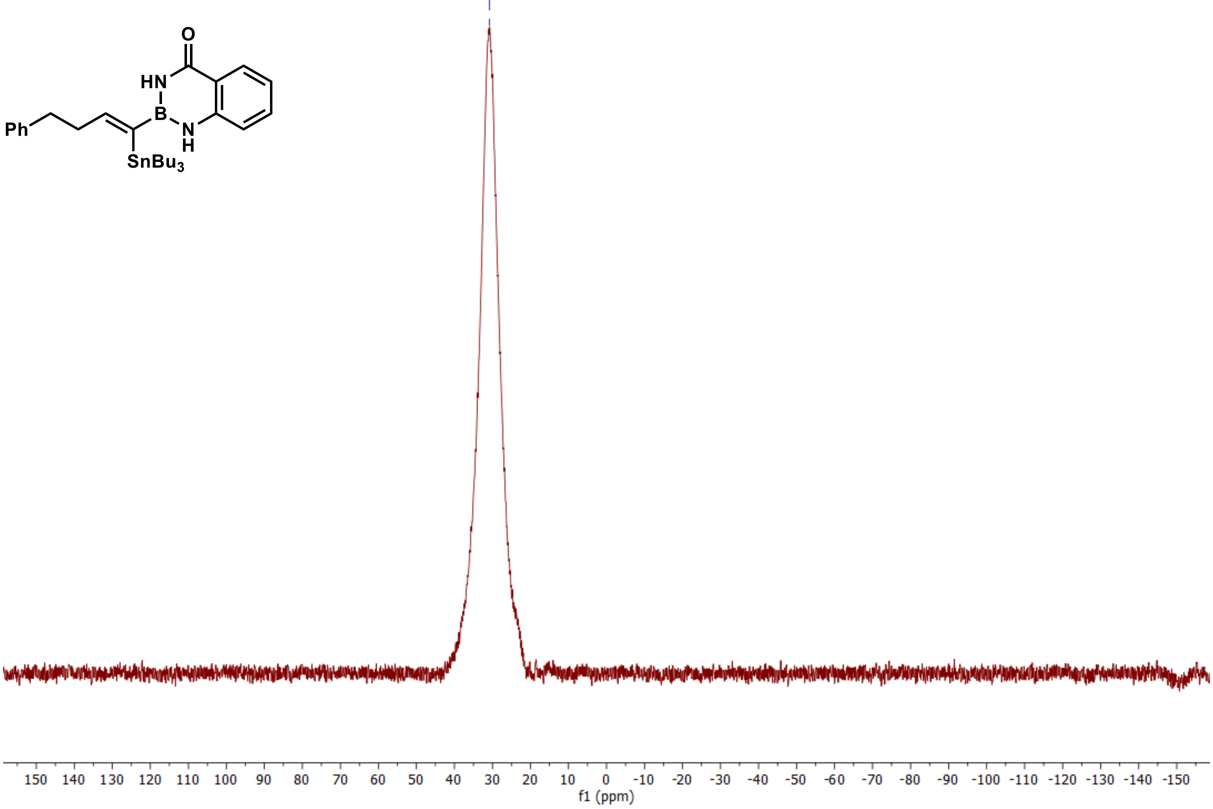

^{119}Sn NMR (149 MHz, $CDCl_3$)

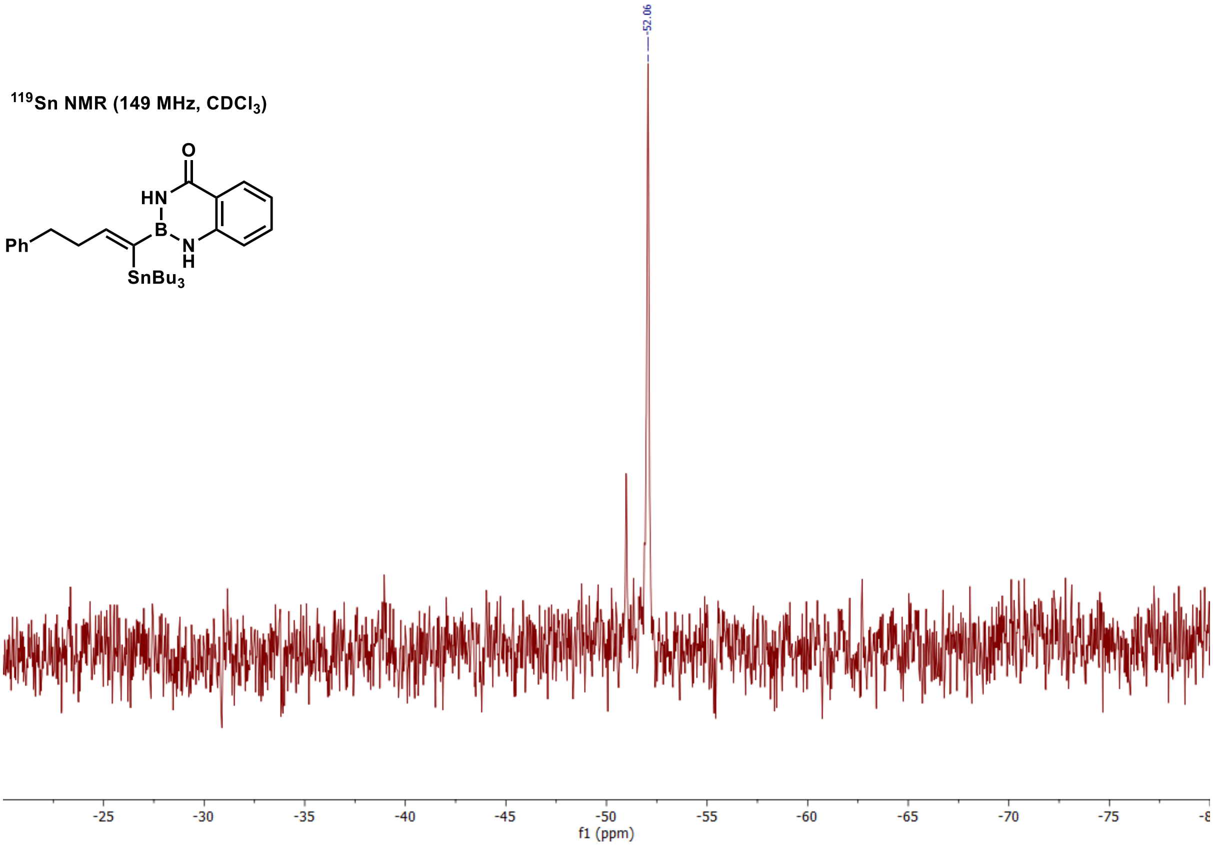

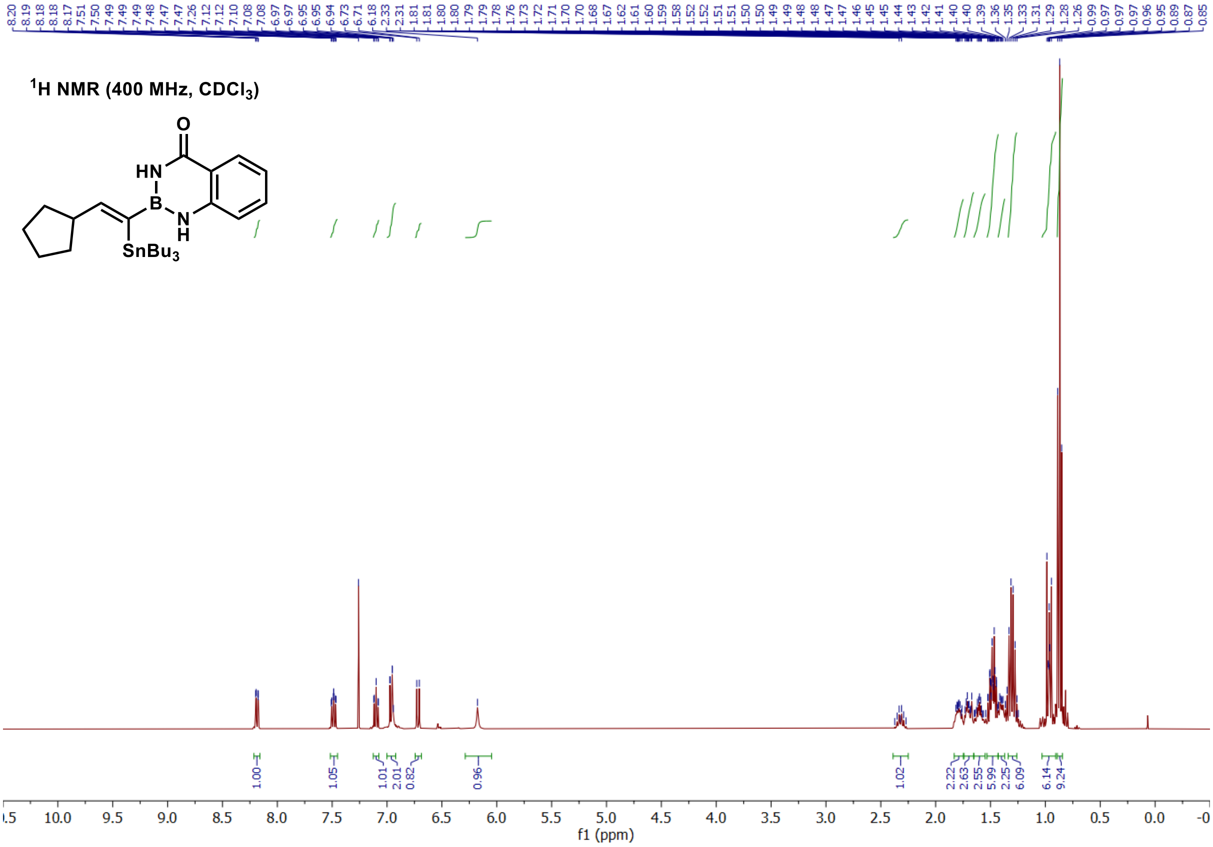
1H NMR (400 MHz, CDCl3)
O
HN
B
N
H
SnBu3
f1 (ppm)

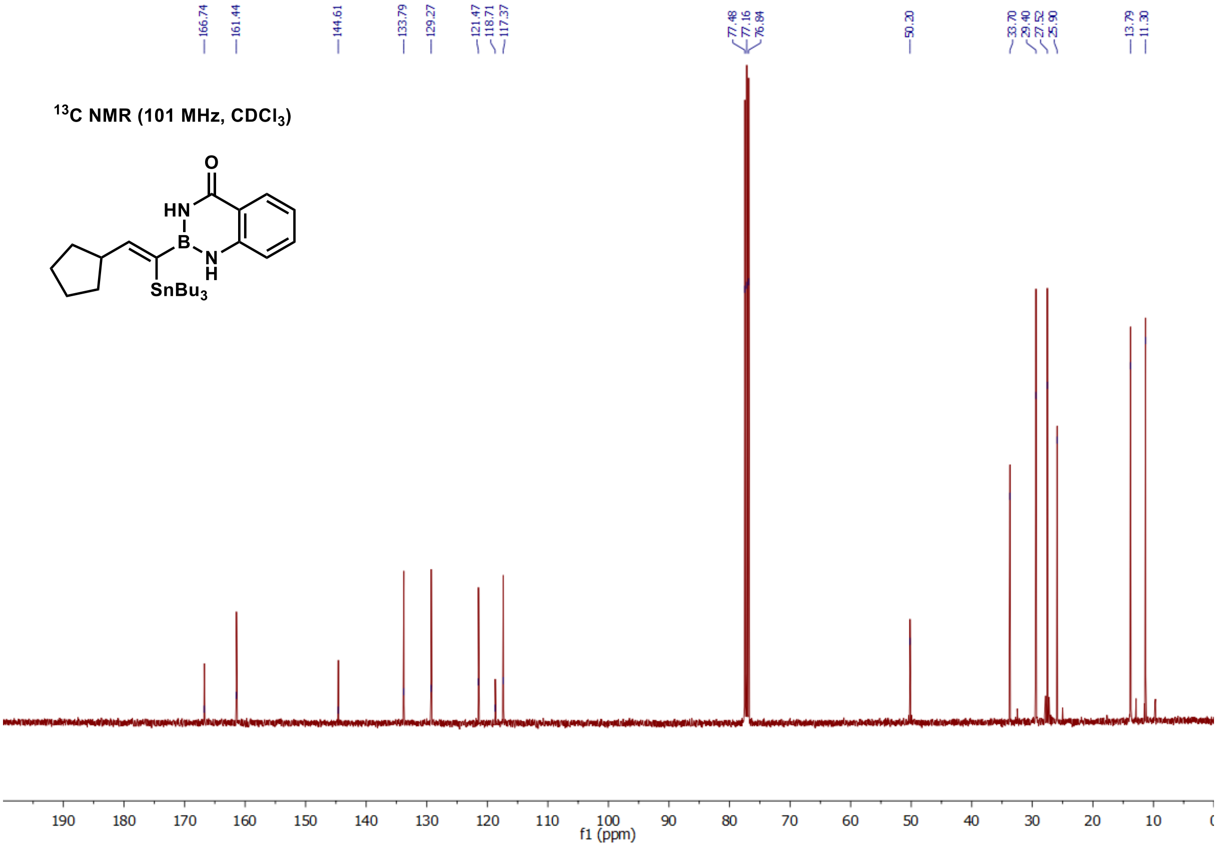
166.74
161.44
144.61
133.79
129.27
121.47
118.71
117.37
77.48
77.16
76.84
50.20
33.70
29.40
27.52
25.90
13.79
11.30
13C NMR (101 MHz, CDCl3)
O
HN
B
N
H
SnBu3
f1 (ppm)

^{11}B NMR (128 MHz, $CDCl_3$)

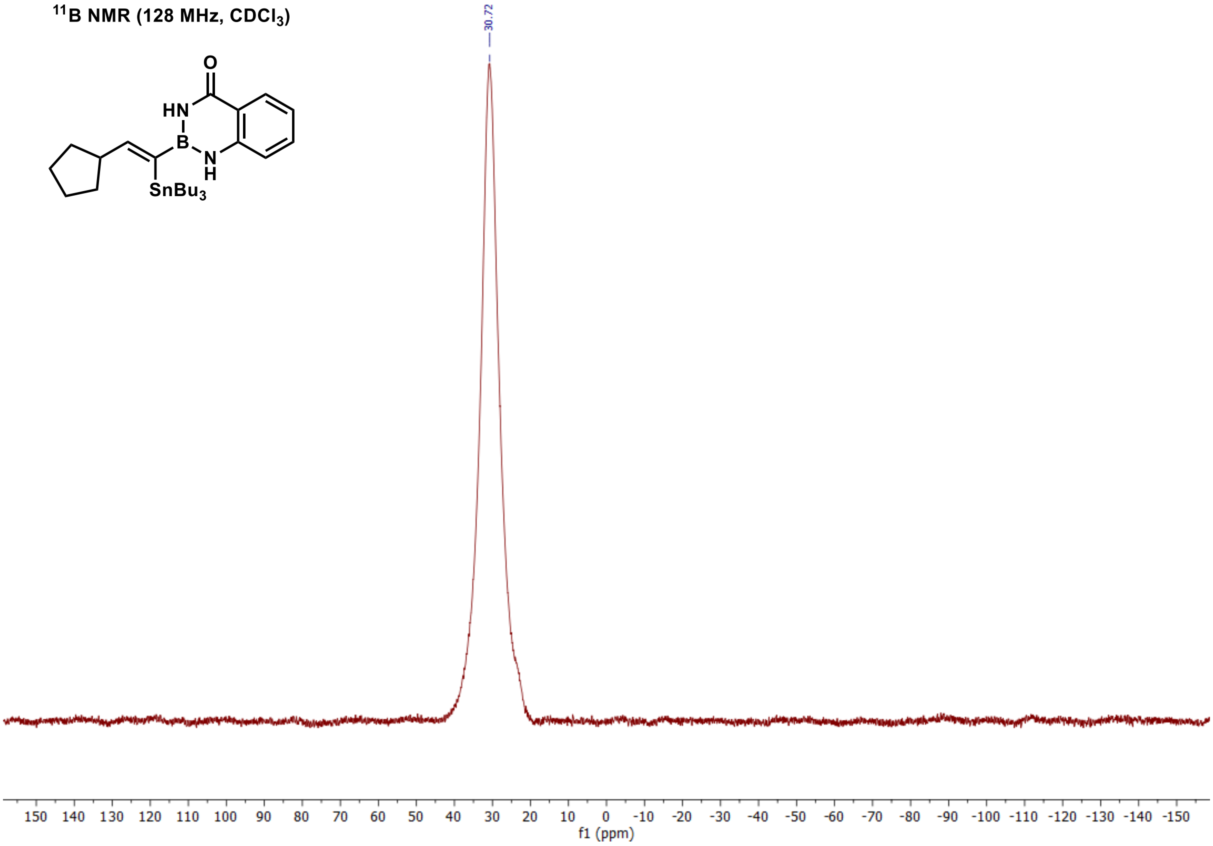

^{119}Sn NMR (149 MHz, $CDCl_3$)

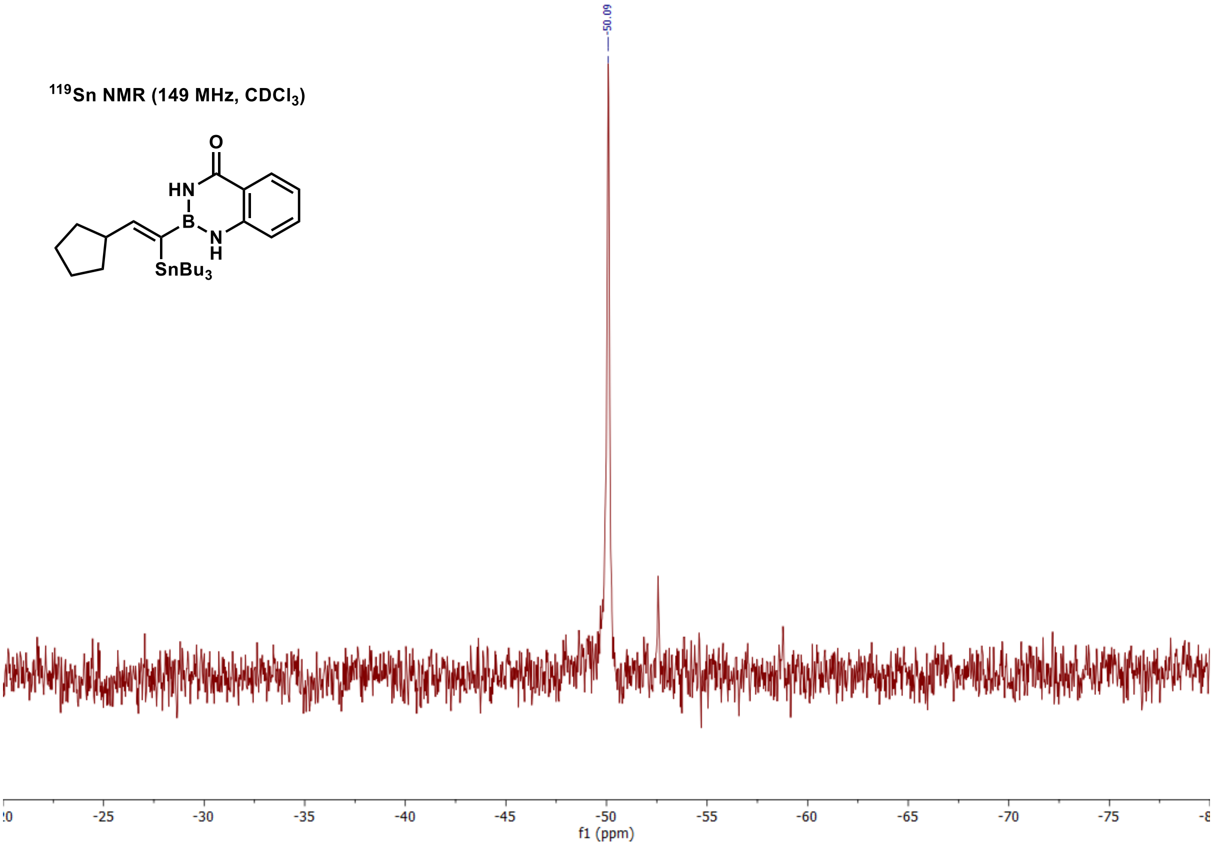

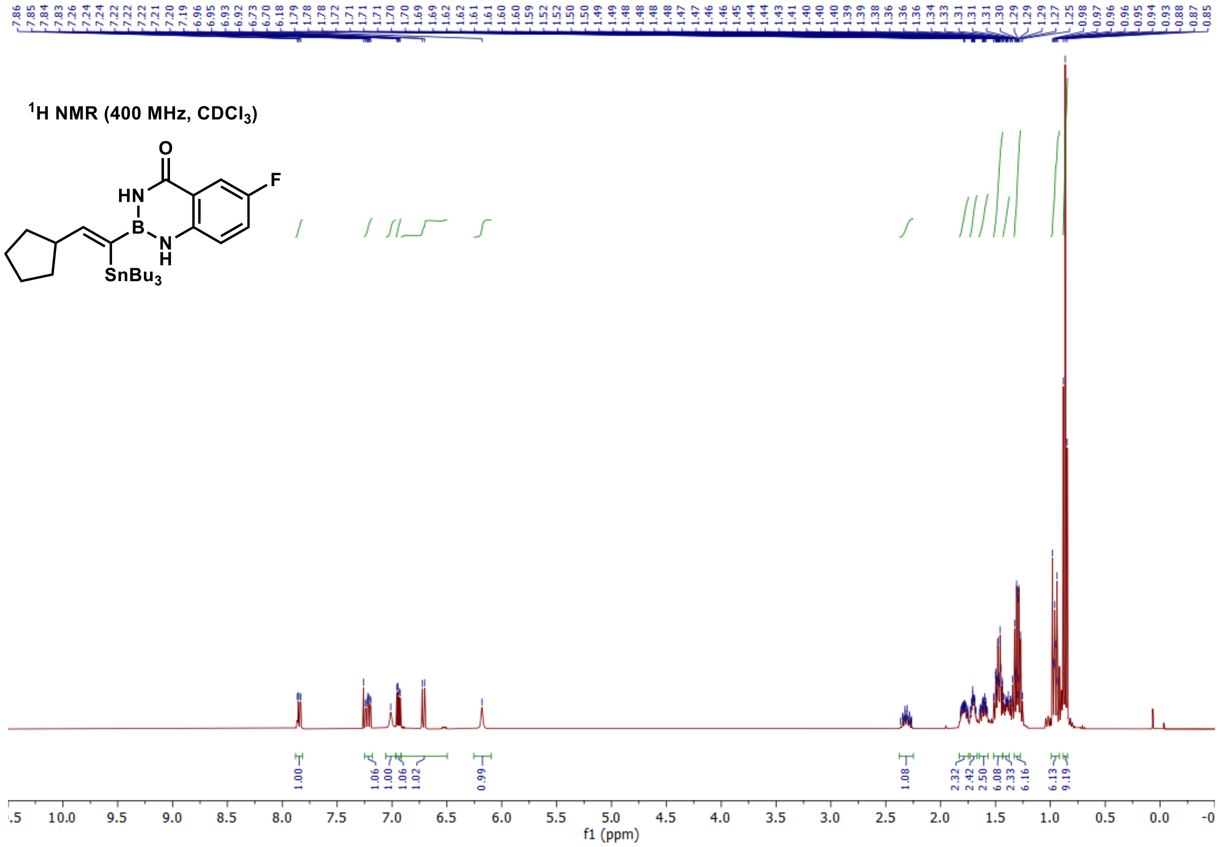
1H NMR (400 MHz, CDCl3)
f1 (ppm)

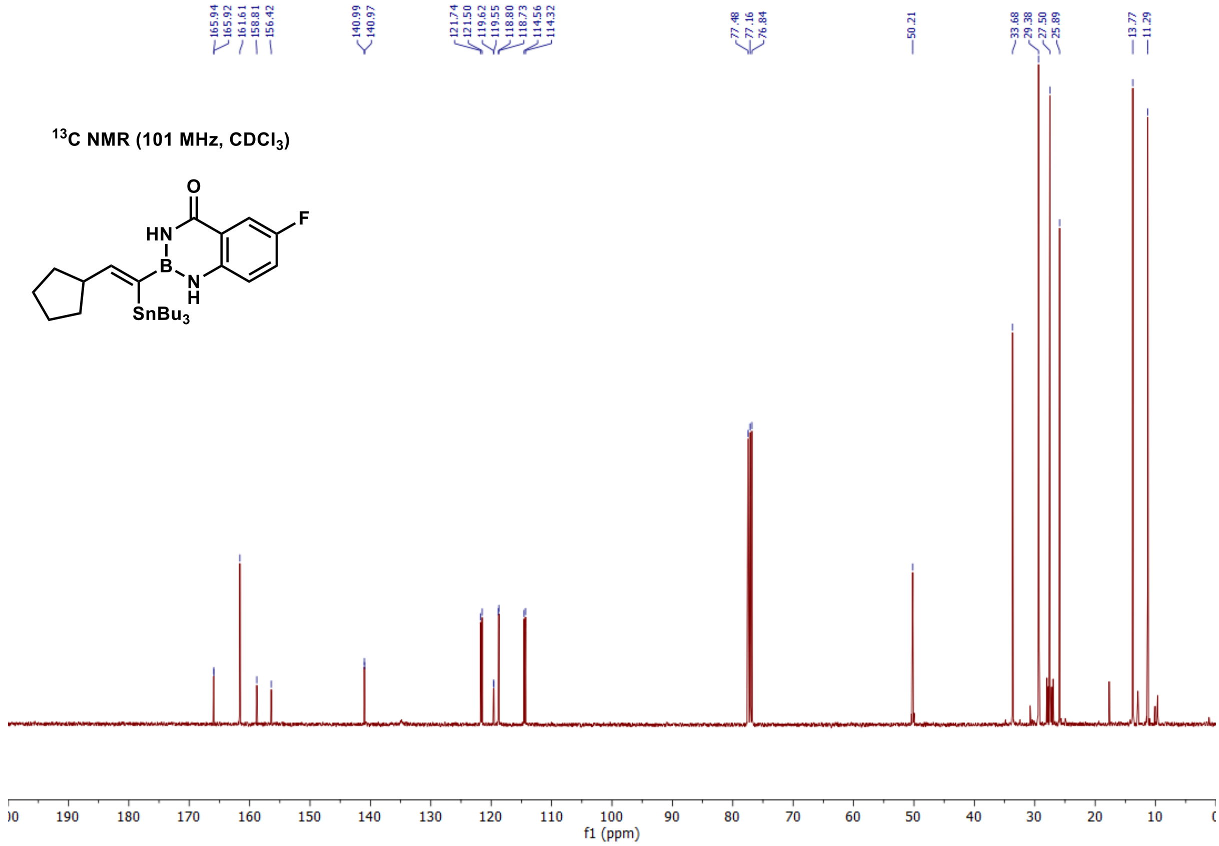
13C NMR (101 MHz, CDCl3)
f1 (ppm)

^{11}B NMR (128 MHz, $CDCl_3$)

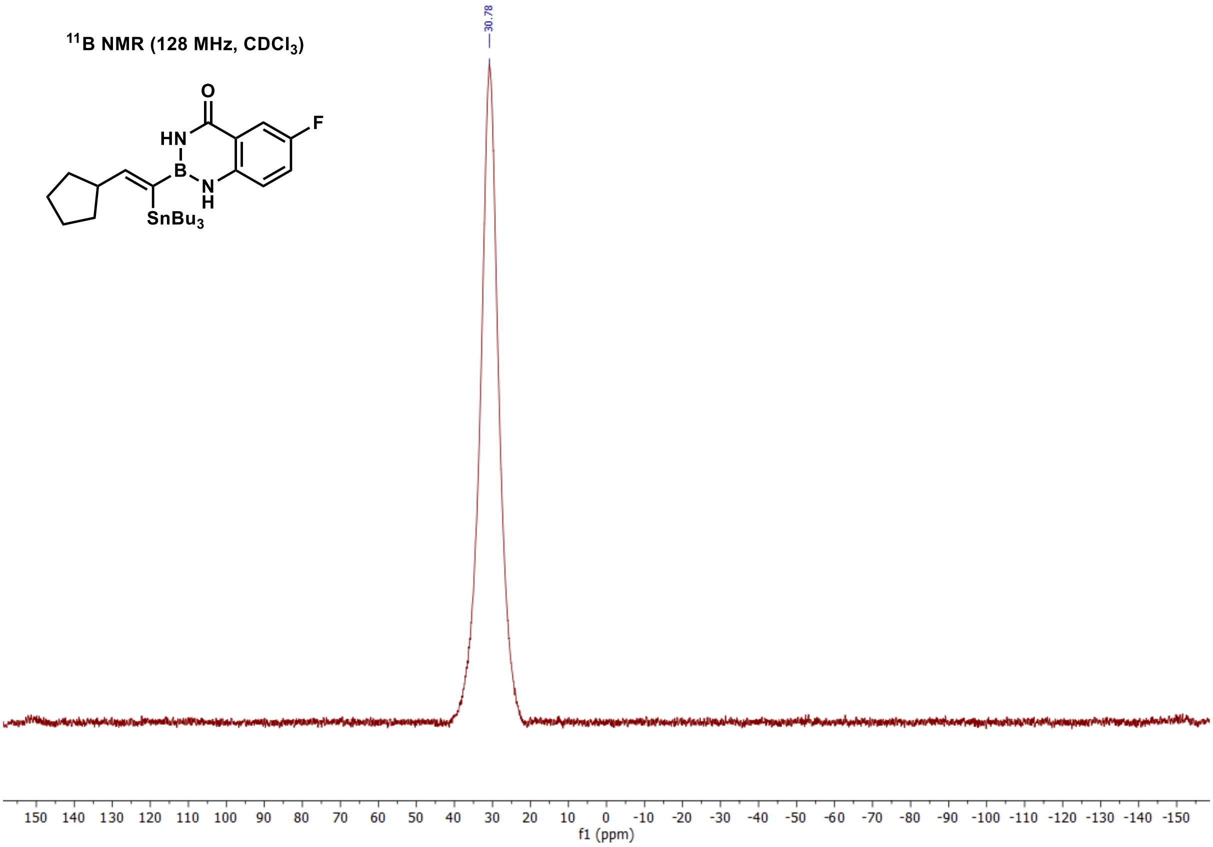

^{19}F NMR (282 MHz, $CDCl_3$)

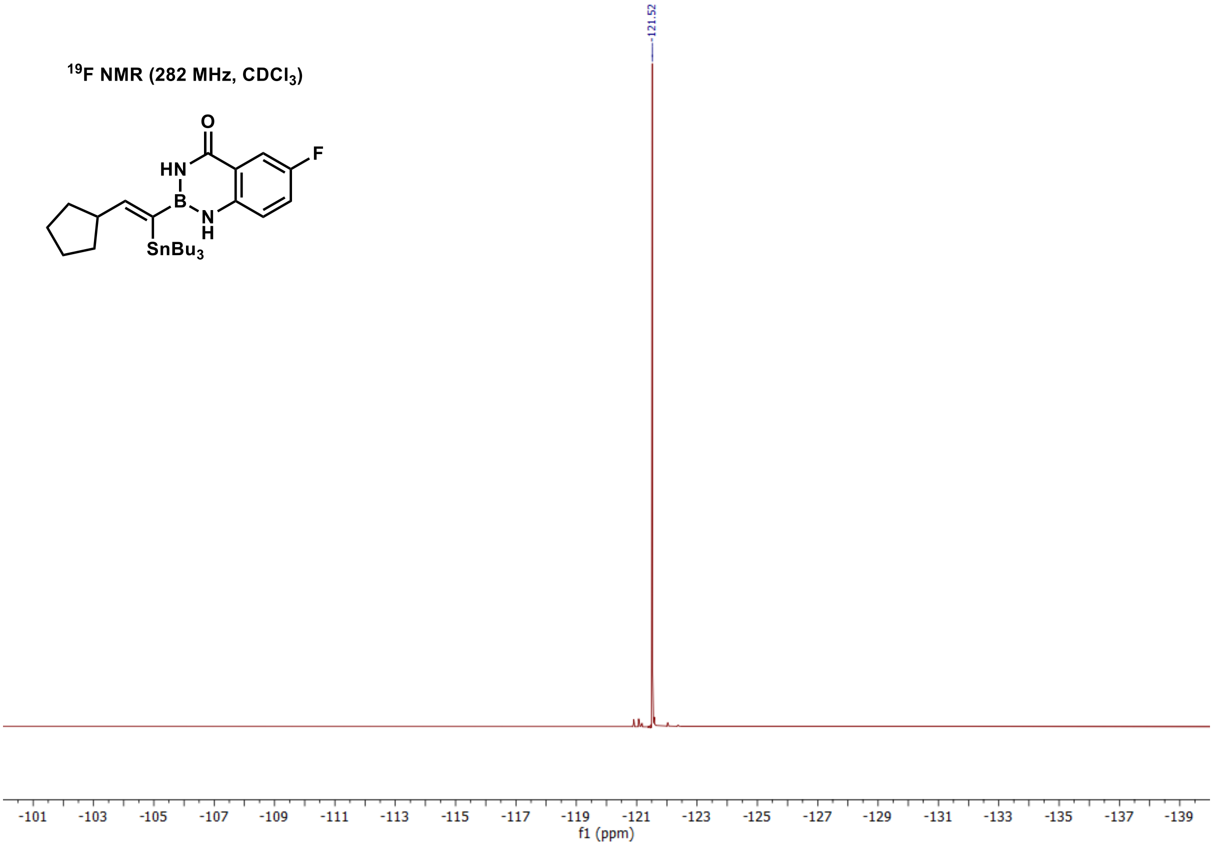

^{119}Sn NMR (149 MHz, CDCl$_3$)

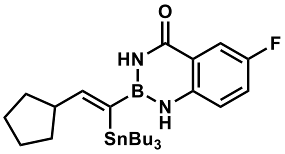

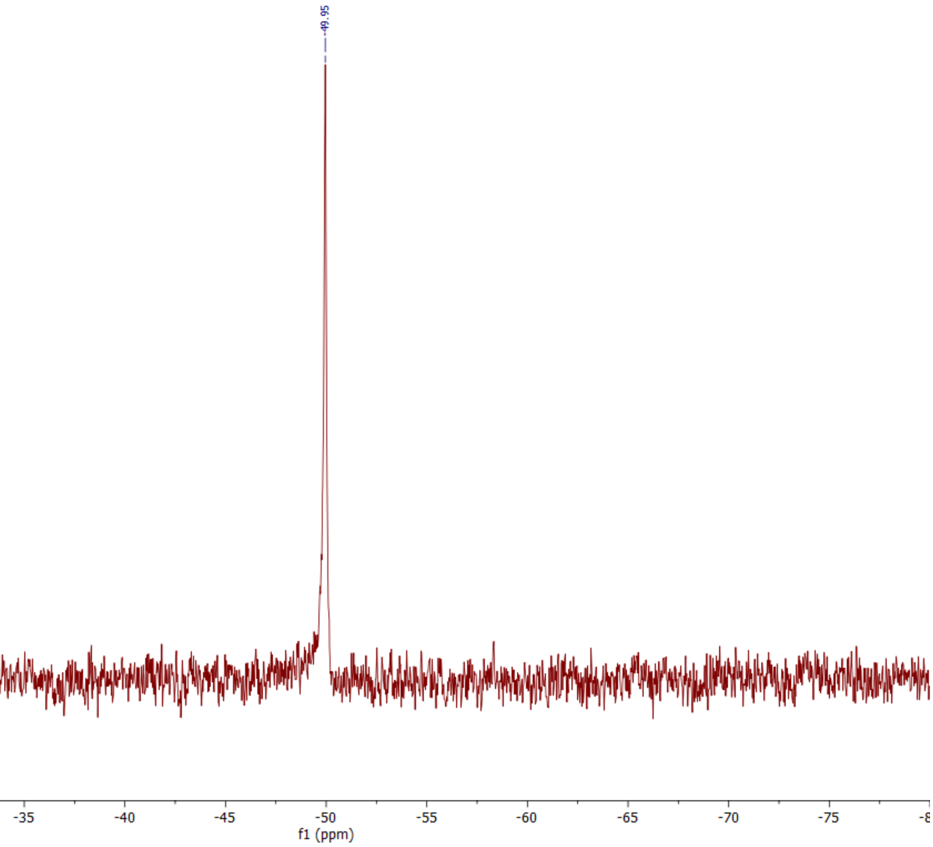

^{1}H NMR (400 MHz, $CDCl_3$)

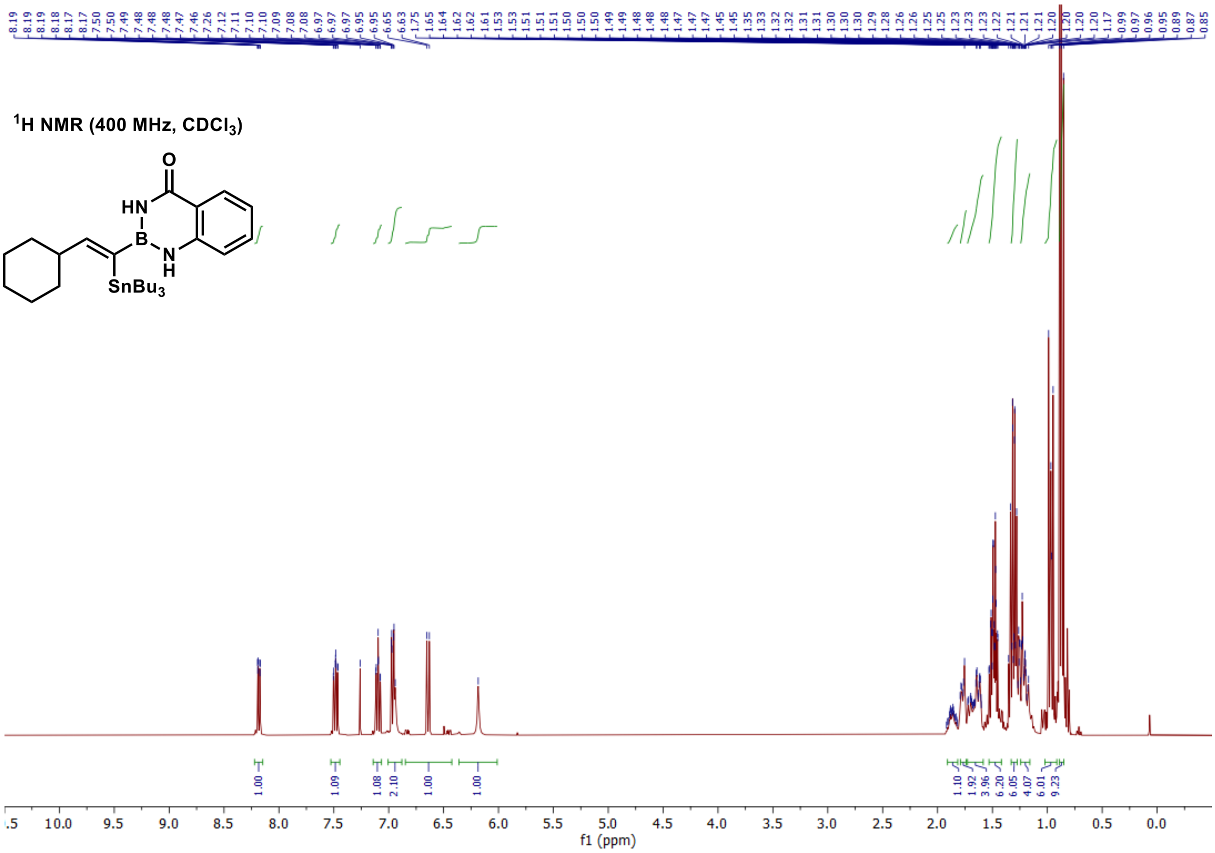

^{13}C NMR (101 MHz, $CDCl_3$)

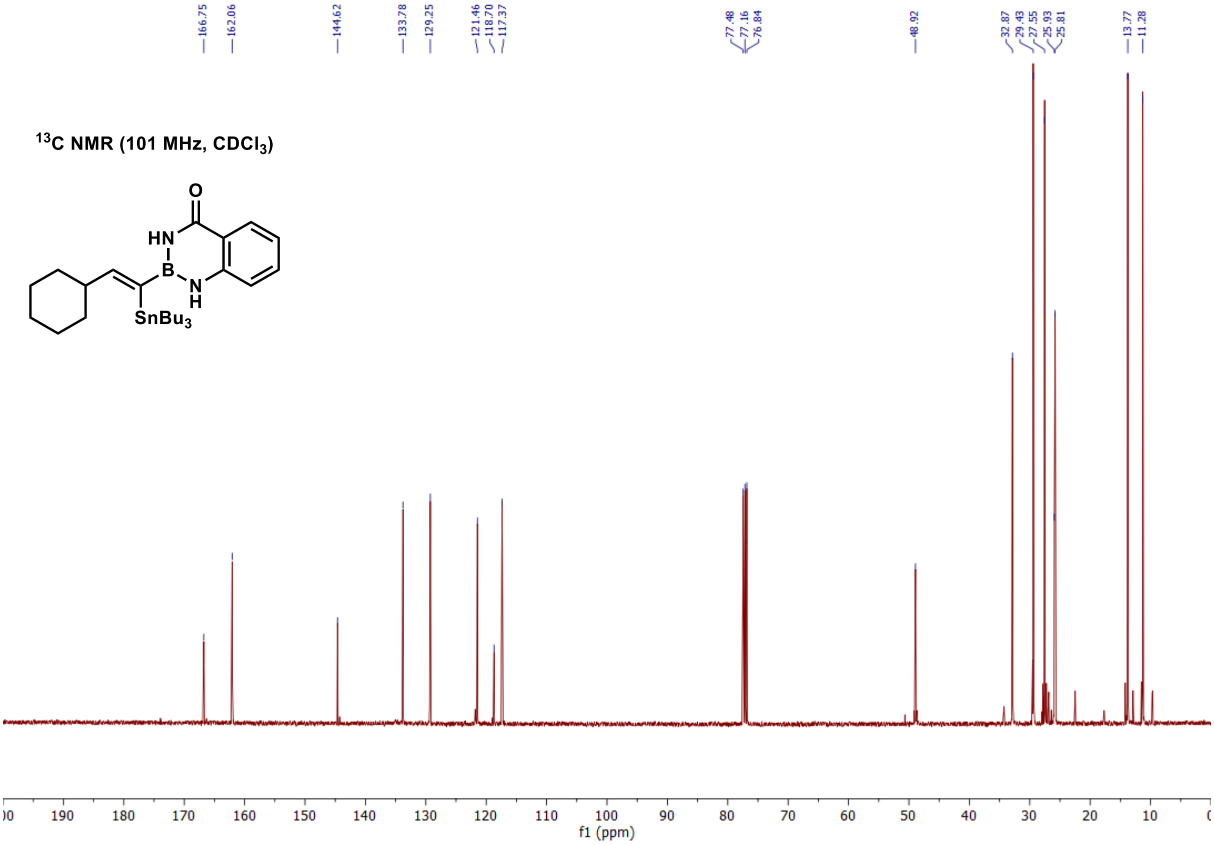

^{11}B NMR (128 MHz, $CDCl_3$)

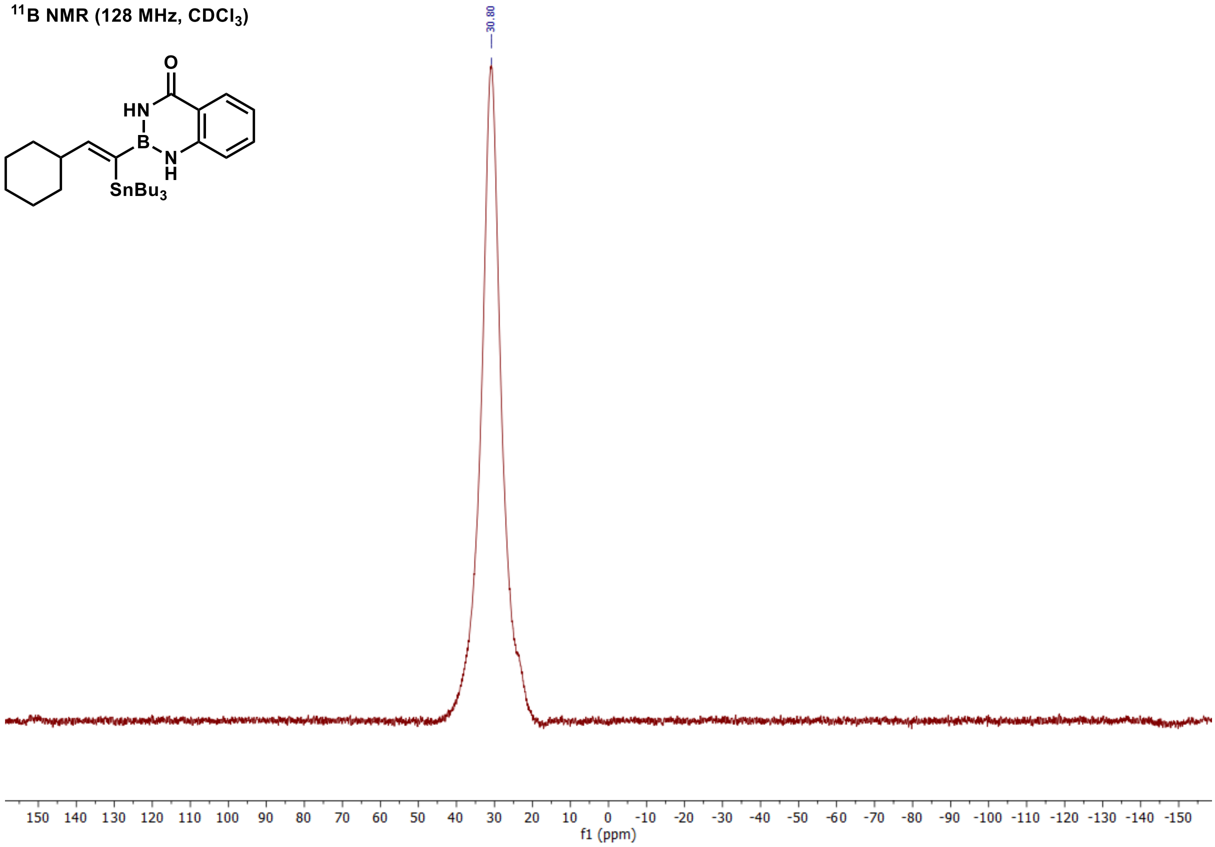

^{119}Sn NMR (149 MHz, $CDCl_3$)

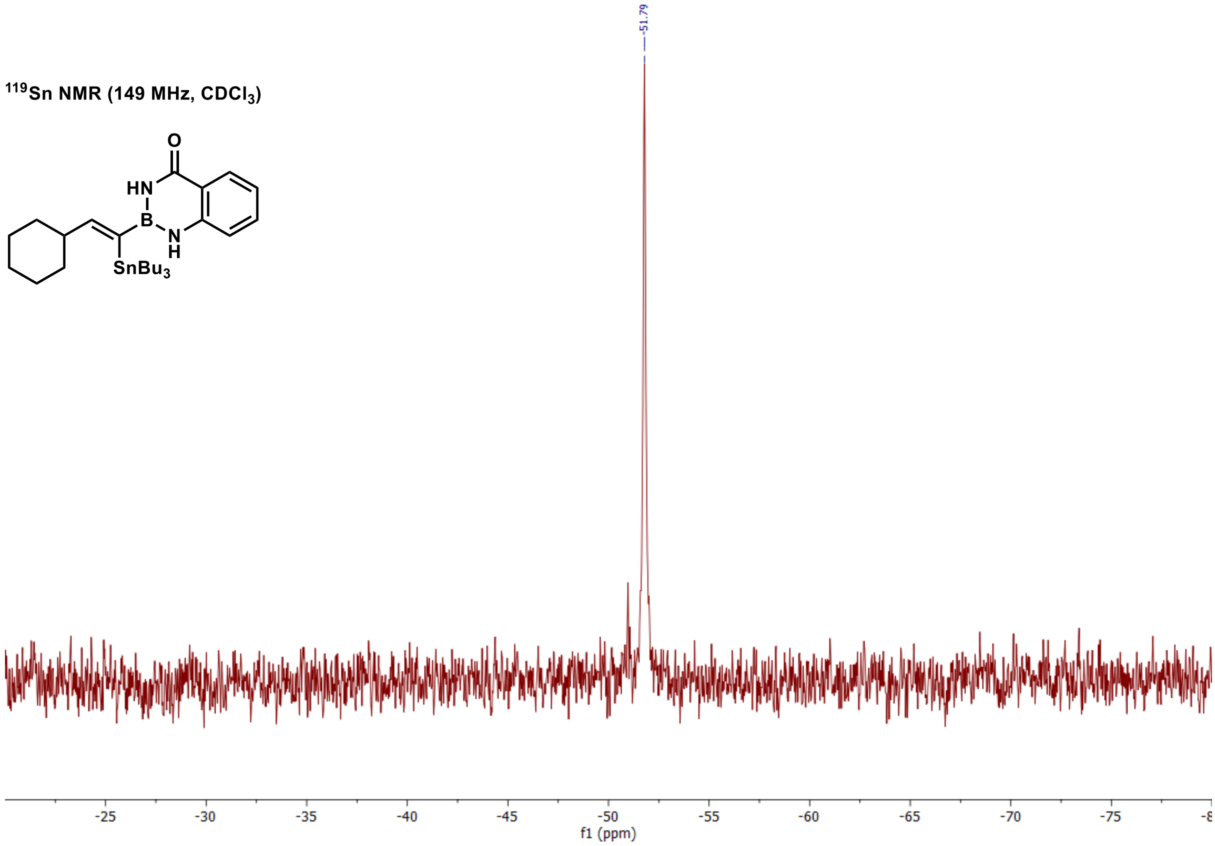

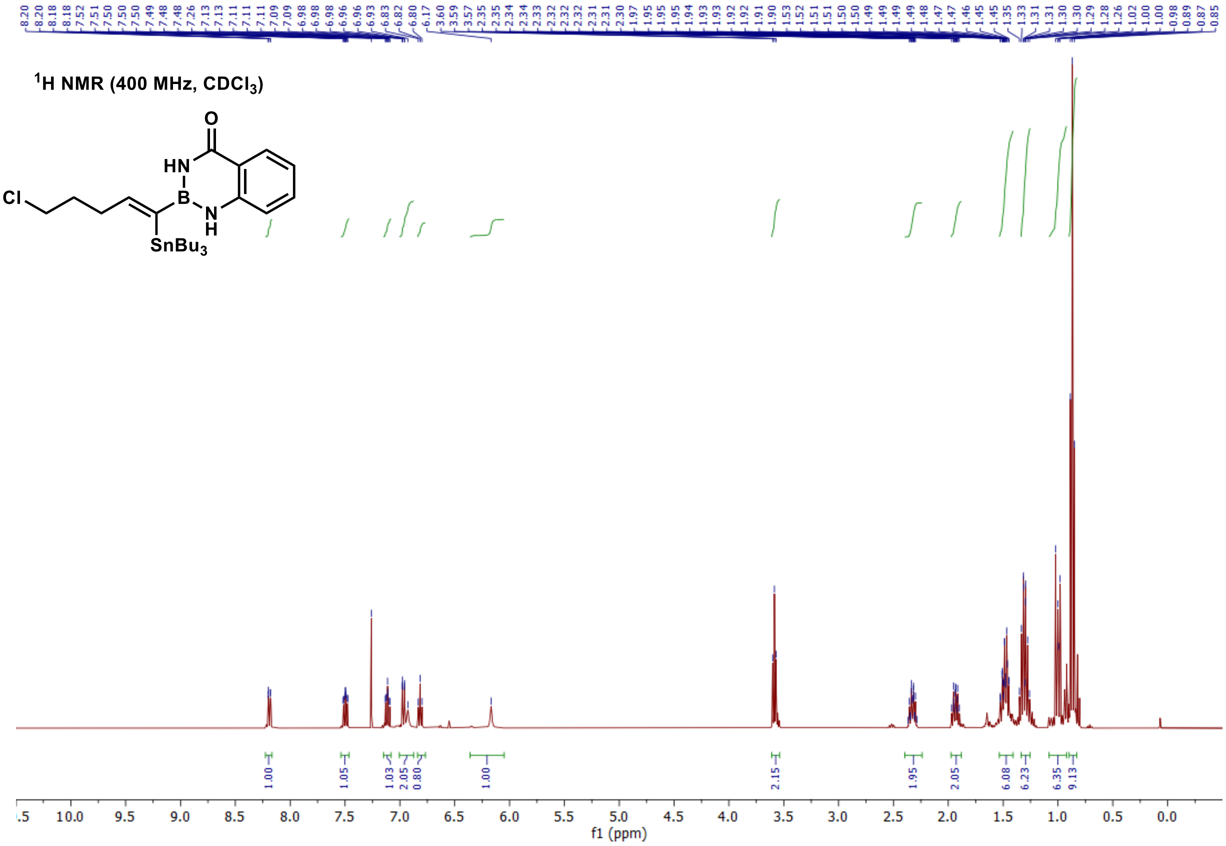
1H NMR (400 MHz, CDCl3)
O
HN
Cl
B
N
H
SnBu3
1.00
1.05
1.03
2.05
0.80
1.00
2.15
1.95
2.05
6.08
6.23
6.35
9.13
f1 (ppm)

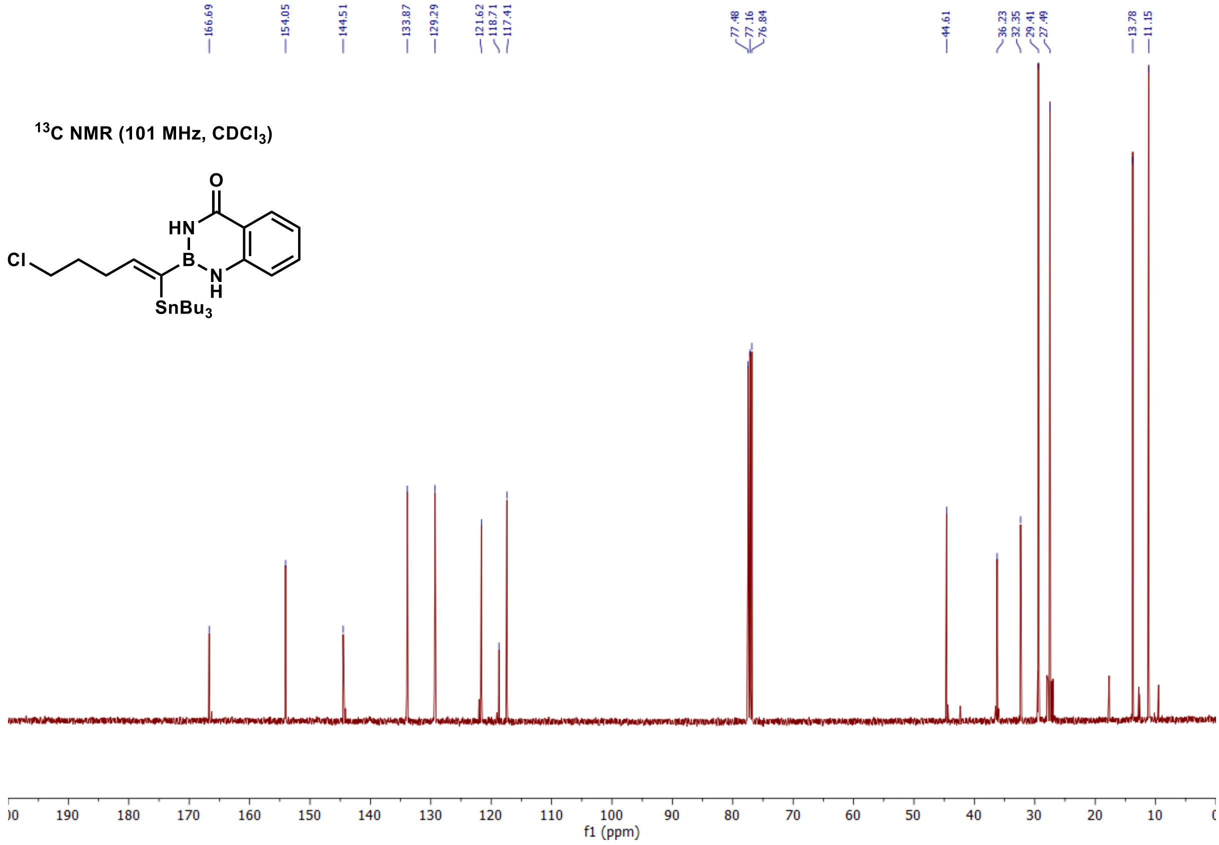
166.69
154.05
144.51
133.87
129.29
121.62
118.71
117.41
77.48
77.16
76.84
44.61
36.23
32.35
29.41
27.49
13.78
11.15
13C NMR (101 MHz, CDCl3)
O
HN
Cl
B
N
H
SnBu3
f1 (ppm)

^{11}B NMR (128 MHz, $CDCl_3$)

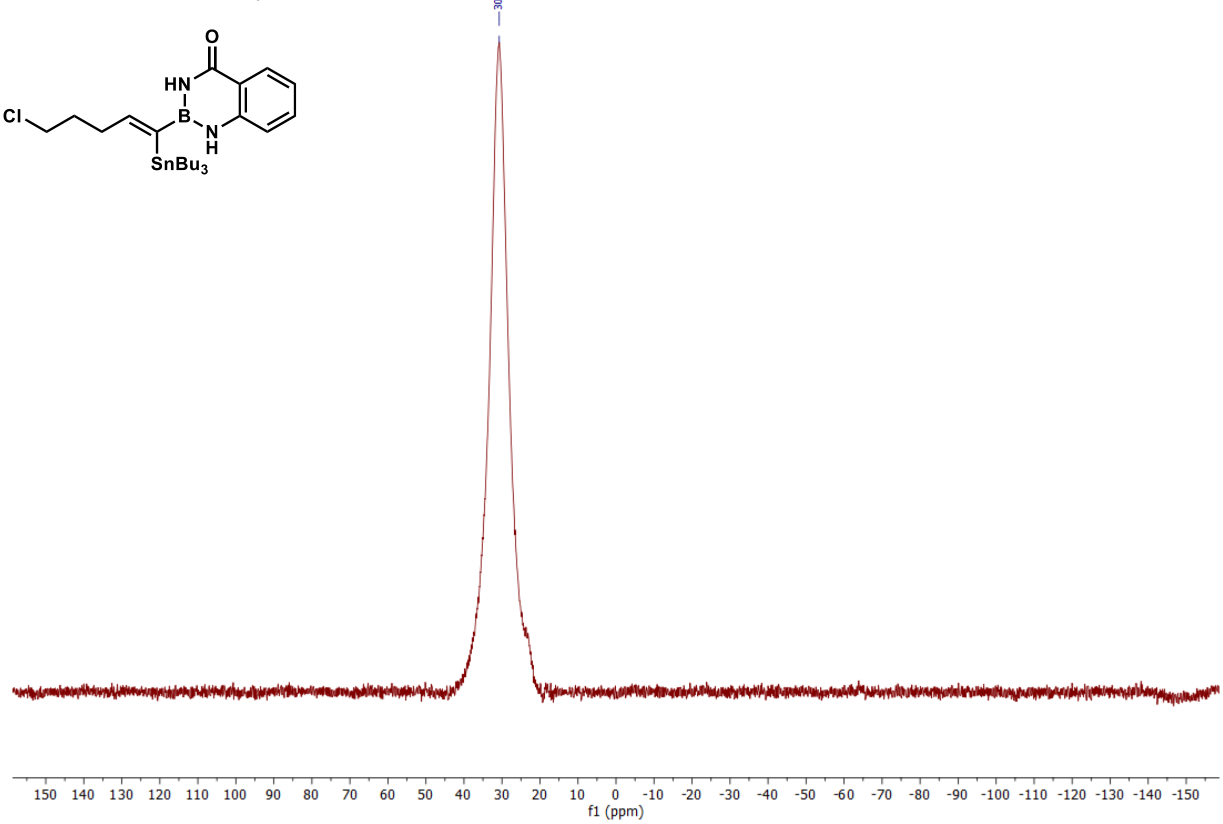

^{119}Sn NMR (149 MHz, $CDCl_3$)

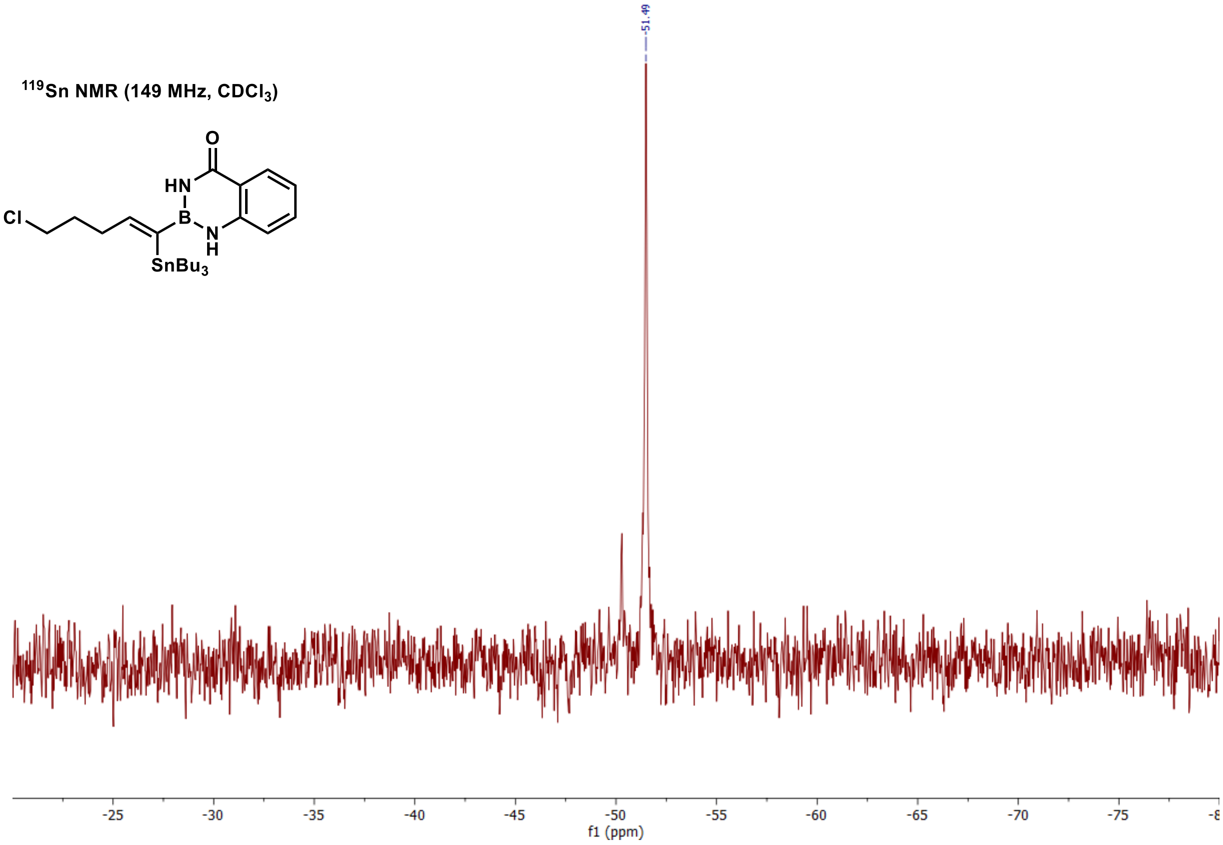

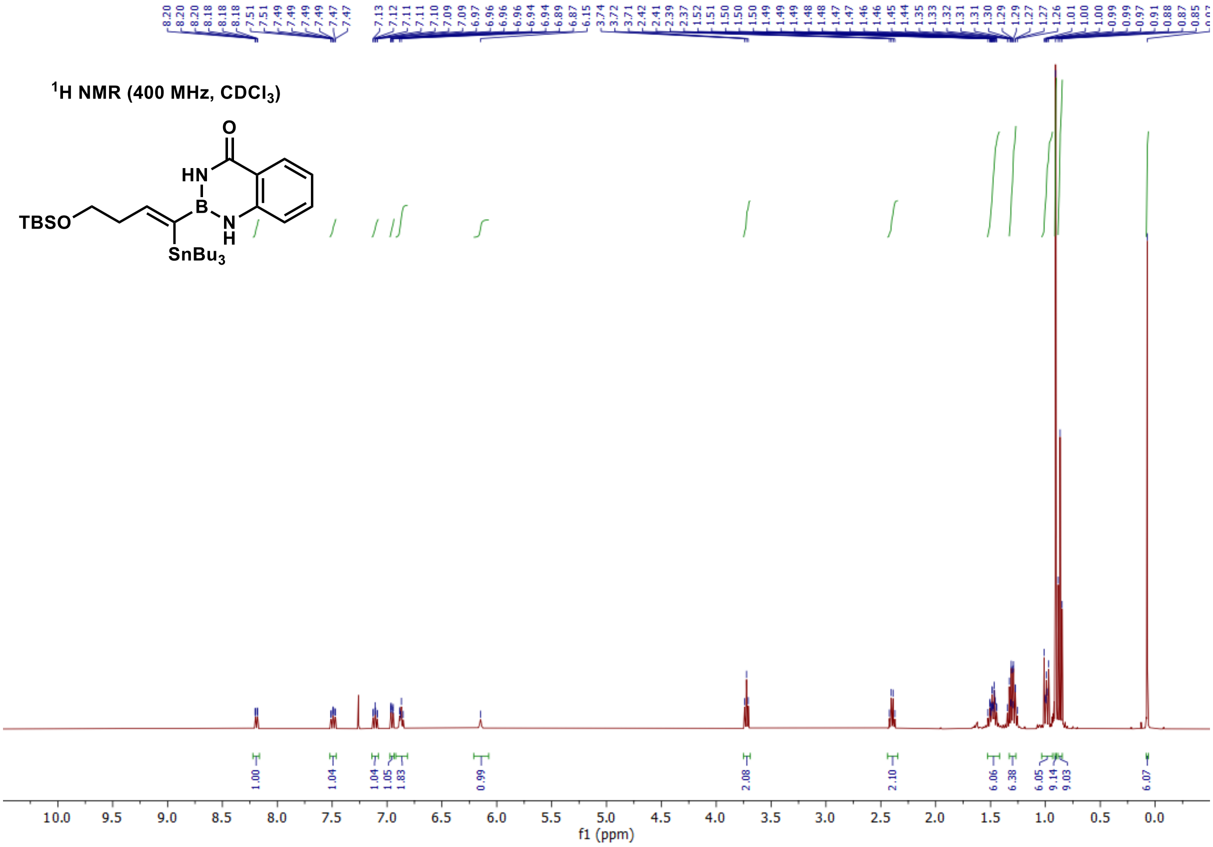

1H NMR (400 MHz, CDCl3)
TBSO
HN
B
N
H
O
SnBu3
f1 (ppm)

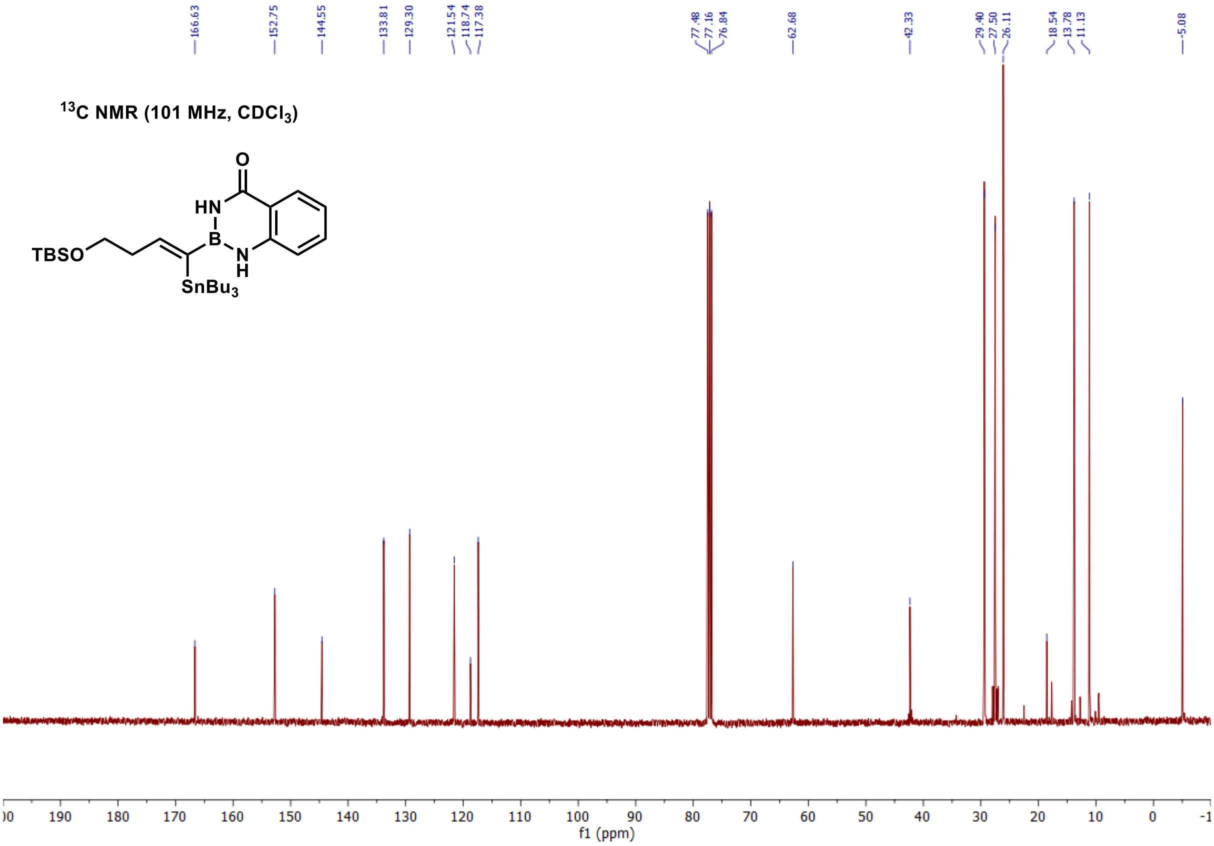

13C NMR (101 MHz, CDCl3)
TBSO
HN
B
N
H
O
SnBu3
166.63
152.75
144.55
133.81
129.30
121.54
118.74
117.38
77.48
77.16
76.84
62.68
42.33
29.40
27.50
26.11
18.54
13.78
11.13
-5.08
f1 (ppm)

^{11}B NMR (128 MHz, $CDCl_3$)

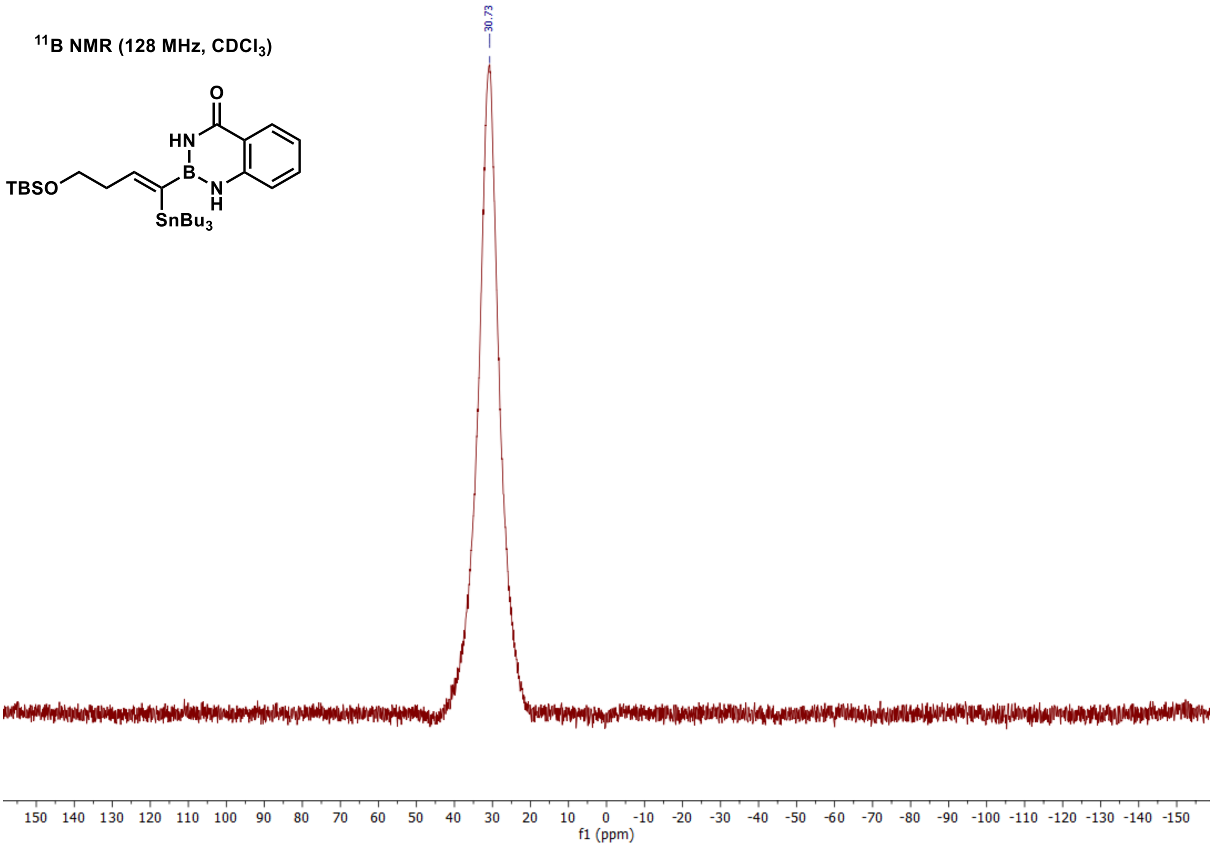

^{119}Sn NMR (149 MHz, $CDCl_3$)

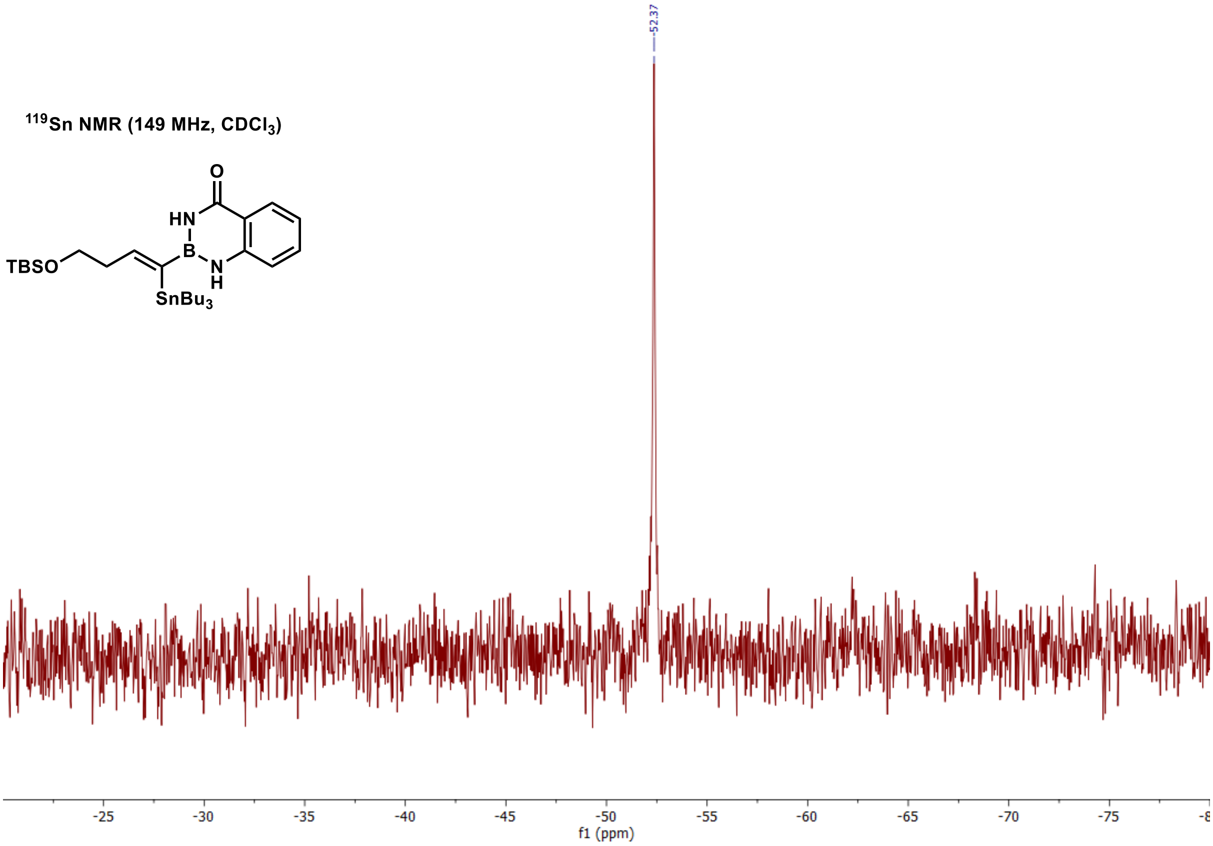

^{1}H NMR (400 MHz, $CDCl_3$)

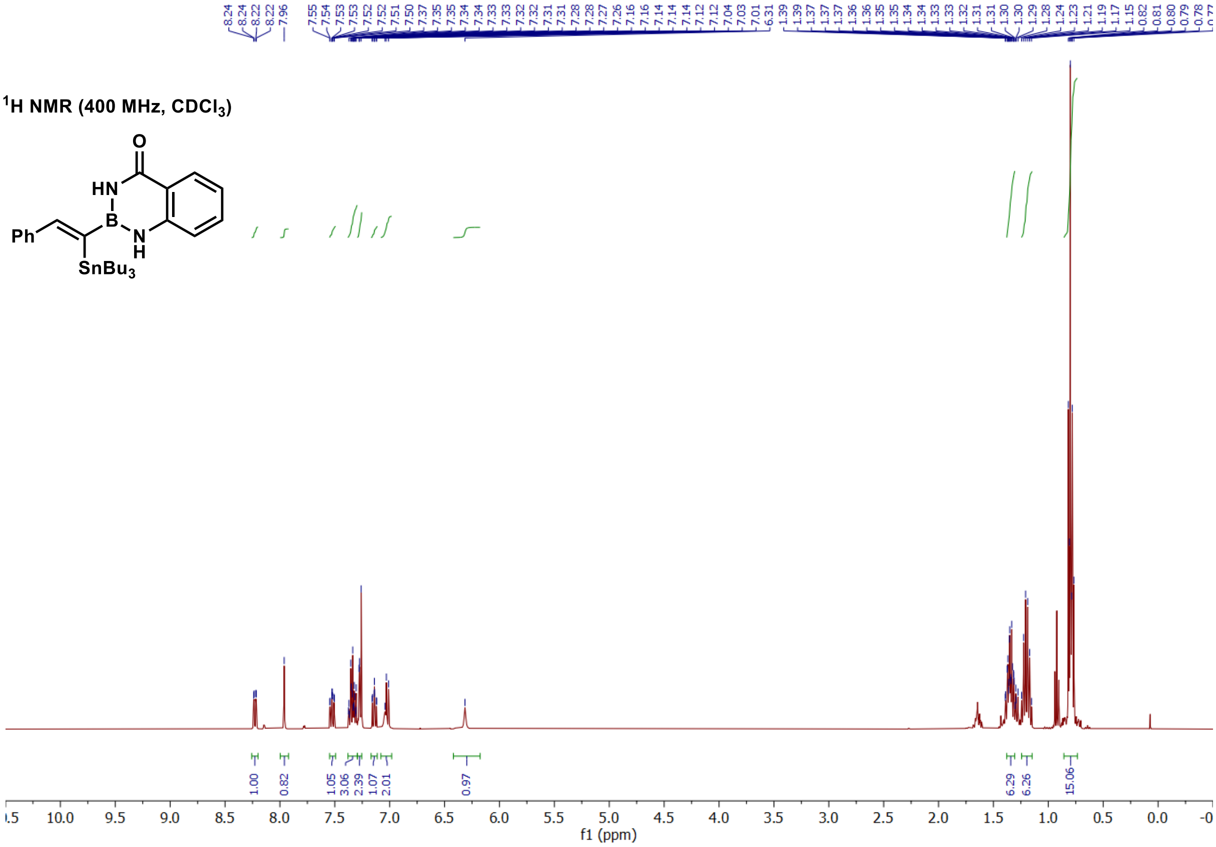

^{13}C NMR (101 MHz, $CDCl_3$)

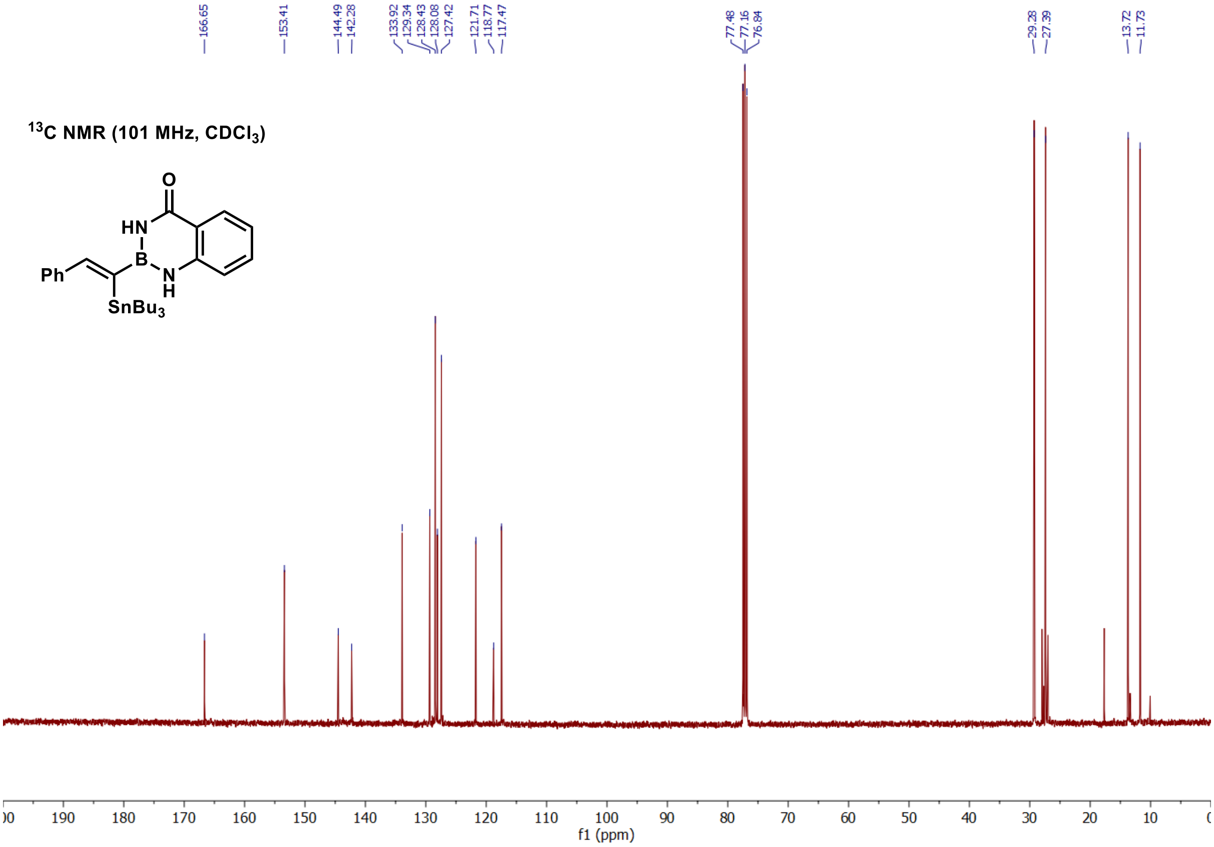

^{11}B NMR (128 MHz, $CDCl_3$)

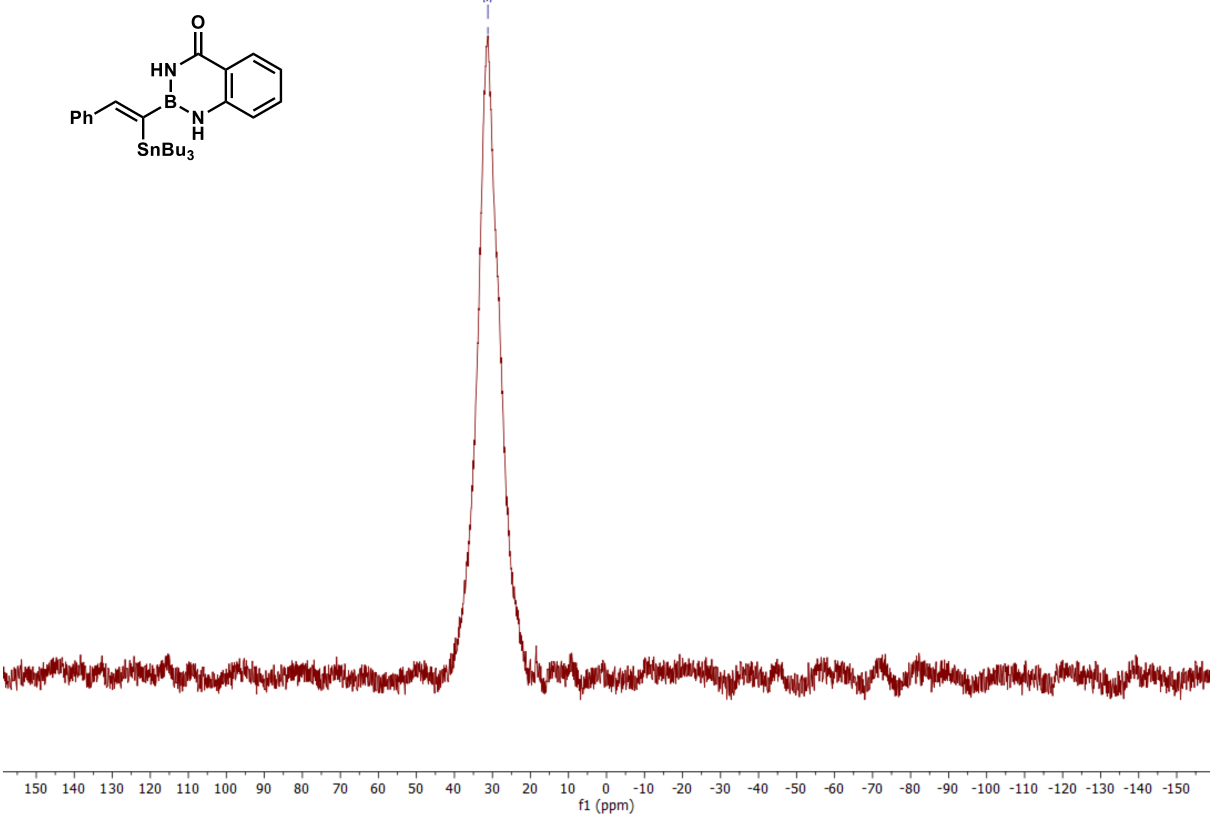

^{119}Sn NMR (149 MHz, $CDCl_3$)

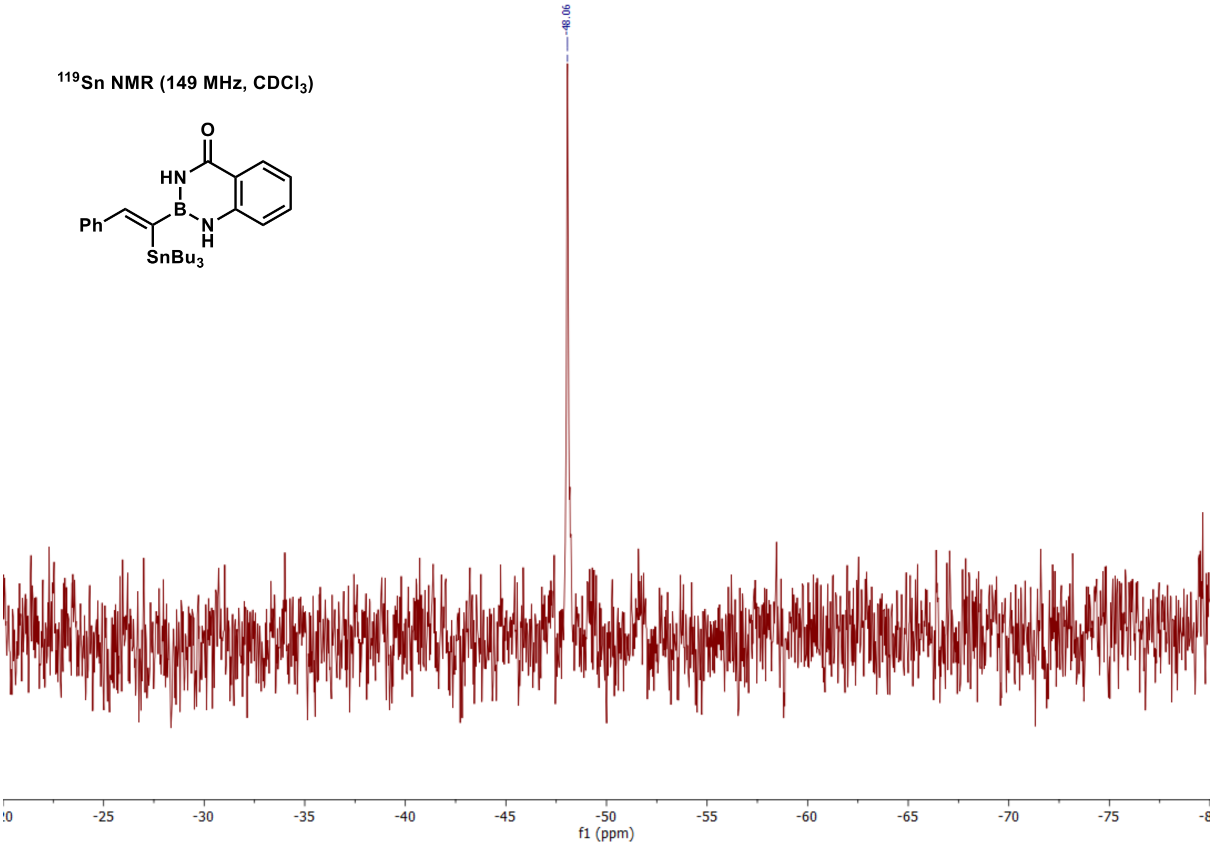

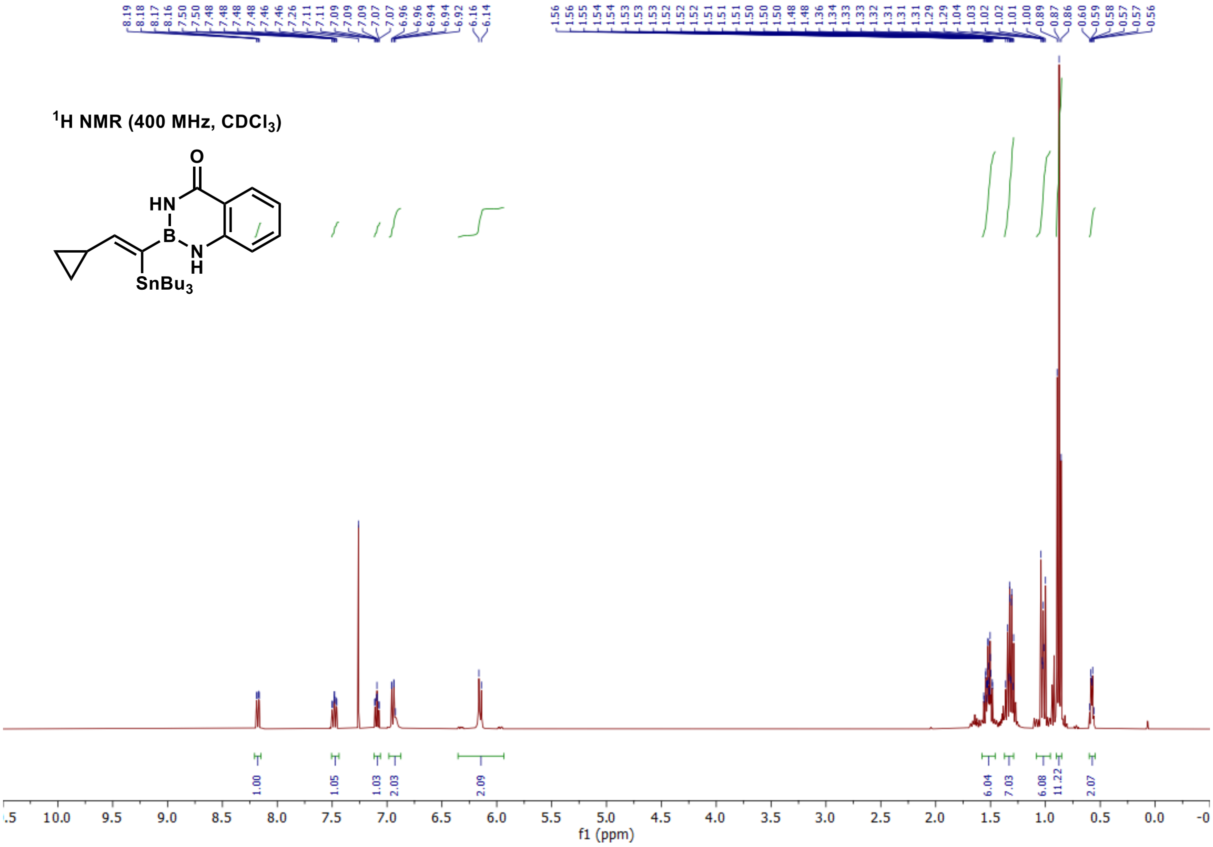
^{1}H NMR (400 MHz, CDCl$_3$)
f1 (ppm)

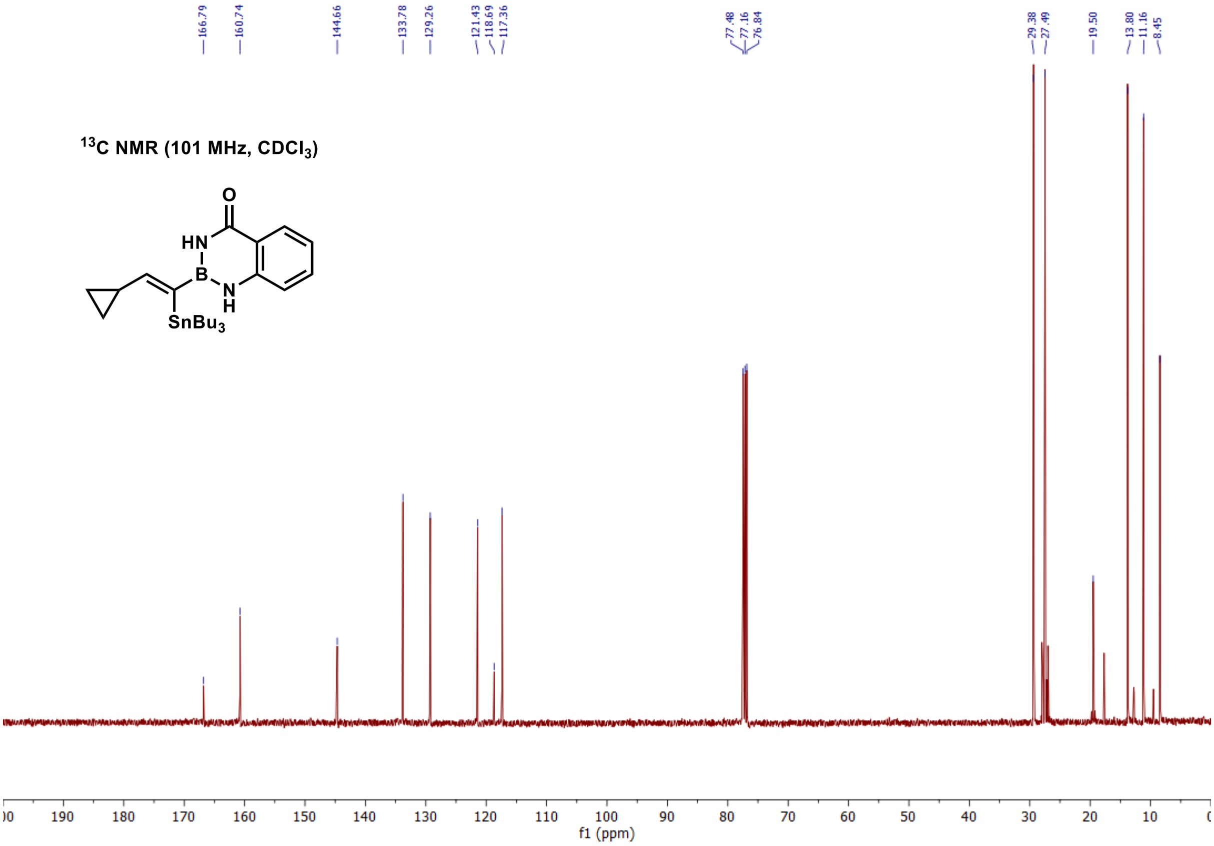
^{13}C NMR (101 MHz, CDCl$_3$)
166.79
160.74
144.66
133.78
129.26
121.43
118.69
117.36
77.48
77.16
76.84
29.38
27.49
19.50
13.80
11.16
8.45
f1 (ppm)

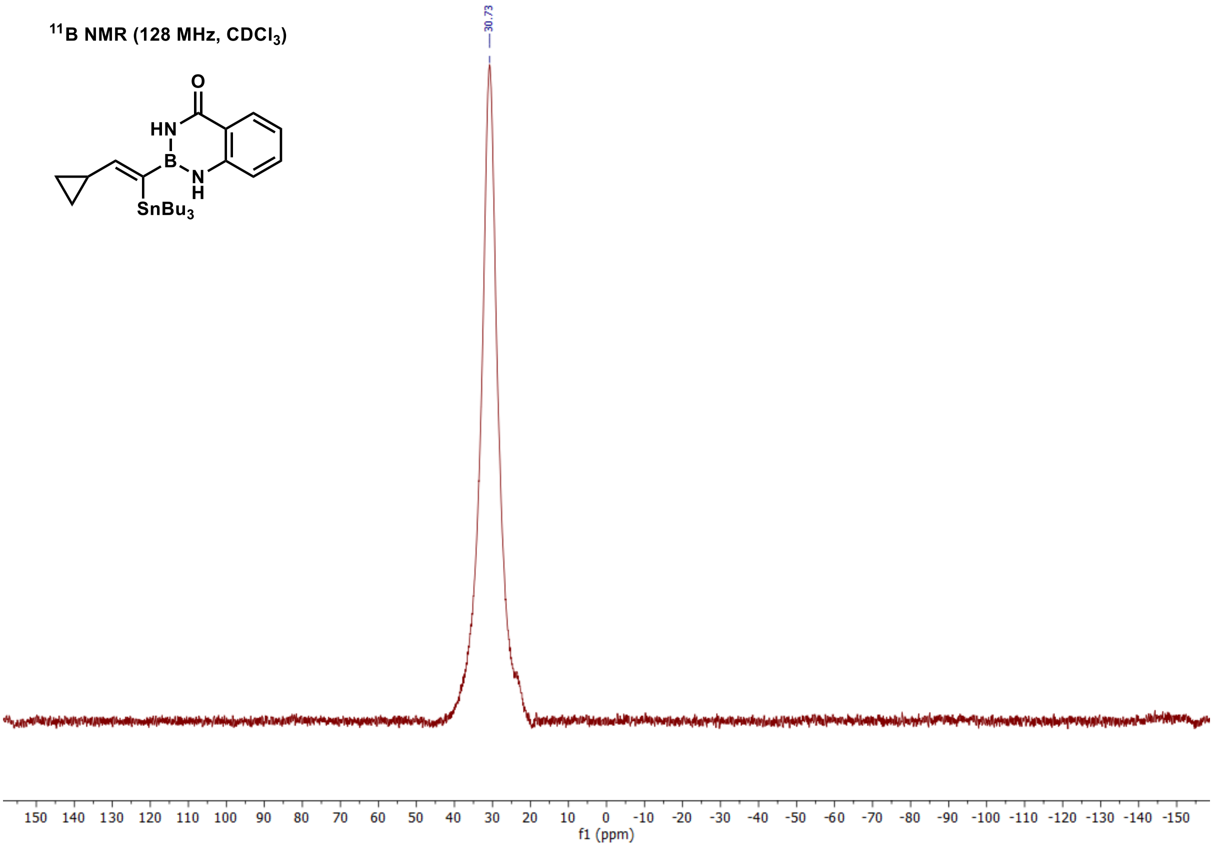

^{11}B NMR (128 MHz, $CDCl_3$)
30.73
O
HN
B
N
H
$SnBu_3$
150 140 130 120 110 100 90 80 70 60 50 40 30 20 10 0 -10 -20 -30 -40 -50 -60 -70 -80 -90 -100 -110 -120 -130 -140 -150
f1 (ppm)

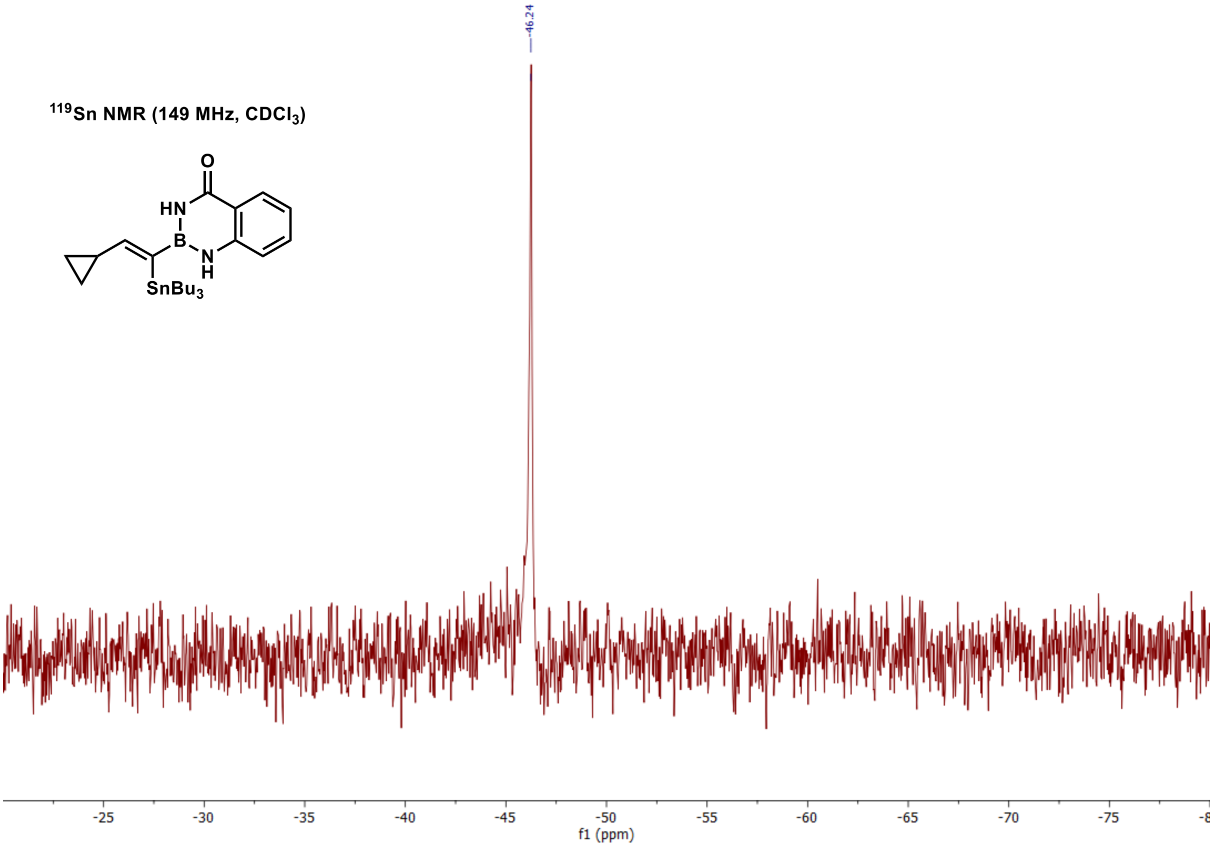

^{119}Sn NMR (149 MHz, $CDCl_3$)
-46.24
O
HN
B
N
H
$SnBu_3$
-25 -30 -35 -40 -45 -50 -55 -60 -65 -70 -75
f1 (ppm)

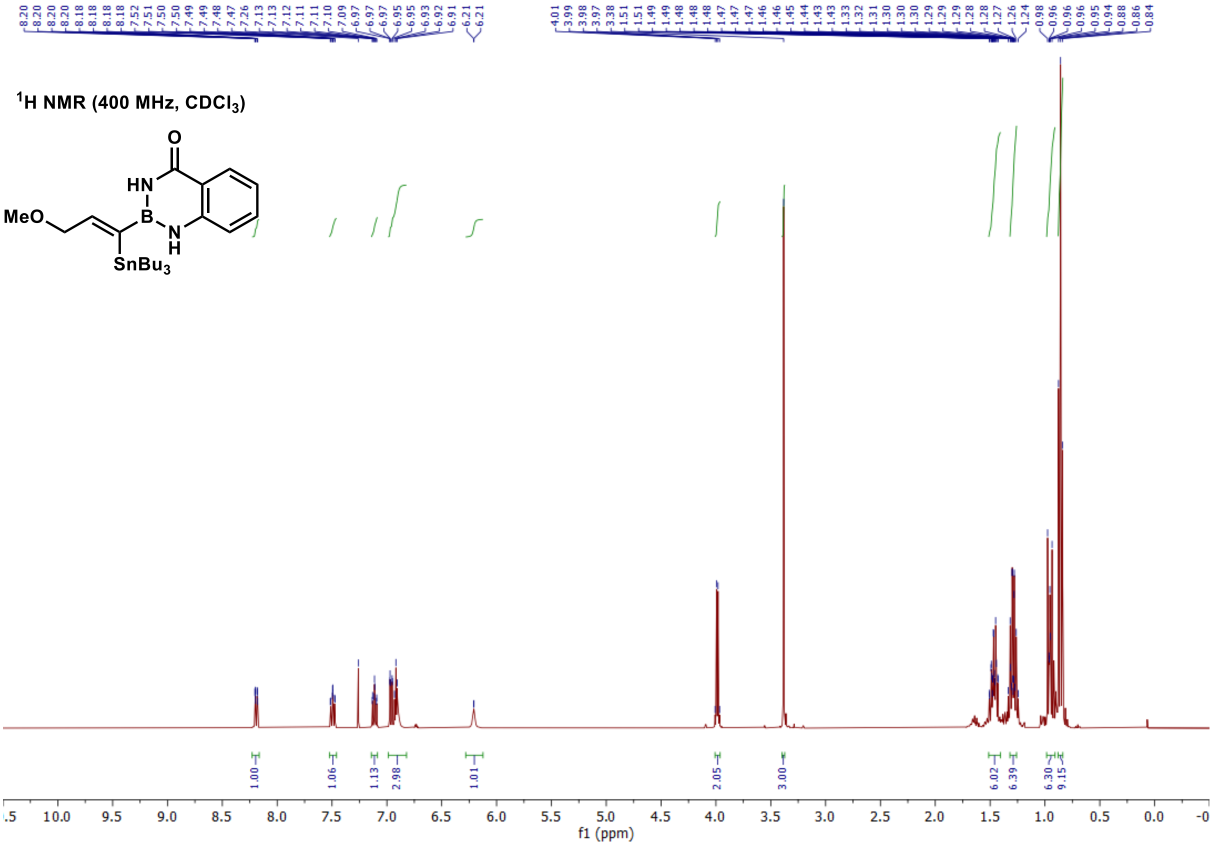
^{1}H NMR (400 MHz, CDCl$_3$)
f1 (ppm)

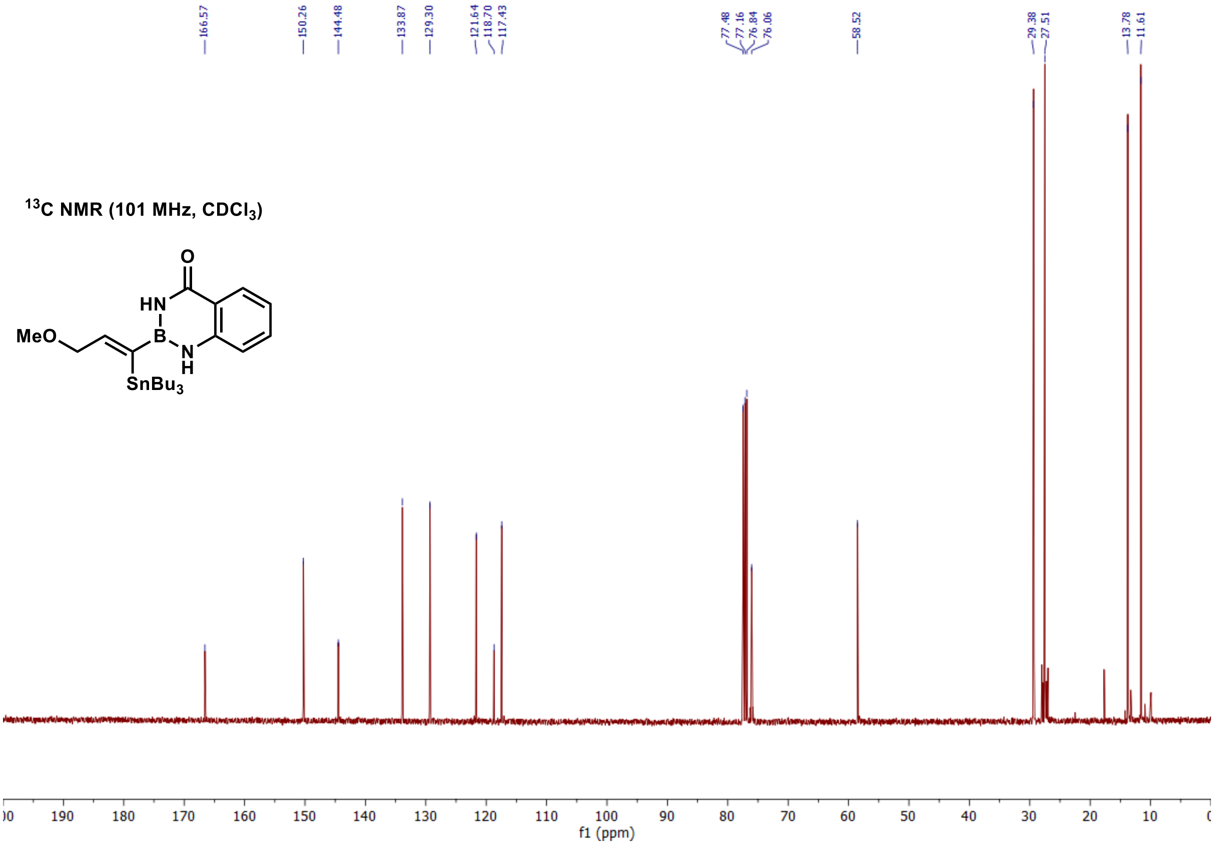
^{13}C NMR (101 MHz, CDCl$_3$)
f1 (ppm)

^{11}B NMR (128 MHz, $CDCl_3$)

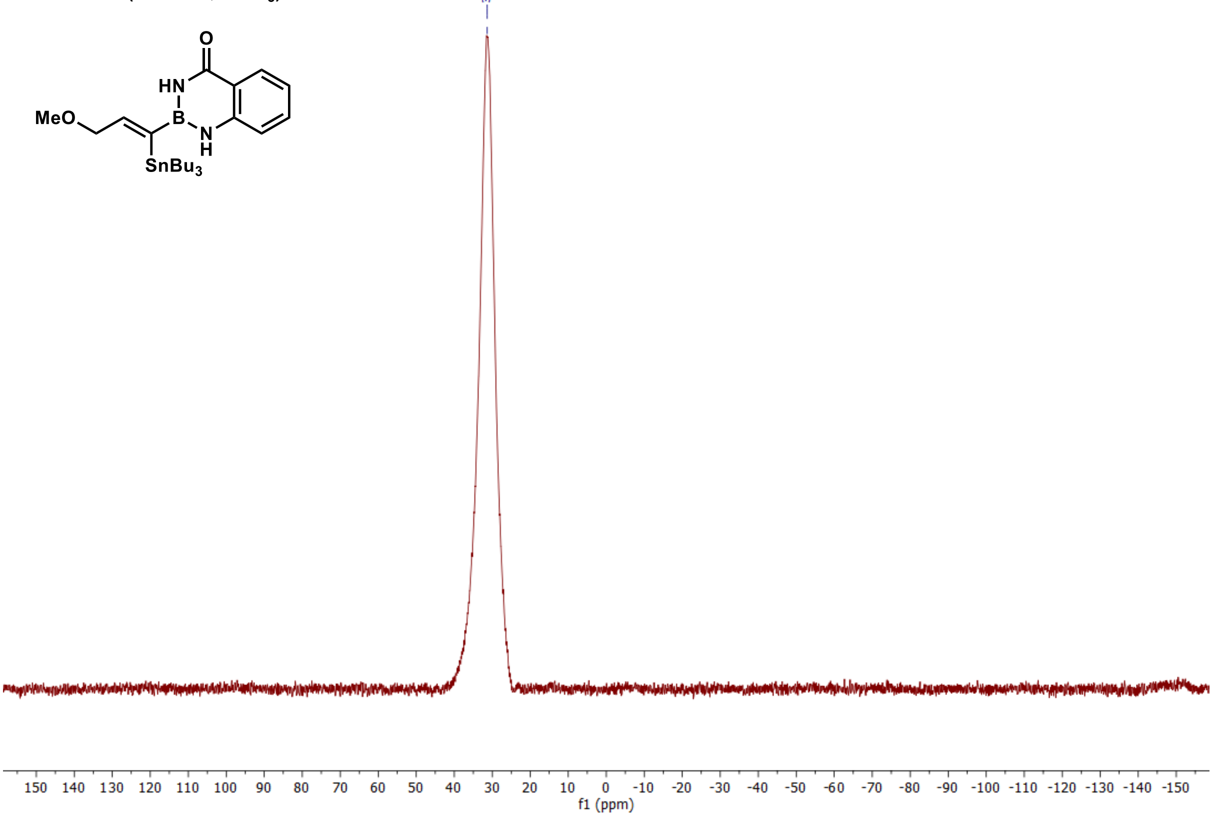

^{119}Sn NMR (149 MHz, $CDCl_3$)

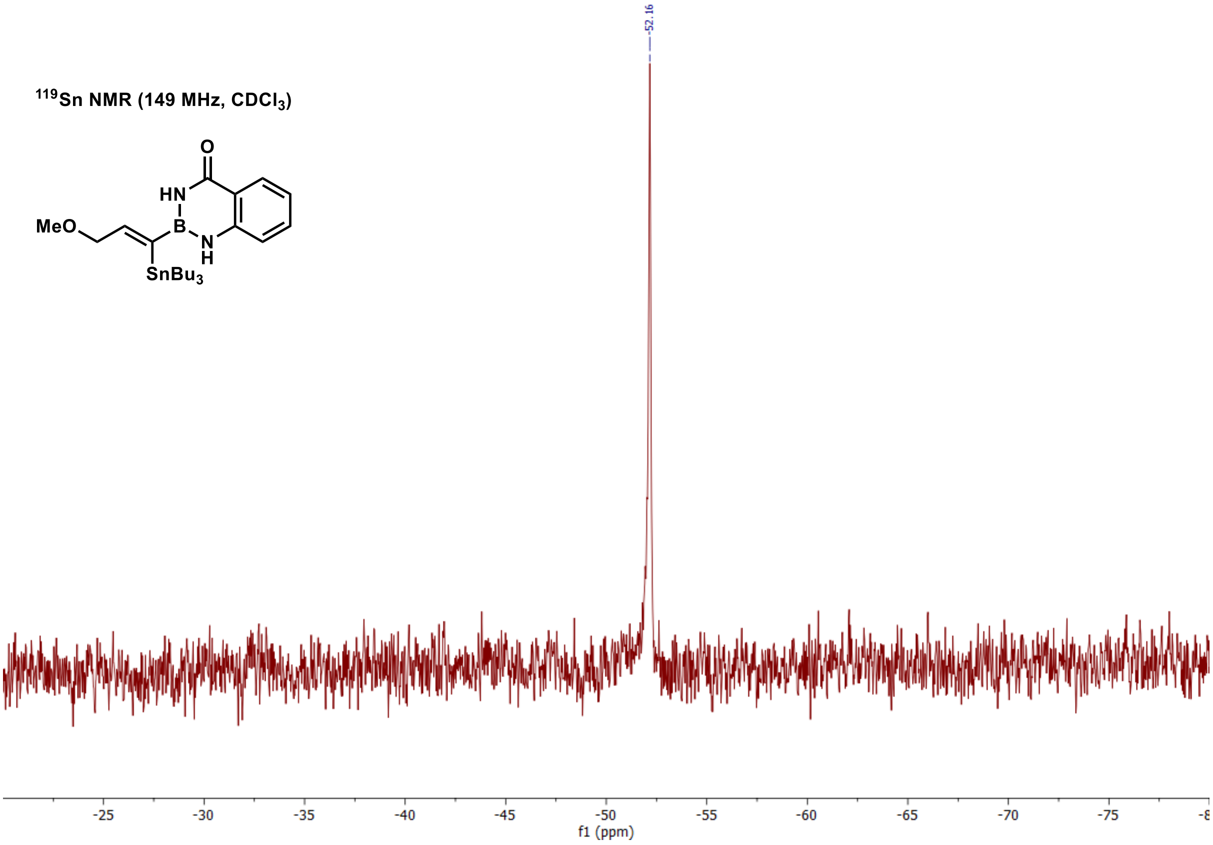

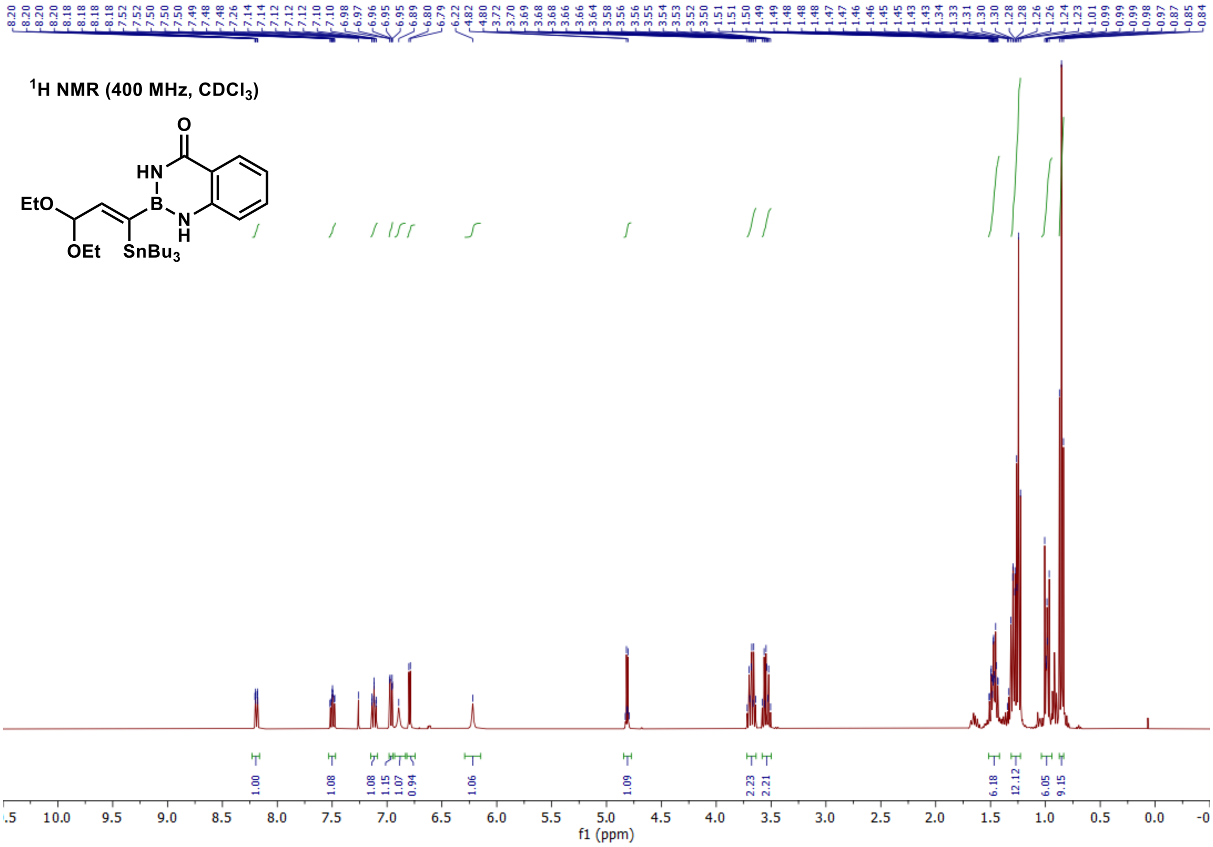
1H NMR (400 MHz, CDCl3)
HN
B
N
H
O
EtO
OEt
SnBu3
f1 (ppm)

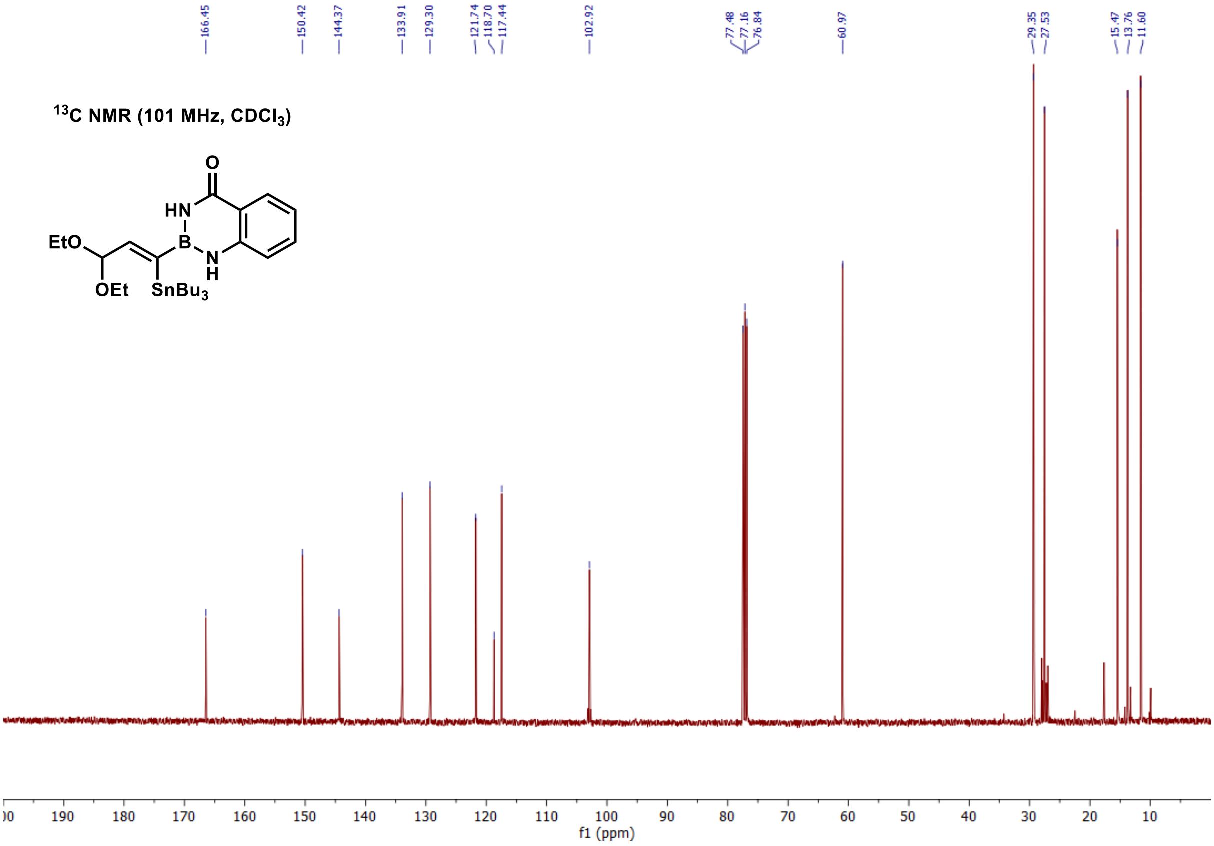
13C NMR (101 MHz, CDCl3)
HN
B
N
H
O
EtO
OEt
SnBu3
166.45
150.42
144.37
133.91
129.30
121.74
118.70
117.44
102.92
77.48
77.16
76.84
60.97
29.35
27.53
15.47
13.76
11.60
f1 (ppm)

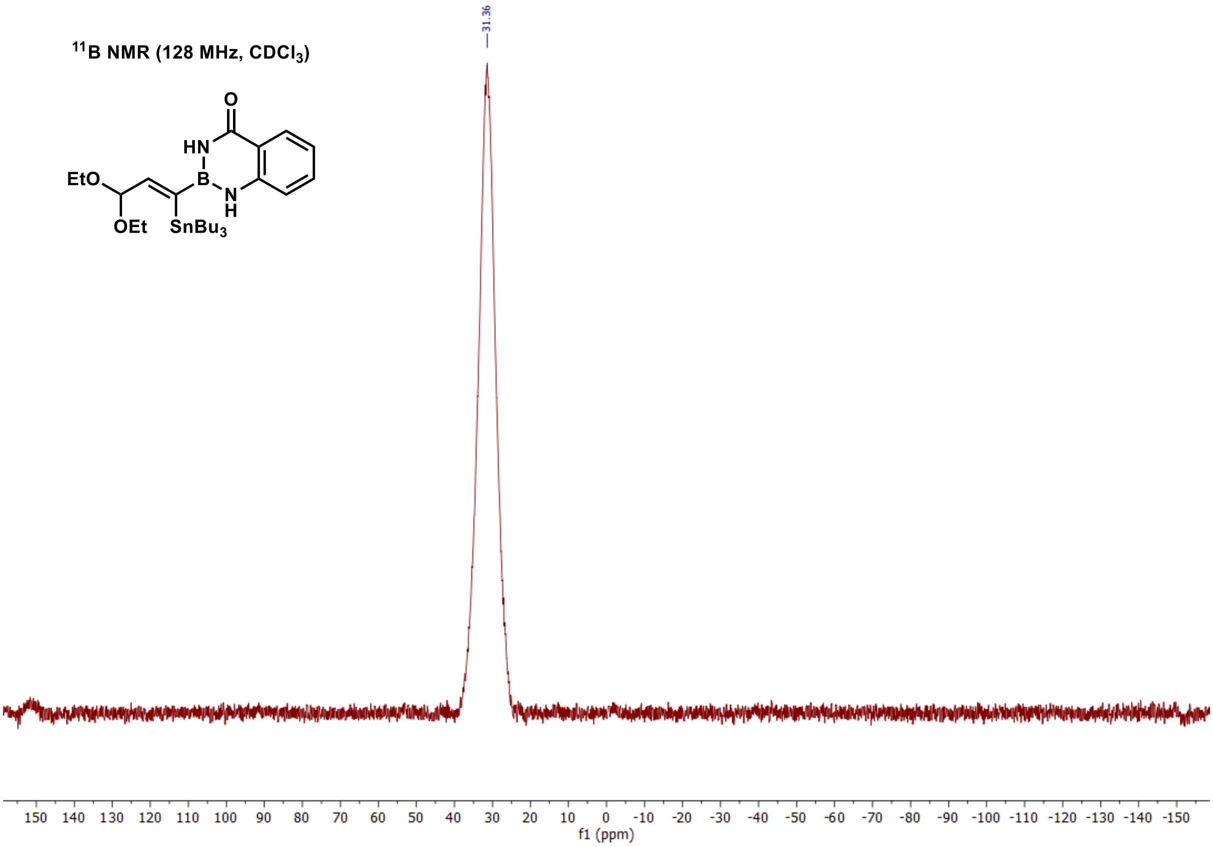

31.36
^{11}B NMR (128 MHz, CDCl$_3$)
O
HN
B
N
H
EtO
OEt
SnBu$_3$
150 140 130 120 110 100 90 80 70 60 50 40 30 20 10 0 -10 -20 -30 -40 -50 -60 -70 -80 -90 -100 -110 -120 -130 -140 -150
f1 (ppm)

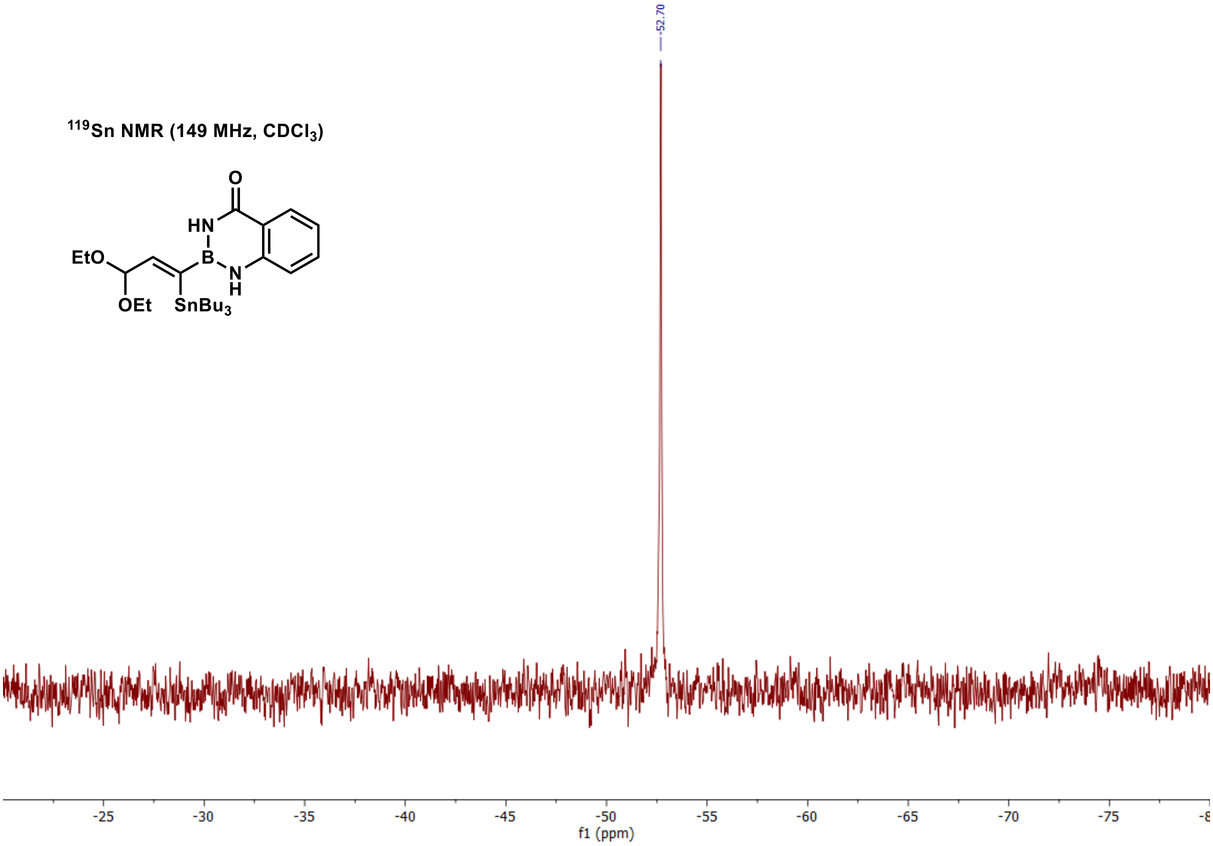

-52.70
^{119}Sn NMR (149 MHz, CDCl$_3$)
O
HN
B
N
H
EtO
OEt
SnBu$_3$
-25 -30 -35 -40 -45 -50 -55 -60 -65 -70 -75
f1 (ppm)

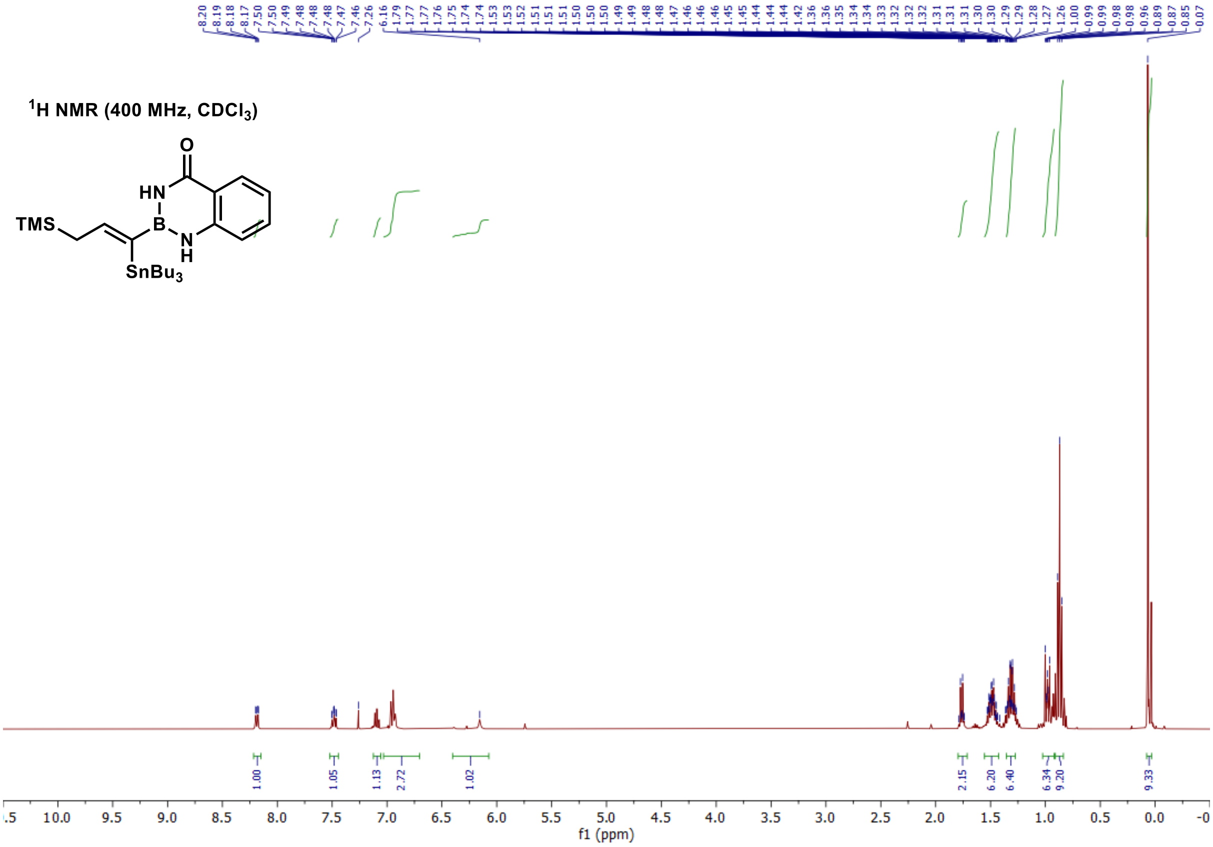
^{1}H NMR (400 MHz, CDCl$_3$)
TMS
SnBu$_3$
f1 (ppm)

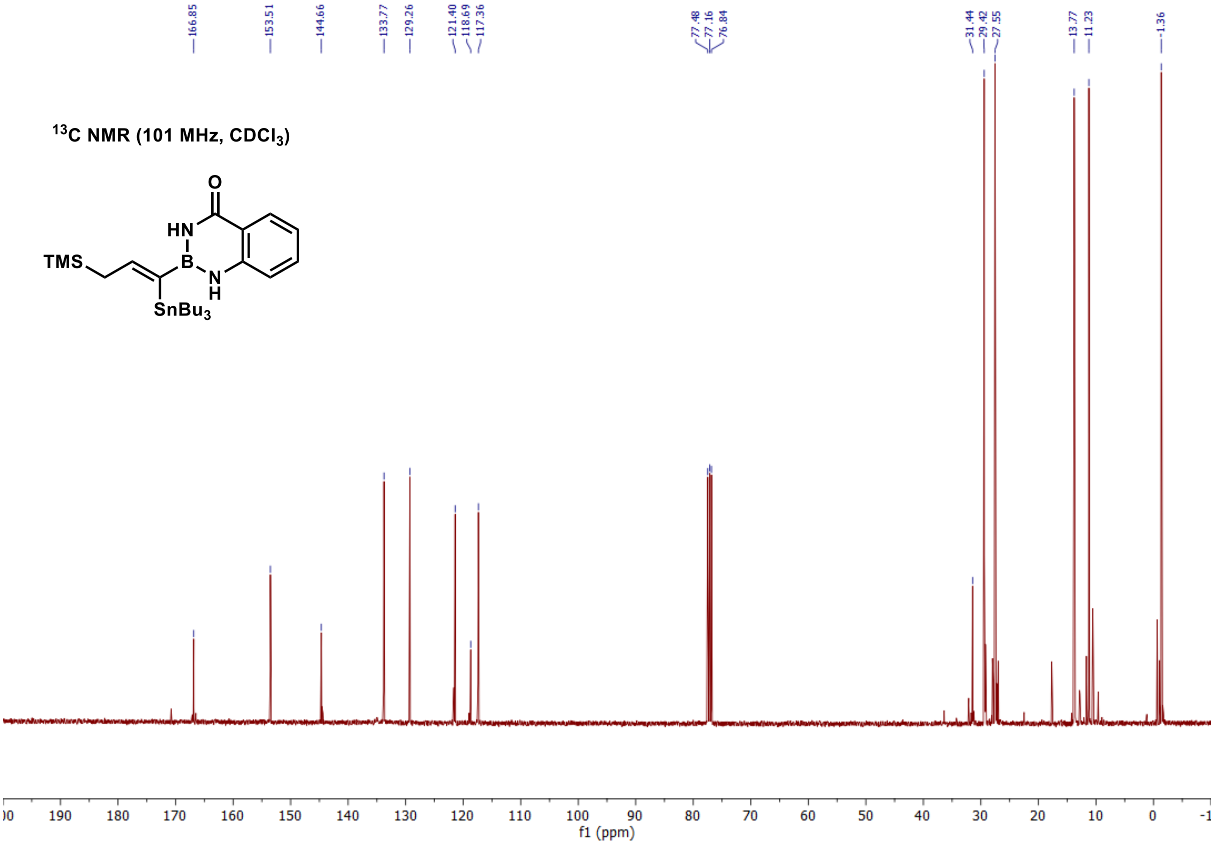
^{13}C NMR (101 MHz, CDCl$_3$)
TMS
SnBu$_3$
f1 (ppm)

^{11}B NMR (128 MHz, $CDCl_3$)

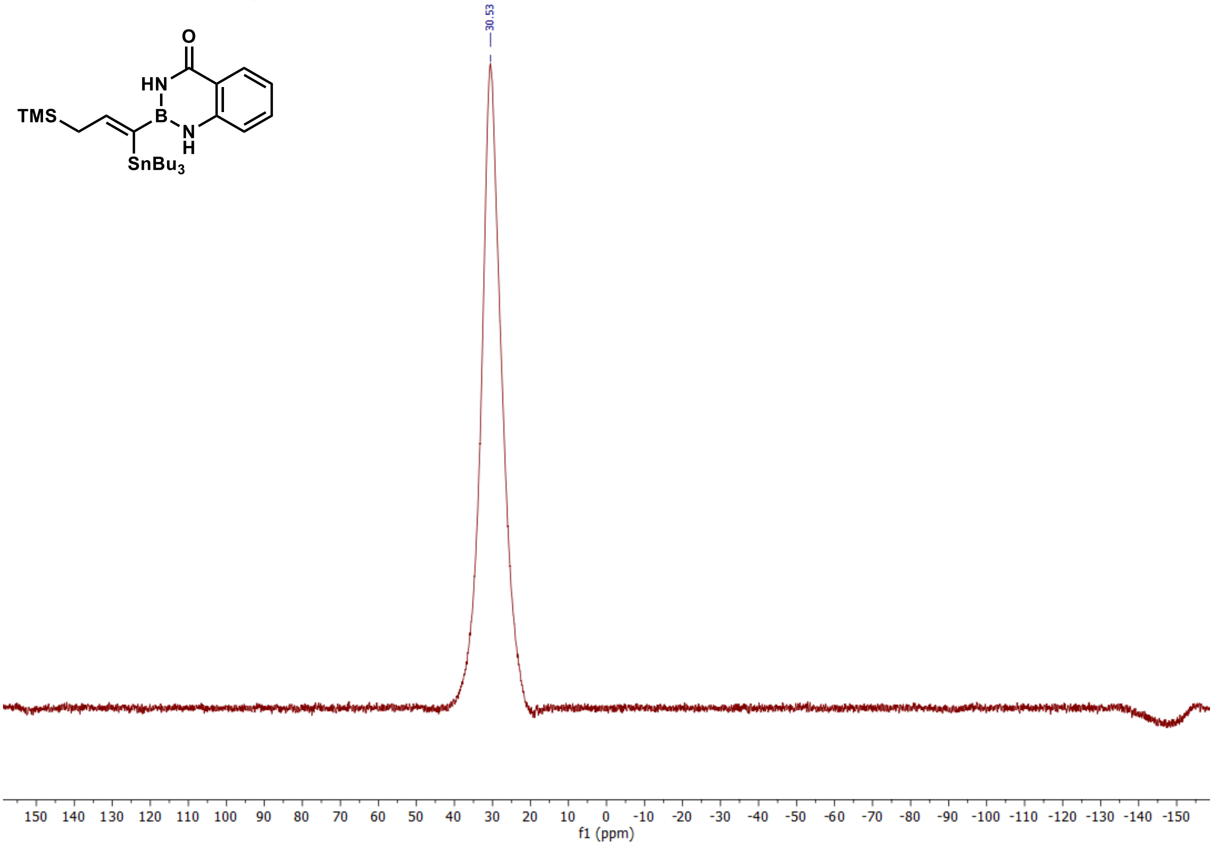

^{119}Sn NMR (149 MHz, $CDCl_3$)

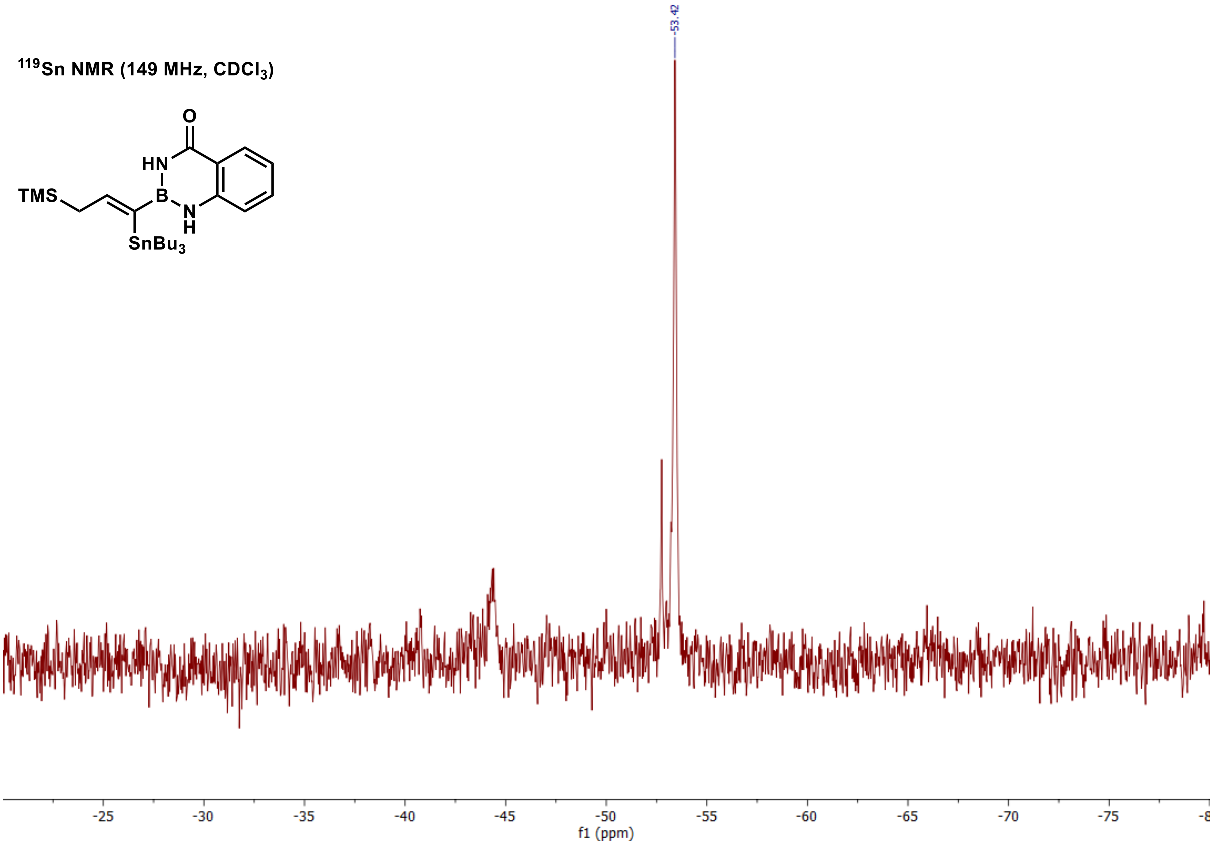

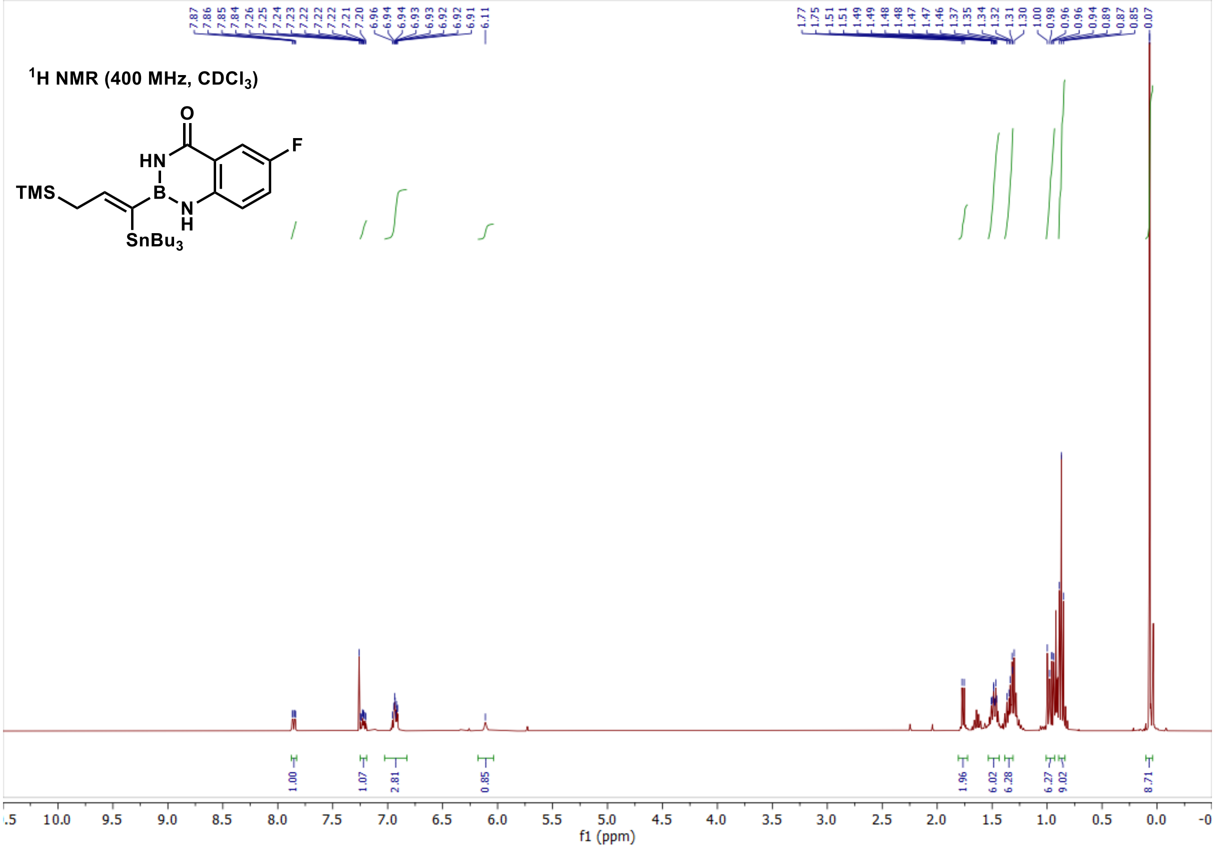
7.87 7.86 7.85 7.84 7.26 7.25 7.24 7.23 7.22 7.22 7.22 7.21 7.20 6.96 6.94 6.94 6.93 6.93 6.92 6.92 6.91 6.11
1.77 1.75 1.51 1.51 1.49 1.49 1.48 1.48 1.47 1.47 1.46 1.37 1.35 1.34 1.32 1.31 1.30 1.00 0.98 0.96 0.96 0.94 0.89 0.87 0.85 0.07
^{1}H NMR (400 MHz, CDCl$_3$)
O
HN
B
N
H
F
TMS
SnBu$_3$
1.00 1.07 2.81 0.85 1.96 6.02 6.28 6.27 9.02 8.71
f1 (ppm)

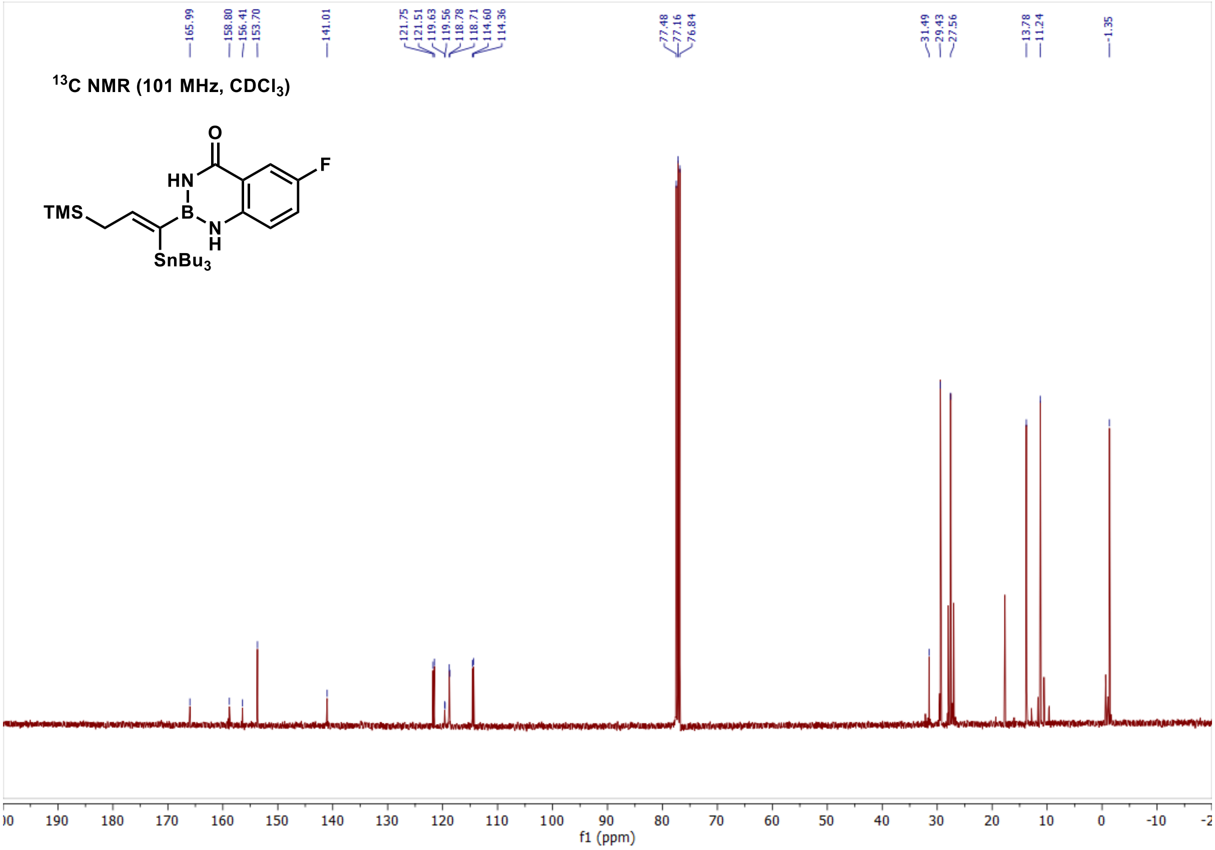
165.99 158.80 156.41 153.70 141.01 121.75 121.51 119.63 119.56 118.78 118.71 114.60 114.36 77.48 77.16 76.84 31.49 29.43 27.56 13.78 11.24 -1.35
^{13}C NMR (101 MHz, CDCl$_3$)
O
HN
B
N
H
F
TMS
SnBu$_3$
f1 (ppm)

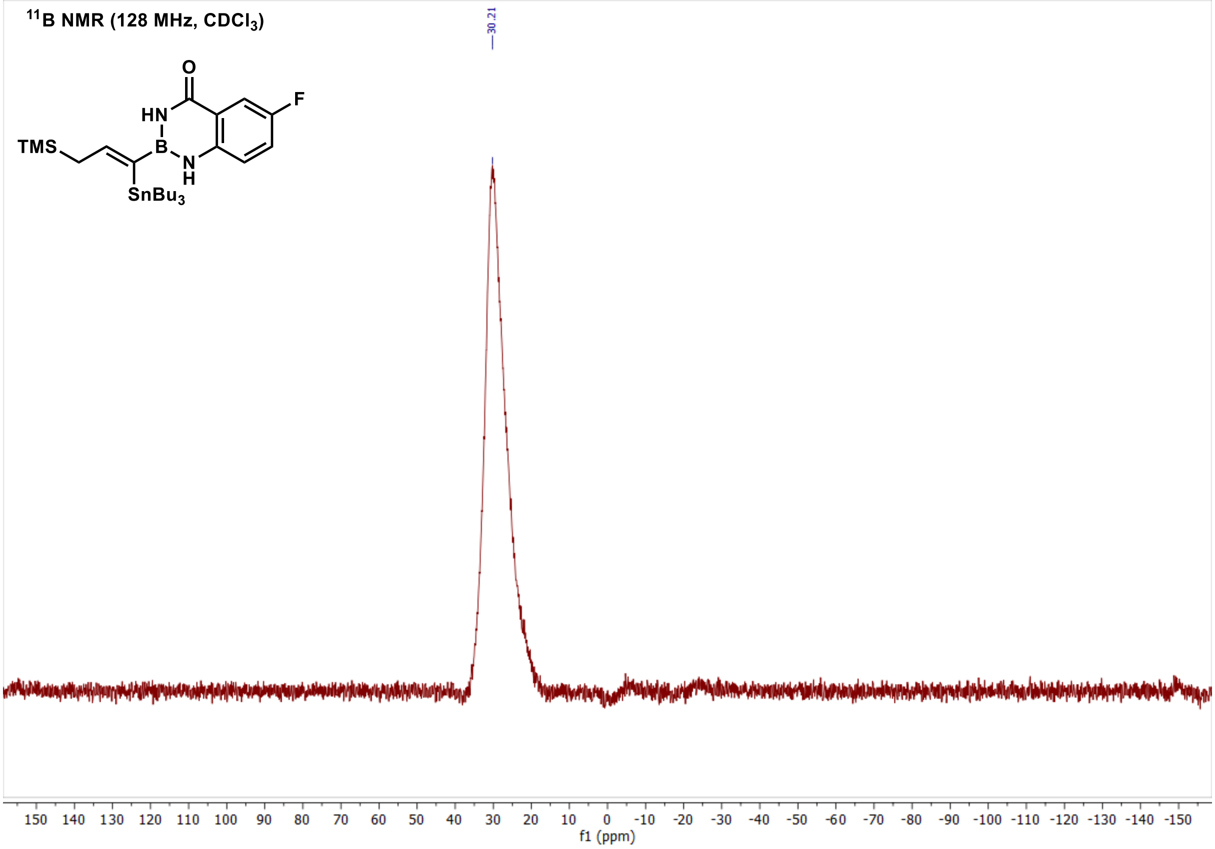

11B NMR (128 MHz, CDCl3)
30.21
O
HN
F
TMS
B
N
H
SnBu3
150 140 130 120 110 100 90 80 70 60 50 40 30 20 10 0 -10 -20 -30 -40 -50 -60 -70 -80 -90 -100 -110 -120 -130 -140 -150
f1 (ppm)

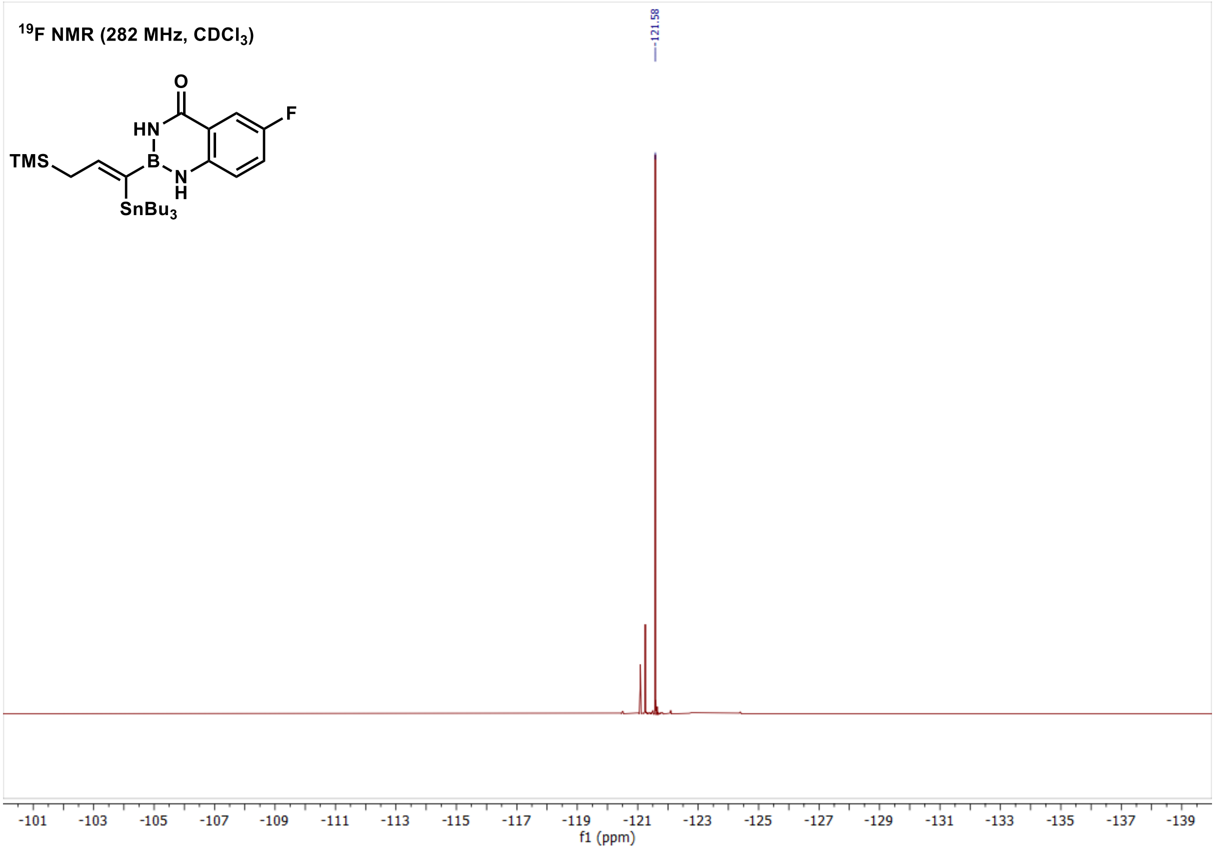

19F NMR (282 MHz, CDCl3)
-121.58
O
HN
F
TMS
B
N
H
SnBu3
-101 -103 -105 -107 -109 -111 -113 -115 -117 -119 -121 -123 -125 -127 -129 -131 -133 -135 -137 -139
f1 (ppm)

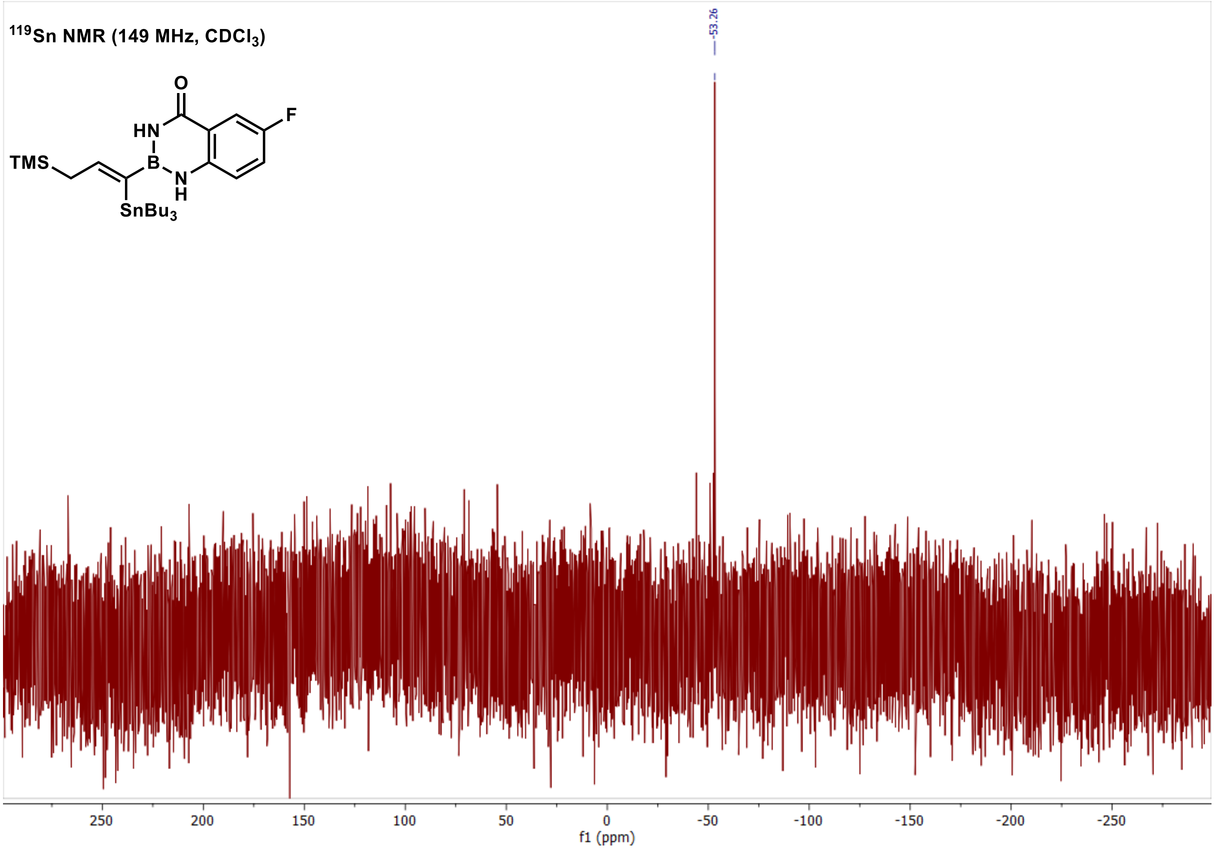

119Sn NMR (149 MHz, CDCl3)
O
HN
F
TMS
B
N
H
SnBu3
-53.26
250
200
150
100
50
0
-50
-100
-150
-200
-250
f1 (ppm)

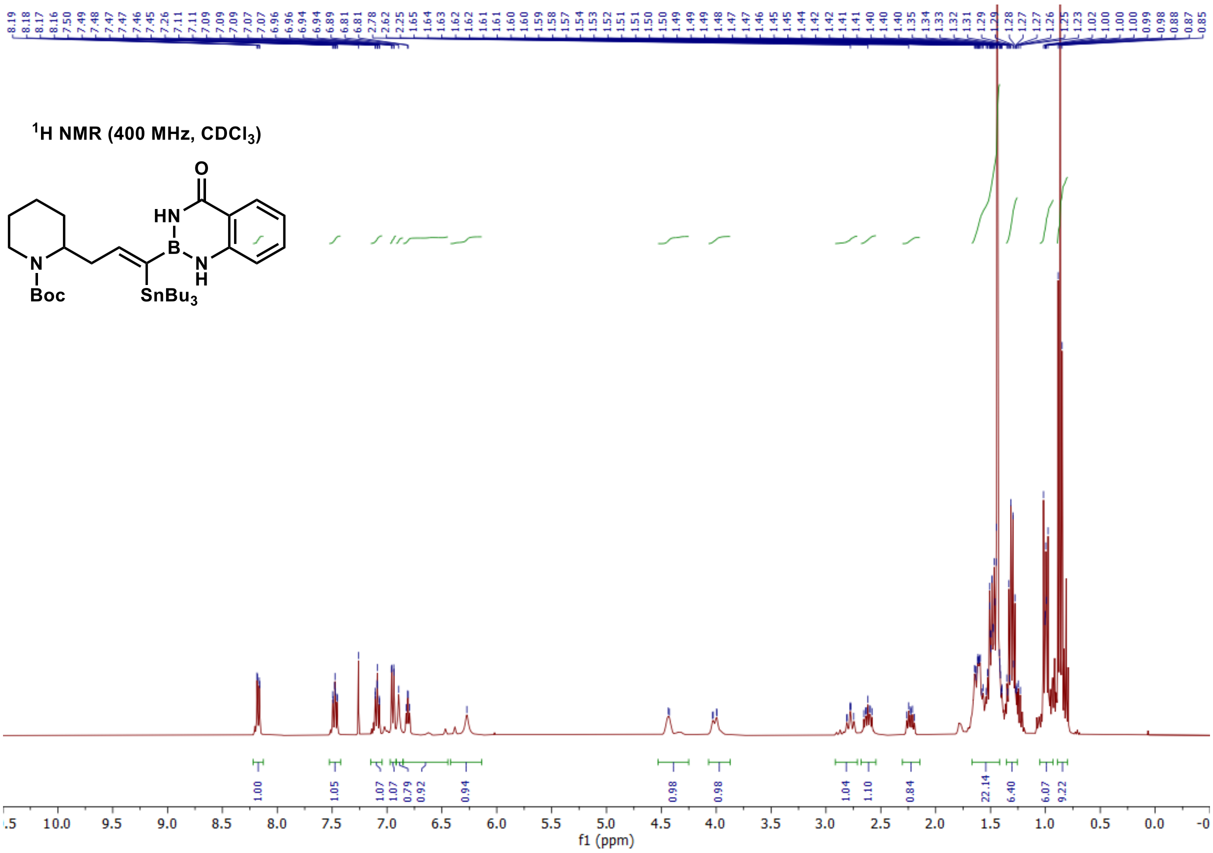
1H NMR (400 MHz, CDCl3)
HN
B
N
H
O
N
Boc
SnBu3
f1 (ppm)

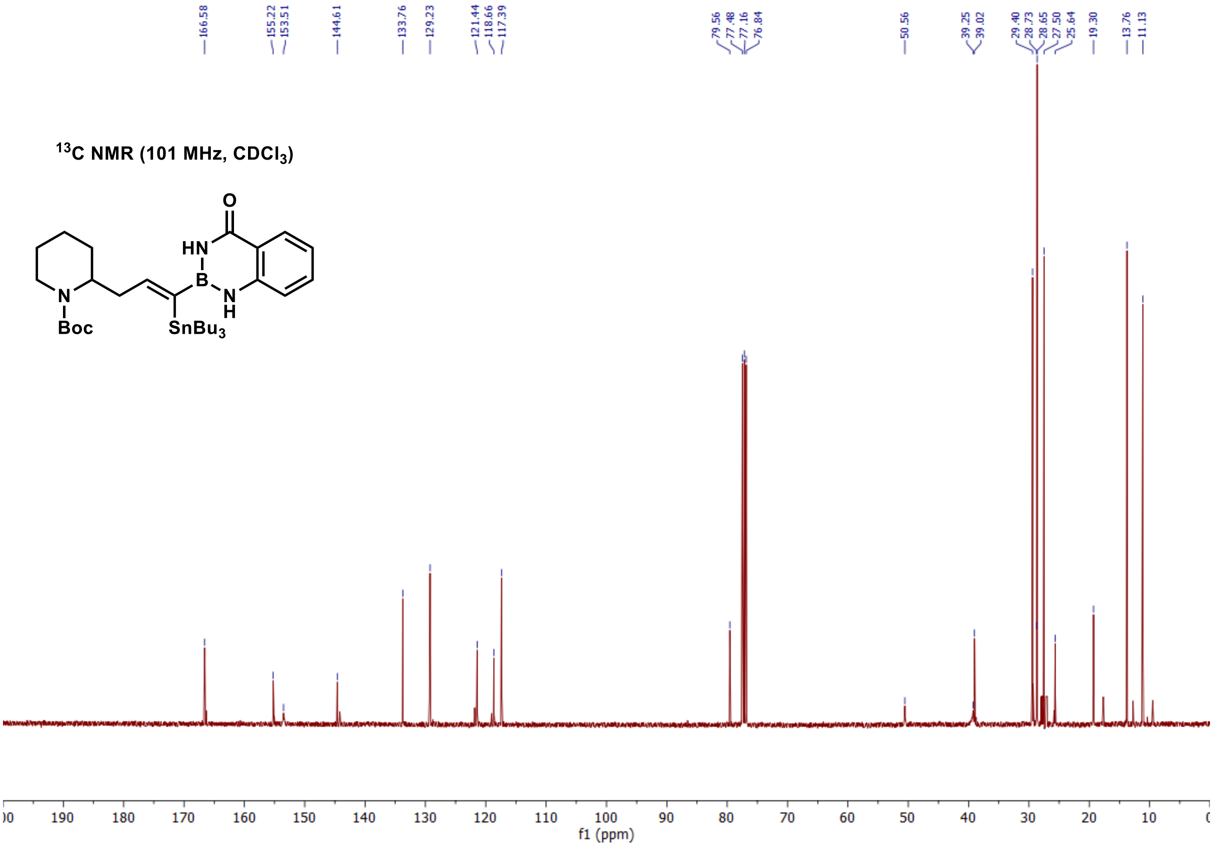
13C NMR (101 MHz, CDCl3)
HN
B
N
H
O
N
Boc
SnBu3
f1 (ppm)

^{11}B NMR (128 MHz, $CDCl_3$)

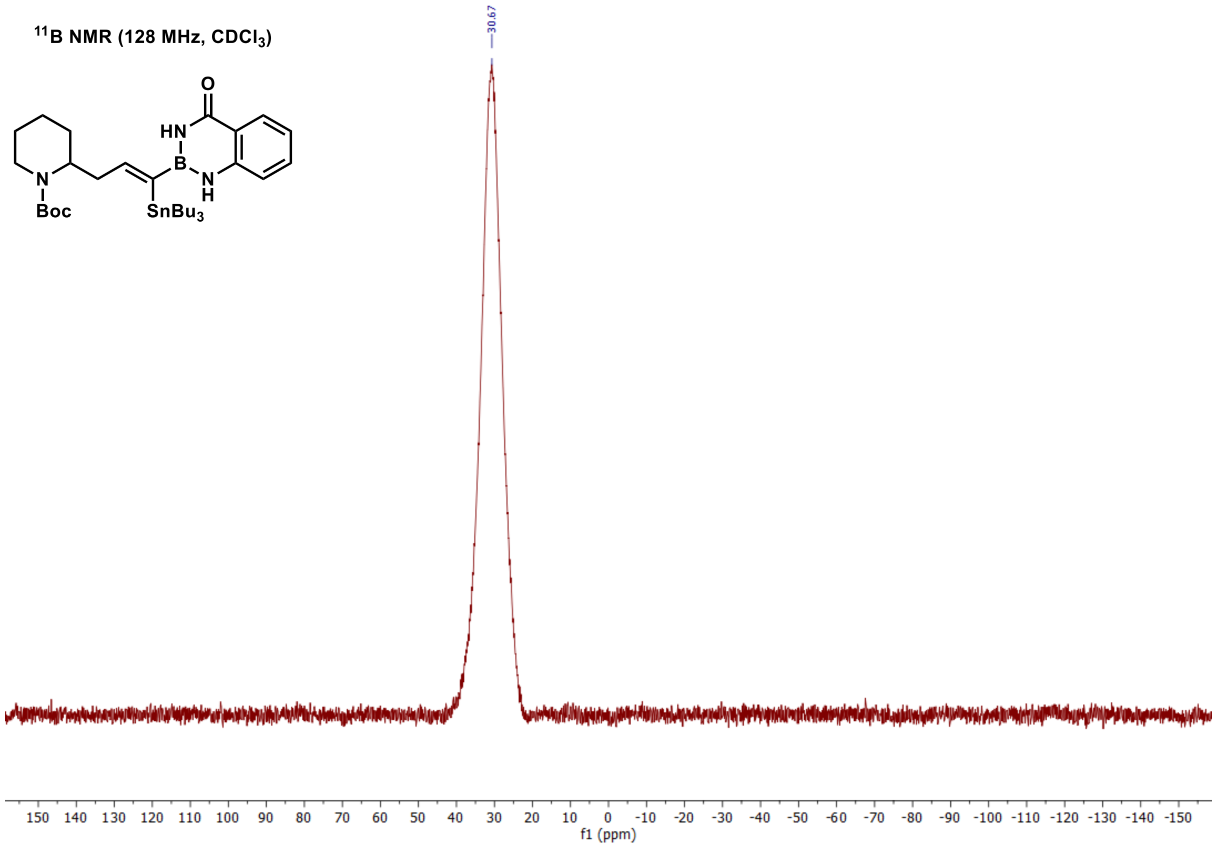

^{119}Sn NMR (149 MHz, $CDCl_3$)

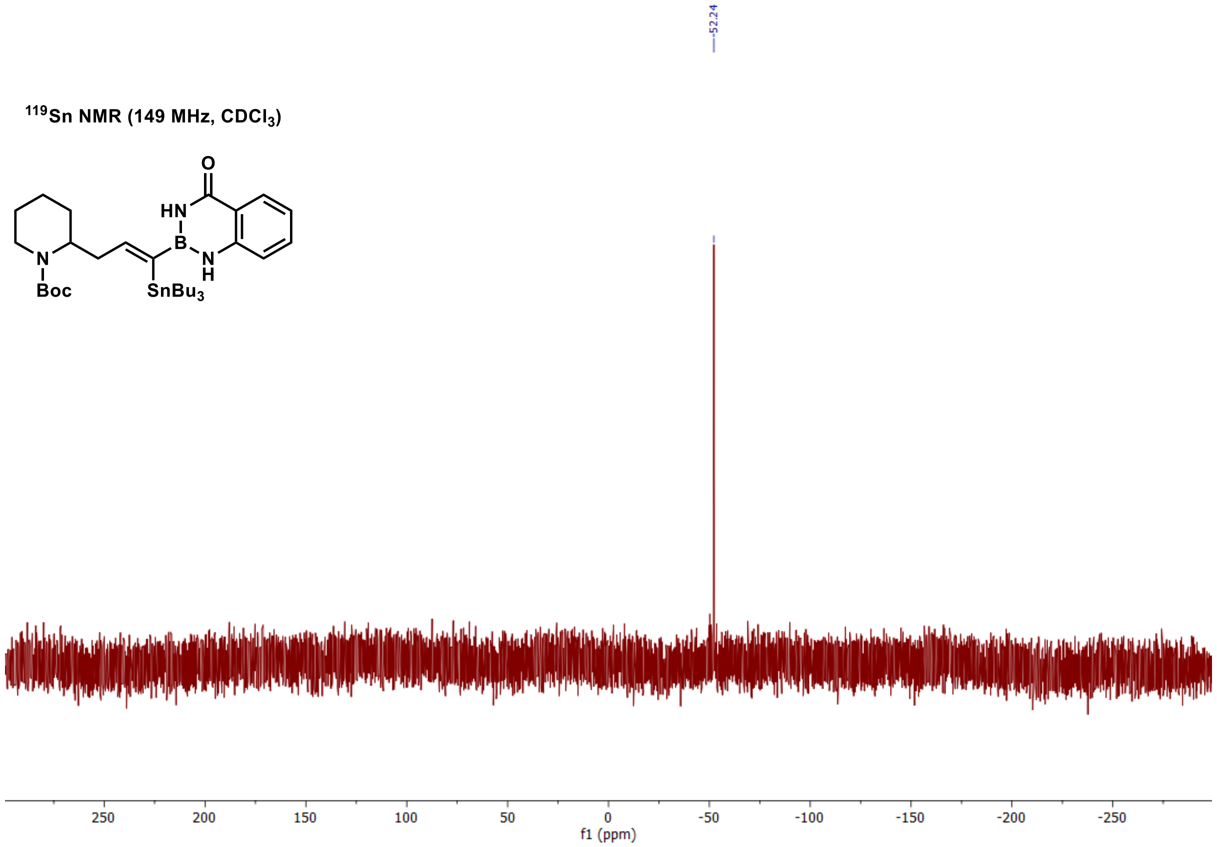

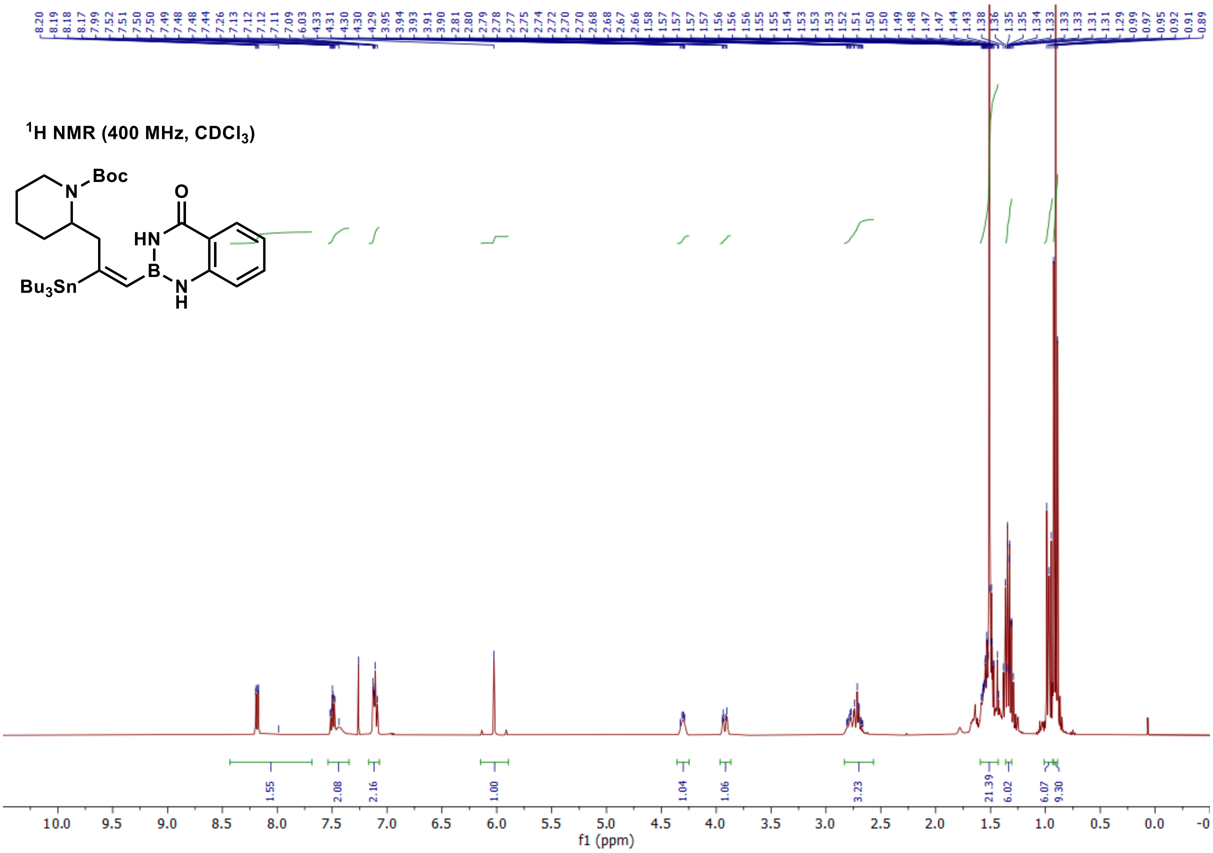
^{1}H NMR (400 MHz, CDCl$_3$)
Boc
N
O
HN
B
N
H
Bu$_3$Sn
f1 (ppm)

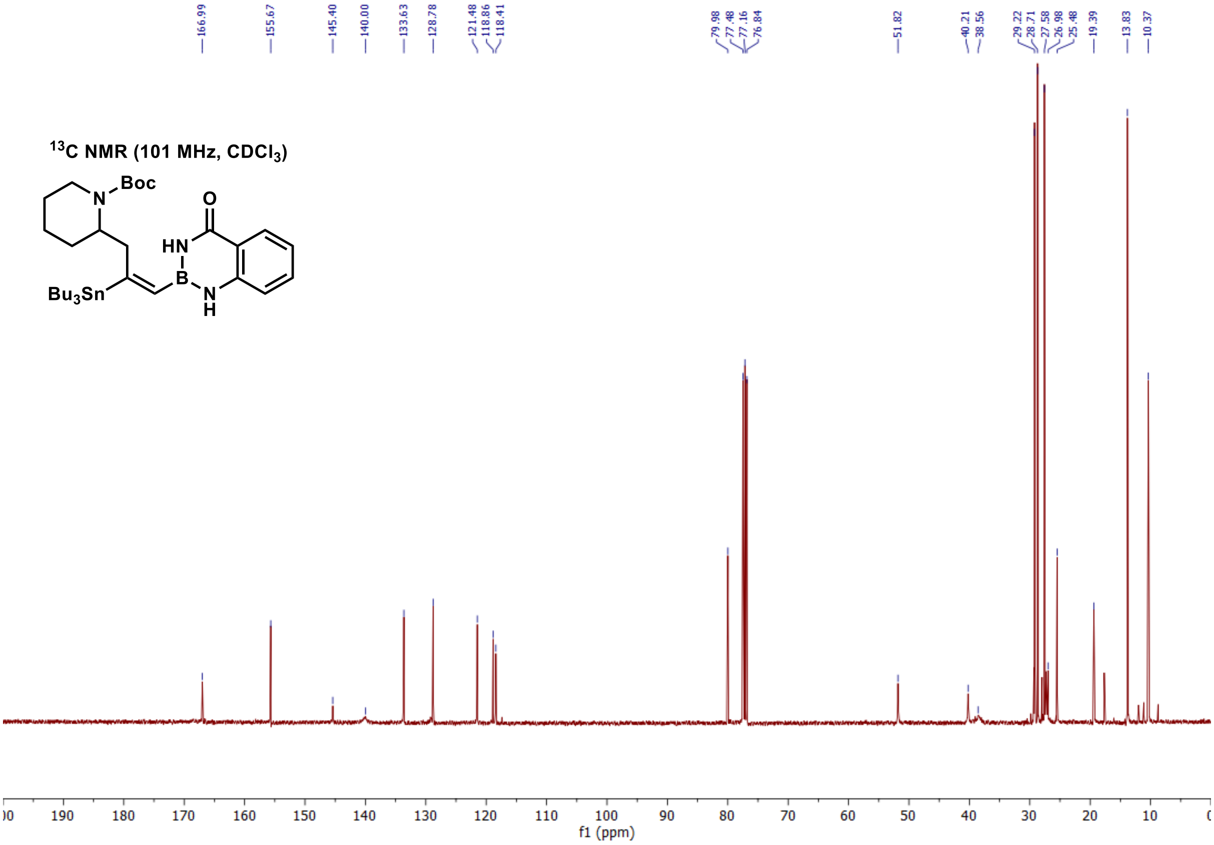
^{13}C NMR (101 MHz, CDCl$_3$)
Boc
N
O
HN
B
N
H
Bu$_3$Sn
f1 (ppm)

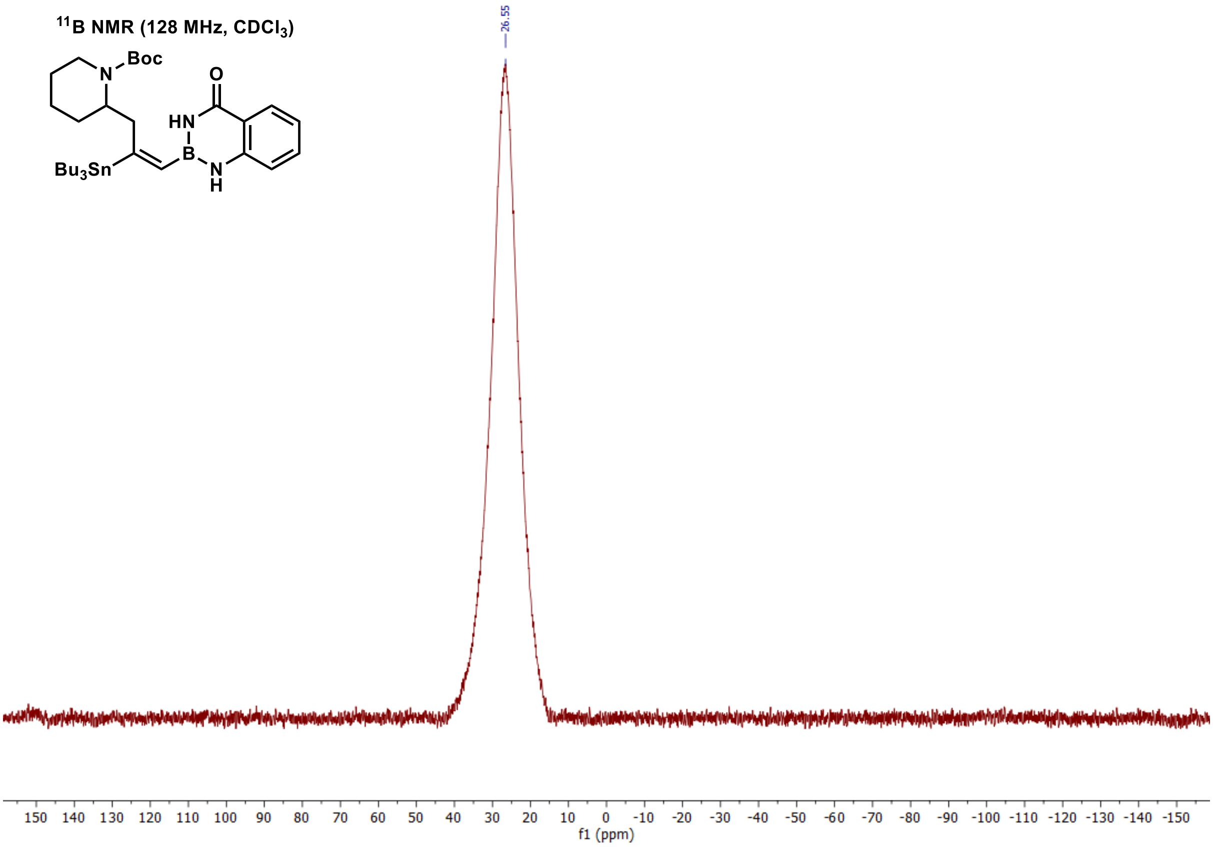

^{11}B NMR (128 MHz, $CDCl_3$)

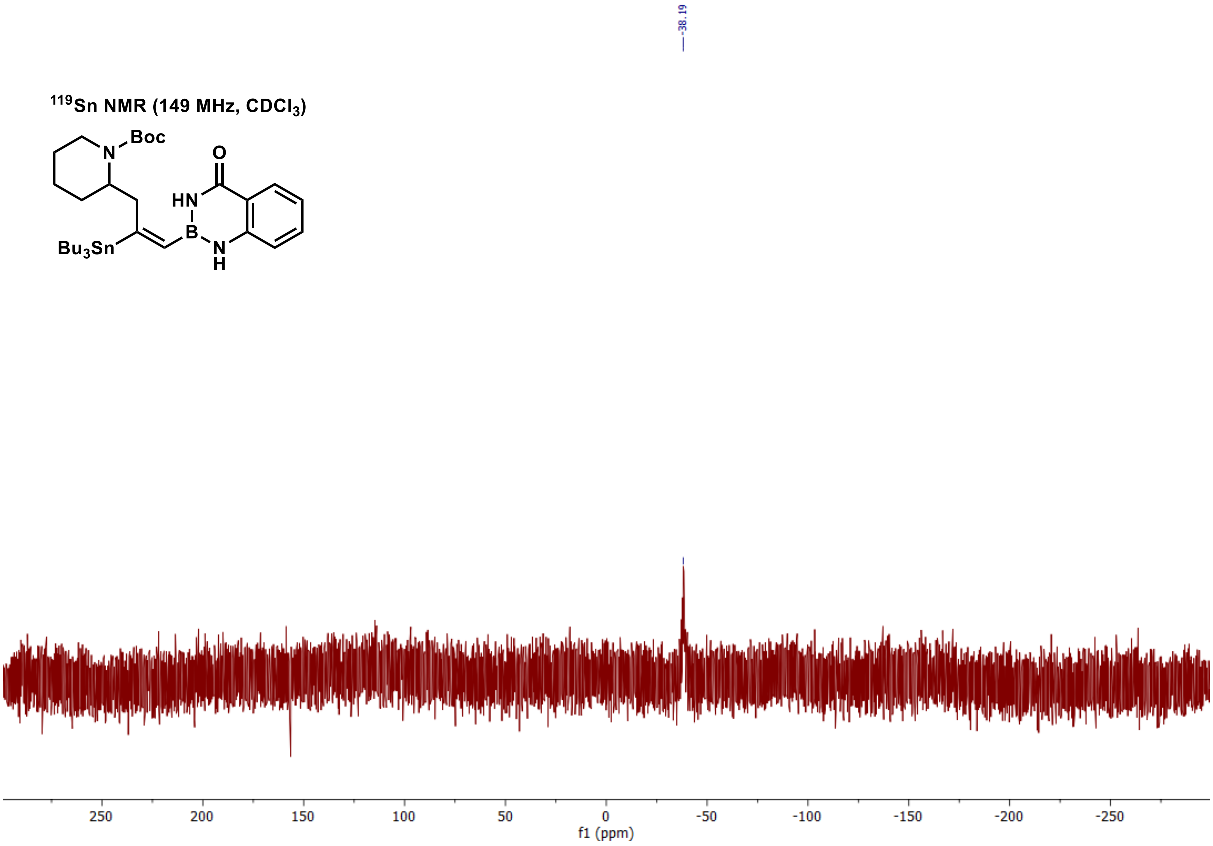

^{119}Sn NMR (149 MHz, $CDCl_3$)

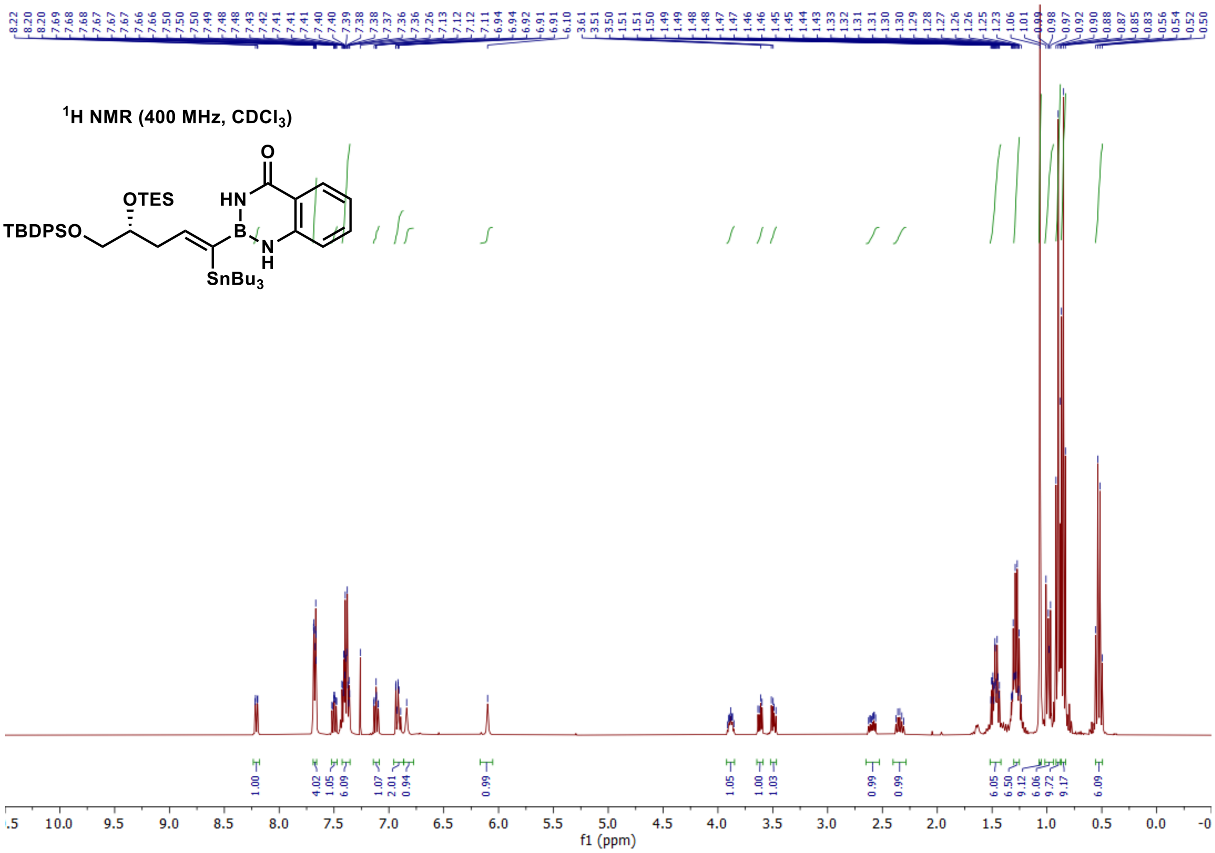
1H NMR (400 MHz, CDCl3)
OTES
TBDPSO
HN
B
N
H
O
SnBu3
f1 (ppm)

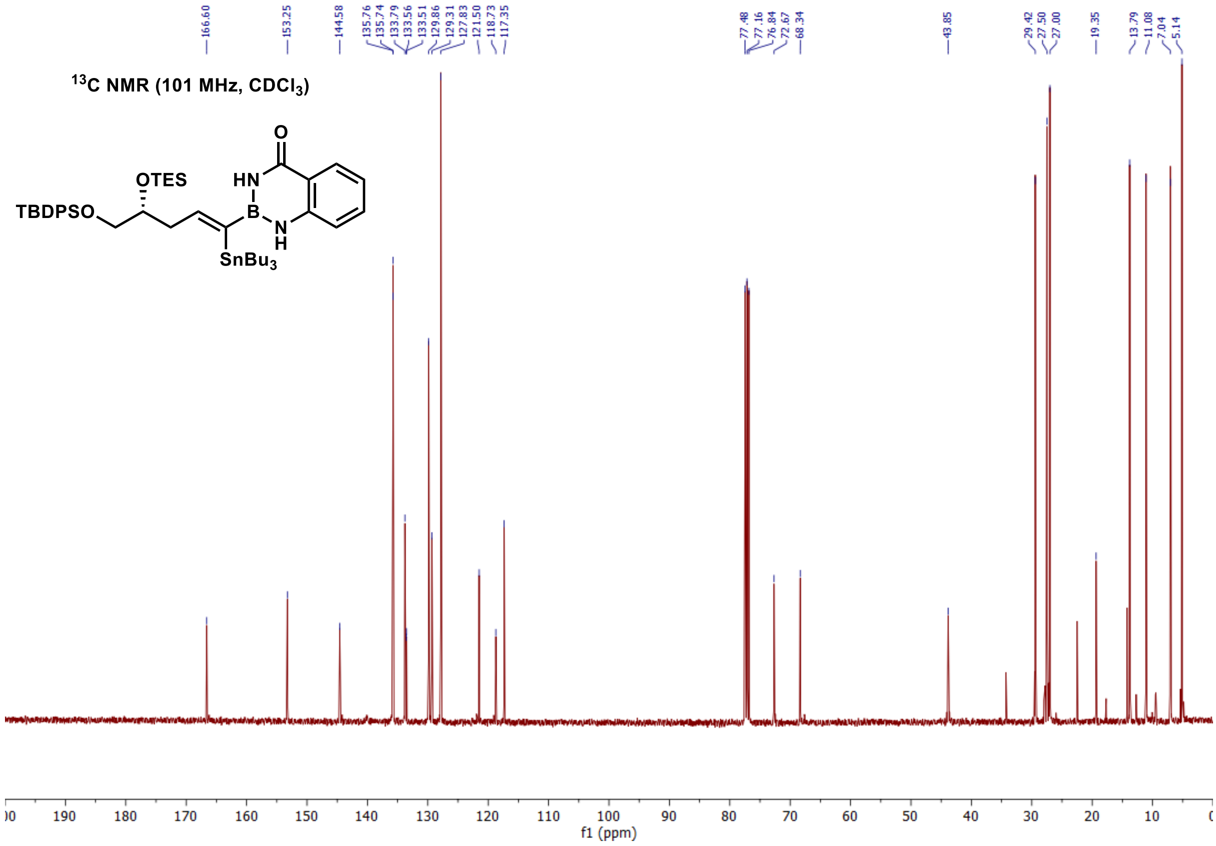
13C NMR (101 MHz, CDCl3)
OTES
TBDPSO
HN
B
N
H
O
SnBu3
166.60
153.25
144.58
135.76
135.74
133.79
133.56
133.51
129.86
129.31
127.83
121.50
118.73
117.35
77.48
77.16
76.84
72.67
68.34
43.85
29.42
27.50
27.00
19.35
13.79
11.08
7.04
5.14
f1 (ppm)

^{11}B NMR (128 MHz, $CDCl_3$)

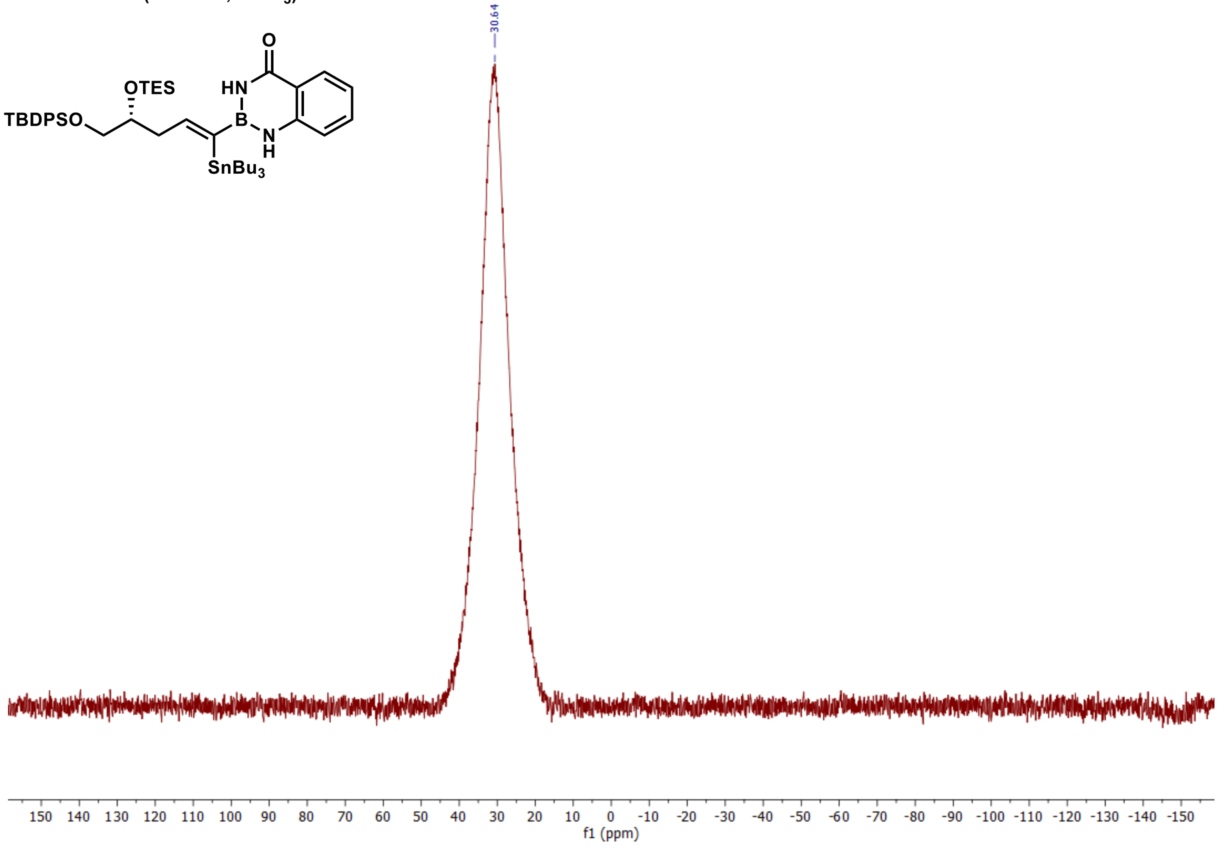

^{119}Sn NMR (149 MHz, $CDCl_3$)

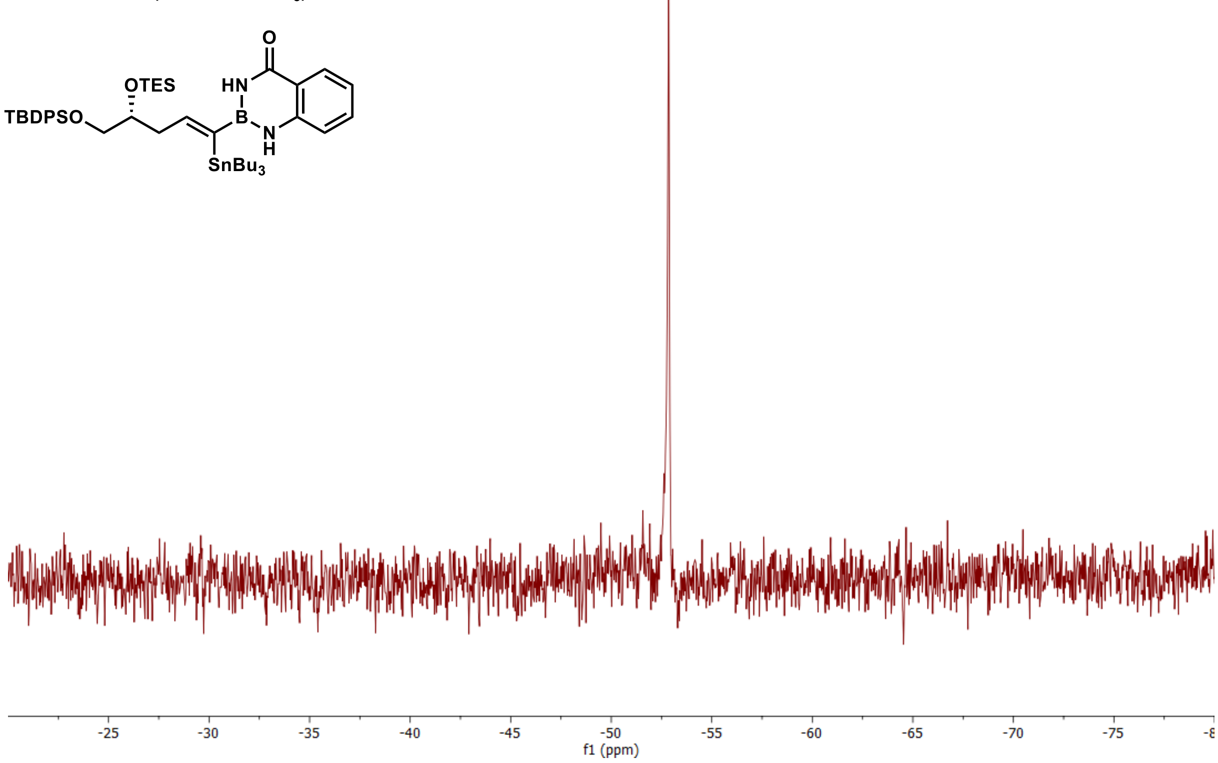

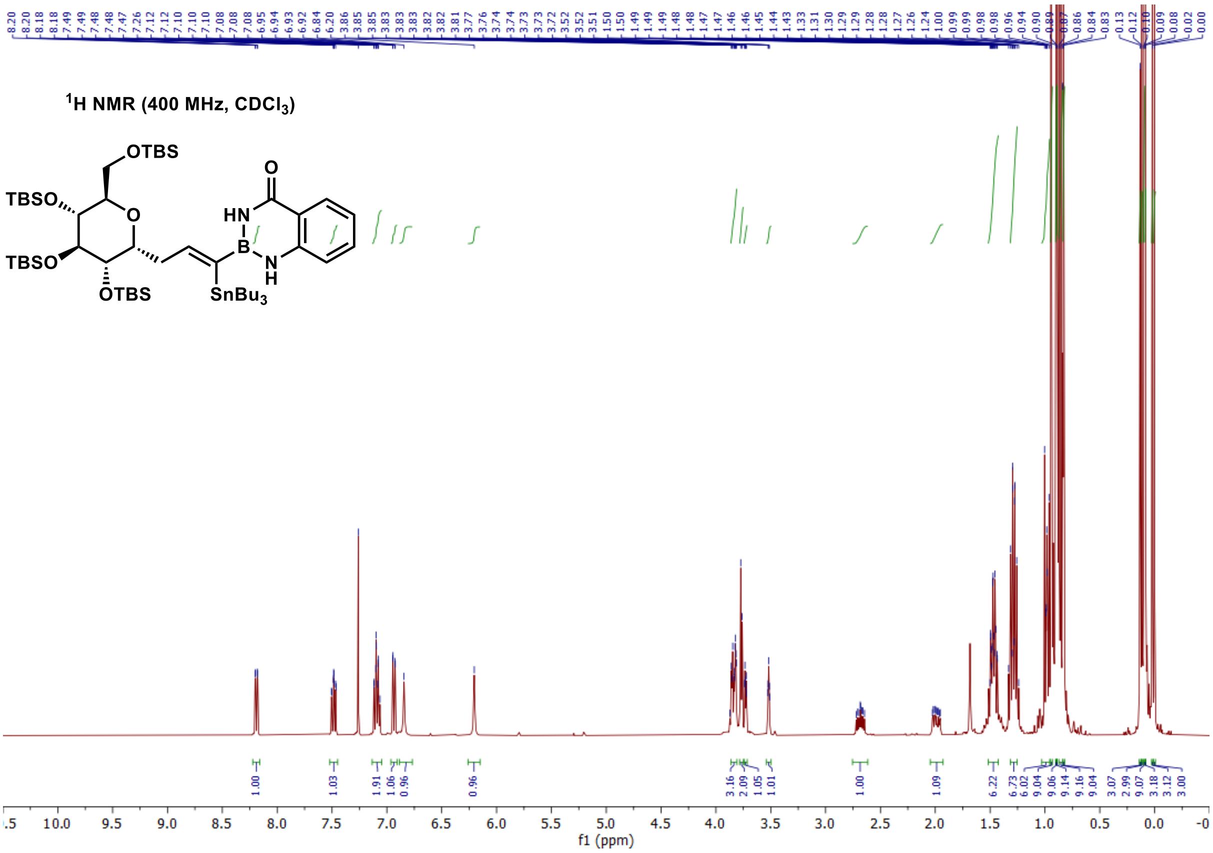
1H NMR (400 MHz, CDCl3)
OTBS
TBSO
TBSO
OTBS
O
HN
B
N
H
SnBu3
f1 (ppm)

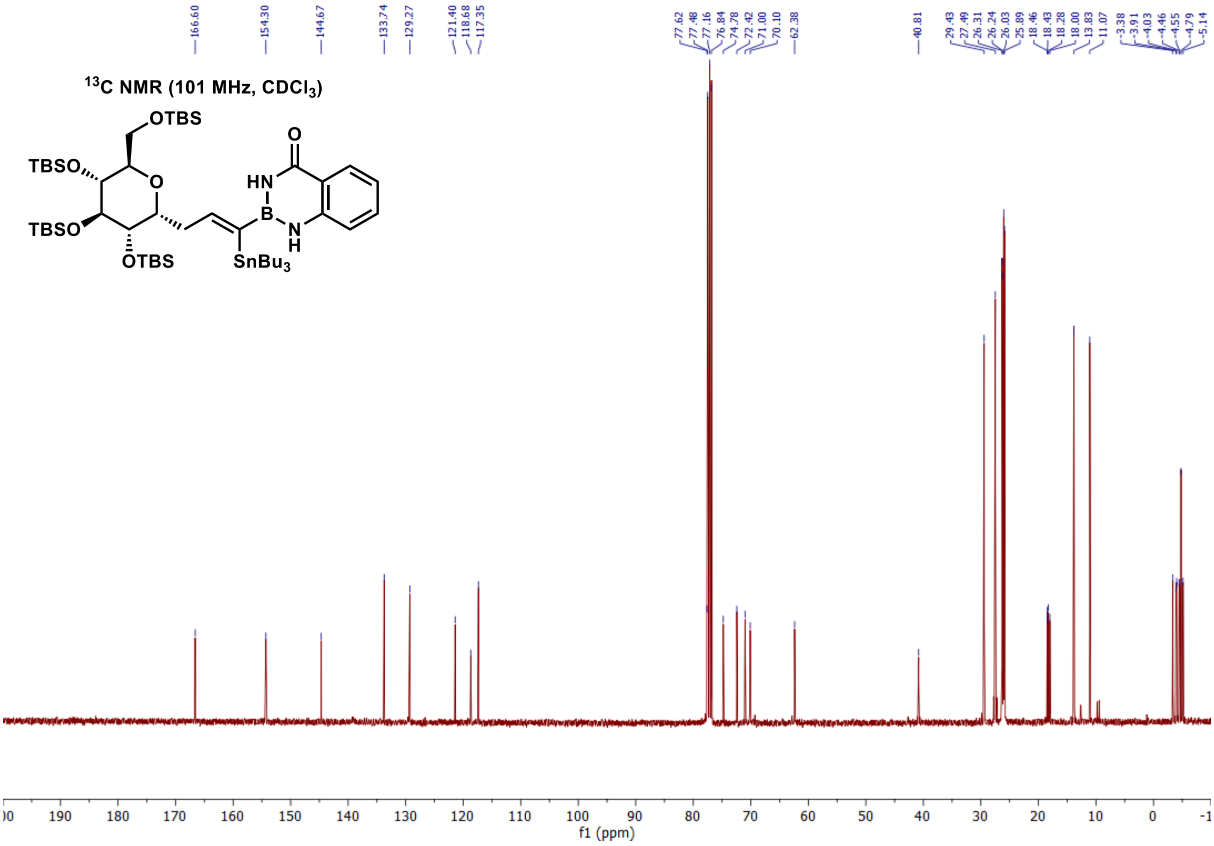
13C NMR (101 MHz, CDCl3)
OTBS
TBSO
TBSO
OTBS
O
HN
B
N
H
SnBu3
f1 (ppm)

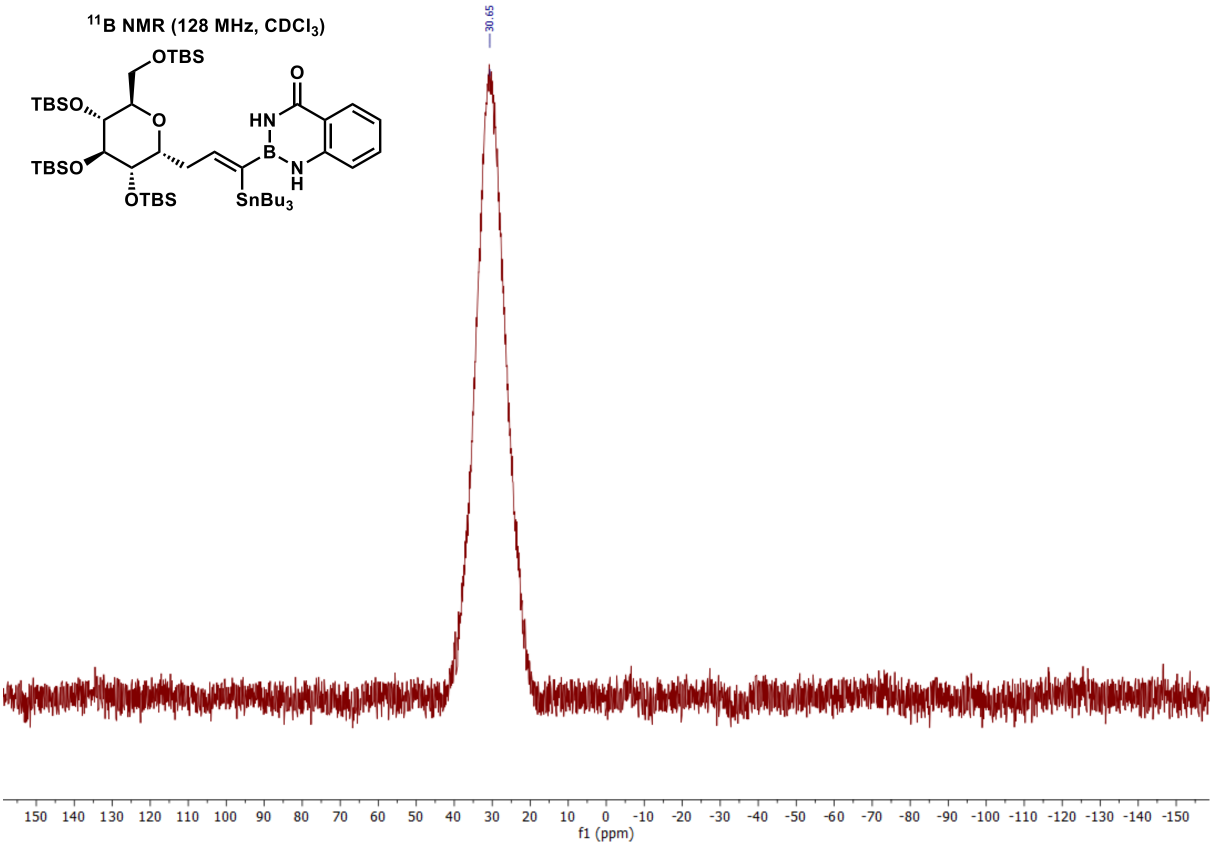

11B NMR (128 MHz, CDCl3)
OTBS
TBSO
TBSO
OTBS
O
HN
B
N
H
O
SnBu3
30.65
150 140 130 120 110 100 90 80 70 60 50 40 30 20 10 0 -10 -20 -30 -40 -50 -60 -70 -80 -90 -100 -110 -120 -130 -140 -150
f1 (ppm)

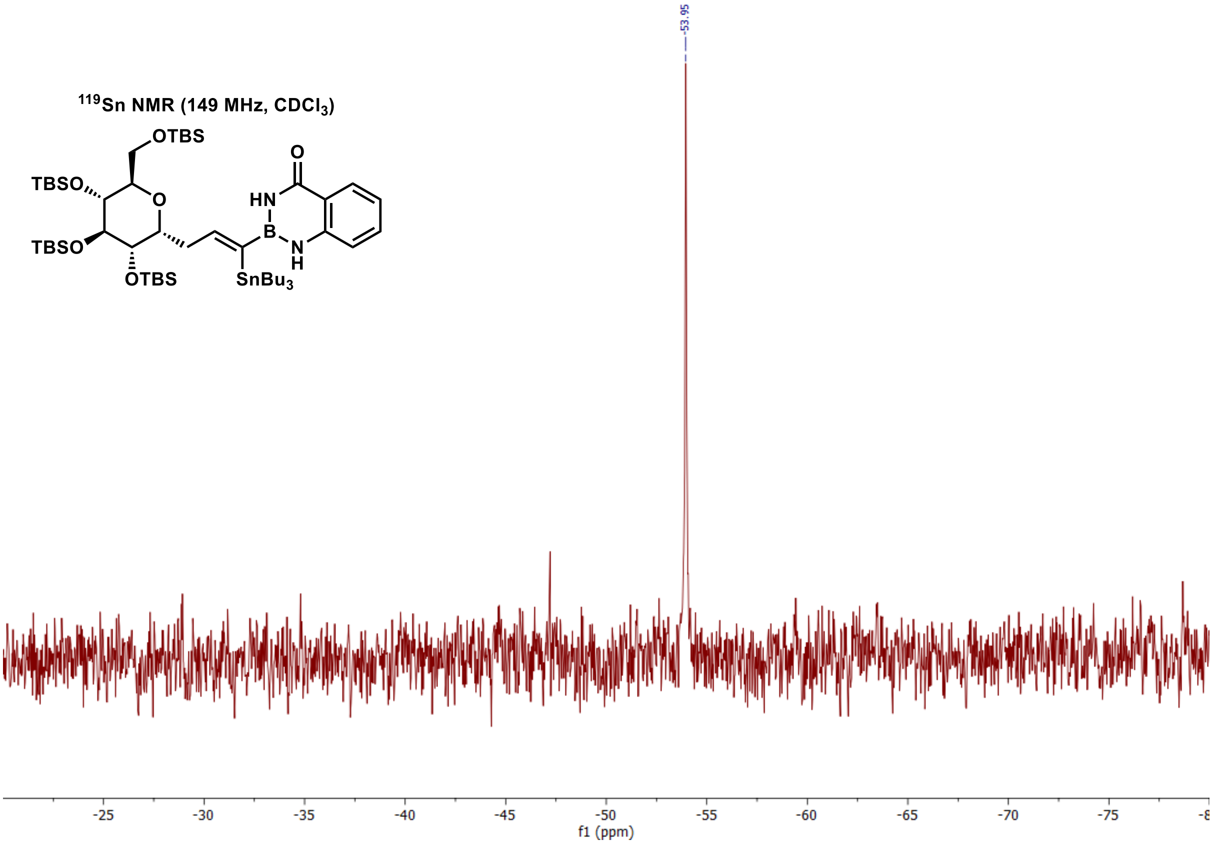

119Sn NMR (149 MHz, CDCl3)
OTBS
TBSO
TBSO
OTBS
O
HN
B
N
H
O
SnBu3
-53.95
-25 -30 -35 -40 -45 -50 -55 -60 -65 -70 -75
f1 (ppm)

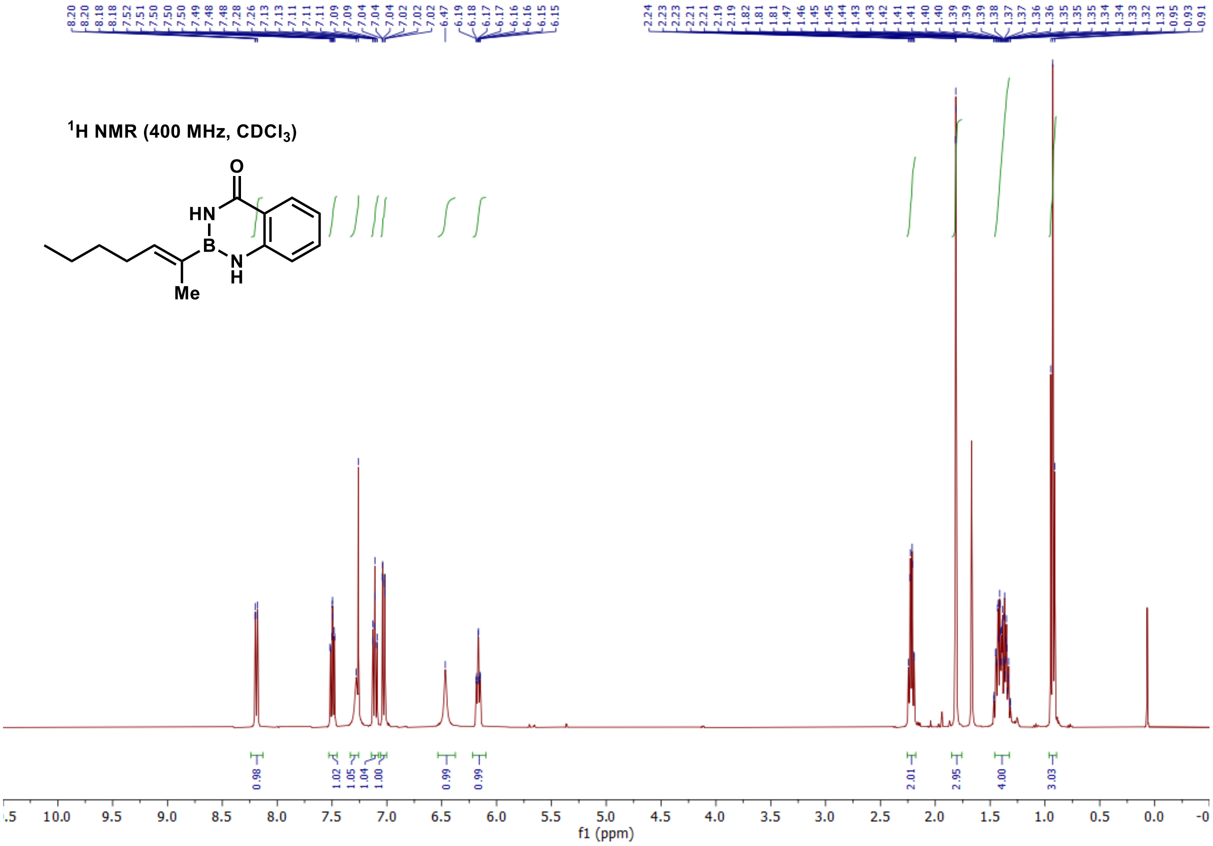
^{1}H NMR (400 MHz, CDCl$_3$)
HN
B
N
H
O
Me
f1 (ppm)

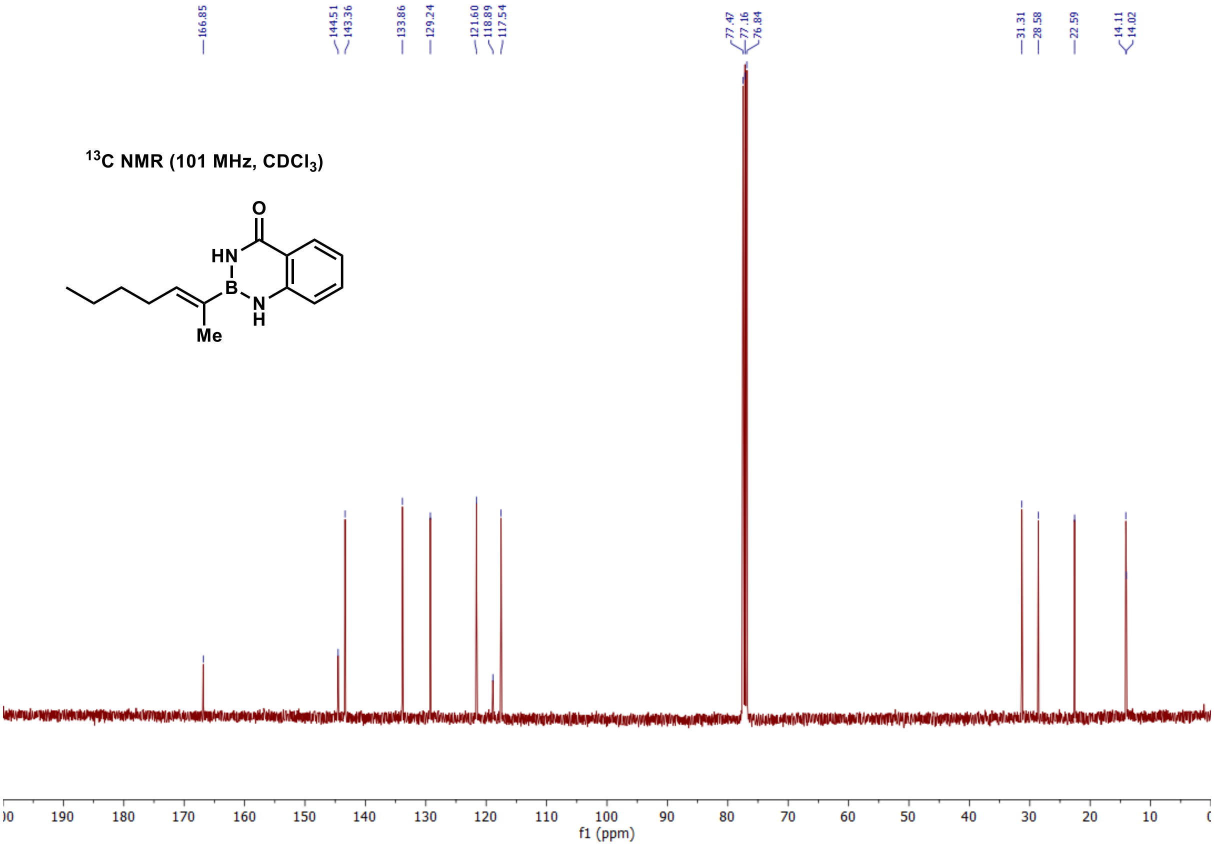
^{13}C NMR (101 MHz, CDCl$_3$)
HN
B
N
H
O
Me
166.85
144.51
143.36
133.86
129.24
121.60
118.89
117.54
77.47
77.16
76.84
31.31
28.58
22.59
14.11
14.02
f1 (ppm)

^{11}B NMR (128 MHz, $CDCl_3$)

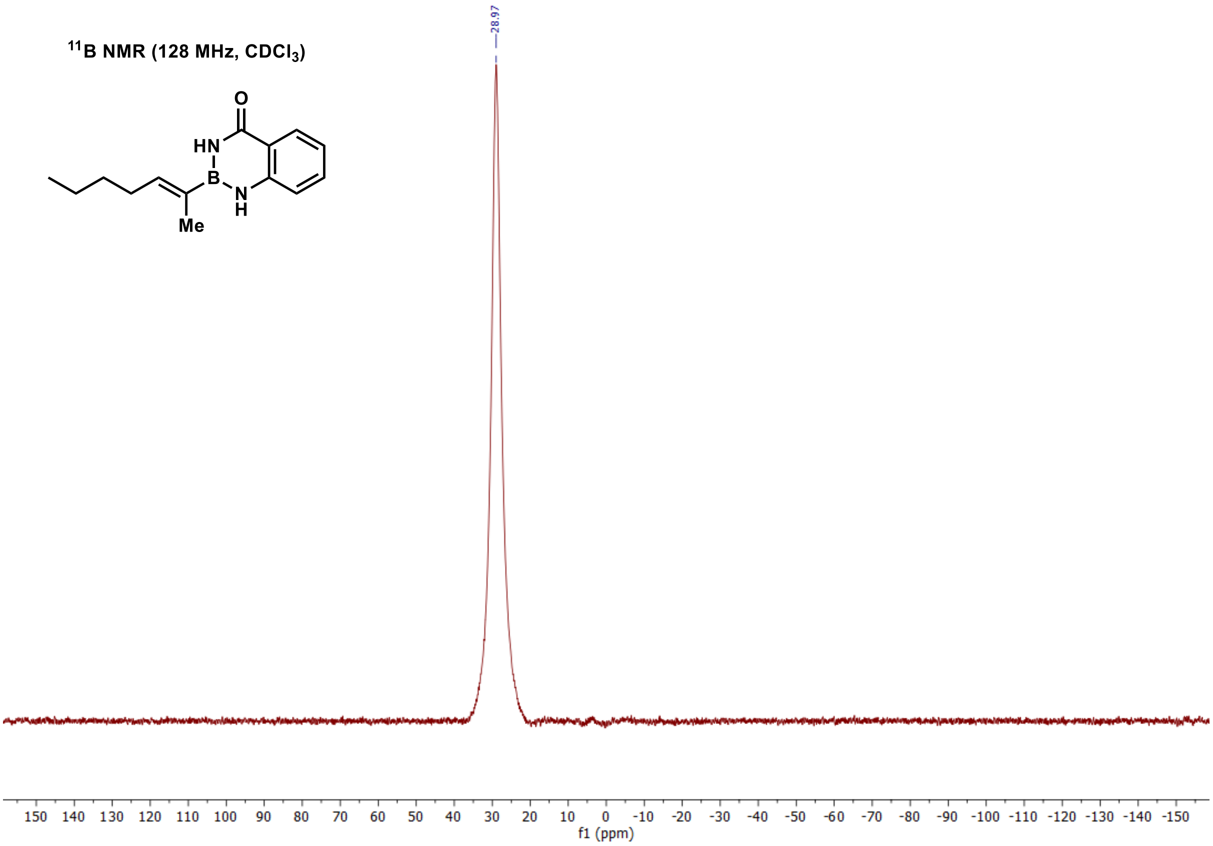

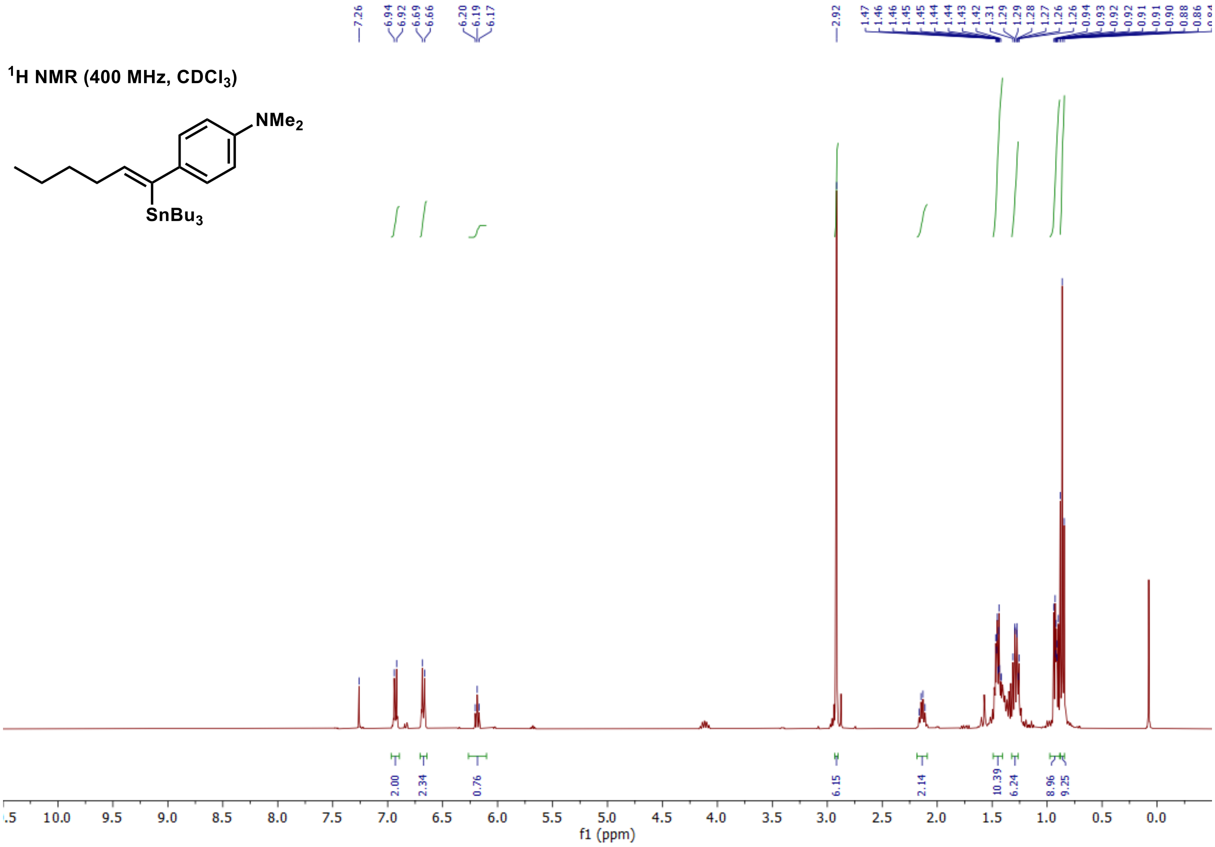

^{1}H NMR (400 MHz, $CDCl_3$)
NMe2
SnBu3
f1 (ppm)

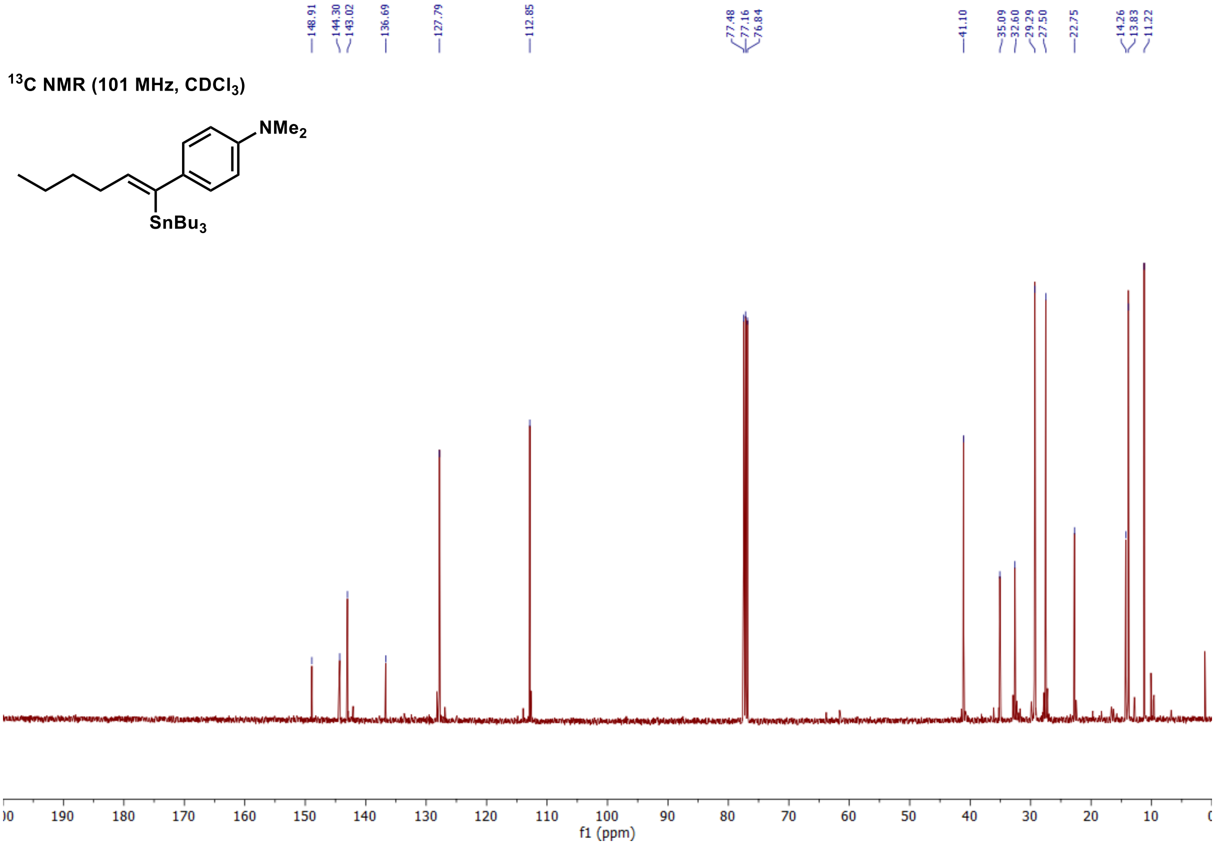

^{13}C NMR (101 MHz, $CDCl_3$)
NMe2
SnBu3
f1 (ppm)

^{119}Sn NMR (149 MHz, $CDCl_3$)

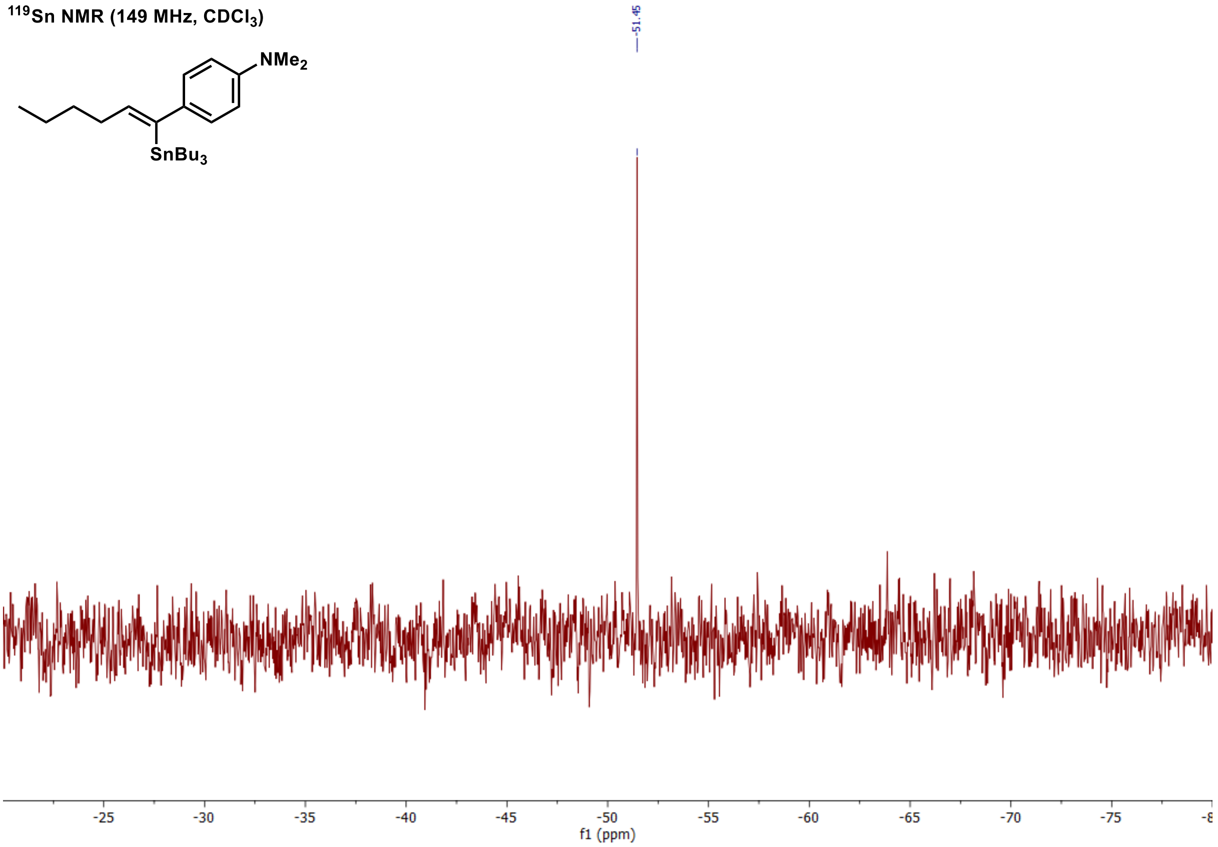

^{1}H NMR (400 MHz, $CDCl_3$)

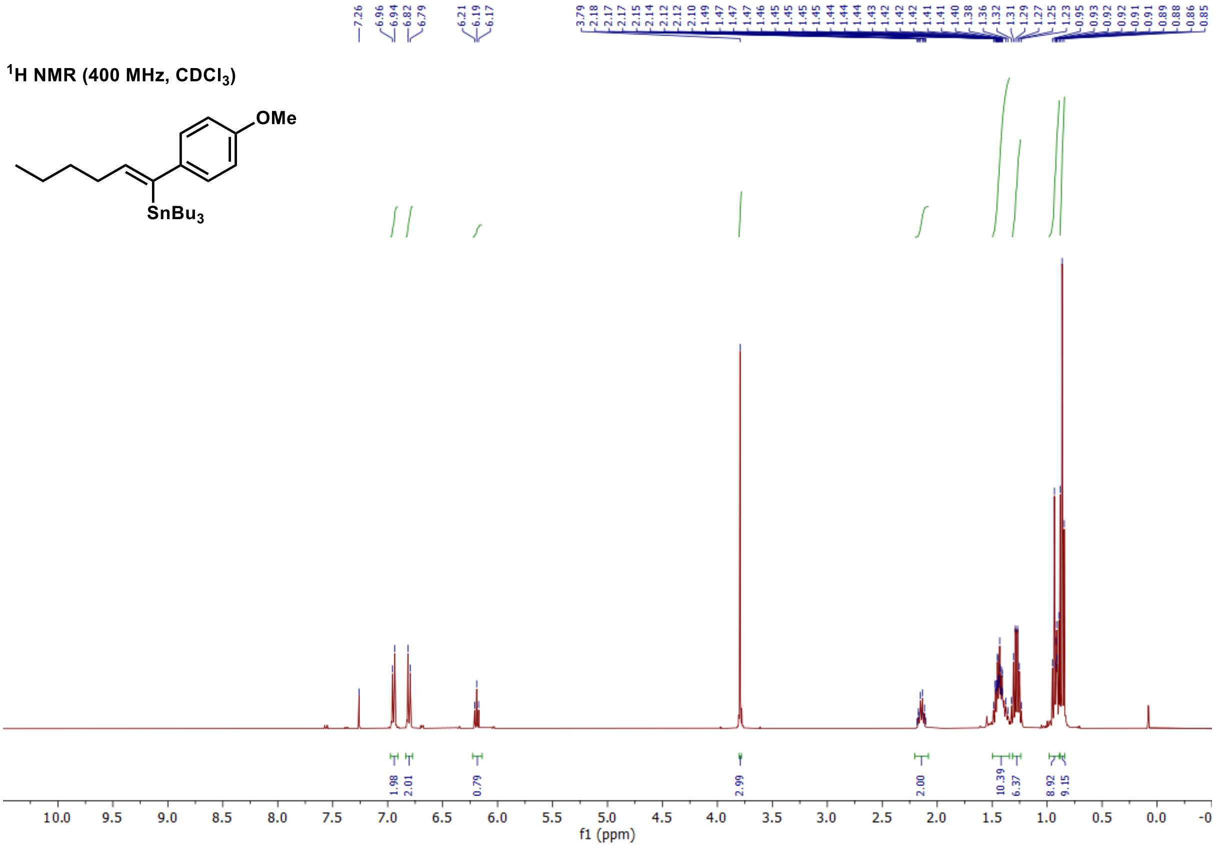

^{13}C NMR (101 MHz, $CDCl_3$)

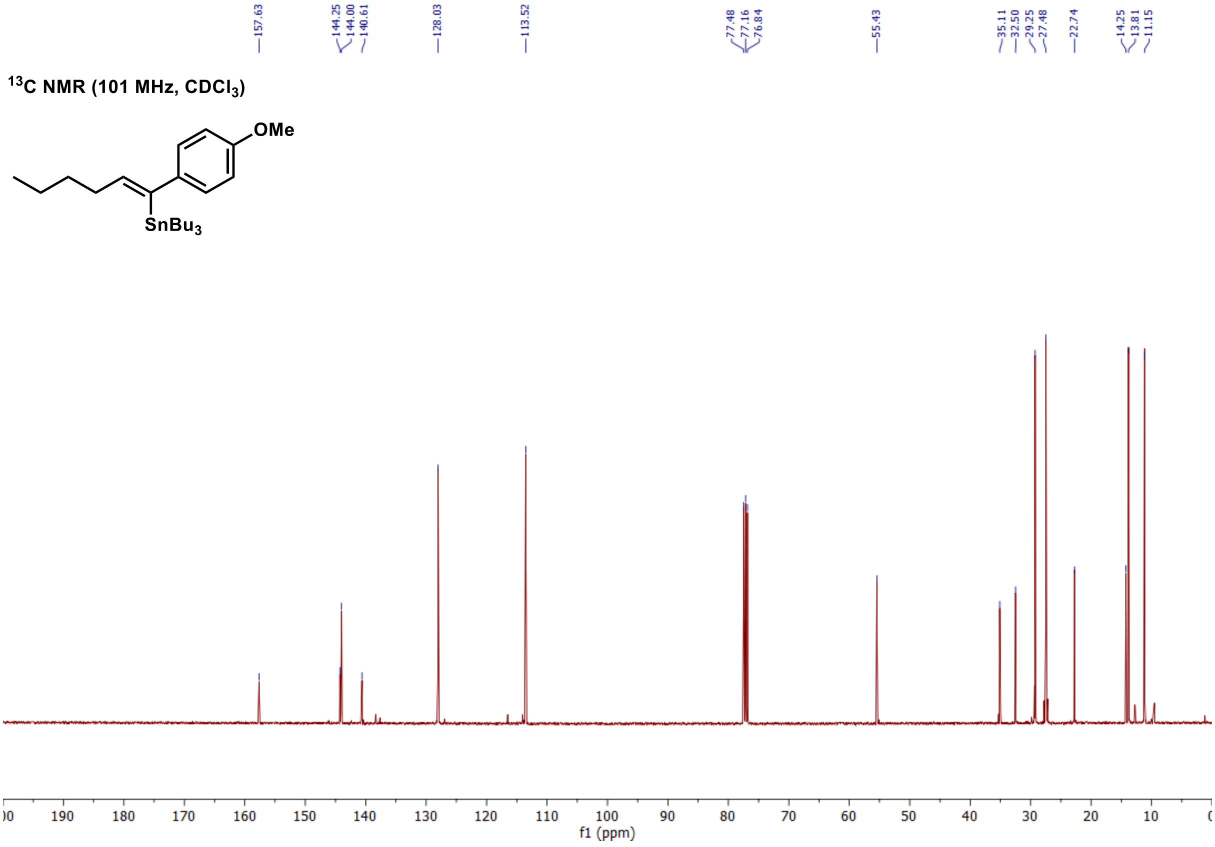

^{119}Sn NMR (149 MHz, $CDCl_3$)

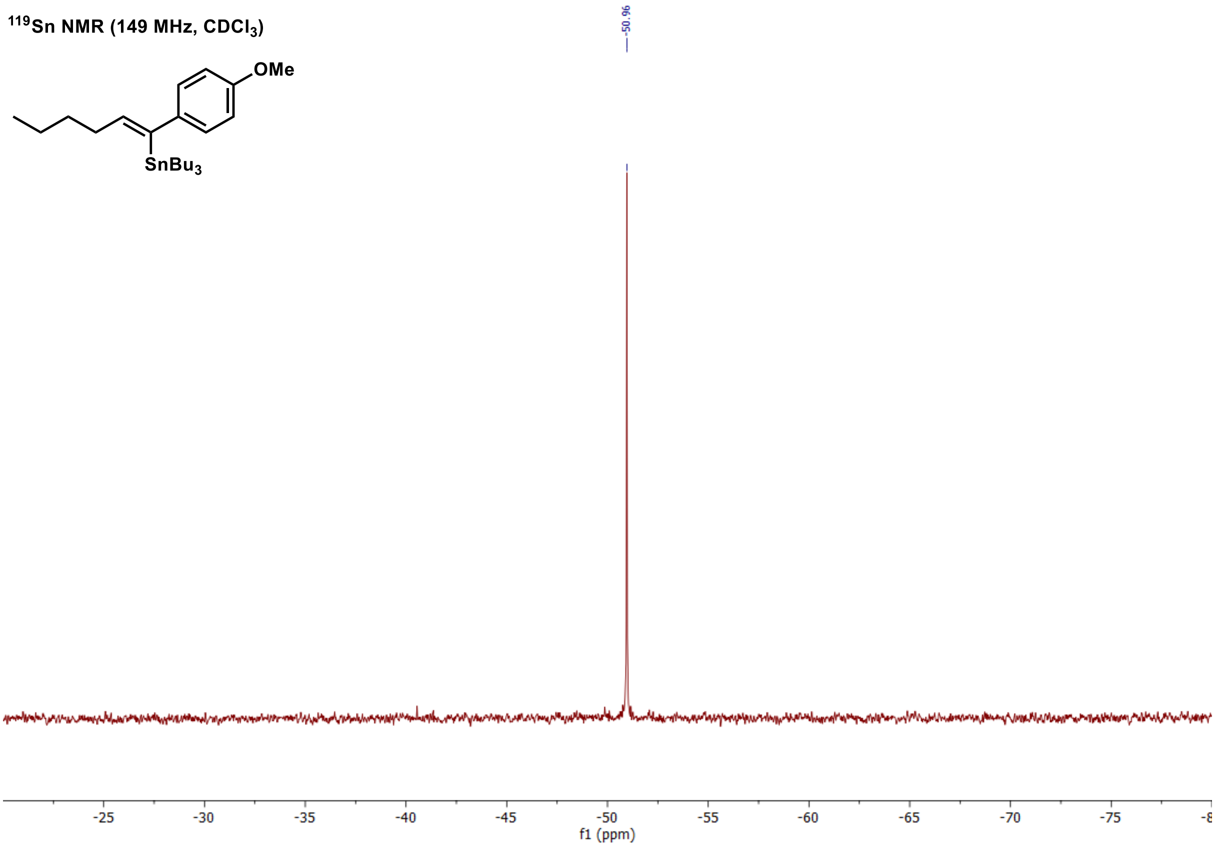

^{1}H NMR (400 MHz, $CDCl_3$)

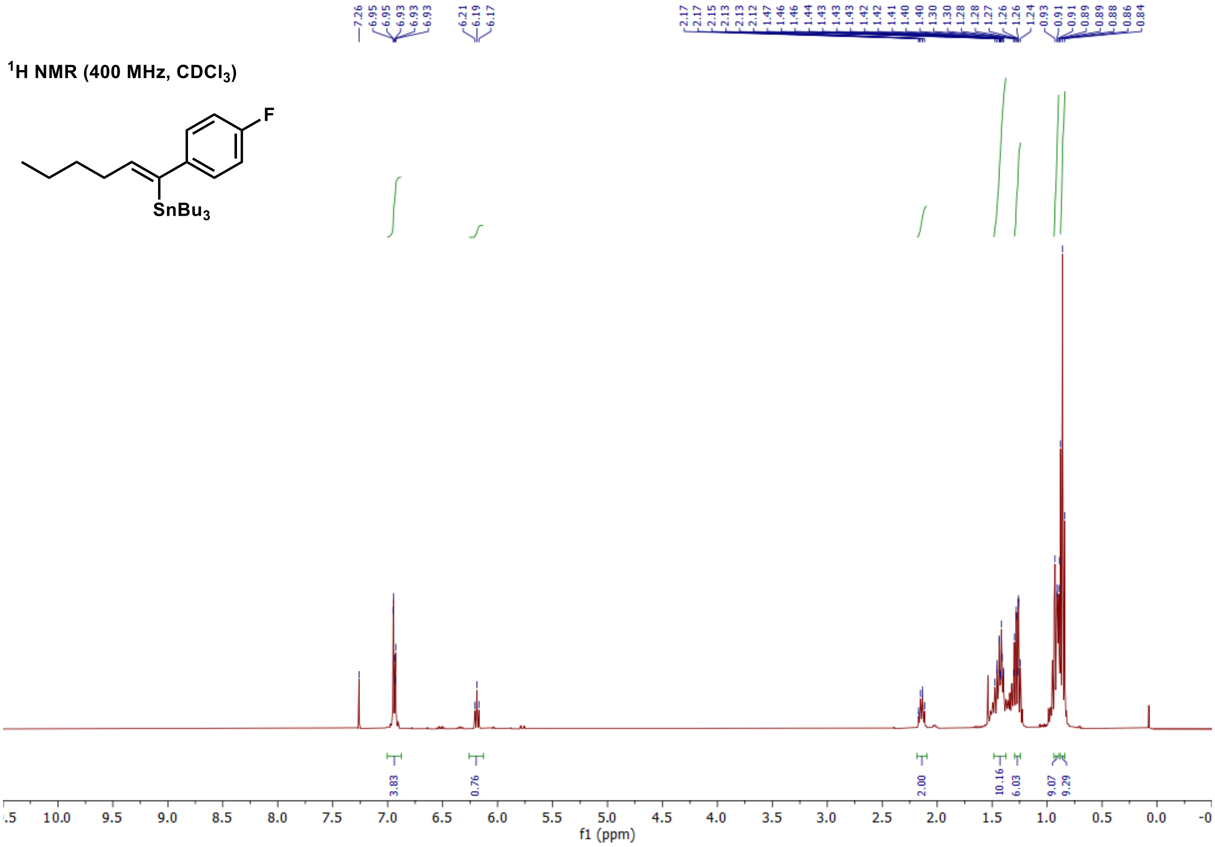

^{13}C NMR (101 MHz, $CDCl_3$)

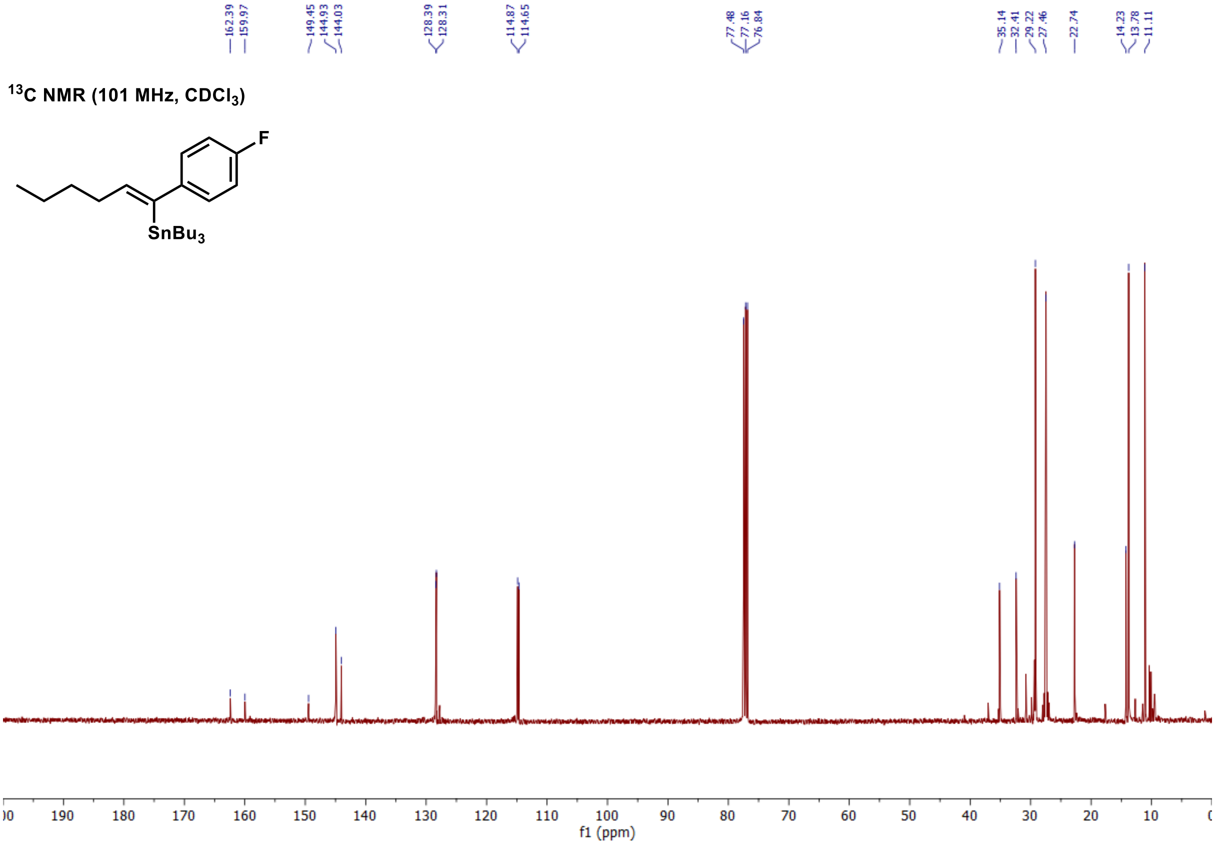

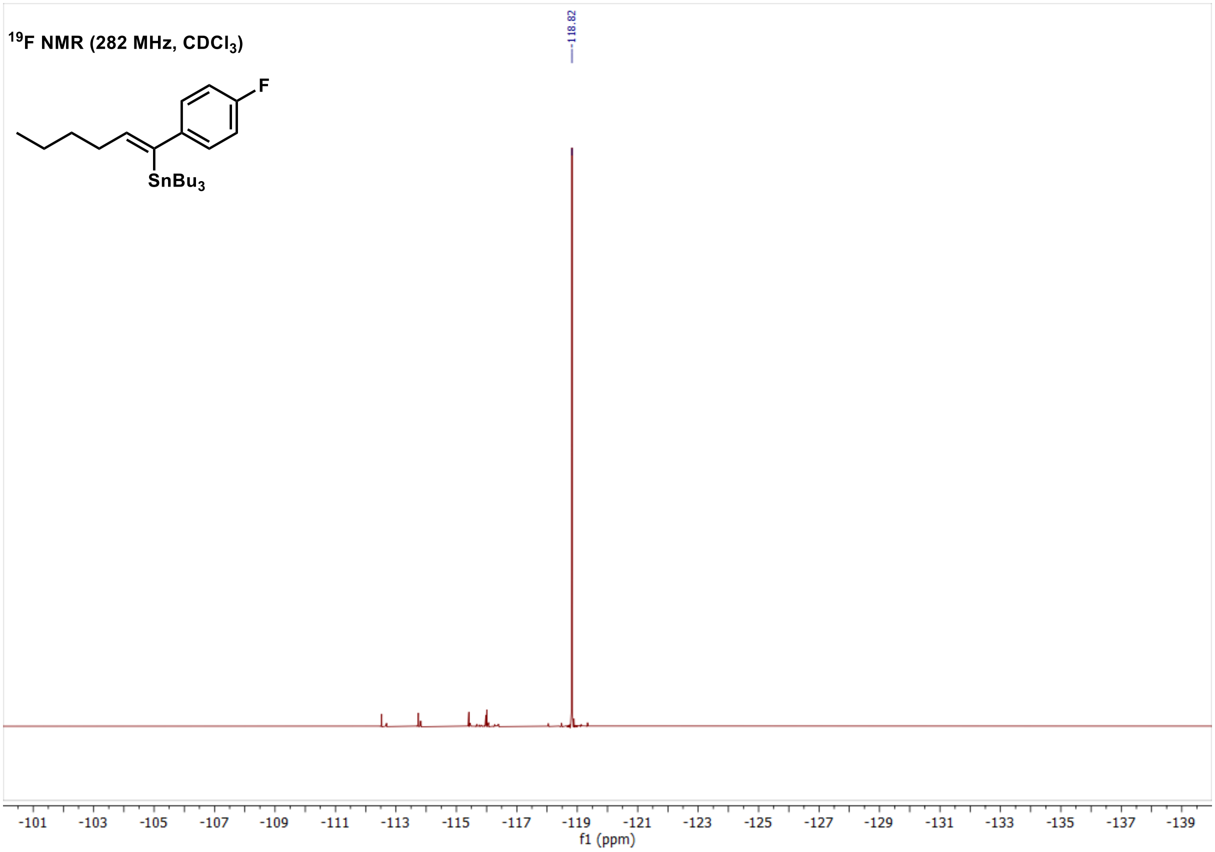

19F NMR (282 MHz, CDCl3)
F
SnBu3
-118.82
-101 -103 -105 -107 -109 -111 -113 -115 -117 -119 -121 -123 -125 -127 -129 -131 -133 -135 -137 -139
f1 (ppm)

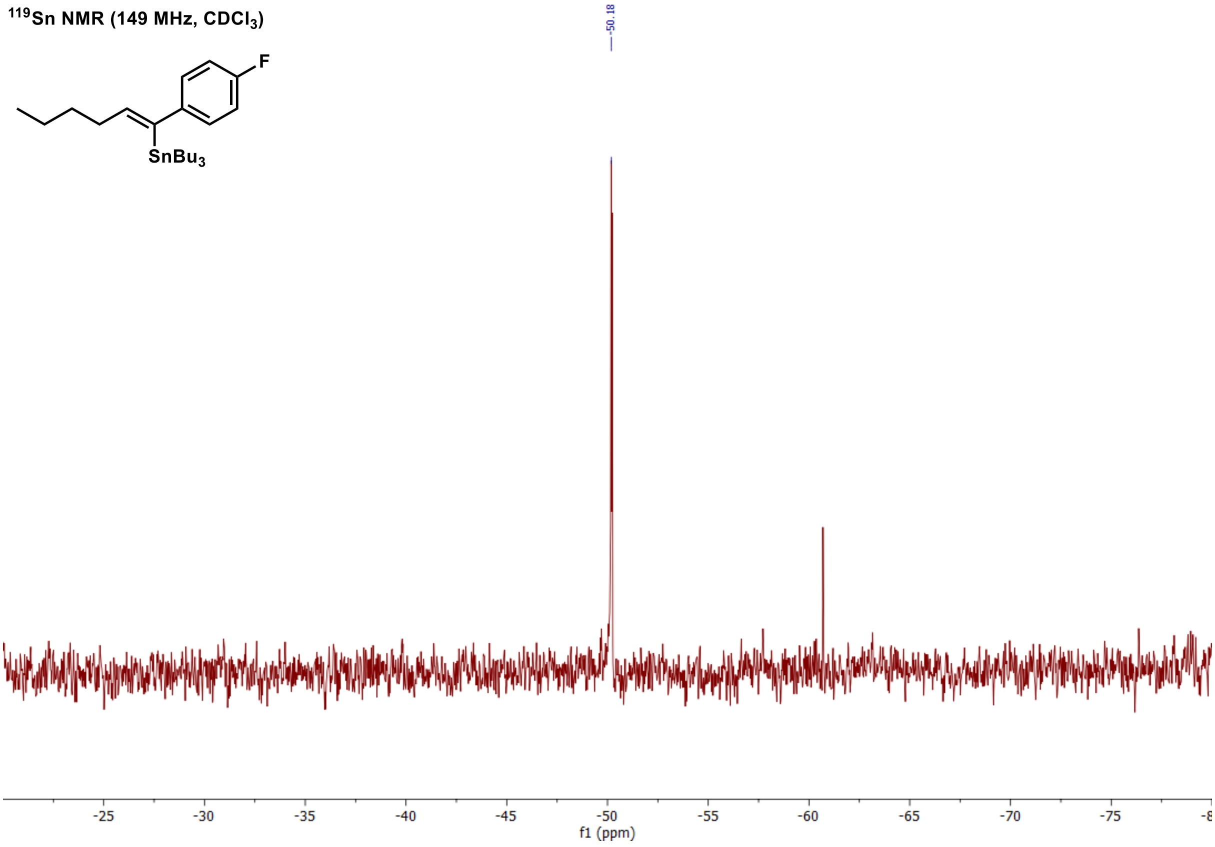

119Sn NMR (149 MHz, CDCl3)
F
SnBu3
-50.18
-25 -30 -35 -40 -45 -50 -55 -60 -65 -70 -75
f1 (ppm)

^{1}H NMR (400 MHz, $CDCl_3$)

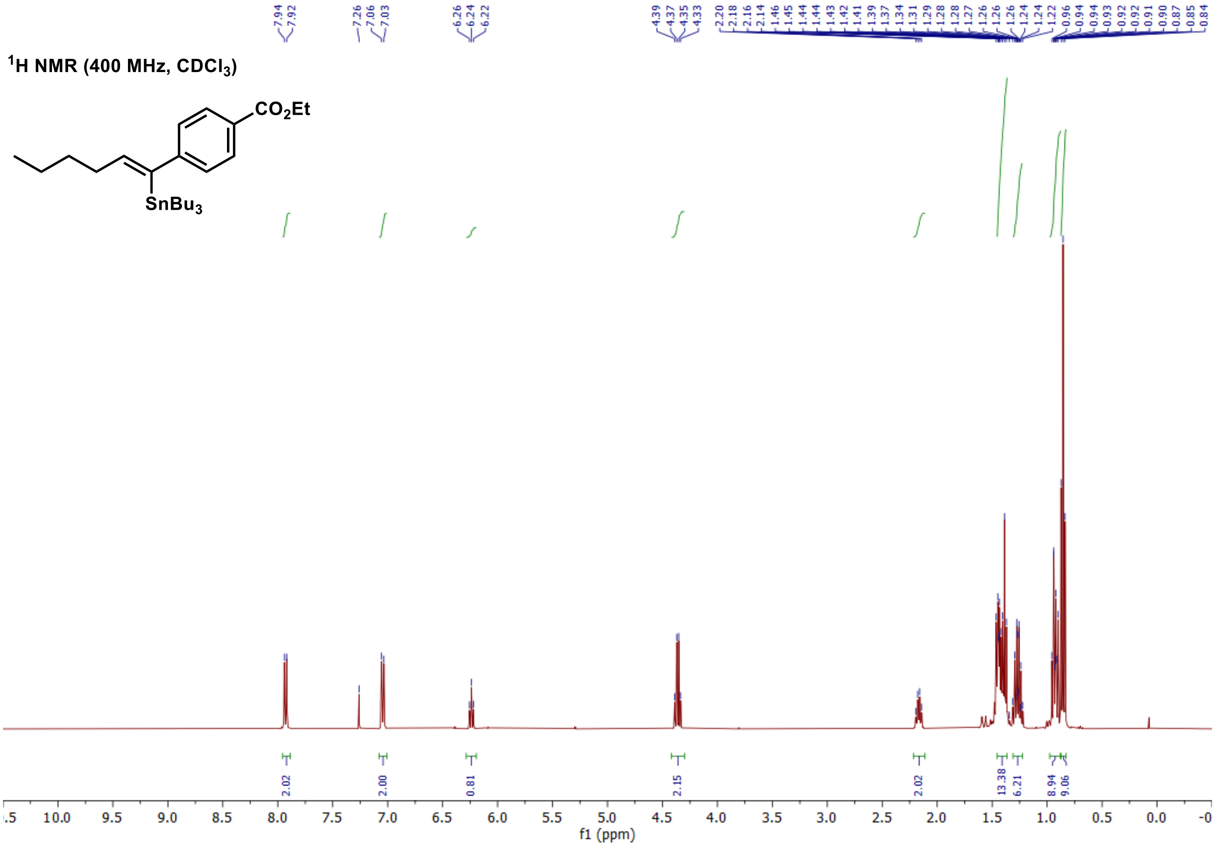

^{13}C NMR (101 MHz, $CDCl_3$)

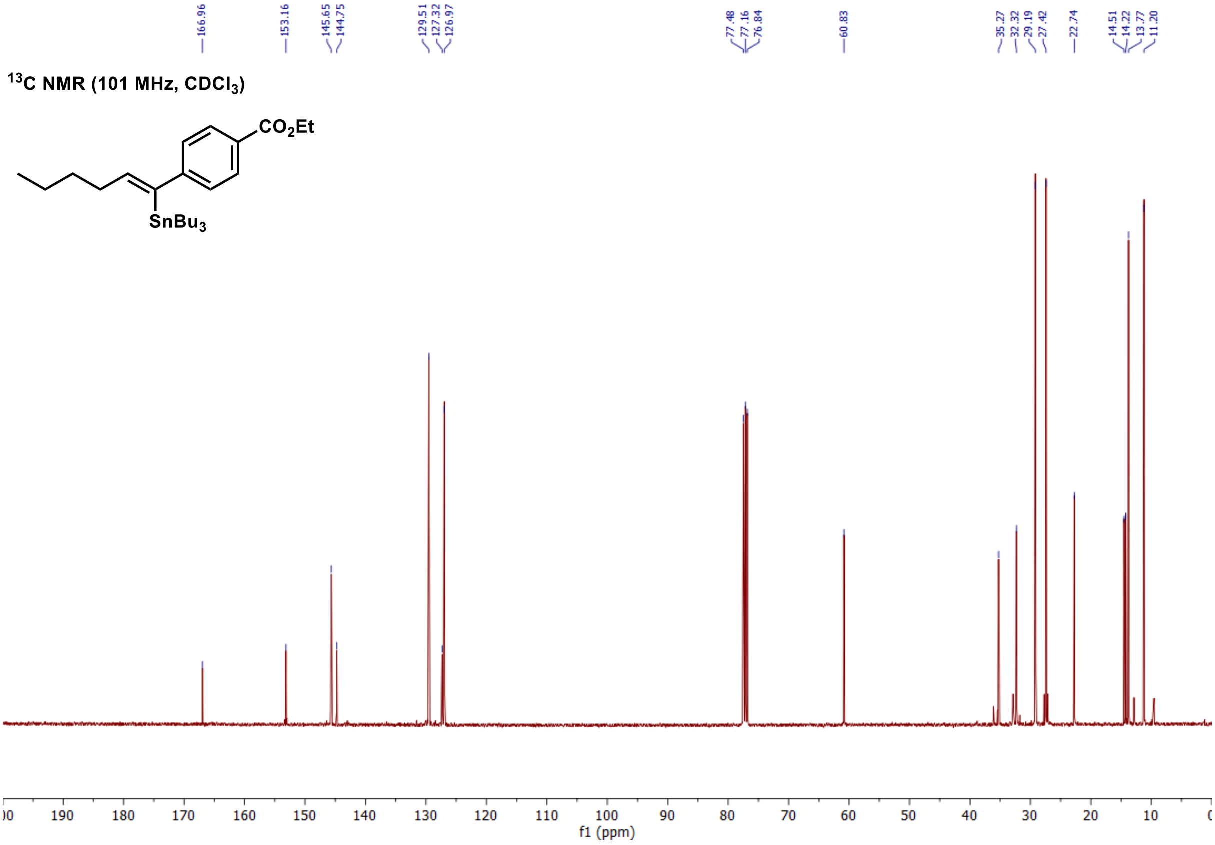

^{119}Sn NMR (149 MHz, $CDCl_3$)

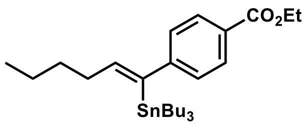

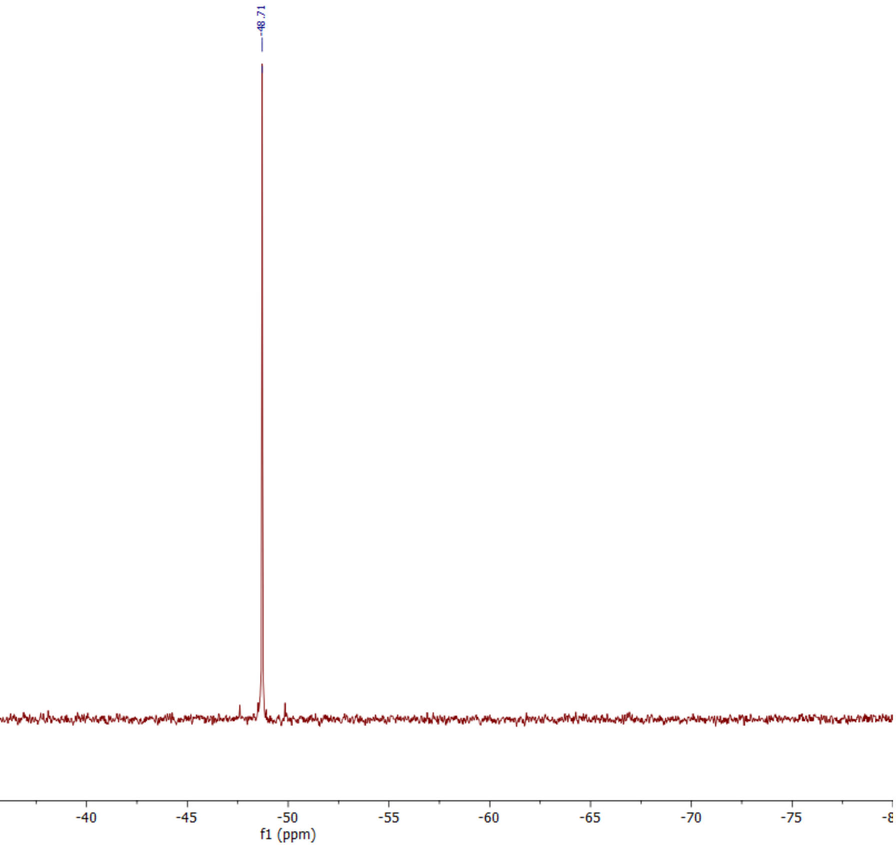

^{1}H NMR (400 MHz, $CDCl_3$)

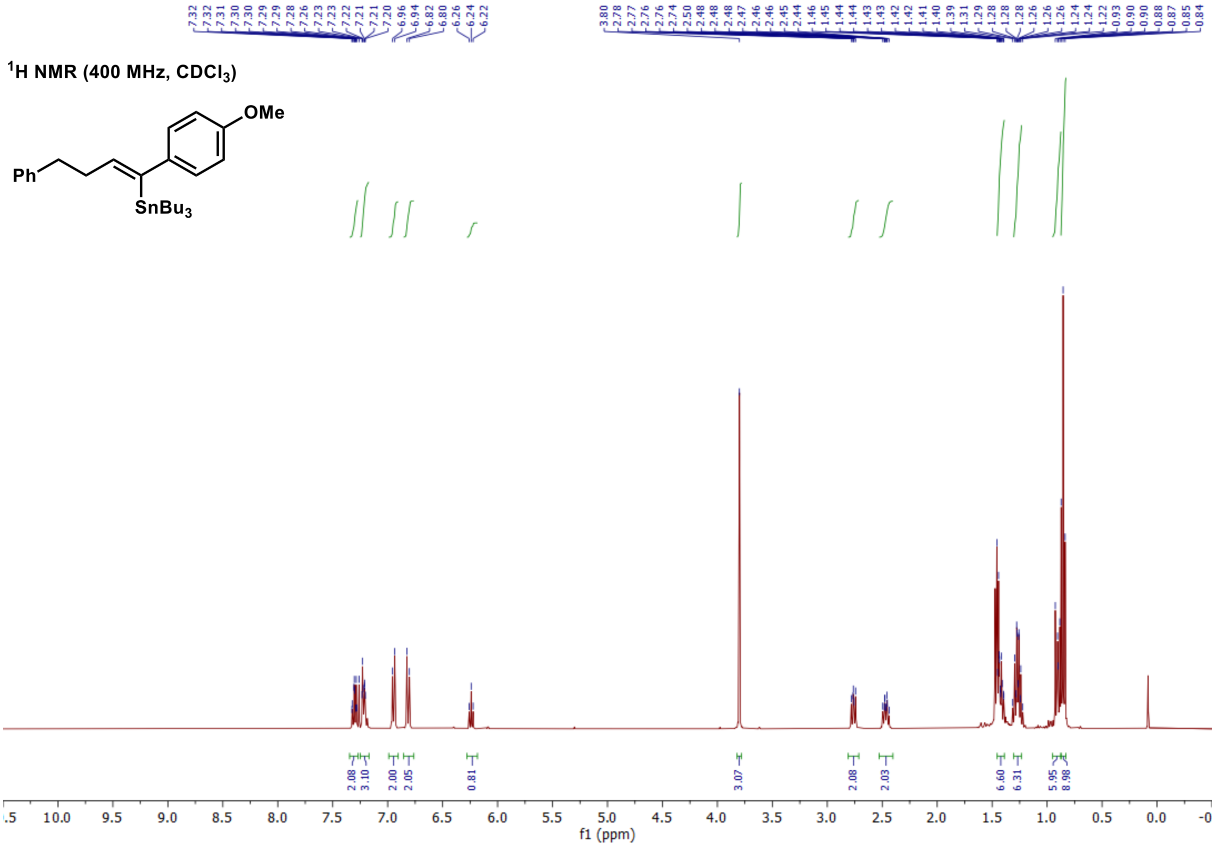

^{13}C NMR (101 MHz, $CDCl_3$)

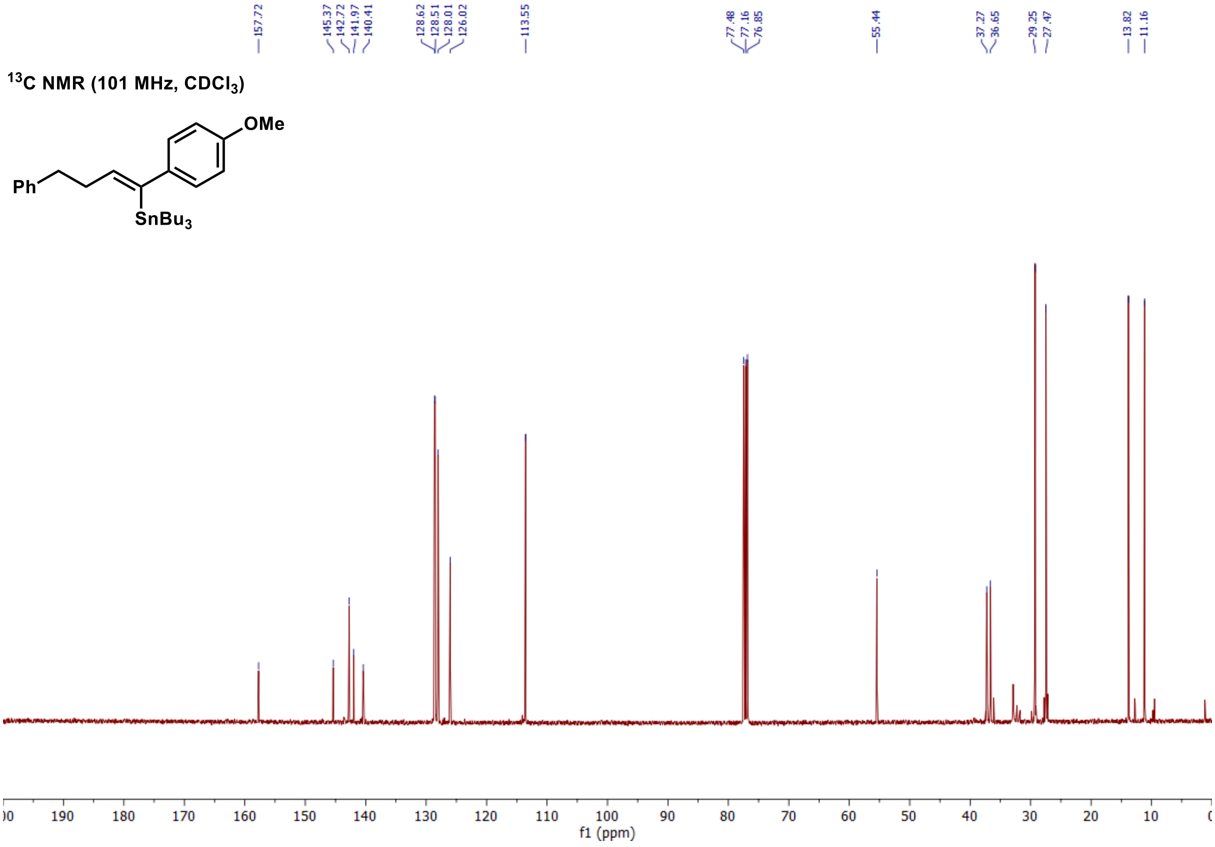

^{119}Sn NMR (149 MHz, $CDCl_3$)

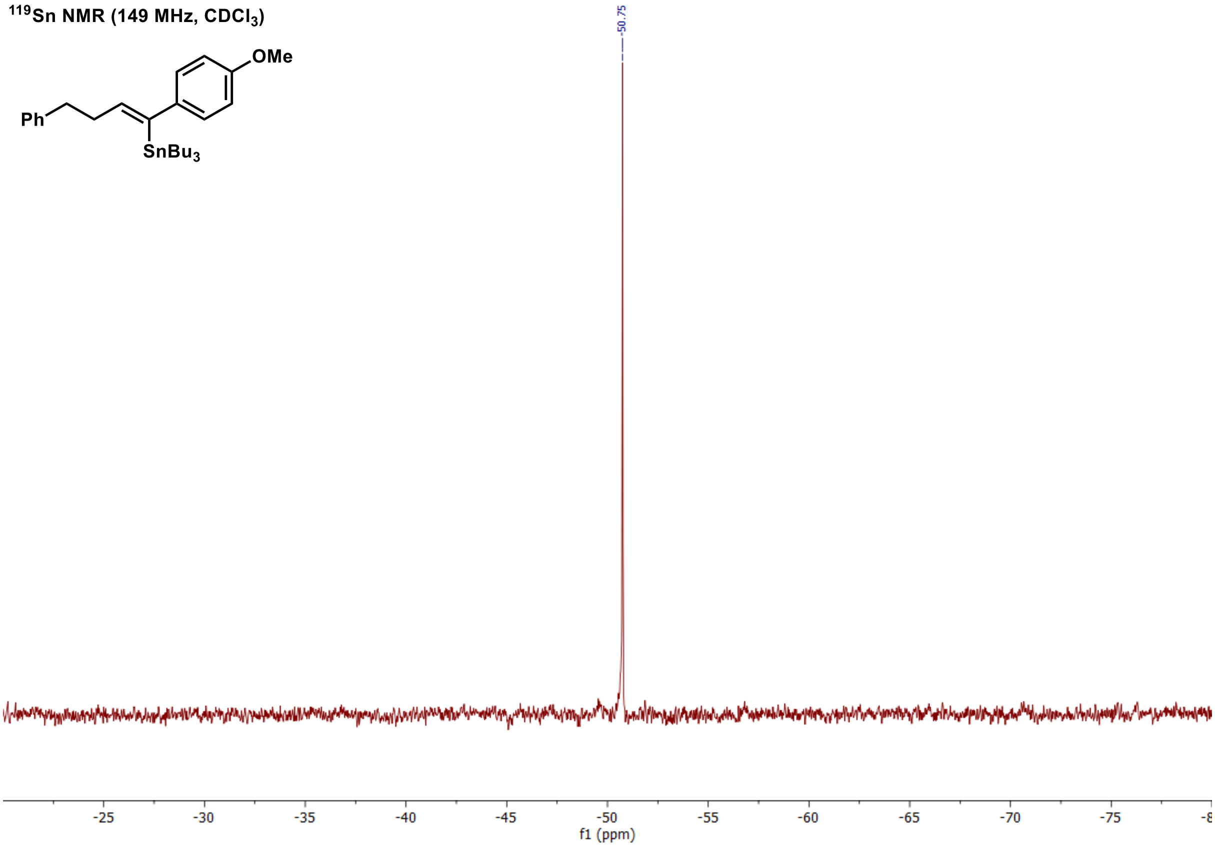

^1H NMR (400 MHz, $CDCl_3$)

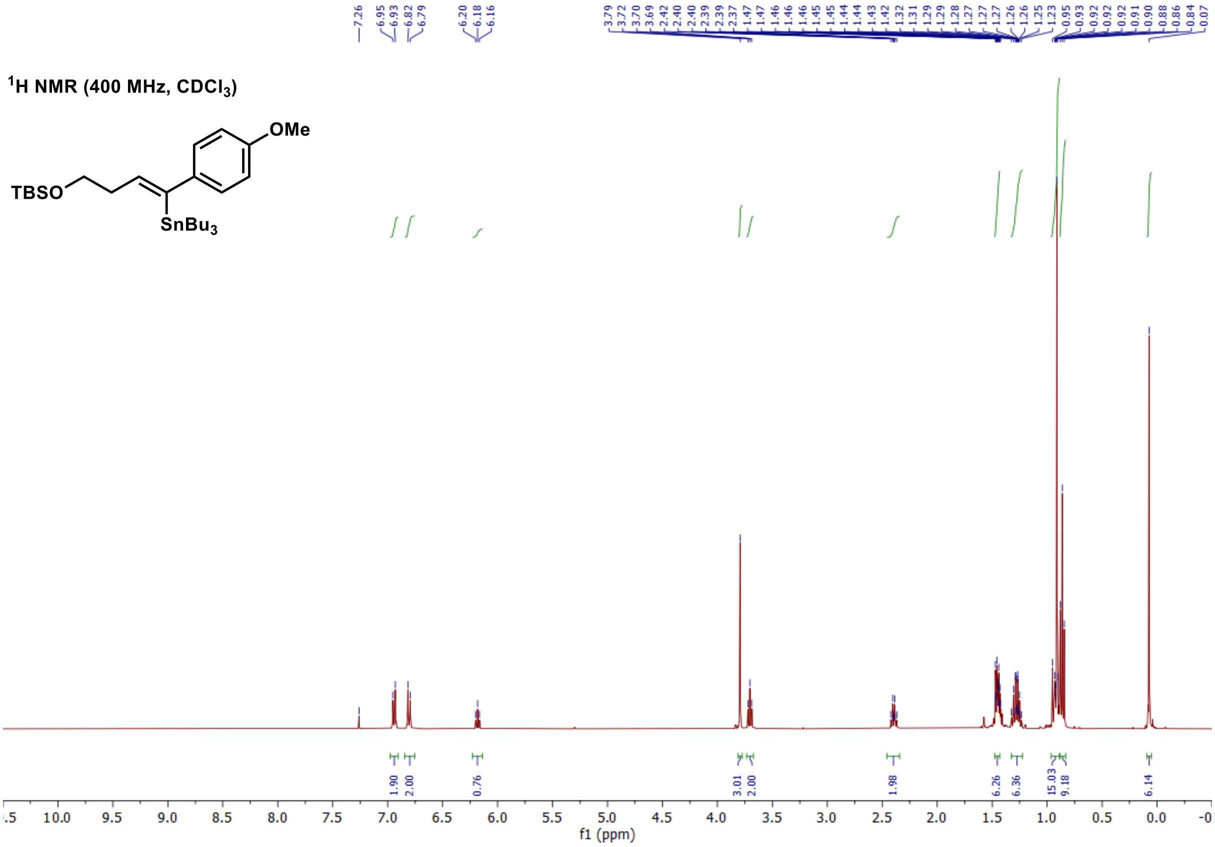

^{13}C NMR (101 MHz, $CDCl_3$)

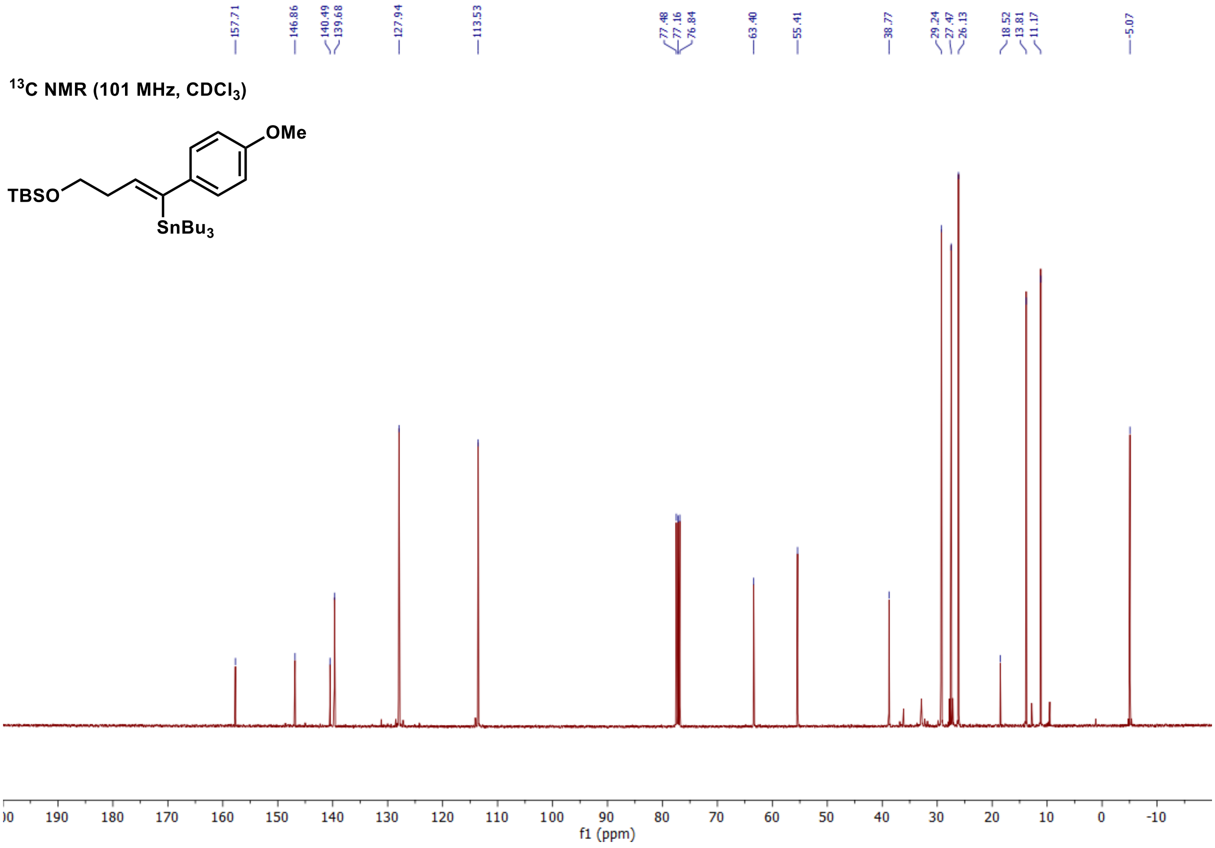

^{119}Sn NMR (149 MHz, $CDCl_3$)

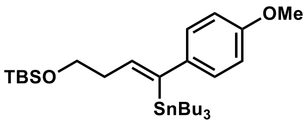

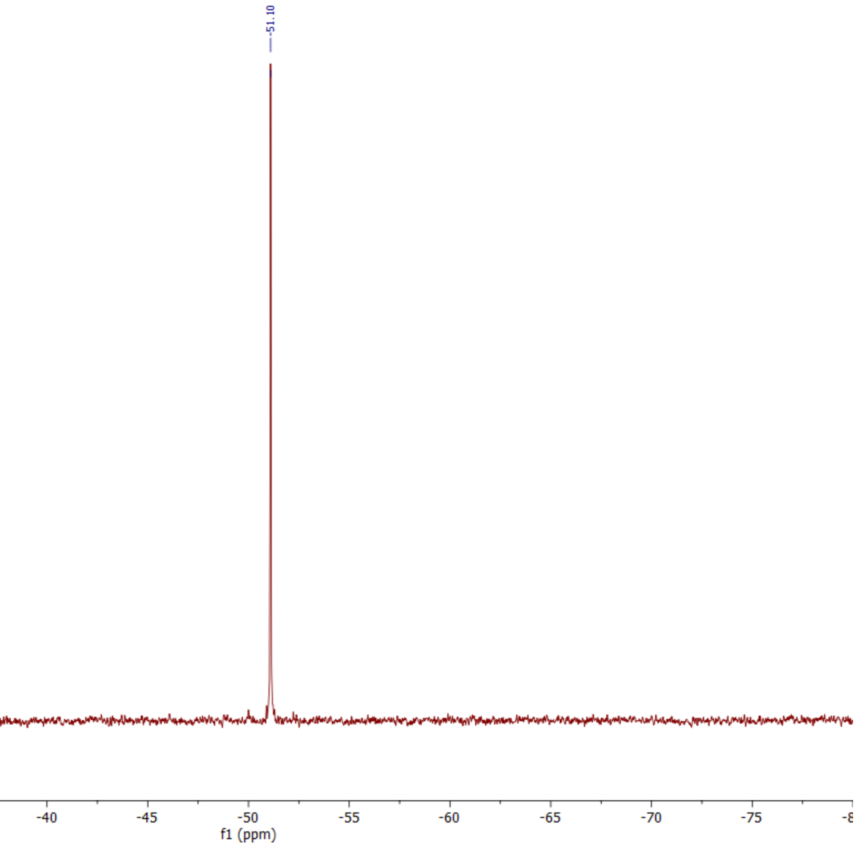

^{1}H NMR (400 MHz, $CDCl_3$)

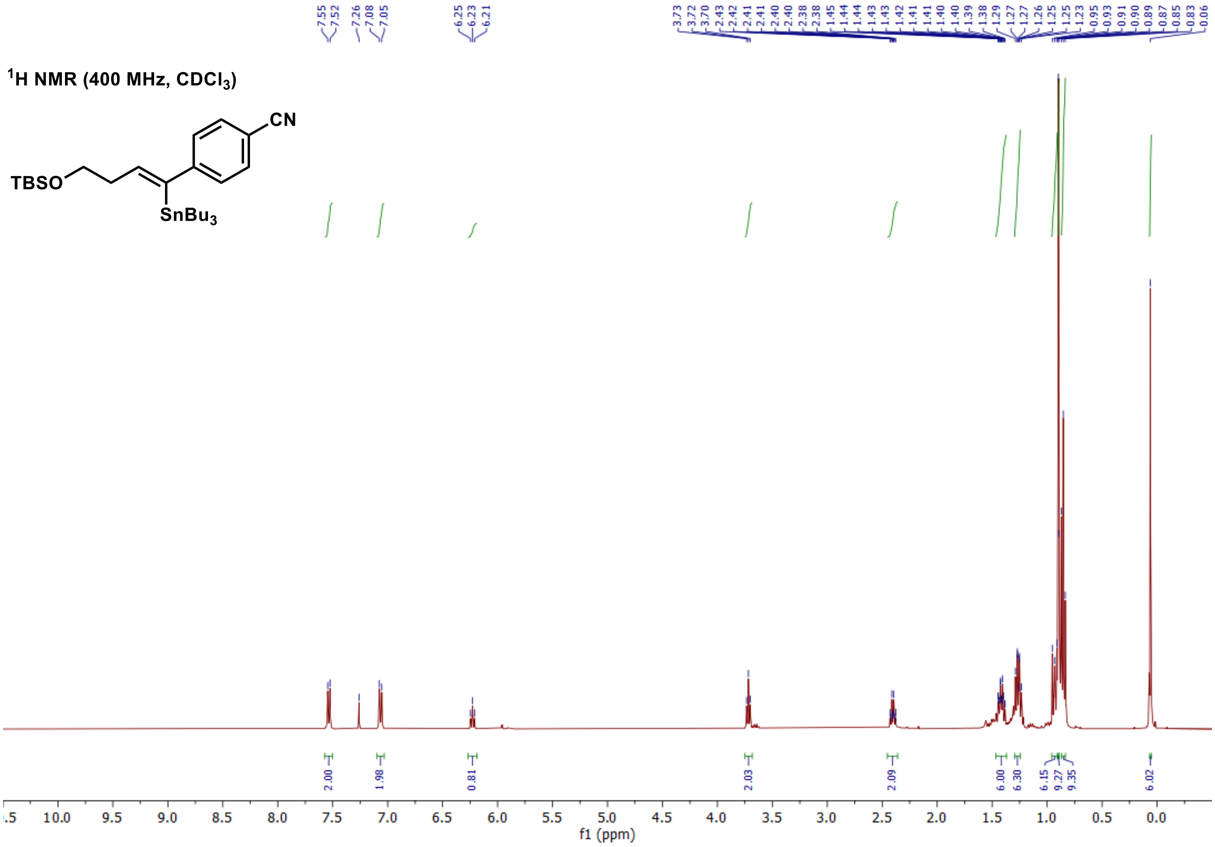

^{13}C NMR (101 MHz, $CDCl_3$)

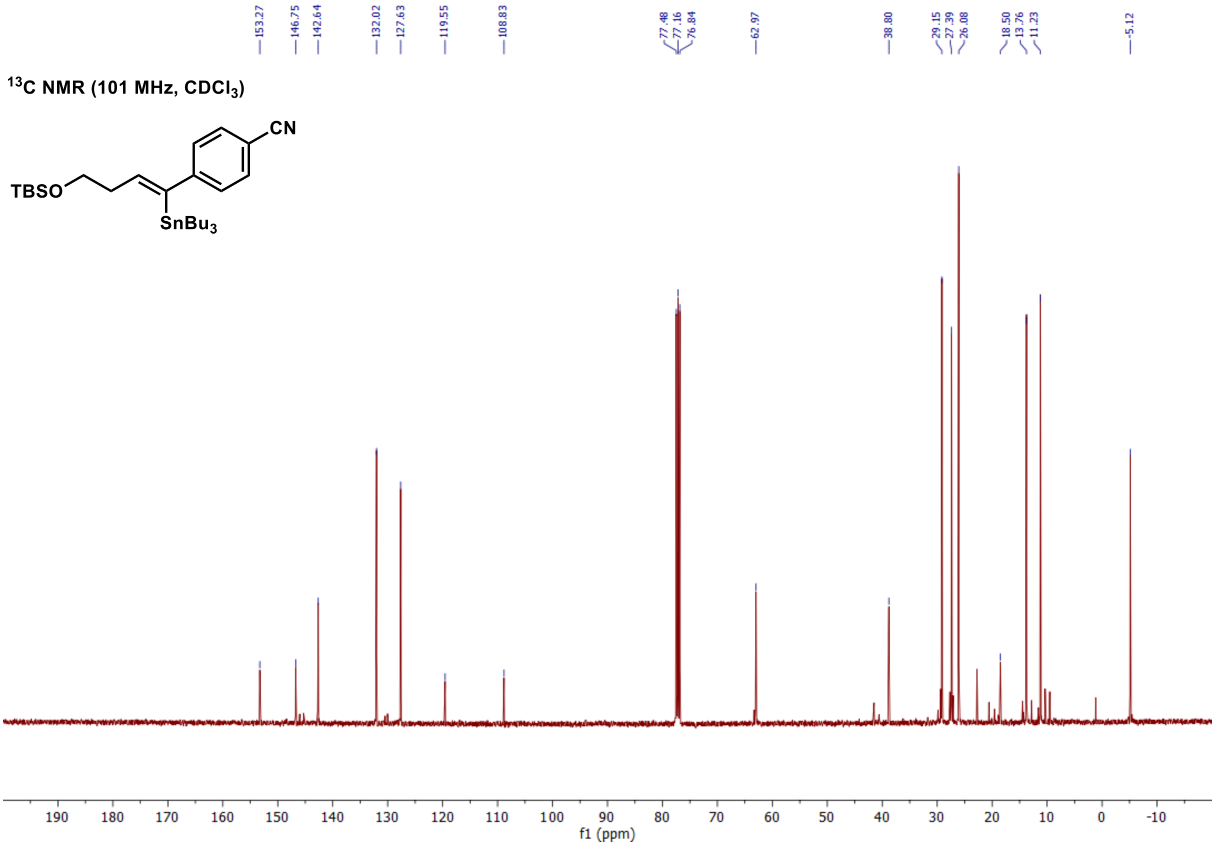

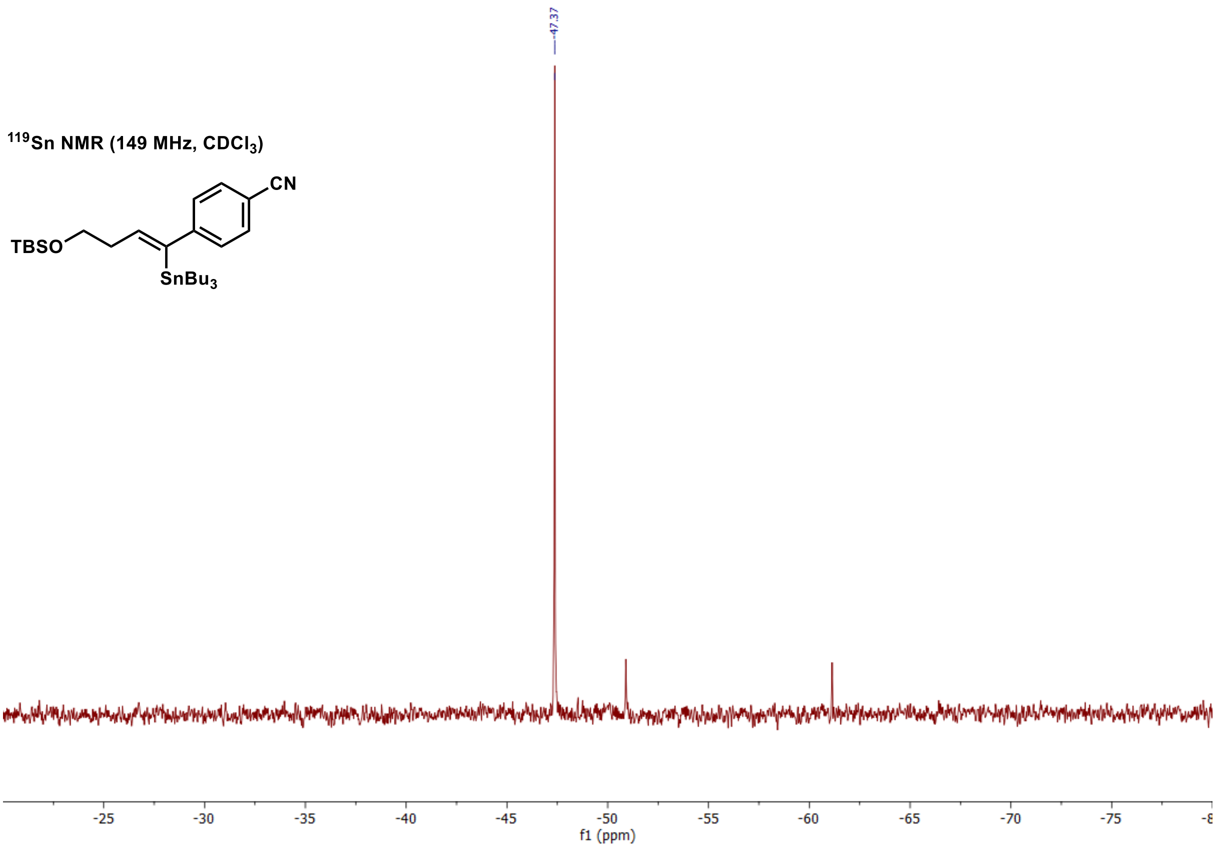

^{119}Sn NMR (149 MHz, CDCl$_3$)
TBSO
CN
SnBu$_3$
-47.37
-25
-30
-35
-40
-45
-50
-55
-60
-65
-70
-75
f1 (ppm)

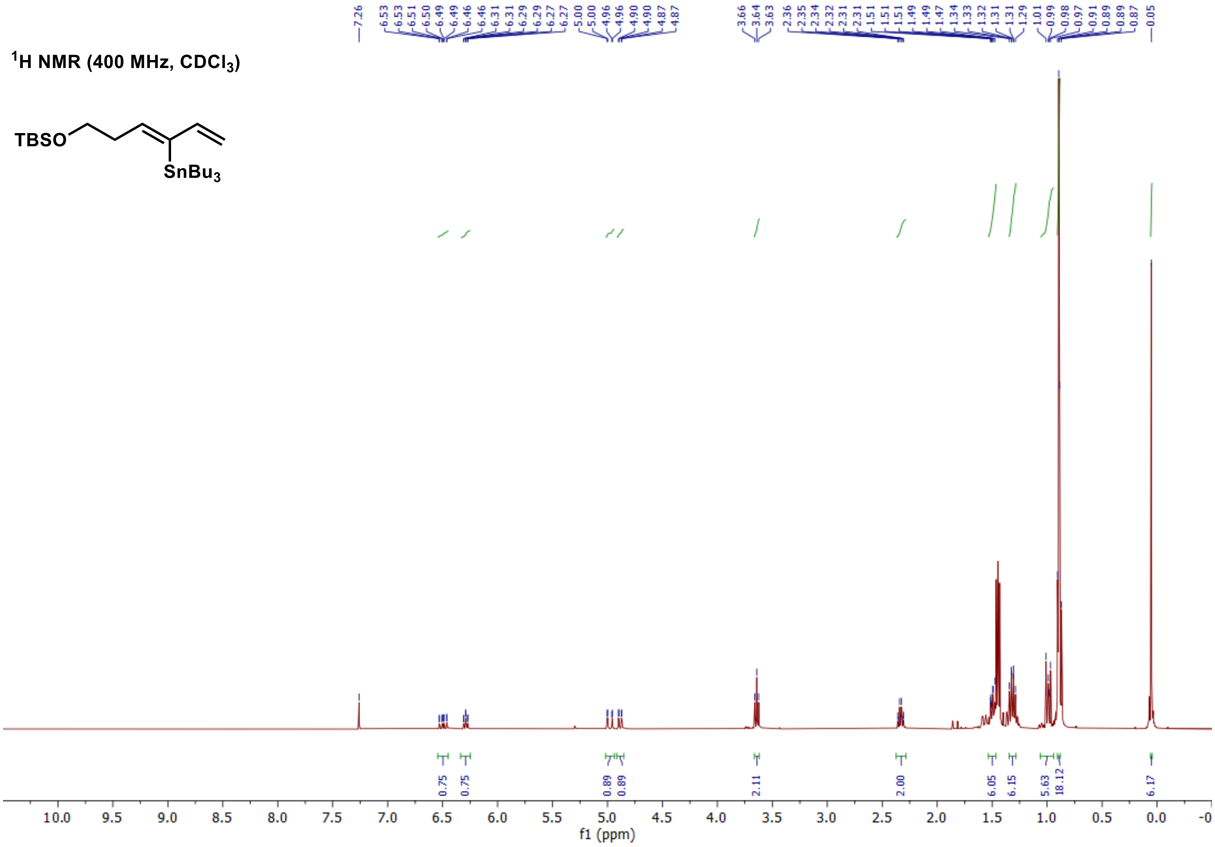

^{1}H NMR (400 MHz, CDCl$_3$)
TBSO
SnBu$_3$
f1 (ppm)

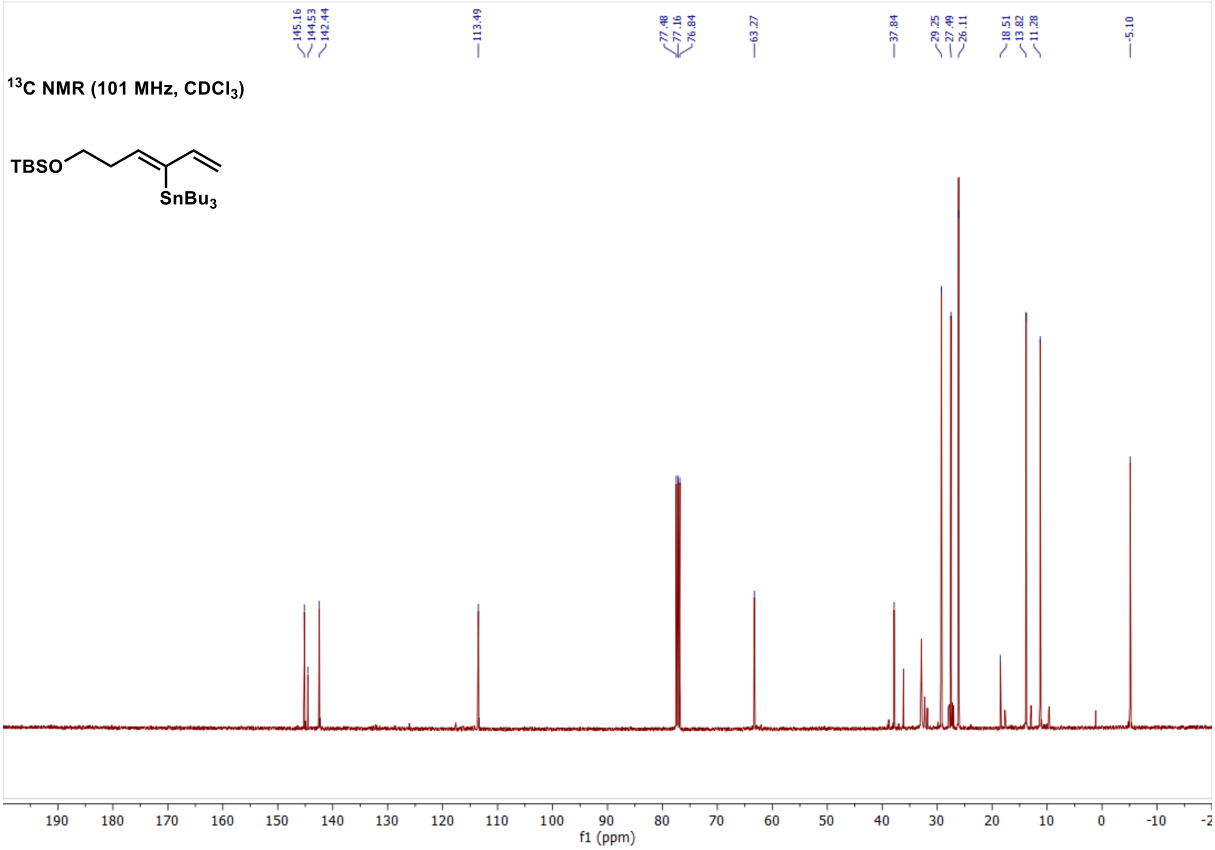

^{13}C NMR (101 MHz, CDCl$_3$)
TBSO
SnBu$_3$
f1 (ppm)

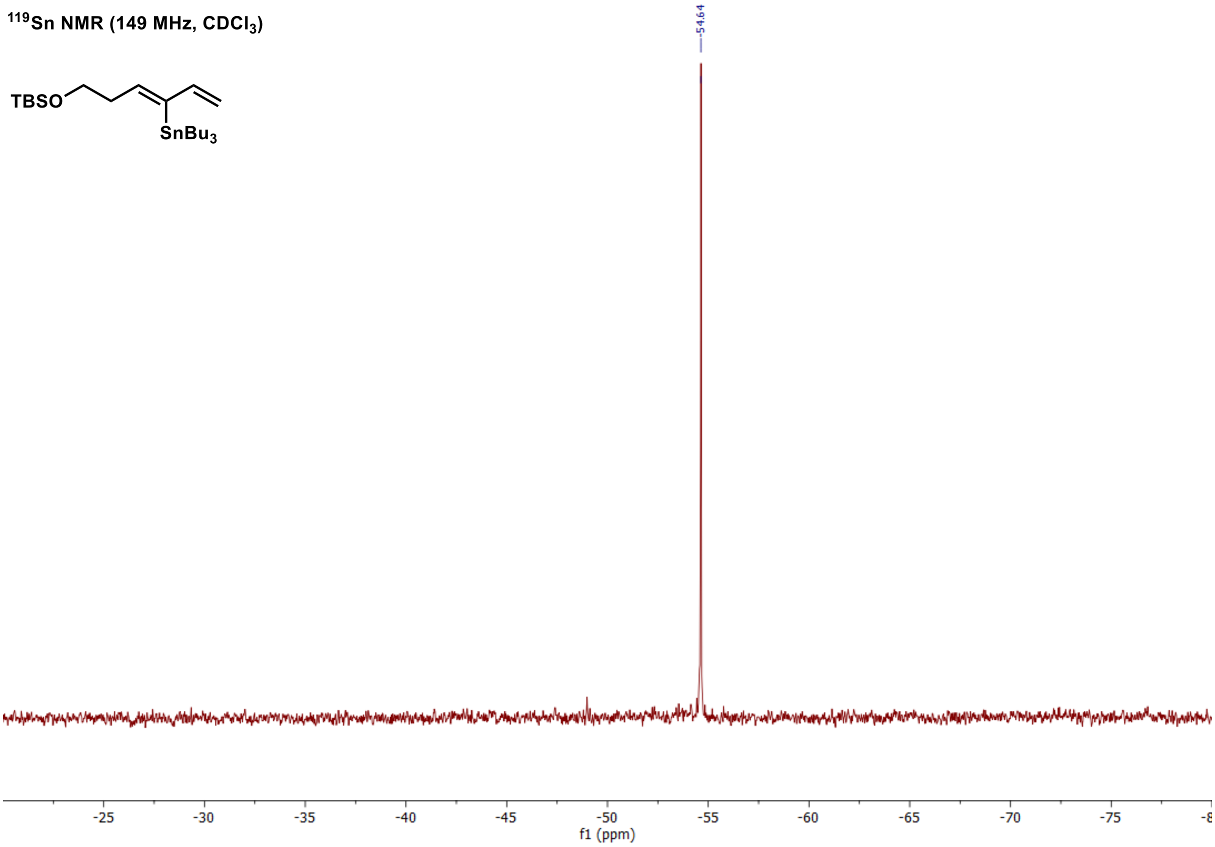

^{119}Sn NMR (149 MHz, CDCl$_3$)
TBSO
SnBu$_3$
54.64
-25
-30
-35
-40
-45
-50
-55
-60
-65
-70
-75
f1 (ppm)

^{1}H NMR (400 MHz, $CDCl_3$)

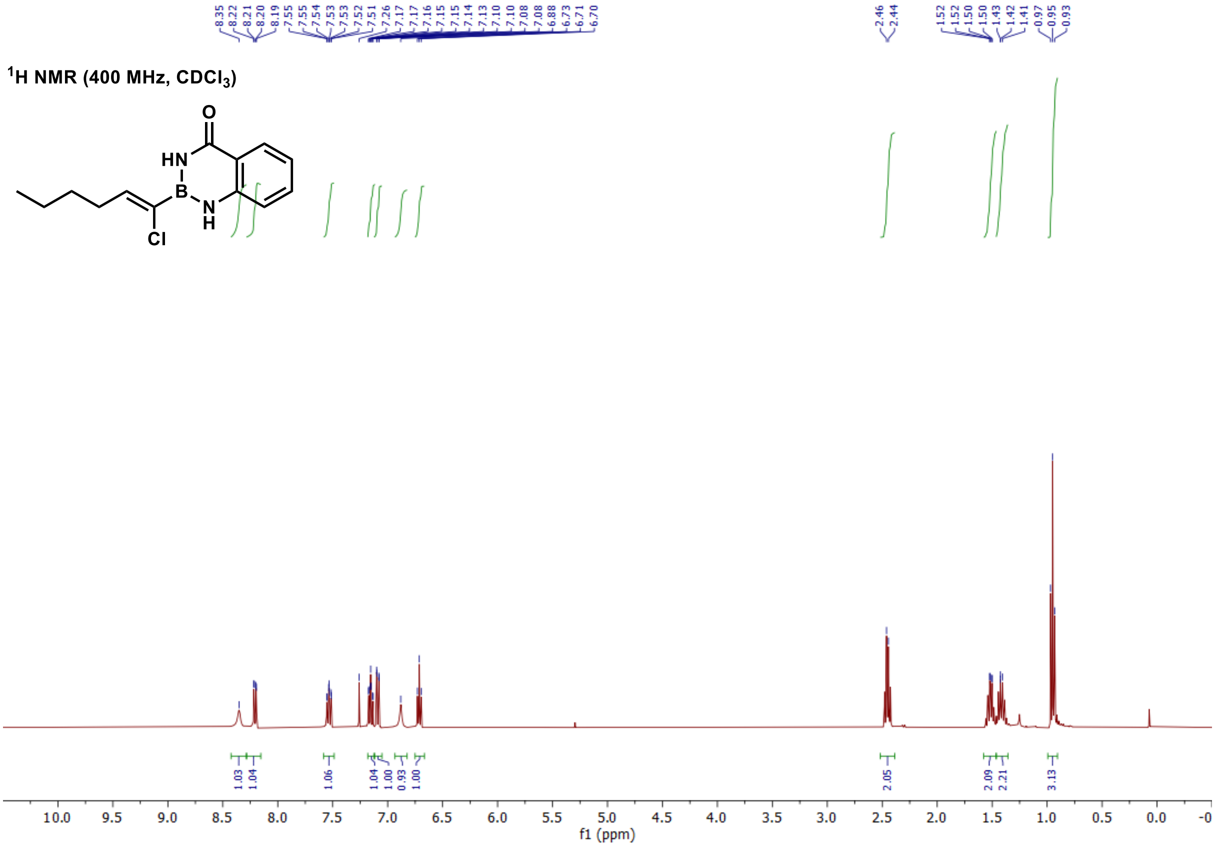

^{13}C NMR (101 MHz, $CDCl_3$)

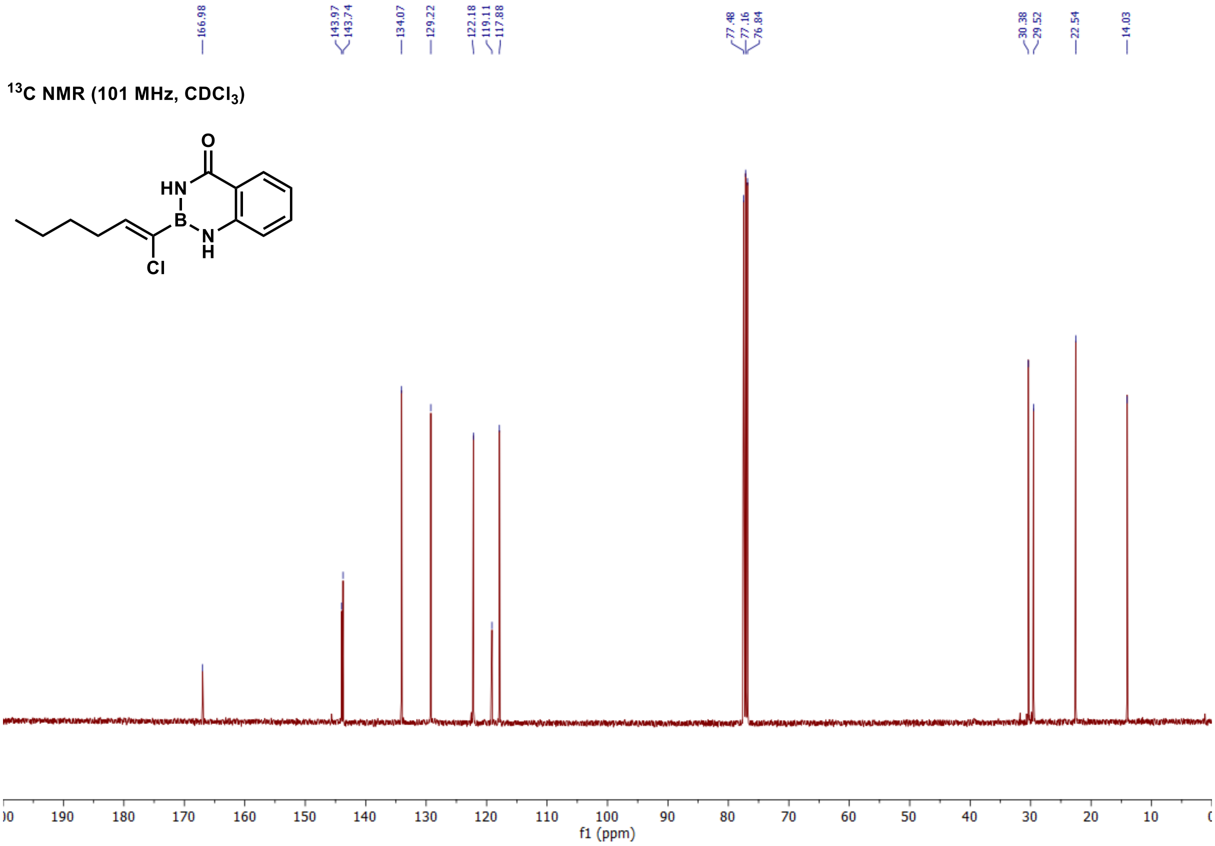

^{11}B NMR (128 MHz, $CDCl_3$)

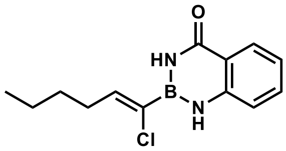

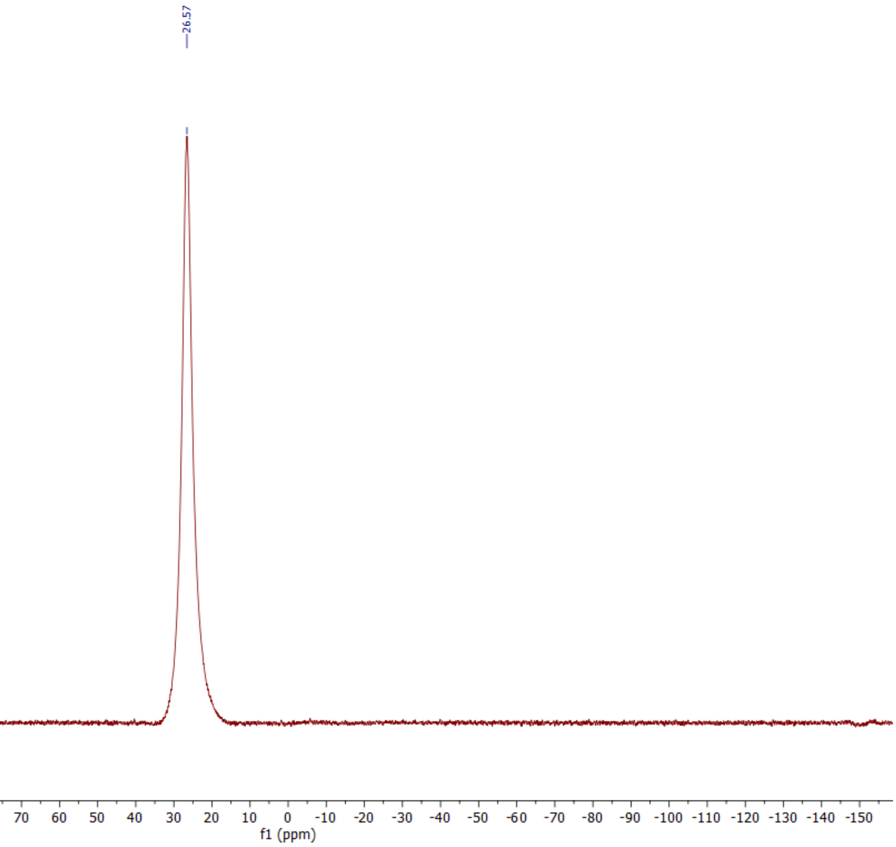

^{1}H NMR (400 MHz, $CDCl_3$)

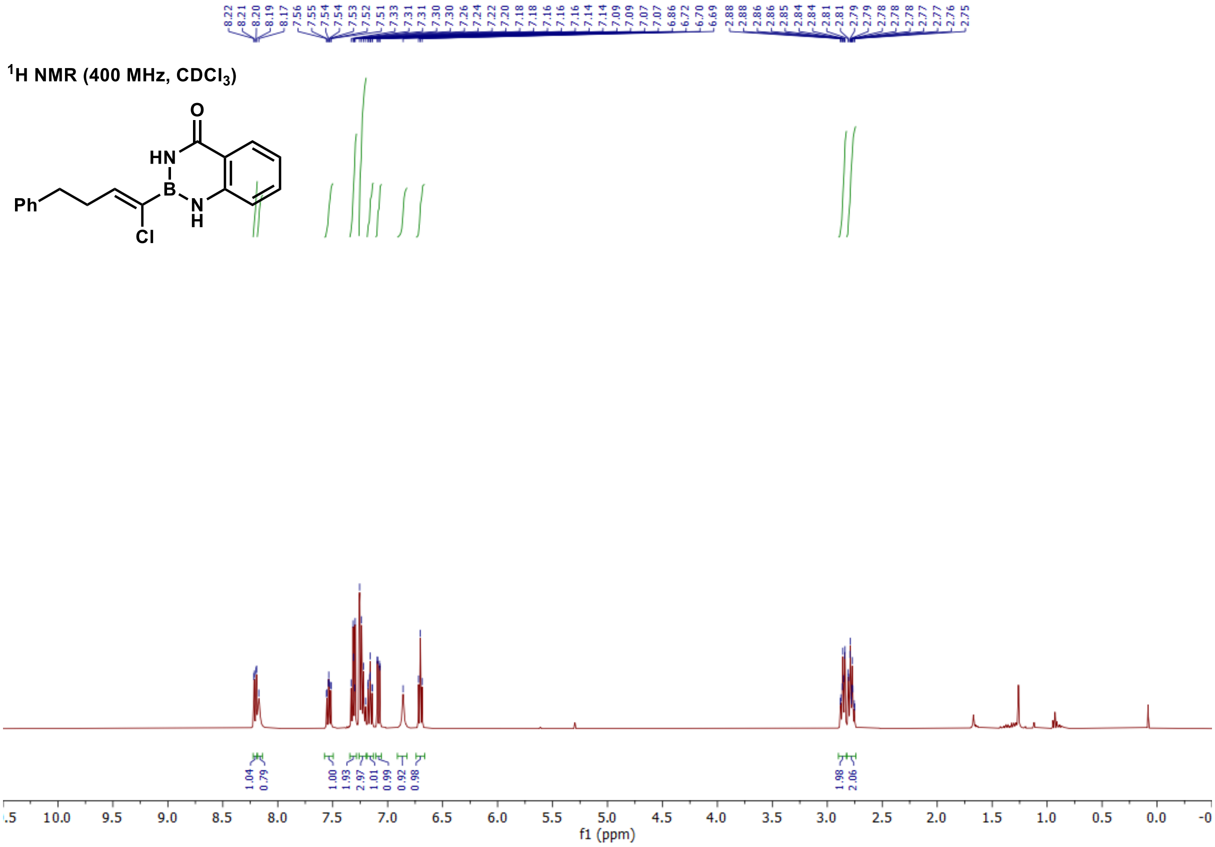

^{13}C NMR (101 MHz, $CDCl_3$)

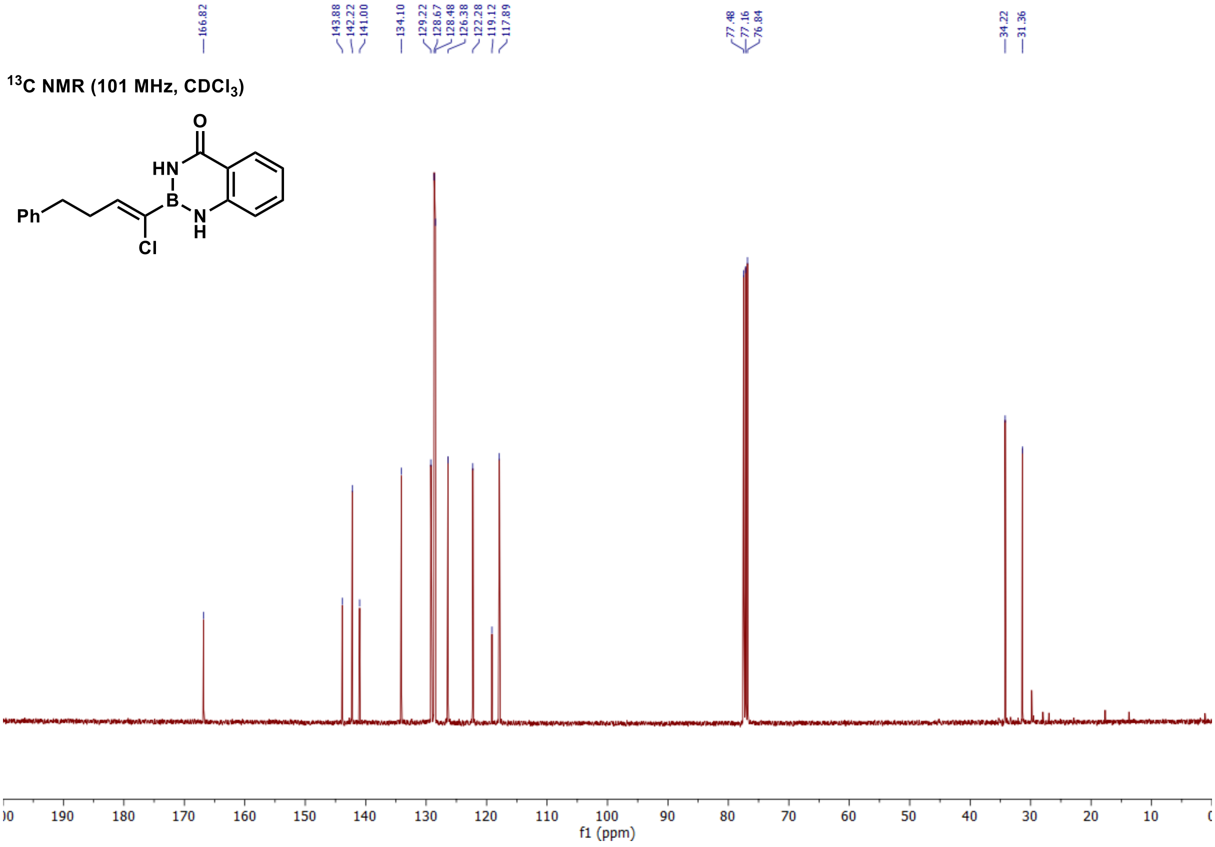

^{11}B NMR (128 MHz, $CDCl_3$)

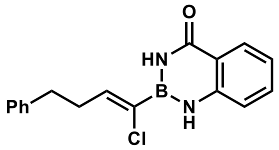

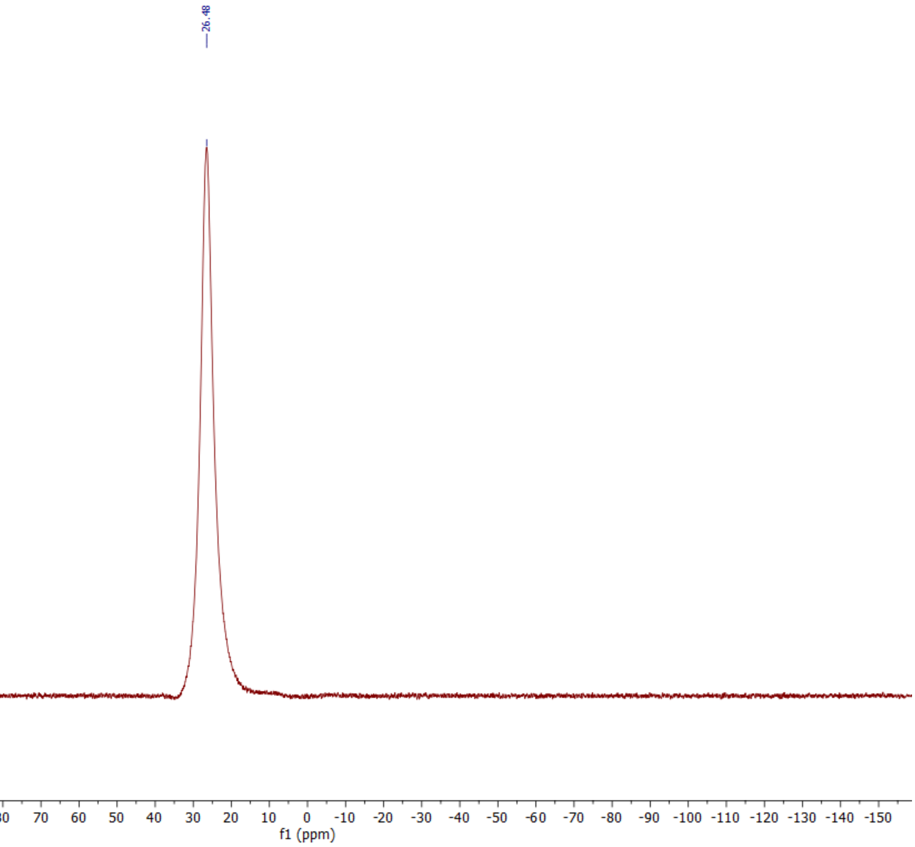

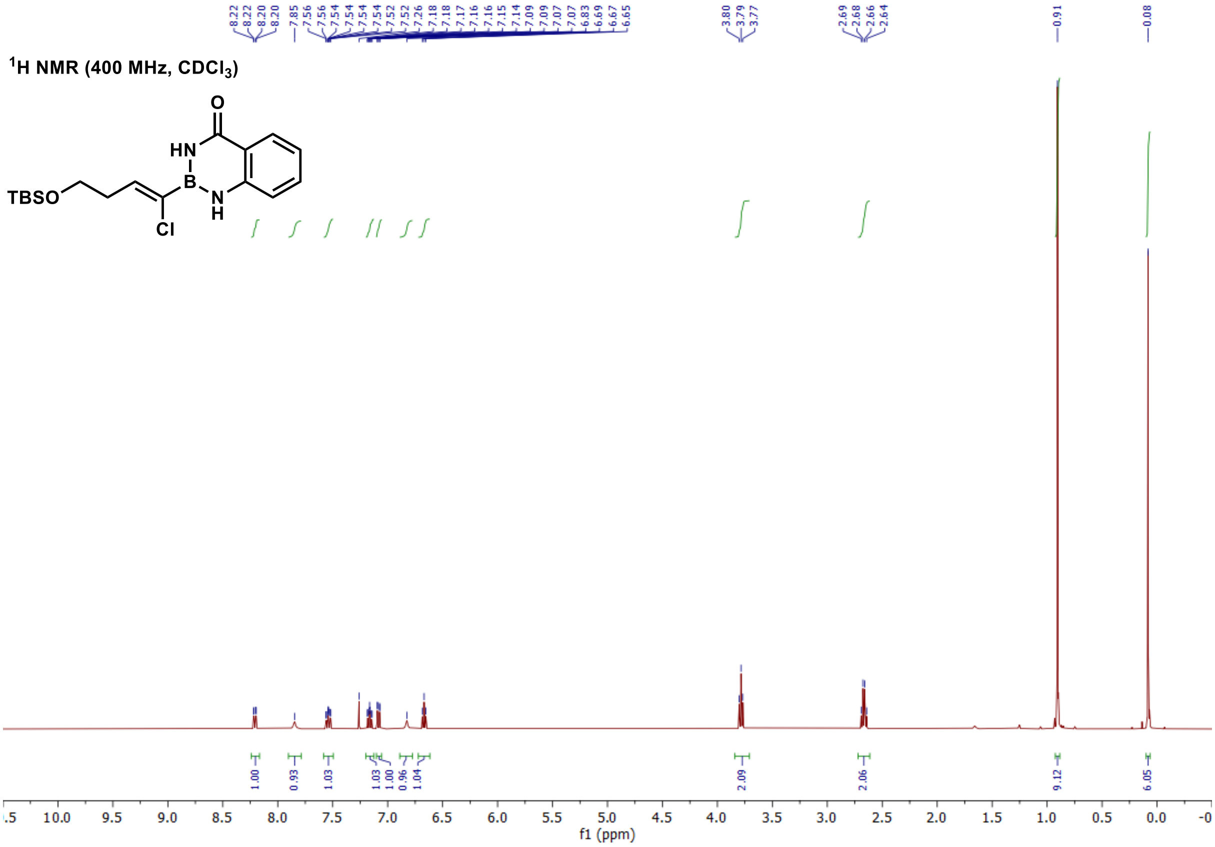

^{1}H NMR (400 MHz, CDCl$_3$)
TBSO
HN
B
N
H
O
Cl
f1 (ppm)

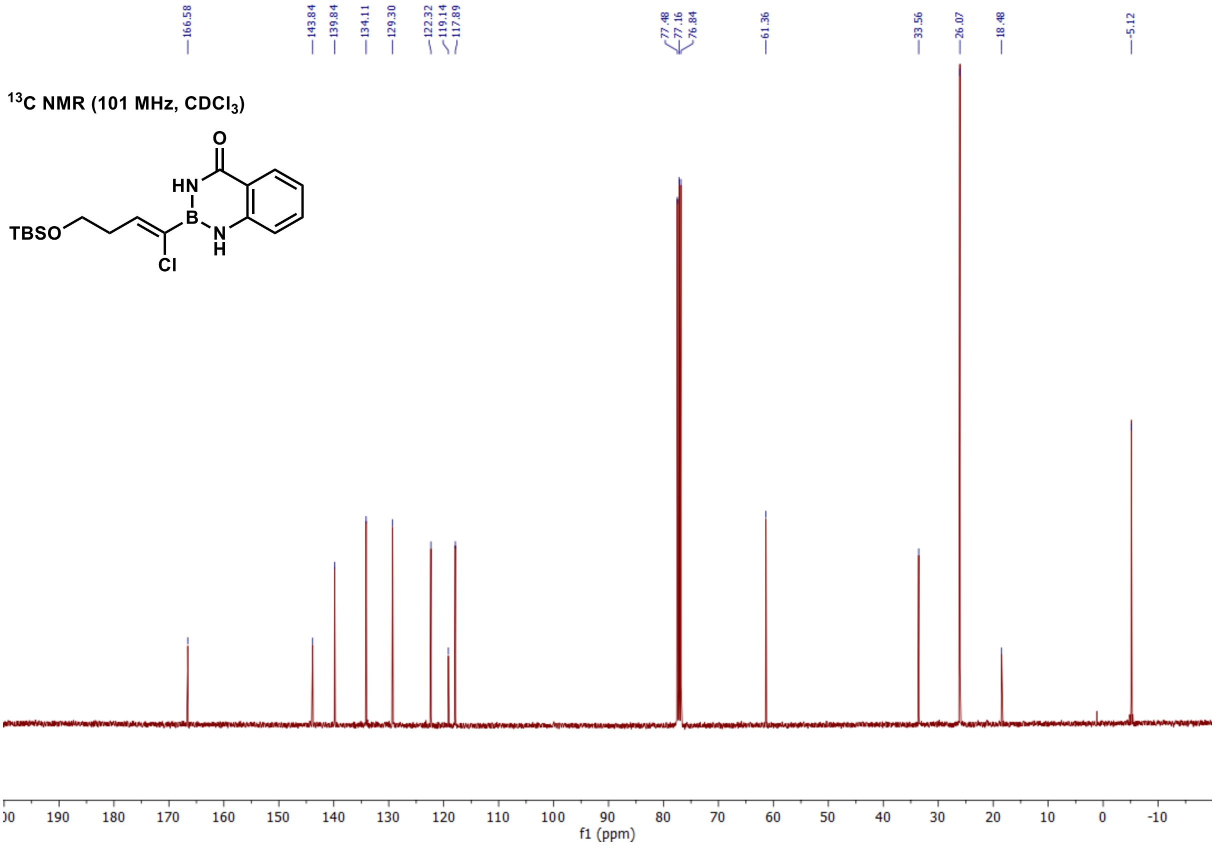

^{13}C NMR (101 MHz, CDCl$_3$)
TBSO
HN
B
N
H
O
Cl
f1 (ppm)

^{11}B NMR (128 MHz, $CDCl_3$)

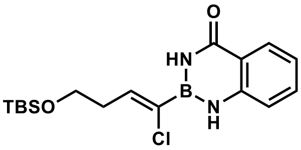

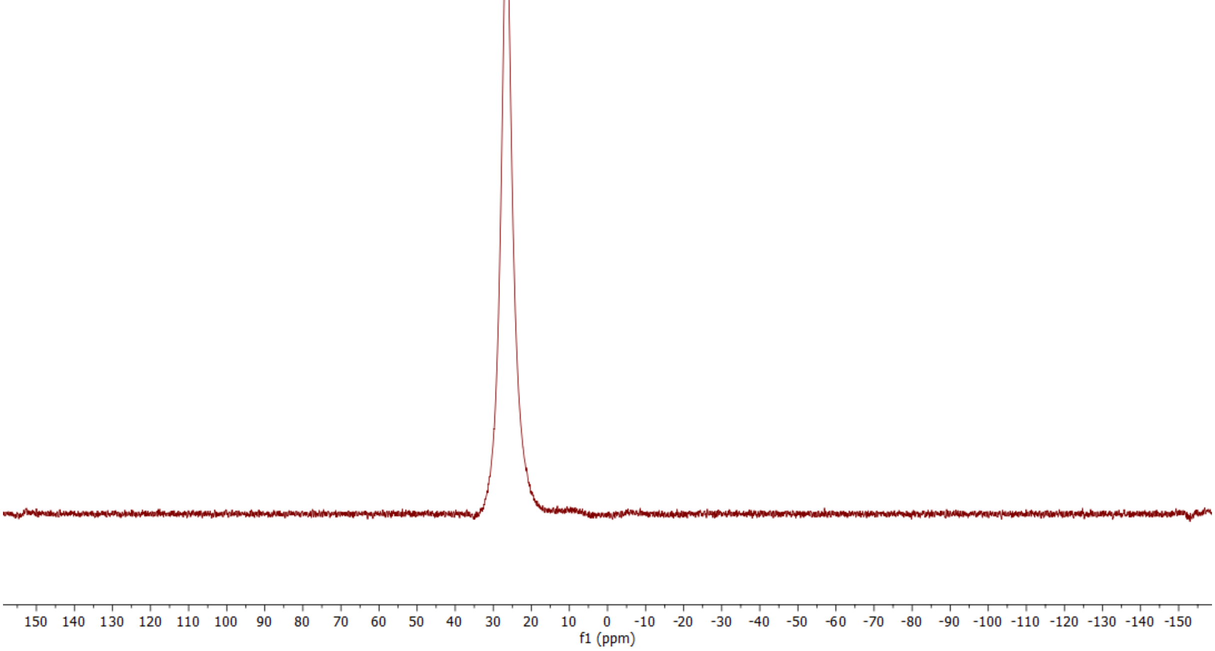

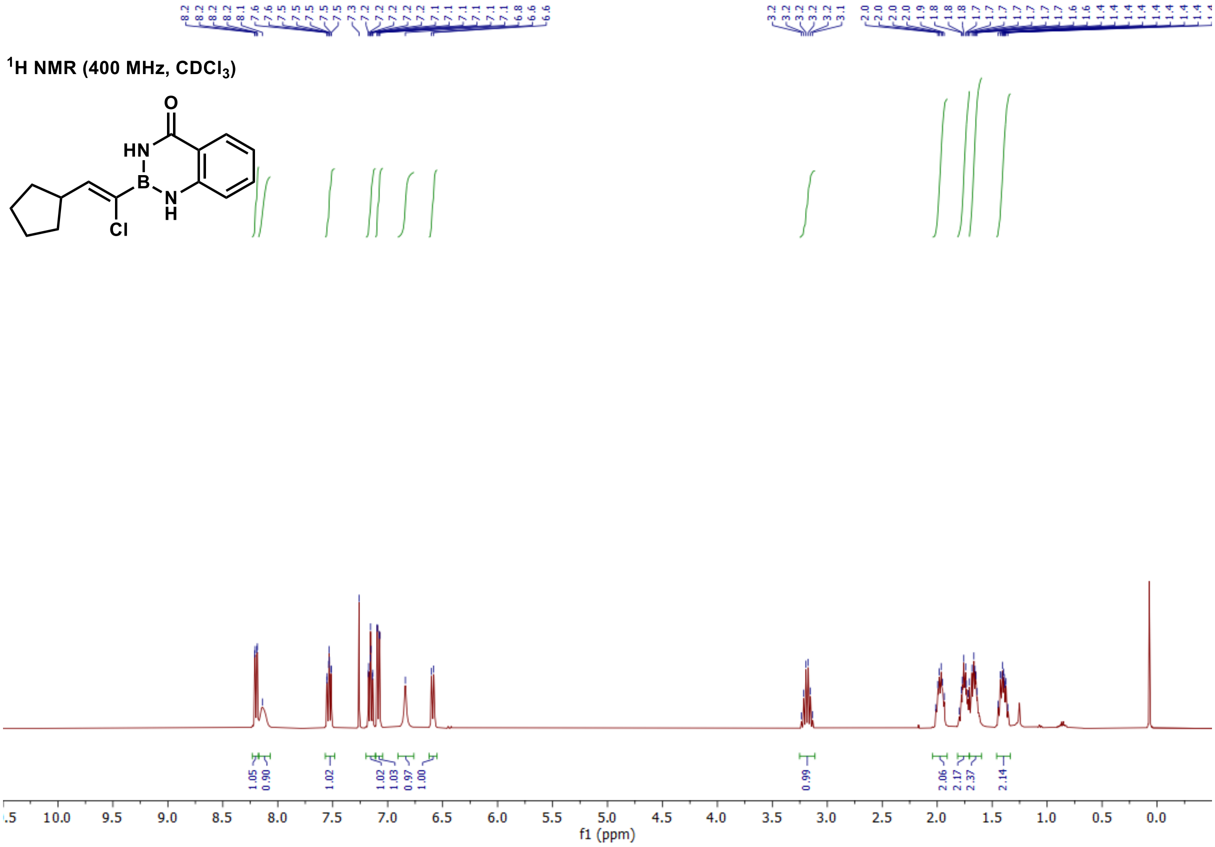
^{1}H NMR (400 MHz, CDCl$_3$)
O
HN
B
N
H
Cl
f1 (ppm)

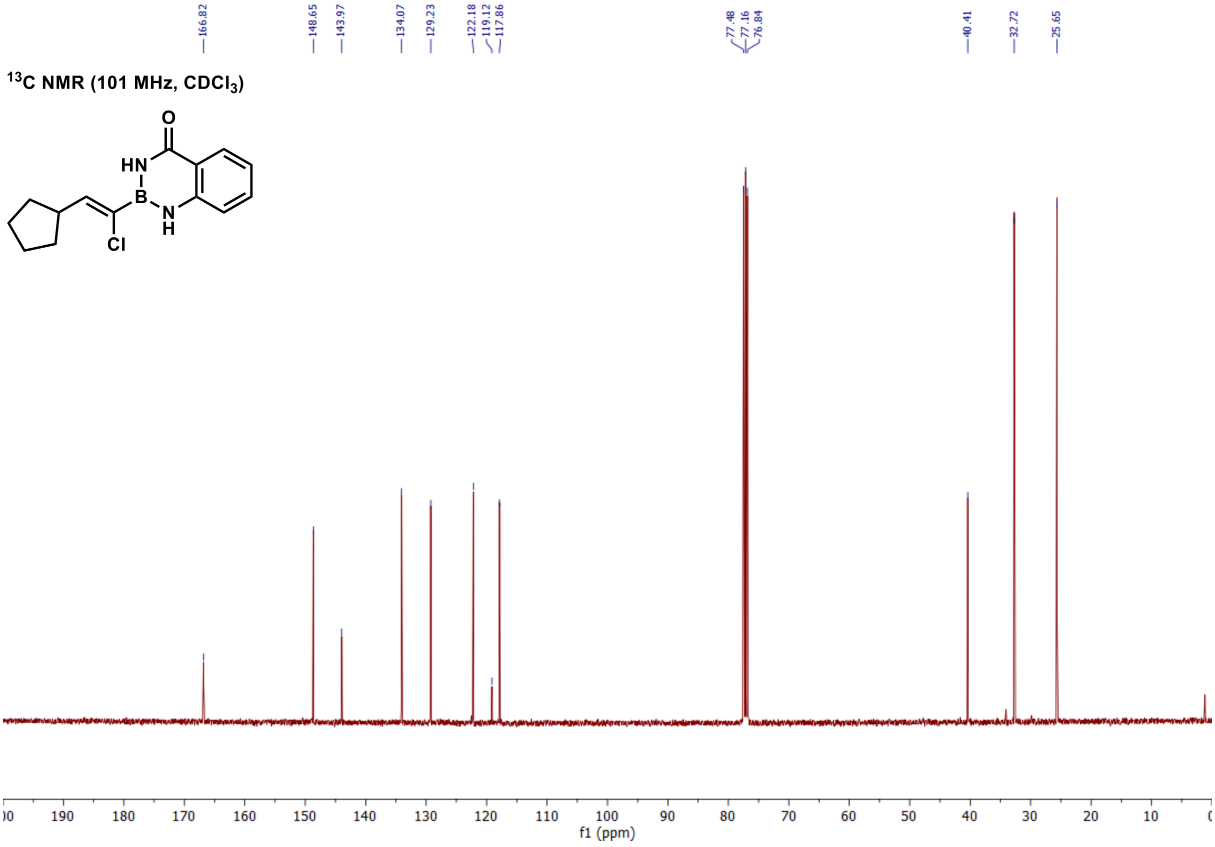
^{13}C NMR (101 MHz, CDCl$_3$)
O
HN
B
N
H
Cl
f1 (ppm)

^{11}B NMR (128 MHz, $CDCl_3$)

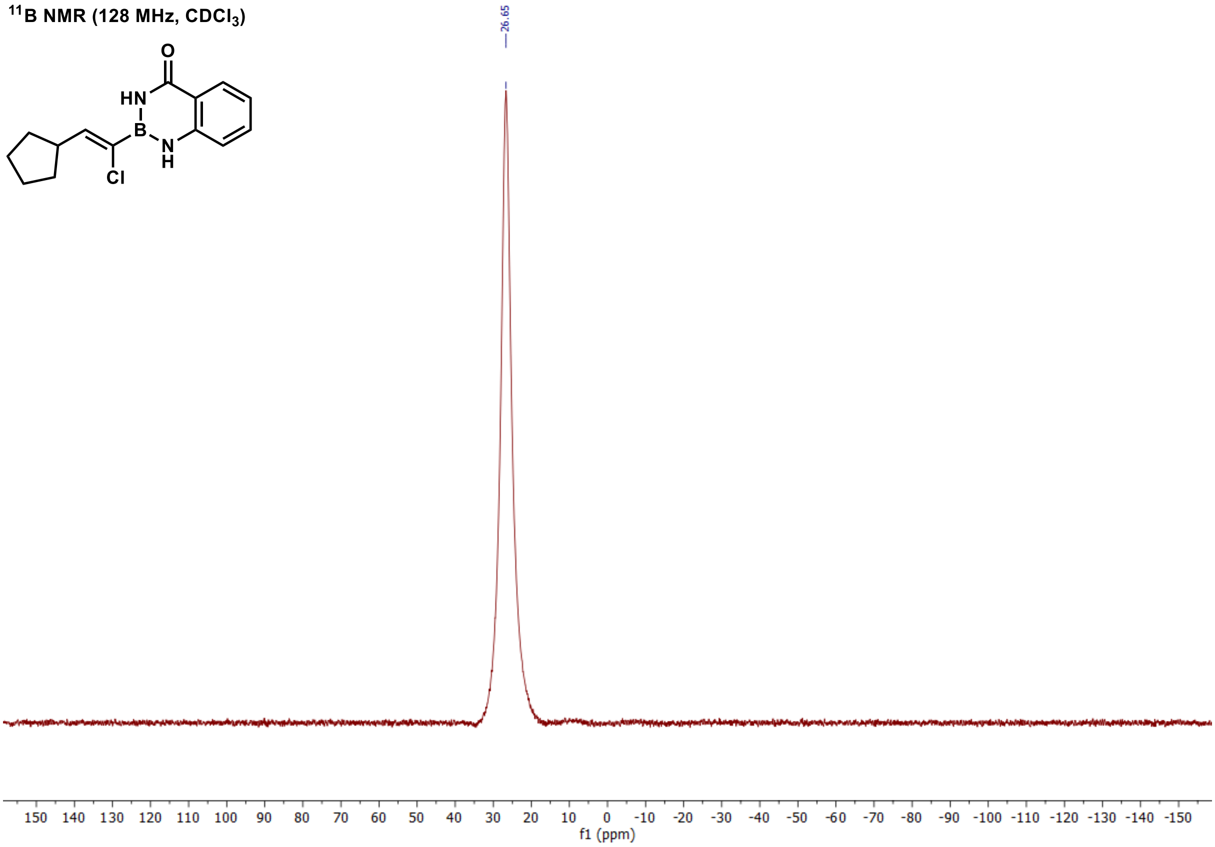

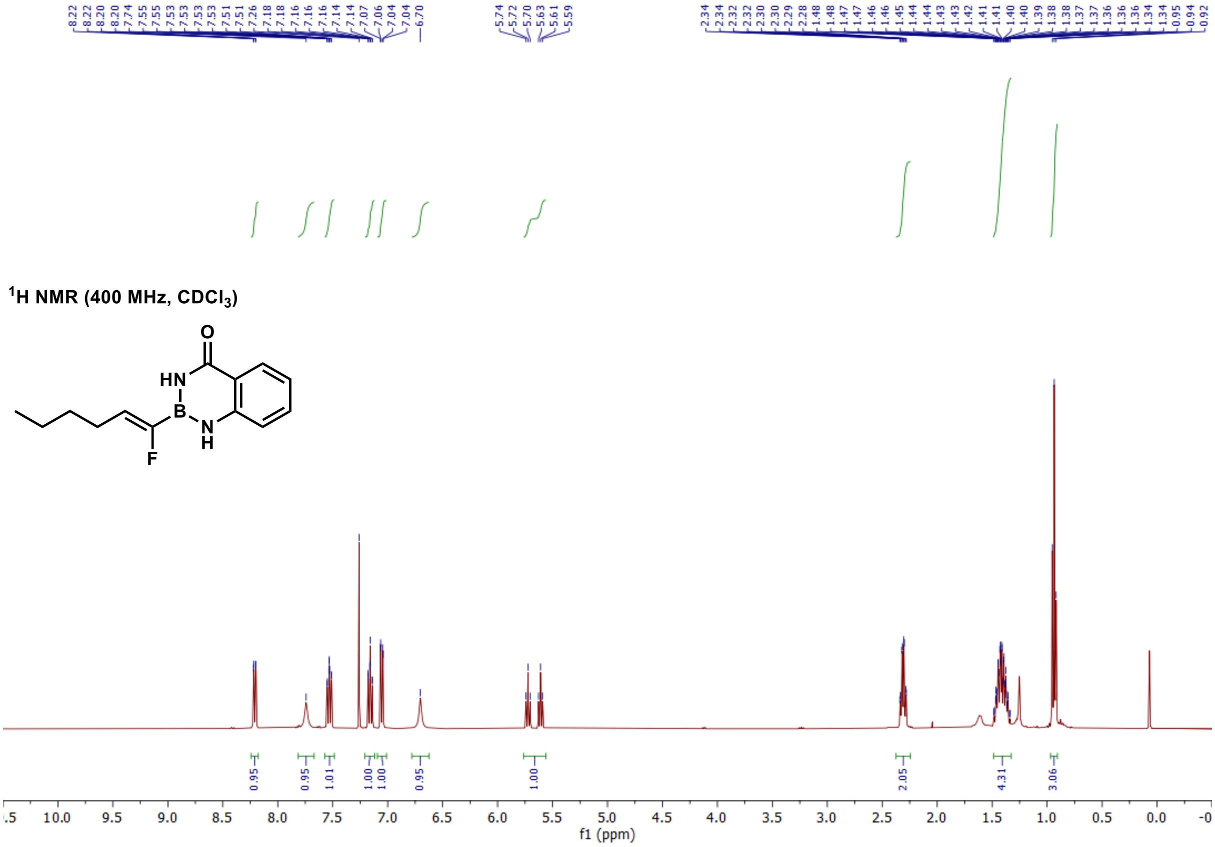

1H NMR (400 MHz, CDCl3)
O
HN
B
N
H
F
f1 (ppm)

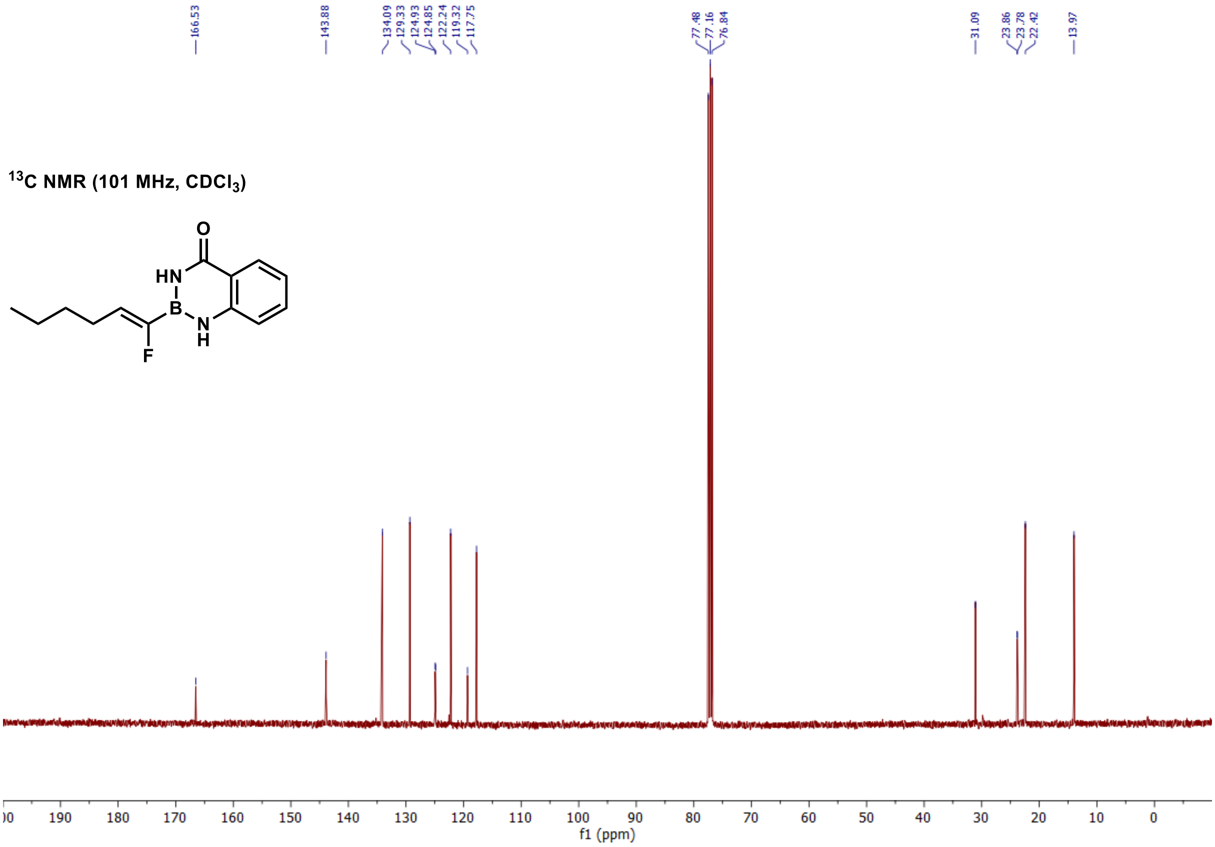

13C NMR (101 MHz, CDCl3)
O
HN
B
N
H
F
f1 (ppm)

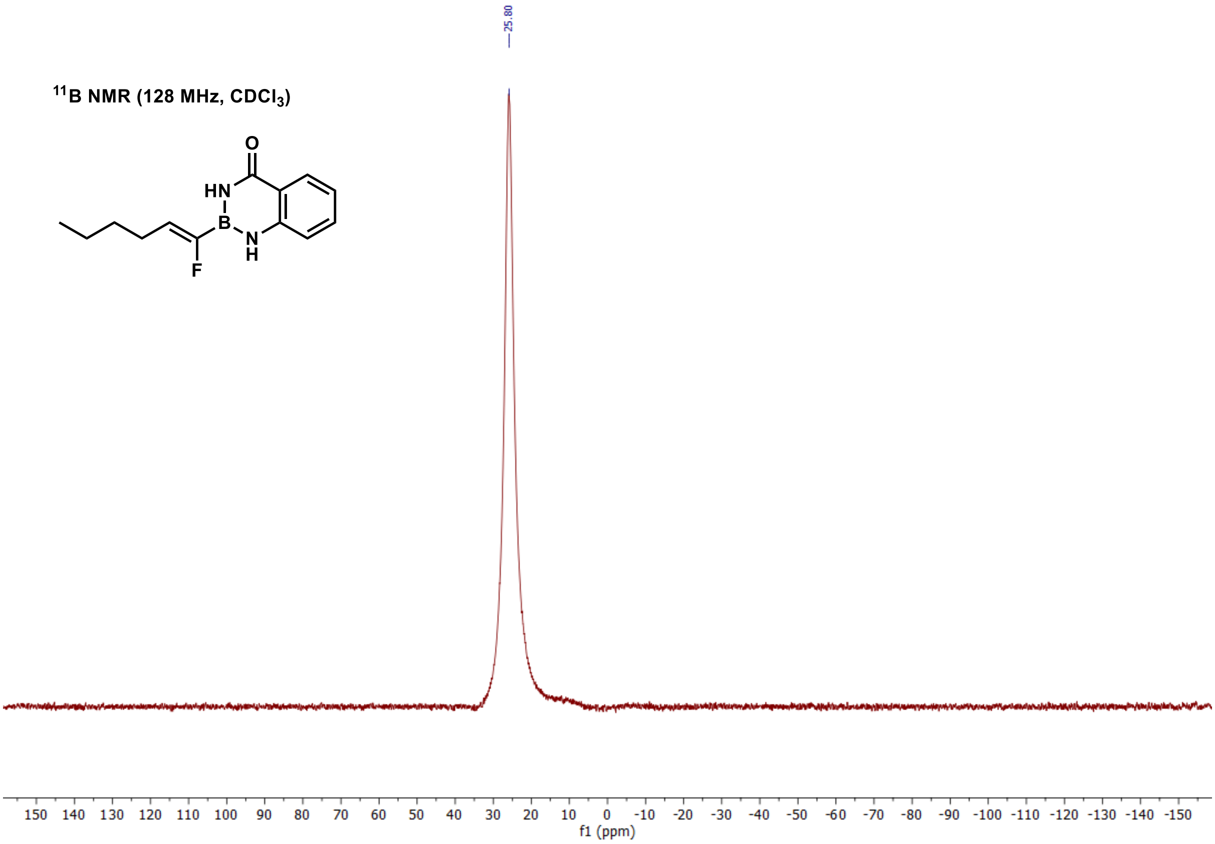
25.80
^{11}B NMR (128 MHz, $CDCl_3$)
O
HN
B
N
H
F
150 140 130 120 110 100 90 80 70 60 50 40 30 20 10 0 -10 -20 -30 -40 -50 -60 -70 -80 -90 -100 -110 -120 -130 -140 -150
f1 (ppm)

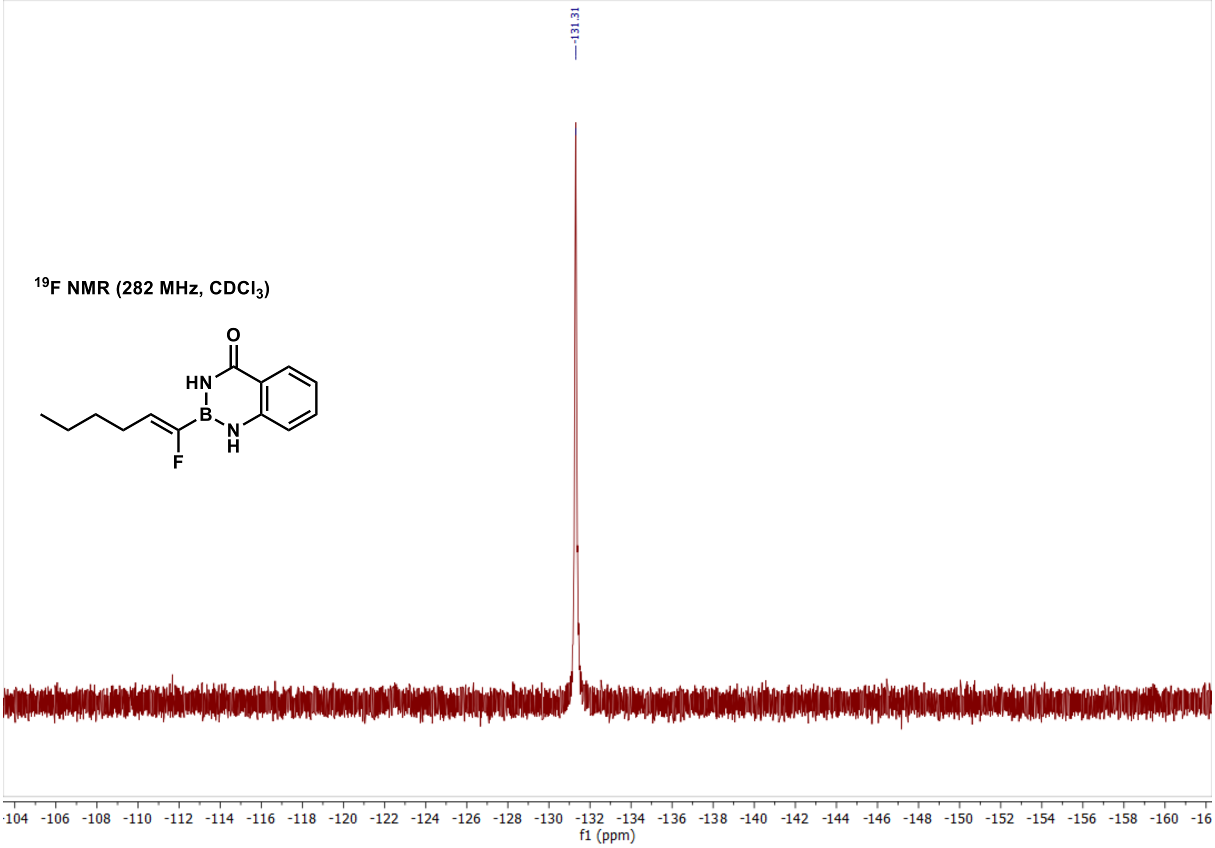
-131.31
^{19}F NMR (282 MHz, $CDCl_3$)
O
HN
B
N
H
F
-104 -106 -108 -110 -112 -114 -116 -118 -120 -122 -124 -126 -128 -130 -132 -134 -136 -138 -140 -142 -144 -146 -148 -150 -152 -154 -156 -158 -160 -16
f1 (ppm)

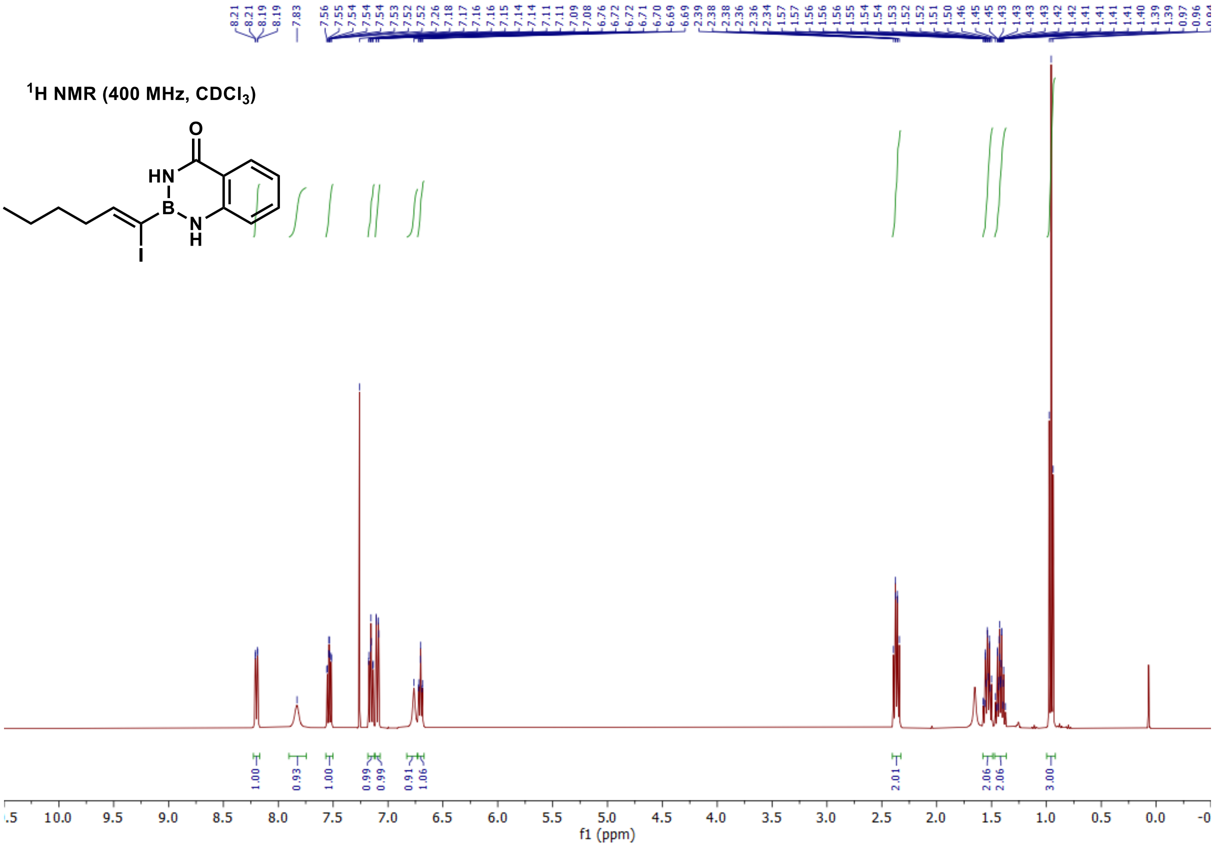

1H NMR (400 MHz, CDCl3)
O
HN
B
N
H
I
1.00
0.93
1.00
0.99
0.99
0.91
1.06
2.01
2.06
2.06
3.00
f1 (ppm)

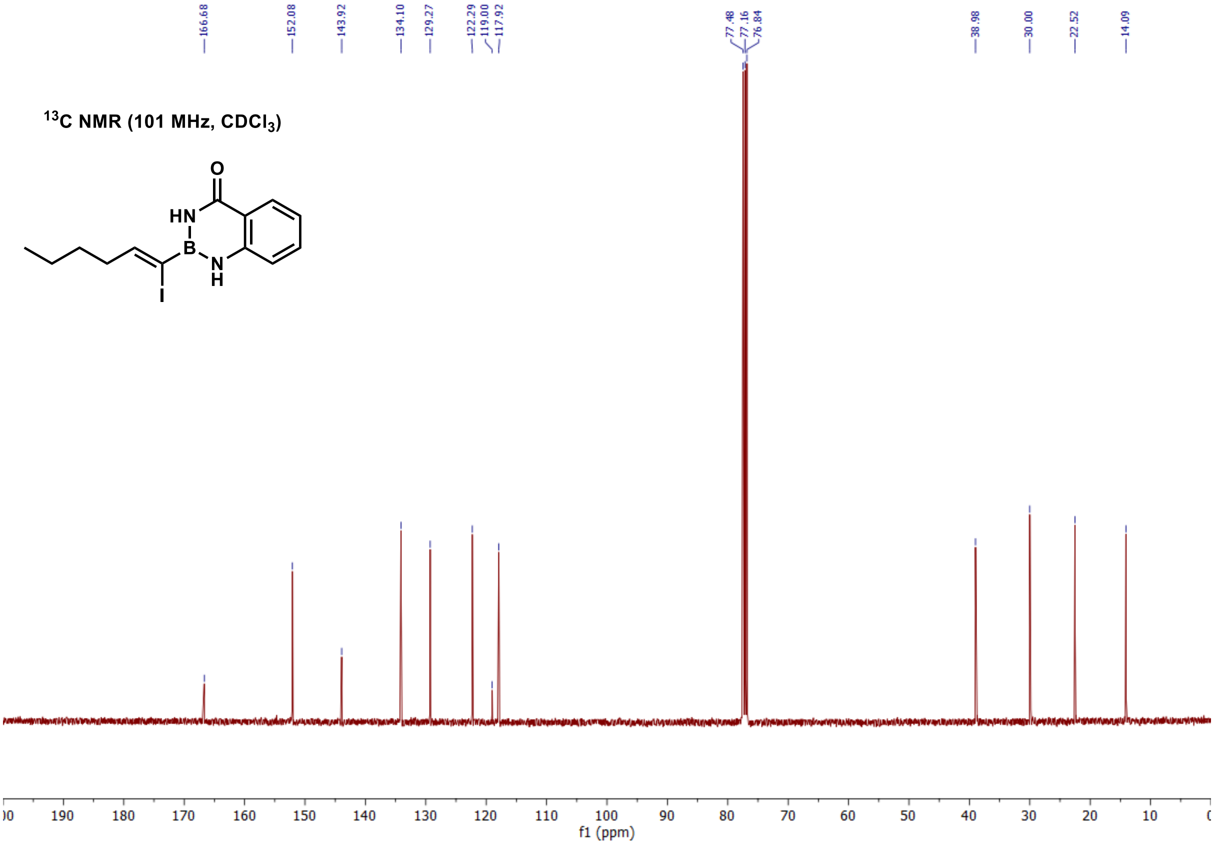

166.68
152.08
143.92
134.10
129.27
122.29
119.00
117.92
77.48
77.16
76.84
38.98
30.00
22.52
14.09
13C NMR (101 MHz, CDCl3)
O
HN
B
N
H
I
f1 (ppm)

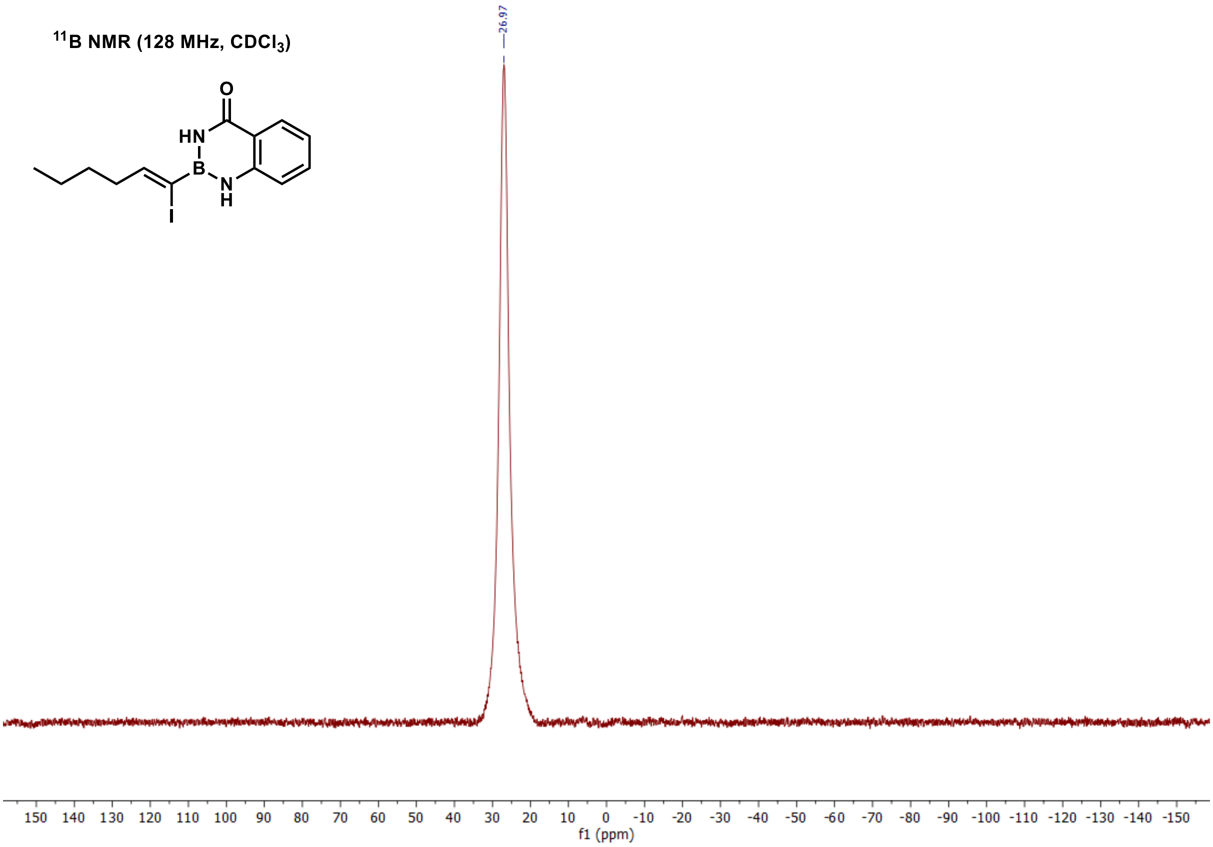

11B NMR (128 MHz, CDCl3)
O
HN
B
N
H
I
26.97
150 140 130 120 110 100 90 80 70 60 50 40 30 20 10 0 -10 -20 -30 -40 -50 -60 -70 -80 -90 -100 -110 -120 -130 -140 -150
f1 (ppm)

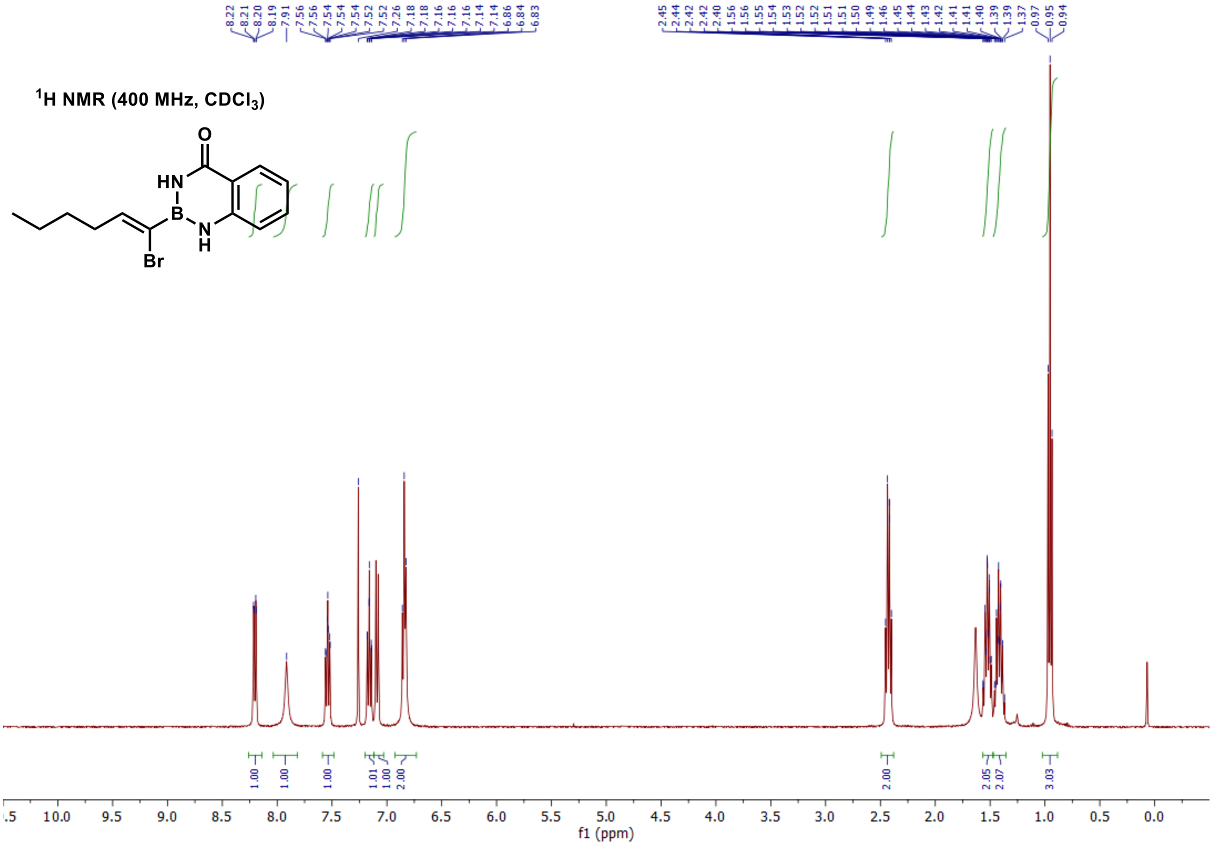
8.22
8.21
8.20
8.19
7.91
7.56
7.56
7.54
7.54
7.54
7.52
7.52
7.26
7.18
7.18
7.16
7.16
7.16
7.14
6.86
6.84
6.83
2.45
2.44
2.42
2.42
2.40
1.56
1.56
1.55
1.54
1.53
1.52
1.52
1.51
1.51
1.50
1.49
1.46
1.45
1.44
1.43
1.42
1.41
1.41
1.40
1.39
1.39
1.37
0.97
0.95
0.94
1H NMR (400 MHz, CDCl3)
O
HN
B
N
H
Br
1.00
1.00
1.00
1.01
1.00
2.00
2.00
2.05
2.07
3.03
f1 (ppm)

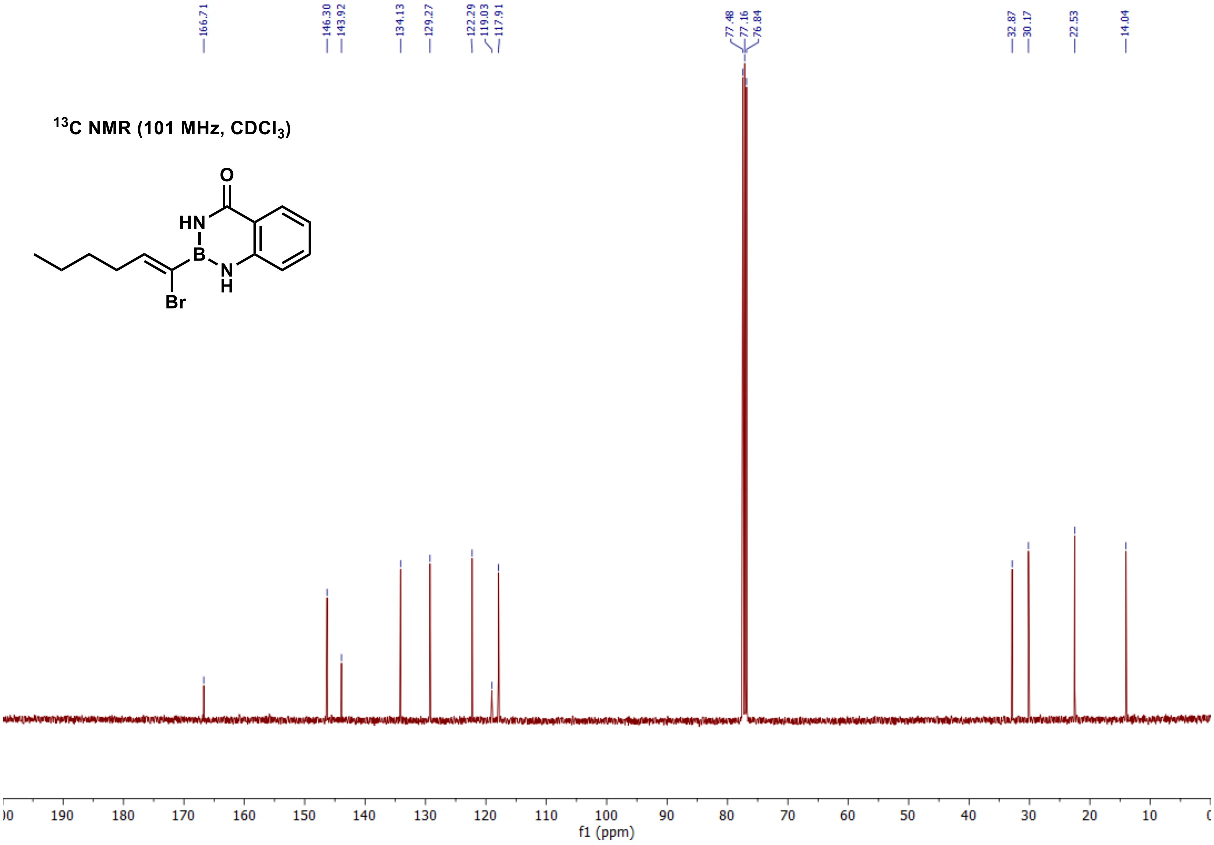
166.71
146.30
143.92
134.13
129.27
122.29
119.03
117.91
77.48
77.16
76.84
32.87
30.17
22.53
14.04
13C NMR (101 MHz, CDCl3)
O
HN
B
N
H
Br
f1 (ppm)

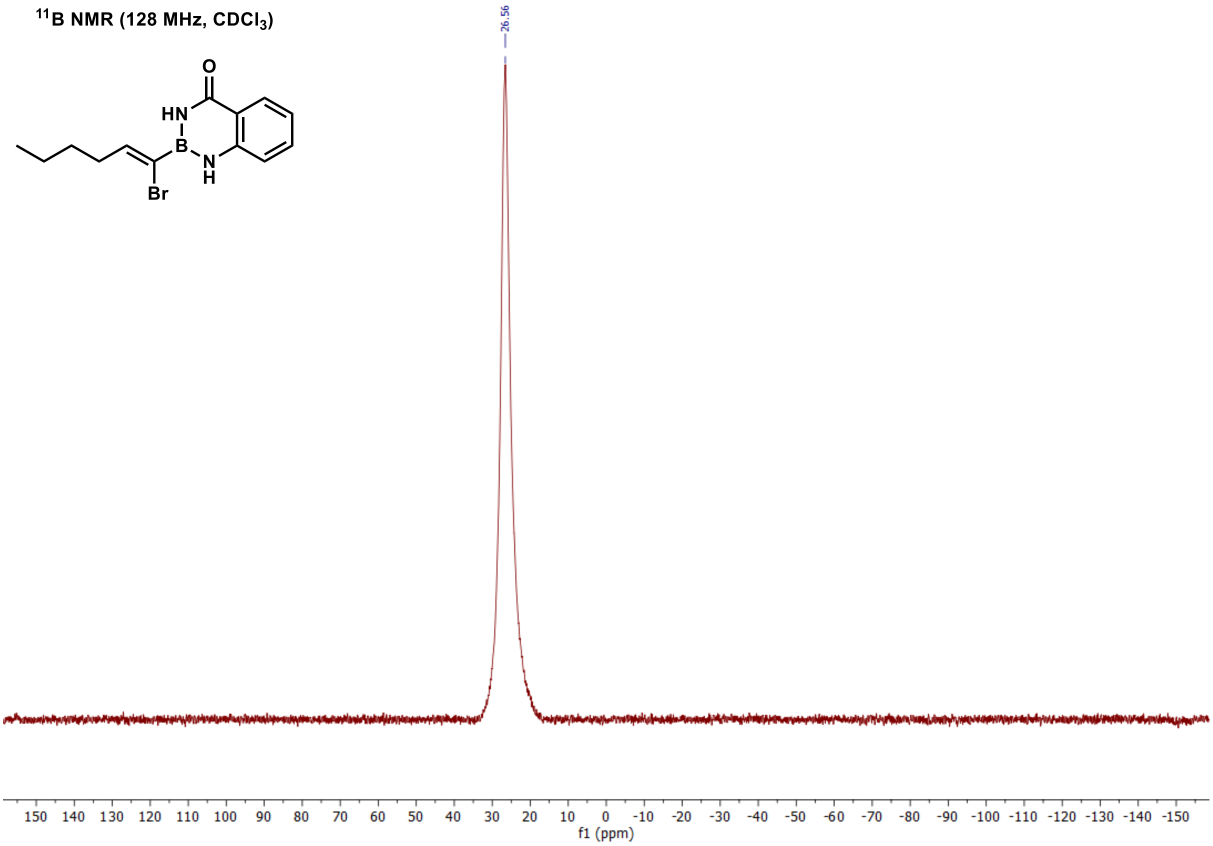

^{11}B NMR (128 MHz, CDCl$_3$)
O
HN
B
N
H
Br
26.56
150 140 130 120 110 100 90 80 70 60 50 40 30 20 10 0 -10 -20 -30 -40 -50 -60 -70 -80 -90 -100 -110 -120 -130 -140 -150
f1 (ppm)

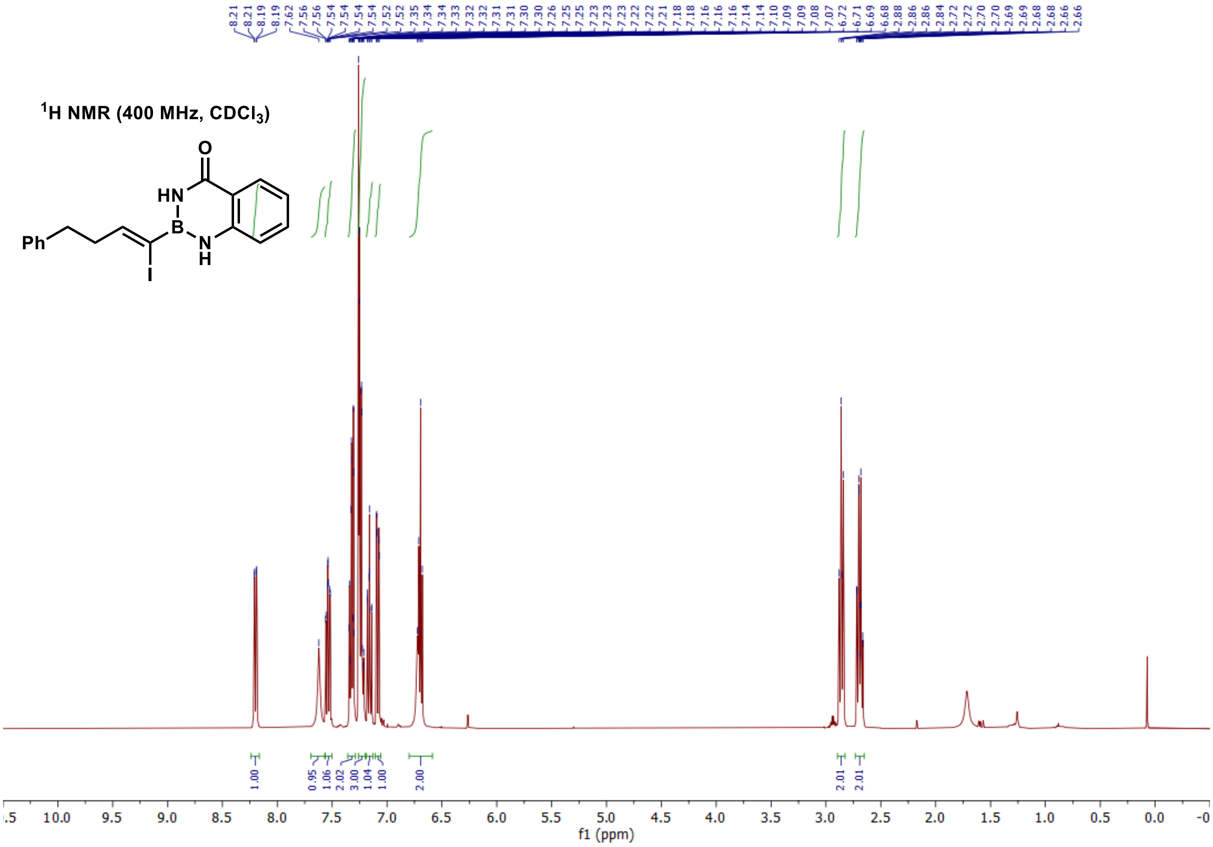
1H NMR (400 MHz, CDCl3)
8.21 8.21 8.19 8.19 7.62 7.56 7.56 7.54 7.54 7.54 7.54 7.52 7.52 7.35 7.34 7.34 7.33 7.32 7.32 7.31 7.31 7.30 7.30 7.26 7.25 7.25 7.23 7.23 7.23 7.22 7.22 7.21 7.21 7.18 7.18 7.16 7.16 7.16 7.14 7.14 7.10 7.09 7.09 7.08 7.07 6.72 6.71 6.69 6.68 2.88 2.86 2.86 2.84 2.72 2.72 2.70 2.70 2.69 2.69 2.68 2.68 2.66 2.66
1.00 0.95 1.06 2.02 3.00 1.04 1.00 2.00 2.01 2.01
f1 (ppm)

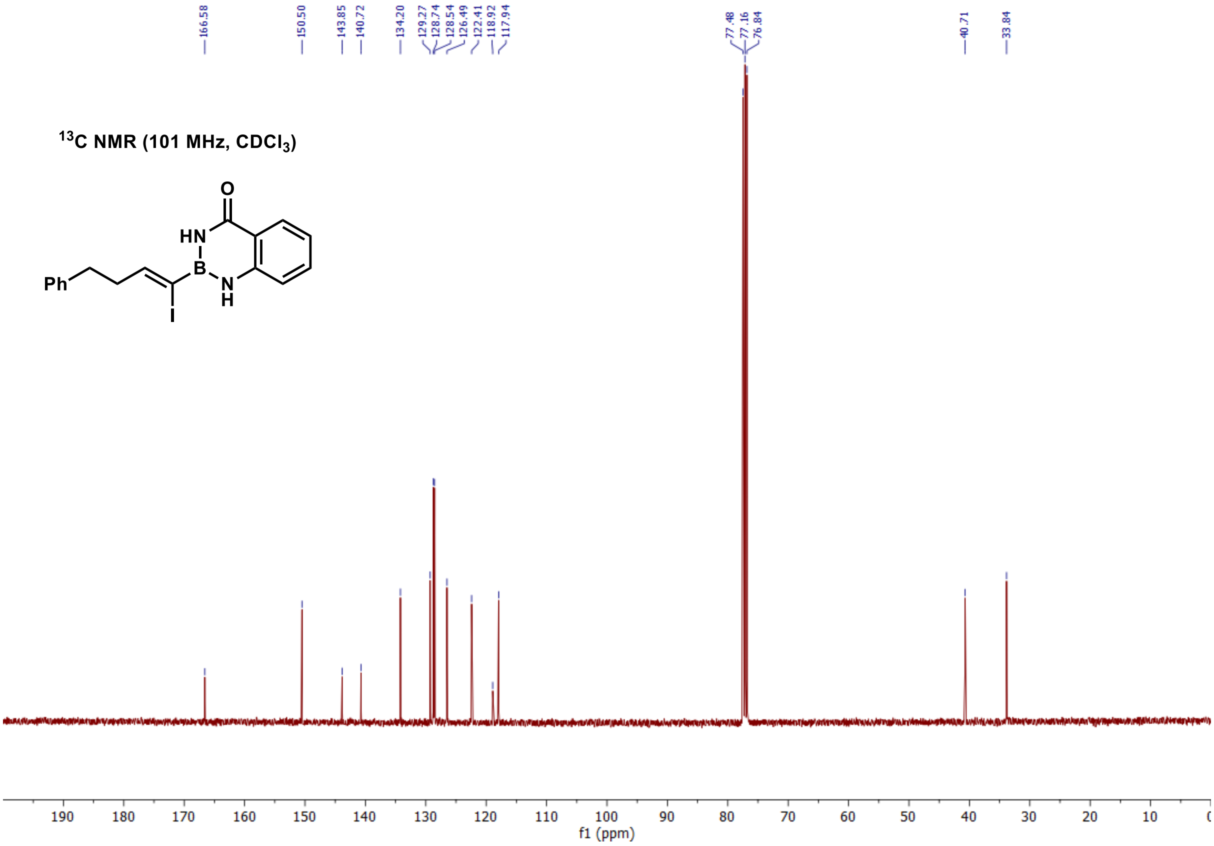
13C NMR (101 MHz, CDCl3)
166.58 150.50 143.85 140.72 134.20 129.27 128.74 128.54 126.49 122.41 118.92 117.94 77.48 77.16 76.84 40.71 33.84
f1 (ppm)

^{11}B NMR (128 MHz, $CDCl_3$)

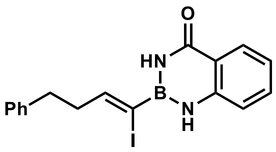

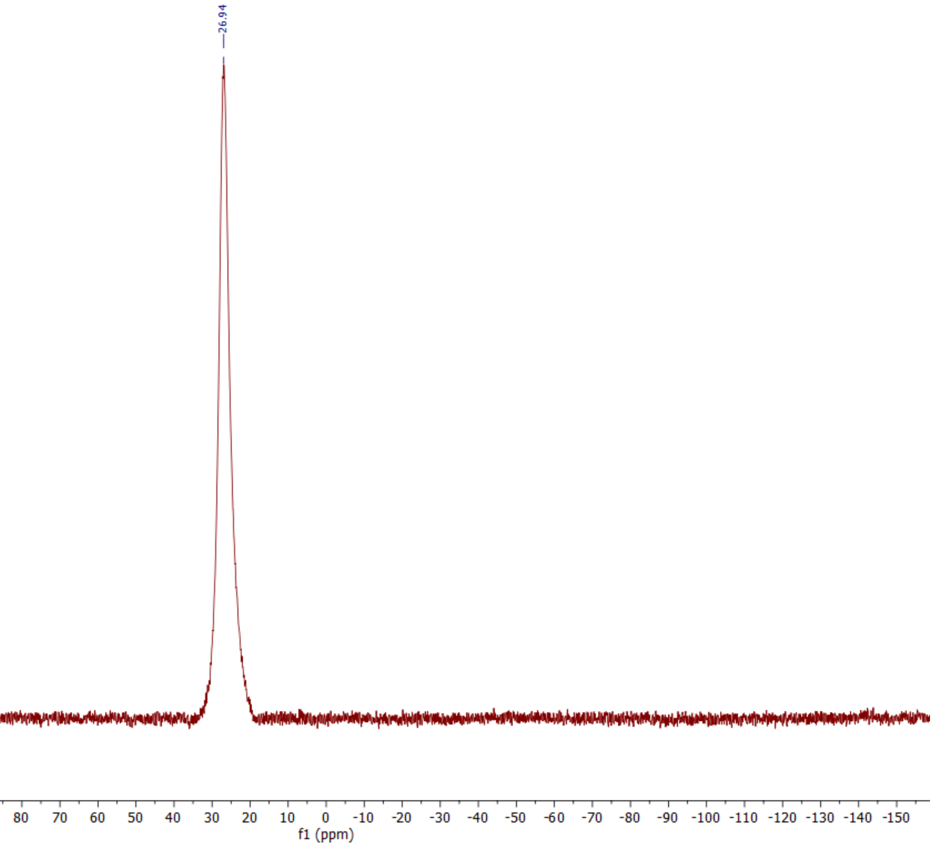

¹H NMR (400 MHz, $CDCl_3$)

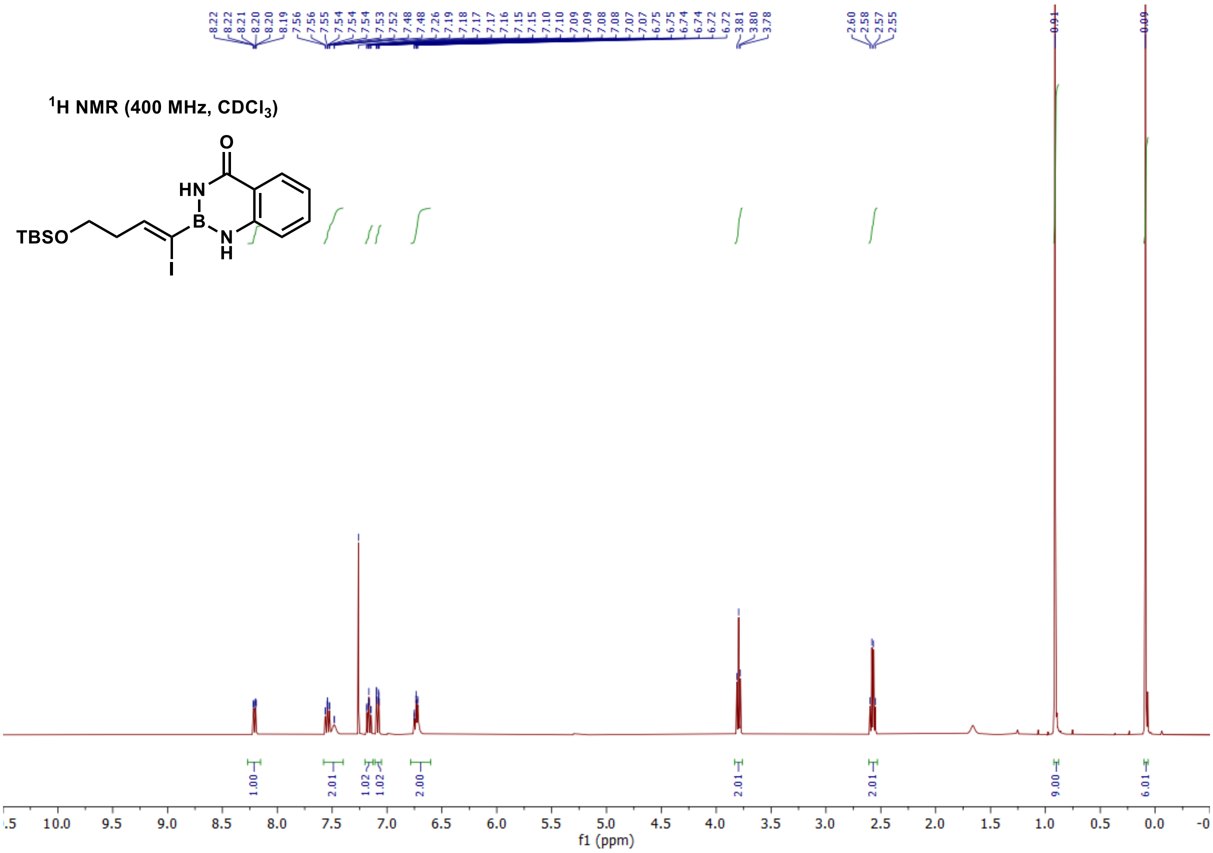

¹³C NMR (101 MHz, $CDCl_3$)

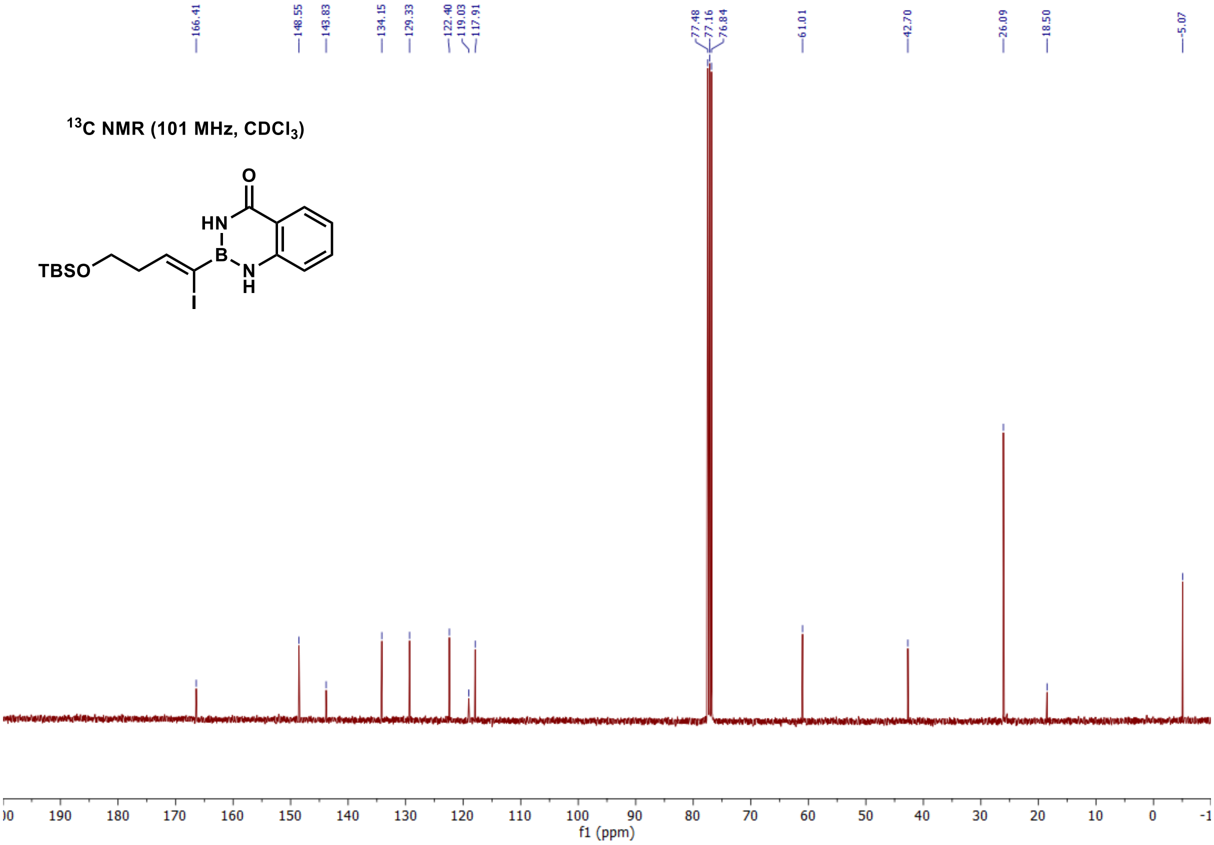

^{11}B NMR (128 MHz, $CDCl_3$)

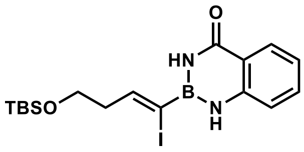

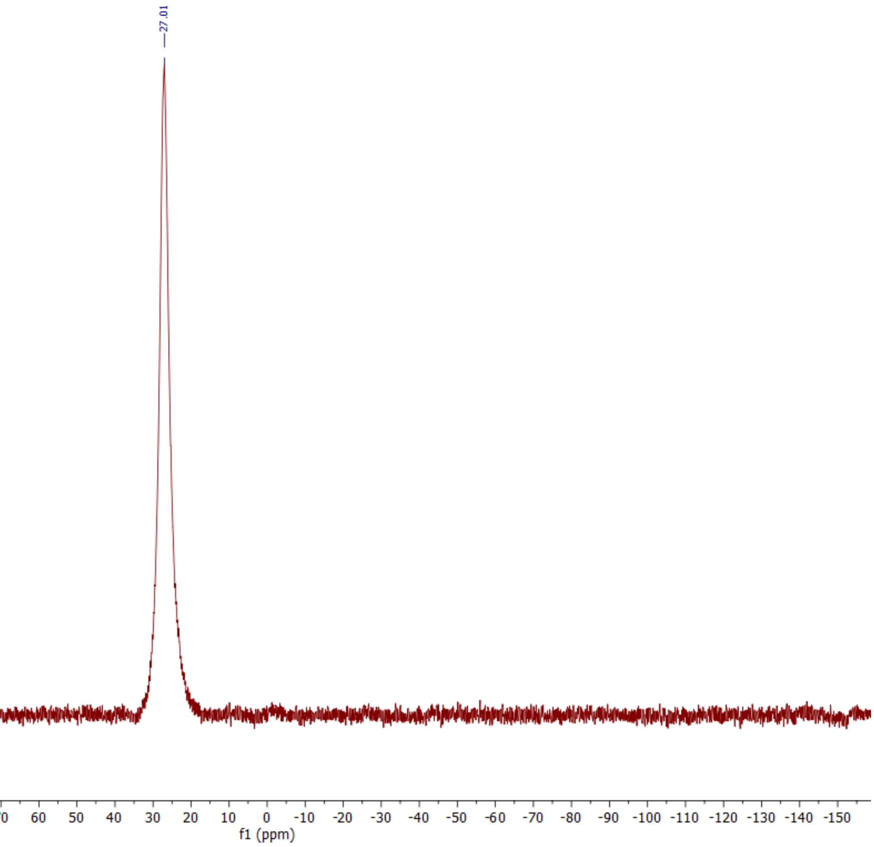

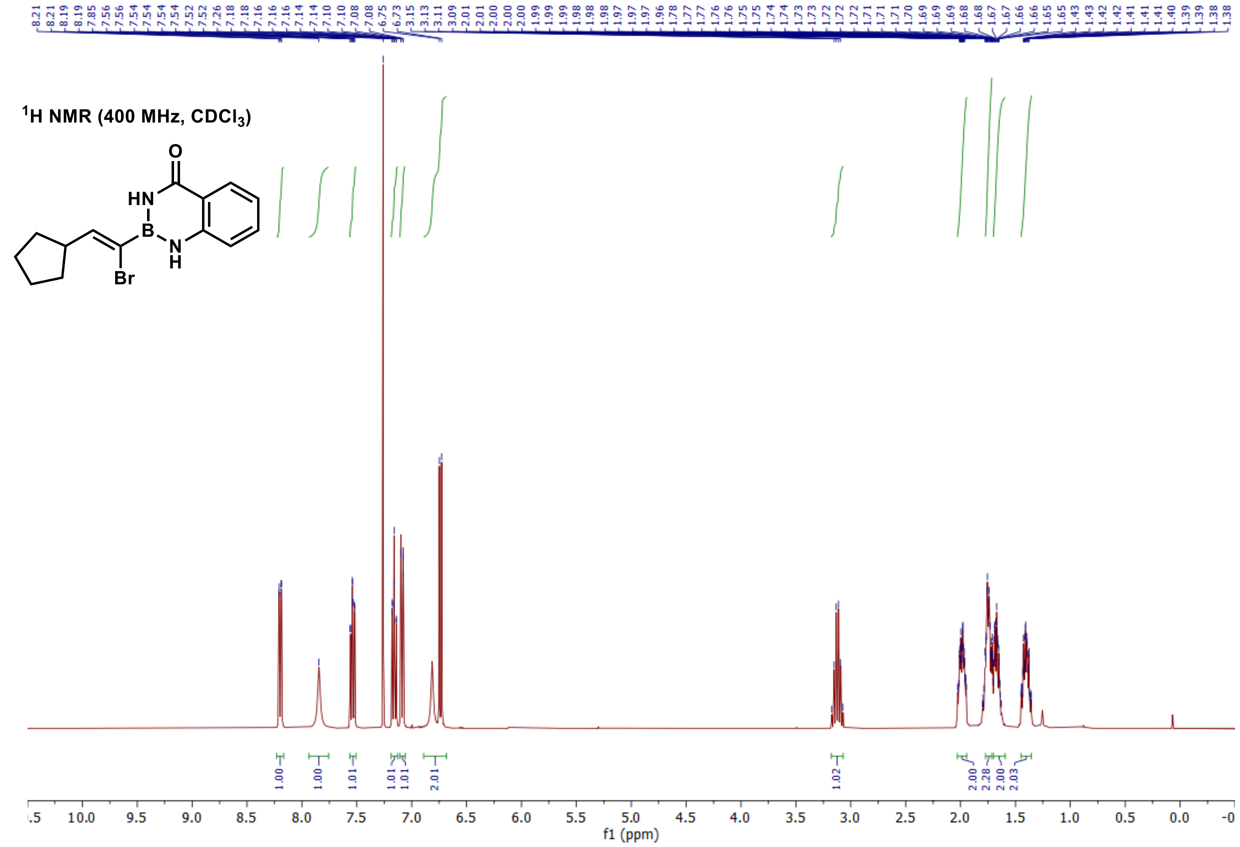
1H NMR (400 MHz, CDCl3)
O
HN
B
N
H
Br
f1 (ppm)

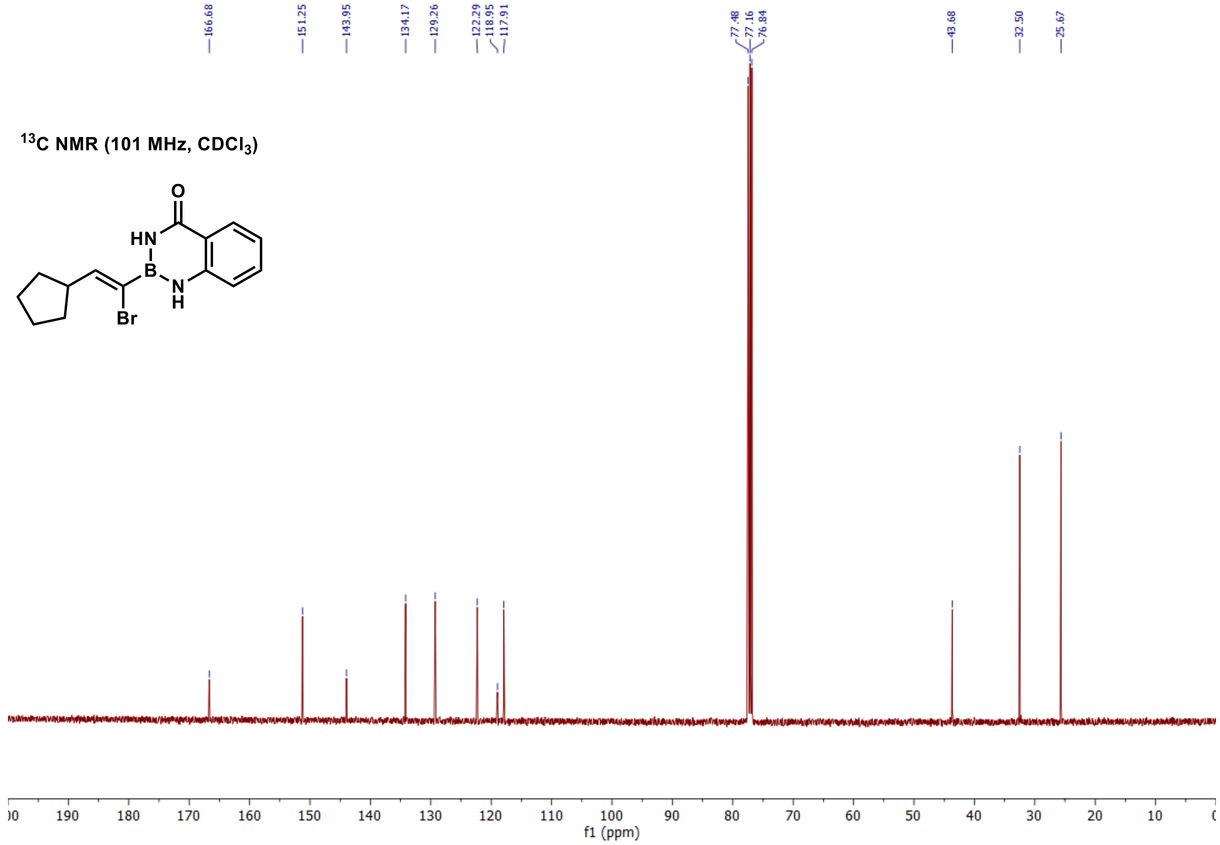
13C NMR (101 MHz, CDCl3)
166.68
151.25
143.95
134.17
129.26
122.29
118.95
117.91
77.48
77.16
76.84
43.68
32.50
25.67
O
HN
B
N
H
Br
f1 (ppm)

^{11}B NMR (128 MHz, $CDCl_3$)

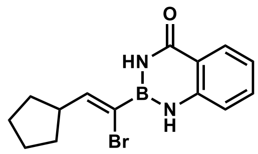

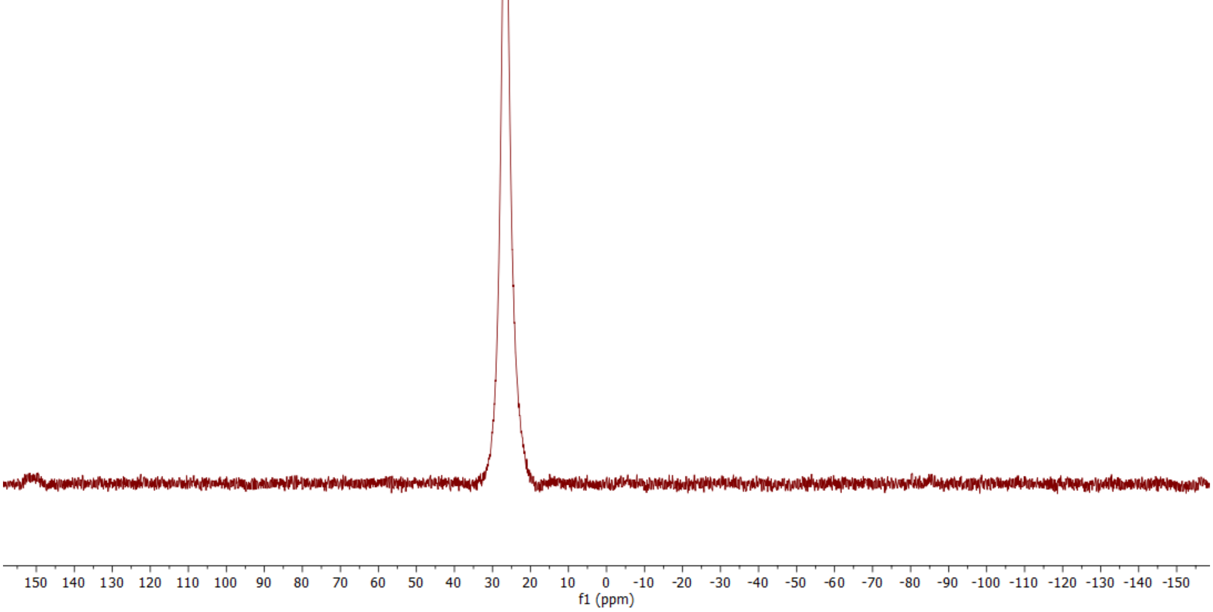

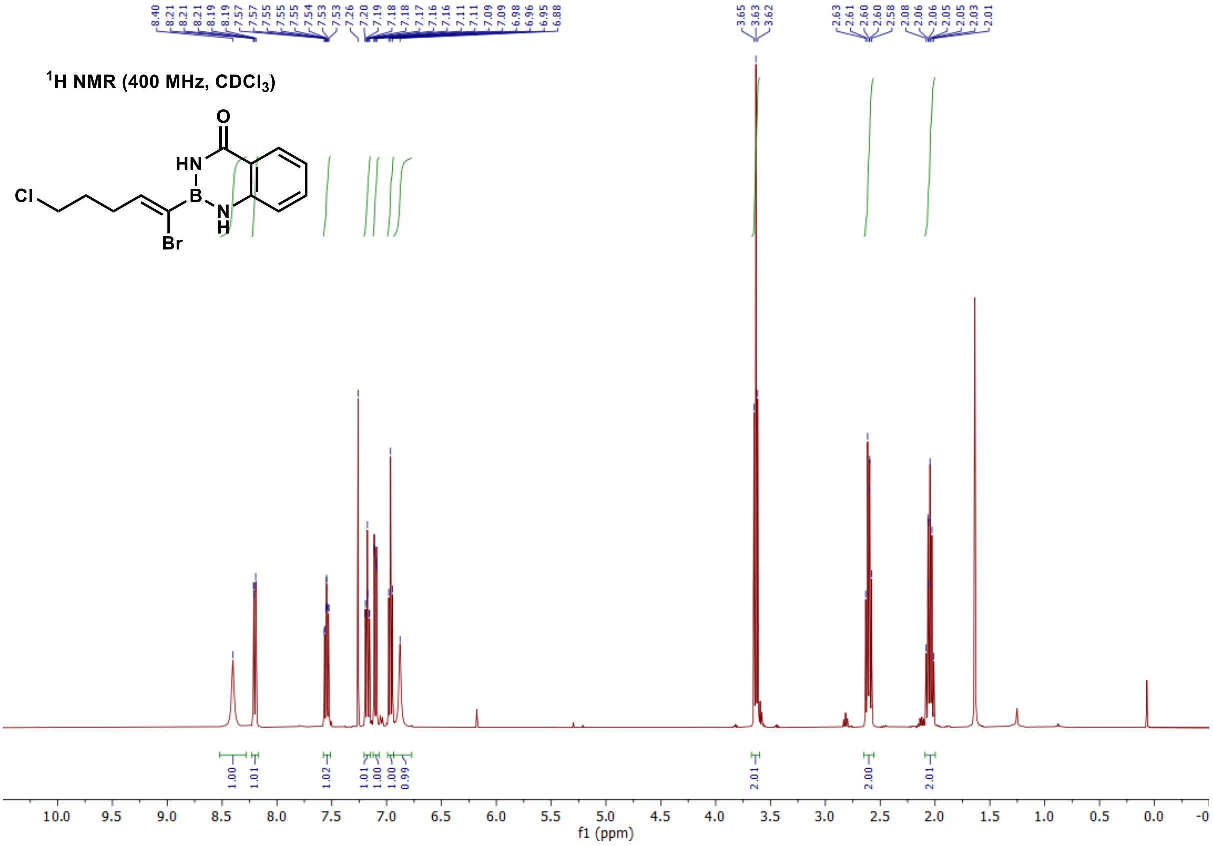

¹H NMR (400 MHz, CDCl₃)
f1 (ppm)

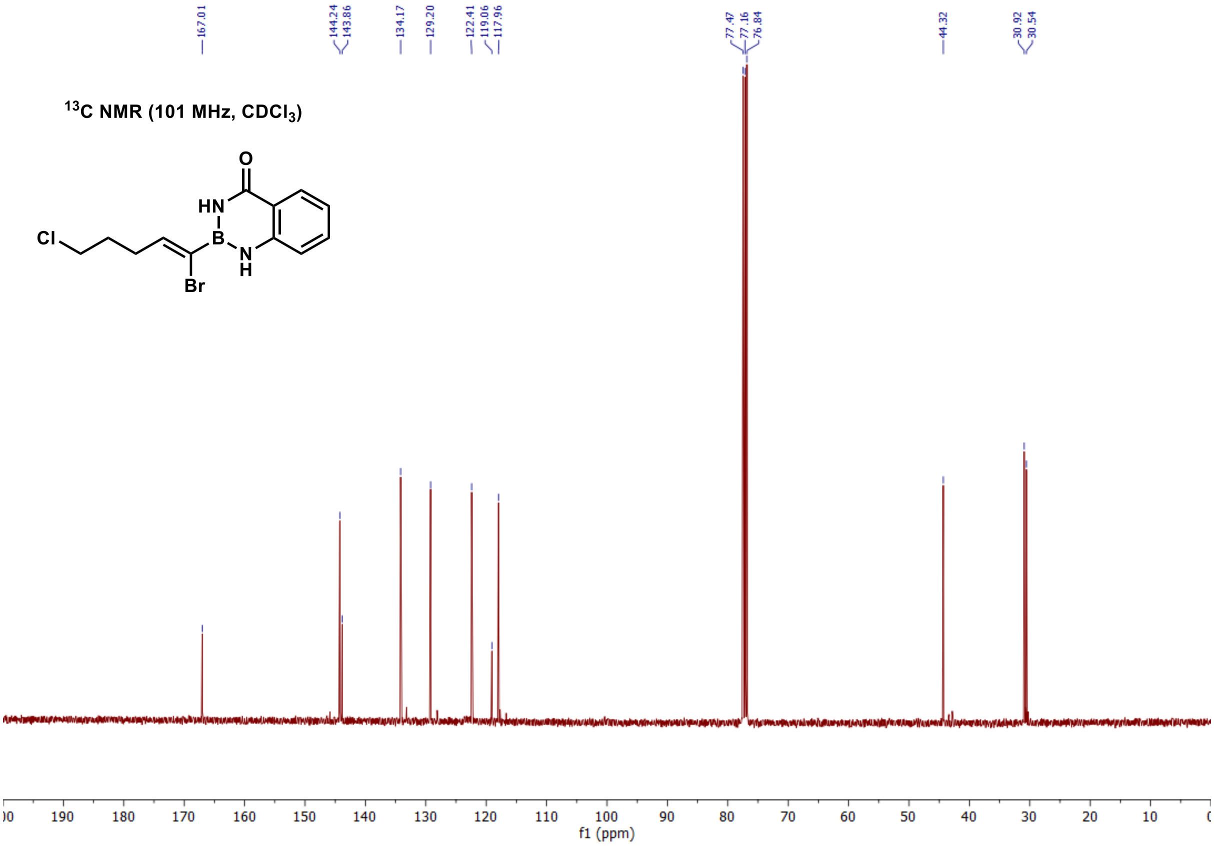

¹³C NMR (101 MHz, CDCl₃)
f1 (ppm)

^{11}B NMR (128 MHz, $CDCl_3$)

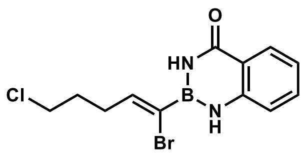

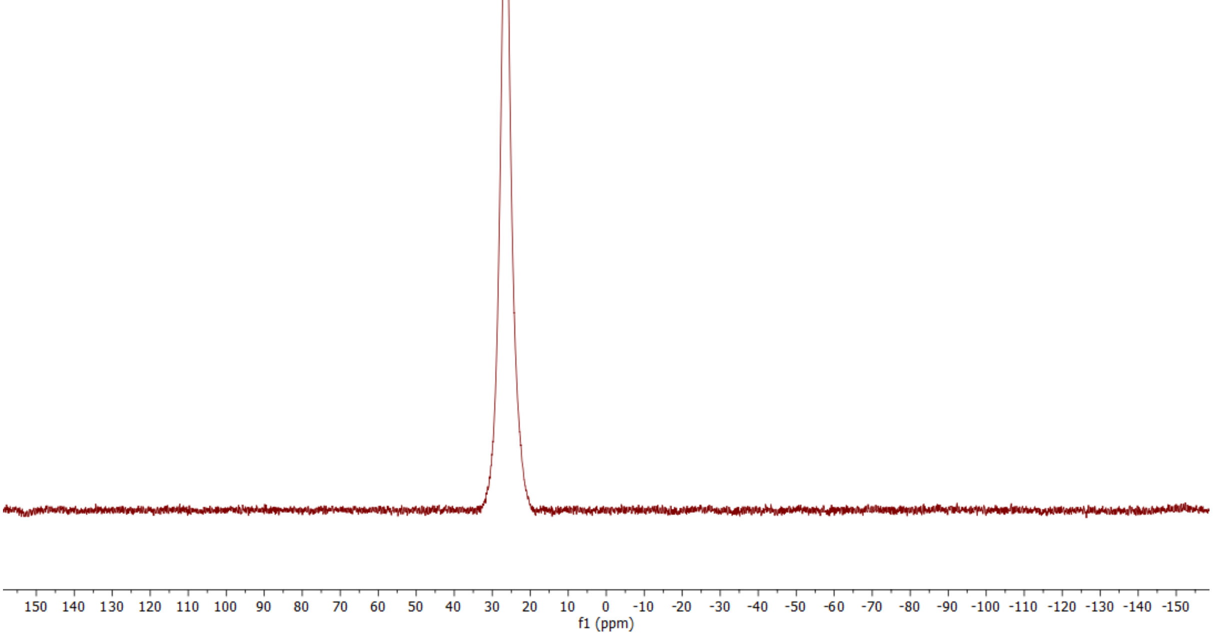

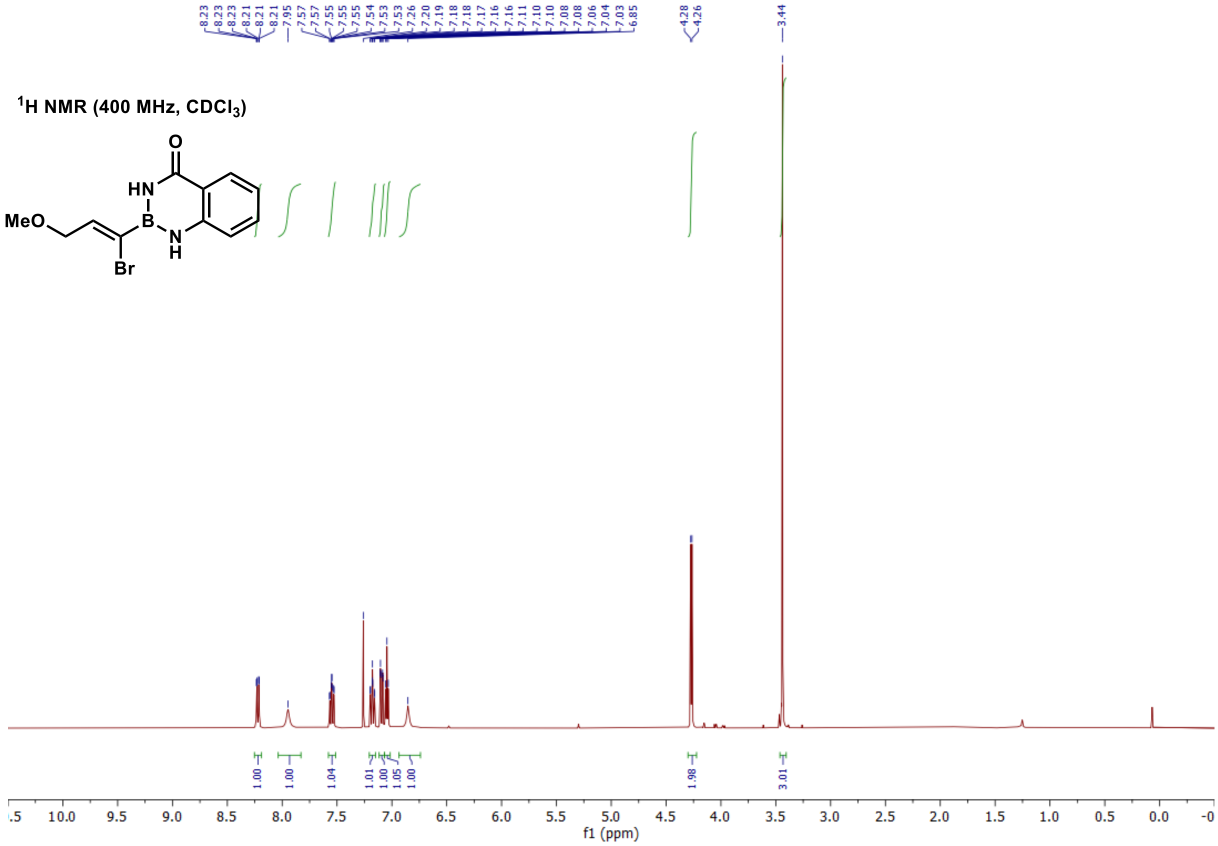
1H NMR (400 MHz, CDCl3)
8.23 8.23 8.23 8.21 8.21 8.21 7.95 7.57 7.57 7.55 7.55 7.55 7.54 7.53 7.53 7.26 7.20 7.19 7.18 7.18 7.17 7.16 7.16 7.11 7.10 7.10 7.08 7.08 7.06 7.04 7.03 6.85
4.28 4.26
3.44
1.00 1.00 1.04 1.01 1.00 1.05 1.00 1.98 3.01
f1 (ppm)

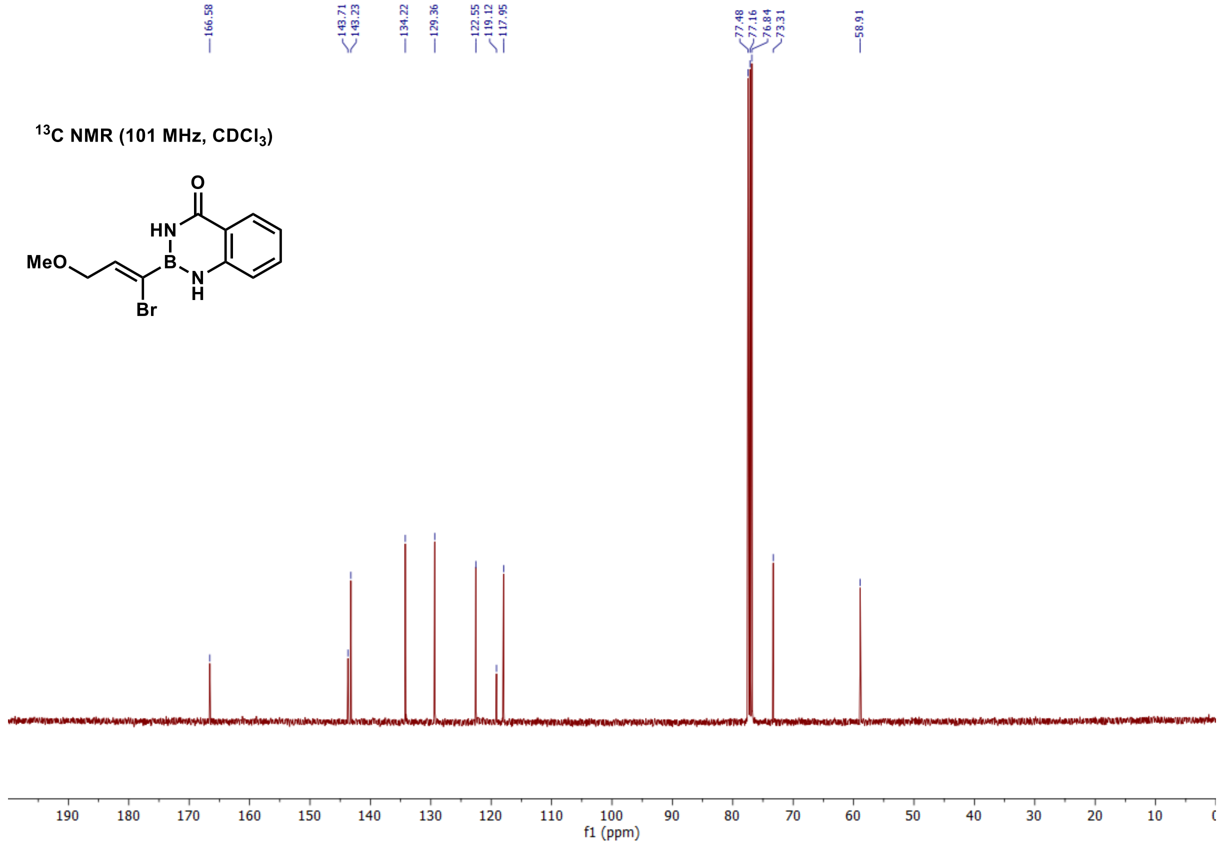
13C NMR (101 MHz, CDCl3)
166.58
143.71 143.23
134.22
129.36
122.55 119.12 117.95
77.48 77.16 76.84 73.31
58.91
f1 (ppm)

^{11}B NMR (128 MHz, $CDCl_3$)

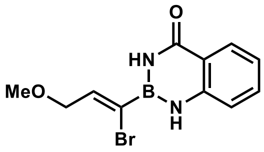

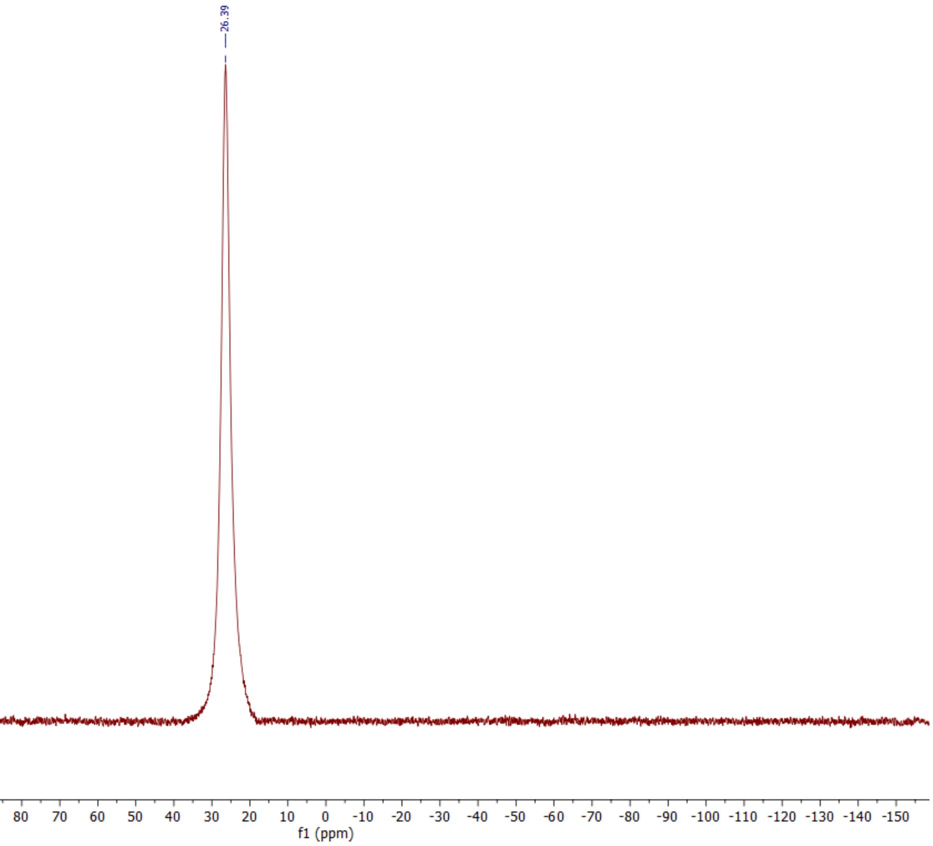

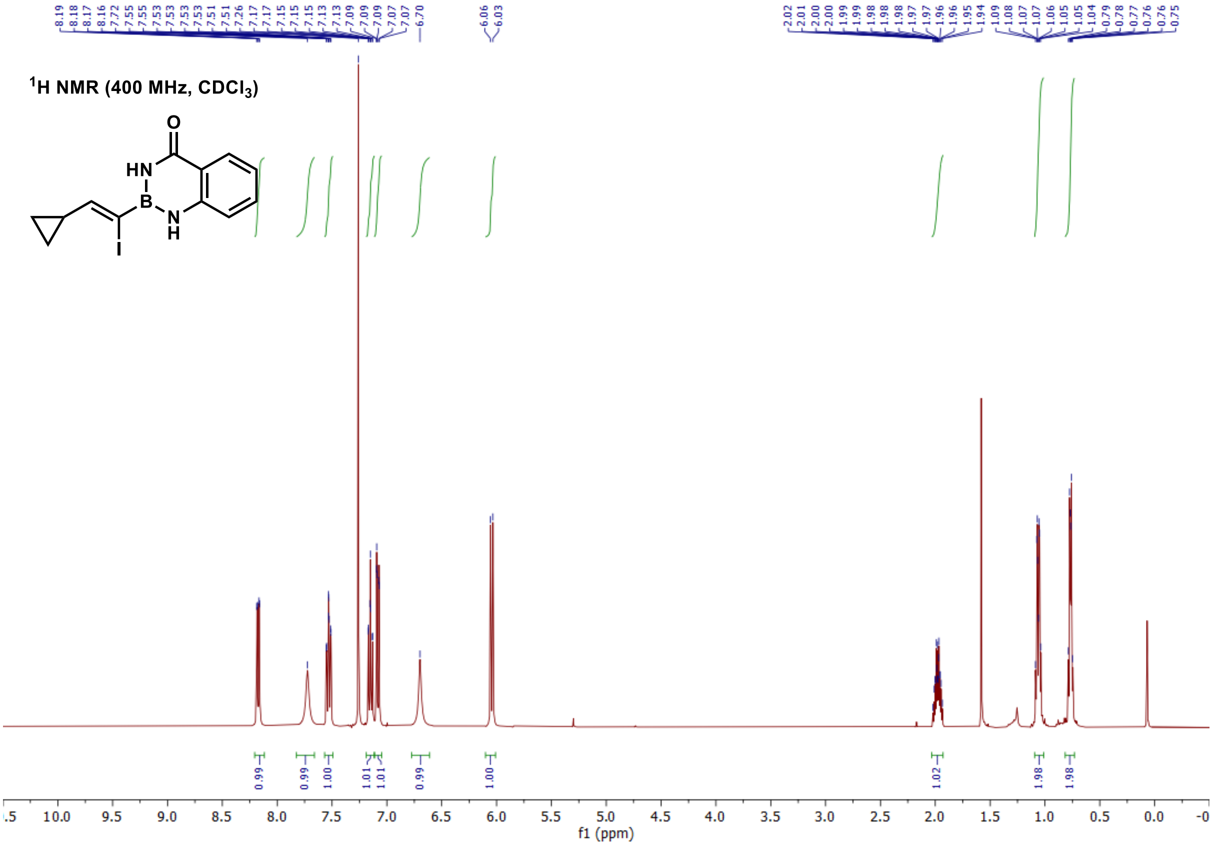
1H NMR (400 MHz, CDCl3)
HN
B
N
H
O
I
f1 (ppm)

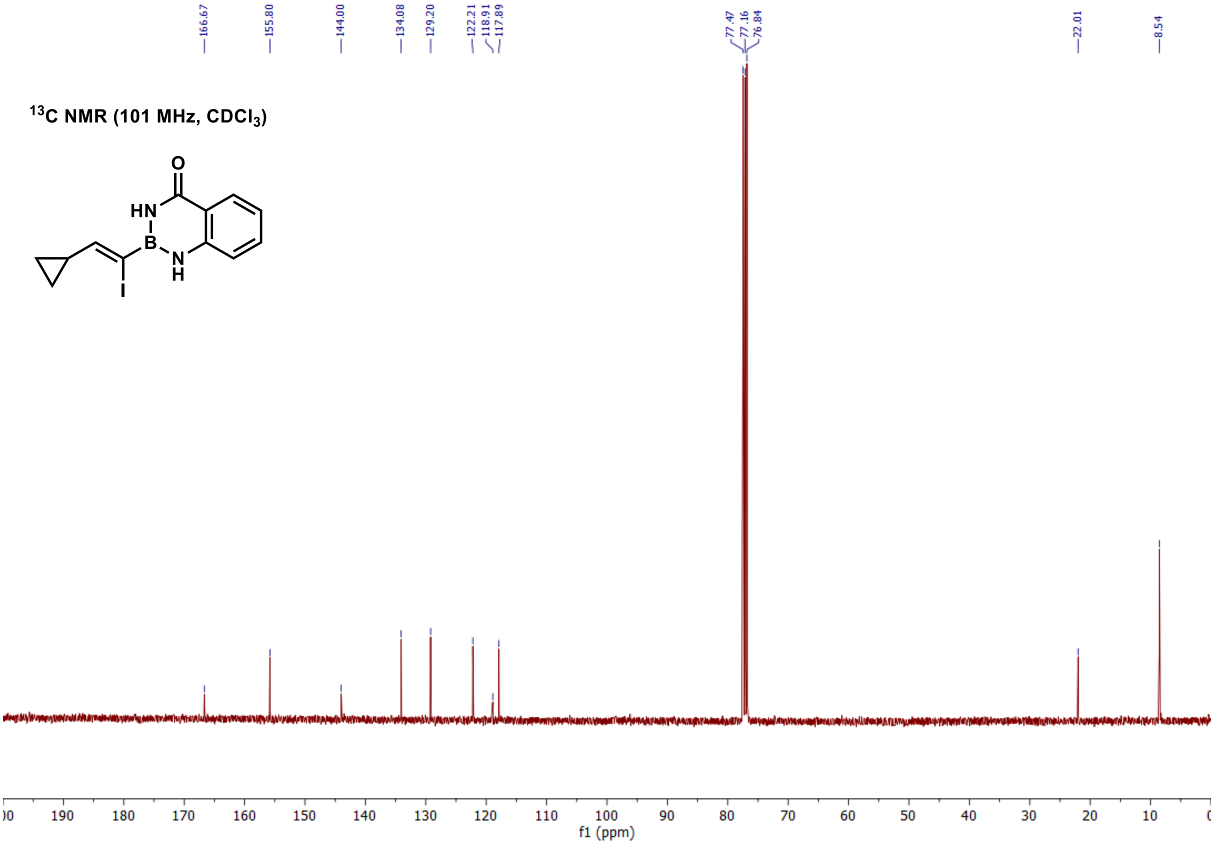
13C NMR (101 MHz, CDCl3)
166.67
155.80
144.00
134.08
129.20
122.21
118.91
117.89
77.47
77.16
76.84
22.01
8.54
HN
B
N
H
O
I
f1 (ppm)

^{11}B NMR (128 MHz, $CDCl_3$)

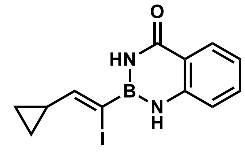

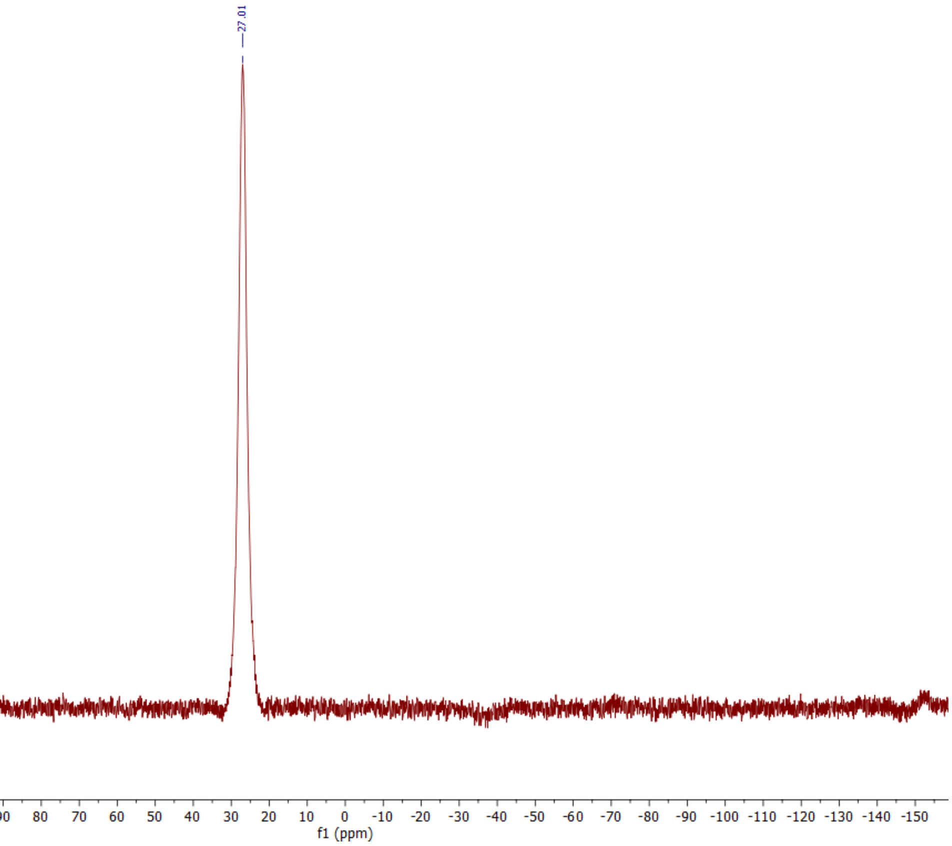

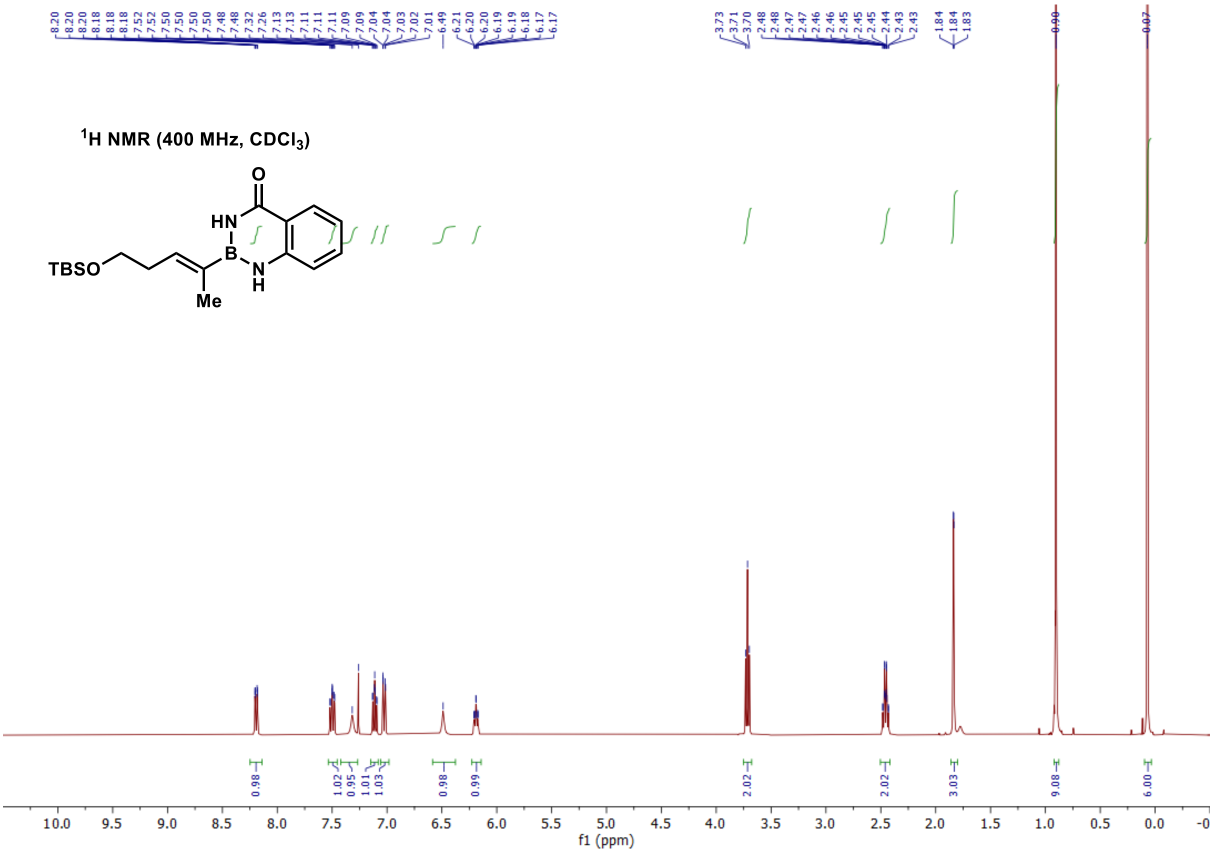

1H NMR (400 MHz, CDCl3)
TBSO
HN
B
N
H
O
Me
f1 (ppm)

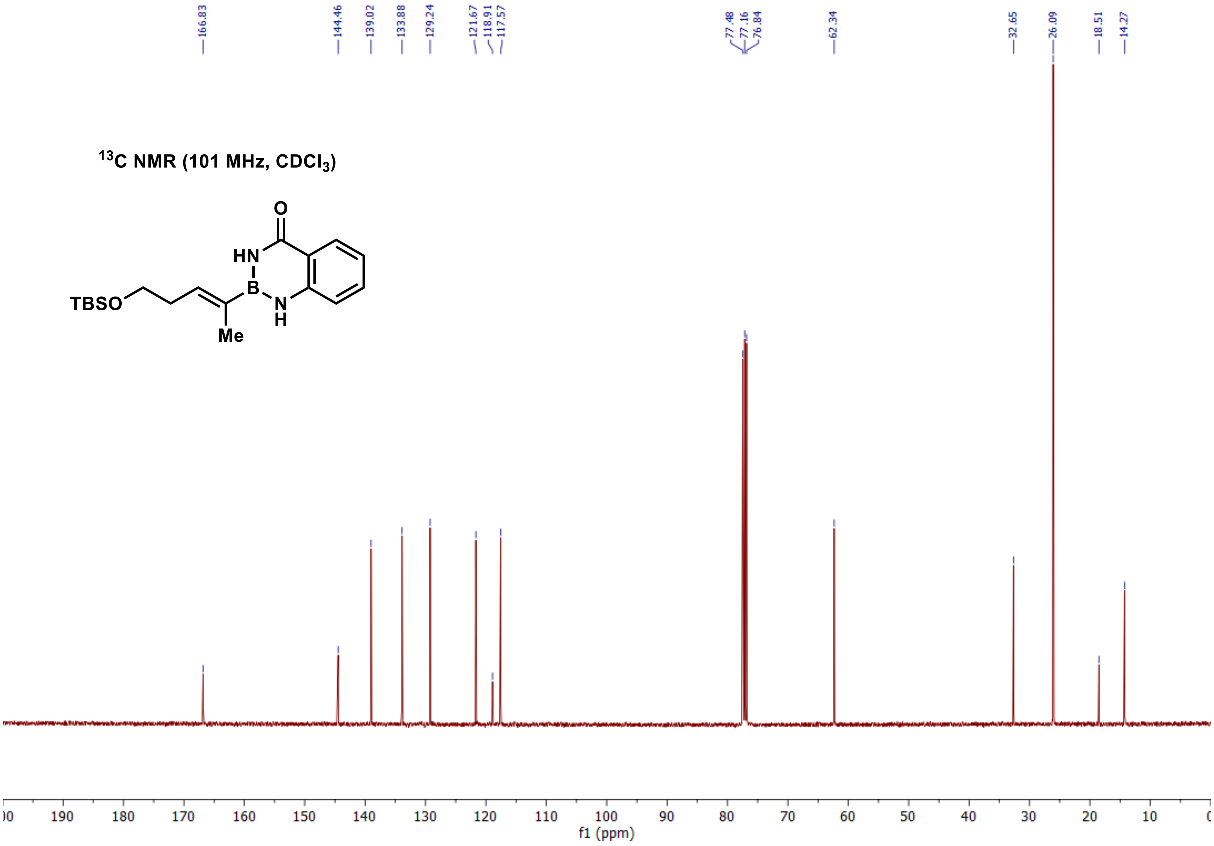

13C NMR (101 MHz, CDCl3)
TBSO
HN
B
N
H
O
Me
166.83
144.46
139.02
133.88
129.24
121.67
118.91
117.57
77.48
77.16
76.84
62.34
32.65
26.09
18.51
14.27
f1 (ppm)

^{11}B NMR (128 MHz, $CDCl_3$)

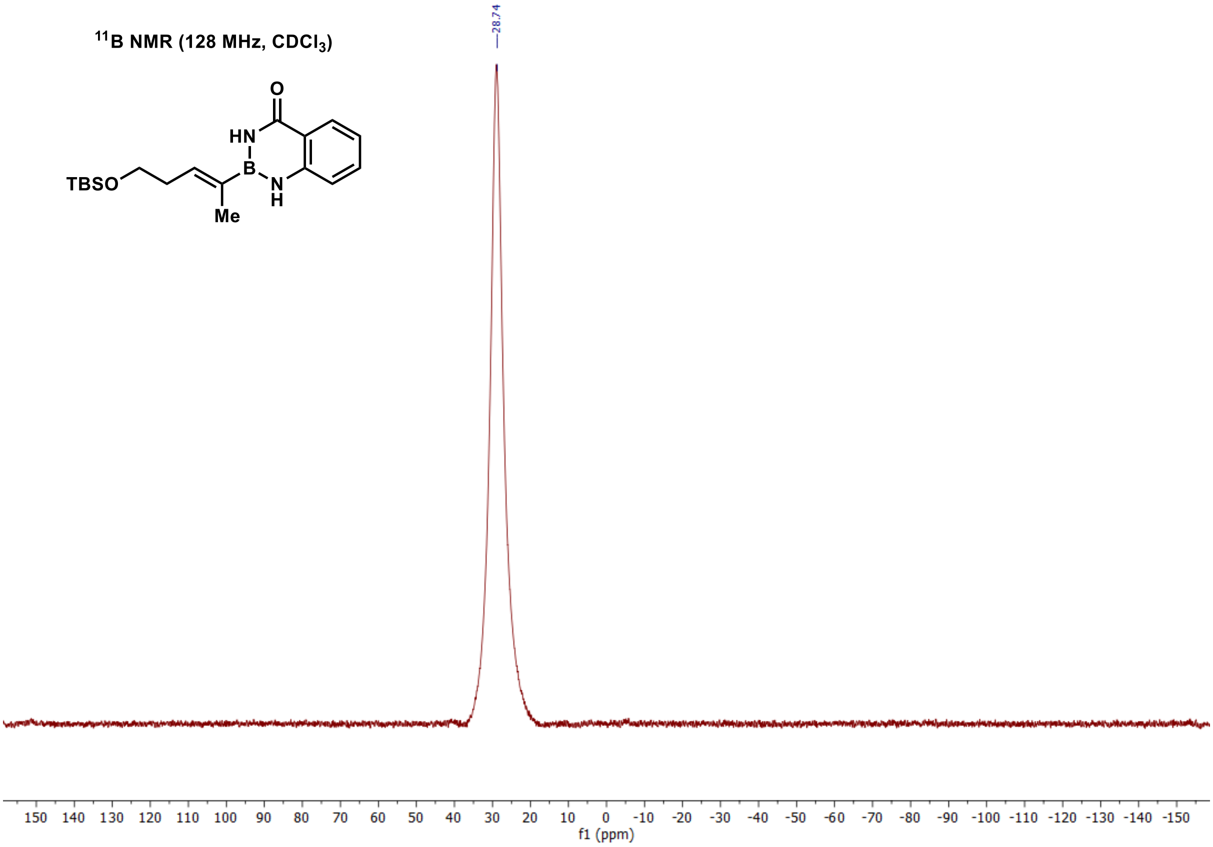

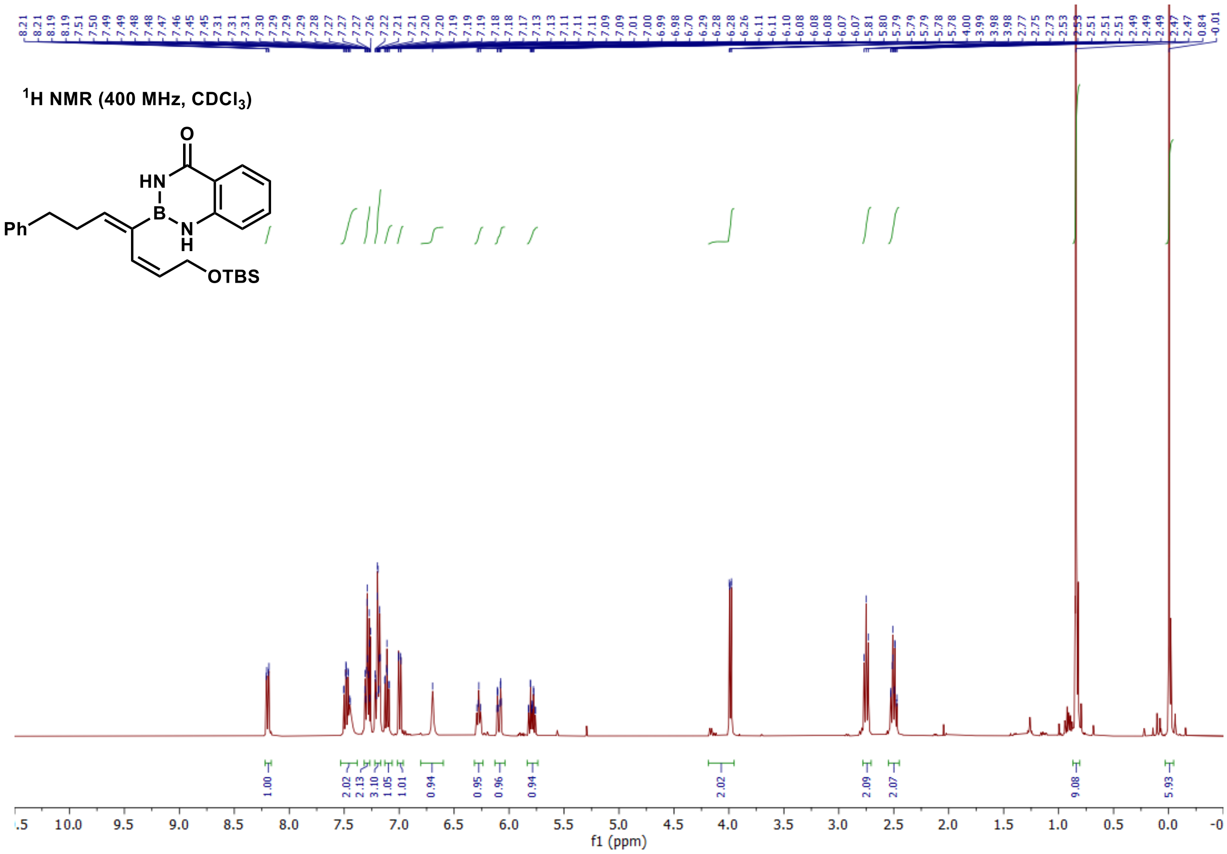
1H NMR (400 MHz, CDCl3)
Ph
OTBS
f1 (ppm)

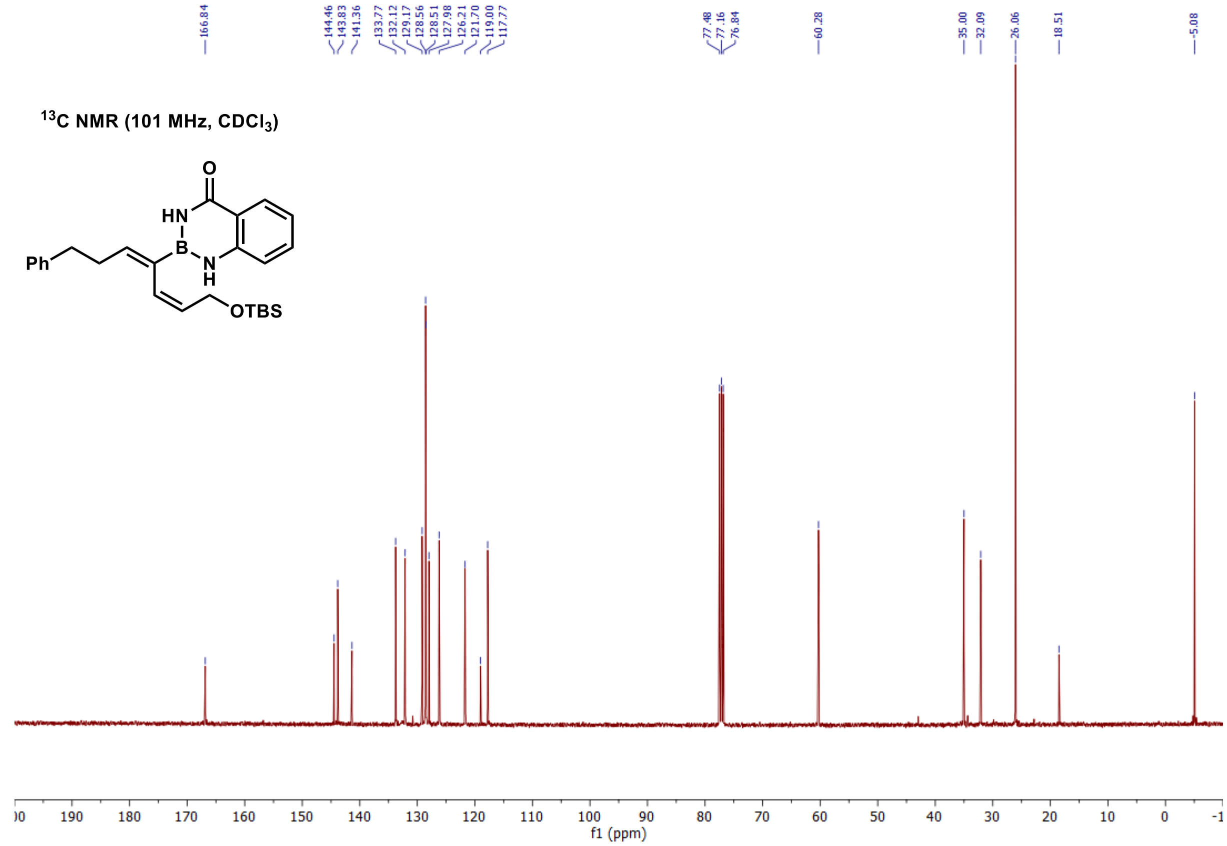
13C NMR (101 MHz, CDCl3)
Ph
OTBS
f1 (ppm)

^{11}B NMR (128 MHz, $CDCl_3$)

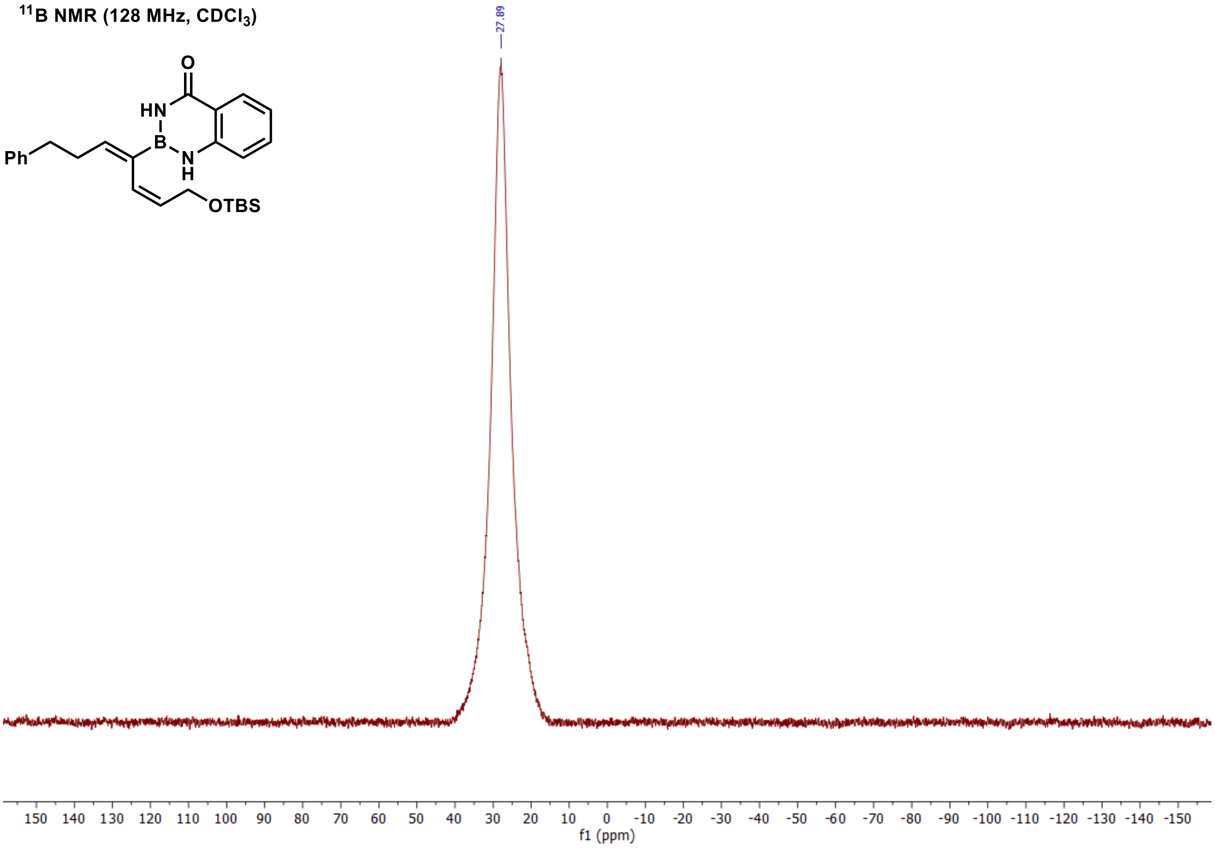

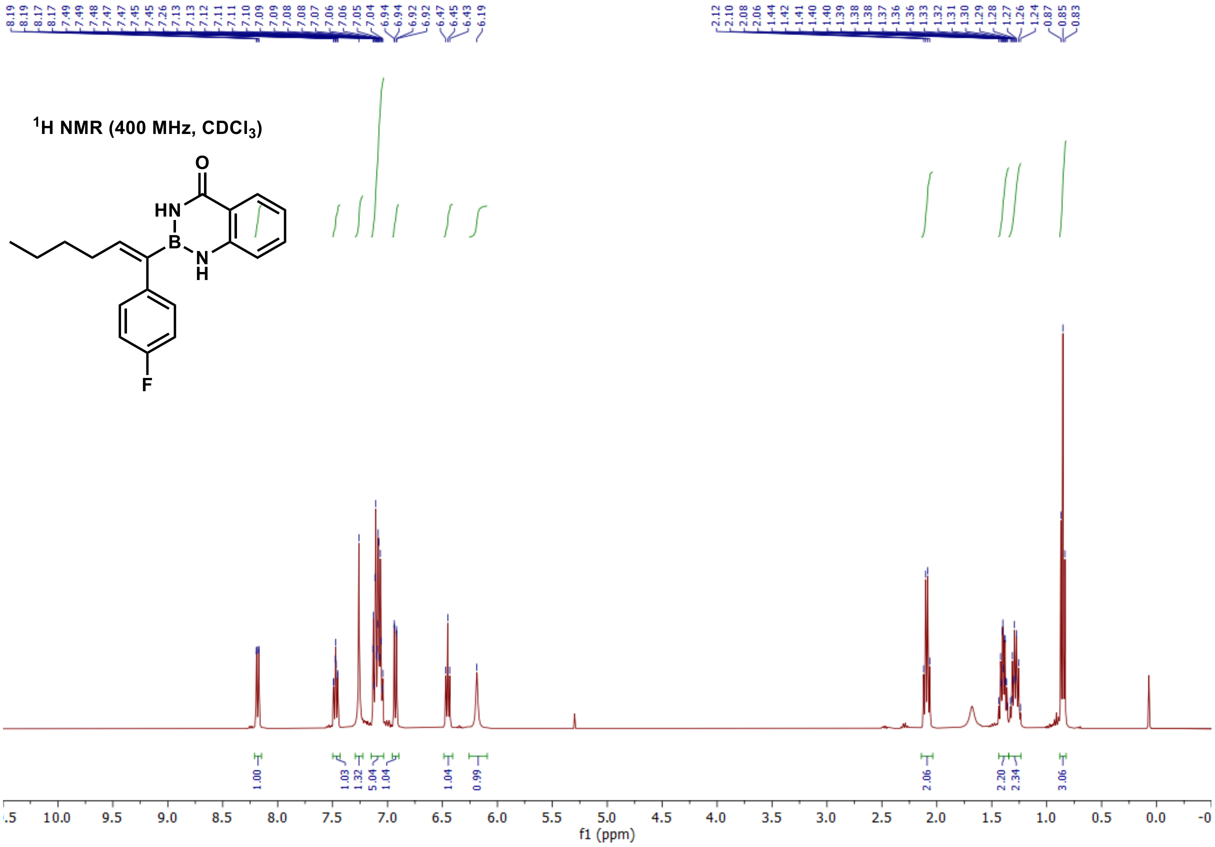
8.19 8.19 8.17 8.17 7.49 7.49 7.48 7.47 7.47 7.45 7.45 7.26 7.13 7.13 7.12 7.11 7.11 7.10 7.09 7.09 7.08 7.08 7.07 7.06 7.06 7.05 7.04 6.94 6.94 6.92 6.47 6.45 6.43 6.19
2.12 2.10 2.08 2.06 1.44 1.42 1.41 1.40 1.40 1.39 1.38 1.38 1.37 1.37 1.36 1.36 1.33 1.32 1.31 1.30 1.29 1.28 1.27 1.26 1.24 0.87 0.85 0.83
1H NMR (400 MHz, CDCl3)
O
HN
B
N
H
F
1.00
1.03
1.32
5.04
1.04
1.04
0.99
2.06
2.20
2.34
3.06
f1 (ppm)

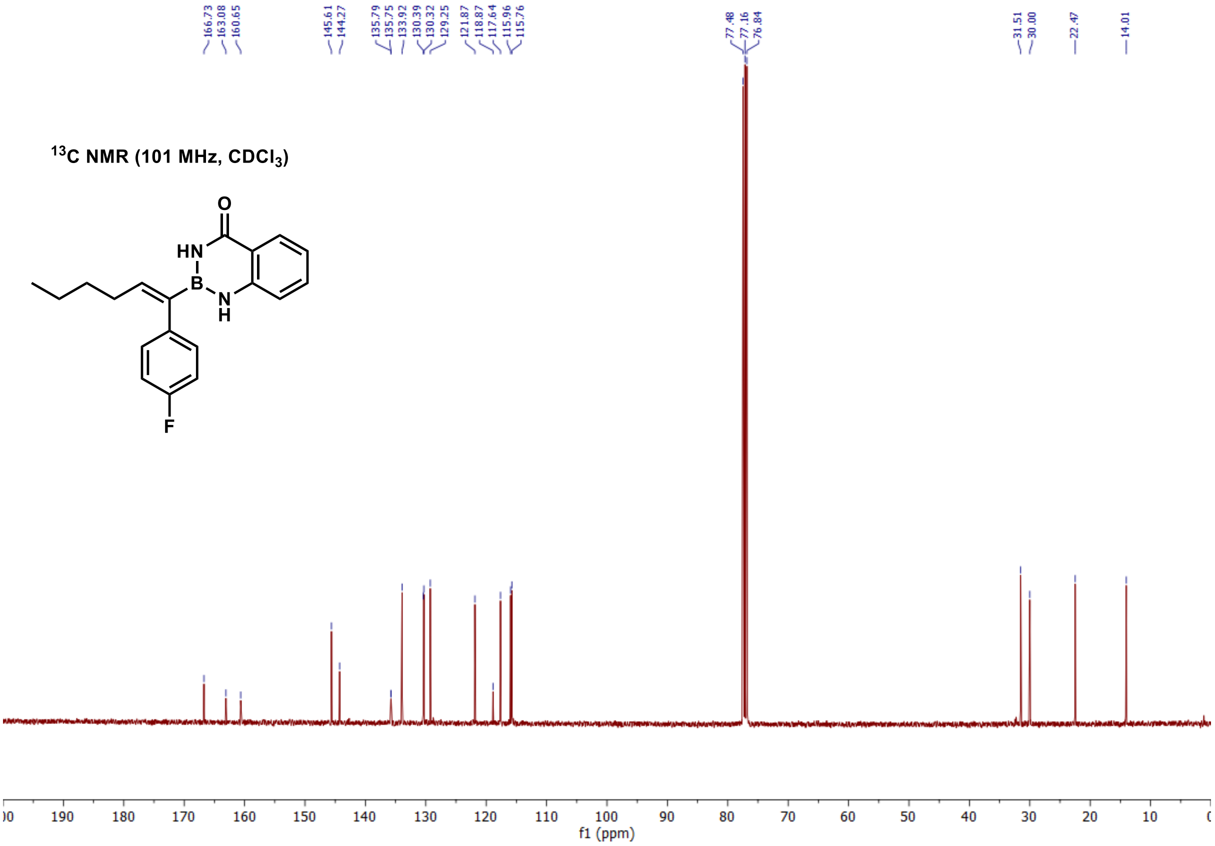
166.73 163.08 160.65 145.61 144.27 135.79 135.75 133.92 130.39 130.32 129.25 121.87 118.87 117.64 115.96 115.76
77.48 77.16 76.84
31.51 30.00 22.47 14.01
13C NMR (101 MHz, CDCl3)
O
HN
B
N
H
F
f1 (ppm)

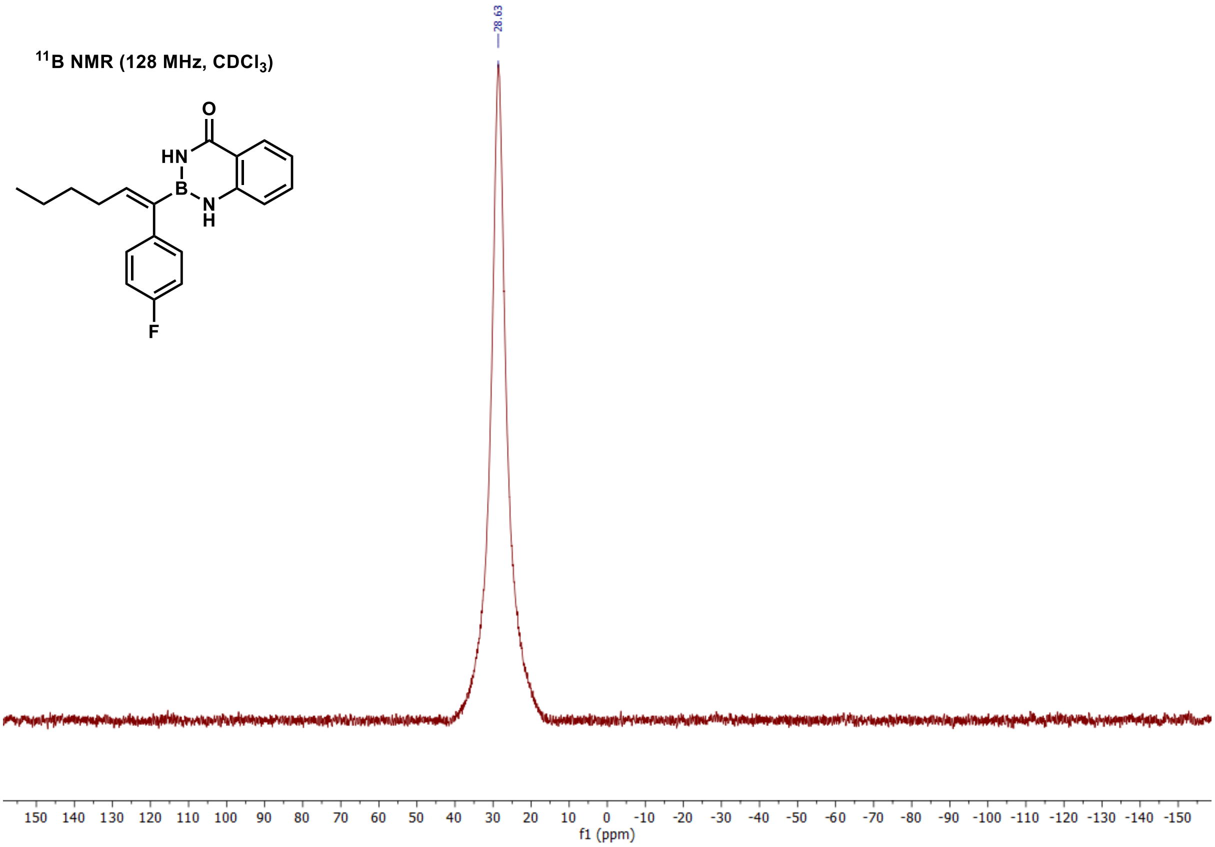

28.63
^{11}B NMR (128 MHz, CDCl$_3$)
O
HN
B
N
H
F
150 140 130 120 110 100 90 80 70 60 50 40 30 20 10 0 -10 -20 -30 -40 -50 -60 -70 -80 -90 -100 -110 -120 -130 -140 -150
f1 (ppm)

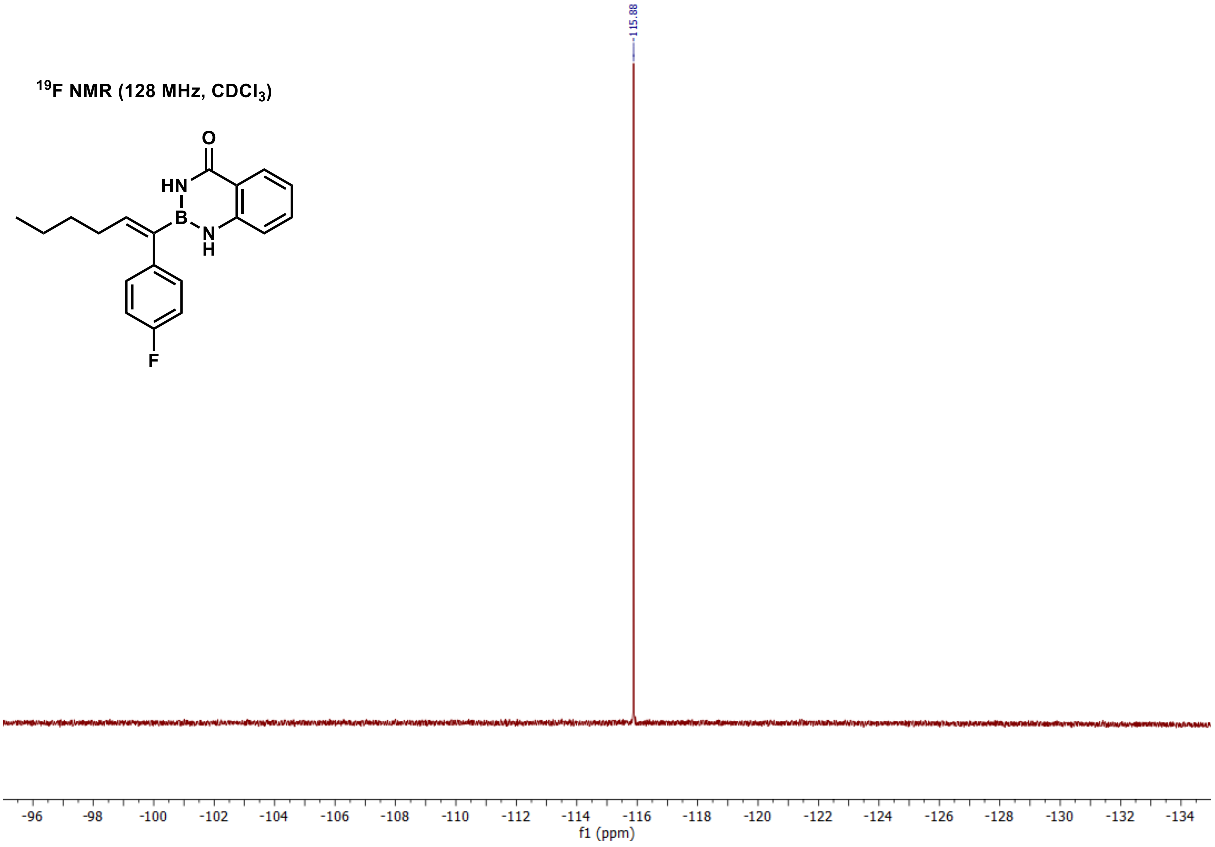

-115.88
^{19}F NMR (128 MHz, CDCl$_3$)
O
HN
B
N
H
F
-96 -98 -100 -102 -104 -106 -108 -110 -112 -114 -116 -118 -120 -122 -124 -126 -128 -130 -132 -134
f1 (ppm)

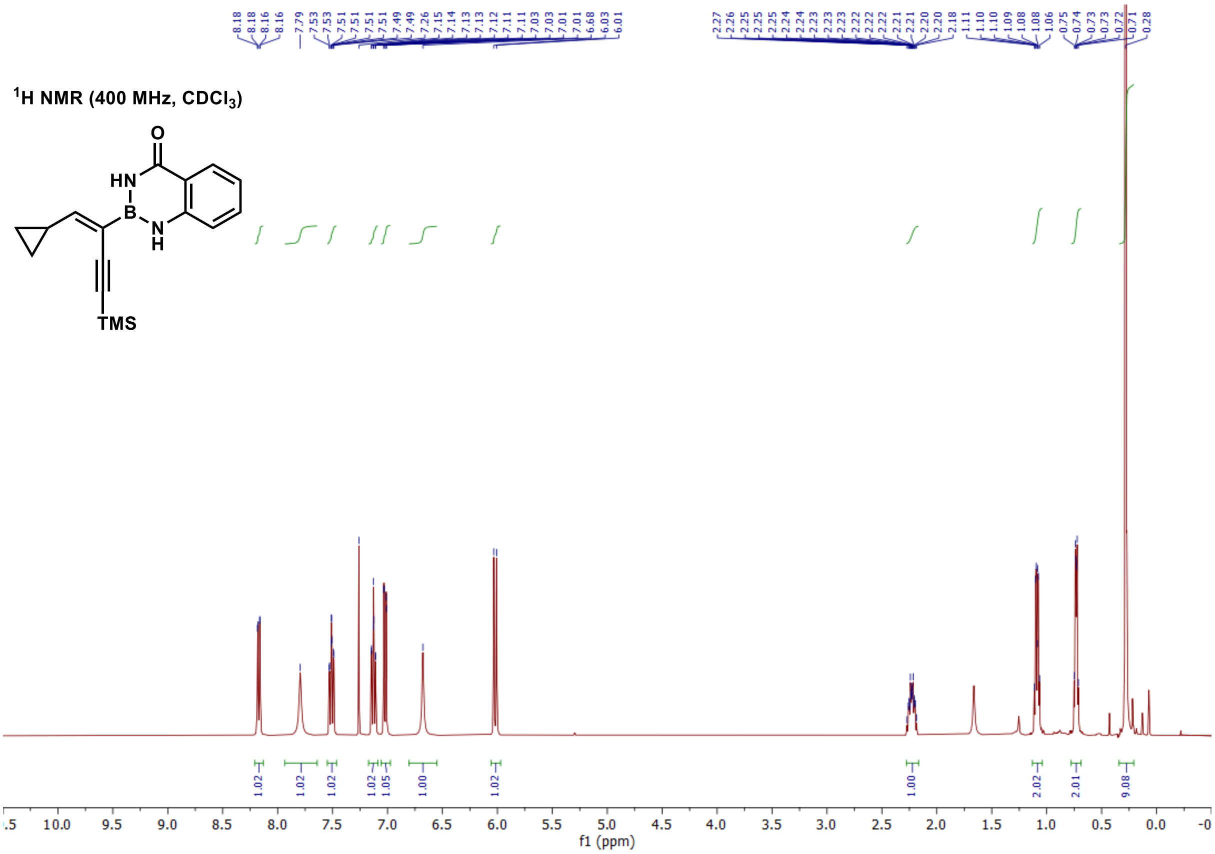
1H NMR (400 MHz, CDCl3)
O
HN
B
N
H
TMS
f1 (ppm)

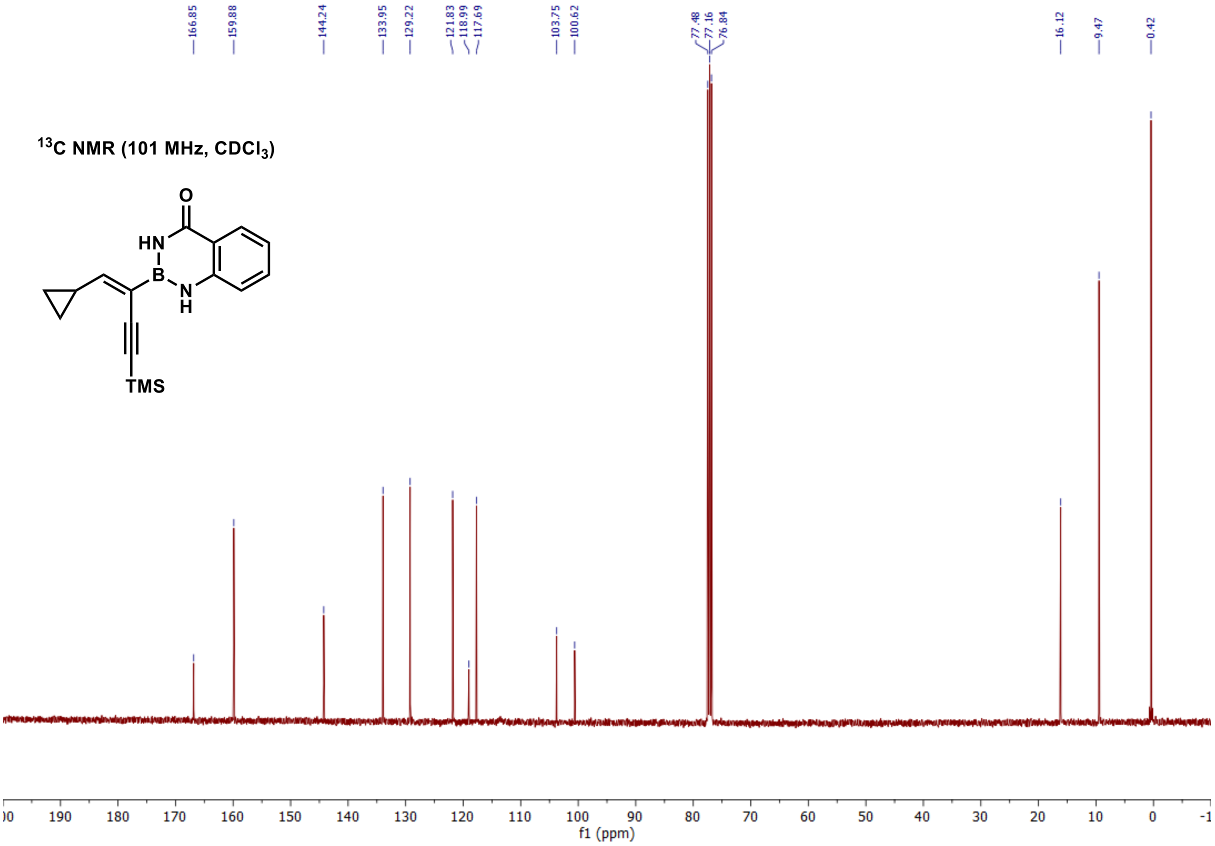
13C NMR (101 MHz, CDCl3)
O
HN
B
N
H
TMS
f1 (ppm)

^{11}B NMR (128 MHz, $CDCl_3$)

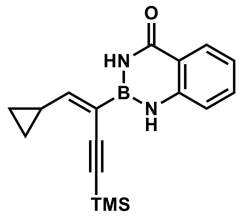

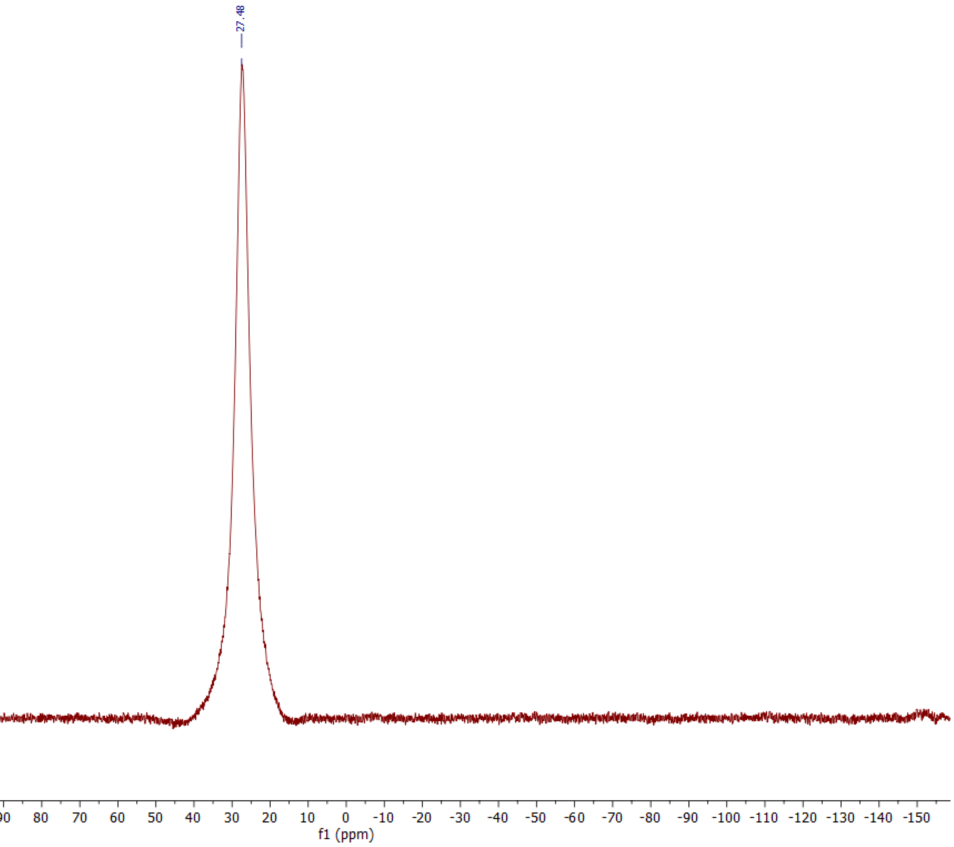

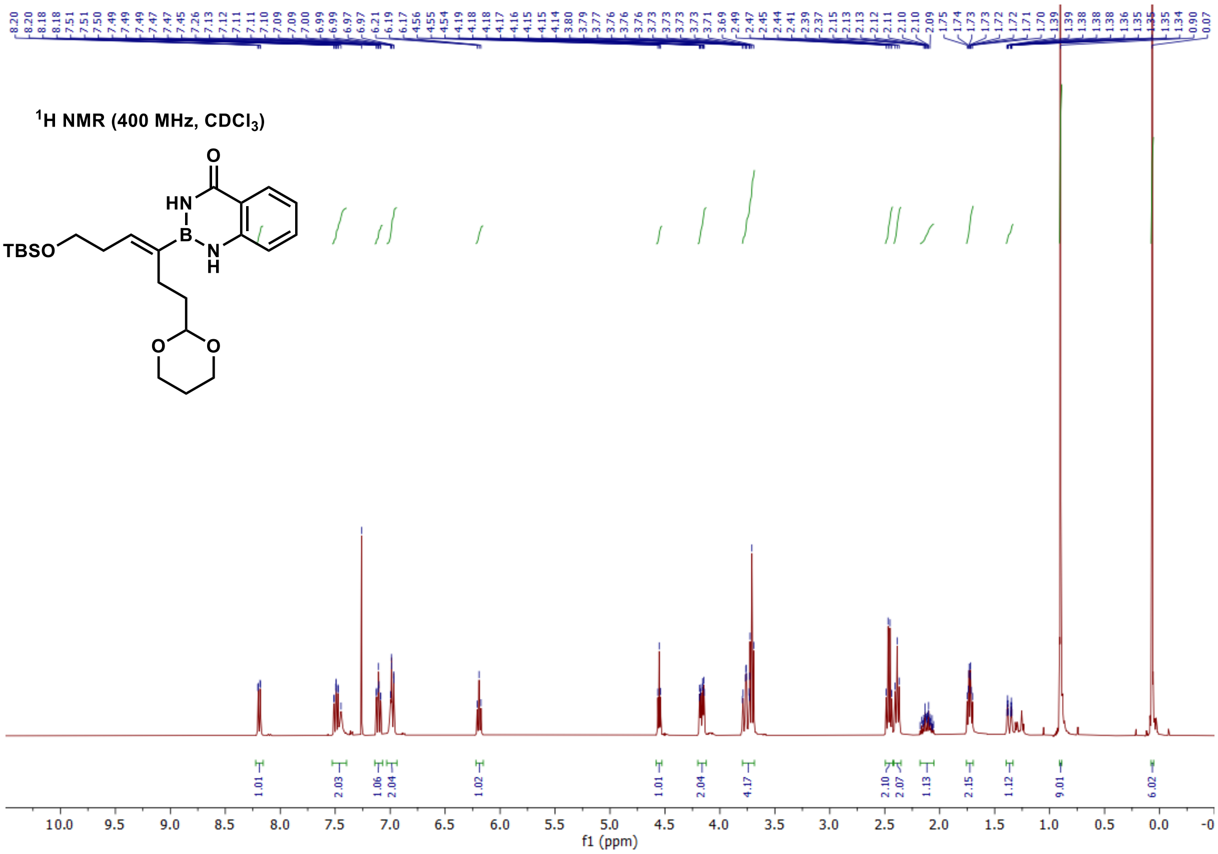

1H NMR (400 MHz, CDCl3)
TBSO
HN
B
N
H
O
8.20 8.20 8.18 8.18 7.51 7.50 7.49 7.49 7.49 7.47 7.47 7.45 7.26 7.13 7.12 7.11 7.11 7.10 7.09 7.09 7.00 6.99 6.99 6.97 6.97 6.21 6.19 6.17 4.56 4.55 4.54 4.19 4.18 4.18 4.17 4.16 4.15 4.15 4.14 3.80 3.79 3.77 3.76 3.76 3.76 3.73 3.73 3.73 3.73 3.71 3.69 2.49 2.47 2.45 2.44 2.41 2.39 2.37 2.15 2.13 2.13 2.12 2.11 2.10 2.10 2.09 1.75 1.74 1.73 1.73 1.72 1.72 1.71 1.70 1.39 1.39 1.38 1.38 1.38 1.36 1.35 1.35 1.35 1.34 0.90 0.07
1.01 2.03 1.06 2.04 1.02 1.01 2.04 4.17 2.10 2.07 1.13 2.15 1.12 9.01 6.02
f1 (ppm)

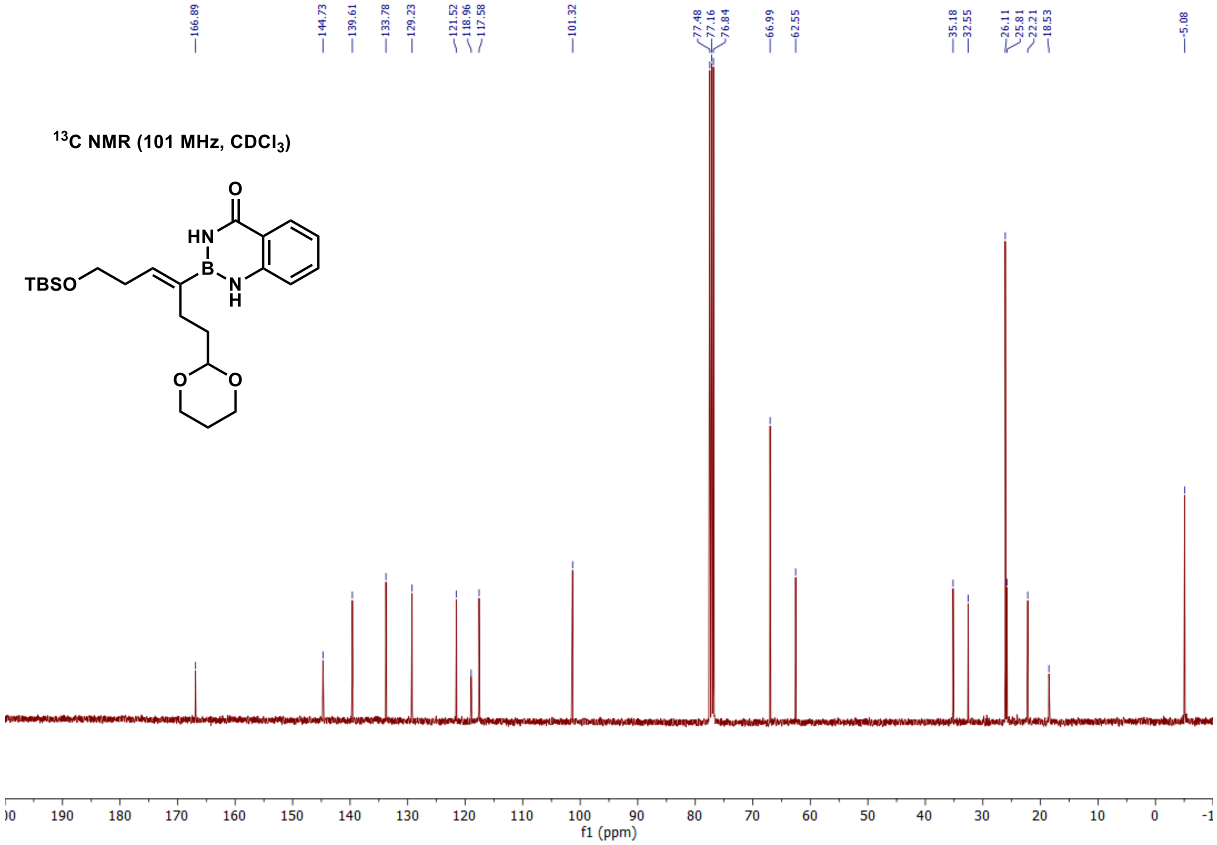

13C NMR (101 MHz, CDCl3)
TBSO
HN
B
N
H
O
166.89 144.73 139.61 133.78 129.23 121.52 118.96 117.58 101.32 77.48 77.16 76.84 66.99 62.55 35.18 32.55 26.11 25.81 22.21 18.53 -5.08
f1 (ppm)

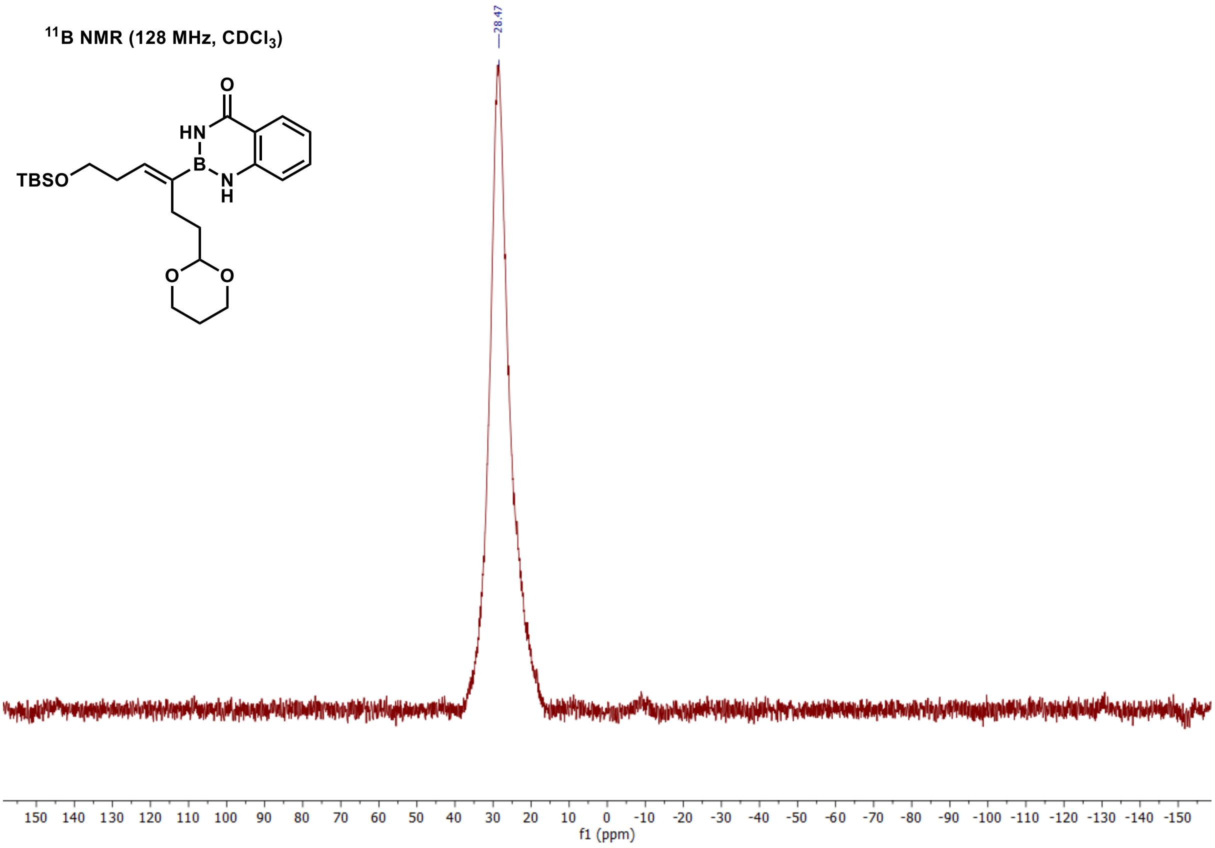
11B NMR (128 MHz, CDCl3)
O
HN
B
N
H
TBSO
O
O
—28.47
150 140 130 120 110 100 90 80 70 60 50 40 30 20 10 0 -10 -20 -30 -40 -50 -60 -70 -80 -90 -100 -110 -120 -130 -140 -150
f1 (ppm)

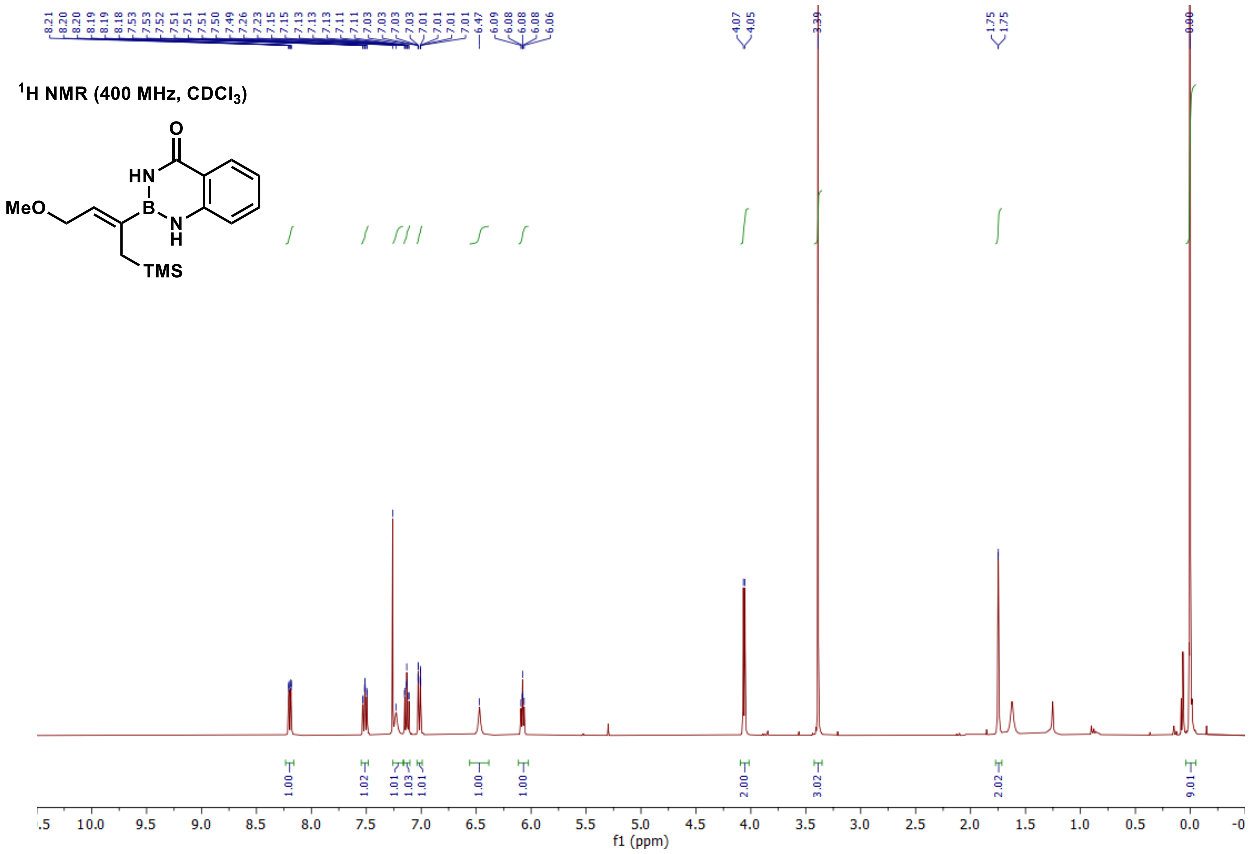
1H NMR (400 MHz, CDCl3)
MeO
HN
B
N
H
O
TMS
8.21 8.20 8.20 8.19 8.19 8.18 7.53 7.53 7.52 7.51 7.51 7.51 7.50 7.49 7.26 7.23 7.15 7.15 7.13 7.13 7.13 7.13 7.11 7.11 7.03 7.03 7.03 7.03 7.01 7.01 7.01 7.01 6.47 6.09 6.08 6.08 6.08 6.06
4.07 4.05
3.39
1.75 1.75
0.00
1.00 1.02 1.01 1.03 1.01 1.00 1.00 2.00 3.02 2.02 9.01
f1 (ppm)

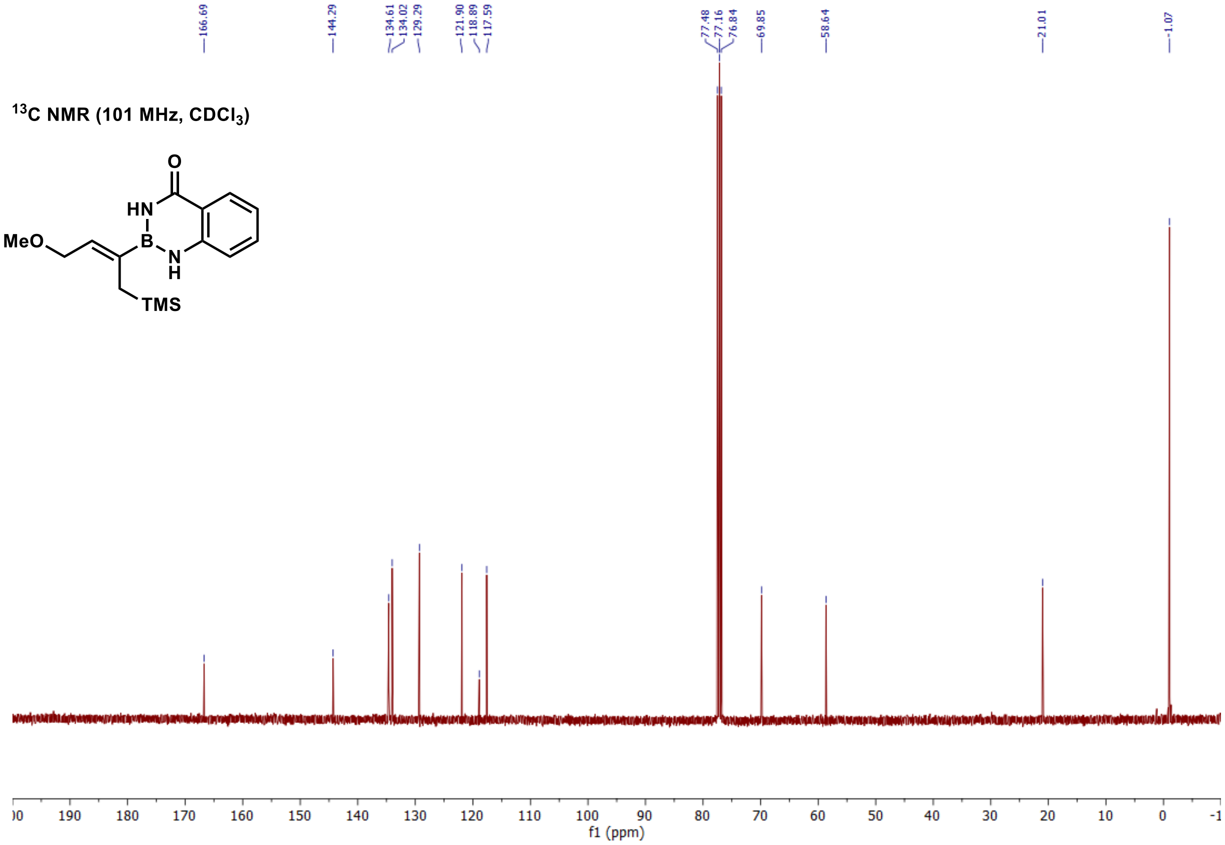
13C NMR (101 MHz, CDCl3)
MeO
HN
B
N
H
O
TMS
166.69
144.29
134.61 134.02 129.29
121.90 118.89 117.59
77.48 77.16 76.84
69.85
58.64
21.01
-1.07
f1 (ppm)

^{11}B NMR (128 MHz, $CDCl_3$)

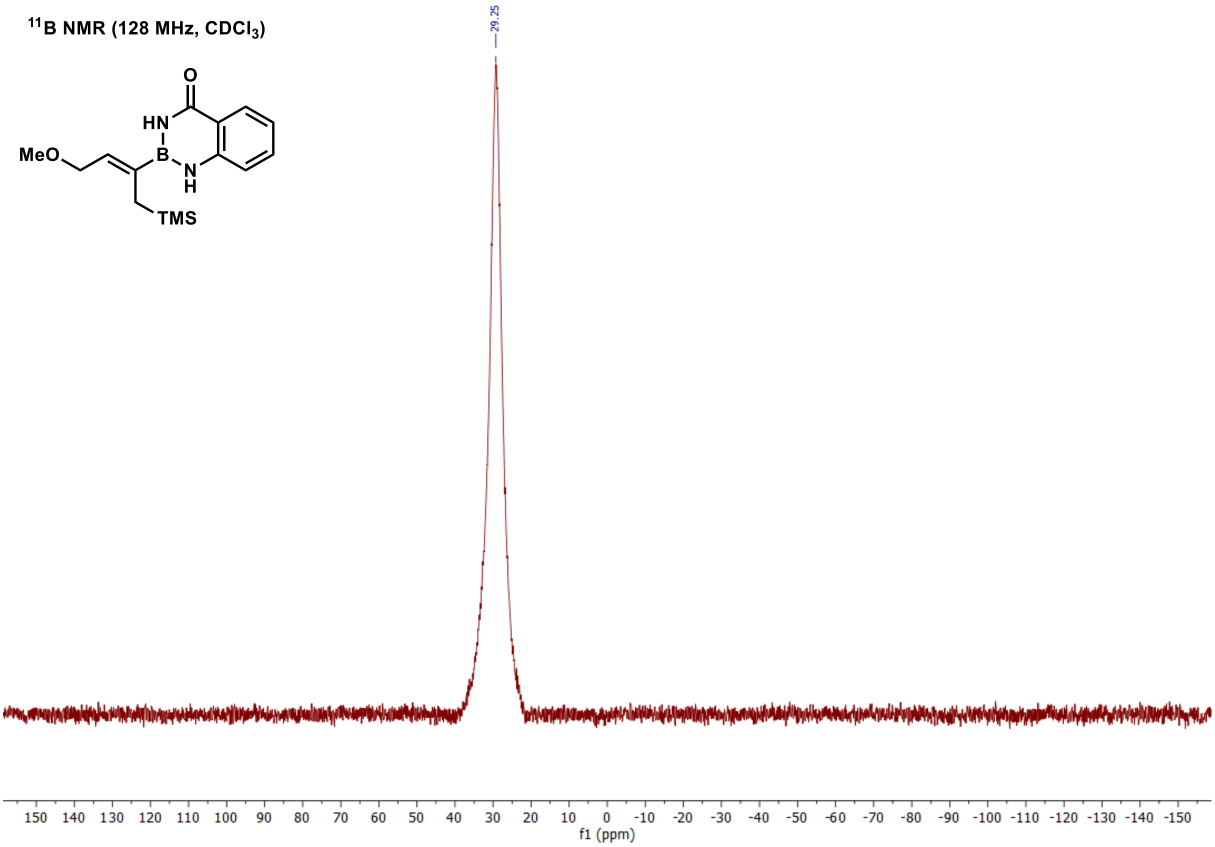

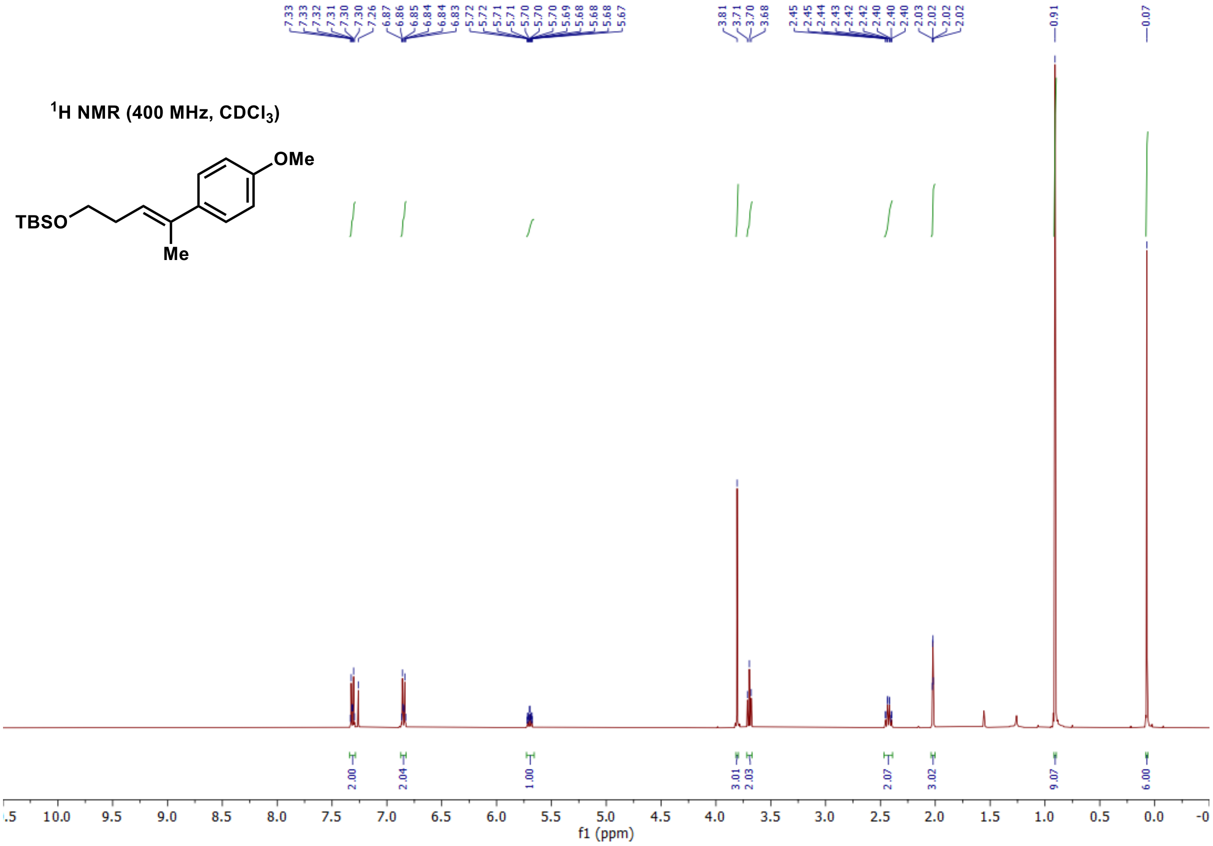

1H NMR (400 MHz, CDCl3)
OMe
TBSO
Me

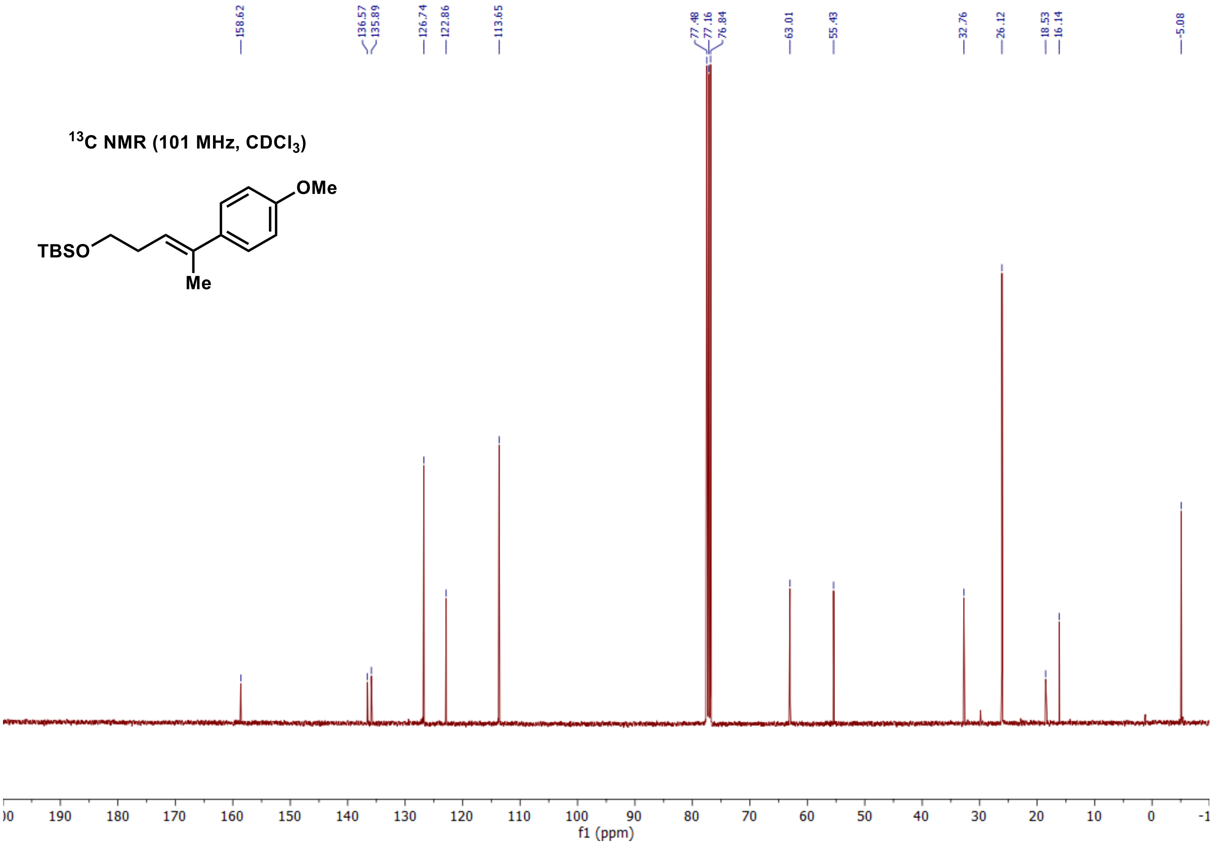

13C NMR (101 MHz, CDCl3)
OMe
TBSO
Me

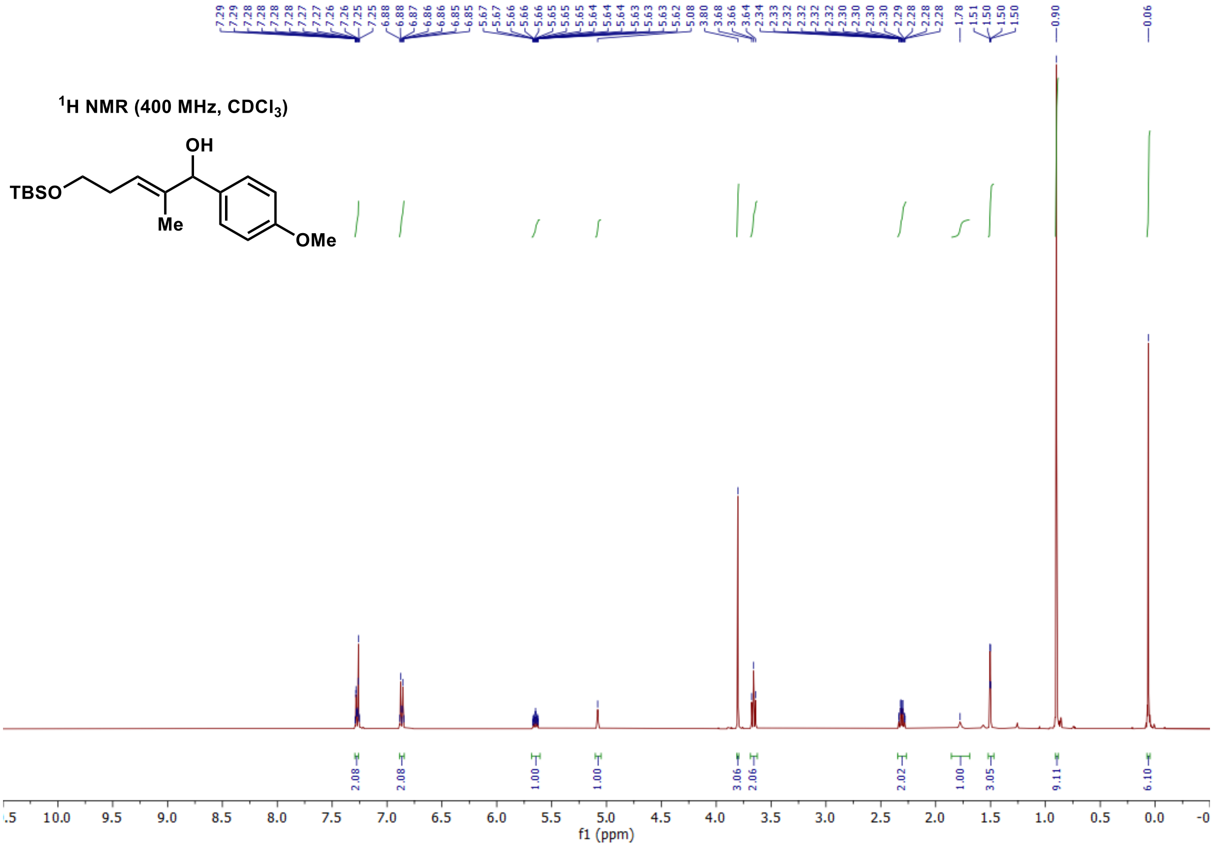
1H NMR (400 MHz, CDCl3)
OH
TBSO
Me
OMe
f1 (ppm)

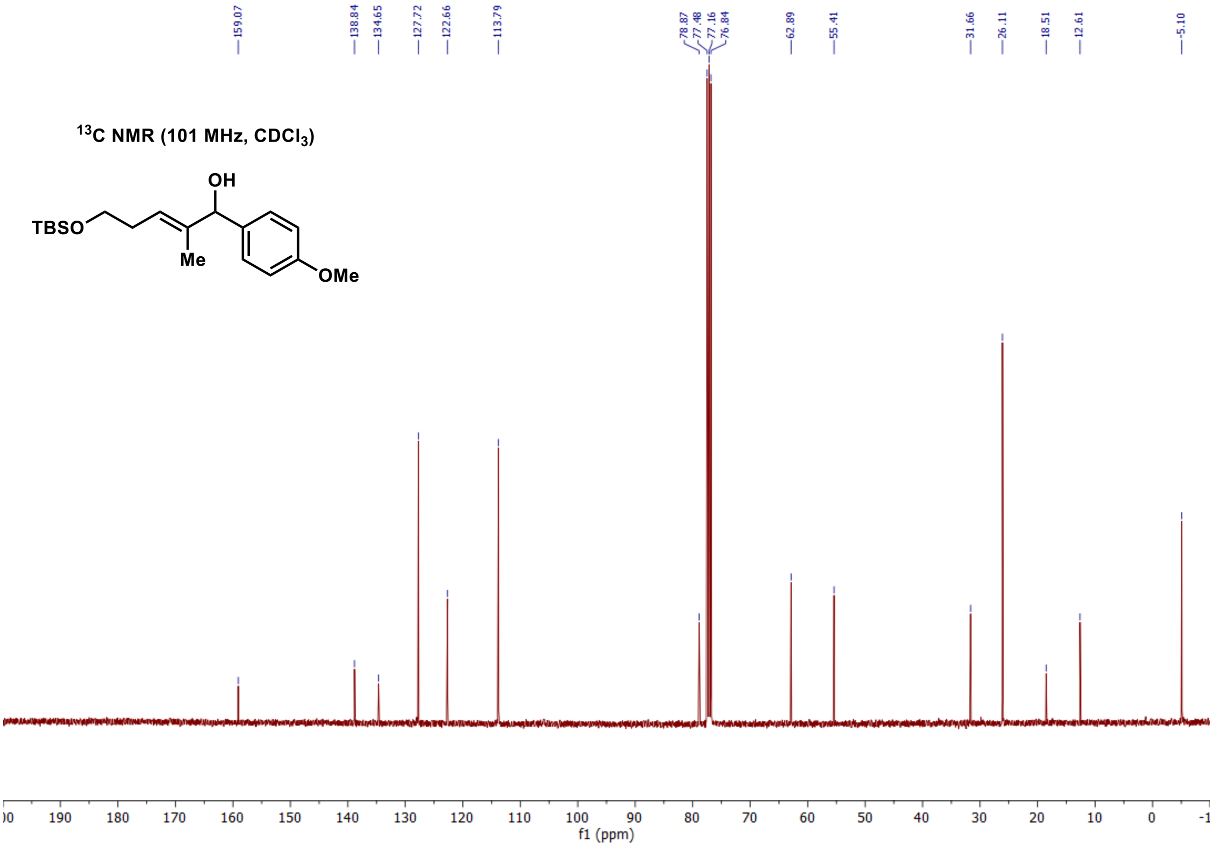
13C NMR (101 MHz, CDCl3)
OH
TBSO
Me
OMe
f1 (ppm)

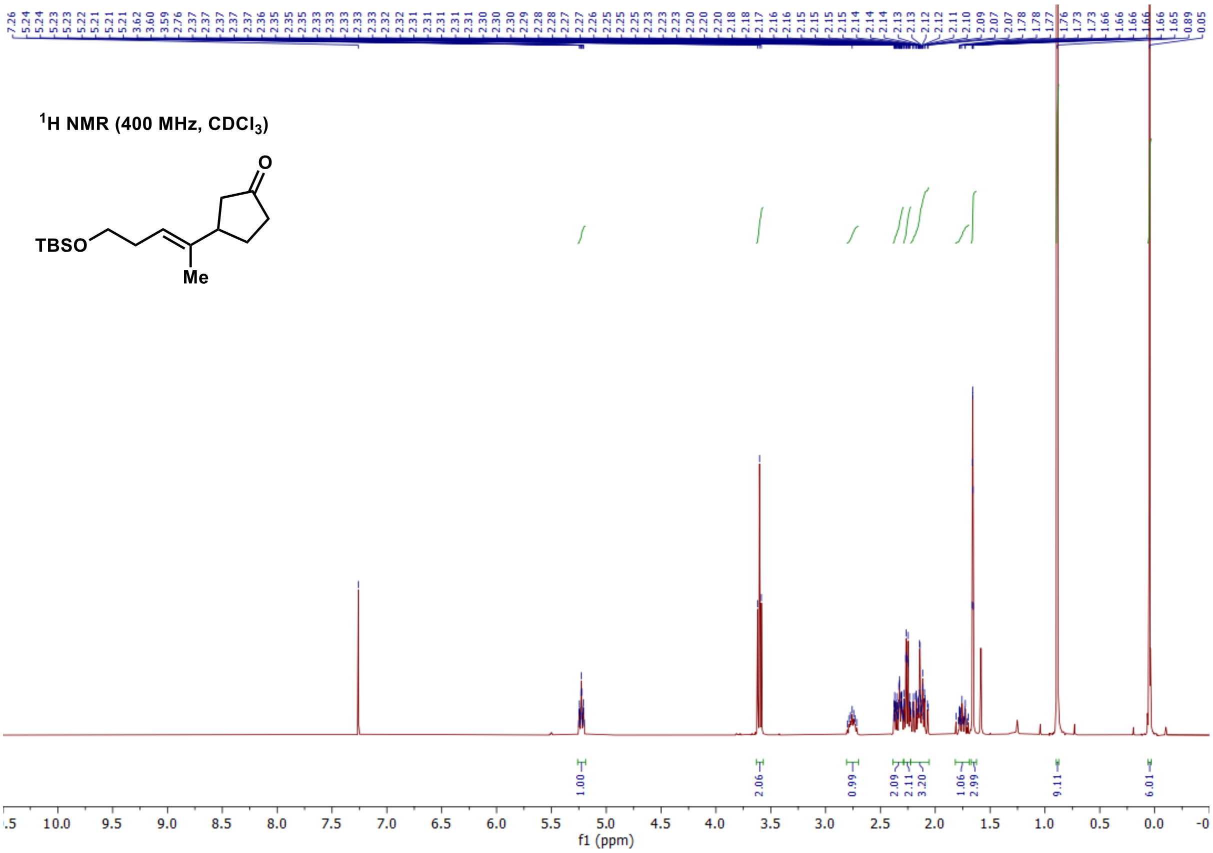
1H NMR (400 MHz, CDCl3)
TBSO
Me
O
f1 (ppm)

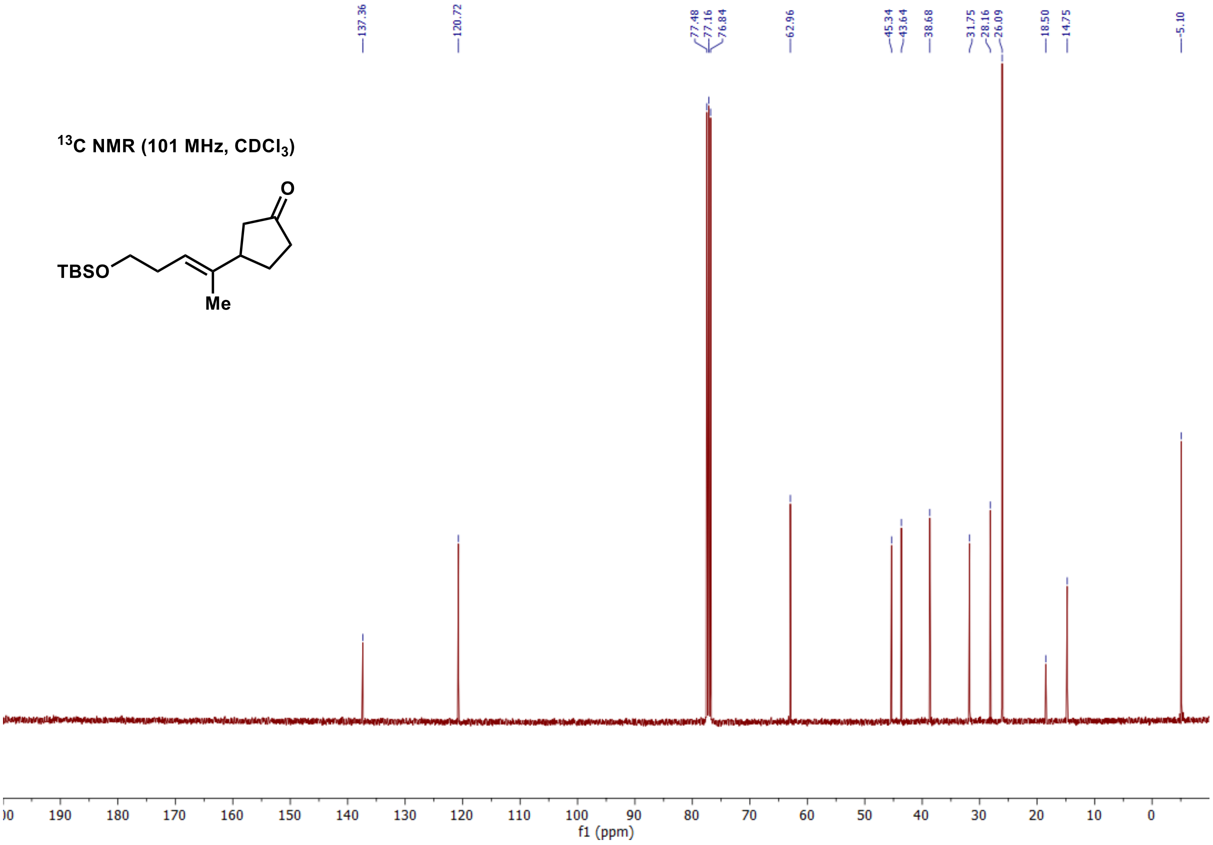
13C NMR (101 MHz, CDCl3)
TBSO
Me
O
137.36
120.72
77.48
77.16
76.84
62.96
45.34
43.64
38.68
31.75
28.16
26.09
18.50
14.75
-5.10
f1 (ppm)
